# Self-Assembled Monolayers and Solvent Composition Control the Orientation and Work Function of Pyrene-Based [7]Helicenes

**DOI:** 10.1021/acs.langmuir.6c01280

**Published:** 2026-09-29

**Authors:** Ivan Alic, Katsiaryna Kutsenka, Kristián Kříž, Lukáš Kalina, David Bína, Jan Hanus, Jaromír Kopeček, Anne de la Pena Lopez, Radek Litvín, Milan Mašát, Eva Kaletová, Dominik Farka

**Affiliations:** † Department of Chemistry, Faculty of Science, 48271 University of South Bohemia in České Budějovice (JU), Branišovská 1760, 370 05 České Budějovice, Czech Republic; ‡ Functional Molecules Group, Institute of Organic Chemistry and Biochemistry (IOCB), Czech Academy of Sciences (CAS), Flemingovo Náměstí 542/2, 160 00 Prague, Czech Republic; § Faculty of Chemistry, 48274 Brno University of Technology (BUT), Purkyňova 118, CZ-612 00 Brno, Czech Republic; ∥ Institute of Plant Molecular Biology, Biology Centre, Czech Academy of Sciences, Branišovská 31, 370 05 České Budějovice, Czech Republic; ⊥ Department of Materials Analysis, Institute of Physics (FZU) of Czech Academy of Sciences (CAS), Na Slovance 2, 182 00 Prague 8, Czech Republic; # Molecular Devices Group, Institute of Organic Chemistry and Biochemistry (IOCB), Czech Academy of Sciences (CAS), Flemingovo Náměstí 542/2, 160 00 Prague, Czech Republic

## Abstract

Self-assembled monolayers (SAMs) and solvent composition critically influence the orientation and electronic properties of functional organic molecules, paving the way for energy conversion and optoelectronic devices. PeakForce quantitative nanomechanics atomic force microscopy (AFM) was used to investigate pyrene-based 2*H*-pyran­[7]­helicene (*P*,*S*,*S*)-(+) and its mirror image, 2*H*-pyran­[7]­helicene (*M*,*R*,*R*)-(−), deposited on self-assembled monolayers (SAMs) with one of the following headgroups: adamantyl, phenyl, and trityl. By varying toluene content and dilution during spin-coating, we achieve changes to the topography, mechanical properties, and work function of our surfaces and are able to link these changes to the achievement of limited control over the modulation of the helicene orientation and gain insight into the self-assembly process on said SAMs. At high dilutions, we were able to detect distinct nucleation sites for helicene structures. These orientational changes and associated disorder correlate directly with work function modulation, suggesting a way and a mechanism for tuning electronic properties via these interfaces. Our findings hint at a simple processing strategy to engineer the molecular orientation and work function, advancing the design of helicene-based optoelectronic materials.

## Graphical abstract

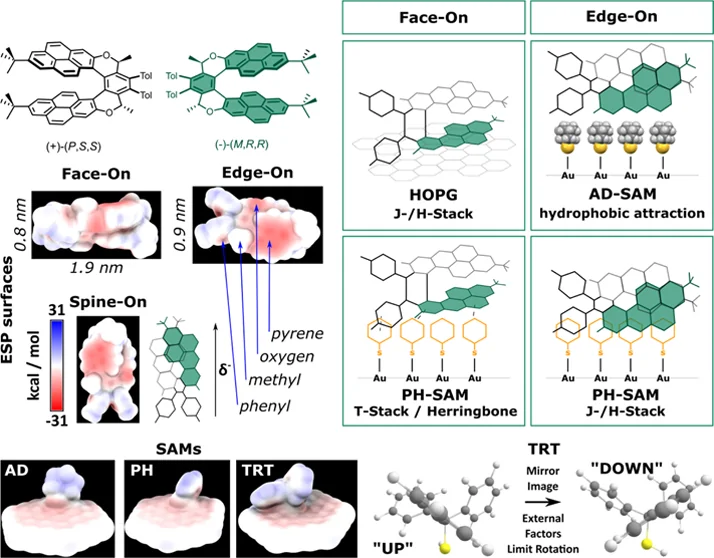

## Introduction

Helicenes, small, chiral, and typically conjugated molecules based on phenanthrenes, have gained a lot of attention in recent years.^1^ Their finite molecular conductivity and optical properties have attracted a lot of attention in specialized applications where chirality and charge-transport or optical properties are crucial: spin-filters,^2,3^ molecular inductors/capacitors,^4^ and naturally chiral emitters.^5,6^ The latter are useful in optoelectronic devices such as CP-OLEDs.^7^ As such, the effort required for their synthesis is warranted by these application prospects. Representative reviews summarize their synthesis, properties, and applications in materials science and optoelectronics.^8–10^

Surfaces play a crucial role for helicenes: they were shown to induce isomerization,^11^ enable chiral separation via self-assembly,^3^ and provide platforms for probing single-molecule phenomena.^12^ Analogous to other organic semiconductors, their work function—and possibly the energy levels in general—may be tuned in the same way^13–15^ and thus avoid the necessity to introduce foreign materials such as doping agents^16,17^ to the material matrix. A facile way to alter the surface properties can be found in self-assembled monolayers (SAMs);^18,19^ these were confirmed to alter the surface potential,^13^ structural orientation,^13^ and mechanical properties in organic semiconductors^20^ and thus avoid the need for dopants^17,21,22^ or chemical modification.^23^ By extension, the change in surface potential can be seen as a change in work function of these organic electrodes.

Identifying the cause of the above effects is often on the verge of, or even beyond, the analytical power of the current state-of-the-art instrumentation.^13^ We therefore employ helicenes both as a molecule of interest and as a probe to visualize their molecular orientation via changes in the surface mechanical properties. Helicene is mechanically anisotropic;^24^ hence, an edge-on^17,25^ orientation (Figure 1) yields minimal elastic deformation, whereas a face-on^17,25^ orientation (Figure 1) produces measurable elastic deformation. A way to gain such data is either via atomic force microscopy (AFM) by acquisition of force curves or via proprietary techniques, such as Bruker’s PeakForce quantitative nanomechanics (PeakForce QNM).^26,27^ In this way, we can distinguish topographical features by their mechanical properties such as adhesion, deformation, and dissipation (Figure S1a). The latter corresponds to the loss of energy of our cantilever and allows us to distinguish between plastic and elastic deformation.^20,26,27^ Additionally, the dipole moment of the helicene of our choice may reveal the orientation of its long axis (Figure 1): if oriented normal to the surface, Kelvin-probe force microscopy may reveal an increase or decrease in the measured surface potential. This corresponds with the dipole being oriented toward or away from the surface, respectively.^28^ Effectively, we have the tools to distinguish the four orientations of our helicene, three of which we observe in this work.

**Figure 1:**
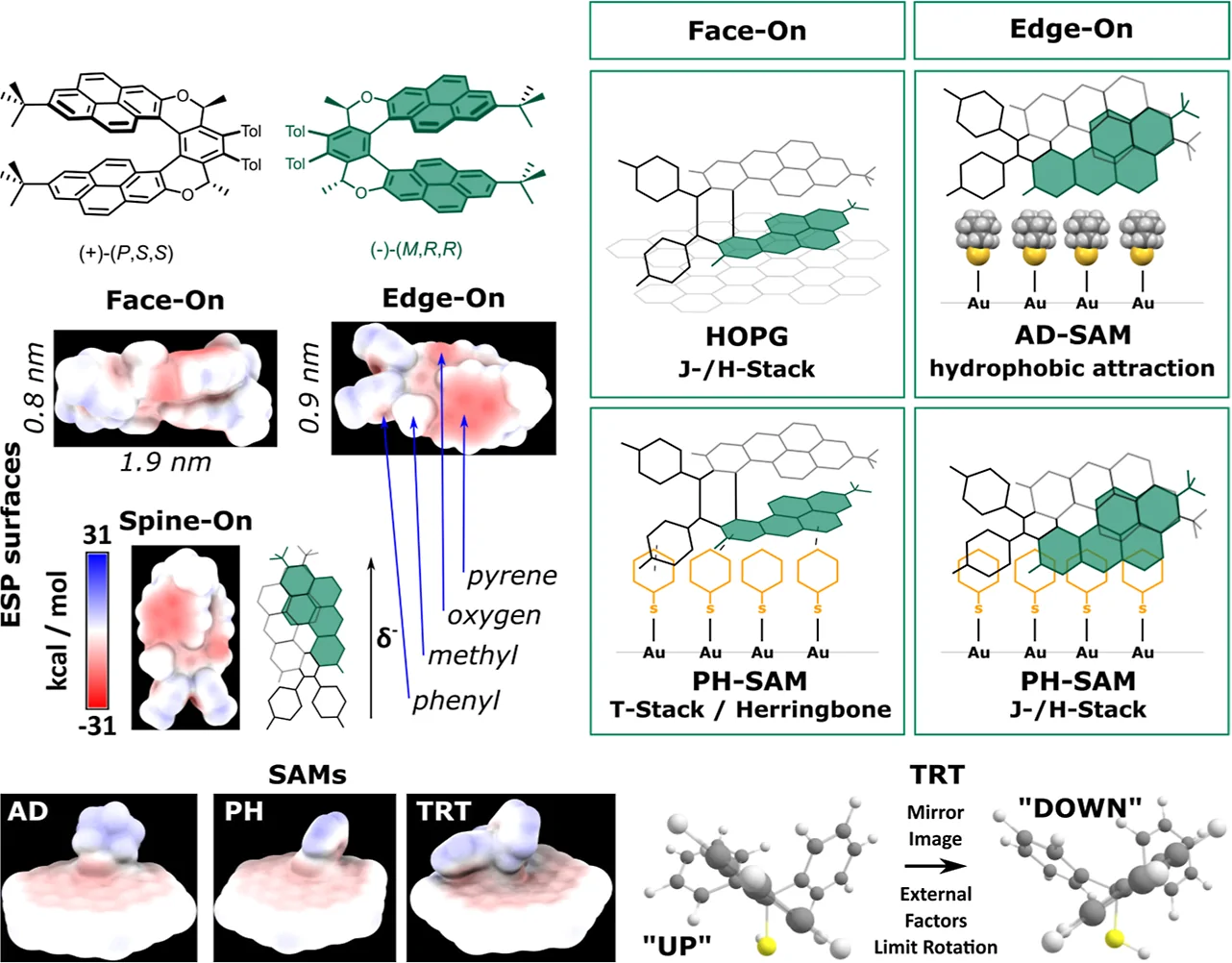
Helicene and SAMs used in this study. Structural formula and ESP surfaces for both P and M-enantiomers of the helicene and all three SAMs are given, as well as possible interactions and helicene orientations on the given surfaces. At last, the ESP surfaces of the three SAMs on gold are shown, and in the peculiar case of TRT-SAM, the possibility to adapt two enantiomers is illustrated. Simulations in different solvents reveal the possibility to favor one of them and yield the second enantiomer virtually impossible to achieve.

The helicene of our choice is 2*H*-pyran­[7]­helicene (*P*,*S*,*S*)-(+) (further “helicene” or *P*-enantiomer) and its mirror image, 2*H*-pyran­[7]­helicene (*M*,*R*,*R*)-(−) (further *M*-helicene). As all helicenes, this molecule resembles a helical coil spring and hence likely retains some of the anisotropic mechanical elasticity along the helix. Consequently, a force applied perpendicular to a surface will result in elastic deformation on a face-on oriented molecule, while close-to no deformation is expected for an edge-on oriented structure (Figure 1).

This helicene was chosen for its substituents, which ensure a stable helical chirality,^11,29^ while its two pyrene-subunits provide a wide base to stabilize the face-on orientation. Deposited via spin-coating,^30,31^ binary mixtures of dichloroethane with small amounts of toluene are employed to achieve aligned crystal growth.^32^

Flame-annealed gold surfaces^13,15,33^ treated with small, compact, and commercially available thiols with aliphatic (adamantyl, AD), aromatic (phenyl, PH), and stereodynamic, aromatic (trityl, TRT)^34^ headgroups act as our molecular templates. In the case of TRT, we use both enantiomers, P and M, in their pure and racemic form to investigate any chirality-induced effects caused by intermolecular interactions,^35^ including dewetting effects.^36,37^ Dewetting was observed in a single batch of gold; hence, the observed effect was likely induced by chiral gold structures rather than by processing. A more detailed discussion can be found in the Supporting Information (Figure S1b).^34,38–40^

Control over edge-on vs. face-on orientation (Figure 1) is broadly relevant beyond helicenes and can strongly influence device performance.^17,25,41,42^ At the same time, this way of investigation proves its worth also in terms of SAM characterization, which is often beyond the technical limits of X-ray based techniques such as X-ray reflection (XRR)^43^ and small-angle X-ray scattering (SAXS).^20^

Here, we address the question of how solvent composition and SAM chemistry jointly control the orientation of pyrene-based [7]­helicene at SAM-modified gold interfaces and how these structural changes correlate with work function modulation. Because of the very limited thickness of the layer, no measurable scattering intensity could be detected by the available SAXS measurements, preventing the acquisition of direct crystallographic or structural evidence. Consequently, the proposed orientation assignments were inferred indirectly from changes in the mechanical properties of the molecular ensembles and variations in the surface work function and should therefore not be regarded as absolute.

We combine PeakForce QNM AFM with KPFM to map orientation-linked mechanical responses^20^ and surface potentials,^13,18,44^ providing a processing strategy to engineer molecular orientations and interface energetics^14,18^ for organic electronics. To quantify the information from QNM PeakForce AFM, we employ an in-house-created script and employ uniform cut-offs to extract information on how the recipe composition influences the prevalence of a given orientation to find that dilution and higher toluene content foster the face-on orientation.

## Methods

### Material Synthesis and Characterization

2*H*-Pyran­[7]­helicene (*P*,*S*,*S*)-(+) and its mirror image, 2*H*-pyran­[7]­helicene (*M*,*R*,*R*)-(−), were synthesized as described elsewhere.^29^ Enantiomer 1 (E1) corresponds to the P helicity (internal batch number KK-459), and enantiomer 2 (E2_ corresponds to the M helicity (KK-461, deposited only on TRT-SAM).

### Thin Layer Preparation

Highly oriented pyrolytic graphite (HOPG) was prepared for spin-coating by cleaving off the top layers with adhesive tape and inspected for residual flakes, visually. Gold films were evaporated thermally in UHV via physical vapor deposition (PVD, instrumentation by Elettrorava company) on freshly cleaved mica and flame-annealed to improve flatness and clean its surface prior SAM-deposition. To form SAMs, solutions of the relevant thiols in ethanol (99.9% purity) were prepared according to established procedures.^13^

The helicene stock solution of 63 μM in dichloroethane (DCE) was prepared and further diluted to form the recipes shown in Table 1. Films were formed via spin-coating 20 μL of respective helicene solutions using a unified spin-coating recipe (90 s at 1500 rpm; 500 rmp/s acceleration, 50 μL solution per sample). All solvents were HPLC grade or better.

**Table 1:** Recipes R1–6 and Their Respective Concentrations Prepared from a Stock Solution (62.7 μM Helicene in DCE)^a^

Recipe #	c (helicene)/μM	Toluene/Helicene molar ratio	c (Toluene): c (DCE)/mol L^–1^: mol L^–1^	Toluene/DCE ratio
R1	63	0	0:12.7	0
R2	53	3.0 × 10^4^	1.7:10.6	0.15
R3	48	4.8 × 10^4^	2.3:9.6	0.3
R4	48	3.0 × 10^4^	1.4:10.6	0.15
R5	0.063	0	0:12.7	0
R6	0.063	3.1 × 10^4^	2:12,700	1.7 × 10^–4^

a Toluene/helicene ratio to visualize the molar ratio between both aromatic components. When combined with a specific surface, such as HOPG, R1 corresponds to HOPG1, etc.

### AFM/STM

All shown mechanical and KPFM and thickness measurements were performed on a Bruker Multimode AFM/STM system using SNL-10 AFM tips (cantilever D) and PFQNE-AL tips, respectively. The measurement modes were “ScanAssist HR in Air” (relying on the Quantitative Nanomechanics, QNM, upgrade) and FM-KPFM, respectively. For thickness measurements, the samples were scratched by the same tip in the contact mode with the maximal set point to remove the physisorbed material by friction.^45^ The reported thickness and presented line profiles are curated and representative for our measurements.

The evaluation of the mechanical properties was done first visually (qualitative evaluation). Later, quantification was done manually via Gwyddion software on a limited dataset including a selection of all SAMs. The latter data served as quality control for the Python script, which was prepared for this exact purpose. From said script, we were able to derive the area % covered by hard features (overlay of 25% lowest deformation AND 25% lowest dissipation) and elastic features (overlay of 25% highest deformation AND 25% lowest dissipation). This approach proved extremely resilient such that even in the presence of artifacts, the same surface would return comparable results, even in the presence of minor measurement artifacts.

KPFM measurements (work function) were reproduced and normalized to gold using a Keysight 5500 AFM in combination with Rocky Mountain tips (25Pt300B, full Platinum tips). The reported work function was calculated from the surface potential measured via KPFM, *U*(surface) as follows

$$\begin{array}{l} measured:\\ {U\left(surface\right)}_{Au-Pt}=\phi_{Au}-\phi_{Pt}\\ {U\left(surface\right)}_{X-Pt}=\phi_{X}-\phi_{Pt}\\ calculated:\\ {U\left(surface\right)}_{X-Au}={U\left(surface\right)}_{X-Pt}-{U\left(surface\right)}_{Au-Pt}=\phi_{X}-\phi_{Au}\\ \phi_{X}={U\left(surface\right)}_{X-Au}+\phi_{Au} \end{array}$$

In other words

$$\phi_{X}={U\left(surface\right)}_{X-Pt}-{U\left(surface\right)}_{Au-Pt}+\phi_{Au}$$

where ϕ_Au_ = 5.1 eV,^46–48^ ϕ_X_ is the work function of the material, and *U*(surface)_X–Pt_ and *U*(surface)_Au_–_Pt_ are the values measured for the material in question and gold (using the same tip in temporal proximity), respectively.

#### Contact Angle

Contact angle measurements were done using a CAM 101 (KSV Instruments, Ltd., Finland), taking 7–10 measurement points at two samples. The R5 and R6 samples typically show an incomplete coverage with helicene.

### Computational Methods

All ab initio calculations were performed in ORCA software.^49^

#### Helicene

Conformational sampling was done in CREST^50^ (no other options than default—XTB level) to find interactions between a helicene and DCE or toluene. Two conformations were chosen: the one with the lowest energy and another low-energy motif significantly different from the first.

The conformers were re-optimized at the ωB97X-V^51^ def2-SVP level, and the interaction energy and dipole moment were computed at ωB97X-V def2-TZVP with CP correction. Based on these, the ESP surfaces were computed, where their dipole is the dipole moment (field-independent basis, in Debye). Computations were performed at the PBE def2-SVP level with D3 correction.^52^

#### SAMs on Gold

Computations were performed at the PBE def2-SVP level with D3 correction.

The gold plate was restrained to the XY plane. This was done by fixing angles between 3 atoms in each of the 3 axes of the C3 golden plate symmetry to 180 deg. Doing so still allows some gold atoms to deviate from the planarity. The optimization, dipole values, and ESP listed below are on the same level of theory. The *z*-axis is perpendicular to the XY plane. In side-views, the representations were rotated such that the *X*-axis points right and the Y-axis points toward the back (skewed slightly to the right for visualization).

### XPS

The SAMs were analyzed via an Axis Ultra DLD spectrometer using a monochromatic Al Kα (hυ = 1486.7 eV) X-ray source operating at 75 W (5 mA, 15 kV) as described elsewhere to determine the thickness, coverage, and chemical identity of the SAM layers.^20,53^

To determine the stoichiometric C/S ratio in the TRT-SAM layer, a series of C 1s and S 2p XPS spectra were evaluated as a function of annealing temperature (room temperature, 60 °C, 70 °C, and 80 °C).

During the deconvolution of the C 1s spectrum, the overall signal was separated into components originating from the trityl molecular framework and components arising from external contamination. The total trityl carbon contribution, C_TRT_, included the main hydrocarbon peak located at 284.5–284.8 eV, which combines the sp^2^ aromatic carbons of the phenyl rings with a possible minor contribution from aliphatic sp^3^ carbons. This approach accounts for the physical resolution limit of the spectrometer for peaks exhibiting only small chemical shifts. To avoid overestimation of the carbon signal due to non-chemisorbed thiols (C–S^–^) or overlap with oxygen-containing contaminants, the C–S peak at 285.2 eV was constrained in the fitting model to the main hydrocarbon peak using the theoretical stoichiometric ratio of 1:18. This ensured that only the fraction of the C–S signal corresponding to the chemisorbed thiolate species (Au–S–C) bound to the gold surface was included in the calculation of the trityl contribution.

Peaks located at higher binding energies were excluded from the trityl framework calculation and assigned to adventitious contamination. The component at approximately 286.3 eV corresponds to C–O bonds, while the peak at 288.4 eV is attributed to O–C=O species. By excluding these oxygen-containing components, the calculated C/S stoichiometric ratio reflects only the intact trityl molecular layer with minimal distortion from surface contaminants.

The raw peak areas (*I*_raw_) were converted to corrected peak areas (I_corr_) using the Kratos instrument sensitivity factors, where RSF­(C 1s) = 0.278 and RSF­(S 2p) = 0.668. The corrected stoichiometric ratio is defined as follows

$$ratio\;C:S=\frac{\frac{I_{raw}\left({C\;1s}_{TRT}\right)}{0.278}}{\frac{I_{raw}\left({S\;2p}_{Au-S-C}\right)}{0.668}}$$

### SEM (Scanning Electron Microscopy)

All experiments were performed on a Tescan Fera 3 (Brno, Czech Republic) instrument at acceleration voltages of 5 and 2 keV.

### NMR

NMR spectra were measured on a Magritek Spinsolve 80 instrument in isopropanol (synthesis grade). These spectra were confirmed by a 400 MHz Bruker AVANCE III spectrometer (^1^H at 400 MHz ^13^C at 101 MHz). The spectra were referenced to the solvent signals δ­(^1^H) = 5.32 ppm, δ­(^13^C) = 53.84 ppm (CD_2_Cl_2_).

### Optical Characterization

Absorbance was measured by a Shimadzu UV-1800, 1 nm slit, 1 mm cuvettes (or 1 cm for samples 5 + 6). Steady-state fluorescence was measured by a Horiba Fluorolog-3 with double monochromators, with excitations at wavelengths of 350 and 405 nm.

For time-resolved fluorescence, a Picoquant FluoTime-300 instrument was utilized in combination with a 405 nm diode laser for excitation, IRF∼250 ps. Additional measurements in DCE/toluene mixtures were recorded on a **JASCO FP-8550 spectrofluorometer** equipped with a xenon lamp. The samples were excited at 370 nm, and emission spectra were collected from 400 to 800 nm using excitation and emission bandwidths of 5 nm, a response time of 0.5 s, a scan speed of 100 nm min^–1^, and a data interval of 0.5 nm. Medium detector sensitivity was used, except for the two most diluted samples, for which high sensitivity was applied.

The spectra of circular dichroism were measured with a Jasco J-715 spectropolarimeter. For the solution state samples, 2 nm slit, 2 mm cuvettes, giving A_374 nm_ ∼ 0.6–1, were used. Using a scan rate of 100 min^–1^, an integration time of 0.5 s, and a bandwidth of 5 nm, the solid state samples were measured. The samples were mounted on an in-house built holder. The measuring beam direction was parallel to the normal of the sample surface.

## Results and Discussion

Since our main focus was on the study of aromatic molecules interacting with aromatic surfaces, we decided to use dichloroethane (DCE) as the main solvent and subsequently dilute some of the helicene solutions with either toluene or DCE. In such a way, an improvement in solubility was achieved by the interplay of dilution and the addition of the aromatic solvent. This binary solvent has been previously studied in terms of its physicochemical properties, and it was determined that first all DCE evaporates before any measurable amounts of toluene leave the solvent.^54^ This results in six different recipes R1–R6, which are applied to the different surfaces: applied to the adamantyl-SAM, surfaces AD1–6 are achieved; when applied to the PH-SAM, surfaces PH1–6 result, respectively.

### PeakForce QNM-AFM

Across HOPG and three SAM chemistries (AD, PH, TRT), solvent composition (toluene: DCE ratio) and solution dilution modulate the helicene orientation: high toluene content and extreme dilution favor face-on elastic responses, whereas concentrated solutions promote edge-on, mechanically hard features. Figure 2 visualizes these trends; detailed images for each surface are given in the Supporting Information. These orientation changes correlate with contact angle variations and with systematic work function shifts measured by KPFM. The cumulative analysis (Figure 2b) is based off an overlay of the deformation with the dissipation channel (Supporting Information, 3D-images found in subfigures e,f).

**Figure 2:**
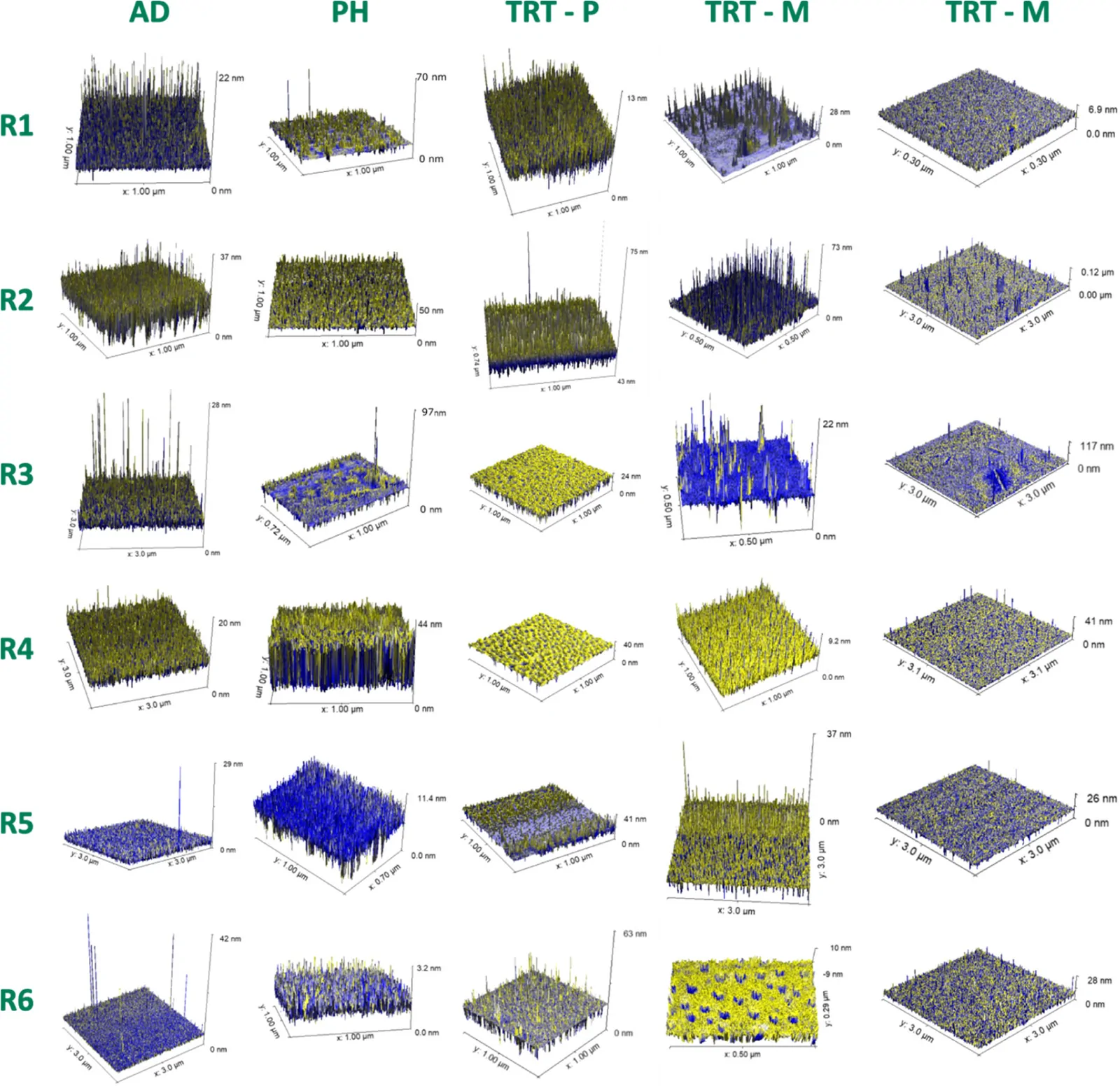
Examples for illustration. Deformation and dissipation channel overlay for all six recipes (R1–6) and three SAMs. Since trityl may adapt chiral properties,^38^ we investigated the behavior of the TRT-SAM with both the P- and M-enantiomer and their racemate and found different behaviors in all three cases, indicative of chirality induced in the surface-bound trityl groups. The deformation channel is given as topography, while dissipation is given as a color change from dark-blue/black to yellow/white. Where multiple assembly types were found, the following are shown: AD1 type 1, AD2 and AD4 type 2, PH3 type 2, PH4 type 1, TRT-P3 and 4 type 2, TRT-M1 type1, TRT-M3 type 2, TRT-M3 type 2. TRT-R3 and 4 type 2, TRT-R5 and 6 type 1. The full, detailed images can be found in Figures S2–S59, and the statistical evaluation can be found in the Supporting Information SII (enantio-pure) and III (racemate study).

#### HOPG

We started out with HOPG, yet we were immediately confronted with challenges of low adhesion to the surface (Figures S2–S5).

For the stock solution diluted by DCE, recipe 1 (HOPG1), we repeatedly observed weak adhesion, which resulted in low-quality images (Figure S2). Elevated features were correlated with low adhesion, high deformation, and low energy dissipation. We therefore conclude that the helicene observed assembles on the surface in a face-on orientation.

For the stock solution diluted by toluene, recipe R2, we observed a clearer image, yet only very small features without distinct deformation/dissipation patterns (Figure S3). This suggests that the addition of toluene favors an edge-on orientation, possibly due to a solvation shell of toluene around the aromatic parts of the helicene molecules. Also, a clearer image was observed, suggesting an improved adhesion.

Further dilution by toluene (R3) or DCE (R4) resulted in no deposition, reflecting poor adhesion of the helicenes on HOPG (Figures S4 and S5). Therefore, we concluded that further investigation should focus on self-assembled monolayers.

#### AD-SAM

Detailed representative measurements for the QNM of AD-SAM can be found in Figures S6–S11, and representative height profiles for the respective thickness can be found in Figure S12. Examples of overlays of the deformation and dissipation channels are found in Figure 2, and in Figure 3a, the statistical evaluation is found.

**Figure 3:**
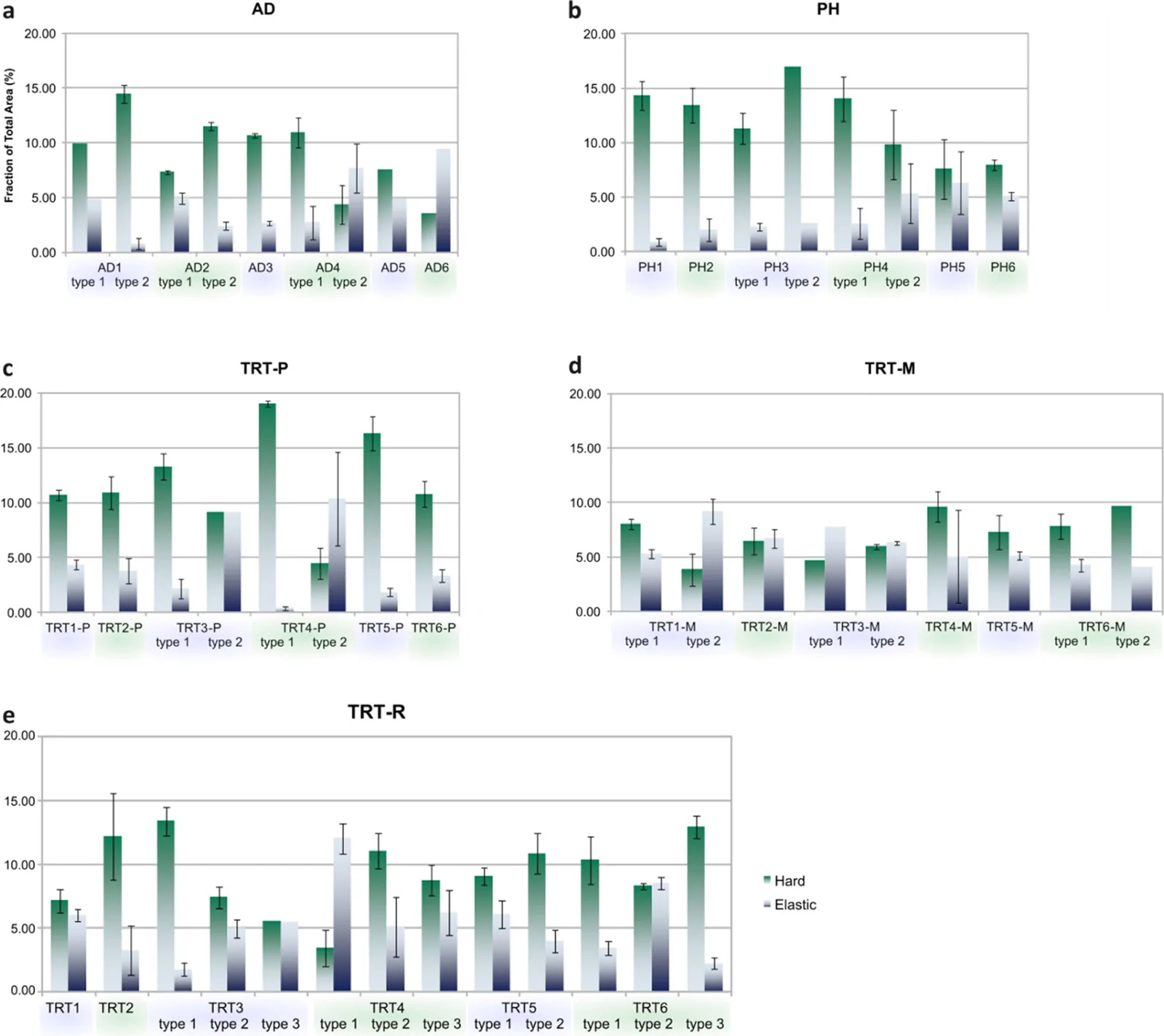
Statistical evaluation of QNM measurements focusing on the areal ratio of hard vs. elastically deforming features. (a–f) AD-SAM, PH-SAM, TRT-SAM with P-helicene, TRT-SAM with M-helicene, and TRT-SAM with a racemic mixture of helicenes, respectively. Individual recipes are grouped by color for better readability as some SAMs resulted in various surface assembly types. All details for individual measurements can be found in the Supporting Information.

In general, the presence of toluene resulted in more deposited material (Table 2). At the two highest concentrations (AD1 and 2), the structural features grew larger, while both recipes lead to a baseline thickness of about 2 nm. Statistically, more harder features were found in AD1 (10 and 14% with respect to assembly type vs. 7 and 12% for AD2; Figure 3a). This correlates with fewer elastically deforming areas for AD1 (5 and 1% vs 5 and 3% in AD2). The baseline thicknesses observed for the two recipes vary slightly. Given the molecule aspect ratio, we interpret a face-on orientation for AD1 and a spine-on orientation for AD2 (the helicene long axis and its dipole oriented perpendicular to the surface).

**Table 2:** Feature Height and Thicknesses of Helicenes Deposited on Various SAMs via Different Preparation Recipes (1–6); in the Case of the Pro-chiral TRT-SAM, Both Enantiomers, P and M, Were Tested^a^

		P-Enantiomer		M-Enantiomer		Racemate	
Surface	Recipe	Feature Height/nm	Film Thickness/nm	Feature Height/nm	Film Thickness/nm	Feature Height/nm	Film Thickness/nm
AD	1	3.0	1.6	n/A		n/A	
	2	7–13	2.0				
	3	3.0	2.0				
	4	2–3	2.5				
	5	3	1.5				
	6	3 or 10	<1				
PH	1	<14	1	n/A		n/A	
	2	<15	1–2				
	3	8–12	1.0				
	4	<6	0.5–1				
	5	<3	1				
	6	<3	<1				
Trt	1	<4	1.0	6–14	3.0	2–8	1.6
	2	<4	1.6	5–10	1.0	8–23	2.0
	3	<4/<5	1.0	<3	1.0	4–13	1.0
	4	<5/<15	2–3	4–5	2.5	1–33	<1
	5	<5/<10	<1	5–14	<1	5–33	1.0
	6	<7	<1	<7	<1	2–20	<1

a For TRT-P 3–5, the second value corresponds to samples where dewetting was observed.

The next pair of recipes, AD3 and 4, compare the effect of increased toluene vs dilution by DCE.

In AD3, we observe a single assembly type, which carries an uncanny resemblance to AD2 type 2 and AD4 type 1. This is indicative of toluene enforcing the formation of harder features (edge-on, 11–12%) and suppressing the formation of elastic features (face-on, ∼3%). AD4, however, exhibits a second assembly type, where the edge-on to face-on ratio is inverted at 4:8 area %. It hence appears that the more DCE is available, i.e., the longer DCE is present before toluene starts to evaporate during the spin-coating process, the more face-on orientation is achieved—as long as a little toluene is present to achieve this change.

This trend is confirmed in the highly dilute recipes, AD5 and 6. Devoid of toluene, AD5 yields similar behavior to AD1, with a consistent 8:5 ratio. Once traces of toluene are present (AD6), the behavior mirrors that of AD4 type 2, and an areal ratio of 3:9 in favor of face-on features is achieved.

From that, we can derive the following trends for the AD-SAM: A combination of 15% or less toluene at concentration levels below 50 μM result in prevalence of a face-on oriented helicene, which is not achievable purely by dilution. In terms of thicknesses, solutions with toluene result in higher baseline thicknesses at high concentrations, yet with dilution, the trend quickly reverses, and at extreme dilutions, toluene prevents the formation of a consistent base-layer as clustering into features is preferred. This suggests that the two solvents are involved in the establishment of the initial contact between the surface and helicene and that toluene is required in order to allow the assembly of helicene–helicene contacts.

#### PH-SAM

Detailed representative measurements for QNM of PH-SAM can be found in Figures S13–S22, and the height profiles for the respective thickness can be found in Figure S22.

In general, the binary solvent leads to smaller thicknesses of the baseline molecular layer covering the SAM as clustering into tall features up to 15 nm in height was preferred (Table 2). The thin overall background is likely to minimize surface energy, i.e., due to a mismatch in lattice parameters of the helicene^29^ vs the PH-SAM. The statistical evaluation of hard vs. elastic surface area can be found in Figure 3b.

At the highest concentrations, PH1 and 2, we observe no differences between recipes, with prevalently edge-on assembly. In area %, we observe ratios of 14:1 and 13:3, respectively. Baseline thicknesses of 1 and 1–2 nm were observed.

Once diluted, AD3 and 4 display two variations of assembly, each. All of them show prevalence of hard features at about 10%, and most of them exhibit few elastic features all about 3%. In PH4 assembly type 2, elastic features cover over 5% of the total surface area.

This trend is elevated only at higher dilutions. In PH5, hard and elastic features almost equalize at a 7:6 area % ratio. In PH6, where traces of toluene are present, this ratio sinks to 7:5. The overall baseline thickness and feature height indicate that at these extremely low concentrations toluene prevents immobilization of the helicene molecules; i.e., it behaves similar to a drug blocking the binding site of the molecule in question.^55^

This is highly different from the situation at higher concentrations, such as in the case of AD2 and AD3, where larger thicknesses would result for similar concentrations, as in their toluene-depleted counterparts. Furthermore, at the highest concentrations tested, the complete absence of toluene was correlated with the virtual absence of elastic features.

At this point, a peculiarity of PH3 and 4 should be noted: out of all recipes, these proved the hardest to measure, with frequent tip changes, artifacts caused by clearing of complete areas by attachment to the tip, etc. This peculiar behavior was encountered for subsequently measured aromatic SAMs as well and is indicative of poor adhesion, and will be discussed at a later point in this article.

#### TRT-SAM: Two Enantiomers and Their Racemate

Due to the stereodynamic^34^ nature of the TRT headgroup, we investigate both the P and M enantiomers in their enantiopure form and as their racemate (indicated as TRT-P, -M, and -R, respectively).

### TRT-SAM-P

Detailed representative measurements for the QNM of TRT-SAM can be found in Figures S23–S26 and S28–S31, and the height profiles for the respective thickness can be found in Figure S32.

In terms of thicknesses and feature height, we observe the greatest baseline thickness in TRT-P4 (2–3 nm, Table 2) and likewise the greatest feature-height of up to 15 nm. In all other cases, the thicknesses were dwarfed by those of the nonstereodynamic^34^ PH SAM. This behavior is later reflected in dewetting behavior, likely caused by a local mismatch in surface and helicene chirality—likely induced by the underlying gold. The statistical comparison of surface area covered in hard and elastic features can be found in Figure 3c.

Just as for the PH-SAM, TRT-P1 and -2 proved highly similar as concentration proved to have the bigger influence, rather than solvent composition. In both cases, a singular assembly type dominated by hard, edge-on features was observed, with only about 4% of the area covered in elastically deforming, face-on features. However, this already presents an increase of the area covered in the elastically deforming surface area.

For TRT-P3 and -4, two assembly types were observed. Dewetting, where hard features dominate (typically where the helicene layer was missing and the SAM was exposed), presents the first. Attempts to reproduce this behavior with a different batch of gold samples failed; hence, this was likely caused by chiral features on the gold rather than by an intrinsic chiral behavior.^56,57^

The second type is visually similar to other measurements in this paper and is reflected in a parity of hard and elastic areas for toluene-rich TRT-P3 and a 4:11 area % ratio found for TRT-P 4. The latter shows a substantial variation between samples, yet none of the samples showed an inversion of the trend.

At extreme dilutions, in TRT-P5 and 6, we observe a reversal to behavior more similar to that of TRT-P1 and 2, yet more distinguished as the hard edge-on oriented helicene covers 12 and 17% of the surface area and utterly dwarves the presence of the elastic, face-on oriented helicene at 2 and 3%, respectively. It appears that where dilution is needed to activate the PH-SAM to achieve a face-on orientation, it becomes more a question of the toluene concentration for the TRT-SAM in the presence of toluene. We later investigate this computationally and are able to link this to the interaction between toluene and the trityl-SAM (see the Computational section of the Supporting Information, Figures S65–S68).

### TRT-SAM-M

The M-enantiomer behaves quite similarly in terms of observable film thickness to the P-enantiomer (Table 2 and Figures S32 and S33). However, the image differs when the film features and its mechanical properties are concerned (Table 2 and Figure 3d). Further, in contrast to the P-enantiomer, no dewetting was observed for this enantiomer. These issues may be also magnified locally by the solvent ratio; either by effective achievement of antisolvent behaviour or the SAM itself was affected thereby. Where larger areas of a specific chirality of the SAM emerge, toroidal structures are observed. This question is further investigated in the computational and optical characterization parts yet could not be confirmed.

Furthermore, dewetting occurred in a single set of samples only. All of these were based on a single batch of evaporated gold samples. Hence, we consider this to originate from a transfer of chirality from the gold toward the TRT-SAM.^56^ Spectroscopic characterization of the solutions and solid-state samples was insufficient to determine any other source (see Supporting Information, Figures S60–S64).

Detailed PeakForce QNM-AFM images can be found in Figures S34–S42, while the evaluation of the mechanical properties can be found in the Supporting Information.

For the first time in this study, recipe R1 resulted in two distinct assembly styles: type 1 with an 8:5 and type 2 with a 4:9 ratio of area % covered in hard and elastically deforming material. Moreover, while type 1 matched the observations for TRT-P 1, type two was more pervasive, i.e., a face-on orientation was preferred. In any case, the thickness observed was three times that of the P-enantiomer, i.e., 3 nm. This leaves the possibility that the helicene could hypothetically be oriented perpendicular to the gold surface. The most important difference was however found in the feature height, where 6–14 nm tall features were observed.

For TRT-M 2, on the face-on dominant assembly type was observed at approximately a 6:7 ratio. Here, the baseline thickness mimicked that of the P enantiomer, yet again much taller features resulted (Figure 3c,d).

For the two slightly diluted recipes, TRT-M3and 4, we did not observe any issues in terms of dewetting and also close to no anomalies in terms of tip changes. This was surprising as these two surfaces should not differ and no global chiral features could be observed. Consequently, we consider the possibility that properties of the underlying substrate are involved either in the suppression or emergence of some of these behaviors. In TRT-M3, we observe two assembly types, which result in an either 4:8 or 6:6 ratio of area % covered by hard and elastic features, respectively. The former was found only in one instance and is most similar to the elastic assembly type found for R1 of this series, despite these two recipes being the most different in terms of toluene content.

For TRT-M 4, on the contrary, only a single assembly type was observed, the diluted variety of R2 exhibiting harder features than the previous samples at a 9:5 ratio (Figure 3d). Interestingly, with dilution of the solution and less helicene present in comparison to both solvents, more plastic deformation was observed, i.e., a cofunction of helicene concentration, the presence of toluene, determines whether neat self-assembly results.

The above observation was repeated at extreme dilutions in the absence of toluene (TRT-M5).

The extreme dilutions, TRT-M 5 and 6, resulted in the dominance of hard features over elastic ones (Figure 3d).

R5 results in a single-assembly type with a 7:5 ratio. Interestingly, the helicene molecules prefer to accumulate in tall features up to 14 nm in height. R6 results in two assembly types, the first of which mimics the observations for the toluene-free recipe (7:4 ratio). The second exhibits an increase of hard features to arrive at an area % ratio of 9:4. Both types resemble the behavior of the other enantiomer in terms of topography, including feature height.

Throughout this work, we repeatedly observed the accumulation of plastically deforming material around hard or elastically deforming features. Our immediate suspicion was the possibility of mushroom-like structures.^57^ These could form to minimize energy through avoidance of deposition on the surface: fist, growth in the *z*-direction forms the initial structure and only later does lateral growth occur via the strong, intermolecular interactions between aromatic (pyrene–pyrene) or aliphatic (methyl–tertbutyl) functional groups. These would easily break off when strained mechanically by the impact of the AFM cantilever stylus. However, closer inspection via SEM did not confirm this across all measurements (Figure S75). We therefore took detailed images of TRT6-M (Figure S42). Said behavior was observed to coincide with the rims of the elevated structures (Figure S42a). These thin areas did indeed correlate with high deformation and high dissipation but are part of the topographic elevation (yellow peaks, Figure S42e–g). As before, these appear in the vicinity of the ordered, in this sample’s case, face-on oriented helicene structures.

What is more important, these detailed images revealed flat, small structures of similar sizes, which we interpret as nucleation sites.

#### Nucleation Sites on PH, TRT-P/-M

The observation of nucleation sites in TRT6-M caught our attention (Figure S42). We therefore set out to find evidence of similar occurrences on other, highly dilute surfaces. We employed a high-resolution tip and uncovered similar structures in PH5 (Figures 4a,d,g and S43 and S44) and TRT5-P (Figures 4b,e,h and S45 and S46), in addition to TRT6-M (Figure 4c,f,i) To find these, a part of the surface with fewer large structures had to be identified, and the space in-between prominent features had to be monitored. Despite our efforts, we have found and identified nucleation sites in these three cases only.

**Figure 4:**
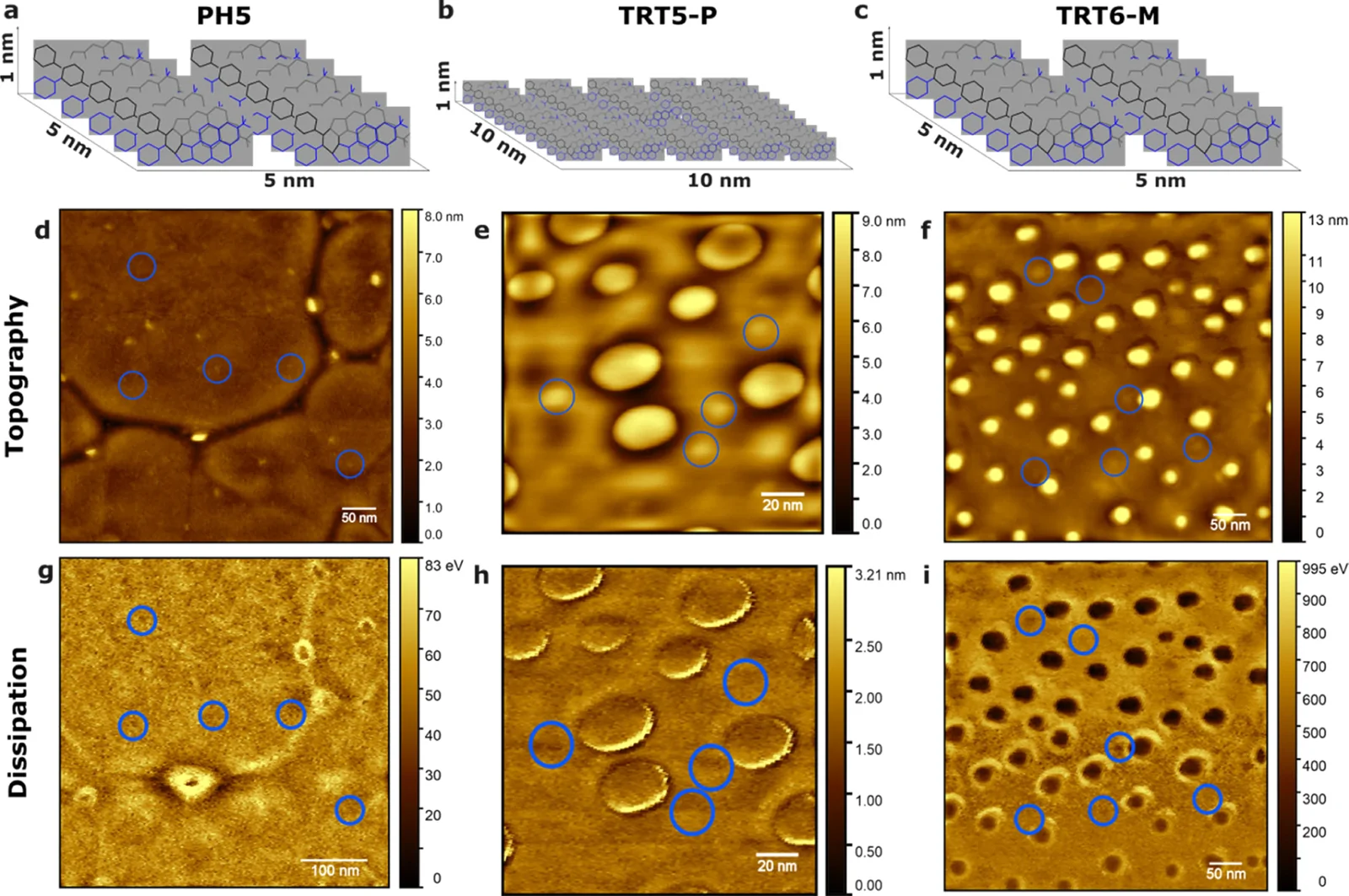
Nucleation sites of three surfaces (highlighted with blue circles). (a,d,g) Illustration of the molecular arrangement, topography, and dissipation channel for PH5, (b,e,h) TRT5-P, and (c,f,i) TRT6-M, respectively.

On PH5, the overall behavior matched previously observed images (Figure S43). Focusing on a small, flat part of the surface, we found several features of a lateral size of about 5 nm. This corresponds to about two stacks of 5 molecules, assuming a rectangular arrangement (Figure 4a). The topography channel revealed that these nucleation sites were 1 nm high, i.e., an edge-on monolayer formed initially, as suggested by previous results. This was confirmed by the dissipation channel (Figure 4g) as these features proved to be very hard and low in dissipation (Figure S44).

On TRT5-P, we previously observed issues with frequent tip changes that remained elusive. This could be related to the significantly smaller tip radius of the high-resolution tips. Again, elastic features dominate the surface in the absence of toluene (Figure S45). In detail, among the dominant features (Figures 4e and S46), nucleation sites of about 10 nm lateral were found, about 1 nm tall. This would correspond to a 5-by-10 molecular arrangement, assuming a rectangular alignment. The dissipation channel indicates an edge-on arrangement (Figure 4h), which is further confirmed when correlated with the dissipation channel (Figure S46e,f). In the more prominent, about 50 nm in diameter, features, we observe further lateral growth before a second, poorly cohesive layer is deposited.

This arrangement is quite different from the findings for TRT6-M, where significantly smaller features were found to be nucleation sites. Similar to PH5, we interpret these as 5-by-2, edge-on-arranged molecular structures. This is quite likely a consequence of the M enantiomers’ improved interaction with the TRT-SAM in this area. As a consequence, we observe smaller accumulations of structurally cohesive material being deposited during the spin-coating process. In the case of the mismatch, much larger structures are needed for the molecules to adhere to the surface.

### TRT-SAM-R (Racemate)

Detailed PeakForce QNM AFM images of the racemate deposited on the TRT-SAM can be found in the Supporting Information (Figures S47–S58), with the profile scans of the height measurements found in Figure S59. On the contrary to pre-existing images, long, linear objects were observed in some cases (Supporting Information, Figure S51). Similar structures were previously observed by de Feyter and colleagues for helicene racemates, while ringlike and C-shaped structures were known for enantiopure helicenes.^58^ These long, linear objects were highly adhesive in all cases but only found in a few samples; hence, both enantiomers prefer separation over intermixing on the surfaces. This might indicate a future use of the TRT-SAM as a tool for the separation of helicenes; however, we admit that such an application is far-fetched and at this point in no way feasible.

In terms of thicknesses, we observe similar values for the base layer to those observed in TRT-P. The elevated topographic features, however, resulted in heights that dwarfed anything else observed in this study, with maxima of up to 33 nm.

For the two highest concentrated solutions, TRT-R1 and 2, we observed two cases of behavior similar to that of TRT-P: In both cases, a single assembly type was observed; in both, hard features were dominant, nominally at 8:6 and 12:3 ratios (Figure 3e).

For TRT-R3 and 4, we observed the largest degrees of variation with three assembly types observed (Figure 3e).

For R3, three types of areas were observed: those almost devoid of elastic features, areas of mixed, and areas covered with hard and elastic materials at equal rates. This reflects a separation of both enantiomers as they present a mix of previously observed ratios.

R4 type 1 was dominated by the elastic, face-on-oriented helicene (3:13), while the other two were both dominated by hard features at ratios of 12:5 and 8:6. The separation into types 2 and 3 emerges as the latter is significantly more adhesive. Oddly, type 1 does not correlate with any of the previously observed behaviors. We therefore consider that a consequence of the steric differences between both enantiomers: the edge-on oriented material likely attracts its own enantiomer only. The flat pyrene rings, on the other hand, may not have these limitations.

The most dilute racemic mixtures, TRT-R5 and 6, again mostly reflect combinations of the two enantiomers.

TRT-R5 is split into two assembly type areas covered in hard and elastic material at ratios of 9:7 and 11:3. Once traces of toluene are present (TRT-R 6), we again observe both images of mixed areas (11:3, type 1) and those devoid of the elastically deforming material (13:3, type 3). As in R3, a balanced coverage was observed, as well (type 2).

These balanced and predominantly elastic areas were observed only in the presence of toluene in racemic mixtures. The film formation clearly undergoes Stranski–Krastanow^59^ assembly and is not recognizable purely from topographic images. We therefore suggest that these elastic features present racemic mixtures and are likely separated layer-by-layer to achieve round rather than high-aspect-ratio features.^58^

#### Optical Characterization

Solutions of the P-enantiomer and the racemate were investigated optically in order to reveal possible interactions with the solvents. Investigations in dichloromethane:toluene mixtures and dichloroethane:toluene mixtures were performed for the pure enantiomers, representing solutions used in recipes R1–R6. The resulting absorption, emission, and CD spectra of all samples were identical within experimental accuracy (Figures S60–S63). The absorption is blue-shifted by about 20 nm for measurements in DCE with respect to measurements in dichloromethane (DCM). Fluorescence lifetimes in dichloromethane were very similar at 12.3 ± 0.2 ns (average ± standard deviation). The emission spectra are shown in Figures S60 and S64. These results indicate that there is not a strong interaction between the solvents molecules and helicene, i.e., toluene acts through activating the SAMs in a way that affects the way the helicene is deposited.

Since we observed different behaviors for our two helicene enantiomers, we investigated our SAMs in terms of CD spectra (Figures S65 and S66). Both the mica and bare gold deposited thereupon showed an intrinsic signal (Figure S65). After annealing and treatment with the SAM-forming molecules, the signal observed was dominated by the substrate properties. Consequently, there is no clear evidence for a chiral TRT-SAM on the macroscopic scale.

### Contact Angle

To illustrate the homogeneity of the surface and discrepancies between individual recipes, contact angle measurements were conducted (Table 3).

**Table 3:**
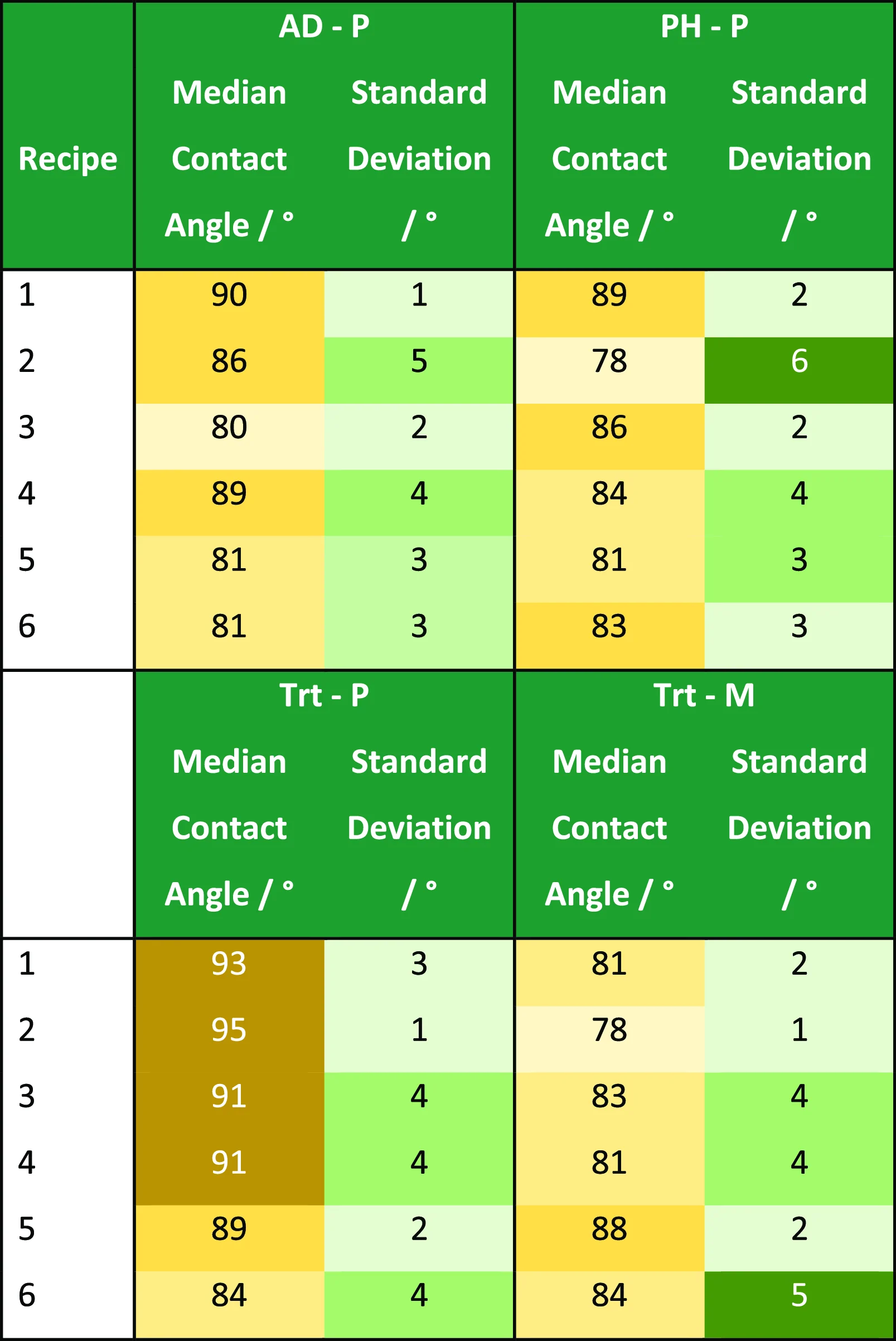
Contact Angle Measurements of Recipes R1–6 for AD, PH, and TRT (P and M Enantiomers) SAMs Covered by the Helicene

As a general trend, the lower the concentration of the helicene, the lower the contact angle, but there are exceptions, such as AD2-3 and PH2. TRT-M completely reversed the trend.

TRT-P shows the highest contact angle, likely related to the high surface area induced by dewetting parts of the surface (on the basis of the first batch of gold samples). In the highly dilute surfaces, we observe both TRT surfaces to converge on the same values, i.e., the macroscopic behavior is determined by structures of similar properties. Hence, problems with deposition start to emerge only at higher concentrations, where the forces driving deposition are much stronger than those driving the structural arrangement.

#### XPS Results

The XPS spectra (Figure 5) were measured to determine the thickness and coverage of the SAMs. Combined with the calculated dipole moments, we would be able to interpret changes in the work function (given below). The thicknesses and molecular coverage are given below (Table 5). The compact PH-SAM showed about twice the coverage as that of the bulky AD and TRT-SAM. In combination with the calculated dipoles (Table 4), we can gain a first-order approximation for the resulting overall dipole created on the surface. By multiplying both values, we arrive at 15, 19, and 16 for AD, PH, and TRT, respectively, i.e., similar values are reached. The slightly larger value for PH-SAM probably overestimates the actual situation since the larger-than-expected thickness for the PH-SAM (Table 5) indicates an incomplete bilayer, which will be removed during the spin-coating application of the helicene. Consequently, the application and orientation of the helicene will play a relevant role in the subsequent KFM measurements.

**Figure 5:**
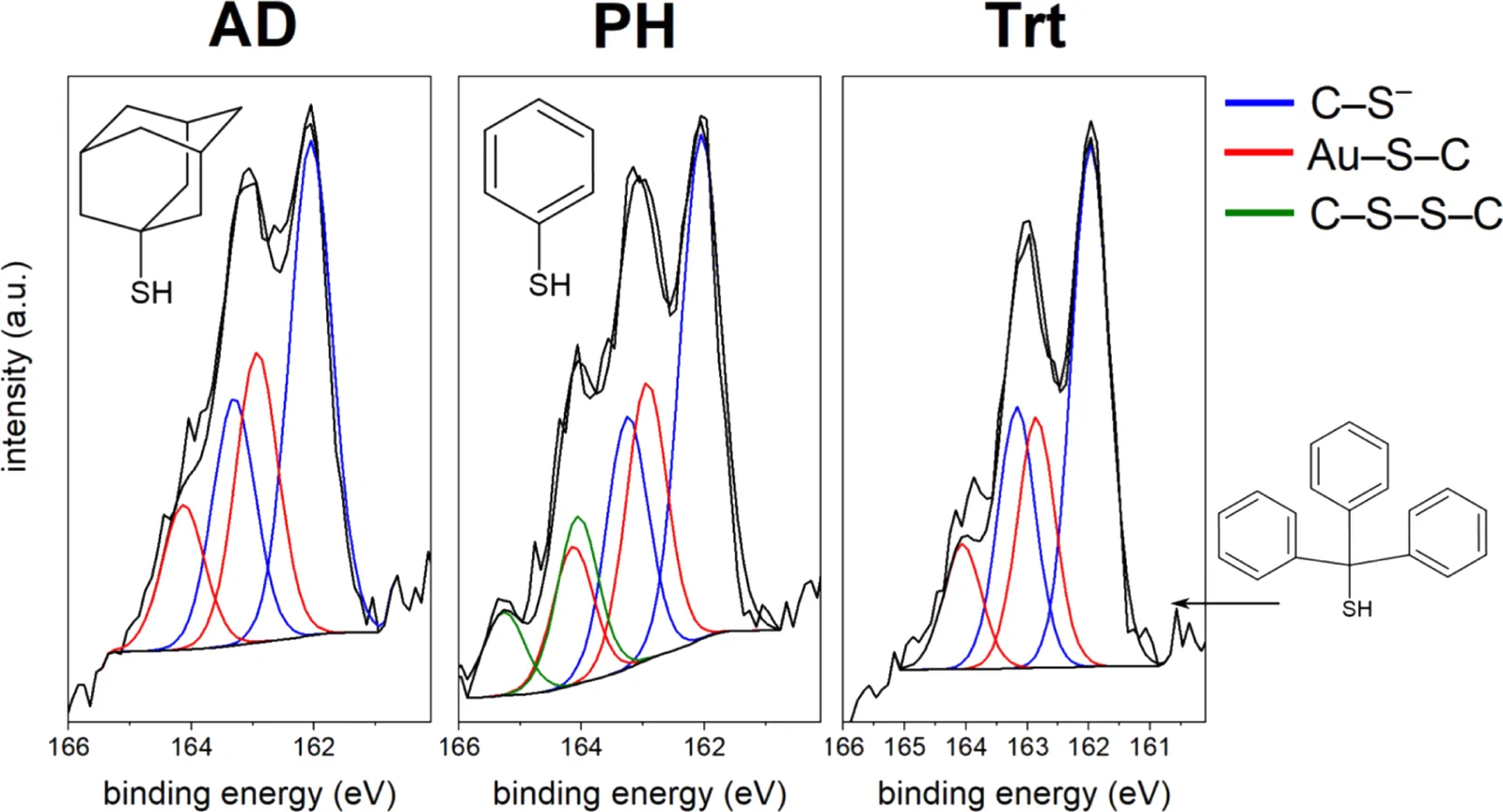
XPS measurements for the SAMs investigated in this work. The shown S 2p spectra were used to determine the values given in Table 5. The structures of the SAM-forming molecules are given for illustration.

**Table 4:** Dipole Moments of the Helicene and the SAMs Employed^a^

Axis/Molecule	*x*	*y*	*z*	Total
Helicene	**–2.46**	0.10	–0.05	2.46
AD-SAM	–0.01	0.17	**4.09**	4.09
PH-SAM (face-on)	1.32	–0.86	**1.87**	2.45
PH-SAM (edge-on)	–0.08	–0.46	**2.71**	2.75
TRT-SAM	0.22	0.33	**5.17**	5.18

a For the helicene, the *x*-axis corresponds to the long axis, as illustrated in Figure 1.

**Table 5:** SAM Thickness and Coverage Obtained from XPS Data^53^

	SAM Thickness/nm	Coverage/10^14^ molecules cm^–2^
AD	1.0	3.74
PH	1.4	7.00
TRT	1.3	3.07

The TRT-SAM has the largest head group and hence is likely to result in the least uniform of the surfaces. Initial XPS results suggest a rather low carbon/sulfur ratio (11:1 and 15:1 after annealing rather than the expected 19:1). Furthermore, annealing led to a decrease of the sulfur signal by 30%, while the carbon signal decreased only by 8%, i.e., small organic thiols were released from the surface and the bare surface was immediately covered by adventitious carbon. We assume this to be caused by cross-contamination when the SAMs were prepared and annealed in temporal vicinity (Figure S67).

We therefore repeated the annealing experiment for RT and annealing at 60, 70, and 80 °C (for 10 min) and were equally surprised. The overall sulfur-to-carbon ratio ranged between 8.4 and 9.2. To discriminate against adventitious sources, we only include C–C bonds in our analysis and ignore C–O and C=O, which are not present in trityl and neither were found in the tritylthiol when investigated by NMR (Figures S68–S70) and only Au–S–C signals for sulfur. The carbon-to-sulfur ratio for the series hence results in 19.8, 20.7, 20.5, and 21.7, respectively (see above). In other words, the TRT-SAM is certainly present and its presence on the surface increases with annealing. However, small hydrocarbon impurities (thiols) bind preferentially to the surface—these are likely contaminations in the commercial compound below the detection limit of our methods. As the TRT headgroup is rather large, a smaller thiol can easily place itself below the large propeller shape of the TRT-SAM and potentially dislodge the larger thiol—i.e., even though not detectable in solution, gold ″filters″ the smaller thiols by binding them preferentially over the intended TRT-SAM. This heterogeneity is reflected in AFM/KFM measurements.

#### Computational Results

To explain the relation between structural properties and the presence and absence of toluene in the deposition solution, we conducted a set of calculations. We simulated the interaction between our helicene and a single solvent molecule in its most likely configurations. A selection of resulting geometries can be found in the Supporting Information (Figure S71).

It was of little surprise that toluene showed in all cases higher interaction energies (Figure S71a–d) as opposed to DCE (Figure S71e–h). Interestingly, the strongest interaction for toluene was found in a spine-on position, while DCE preferred the interaction with the pyrene ring system. The latter position was not clearly favorable for one or the other co-solvent, energetically. This explains why in some cases, both face-on and edge-on oriented material would be observed.

As part of our investigation, we compared the dipole moments of the helicene and single SAM-forming molecules and calculated their ESP surfaces (Table 4 and Figures 1 and S72 and S73). We attempted optimization of trimers, yet these failed to optimize completely and are presented for illustration only.

The dipole moments are of importance to use as we intended to investigate the surface potentials via KFM in order to determine changes to the work function of the gold electrodes we created. The work function was shown to be influenced by SAMs.^14,18^ These changes can be correlated with the SAMs dipole moment, where a dipole pointing away from the surface lowers the work function, while it pointing toward the surface heightens it and hence makes it harder to remove electrons from the surface (“pillow effect”).^28,60^ These effects were reported in SAMs, but also rather large, several nanometer-long SAMs were shown to be relevant for the pillow effect.^61^

The dipoles of all three investigated SAMs were of the same orientation. Except for the PH-SAM, the dipole moments of the SAMs were much larger than the dipole of the helicene.

Since the TRT-SAM was the most peculiar of the SAMs in this study, we investigated its behavior in the presence of the binary solvent (Figure S74a). We sampled the conformational space via CREST with 10 DCE and 2 toluene molecules (-temp 500 -tnmd 600 options). The minimal energy conformers of both conformers were taken. Both were reoptimized with or without the solvent shell at the ωB97X-V def2-SVP level.

Then, we attempted to map the energetic barrier in-between the two enantiomers, which we term “Up” and “Down”, arbitrarily (Figure 1). *In vacuo* (Figure S74b), their energetic difference was found to be <0.1 kcal mol^–1^. Recalculation at ωB97X–V def2-TZVP (Figure S74c) increased the difference in a non-significant manner as it remained below 0.1 kcal mol^–1^. We therefore performed NEB on PBE SVP in ORCA to obtain the isomerisation barrier separating these two enantiomers. *In vacuo* and on gold, both were found to be around 2.5 kcal mol^–1^. This was of little surprise but did not help explain the differences we earlier encountered experimentally. Hence, we repeated our calculation in the presence of solvent molecules (Figure S74d). This increased the barrier significantly (by 100 kcal mol^–1^). The value is of course magnified by the reorganization of a large number of solvent molecules and that only a single reorganization pathway was considered (the minimum); however, it illustrates that the solvent can play a significant role in these processes. This would not explain a global transformation over the complete surface but may explain the local changes and differences between how the two helicene enantiomers are deposited onto the TRT-SAM.

#### KPFM Results

The structural effects observed are of little consequence, lest put into perspective of their effect on the electronic behavior. Therefore, we were interested to correlate mechanical behavior to the surface potential or rather the work function since we calibrated our tip on a clean gold surface. From the computational and XPS results, it became apparent that the orientation of the helicene likely results in a measurable change in work function.^60^

We limited our experiments to R1 and R2, which gave the most stable, fully covered surfaces devoid of tip changes. The results are given in Figure 6; a statistical representation of the average measured value is given in Table 6.

**Figure 6:**
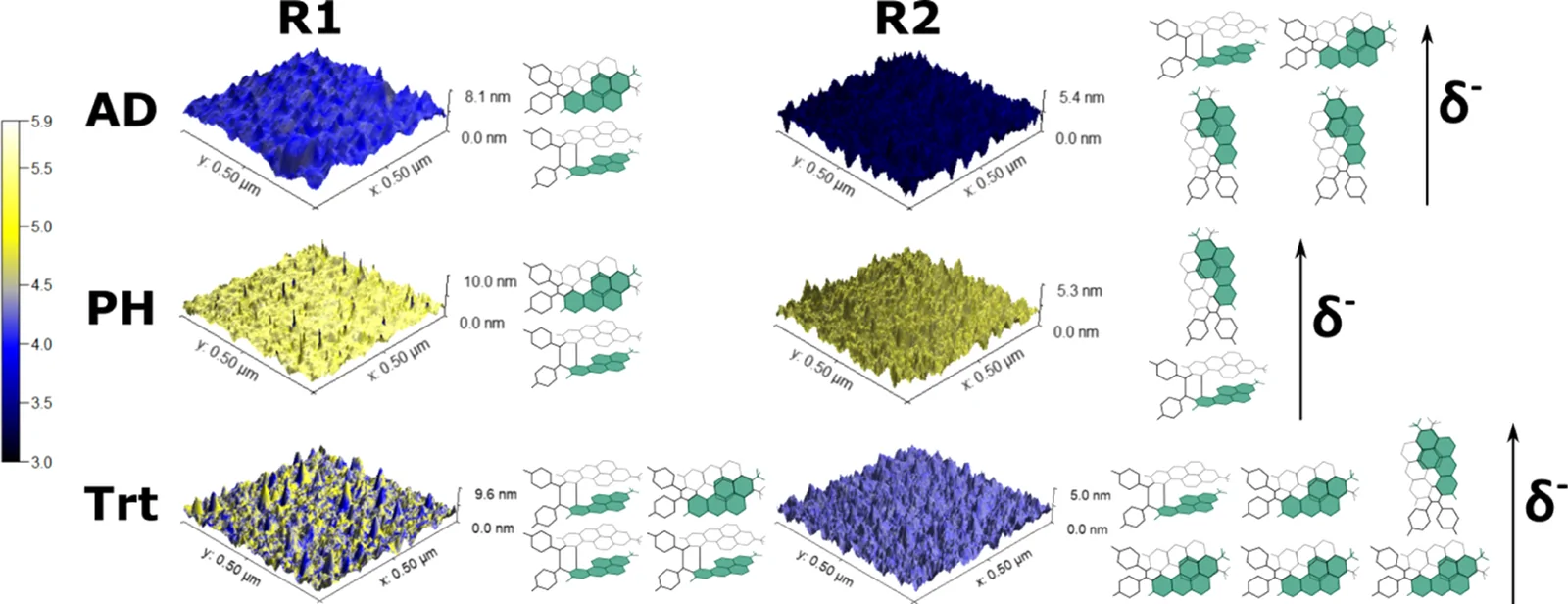
Work function determined via KFM measurements and consequent interpretation of the molecular orientation based off of computational results. On the right of each representative scan, we show the most likely orientation of the helicene in the base layer (lower) and of the respective structures (upper). In the case of multiple possibilities, we indicate the same by multiple structures. R2 likely contains the spine-on oriented helicene, which facilitates the removal of electrons and hence lowers the work function.

**Table 6:** Work Function Determined via KFM Measurements, Including Standard Deviation and Sample Size^a^

	Average/eV	Standard deviation/eV	Samples
AD1	4.1	0.28	3
AD2	3.4	0.10	3
PH1	5.4	0.03	4
PH2	5.0	0.03	3
TRT-P1	5.4	0.45	5
TRT-P2	4.8	0.15	5

a The large variability for the AD samples can be explained by variations in spacing;^65^ for TRT, it is most likely related to the amount of disordered structures (plastic deformation) and the local surface potential variability which results.

The topography of the gold–SAM–helicene layers was overlaid with the work function (i.e., the corrected surface potential). Consequently, the color change correlates with alterations of the work function and thus should be constant if we have a uniform, homogeneous material.

One outright trend is apparent: the highest work function is observed for the most aromatic SAM, while the farther the conjugated material is separated from the gold layer, the lower its value becomes. A second trend is that the addition of toluene leads to a smaller work function on all three surfaces (0.4–1.2 eV) and for the bulky headgroups to less deviation (1SD in AD and TRT SD drops to one-third in the presence of toluene). The latter may be related to a reorganization of the SAM layer in the presence of the aromatic solvent.

In the case of TRT-P, the addition of toluene results in a more homogeneous work function distribution across the surface, while the work function in TRT1-P varies substantially across the surface. The first effect is not merely related to electrical insulation by non-conductive groups as the addition of aromatic groups leads to a higher work function. When we correlate the observed behavior with structural–mechanical properties obtained from QNM measurements, the situation becomes slightly clearer.

AD1 (R1; Figure S6e,f) showed more dissipation linked to deformation as opposed to AD2 (R2; Figure S7e,f); hence, the presence of plastic deformation means a lower work function. For (Figure S13e,f) and PH2 (Figure S14e,f), the deformation images are more comparable, but again, the lower work function correlates with more plastic deformation and fewer hard features. For TRT-P, the plastic deformation signal was found in both TRT1-P (Figure S23e,f) and TRT2-P (Figure S24e,f) and cover a comparable area, and both surfaces feature comparable face-on and edge-on oriented helicenes, based on PeakForce QNM results.

The work functions thus relate to mechanical properties as follow is: ϕ_plasticdef_. < ϕ_non‑def._ < ϕ_elasticdef._, a relationship that evokes a similarity to disorder theory and electric conduction in conductive polymers.^25^ The plastically deforming material is poorly cohesive and thus likely structurally disordered, while the non-deforming is electronically disordered, with a poor overlap of wavefunctions of the helicene and the underlying SAM.^25^ The elastic, face-on oriented materials then likely exhibit low electronic disorder and hence the highest overlap of wavefunctions. This observation, so we hypothesize, suggests a possible link between electronic disorder (charge-transport) and the work function.

This hypothesis is in line with the previous work on PEDOT,^13^ where conductive polymer chains aligned perpendicular to the surface exhibited the highest surface potential (and hence ultimately the higher work function), while other crystalline orientations underperformed. This is in contrast to work on undoped polyphenylene oligomers,^62^ in which the aromatic, rather than the highly conductive quinoid state, would be prevalent.^63,64^ Clearly, more data is relevant to establish such a link reliably, so we remain with a conventional interpretation of the data.

If we were only concerned with the work function modulation of a gold electrode by a SAM, it could be well-described by the push-back or “pillow” effect reported by Vázques and co-workers.^28,60^ There, the Pauli exchange repulsion results in a compression of the electron cloud. This results in an interfacial dipole and a change of the work function in dependence of the SAMs headgroup, the linker,^19^ and its orientation: A dipole that points away from the surface lowers the work function as it facilitates removal of electrons from the surface, while a dipole pointing in the opposite direction—toward the surface—helps the surface retain electrons, i.e., higher energies are required to access the equivalent orbitals.

On the basis of XPS observations combined with the calculated dipoles (see above) of the SAMs, the work function of our SAMs will be comparable but ultimately not the same.

Now, when the helicene layer is added, the maximum work function should be observed when the maximum dipole points away from the substrate surface (spine-on). Since the dipole of our SAMs is on the same order of magnitude, the helicene orientation will have a measurable effect on the work function.

In our case, we arrive at work functions given in Table 6, where the bigger work function is always found in toluene-free recipes (Figure 6, R1). In the absence of toluene, also, the largest variation in work function is found (Table 6), i.e., toluene helps achieve a more uniform SAM (removal of impurities, adlayers, orientation control, etc.).

The film thicknesses and feature heights were investigated across multiple samples and the measured number was divided by the dimensions of our helicene. The best match was chosen as the prevalent orientation and can be seen in Figure 6, next to the 3D visualization of the KPFM measurement. In all three R2 examples, the spine-on-oriented helicene is the most likely explanation for the lower work function.

With the control of alignment over the helicene orientation, this may present an elegant way to answer an important question in helicene research: whether the conduction therein proceeds preferentially through the π-stack or through the structures conjugated backbone. Such an experiment would require the comparison of two almost identical helicene systems; one that is fully conjugated and the other with its conjugation interrupted (just as in our case). To answer that question remains a task for future research.

## Conclusions

We demonstrate that mechanically anisotropic helicenes (see the graphical abstract for a simplified illustration) enable orientation readout with PeakForce QNM and how this can be supported by KFM measurements to achieve maximum synergies in terms of the characterization of the molecular orientation. In this way, we are able to gain insight into whether the thin films consist of edge-on, face-on, and spine-on material, which result in hard areas, elastic deformation, and shifts in the work function, respectively.

We observe three SAMs with adamantyl (AD), phenyl (PH), and trityl (TRT) headgroups.

For the AD-SAM, the helicene assembles into hard features (∼edge-on) and only at 1000-fold dilution does the face-on orientation dominate—and only in the presence of traces of toluene.

For the PH-SAM, again, hard features dominate, and only the extreme dilutions result in a situation where both populations get close to each other.

At last, the TRT-SAM proved to be complex. No global chirality can be observed, and yet, we observe distinct assembly types for the two enantiomers and the racemic mixture alike. The presence of toluene and moderate dilution achieve the largest areas covered in elastically deforming features, and yet, the same recipes prove the hardest to characterize, with poor adhesion to the SAM (PH and TRT-P) or in a single batch of samples by dewetting behavior (TRT-P).

Optical characterization opposes the notion of a strong toluene–helicene interaction, and thus, this is likely related to the solvent catalyzing the interaction between the SAM and the helicene molecule.

The racemic mixture results in structures that are likely enantiopure (round features) or in elastically deforming spherical features, where a layer-by-layer separation may occur. These were the only surfaces where elongated, rodlike features were observed, suggestive of racemic crystallites.^58^

These findings provide a simple processing strategy to tune the work function in organic electronics and motivate future studies on conductance pathways in fully conjugated helicenes and on direct transport measurements to test the proposed tunneling through π-stacks.

The use of helicenes for studies of such extremely thin films is of particular interest where conventional techniques fail due to their detection limits. Further, we were able to correlate these mechanical effects with electronic properties, specifically the work function. The worse the disorder of the material is, the lower the work function becomes; i.e., plastic deformation (electronic and mechanical disorder) < edge-on orientation (electronic disorder) < face-on orientation (lowest electronic disorder). This indicates a poor overlap of wavefunctions or mismatch of orbital energies in the edge-on orientation vs. the SAM and gold surface. This is linked to a transmission of the work function and likely acts in tandem with the “pillow” effect, which is typically considered to be limited to SAMs, rather than thin films.

If this effect is truly proven to be related to the overlap of wavefunctions and thus to conductance,^13^ this work becomes an important stepping stone for the research of molecular conductance in helicenes: it indicates that helicene conductance and their mode of conductions (tunneling through the π-stack vs. conduction along the covalent backbone) could be studied indirectly through a combination of PeakForce QNM and KFM, in addition to break-junction experiments, which come with their own set of challenges.^12^

## Supplementary Material







## Notes

Script-based quantification of the mechanical features of pure enantiomers (PDF)
QNM guidance and peculiarities of the trityl group; QNM results; spectroscopy; XPS; NMR: computational section; and SEM (PDF)
Script-based quantification of the mechanical features for racemate measurements (PDF)
