# Supplementary material for: Self-Assembled Monolayers and Solvent Composition Control the Orientation and Work Function of Pyrene-Based [7]Helicenes
Source: Langmuir. 2026 Sep 29;42(40):29622–37. doi: 10.1021/acs.langmuir.6c01280 (PMC13674279; doi:10.1021/acs.langmuir.6c01280)
Supplement: Supplementary file 1 [file la6c01280_si_001.pdf]

Supplementary information II:

Self-Assembled Monolayers and Solvent Composition Control Orientation and Work Function of Pyrene-Based [7]Helicenes

Ivan Alic[a+], Katsiaryna Kutsenka[b+], Kristián Kříž[a], Lukáš Kalina[c], David Bína[a,d], Jan Hanus[b], Jaromír Kopeček[e], Anne de la Pena Lopez[a], Radek Litvín[a,d], Milan Mašát*[f], Eva Kaletová[f], Dominik Farka* [a,b]

[a] Department of Chemistry, Faculty of Science, University of South Bohemia in České Budějovice (JU), Branišovská 1760, 370 05 České Budějovice, Czech Republic.
[b] Functional Molecules Group, Institute of Organic Chemistry and Biochemistry (IOCB), Czech Academy of Sciences (CAS); Flemingovo Náměstí 542/2, 160 00 Prague, Czech Republic.
[c] Faculty of Chemistry, Brno University of Technology (BUT), Purkyňova 118, CZ-612 00 Brno, Czech Republic
[d] Institute of Plant Molecular Biology, Biology Centre, Czech Academy of Sciences, Branišovská 31, 370 05 České Budějovice, Czech Republic
[e] Department of Materials Analysis, Institute of Physics (FZU) of Czech Academy of Sciences (CAS); Na Slovance 2, 182 00 Prague 8, Czech Republic.
[f] Molecular Devices Group, Institute of Organic Chemistry and Biochemistry (IOCB), Czech Academy of Sciences (CAS); Flemingovo Náměstí 542/2, 160 00 Prague, Czech Republic.

*corresponding author
[+] These authors contributed equally to the work

Number of pages: 86
Number of figures: 80
Number of schemes:0
Number of tables: 0

Table of Contents:
S3 Script-based quantification of mechanical features of pure enantiomers

Mechanical Features and applied masks.

All the below data were generated via our in-house created Python script.
All the below figures relate to a single QNM-AFM measurement. Each consists of 3 rows of three images. From left to right, the row consists of:
Row 1: Deformation, Dissipation, and deformation with Hard mask overlay (colour: teal)
Row 2: Deformation with Elastic, Plastic, and Adhesive mask, respectively (colour, teal)
Row 3: Histogram of deformation channel, histogram of dissipation channel, and box plot of all four fractions (emergent from the mask area)

The masks were generated as follows:
Hard: areas with **lowest** 25% deformation AND **lowest** 25% dissipation
Elastic: areas with **highest** 25% deformation AND **lowest** 25% dissipation

Plastic: areas with **highest** 25% deformation AND **highest** 25% dissipation
Adhesive: areas with **lowest** 25% deformation AND **highest** 25% dissipation

All images contain a header indicative of the original file-name.
All measured images were treated by a data cutoff (1.5 * interquartile range) for optimal visualization (integral to the script).

AD, Recipe 1, type 1

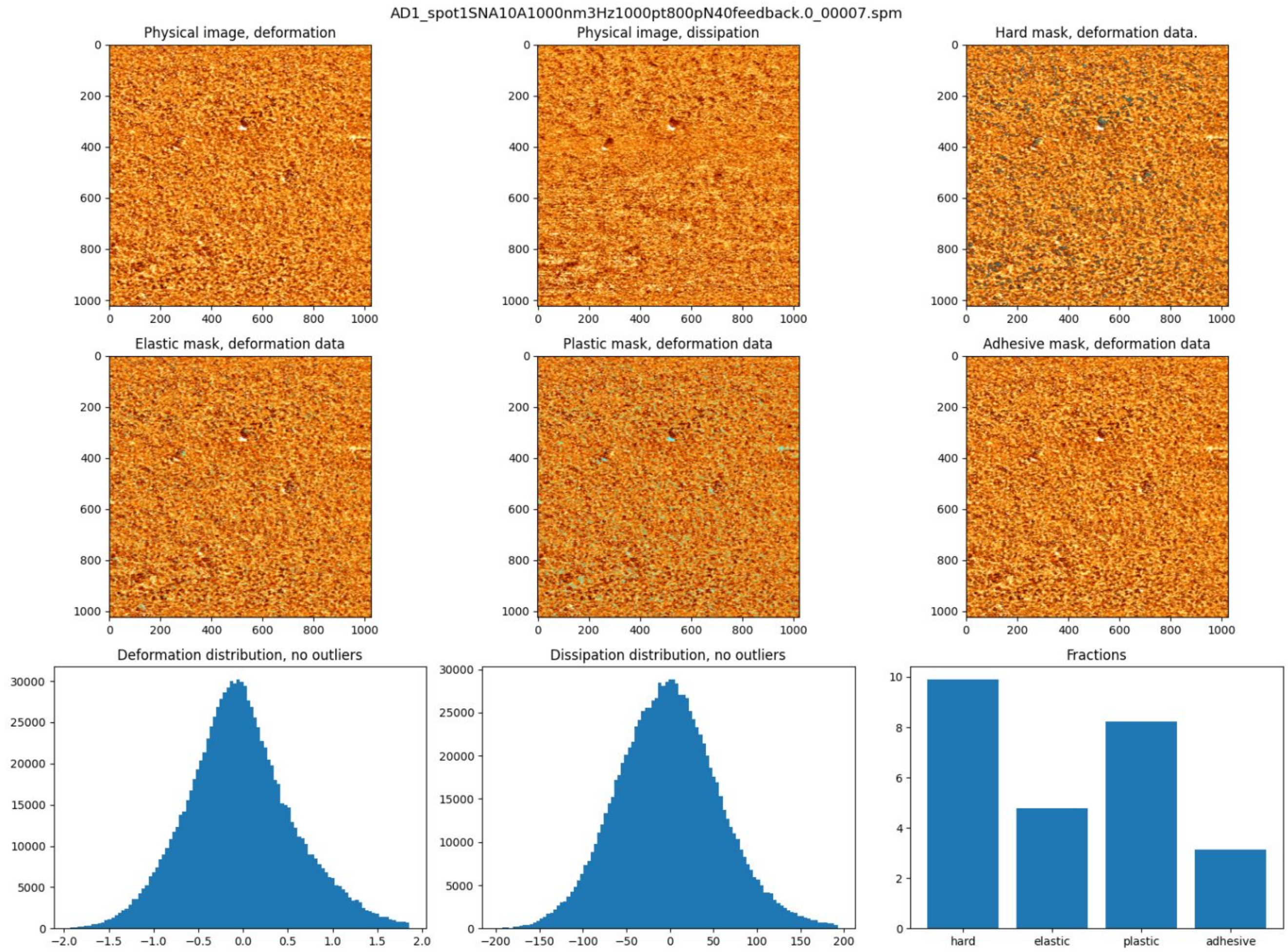

AD, Recipe 2, type 1

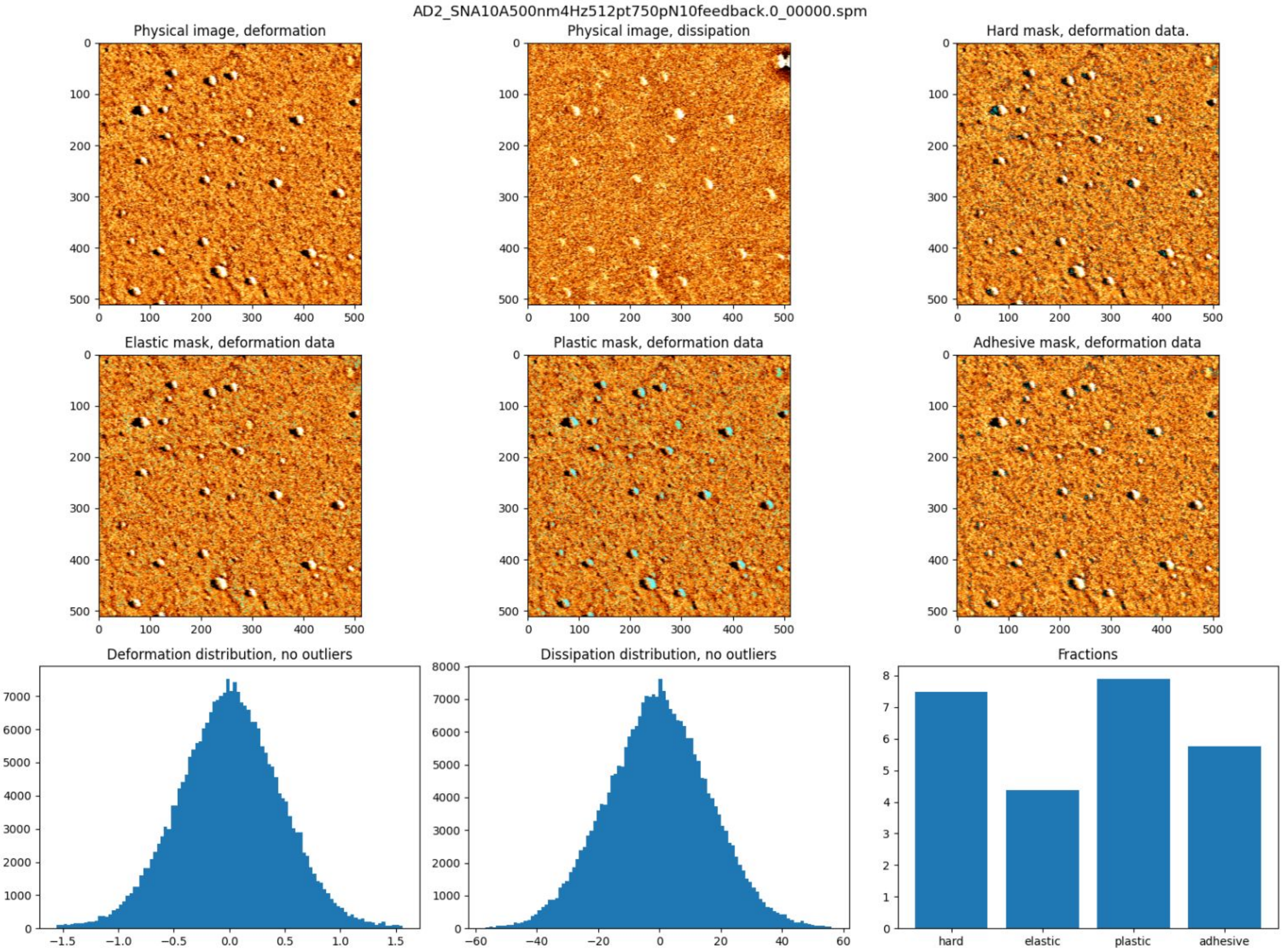

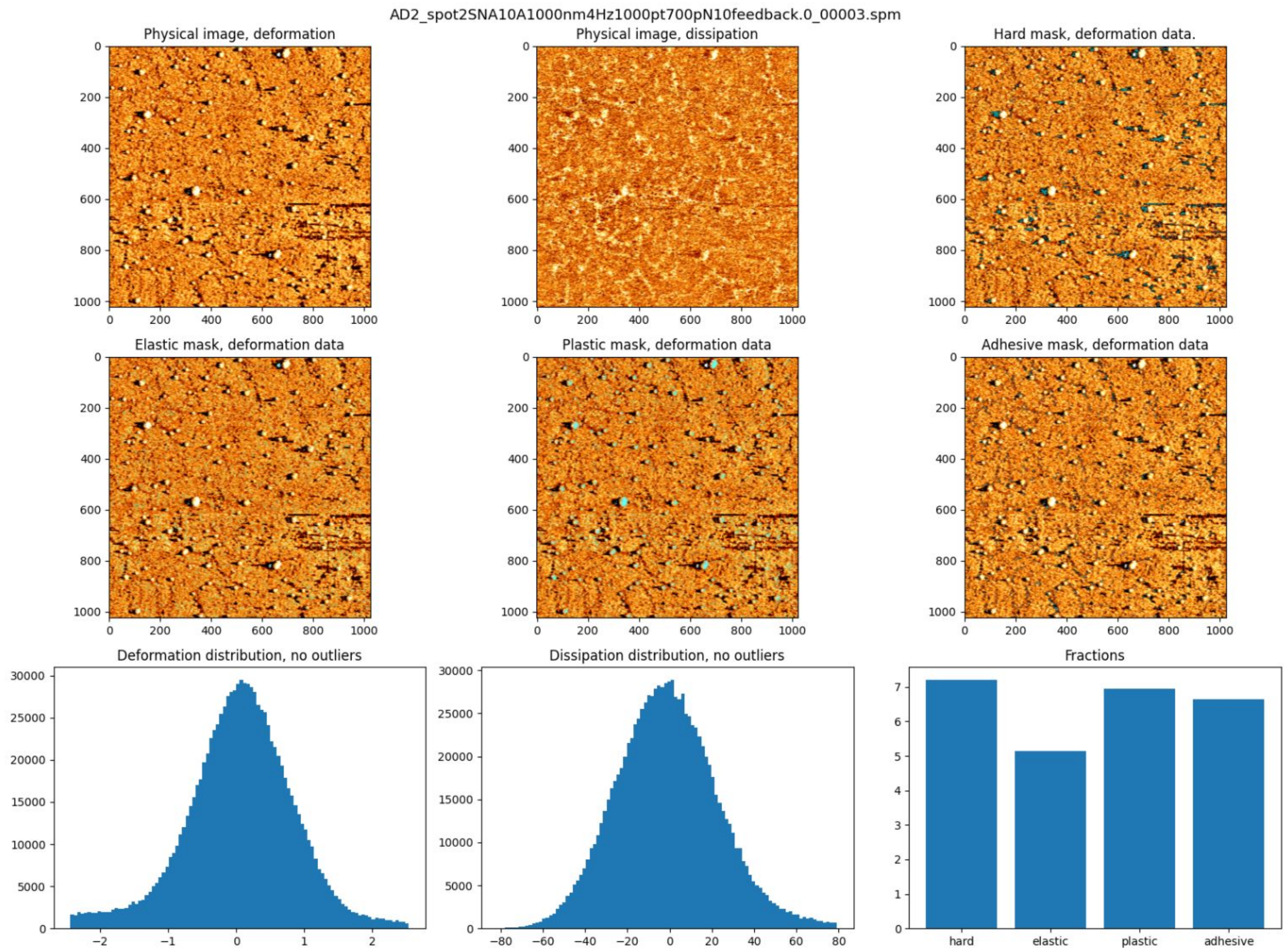

AD2_spot2SNA10A1000nm4Hz1000pt700pN10feedback.0_00003.spm
Physical image, deformation
Physical image, dissipation
Hard mask, deformation data.
Elastic mask, deformation data
Plastic mask, deformation data
Adhesive mask, deformation data
Deformation distribution, no outliers
Dissipation distribution, no outliers
Fractions
hard
elastic
plastic
adhesive

AD, Recipe 2, type 2

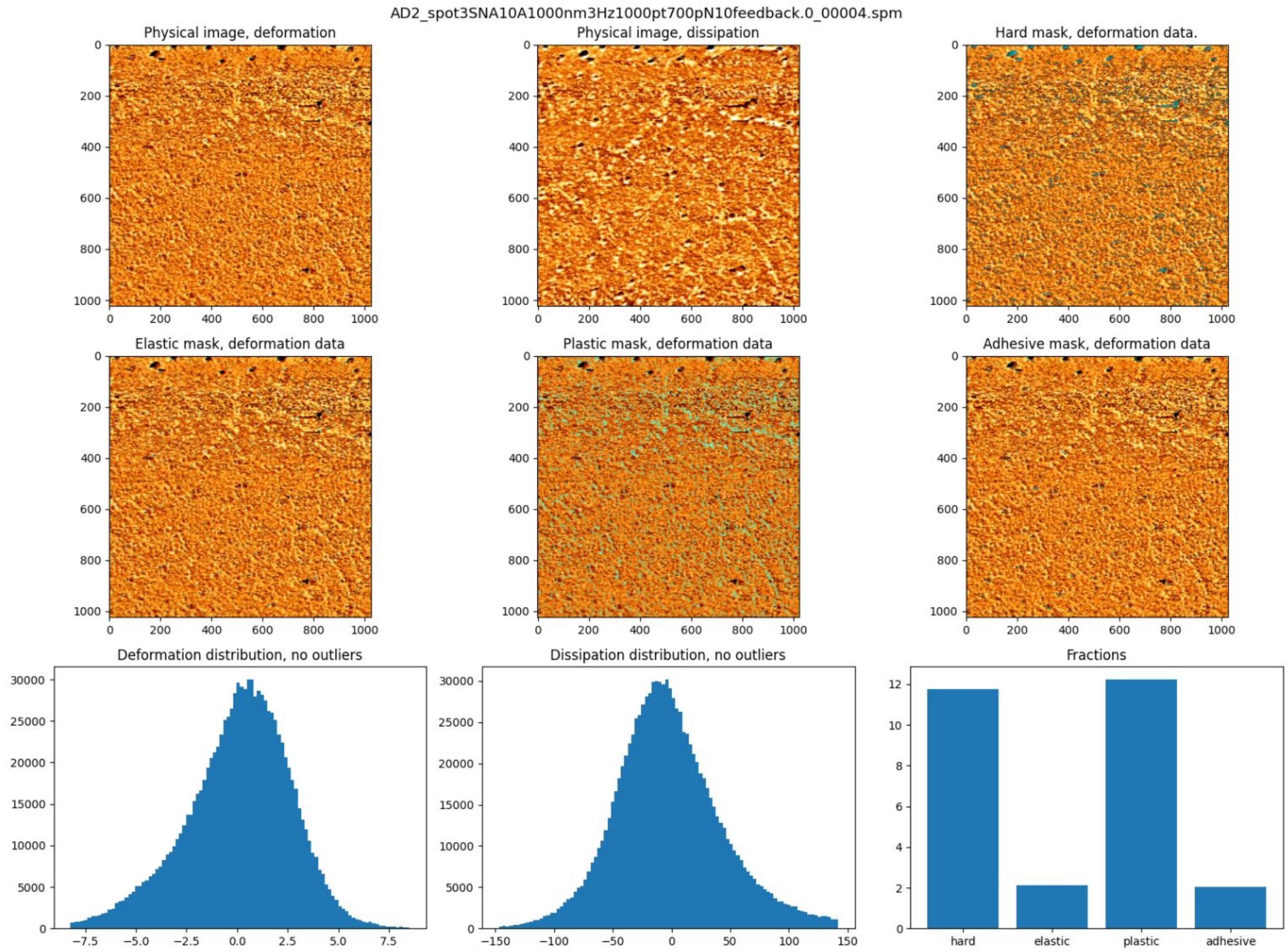

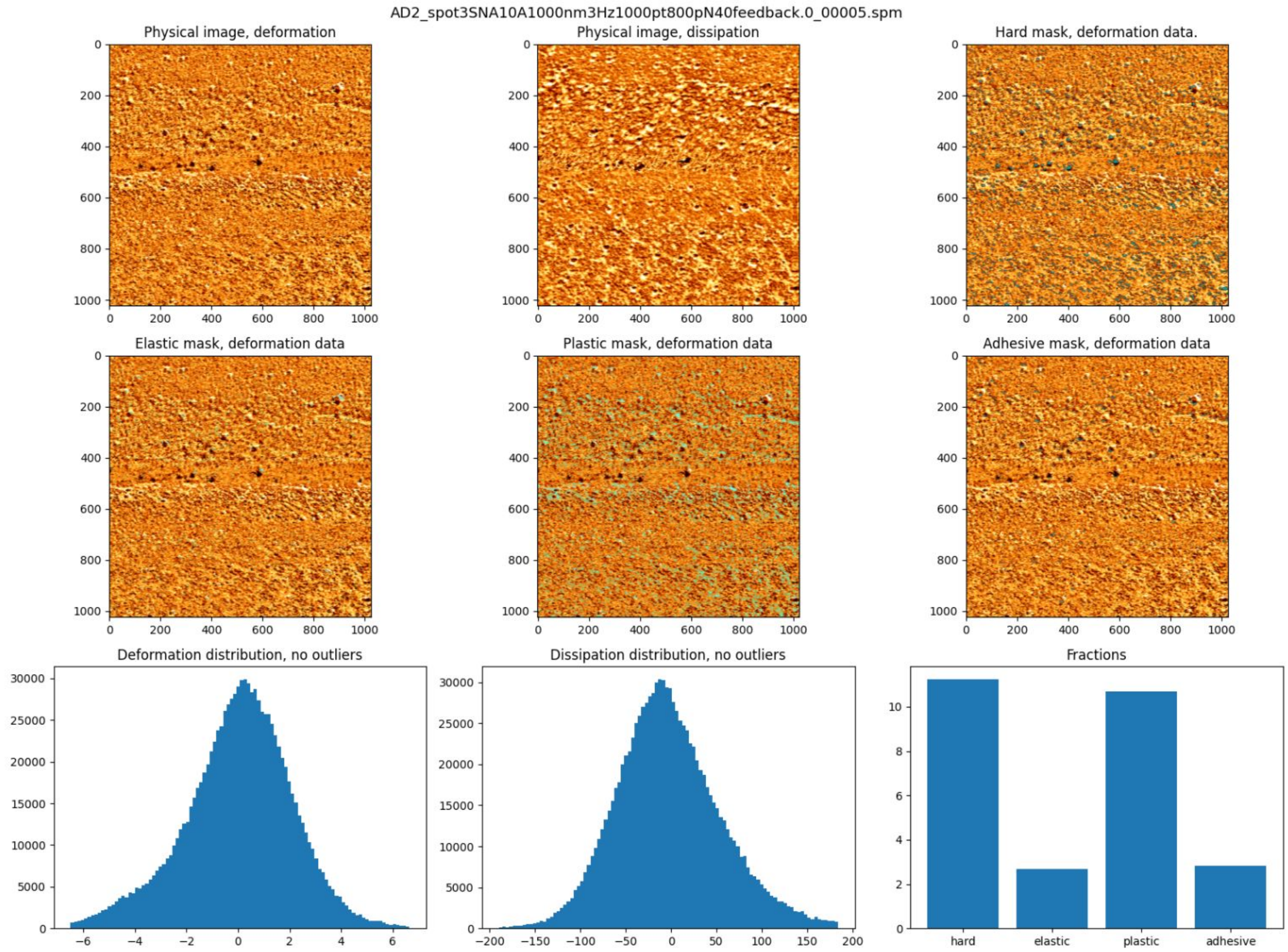

AD2_spot3SNA10A1000nm3Hz1000pt800pN40feedback.0_00005.spm
Physical image, deformation
Physical image, dissipation
Hard mask, deformation data.
Elastic mask, deformation data
Plastic mask, deformation data
Adhesive mask, deformation data
Deformation distribution, no outliers
Dissipation distribution, no outliers
Fractions
hard
elastic
plastic
adhesive

AD, Recipe 3

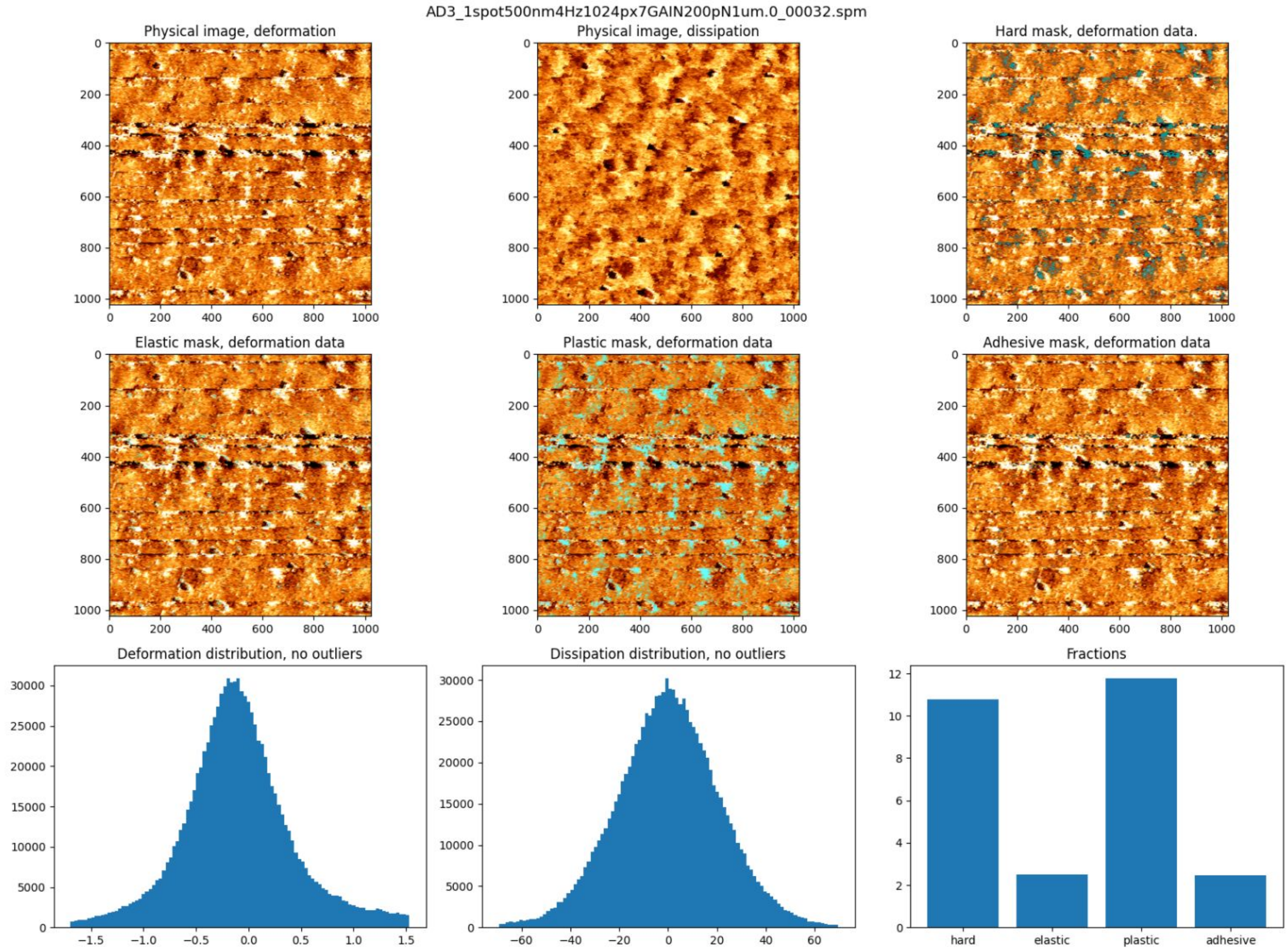
AD3_1spot500nm4Hz1024px7GAIN200pN1um.0_00032.spm
Physical image, deformation
Physical image, dissipation
Hard mask, deformation data.
Elastic mask, deformation data
Plastic mask, deformation data
Adhesive mask, deformation data
Deformation distribution, no outliers
Dissipation distribution, no outliers
Fractions
hard
elastic
plastic
adhesive

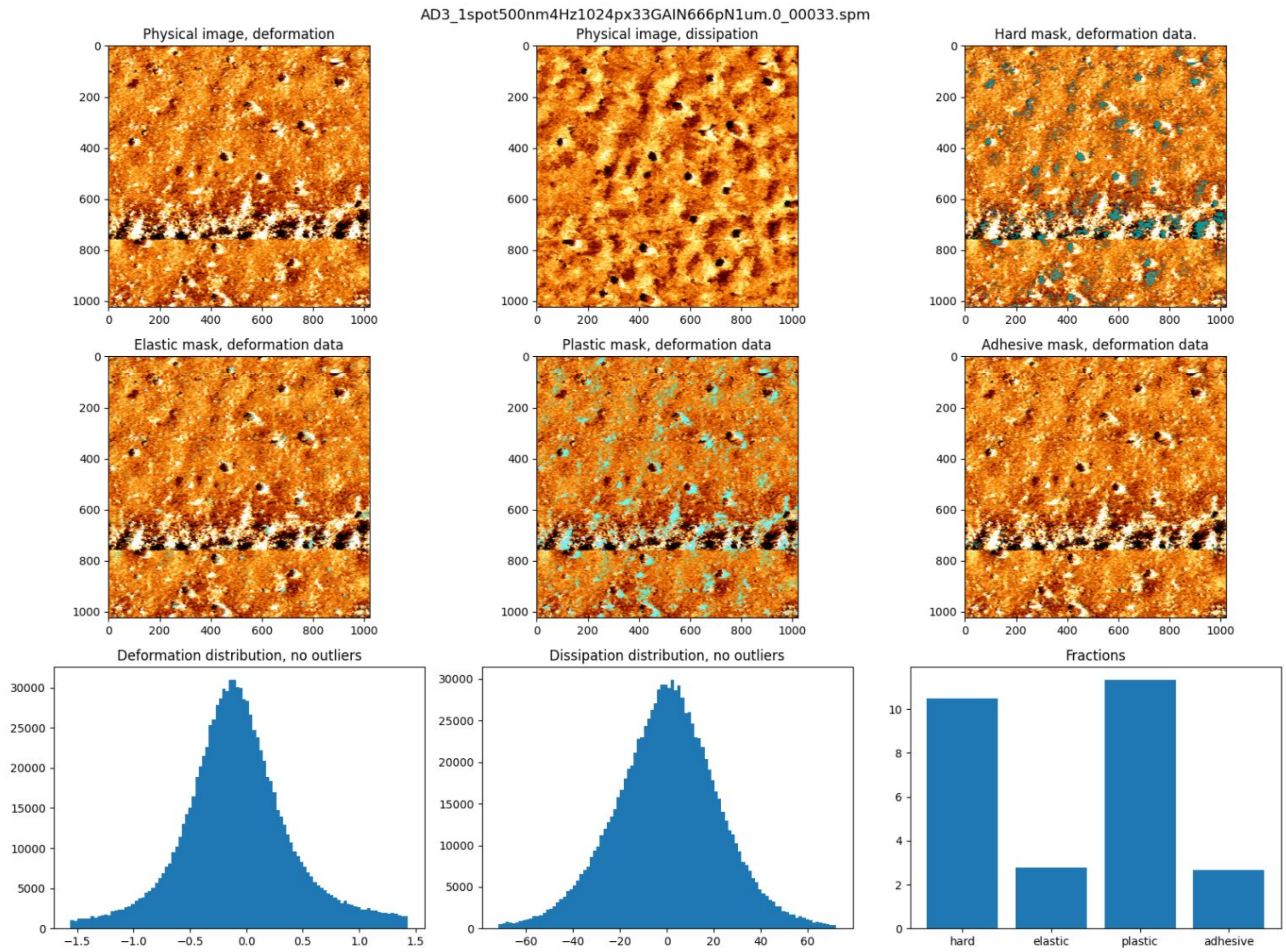
AD3_1spot500nm4Hz1024px33GAIN666pN1um.0_00033.spm
Physical image, deformation
Physical image, dissipation
Hard mask, deformation data.
Elastic mask, deformation data
Plastic mask, deformation data
Adhesive mask, deformation data
Deformation distribution, no outliers
Dissipation distribution, no outliers
Fractions
hard
elastic
plastic
adhesive

AD, Recipe 4, type 1

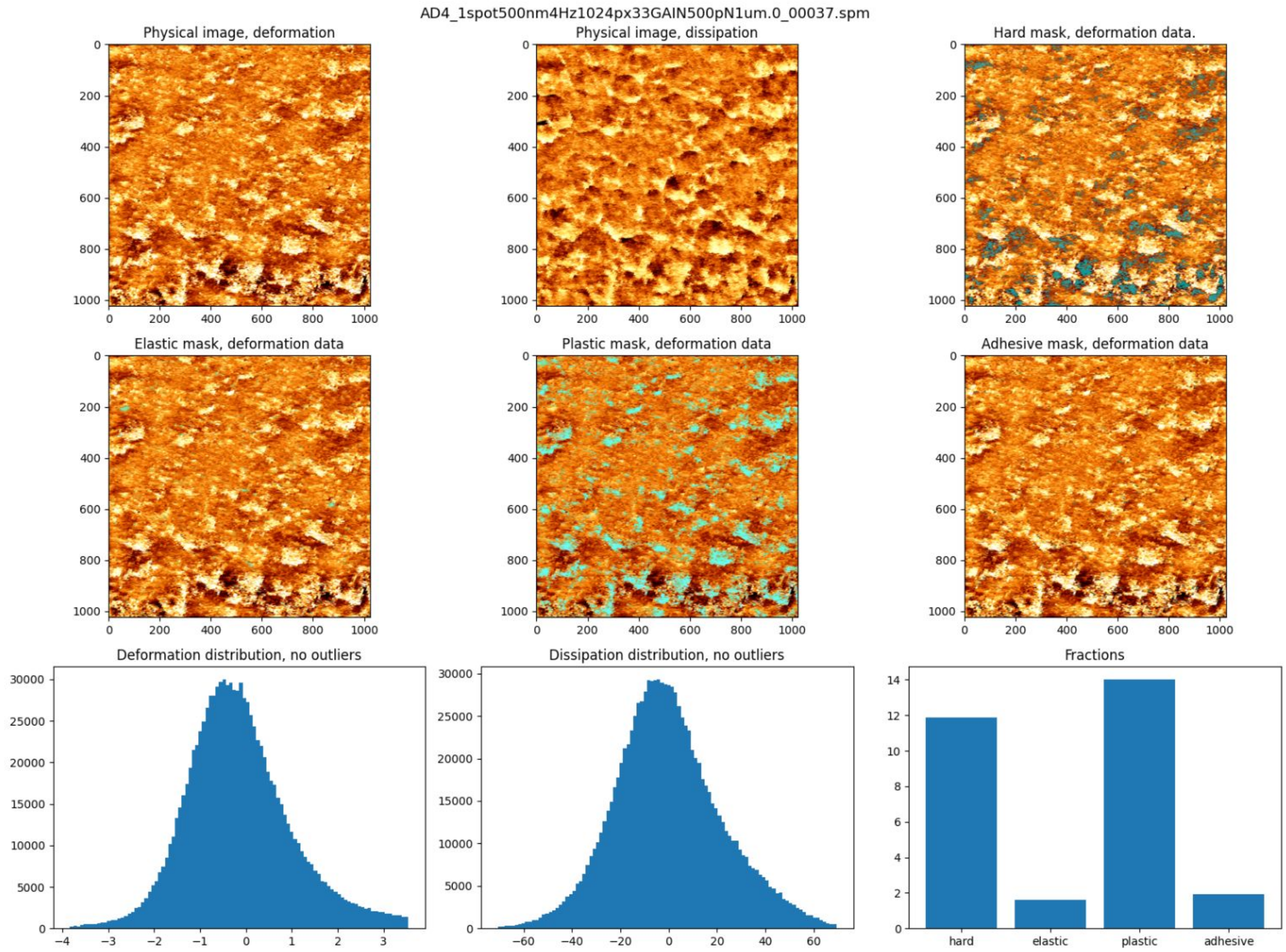

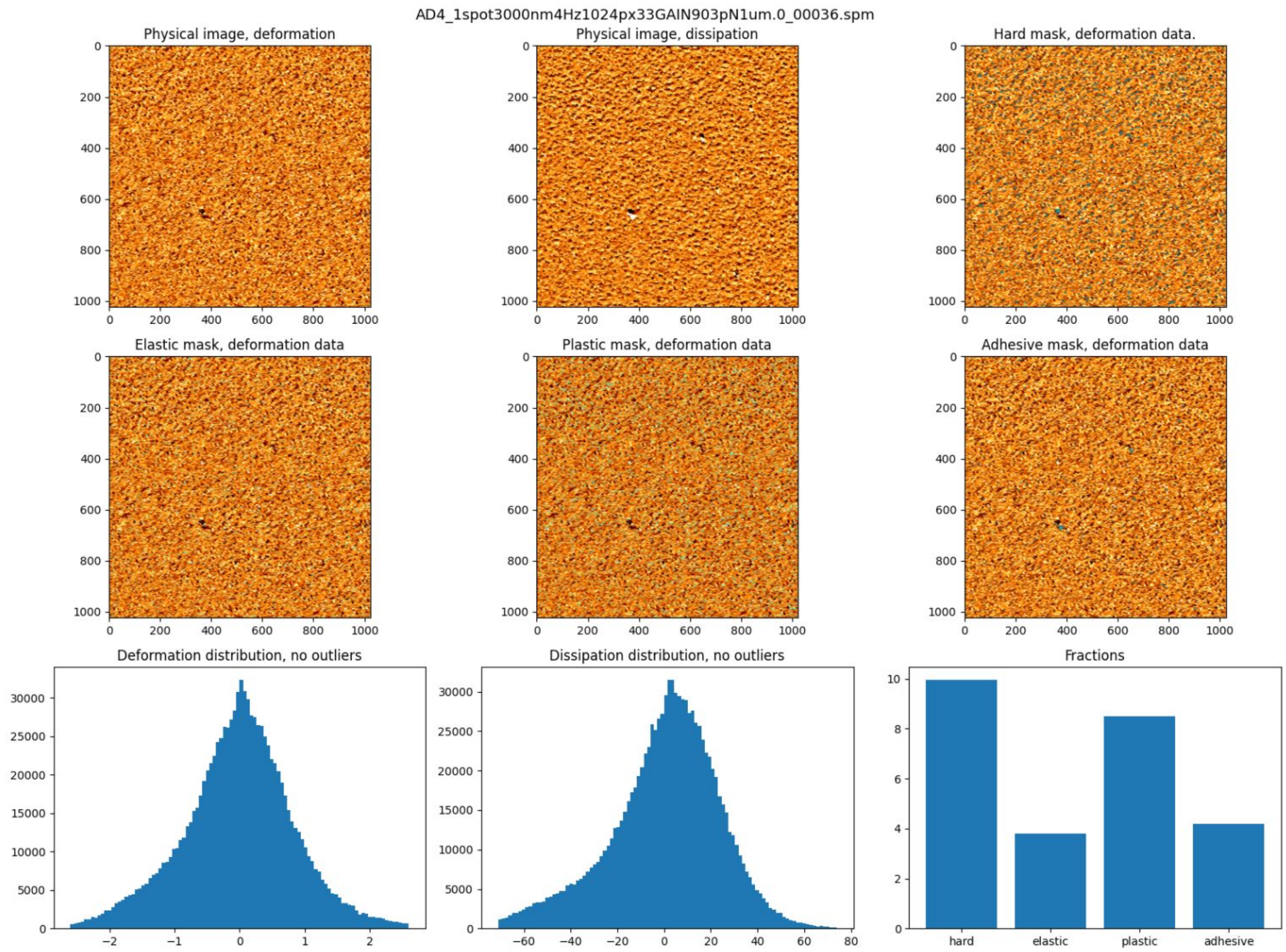
AD4_1spot3000nm4Hz1024px33GAIN903pN1um.0_00036.spm
Physical image, deformation
Physical image, dissipation
Hard mask, deformation data.
Elastic mask, deformation data
Plastic mask, deformation data
Adhesive mask, deformation data
Deformation distribution, no outliers
Dissipation distribution, no outliers
Fractions
hard
elastic
plastic
adhesive

AD, Recipe 4, type 2

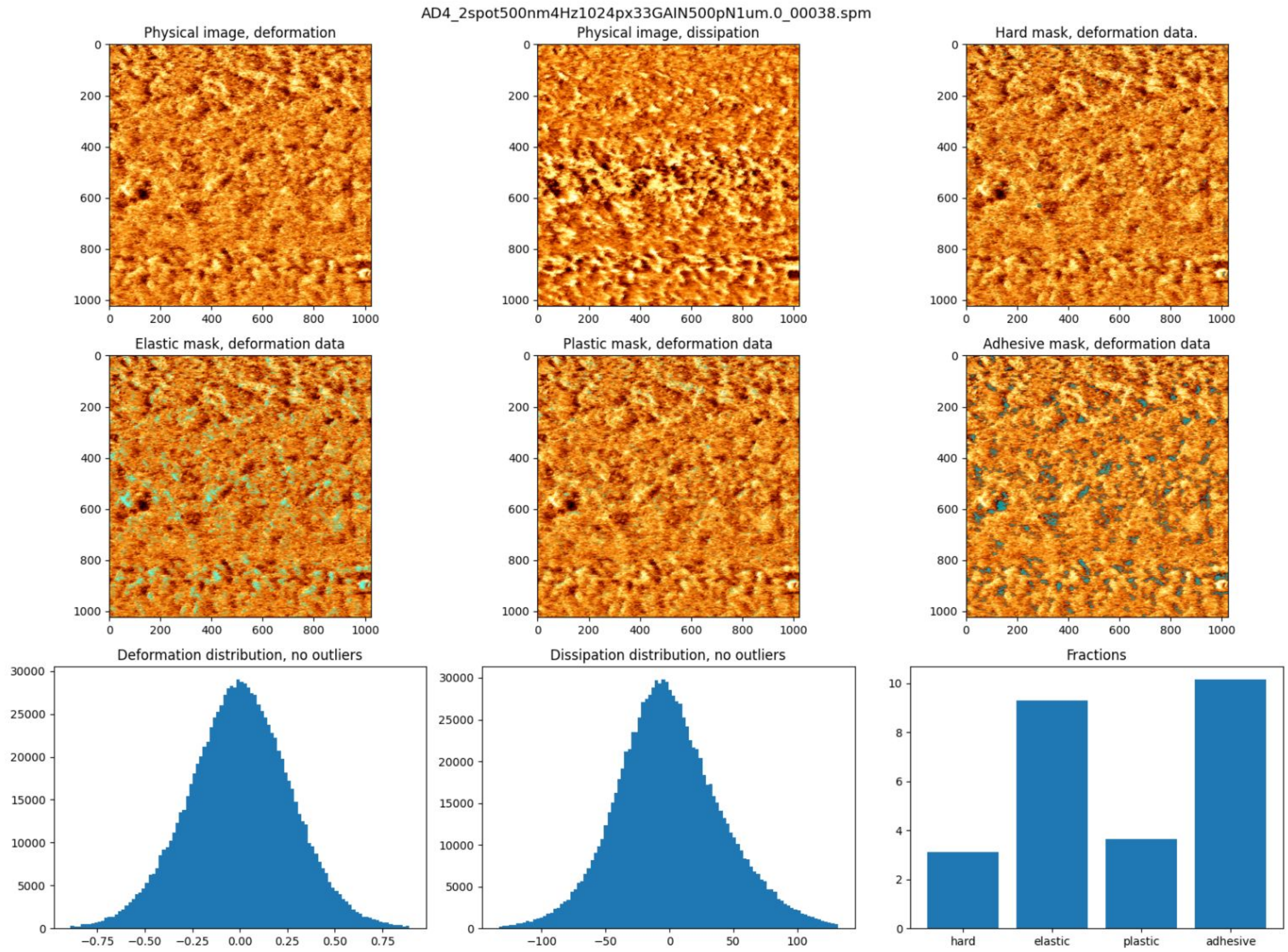

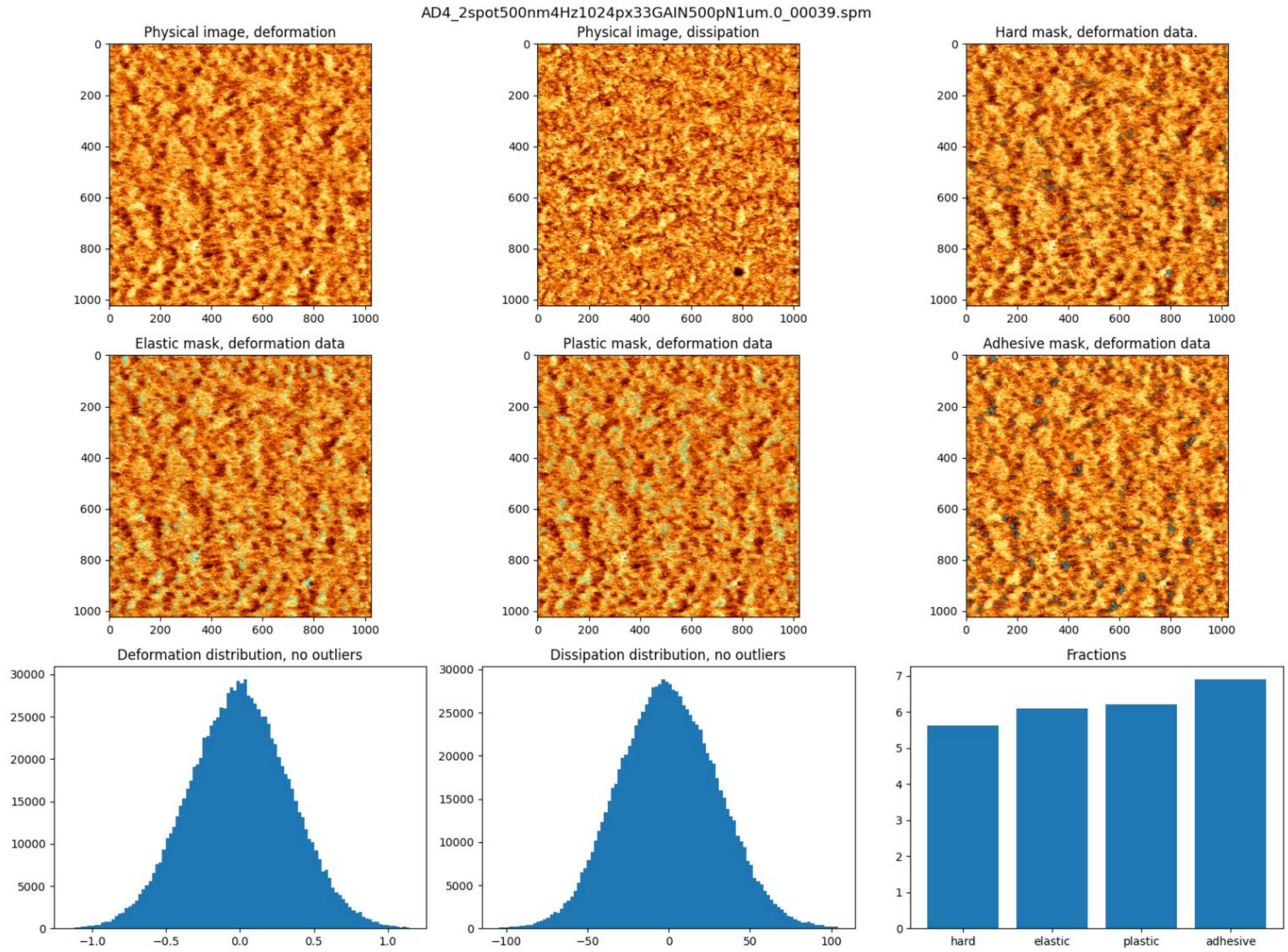
AD4_2spot500nm4Hz1024px33GAIN500pN1um.0_00039.spm
Physical image, deformation
Physical image, dissipation
Hard mask, deformation data.
Elastic mask, deformation data
Plastic mask, deformation data
Adhesive mask, deformation data
Deformation distribution, no outliers
Dissipation distribution, no outliers
Fractions
hard
elastic
plastic
adhesive

AD, Recipe 5

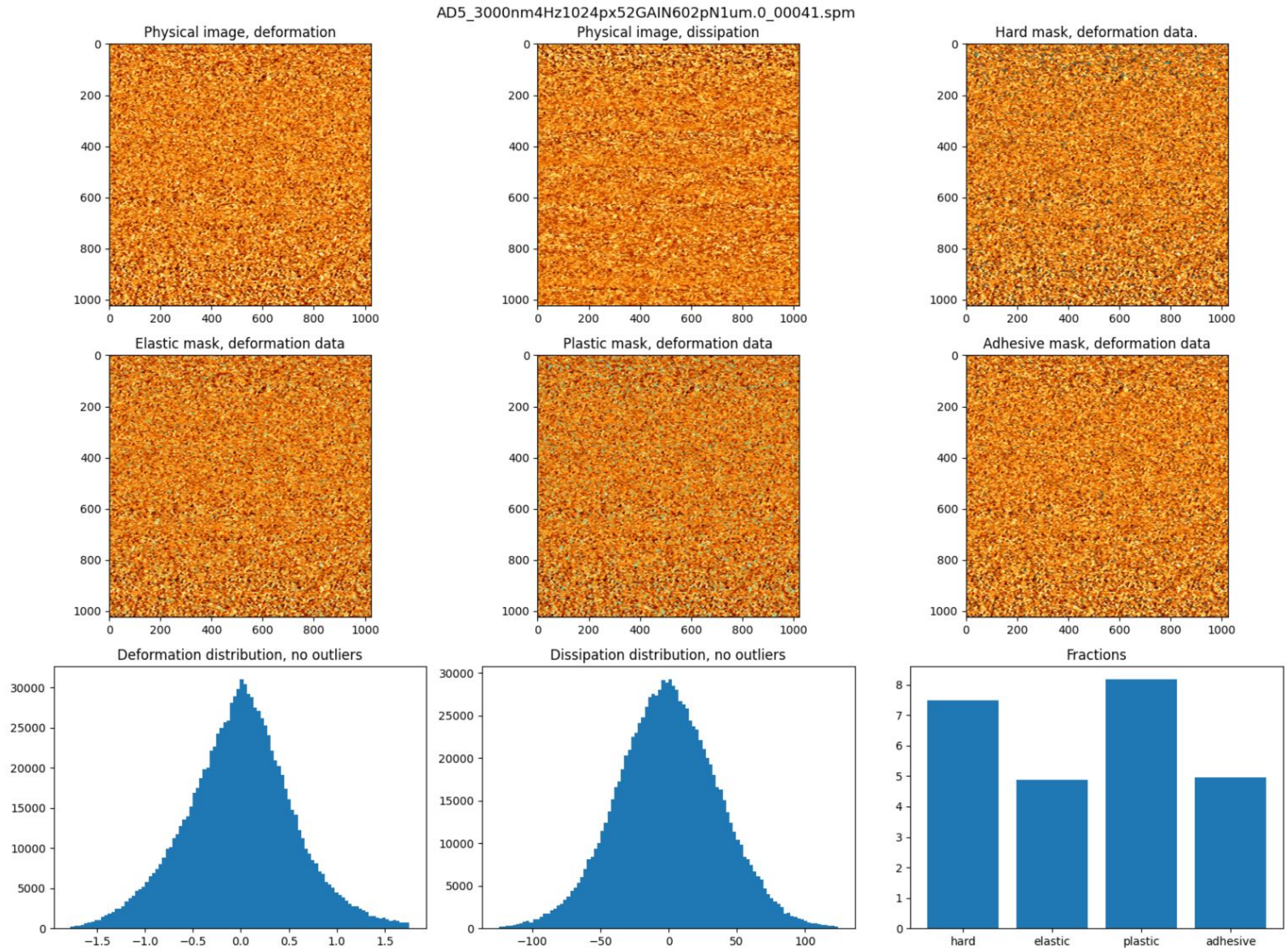
AD5_3000nm4Hz1024px52GAIN602pN1um.0_00041.spm
Physical image, deformation
Physical image, dissipation
Hard mask, deformation data.
Elastic mask, deformation data
Plastic mask, deformation data
Adhesive mask, deformation data
Deformation distribution, no outliers
Dissipation distribution, no outliers
Fractions
hard
elastic
plastic
adhesive

AD, Recipe 6

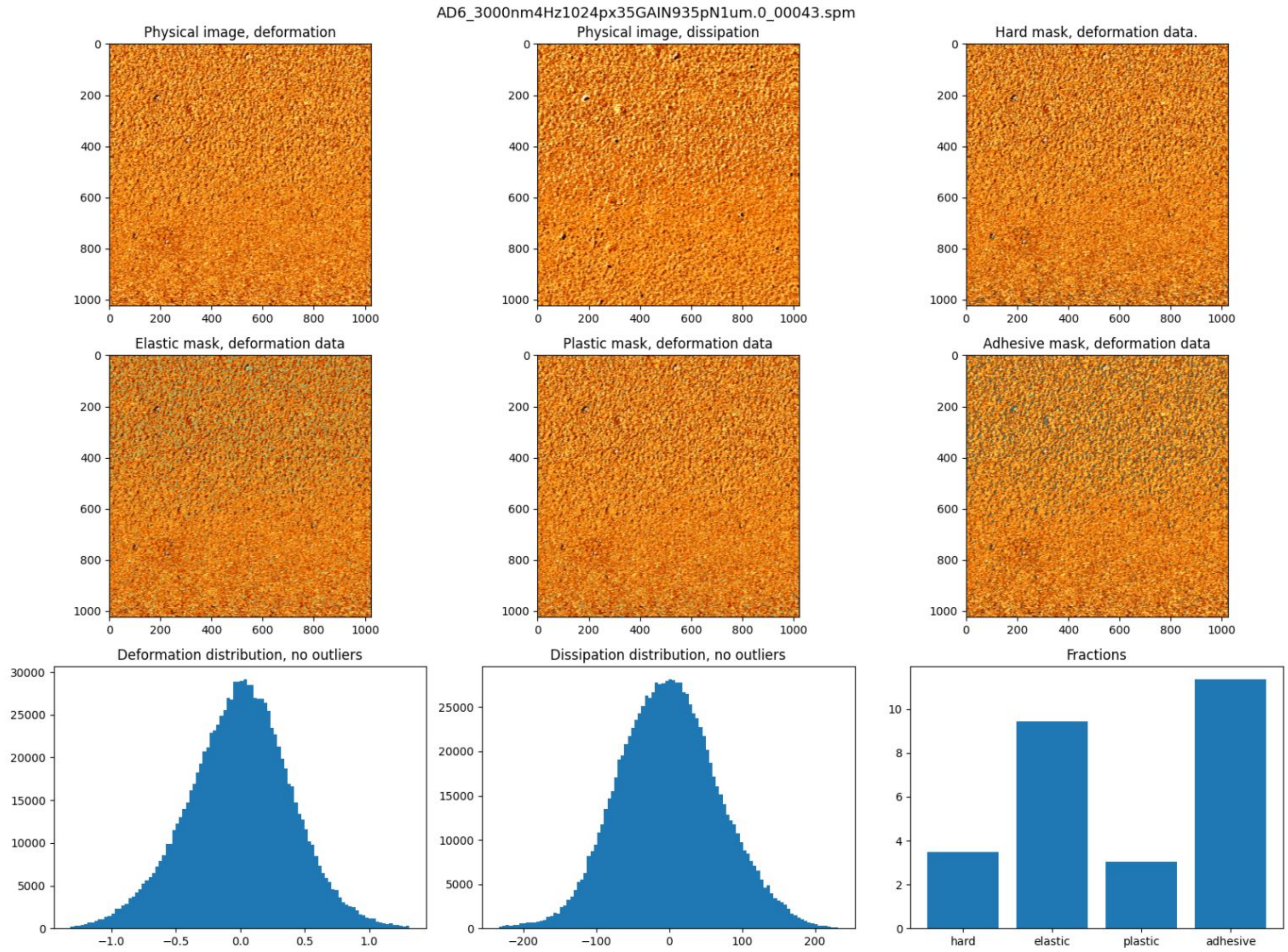

AD6_3000nm4Hz1024px35GAIN935pN1um.0_00043.spm
Physical image, deformation
Physical image, dissipation
Hard mask, deformation data.
Elastic mask, deformation data
Plastic mask, deformation data
Adhesive mask, deformation data
Deformation distribution, no outliers
Dissipation distribution, no outliers
Fractions
hard
elastic
plastic
adhesive

PH, Recipe 1

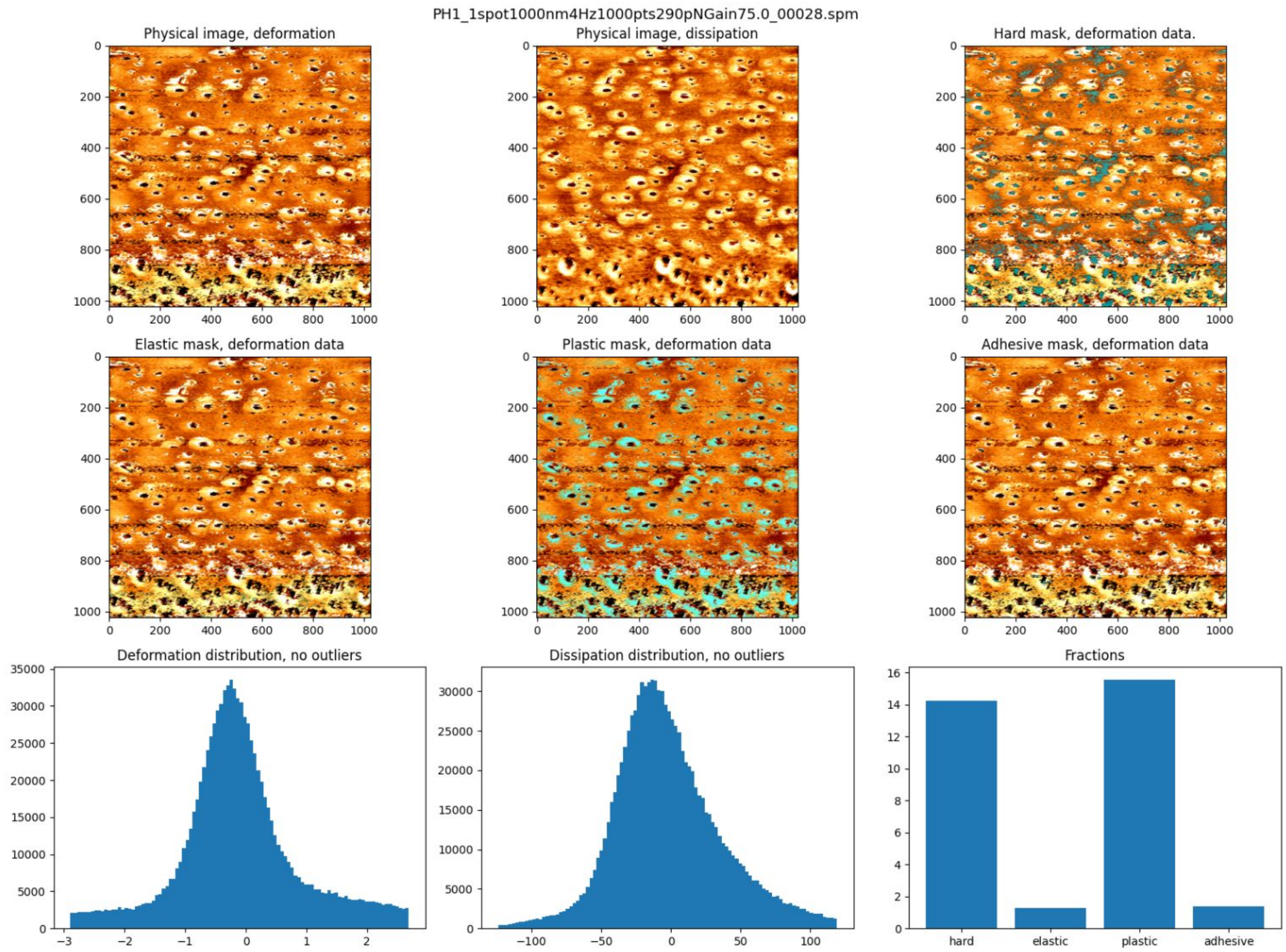
PH1_1spot1000nm4Hz1000pts290pNGain75.0_00028.spm
Physical image, deformation
Physical image, dissipation
Hard mask, deformation data.
Elastic mask, deformation data
Plastic mask, deformation data
Adhesive mask, deformation data
Deformation distribution, no outliers
Dissipation distribution, no outliers
Fractions
hard
elastic
plastic
adhesive

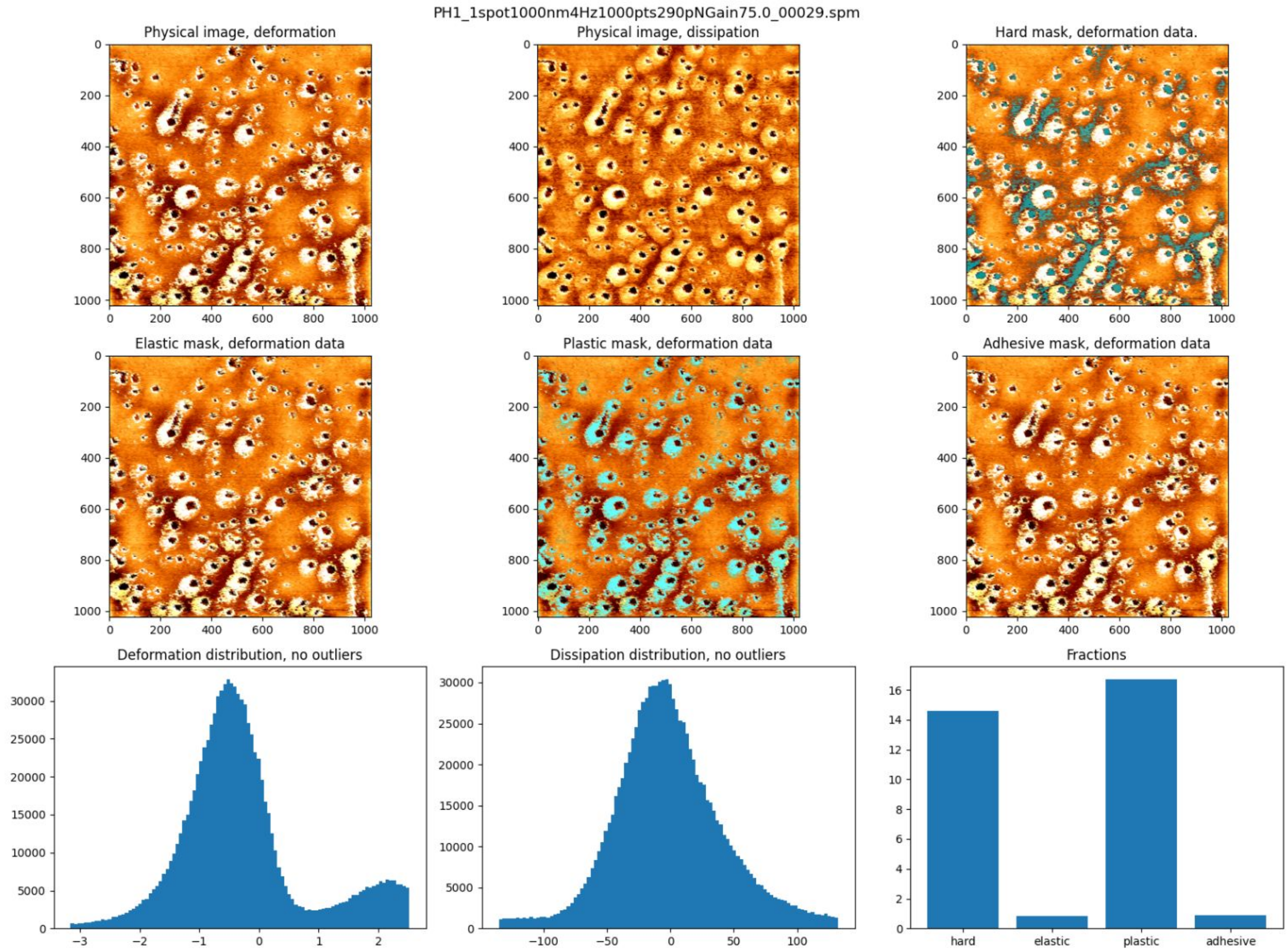
PH1_1spot1000nm4Hz1000pts290pNGain75.0_00029.spm
Physical image, deformation
Physical image, dissipation
Hard mask, deformation data.
Elastic mask, deformation data
Plastic mask, deformation data
Adhesive mask, deformation data
Deformation distribution, no outliers
Dissipation distribution, no outliers
Fractions
hard
elastic
plastic
adhesive

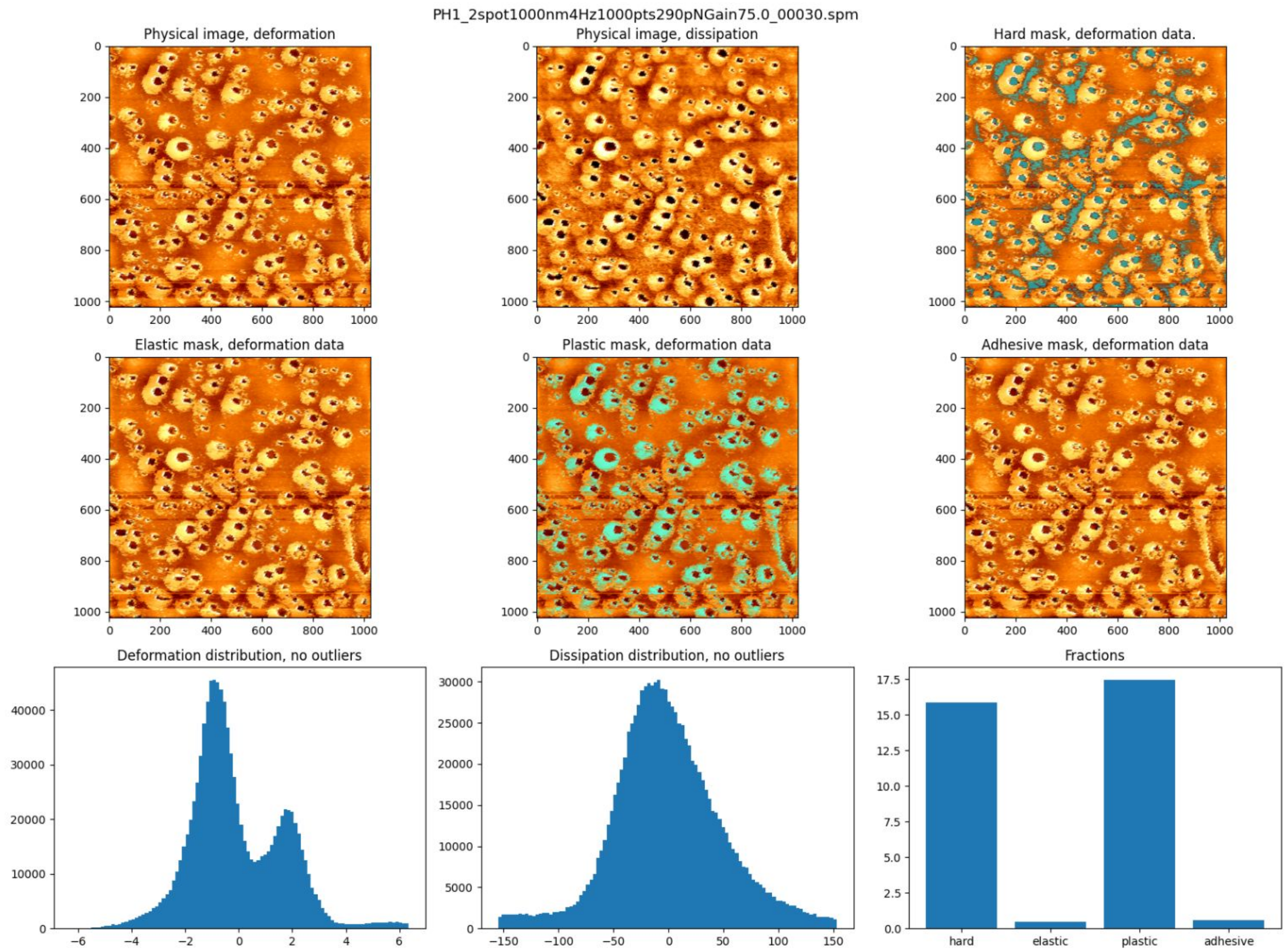
PH1_2spot1000nm4Hz1000pts290pNGain75.0_00030.spm
Physical image, deformation
Physical image, dissipation
Hard mask, deformation data.
Elastic mask, deformation data
Plastic mask, deformation data
Adhesive mask, deformation data
Deformation distribution, no outliers
Dissipation distribution, no outliers
Fractions
hard
elastic
plastic
adhesive

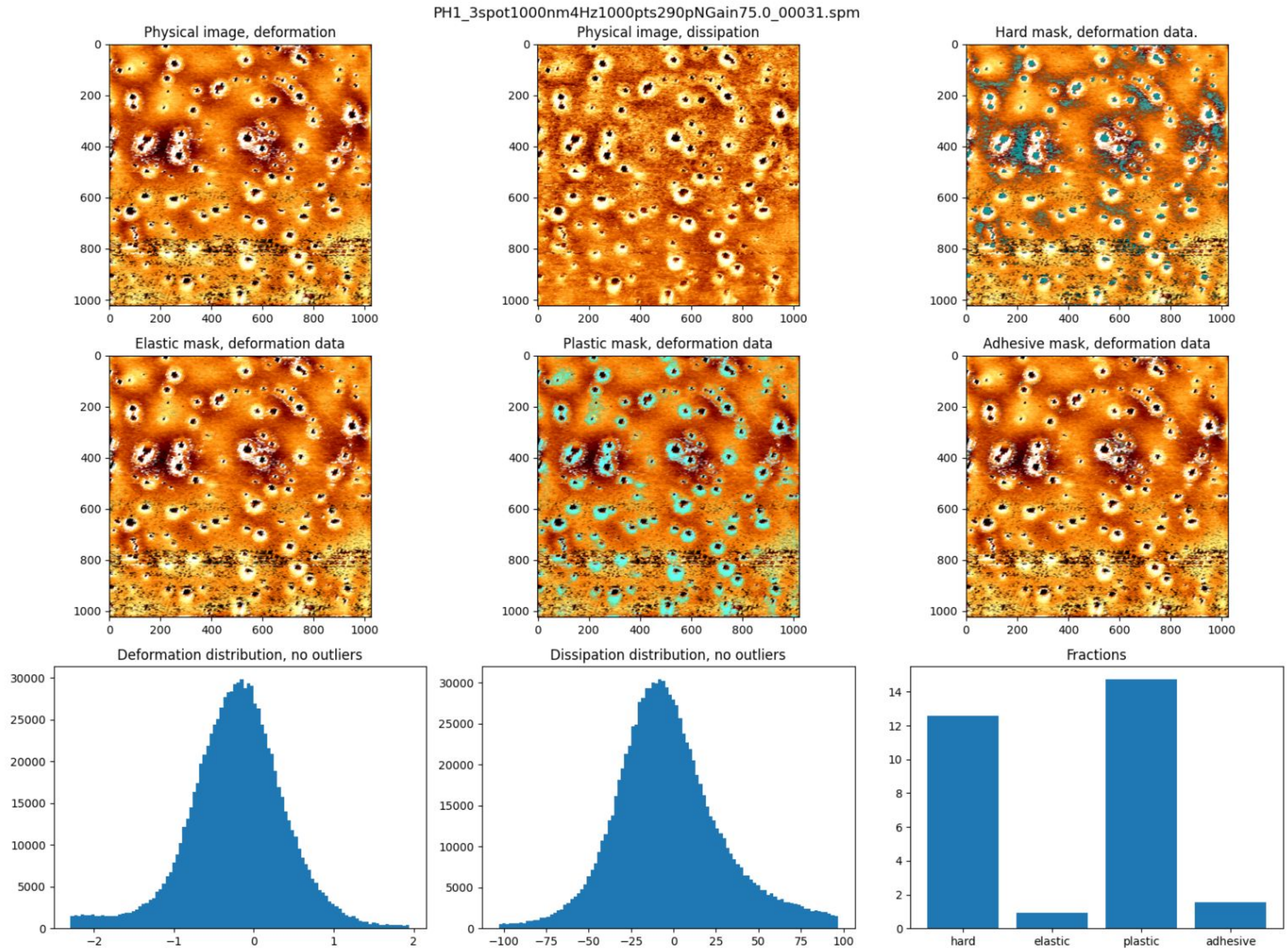
PH1_3spot1000nm4Hz1000pts290pNGain75.0_00031.spm
Physical image, deformation
Physical image, dissipation
Hard mask, deformation data.
Elastic mask, deformation data
Plastic mask, deformation data
Adhesive mask, deformation data
Deformation distribution, no outliers
Dissipation distribution, no outliers
Fractions
hard
elastic
plastic
adhesive

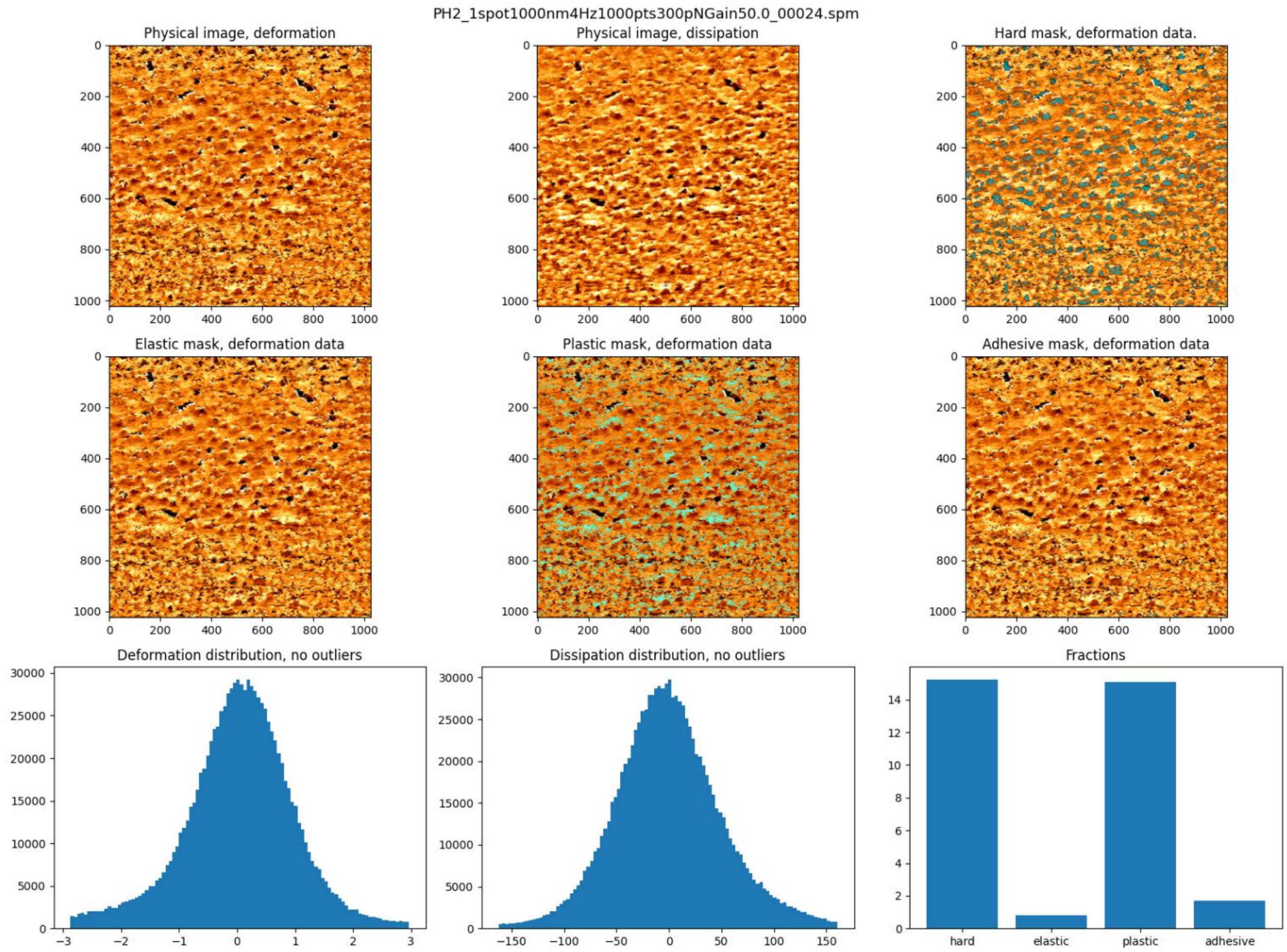
PH2_1spot1000nm4Hz1000pts300pNGain50.0_00024.spm
Physical image, deformation
Physical image, dissipation
Hard mask, deformation data.
Elastic mask, deformation data
Plastic mask, deformation data
Adhesive mask, deformation data
Deformation distribution, no outliers
Dissipation distribution, no outliers
Fractions
hard
elastic
plastic
adhesive

PH Recipe 2

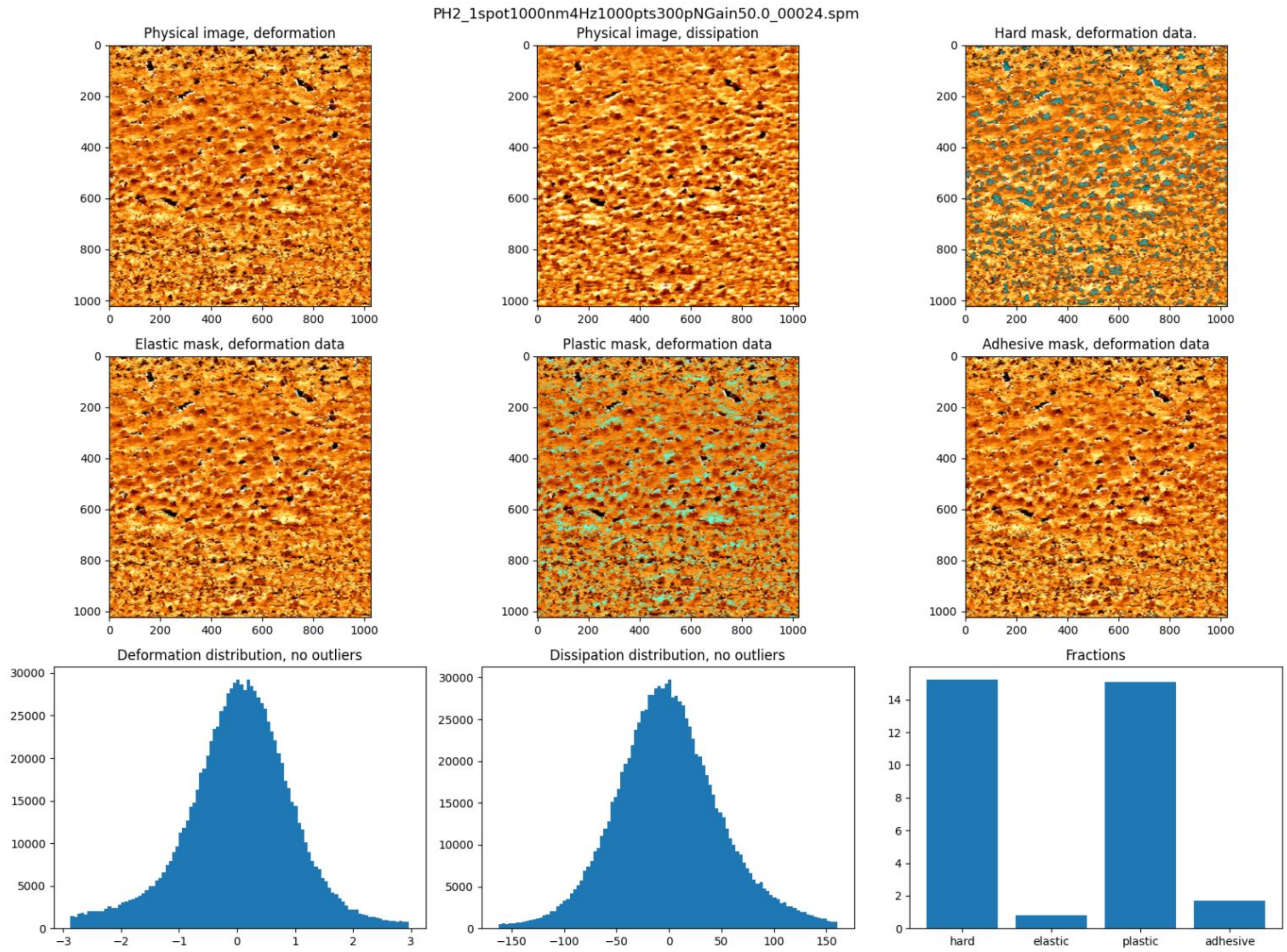

PH2_1spot1000nm4Hz1000pts300pNGain50.0_00024.spm
Physical image, deformation
Physical image, dissipation
Hard mask, deformation data.
Elastic mask, deformation data
Plastic mask, deformation data
Adhesive mask, deformation data
Deformation distribution, no outliers
Dissipation distribution, no outliers
Fractions
hard
elastic
plastic
adhesive

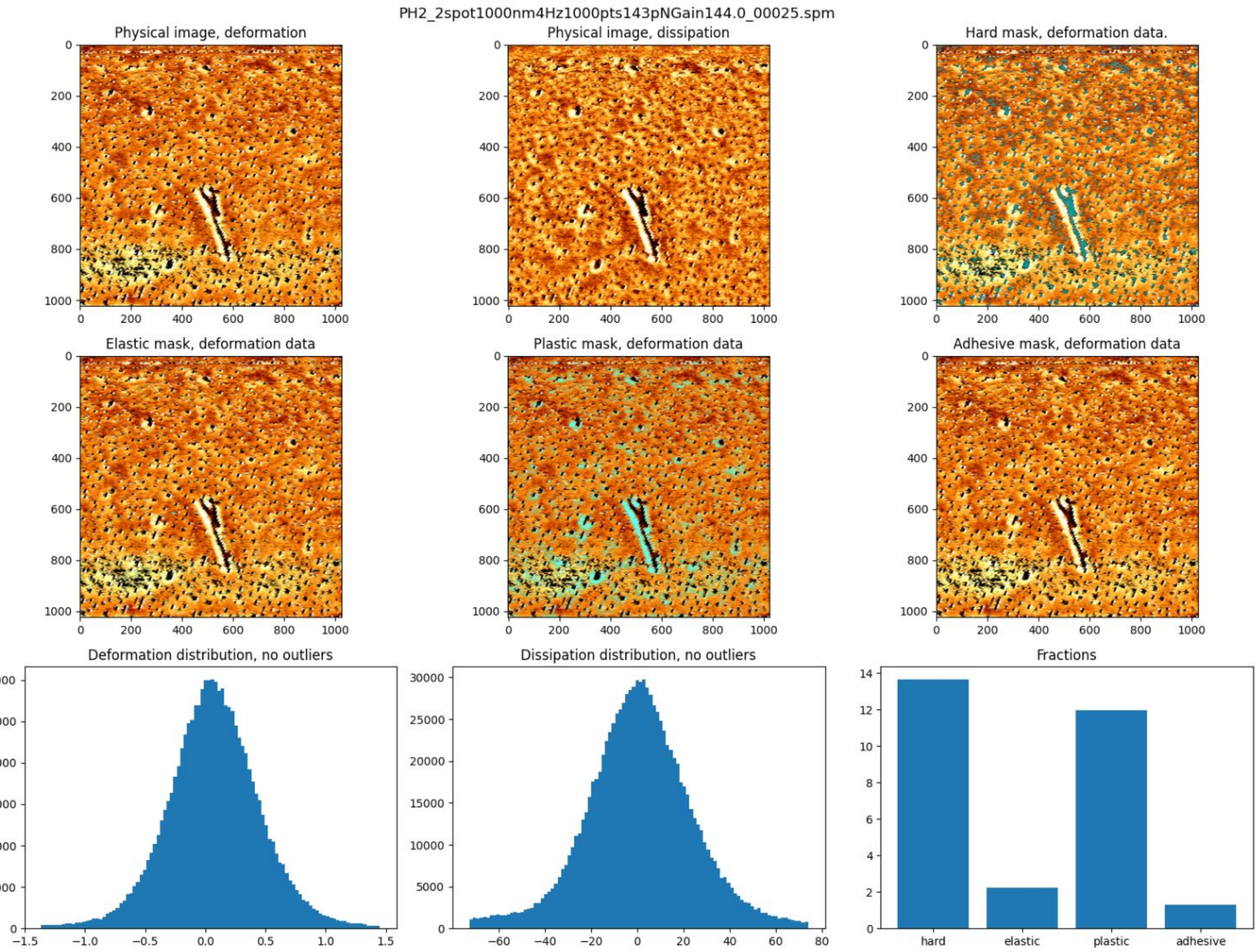

REMOVED from statistics, yet similar output as other results in this group.

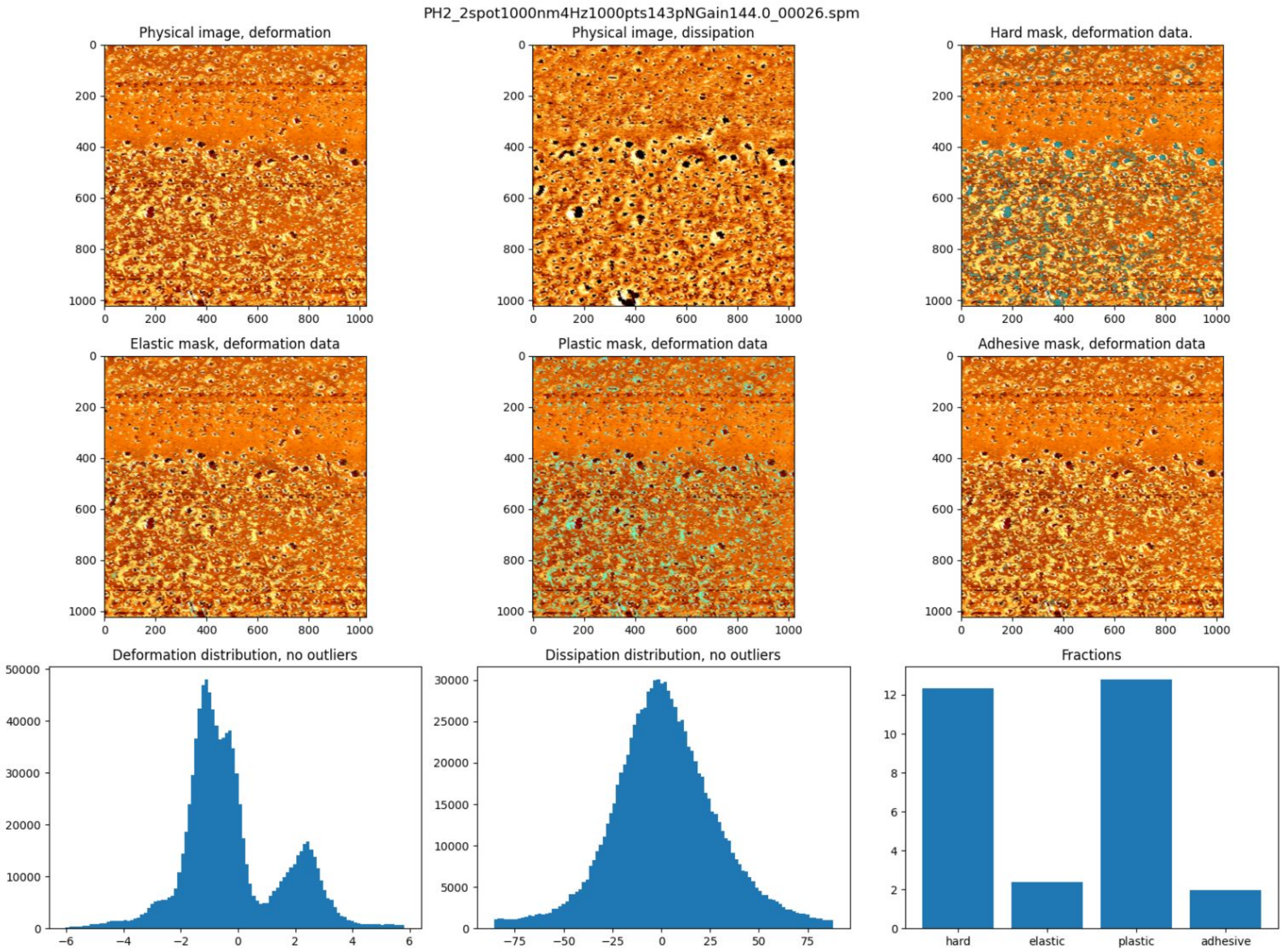
PH2_2spot1000nm4Hz1000pts143pNGain144.0_00026.spm
Physical image, deformation
Physical image, dissipation
Hard mask, deformation data.
Elastic mask, deformation data
Plastic mask, deformation data
Adhesive mask, deformation data
Deformation distribution, no outliers
Dissipation distribution, no outliers
Fractions
hard
elastic
plastic
adhesive

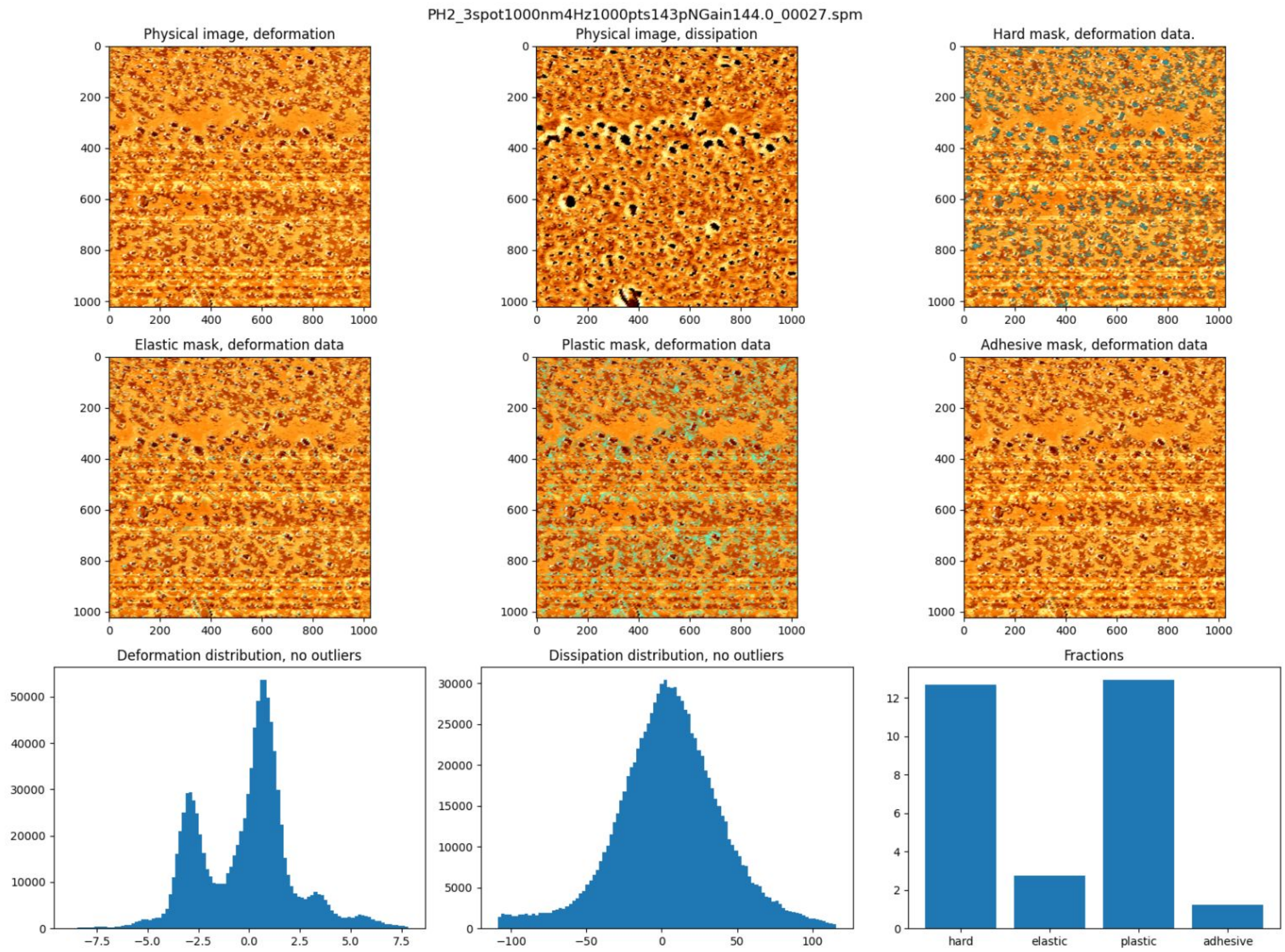
PH2_3spot1000nm4Hz1000pts143pNGain144.0_00027.spm
Physical image, deformation
Physical image, dissipation
Hard mask, deformation data.
Elastic mask, deformation data
Plastic mask, deformation data
Adhesive mask, deformation data
Deformation distribution, no outliers
Dissipation distribution, no outliers
Fractions
hard
elastic
plastic
adhesive

PH Recipe 3, type 1

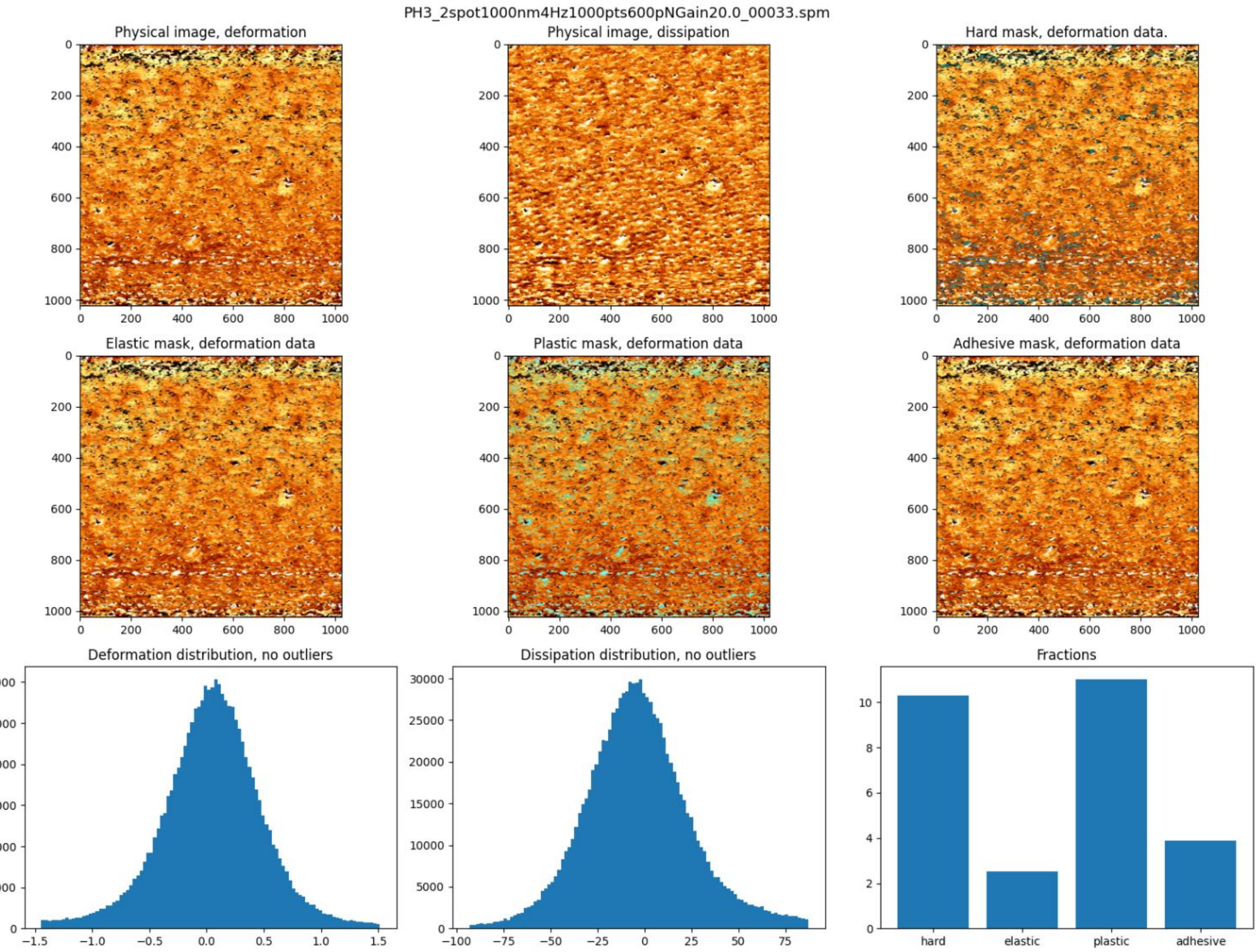

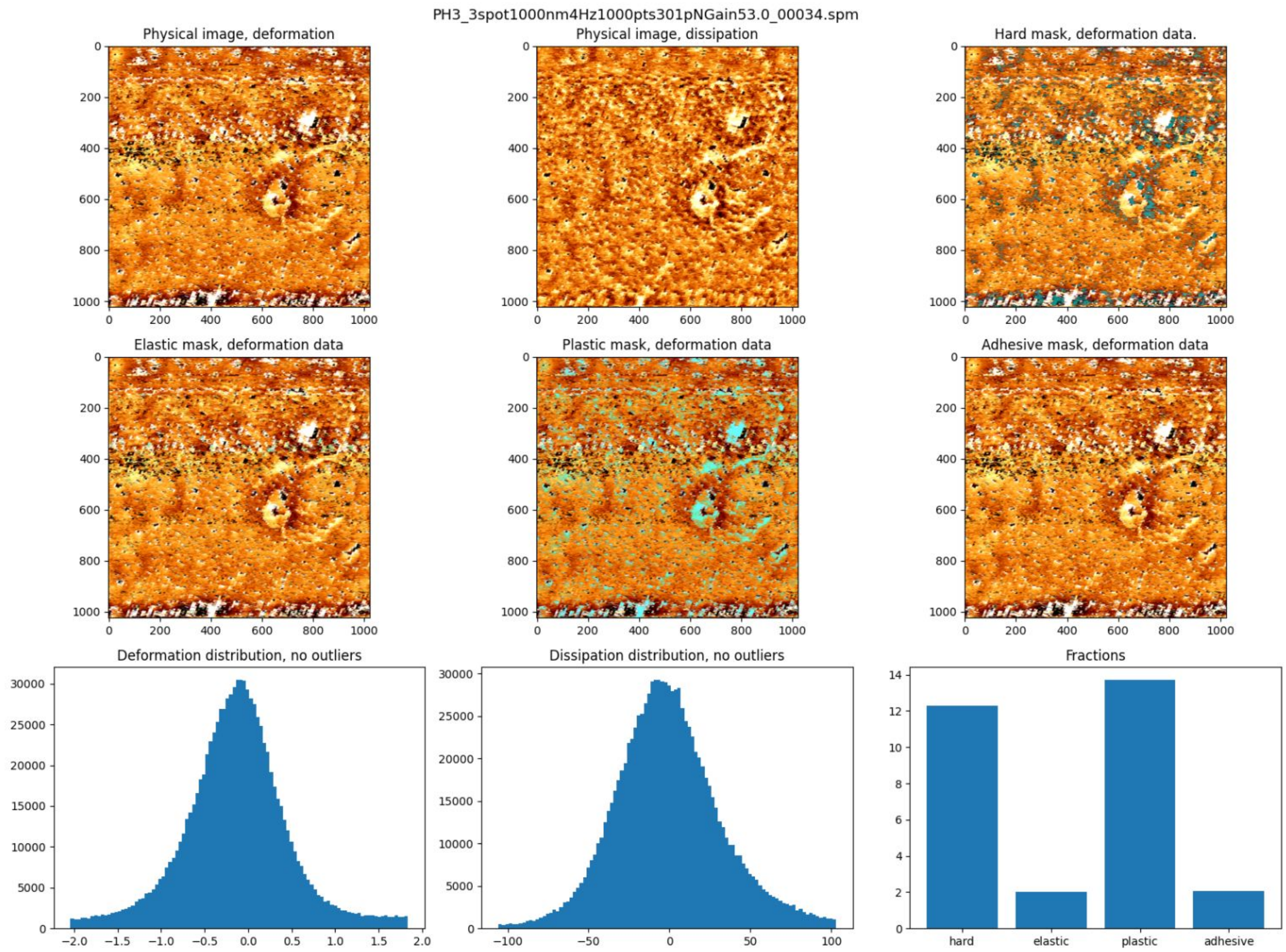
PH3_3spot1000nm4Hz1000pts301pNGain53.0_00034.spm
Physical image, deformation
Physical image, dissipation
Hard mask, deformation data.
Elastic mask, deformation data
Plastic mask, deformation data
Adhesive mask, deformation data
Deformation distribution, no outliers
Dissipation distribution, no outliers
Fractions
hard
elastic
plastic
adhesive

PH Recipe 3, type 2

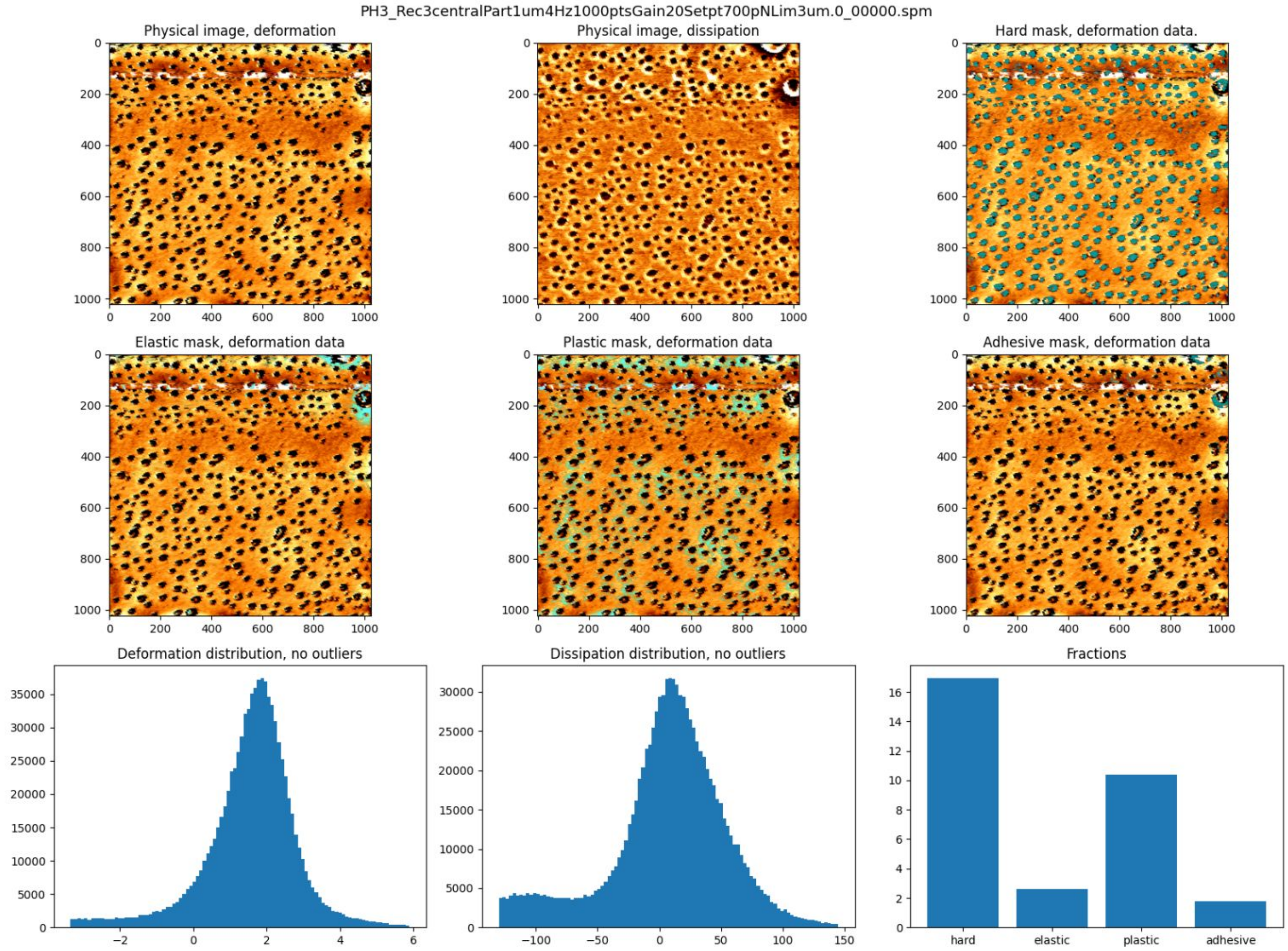

PH Recipe 4

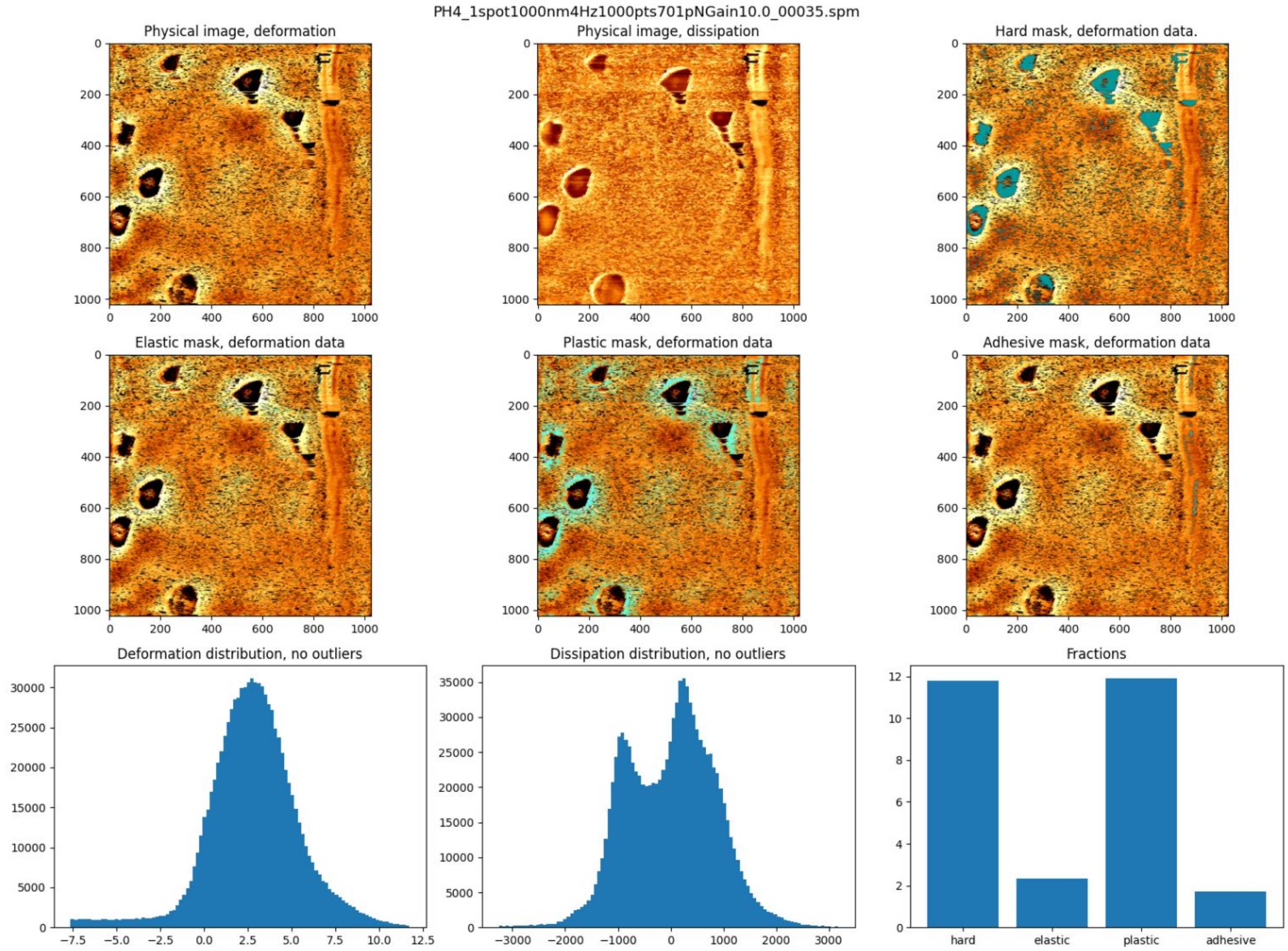
PH4_1spot1000nm4Hz1000pts701pNGain10.0_00035.spm
Physical image, deformation
Physical image, dissipation
Hard mask, deformation data.
Elastic mask, deformation data
Plastic mask, deformation data
Adhesive mask, deformation data
Deformation distribution, no outliers
Dissipation distribution, no outliers
Fractions
hard
elastic
plastic
adhesive

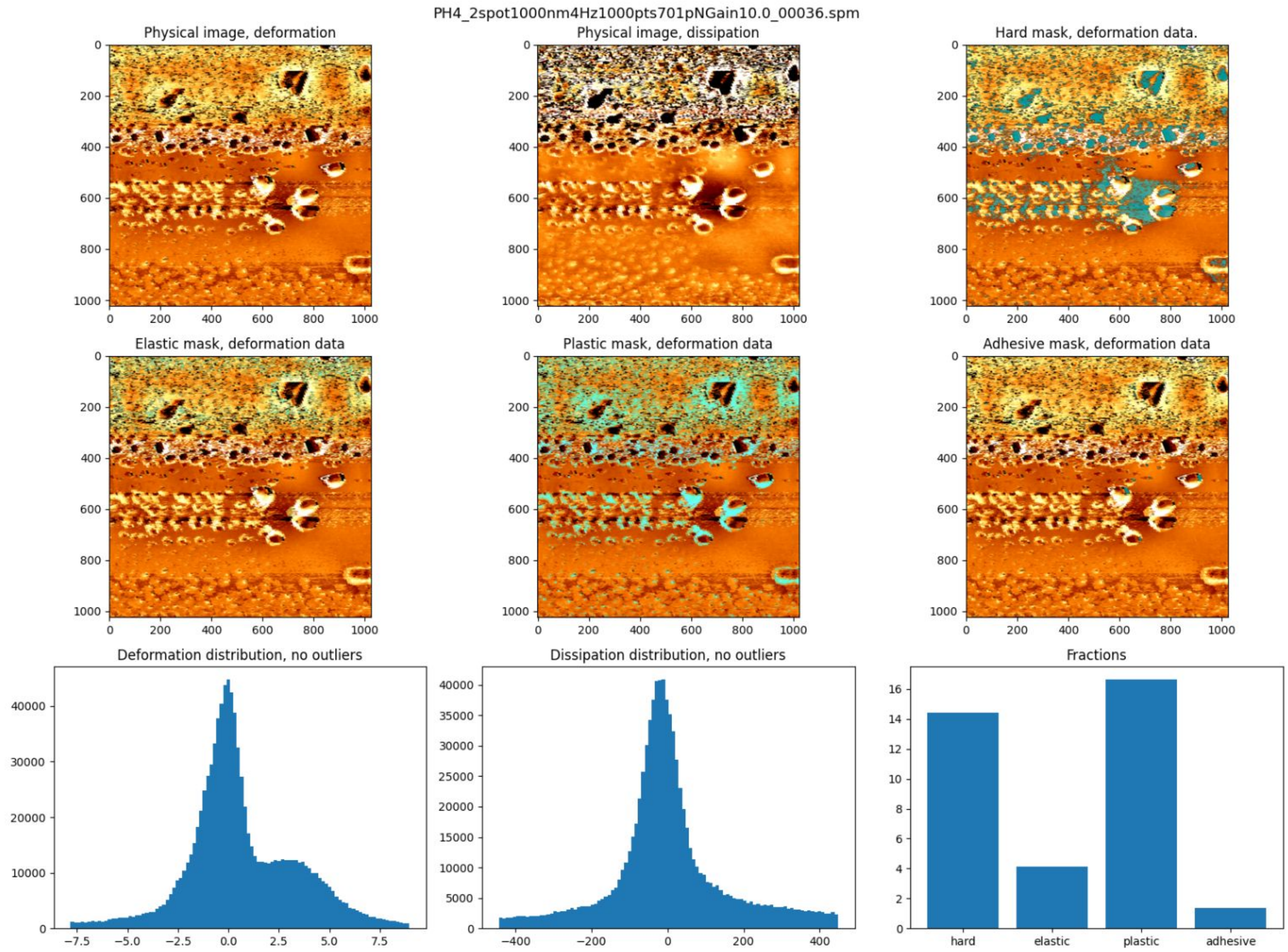
PH4_2spot1000nm4Hz1000pts701pNGain10.0_00036.spm
Physical image, deformation
Physical image, dissipation
Hard mask, deformation data.
Elastic mask, deformation data
Plastic mask, deformation data
Adhesive mask, deformation data
Deformation distribution, no outliers
Dissipation distribution, no outliers
Fractions
hard
elastic
plastic
adhesive

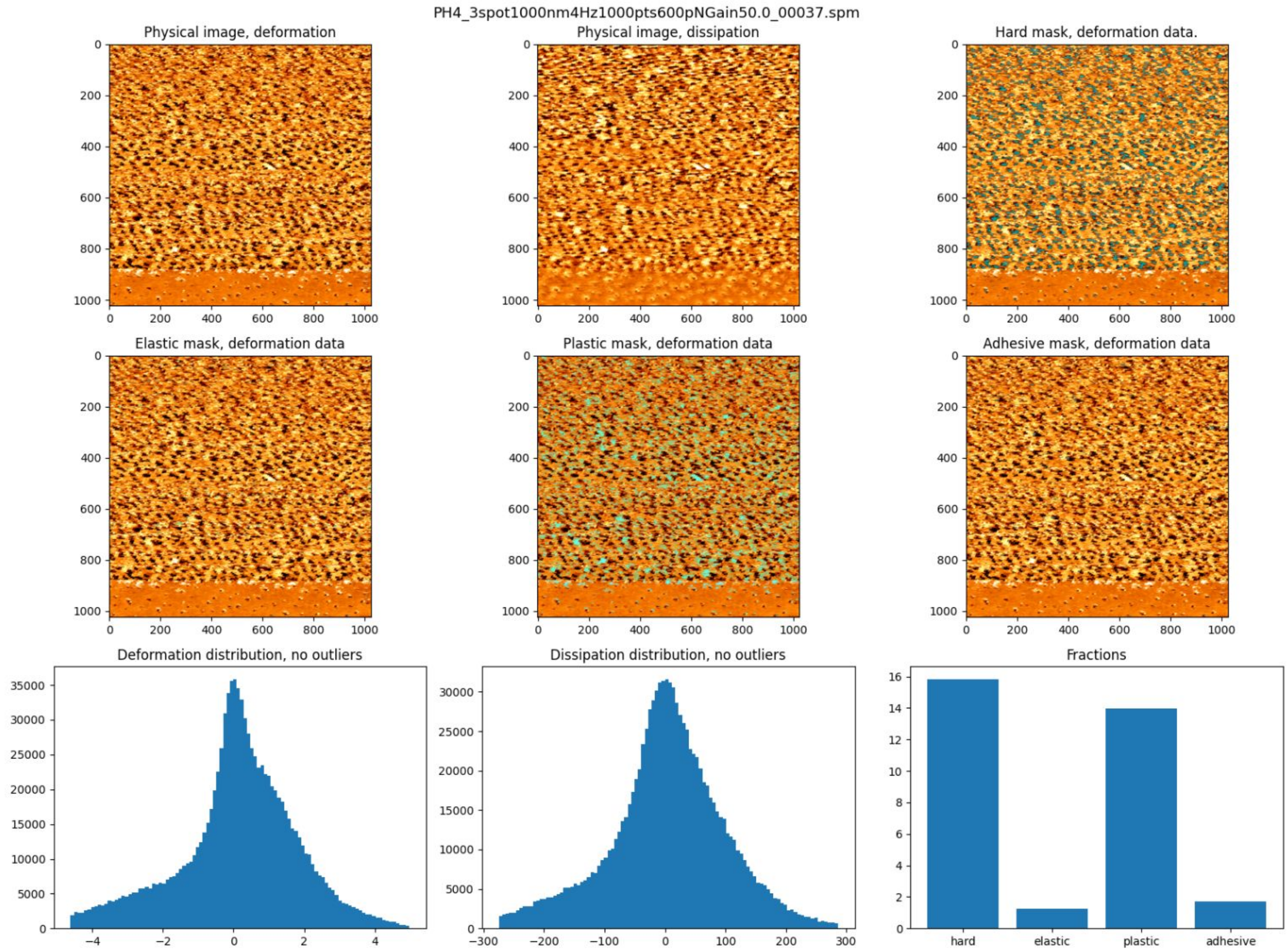
PH4_3spot1000nm4Hz1000pts600pNGain50.0_00037.spm
Physical image, deformation
Physical image, dissipation
Hard mask, deformation data.
Elastic mask, deformation data
Plastic mask, deformation data
Adhesive mask, deformation data
Deformation distribution, no outliers
Dissipation distribution, no outliers
Fractions
hard
elastic
plastic
adhesive

PH Recipe 4, type 2

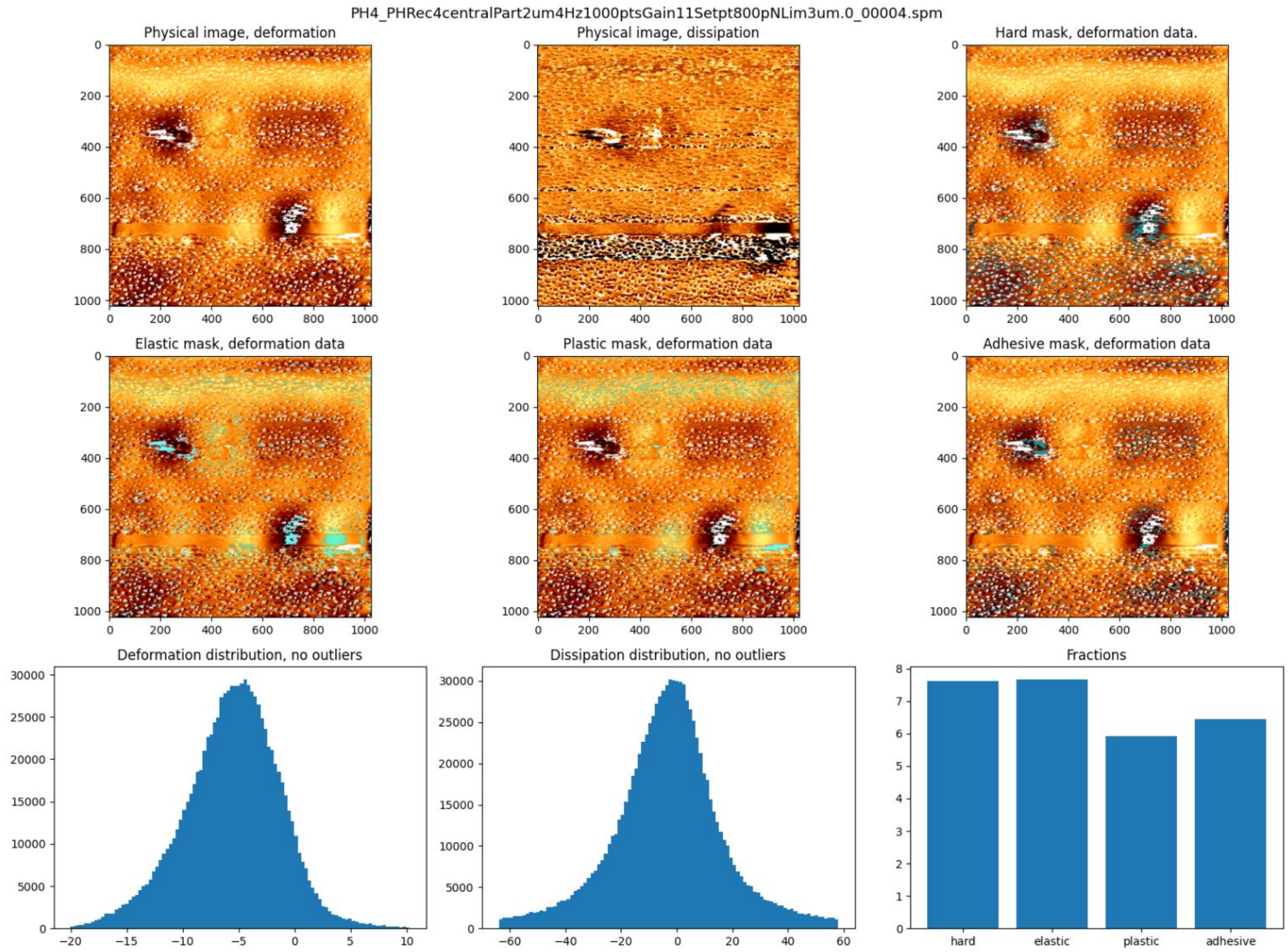

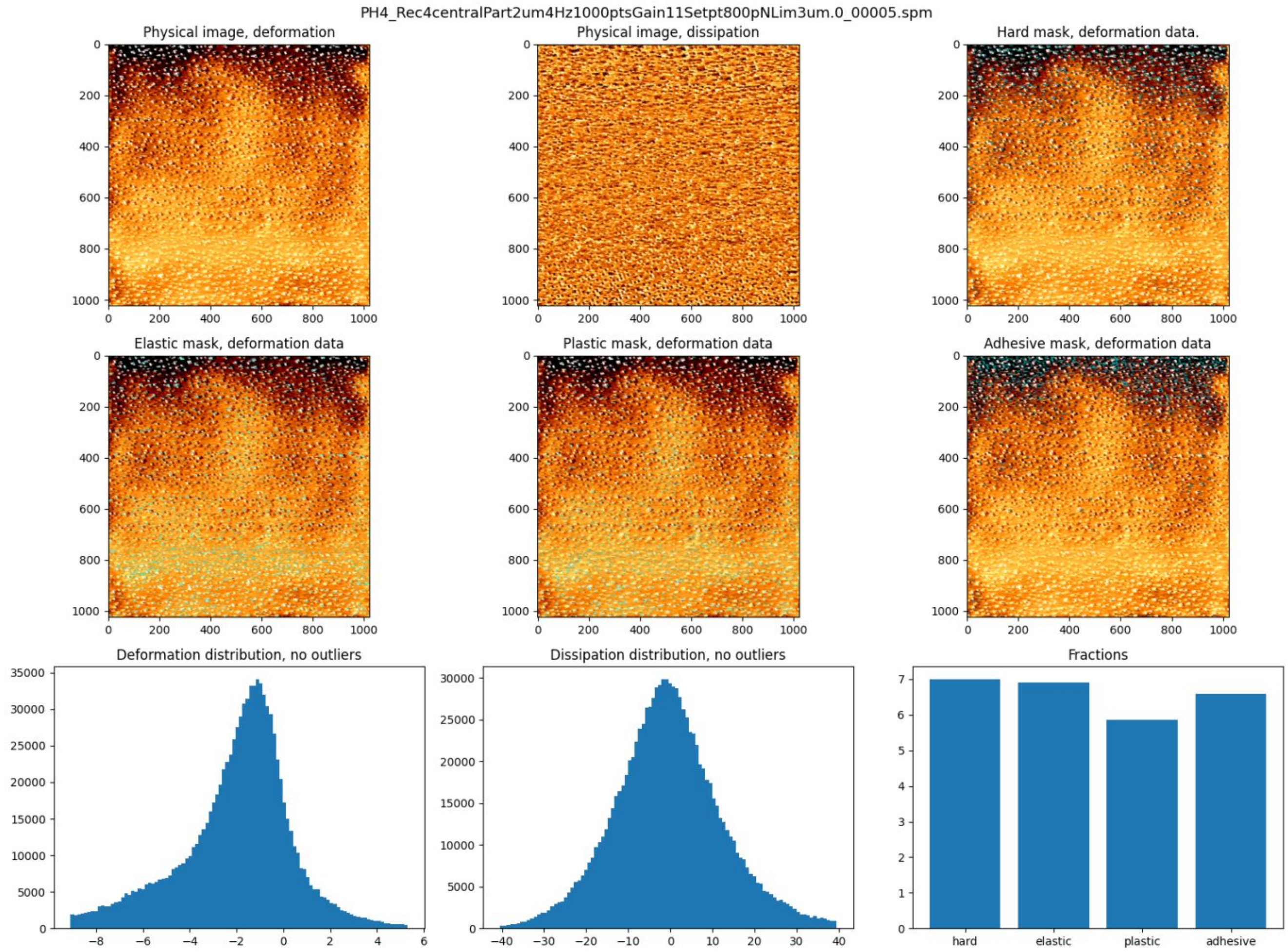
PH4_Rec4centralPart2um4Hz1000ptsGain11Setpt800pNLim3um.0_00005.spm
Physical image, deformation
Physical image, dissipation
Hard mask, deformation data.
Elastic mask, deformation data
Plastic mask, deformation data
Adhesive mask, deformation data
Deformation distribution, no outliers
Dissipation distribution, no outliers
Fractions
hard
elastic
plastic
adhesive

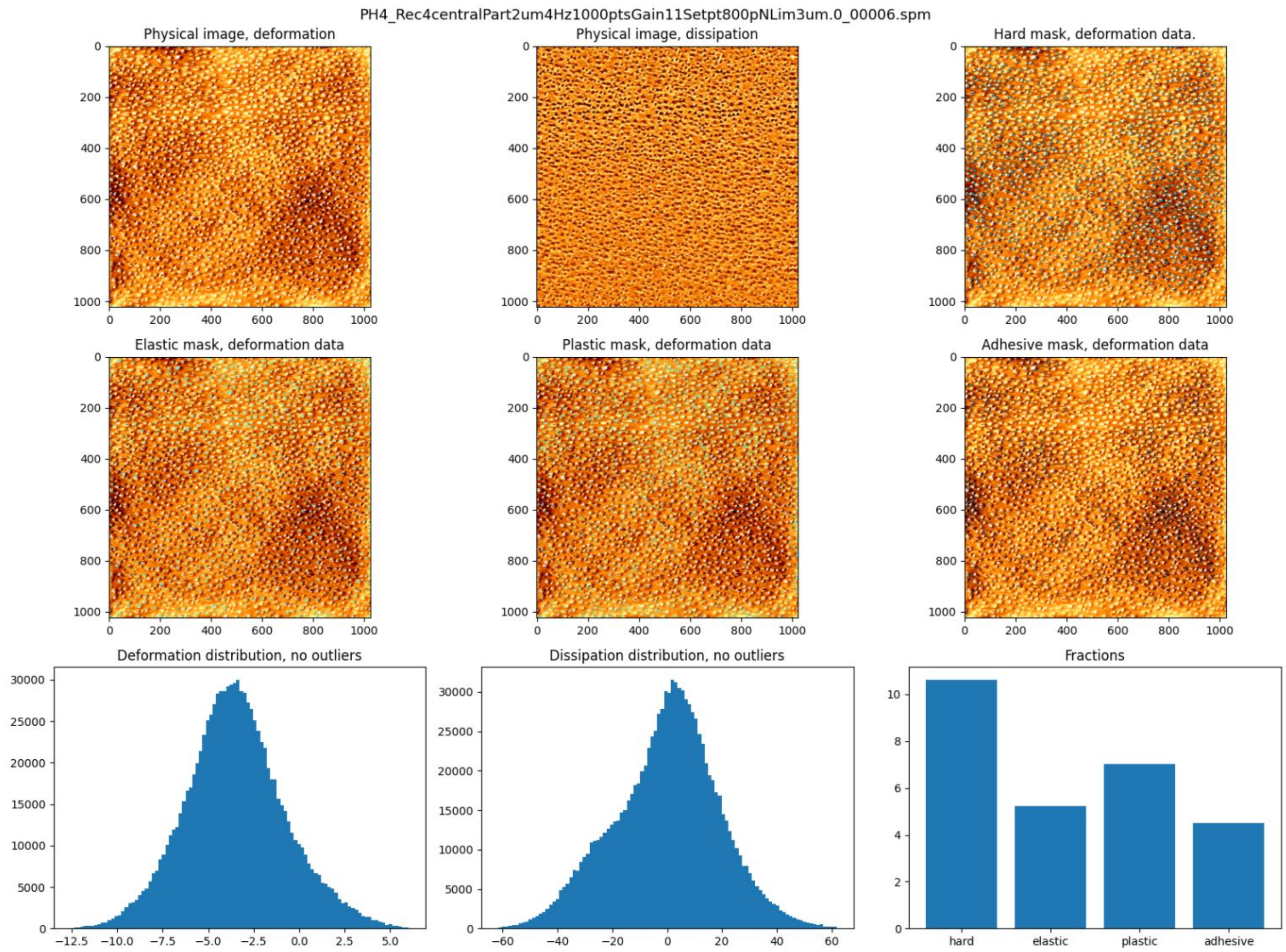
PH4_Rec4centralPart2um4Hz1000ptsGain11Setpt800pNLim3um.0_00006.spm
Physical image, deformation
Physical image, dissipation
Hard mask, deformation data.
Elastic mask, deformation data
Plastic mask, deformation data
Adhesive mask, deformation data
Deformation distribution, no outliers
Dissipation distribution, no outliers
Fractions
hard
elastic
plastic
adhesive

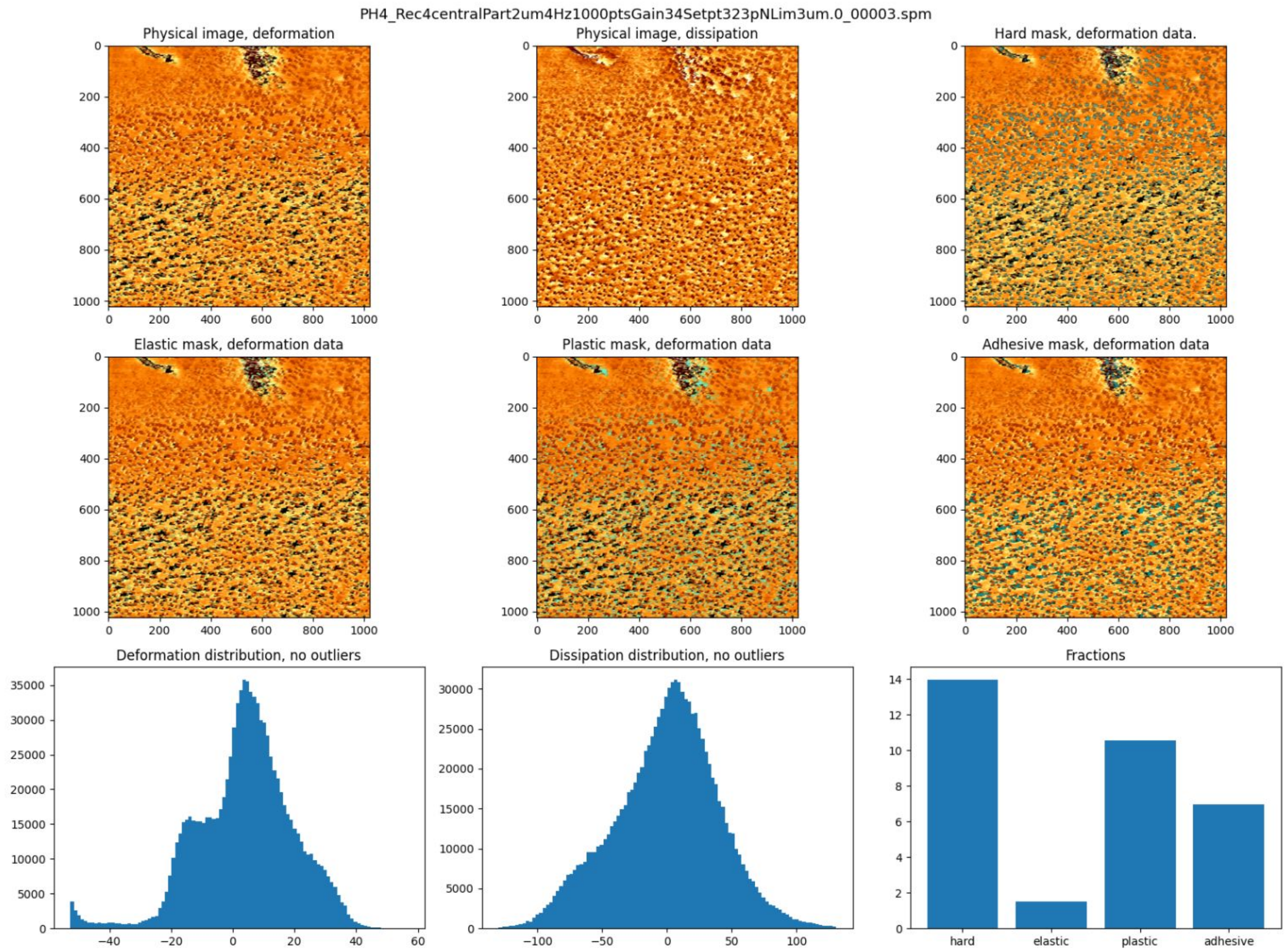
PH4_Rec4centralPart2um4Hz1000ptsGain34Setpt323pNLim3um.0_00003.spm
Physical image, deformation
Physical image, dissipation
Hard mask, deformation data.
Elastic mask, deformation data
Plastic mask, deformation data
Adhesive mask, deformation data
Deformation distribution, no outliers
Dissipation distribution, no outliers
Fractions
hard
elastic
plastic
adhesive

PH Recipe 5

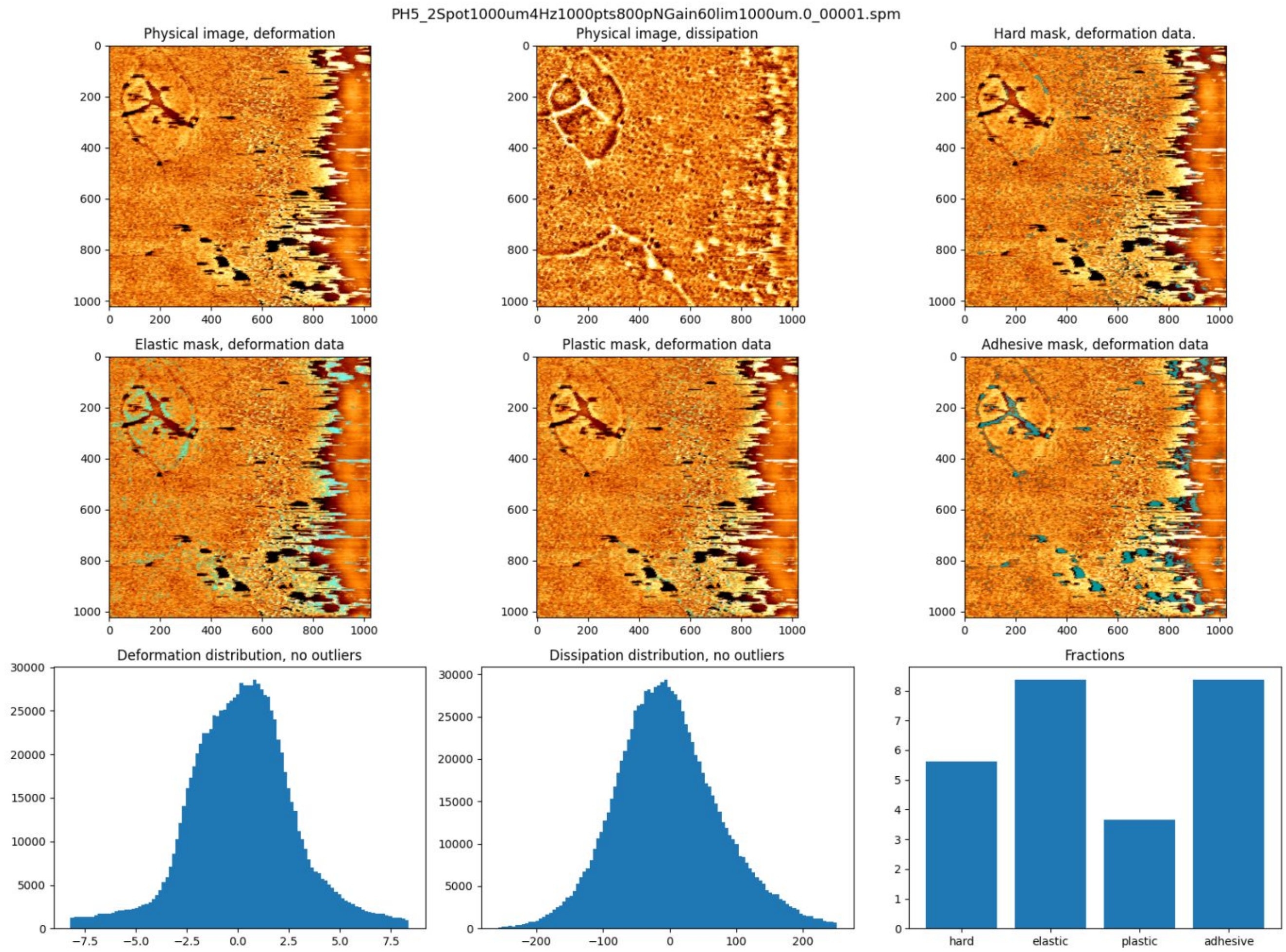
PH5_2Spot1000um4Hz1000pts800pNGain60lim1000um.0_00001.spm
Physical image, deformation
Physical image, dissipation
Hard mask, deformation data.
Elastic mask, deformation data
Plastic mask, deformation data
Adhesive mask, deformation data
Deformation distribution, no outliers
Dissipation distribution, no outliers
Fractions
hard
elastic
plastic
adhesive

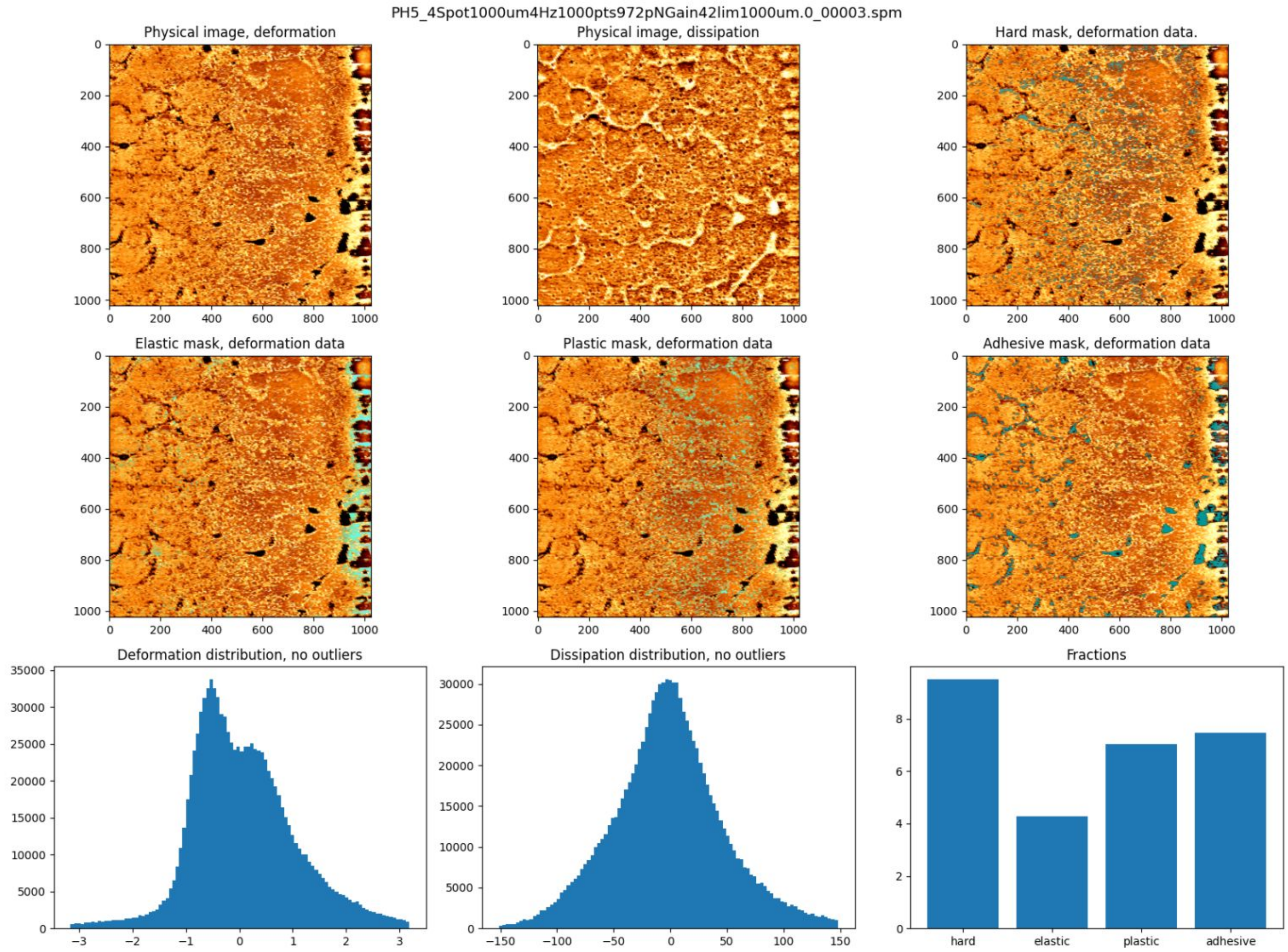
PH5_4Spot1000um4Hz1000pts972pNGain42lim1000um.0_00003.spm
Physical image, deformation
Physical image, dissipation
Hard mask, deformation data.
Elastic mask, deformation data
Plastic mask, deformation data
Adhesive mask, deformation data
Deformation distribution, no outliers
Dissipation distribution, no outliers
Fractions
hard
elastic
plastic
adhesive

PH Recipe 6

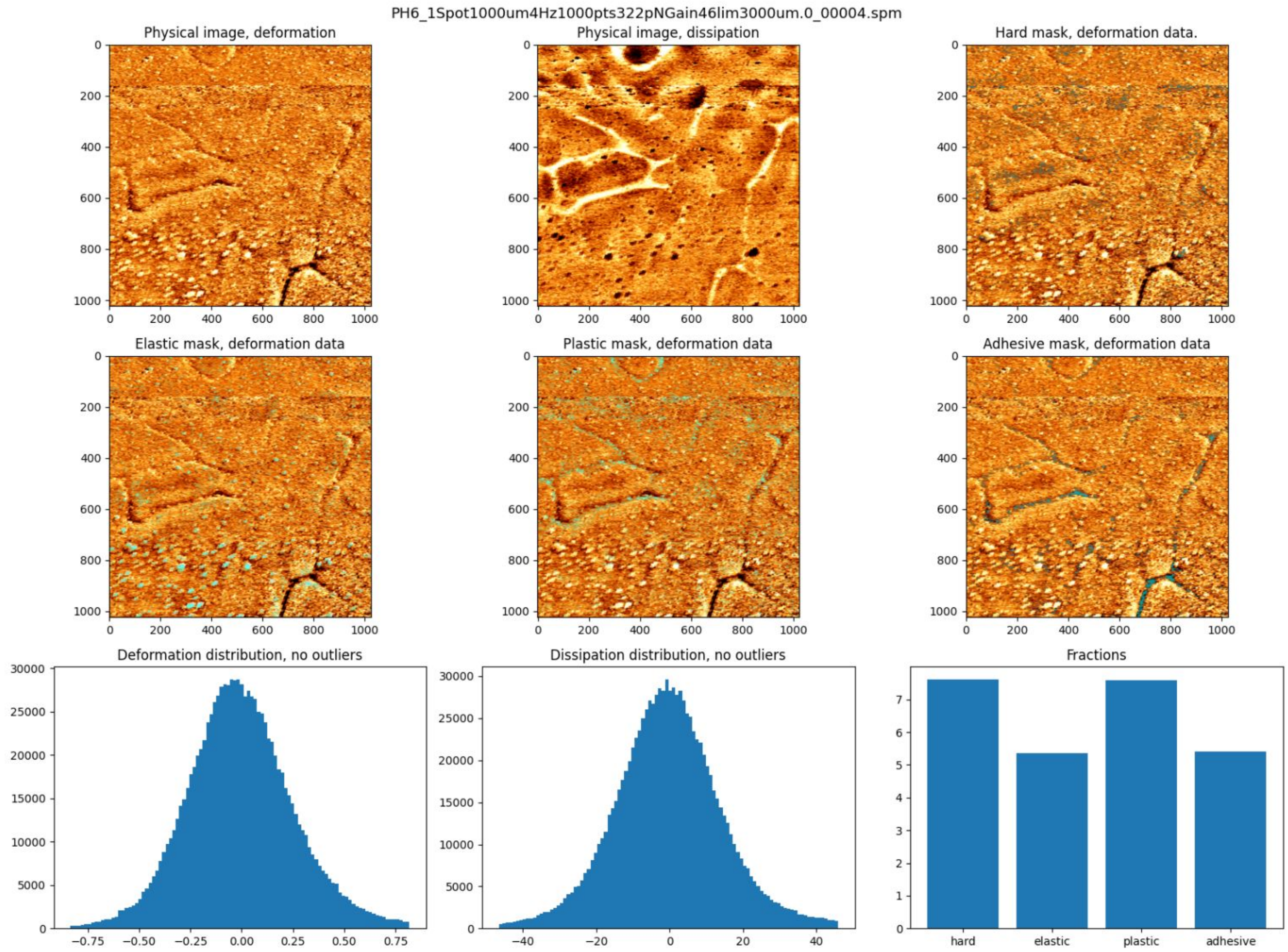
PH6_1Spot1000um4Hz1000pts322pNGain46lim3000um.0_00004.spm
Physical image, deformation
Physical image, dissipation
Hard mask, deformation data.
Elastic mask, deformation data
Plastic mask, deformation data
Adhesive mask, deformation data
Deformation distribution, no outliers
Dissipation distribution, no outliers
Fractions
hard
elastic
plastic
adhesive

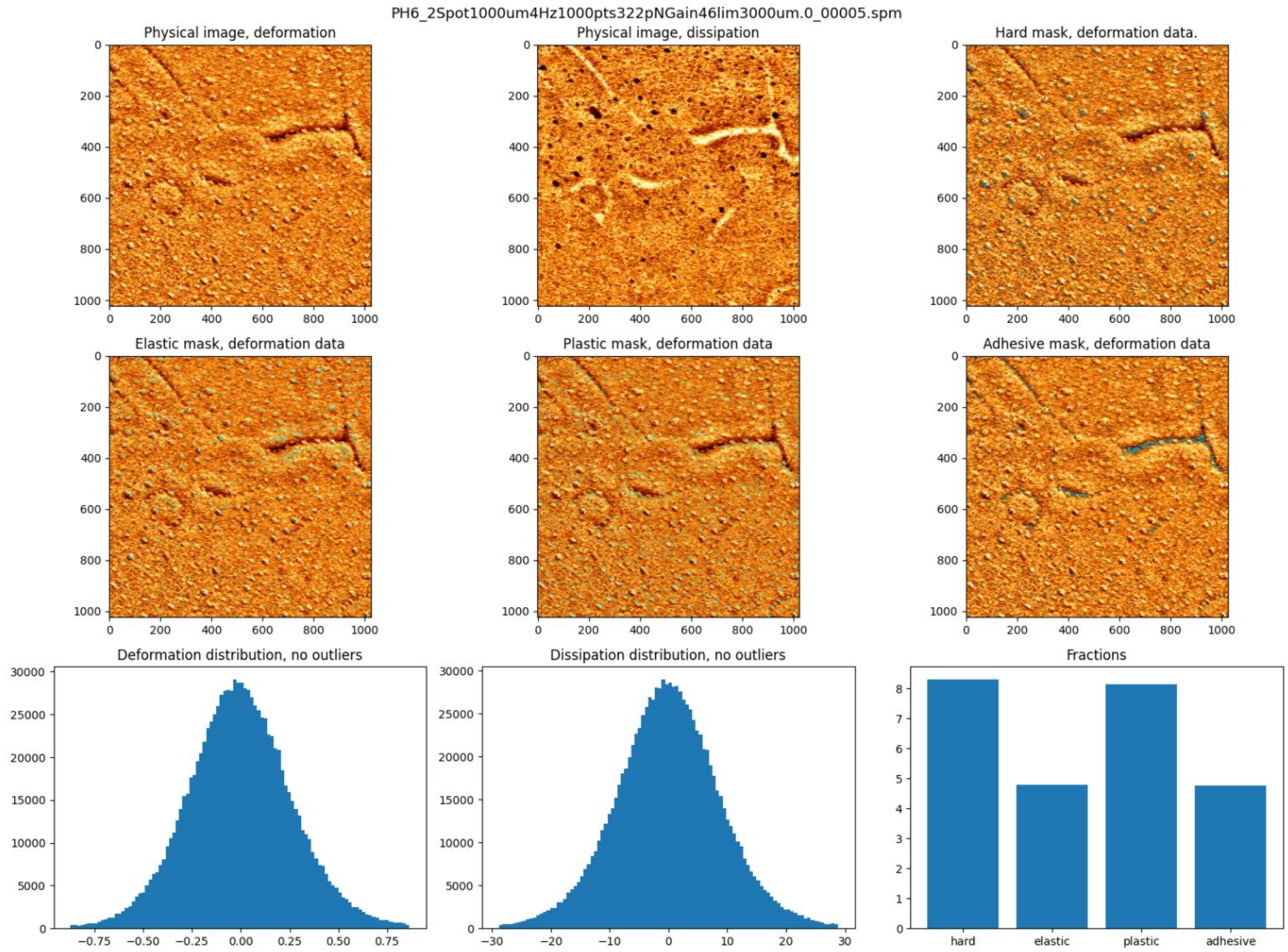

PH6_2Spot1000um4Hz1000pts322pNGain46lim3000um.0_00005.spm
Physical image, deformation
Physical image, dissipation
Hard mask, deformation data.
Elastic mask, deformation data
Plastic mask, deformation data
Adhesive mask, deformation data
Deformation distribution, no outliers
Dissipation distribution, no outliers
Fractions
hard
elastic
plastic
adhesive

TRT-P, Recipe 1

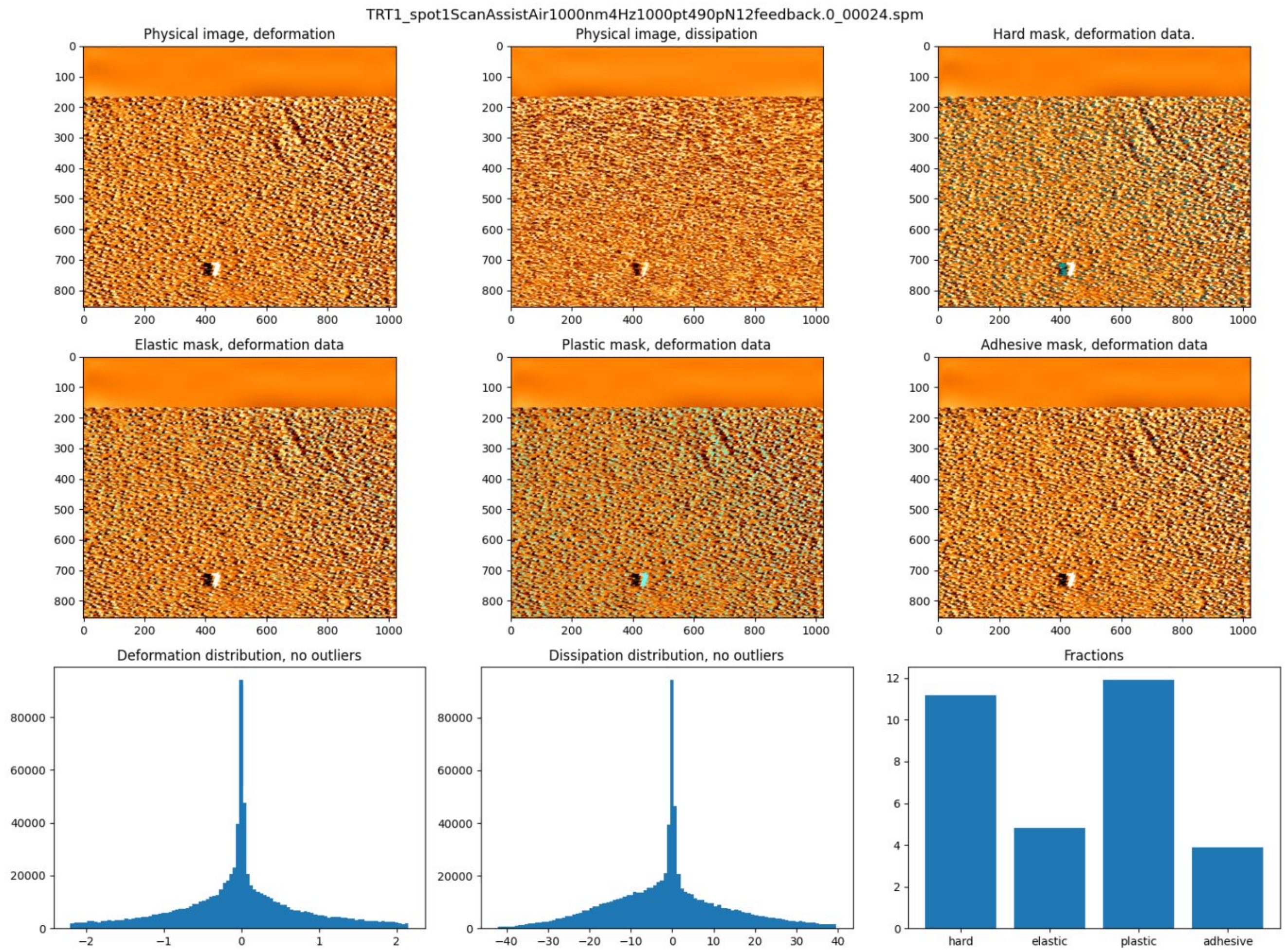

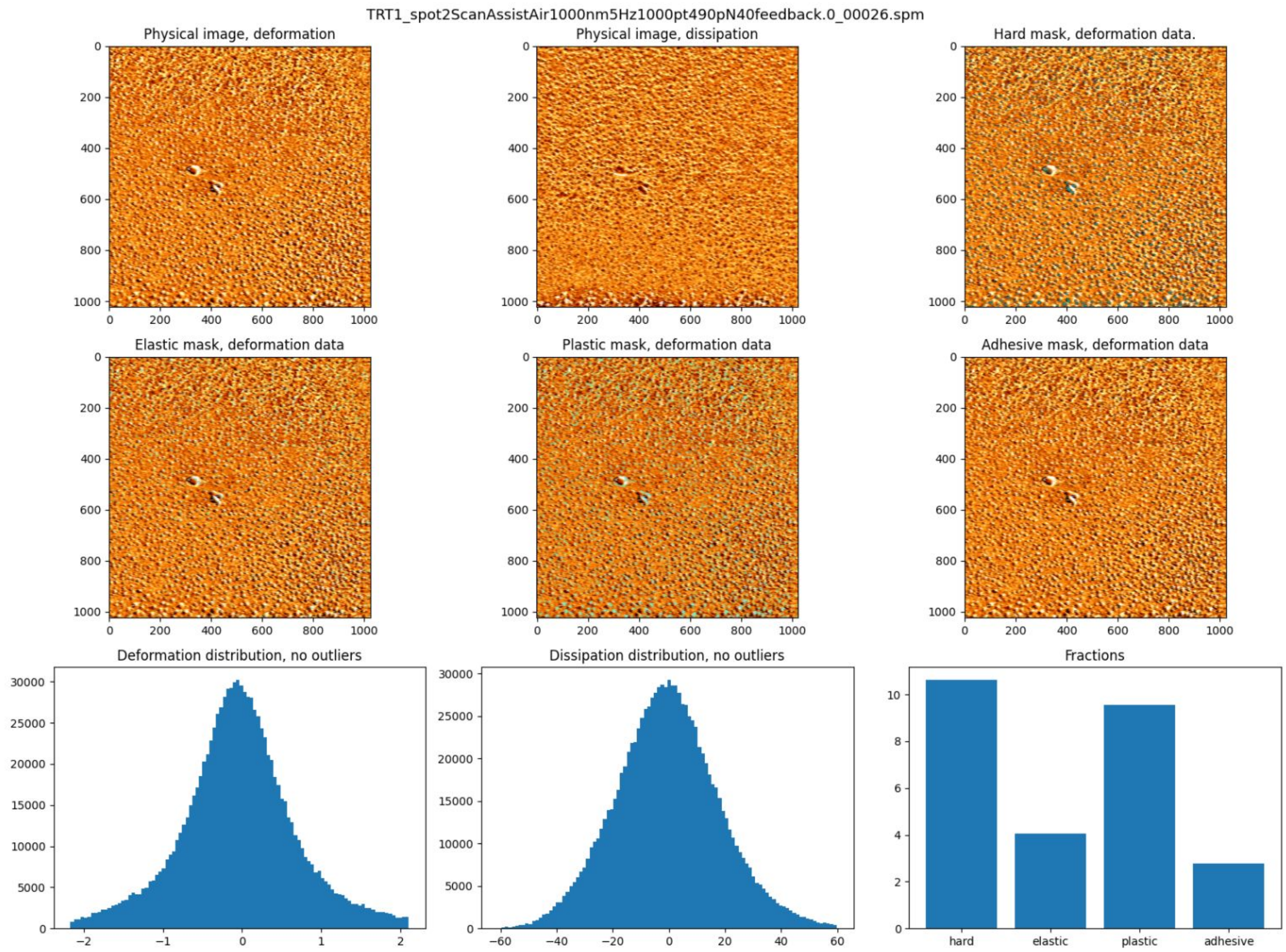

TRT1_spot2ScanAssistAir1000nm5Hz1000pt490pN40feedback.0_00026.spm
Physical image, deformation
Physical image, dissipation
Hard mask, deformation data.
Elastic mask, deformation data
Plastic mask, deformation data
Adhesive mask, deformation data
Deformation distribution, no outliers
Dissipation distribution, no outliers
Fractions
hard
elastic
plastic
adhesive

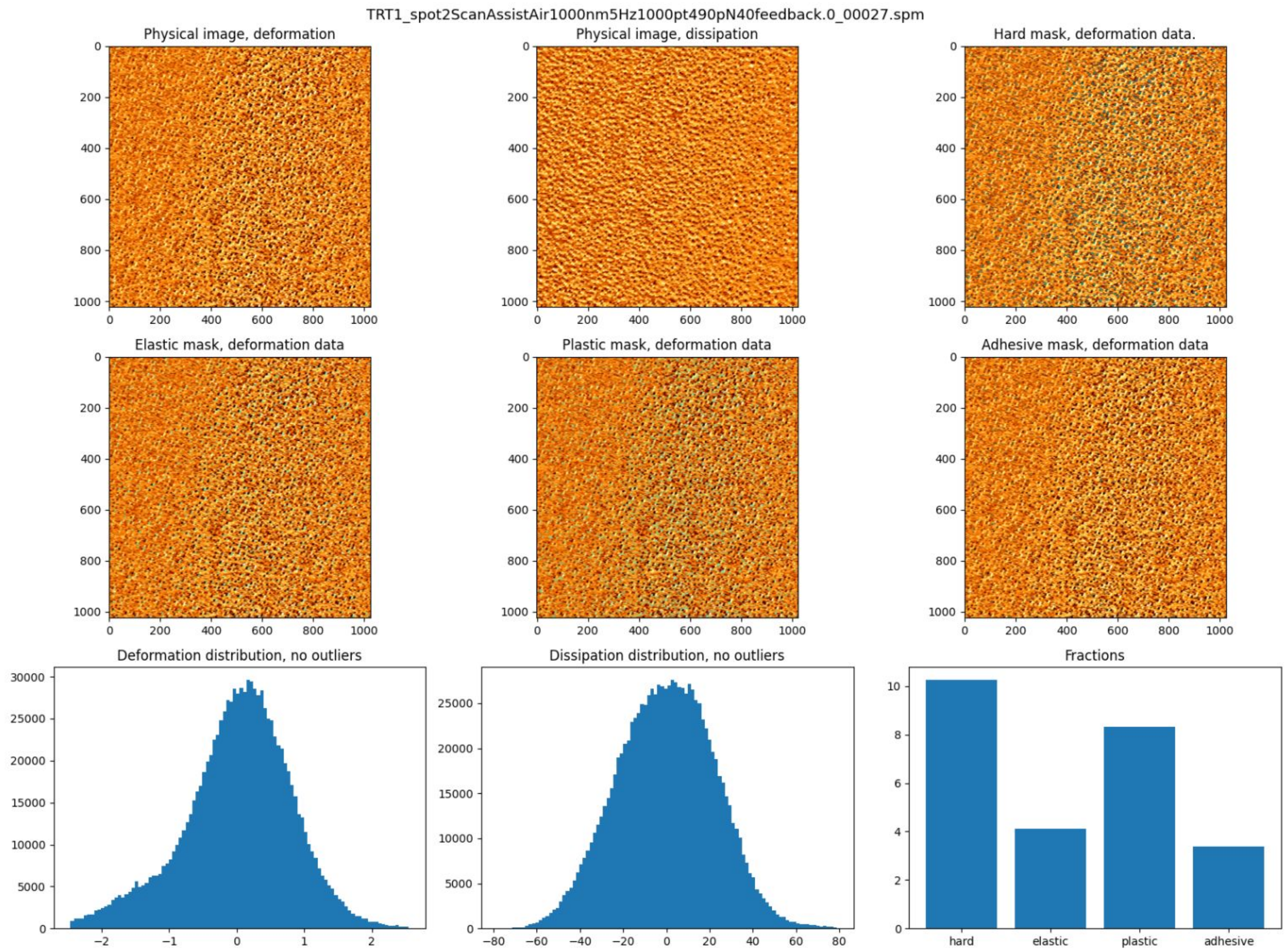
TRT1_spot2ScanAssistAir1000nm5Hz1000pt490pN40feedback.0_00027.spm
Physical image, deformation
Physical image, dissipation
Hard mask, deformation data.
Elastic mask, deformation data
Plastic mask, deformation data
Adhesive mask, deformation data
Deformation distribution, no outliers
Dissipation distribution, no outliers
Fractions
hard
elastic
plastic
adhesive

TRT-P, Recipe 2

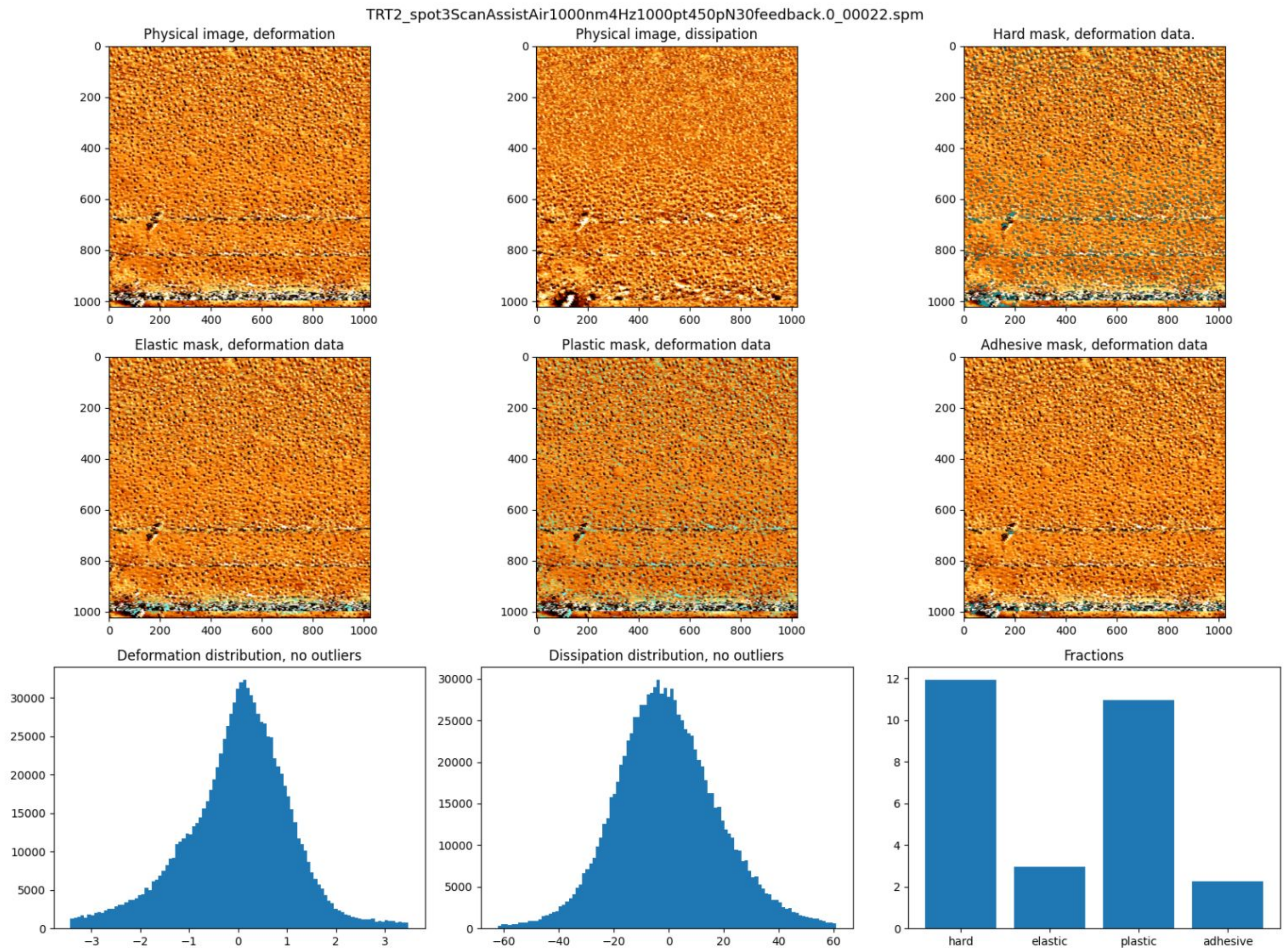

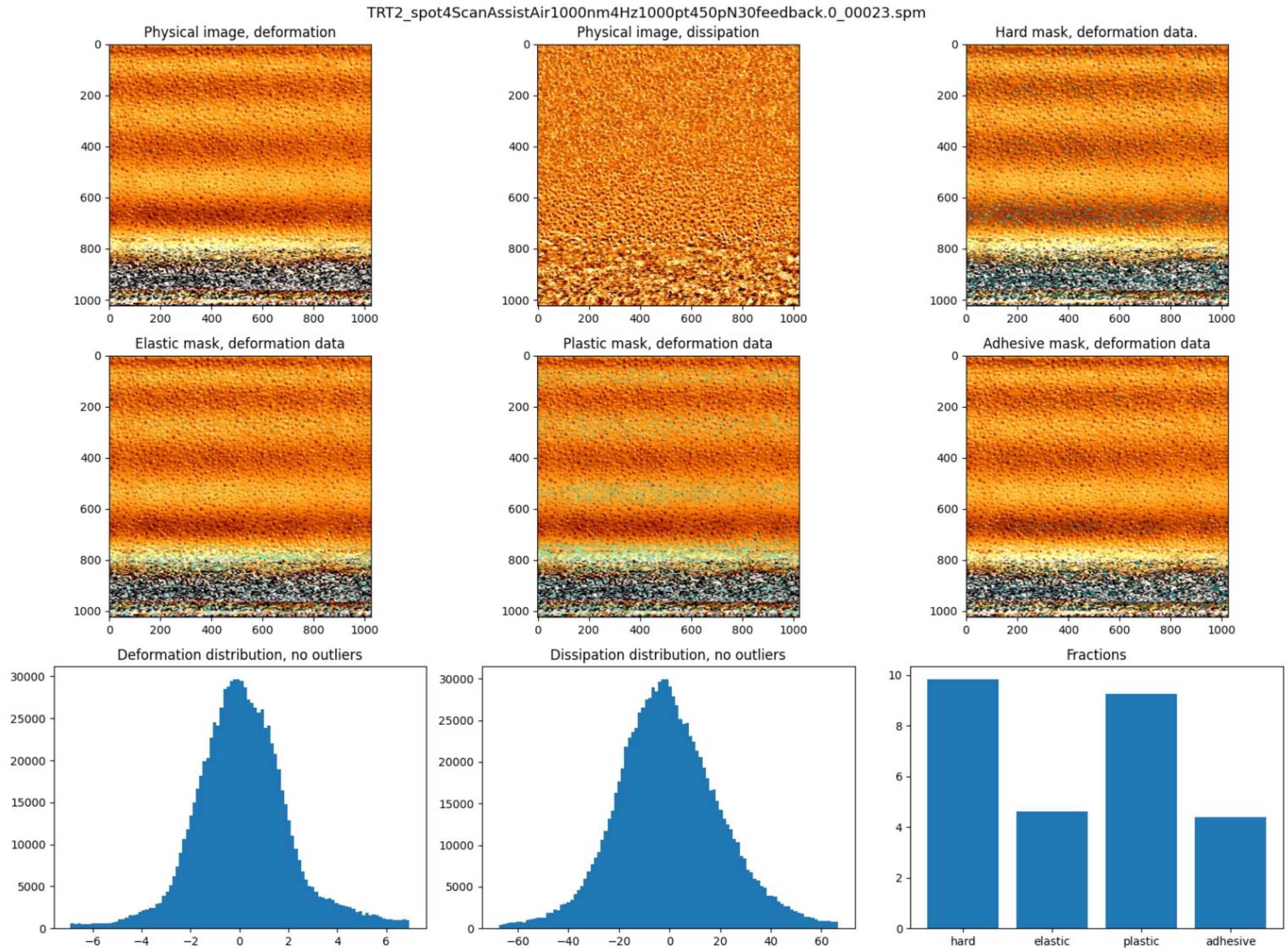

TRT2_spot4ScanAssistAir1000nm4Hz1000pt450pN30feedback.0_00023.spm
Physical image, deformation
Physical image, dissipation
Hard mask, deformation data.
Elastic mask, deformation data
Plastic mask, deformation data
Adhesive mask, deformation data
Deformation distribution, no outliers
Dissipation distribution, no outliers
Fractions
hard
elastic
plastic
adhesive

TRT-P, Recipe 3, type 1

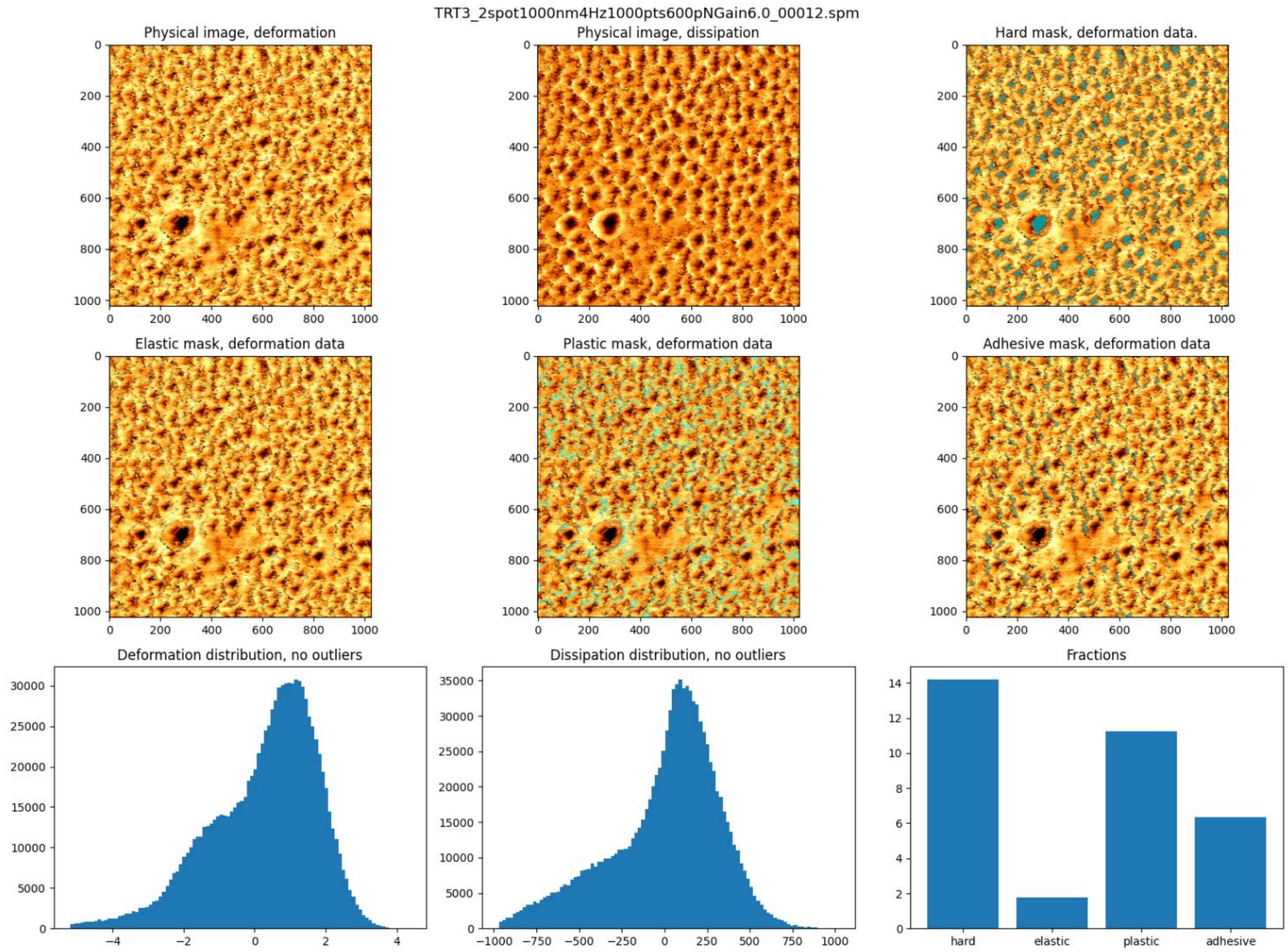

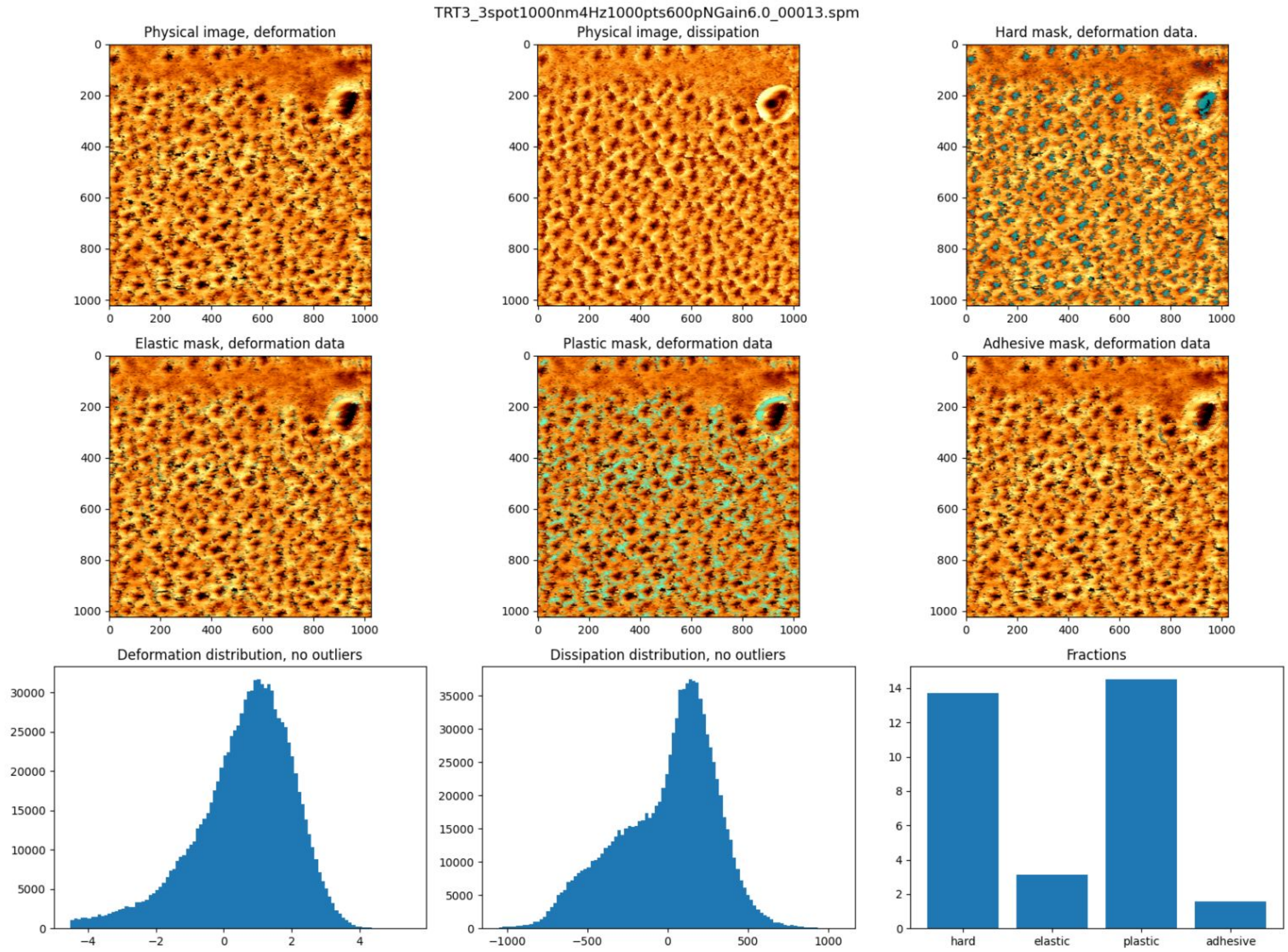

TRT3_3spot1000nm4Hz1000pts600pNGain6.0_00013.spm
Physical image, deformation
Physical image, dissipation
Hard mask, deformation data.
Elastic mask, deformation data
Plastic mask, deformation data
Adhesive mask, deformation data
Deformation distribution, no outliers
Dissipation distribution, no outliers
Fractions
hard
elastic
plastic
adhesive

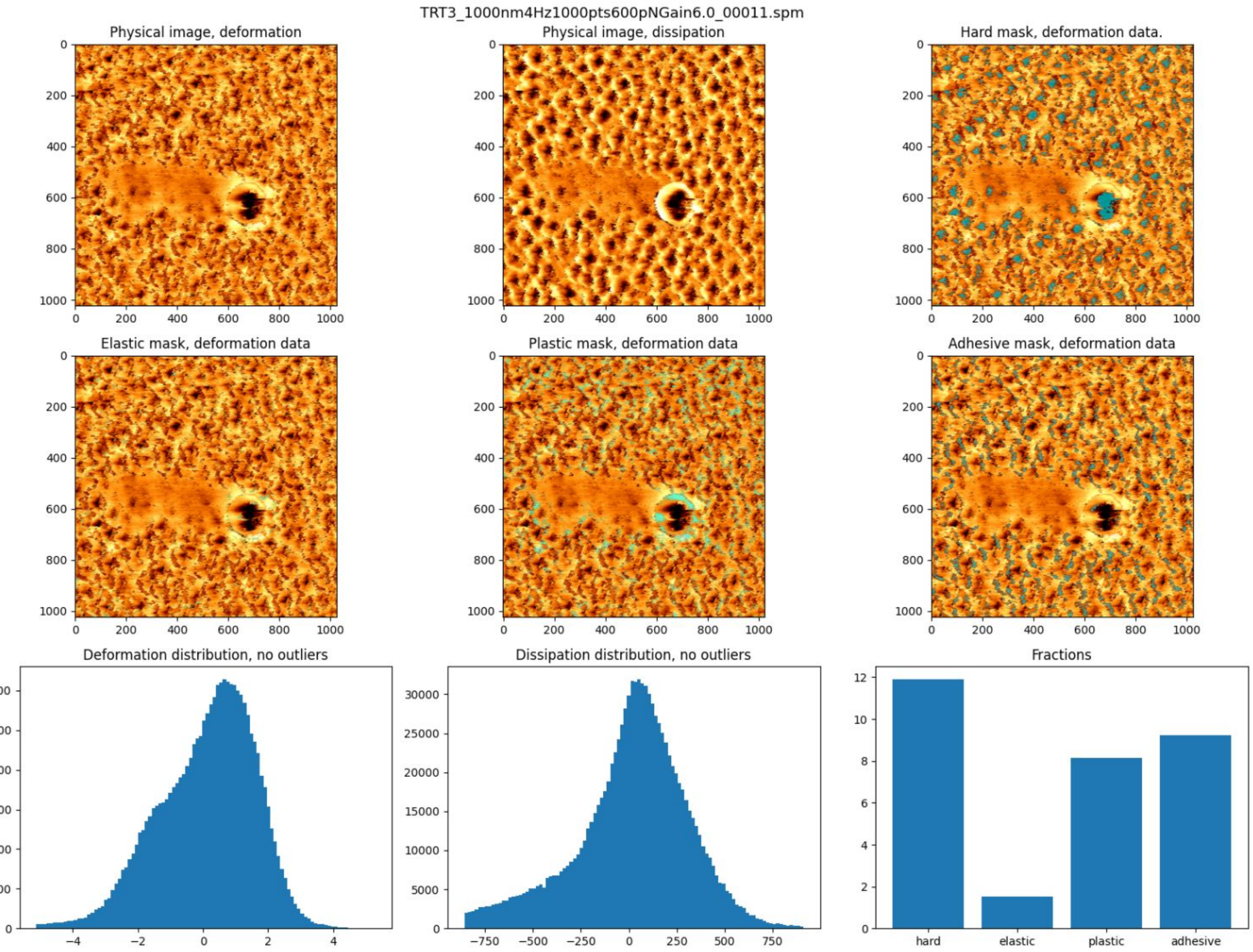
TRT3_1000nm4Hz1000pts600pNGain6.0_00011.spm
Physical image, deformation
Physical image, dissipation
Hard mask, deformation data.
Elastic mask, deformation data
Plastic mask, deformation data
Adhesive mask, deformation data
Deformation distribution, no outliers
Dissipation distribution, no outliers
Fractions
hard
elastic
plastic
adhesive

TRT-P, Recipe 3, type 2

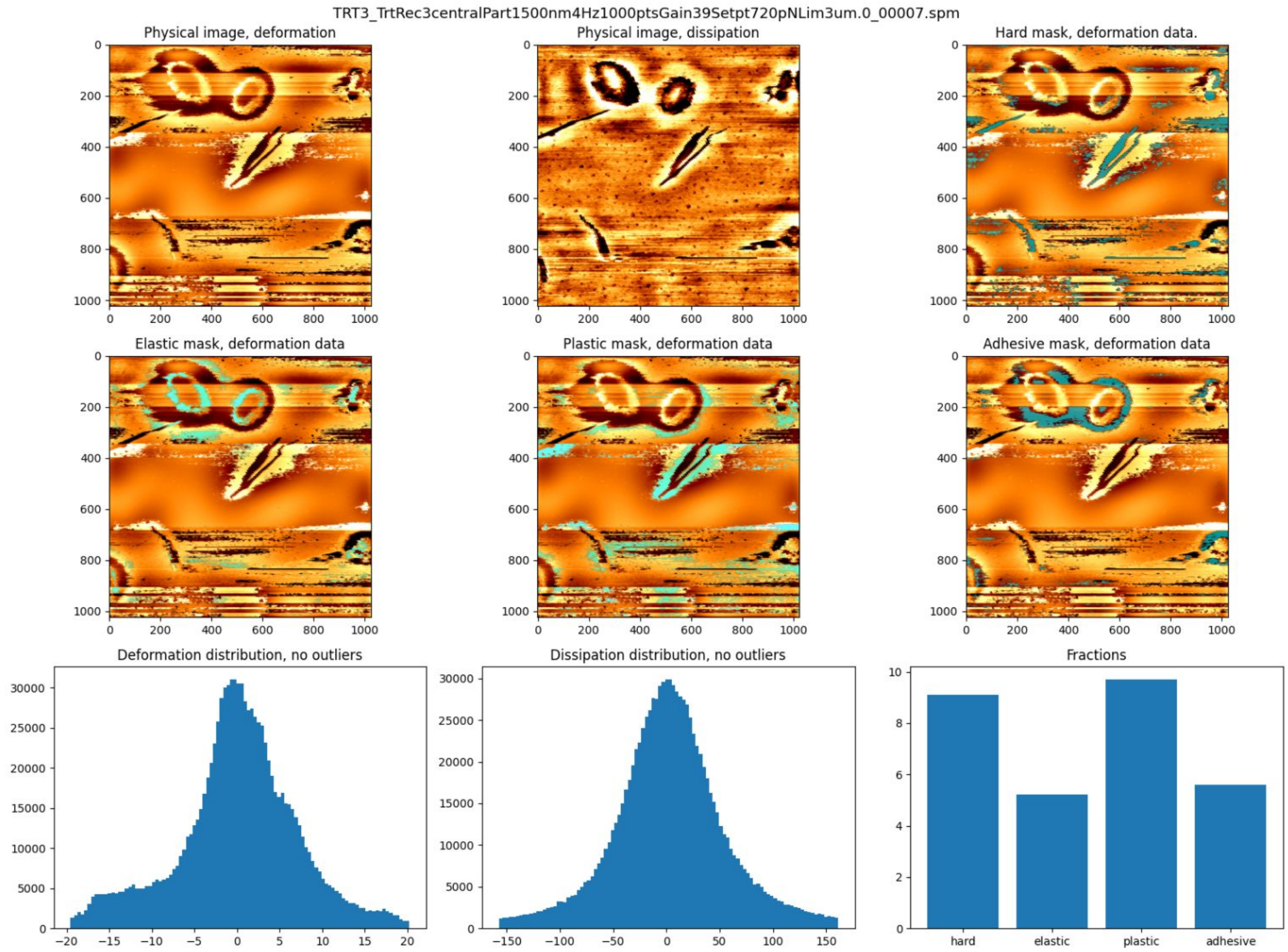

TRT-P, Recipe 4, type 1

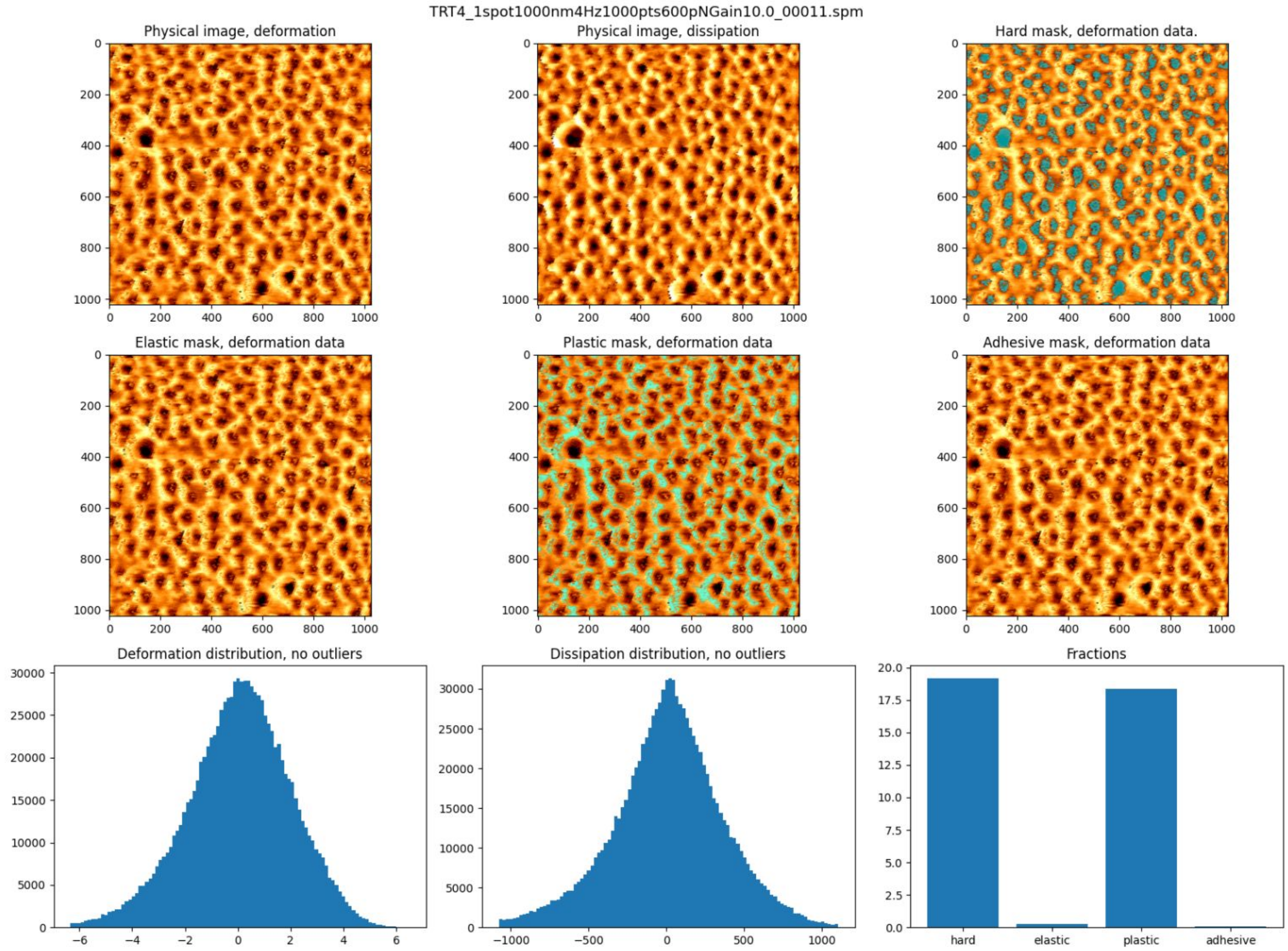

No adhesive fraction. Image looks ok.

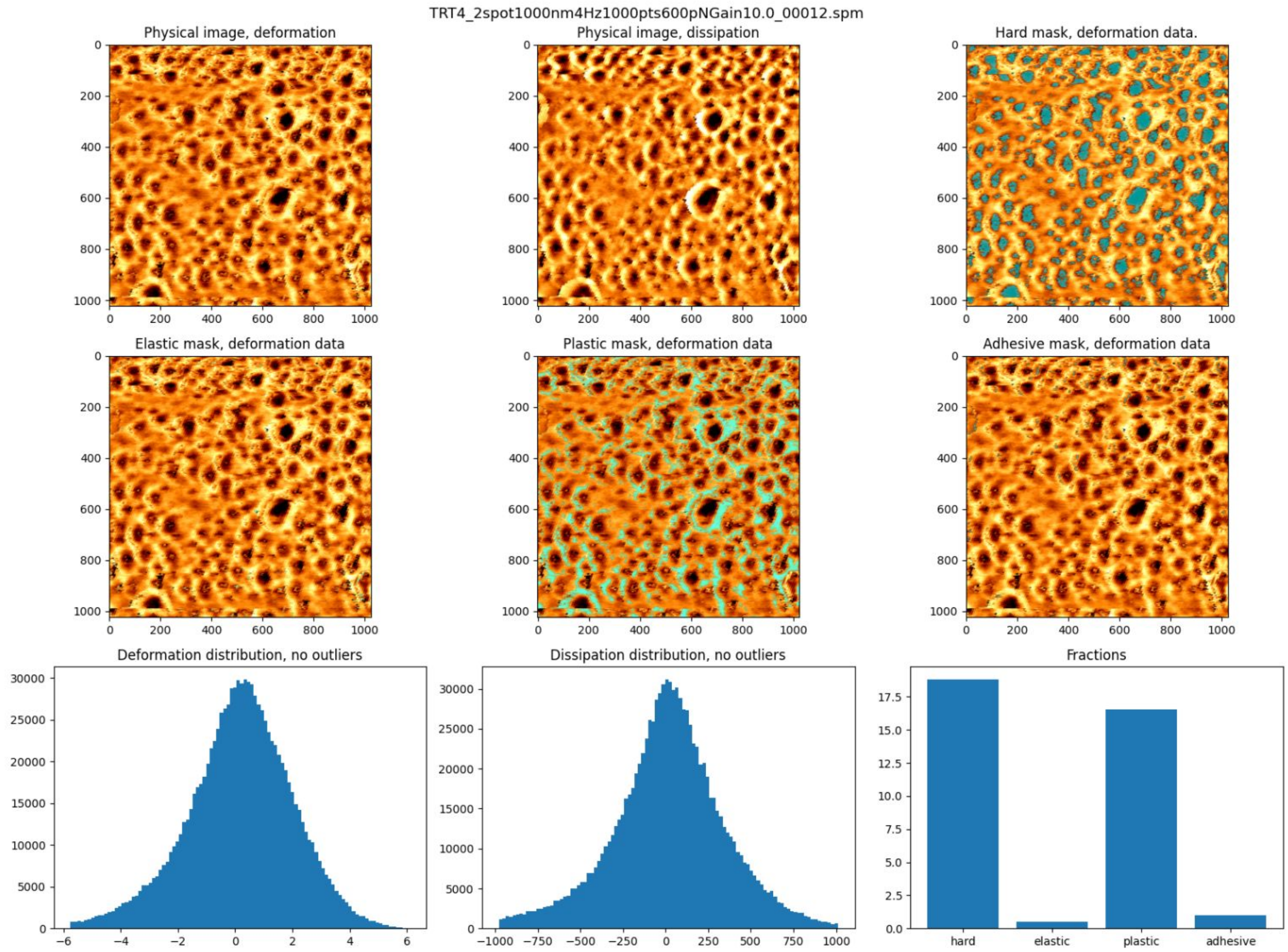
TRT4_2spot1000nm4Hz1000pts600pNGain10.0_00012.spm
Physical image, deformation
Physical image, dissipation
Hard mask, deformation data.
Elastic mask, deformation data
Plastic mask, deformation data
Adhesive mask, deformation data
Deformation distribution, no outliers
Dissipation distribution, no outliers
Fractions
hard
elastic
plastic
adhesive

TRT-P, Recipe 4, type 2

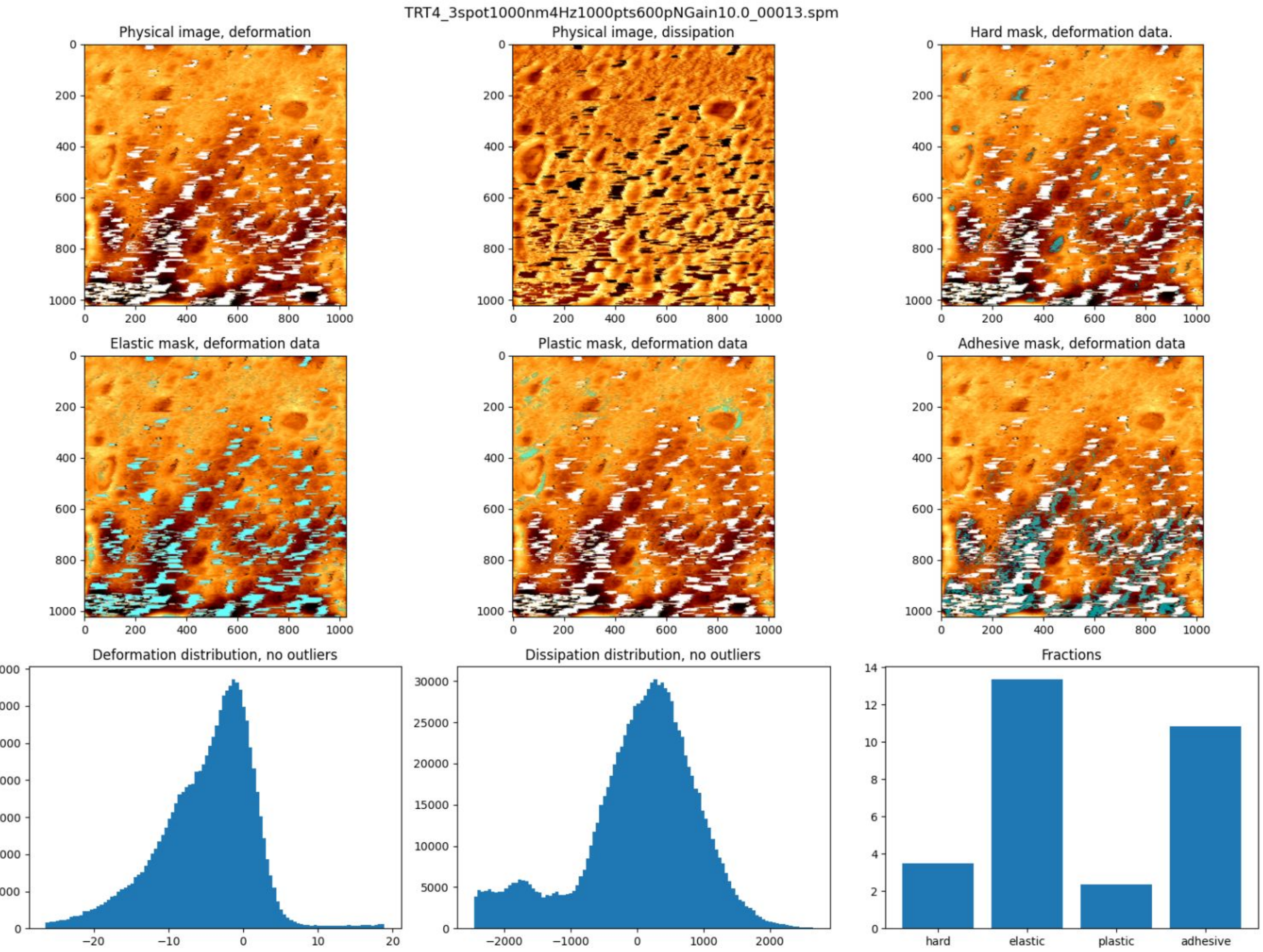

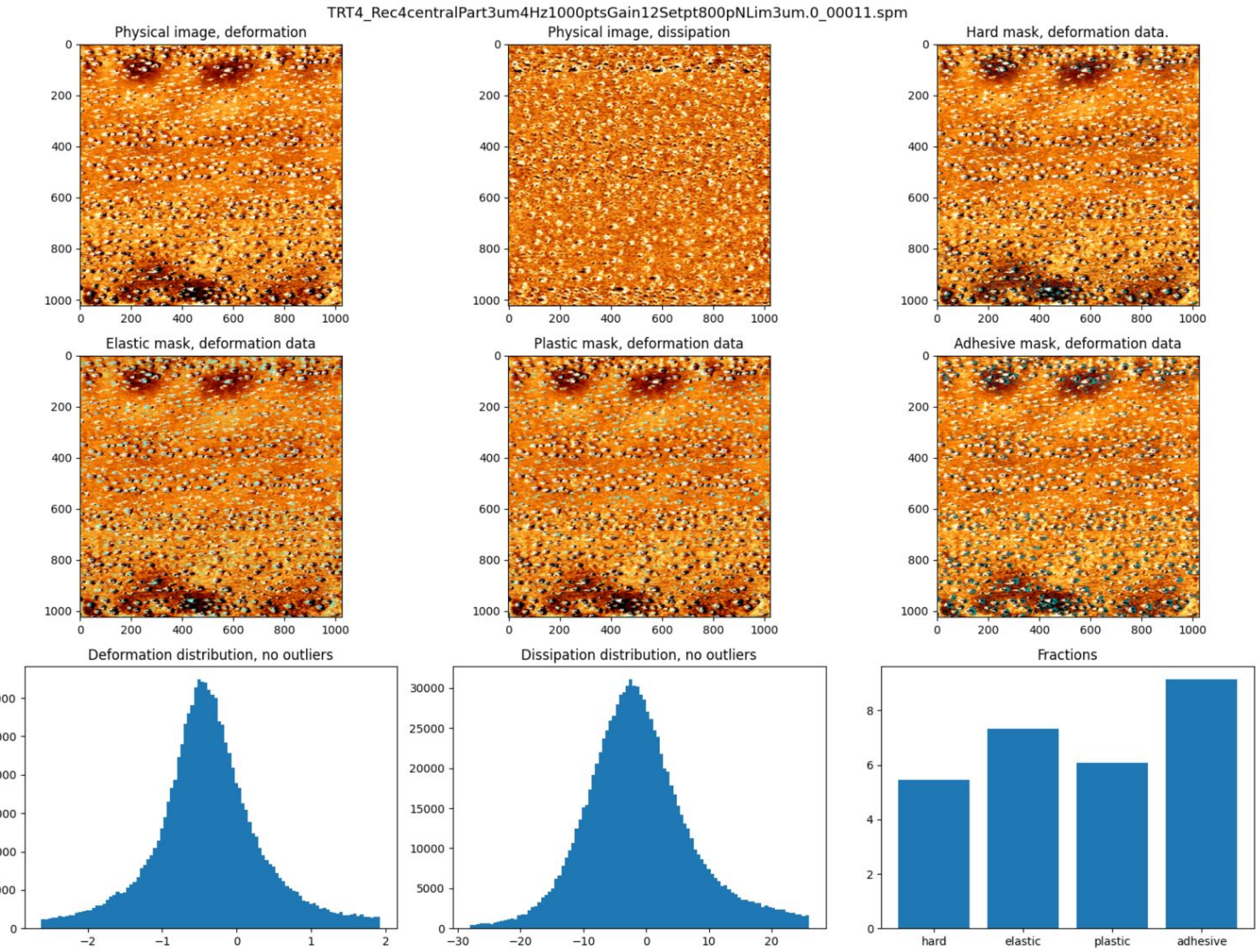

TRT4_Rec4centralPart3um4Hz1000ptsGain12Setpt800pNLim3um.0_00011.spm
Physical image, deformation
Physical image, dissipation
Hard mask, deformation data.
Elastic mask, deformation data
Plastic mask, deformation data
Adhesive mask, deformation data
Deformation distribution, no outliers
Dissipation distribution, no outliers
Fractions
hard
elastic
plastic
adhesive

TRT-P, Recipe 5

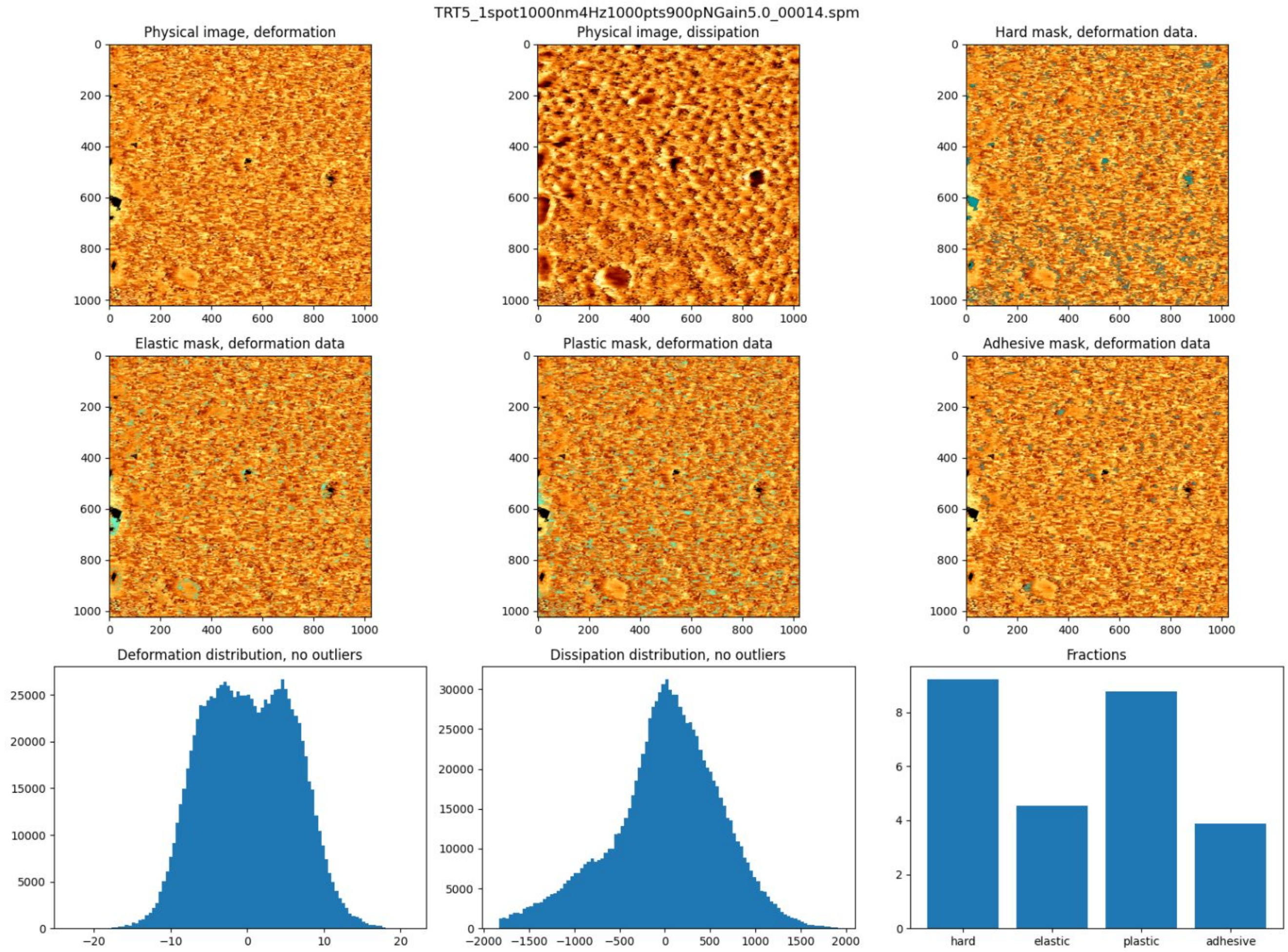

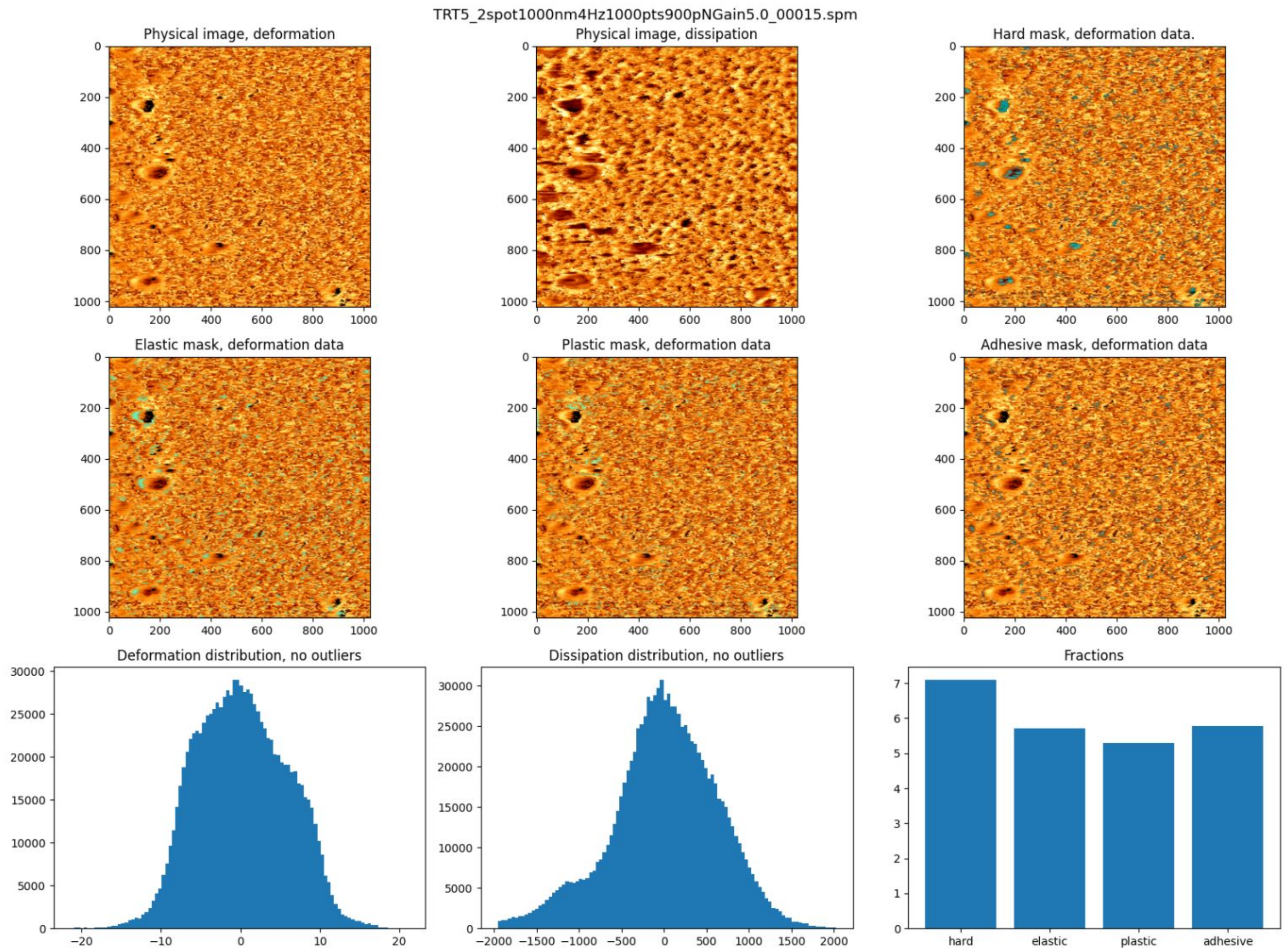
TRT5_2spot1000nm4Hz1000pts900pNGain5.0_00015.spm
Physical image, deformation
Physical image, dissipation
Hard mask, deformation data.
Elastic mask, deformation data
Plastic mask, deformation data
Adhesive mask, deformation data
Deformation distribution, no outliers
Dissipation distribution, no outliers
Fractions
hard
elastic
plastic
adhesive

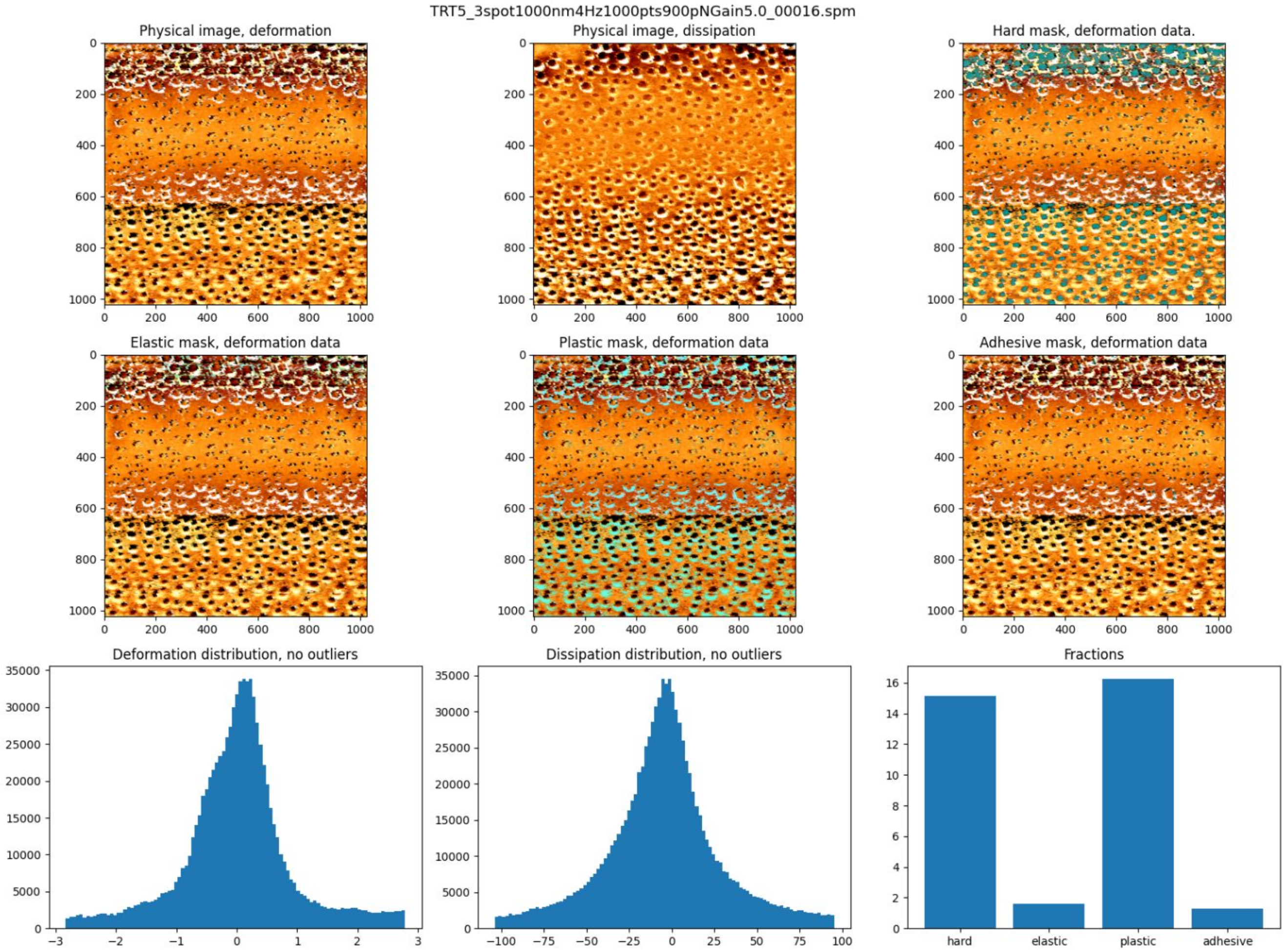
TRT5_3spot1000nm4Hz1000pts900pNGain5.0_00016.spm
Physical image, deformation
Physical image, dissipation
Hard mask, deformation data.
Elastic mask, deformation data
Plastic mask, deformation data
Adhesive mask, deformation data
Deformation distribution, no outliers
Dissipation distribution, no outliers
Fractions
hard
elastic
plastic
adhesive

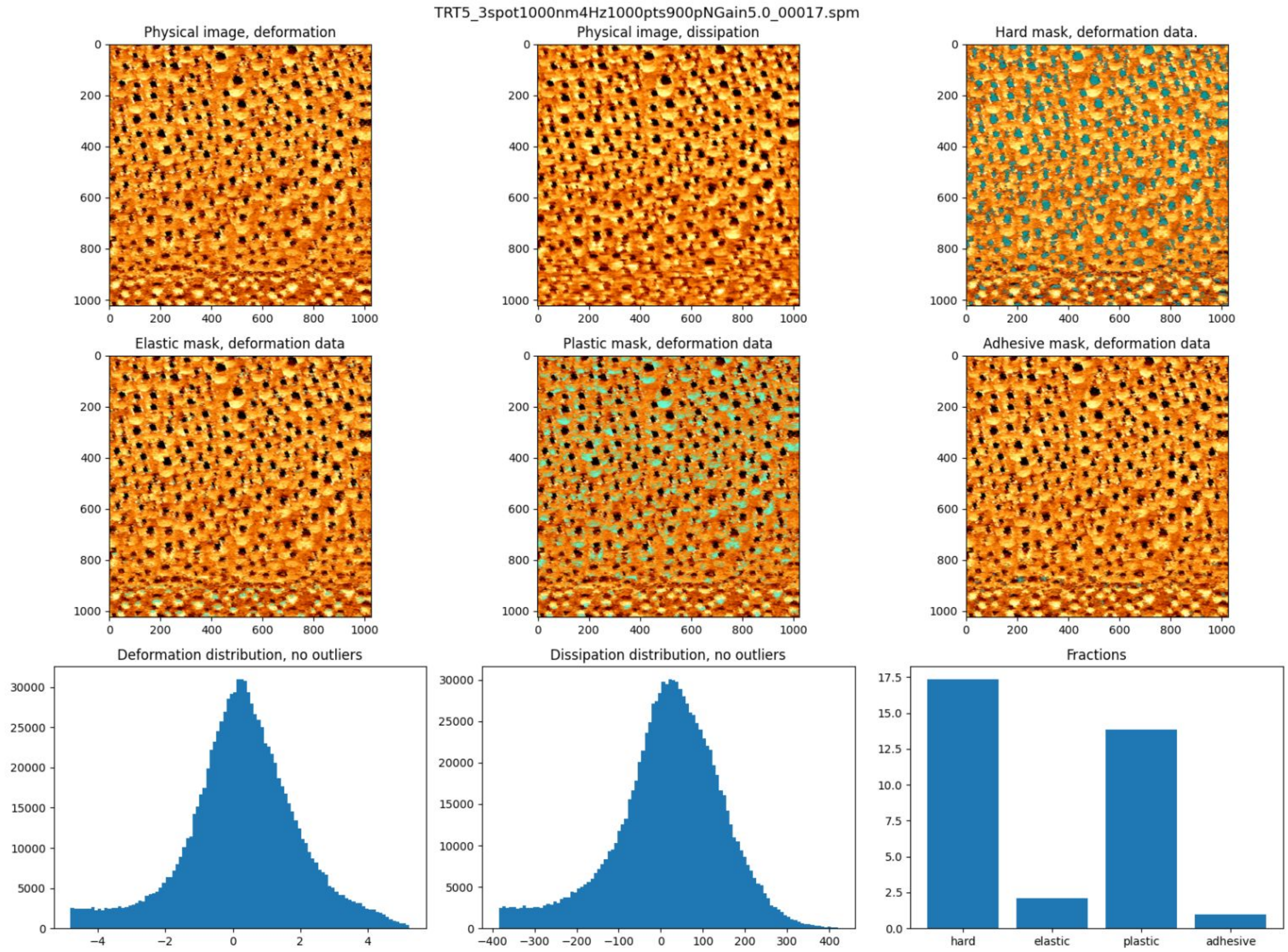
TRT5_3spot1000nm4Hz1000pts900pNGain5.0_00017.spm
Physical image, deformation
Physical image, dissipation
Hard mask, deformation data.
Elastic mask, deformation data
Plastic mask, deformation data
Adhesive mask, deformation data
Deformation distribution, no outliers
Dissipation distribution, no outliers
Fractions
hard
elastic
plastic
adhesive

TRT-P, Recipe 6

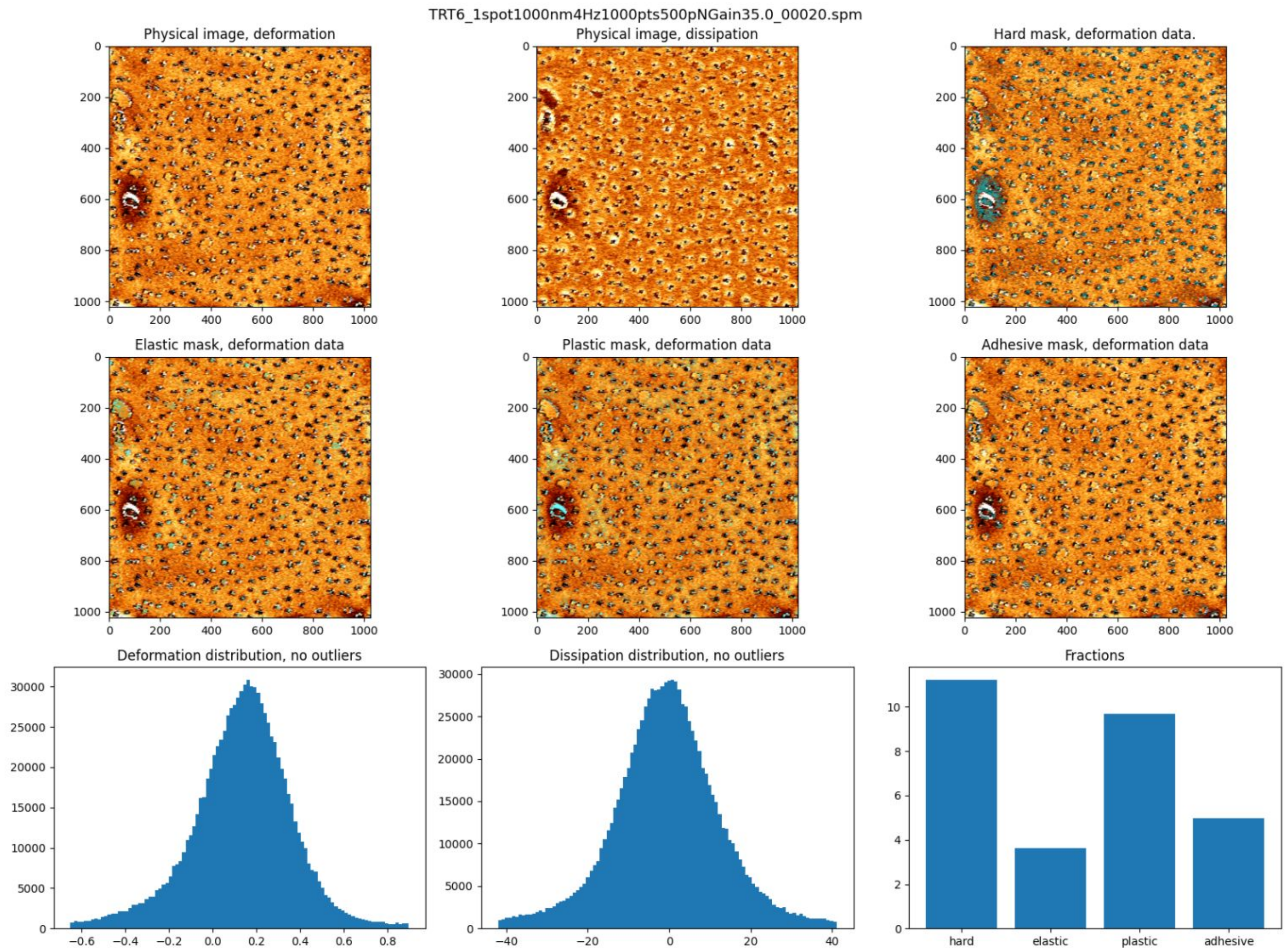

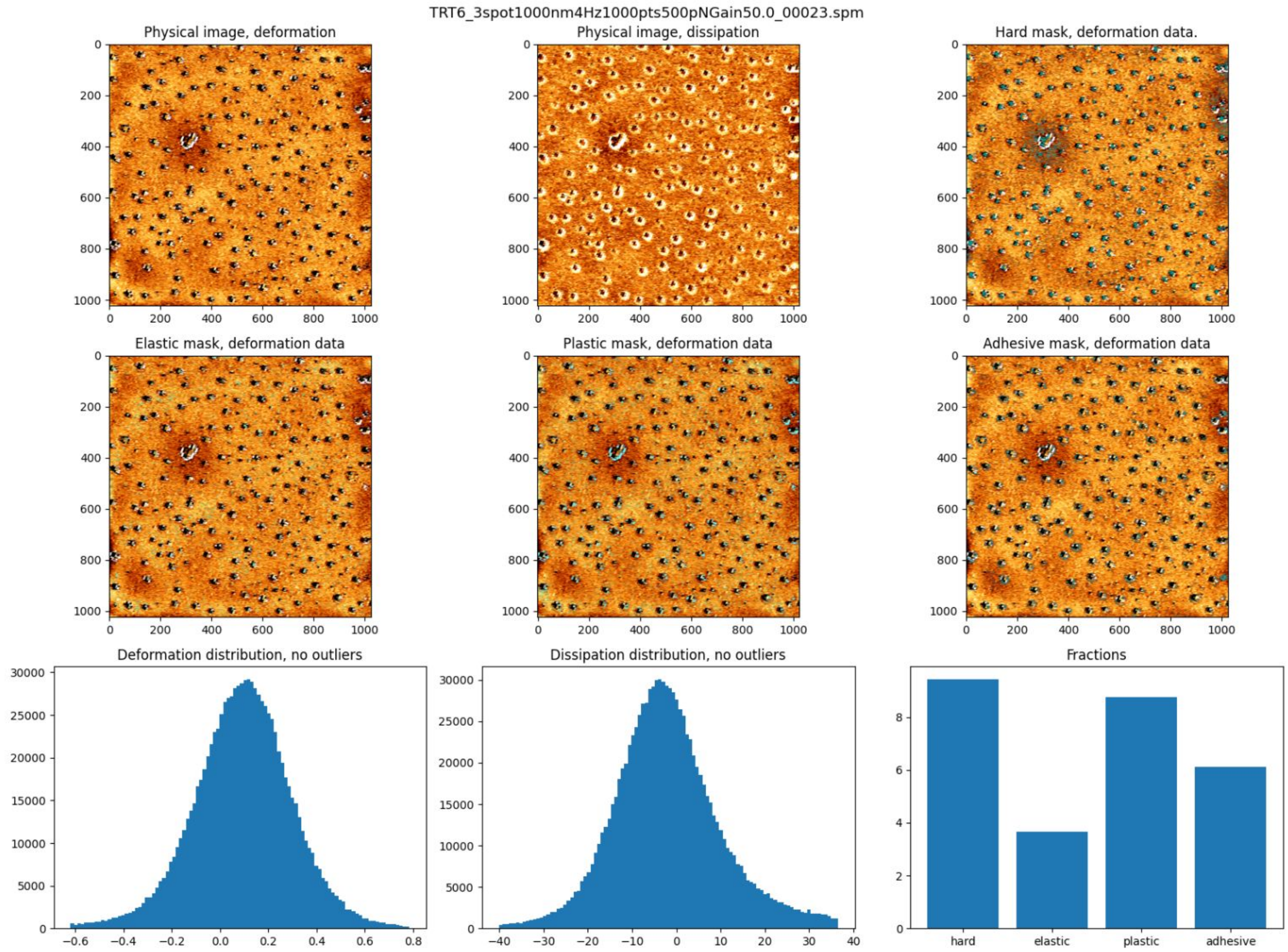
TRT6_3spot1000nm4Hz1000pts500pNGain50.0_00023.spm
Physical image, deformation
Physical image, dissipation
Hard mask, deformation data.
Elastic mask, deformation data
Plastic mask, deformation data
Adhesive mask, deformation data
Deformation distribution, no outliers
Dissipation distribution, no outliers
Fractions
hard
elastic
plastic
adhesive

TRT-M, Recipe 1, type 1

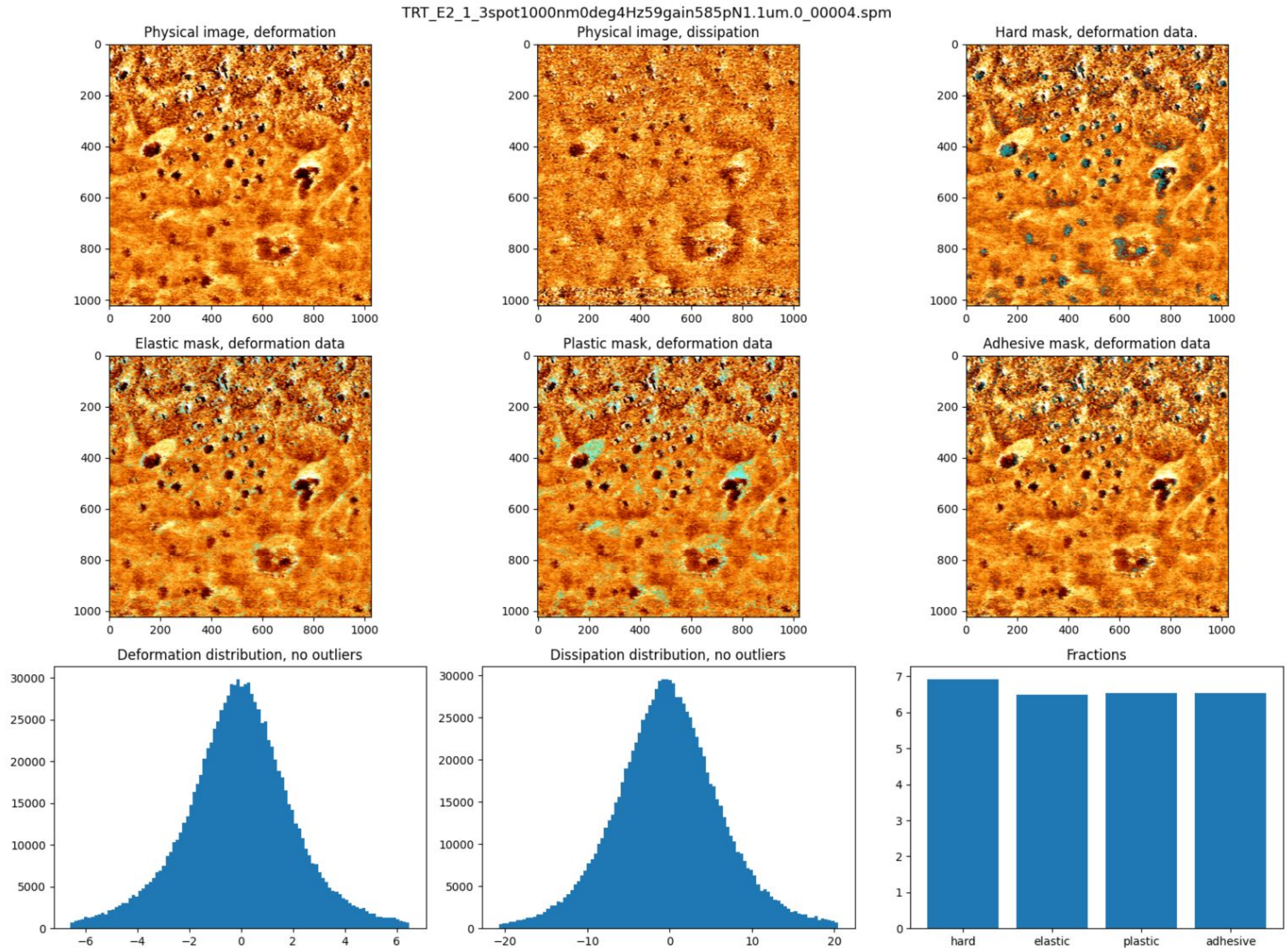

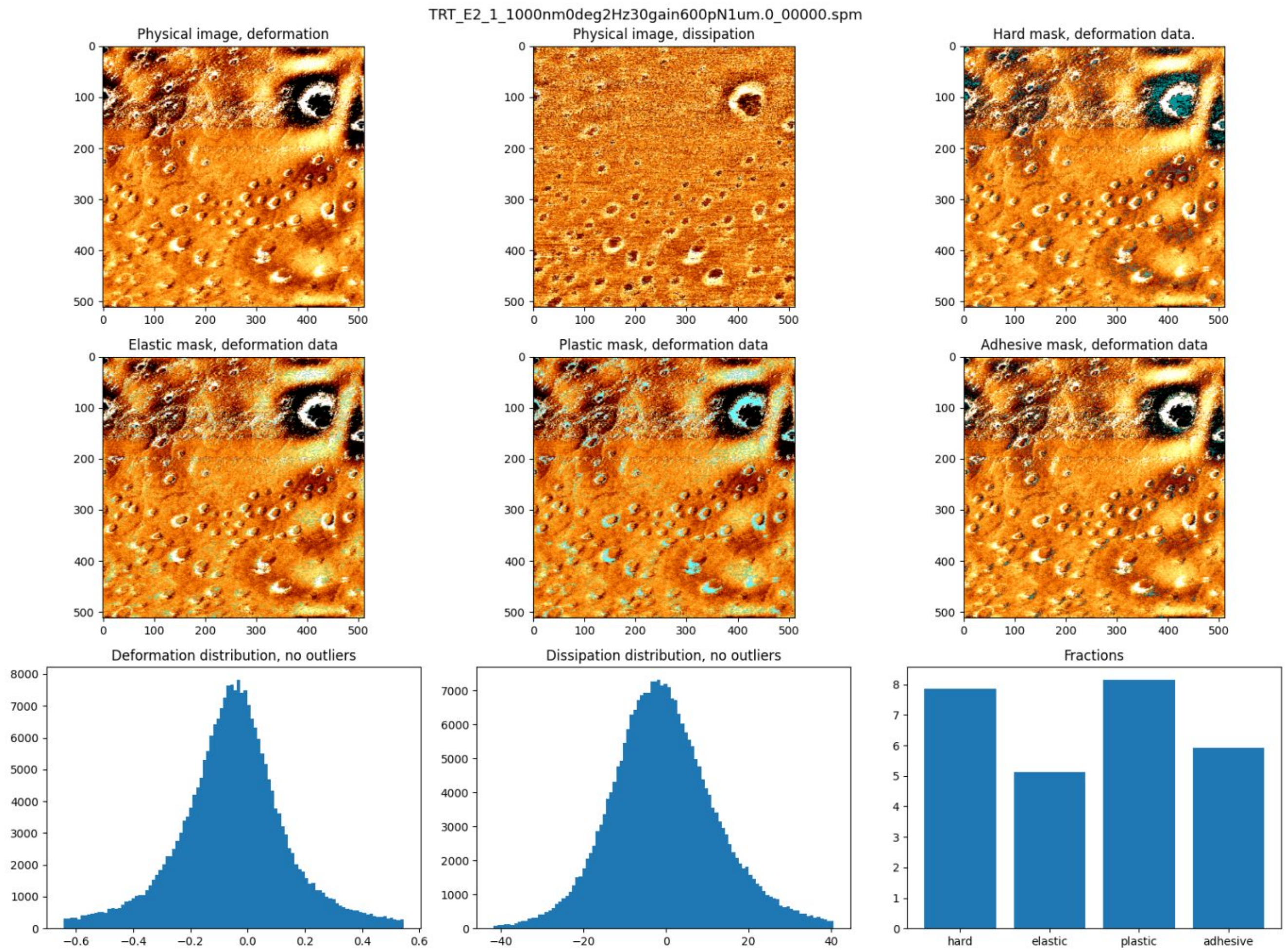
TRT_E2_1_1000nm0deg2Hz30gain600pN1um.0_00000.spm
Physical image, deformation
Physical image, dissipation
Hard mask, deformation data.
Elastic mask, deformation data
Plastic mask, deformation data
Adhesive mask, deformation data
Deformation distribution, no outliers
Dissipation distribution, no outliers
Fractions
hard
elastic
plastic
adhesive

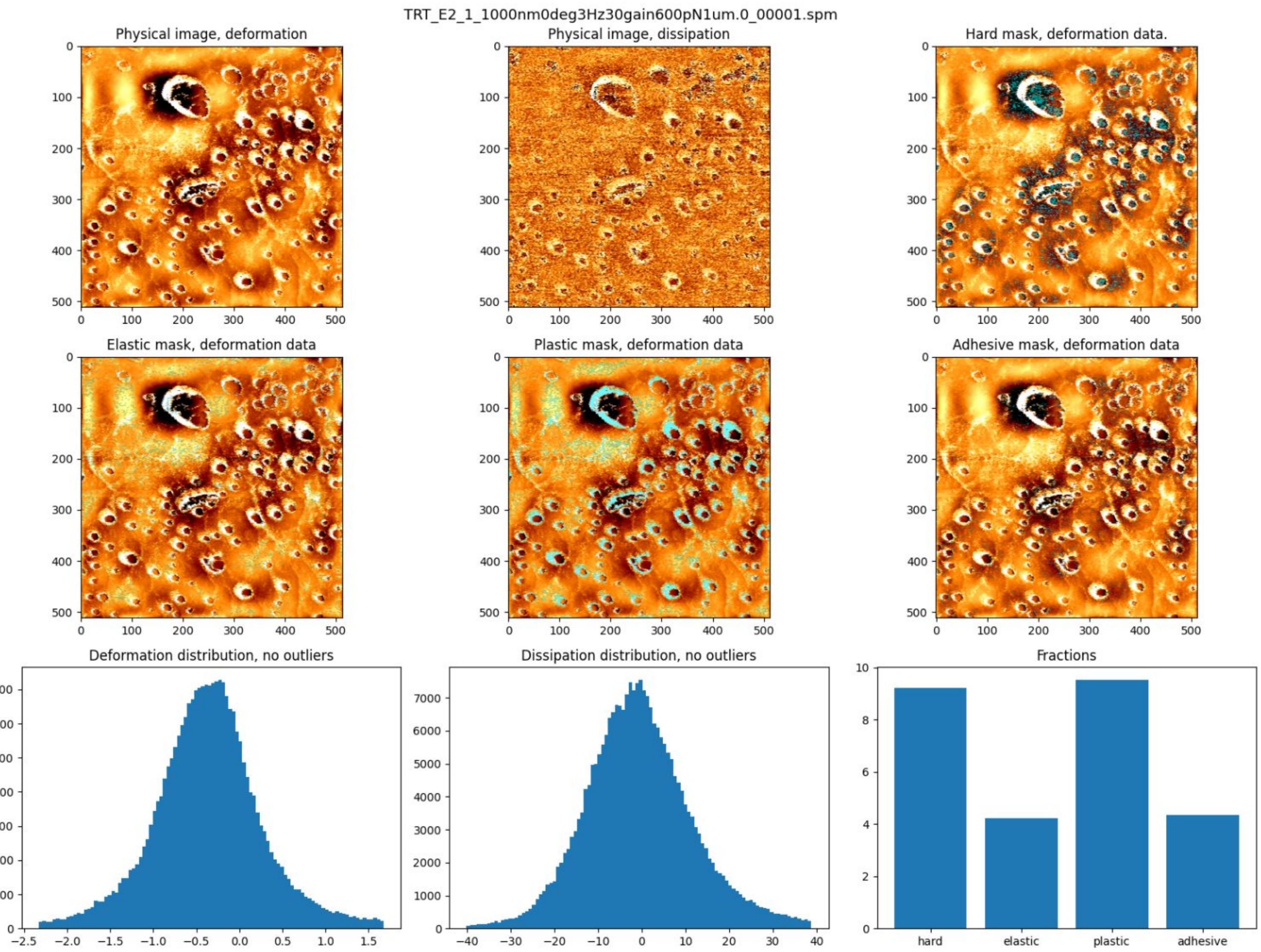
TRT_E2_1_1000nm0deg3Hz30gain600pN1um.0_00001.spm
Physical image, deformation
Physical image, dissipation
Hard mask, deformation data.
Elastic mask, deformation data
Plastic mask, deformation data
Adhesive mask, deformation data
Deformation distribution, no outliers
Dissipation distribution, no outliers
Fractions
hard
elastic
plastic
adhesive

TRT-M, Recipe 1, type 2

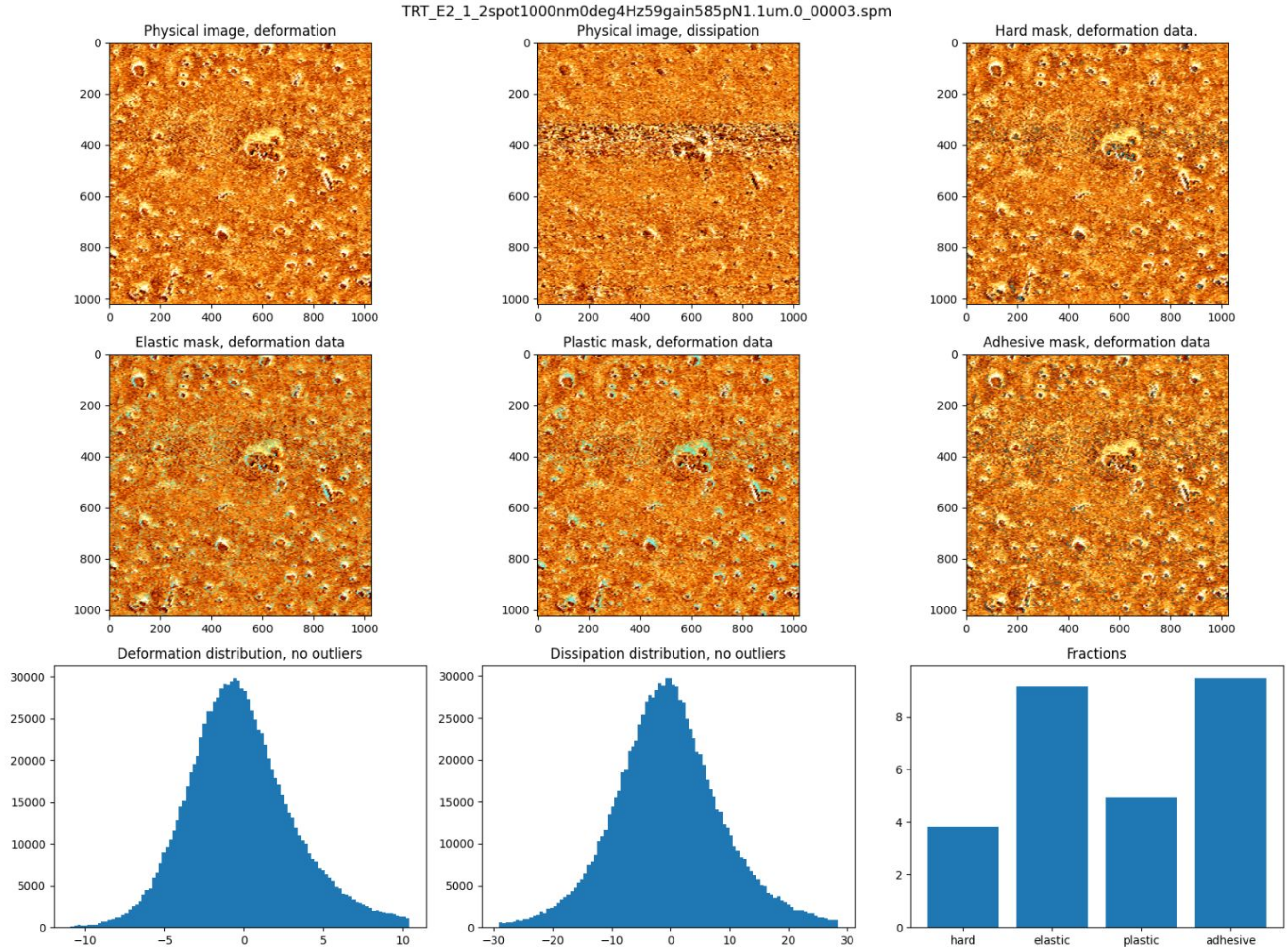

TRT-M, Recipe 2

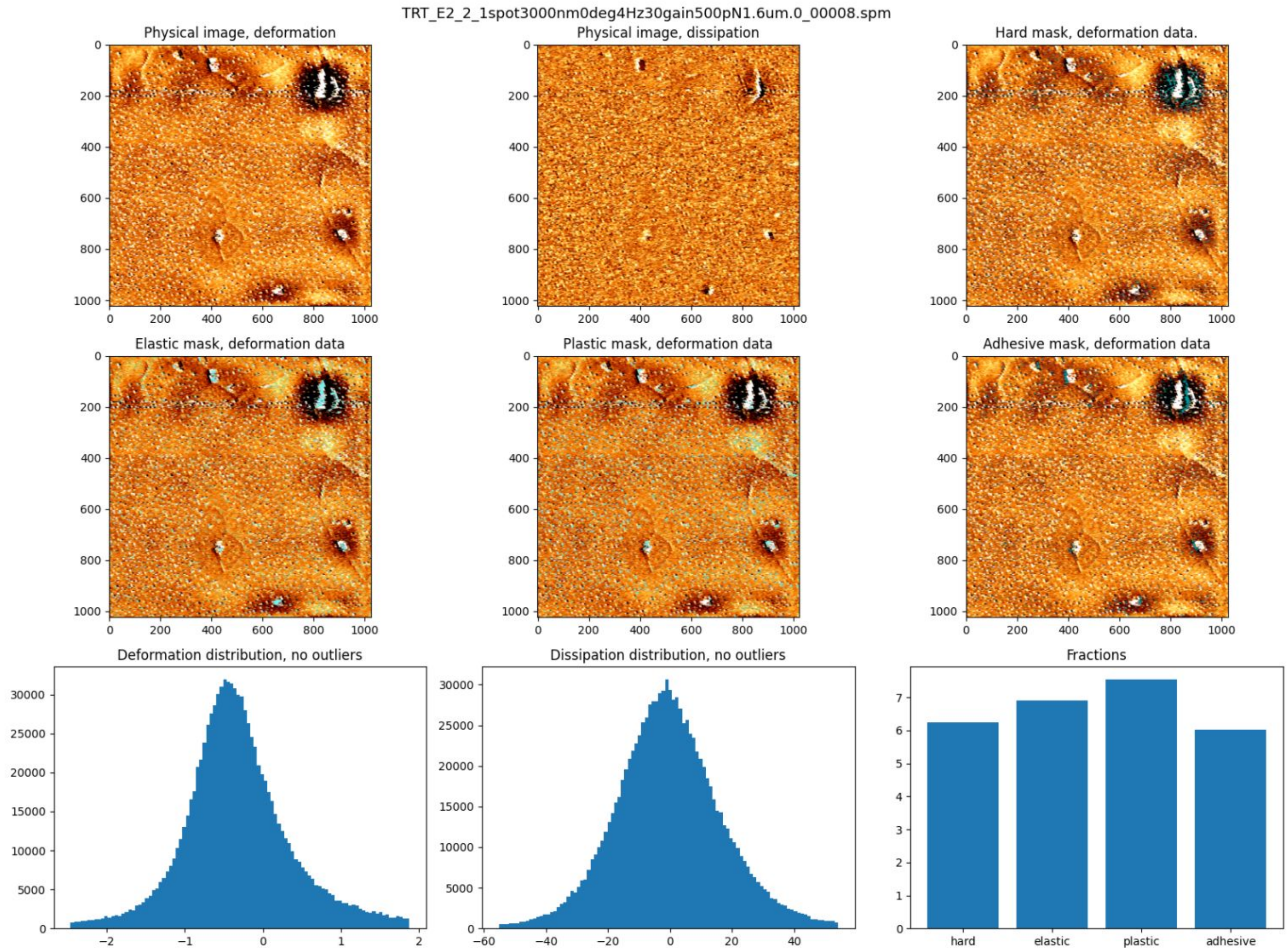

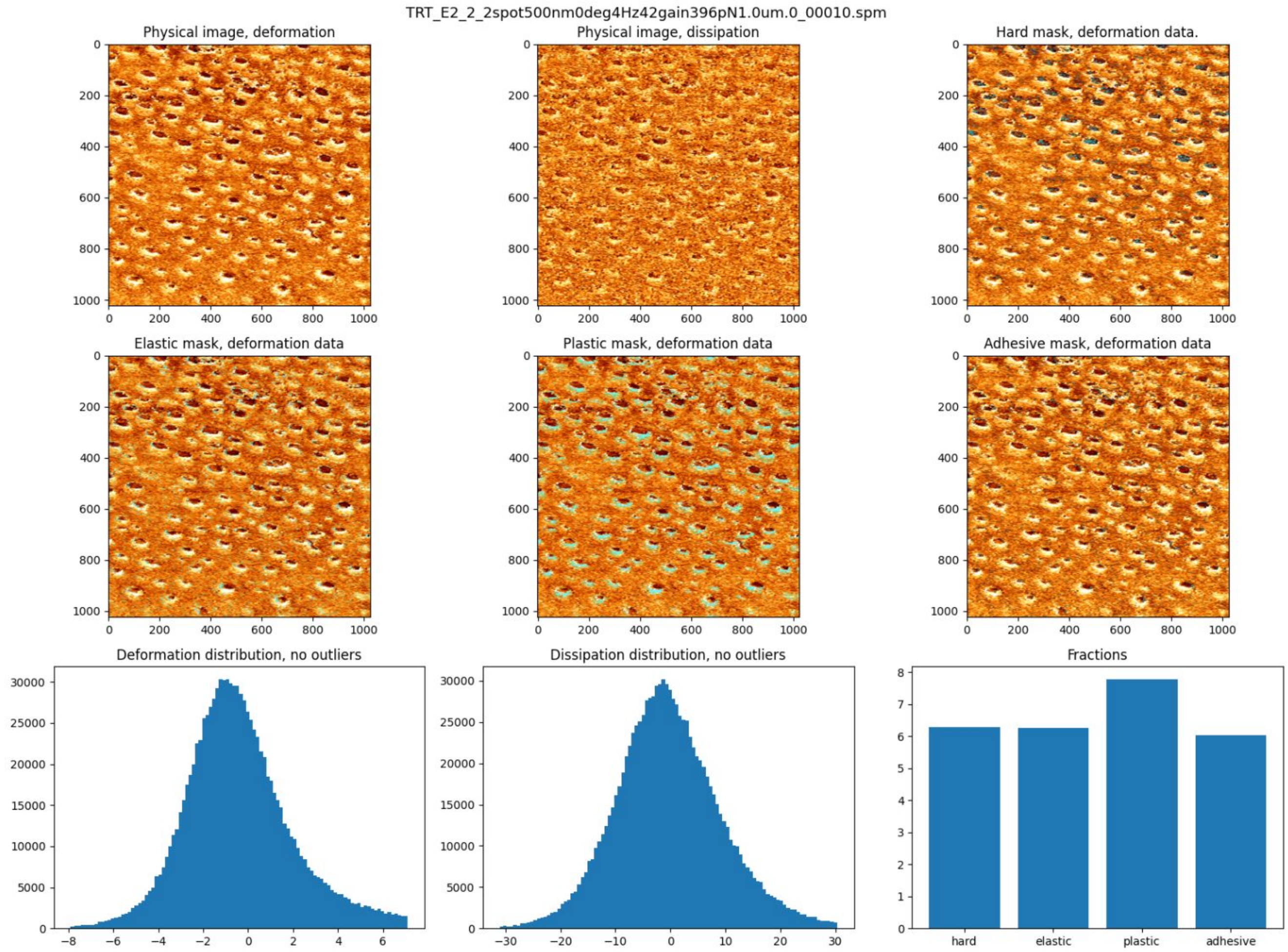
TRT_E2_2_2spot500nm0deg4Hz42gain396pN1.0um.0_00010.spm
Physical image, deformation
Physical image, dissipation
Hard mask, deformation data.
Elastic mask, deformation data
Plastic mask, deformation data
Adhesive mask, deformation data
Deformation distribution, no outliers
Dissipation distribution, no outliers
Fractions
hard
elastic
plastic
adhesive
0
200
400
600
800
1000
5000
10000
15000
20000
25000
30000
−8
−6
−4
−2
2
4
6
−30
−20
−10
10
20
30
1
2
3
4
5
6
7
8

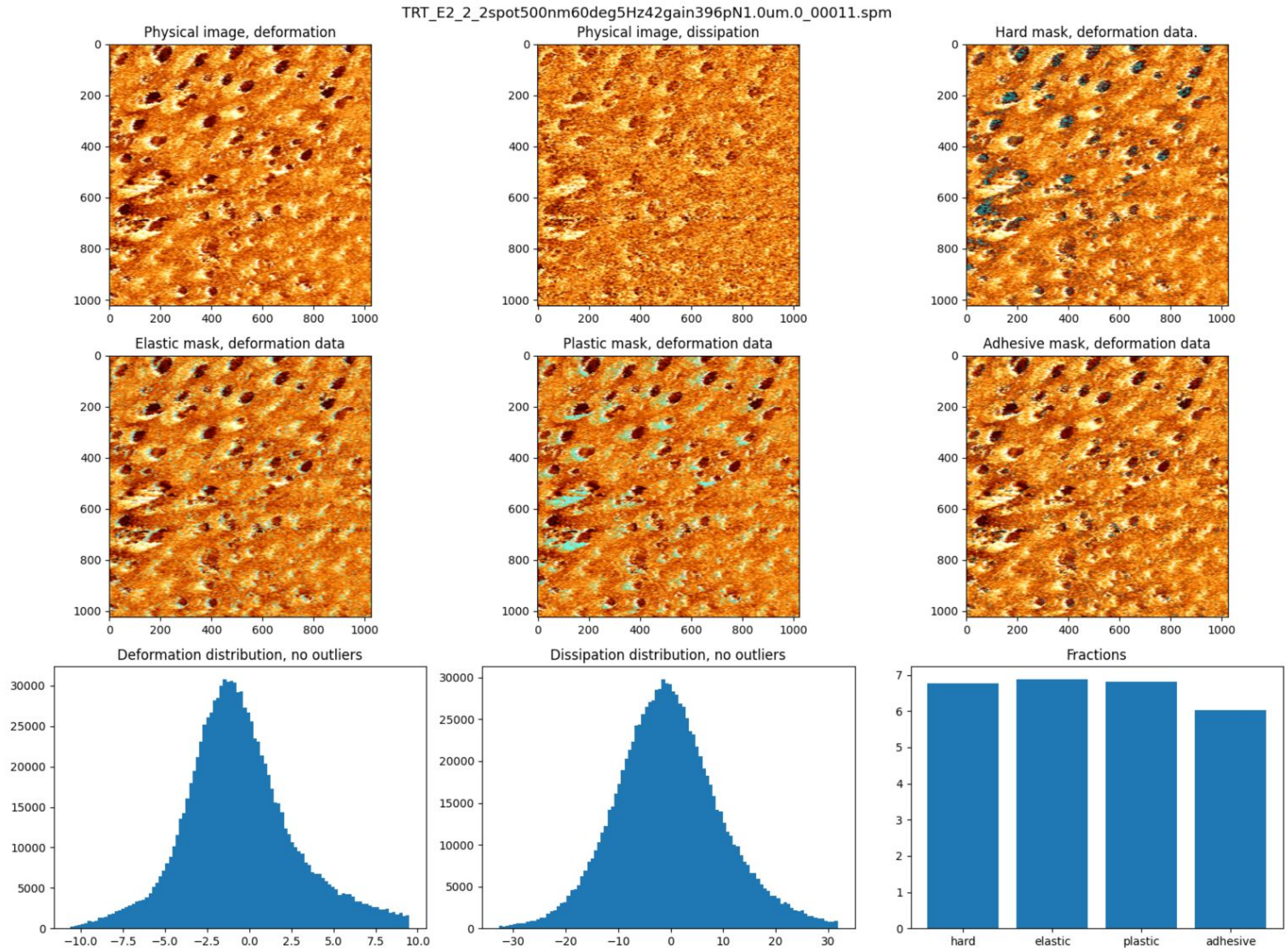
TRT_E2_2_2spot500nm60deg5Hz42gain396pN1.0um.0_00011.spm
Physical image, deformation
Physical image, dissipation
Hard mask, deformation data.
Elastic mask, deformation data
Plastic mask, deformation data
Adhesive mask, deformation data
Deformation distribution, no outliers
Dissipation distribution, no outliers
Fractions
hard
elastic
plastic
adhesive

TRT-M, Recipe 3, type 1

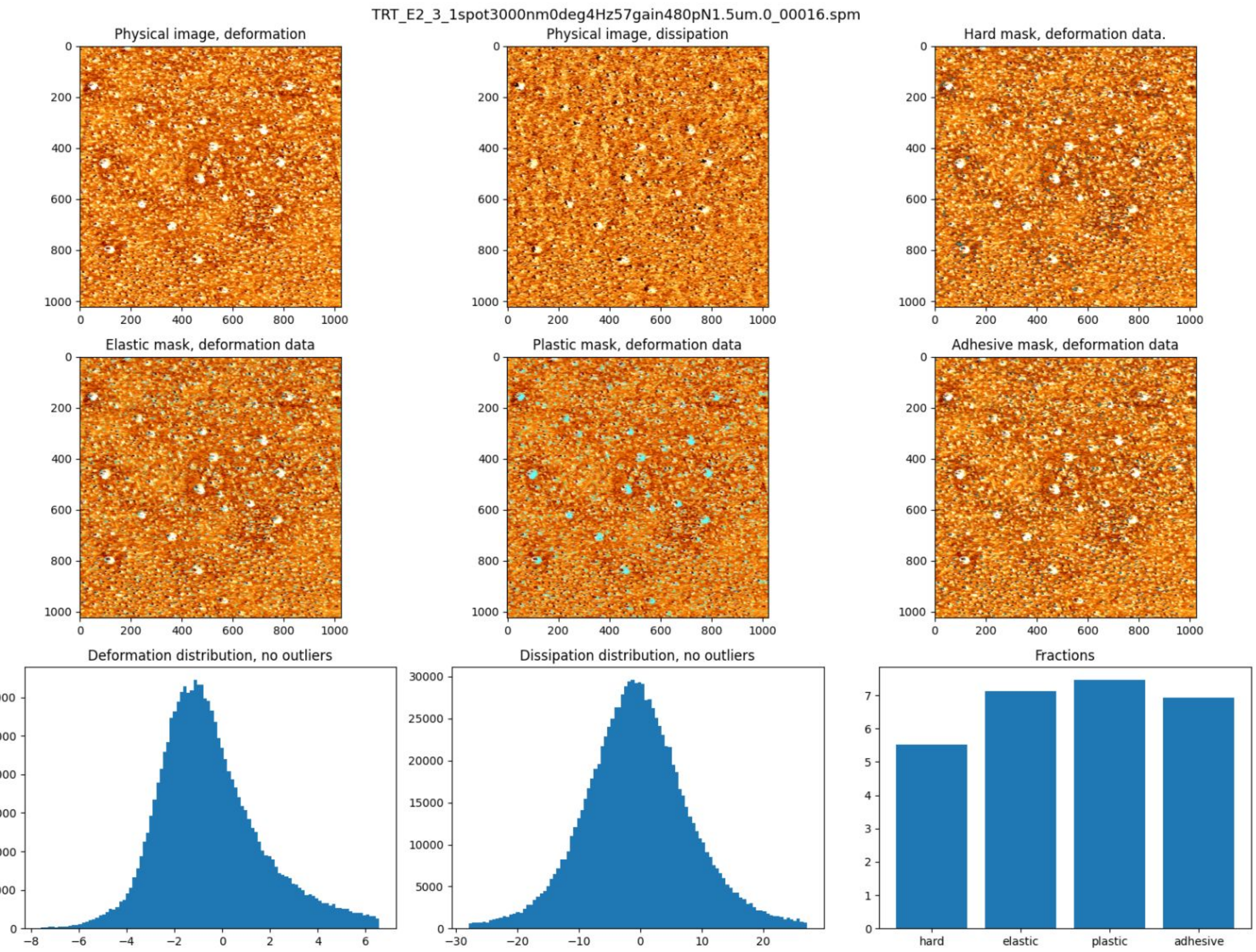

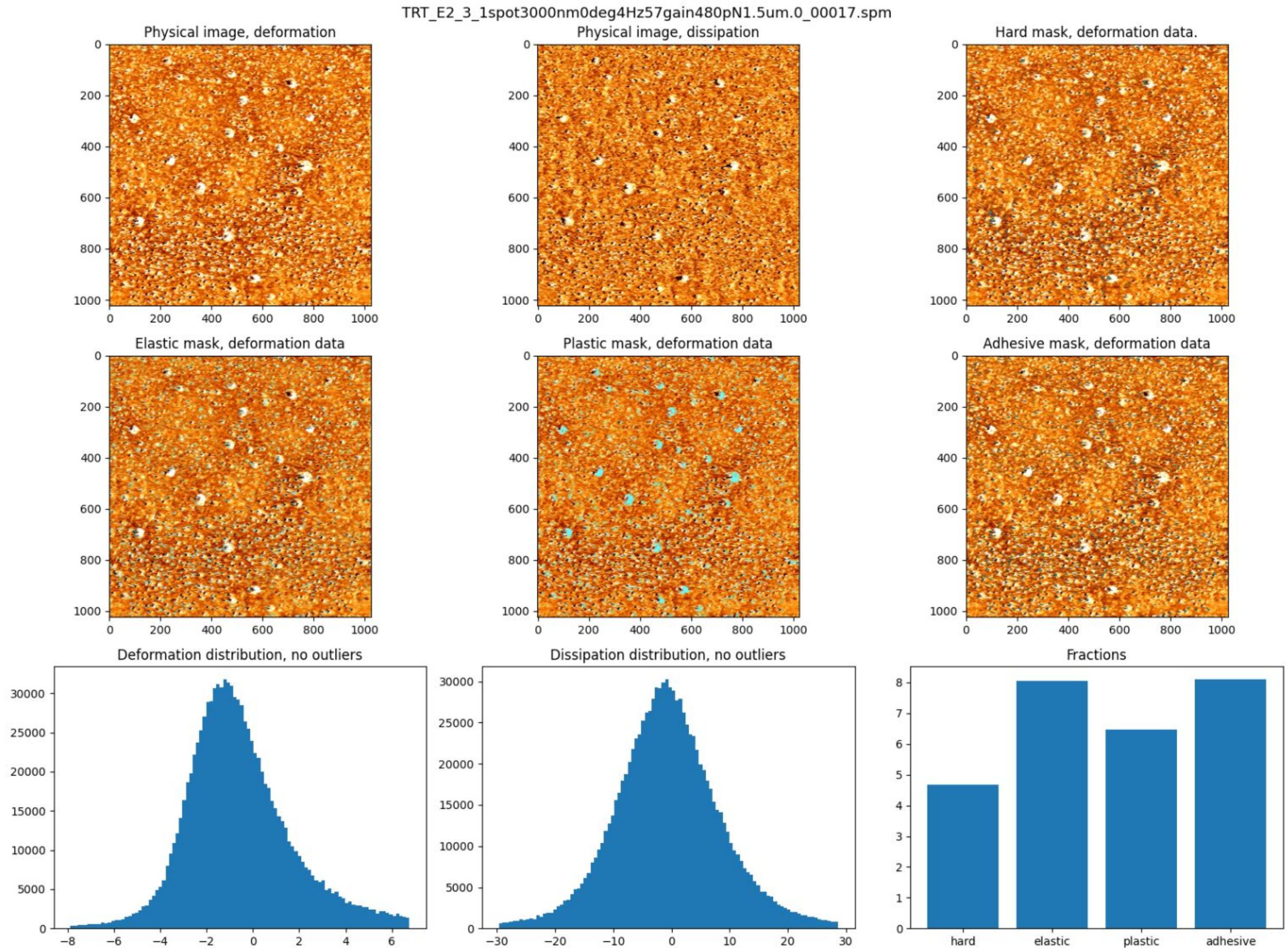
TRT_E2_3_1spot3000nm0deg4Hz57gain480pN1.5um.0_00017.spm
Physical image, deformation
Physical image, dissipation
Hard mask, deformation data.
Elastic mask, deformation data
Plastic mask, deformation data
Adhesive mask, deformation data
Deformation distribution, no outliers
Dissipation distribution, no outliers
Fractions
hard
elastic
plastic
adhesive

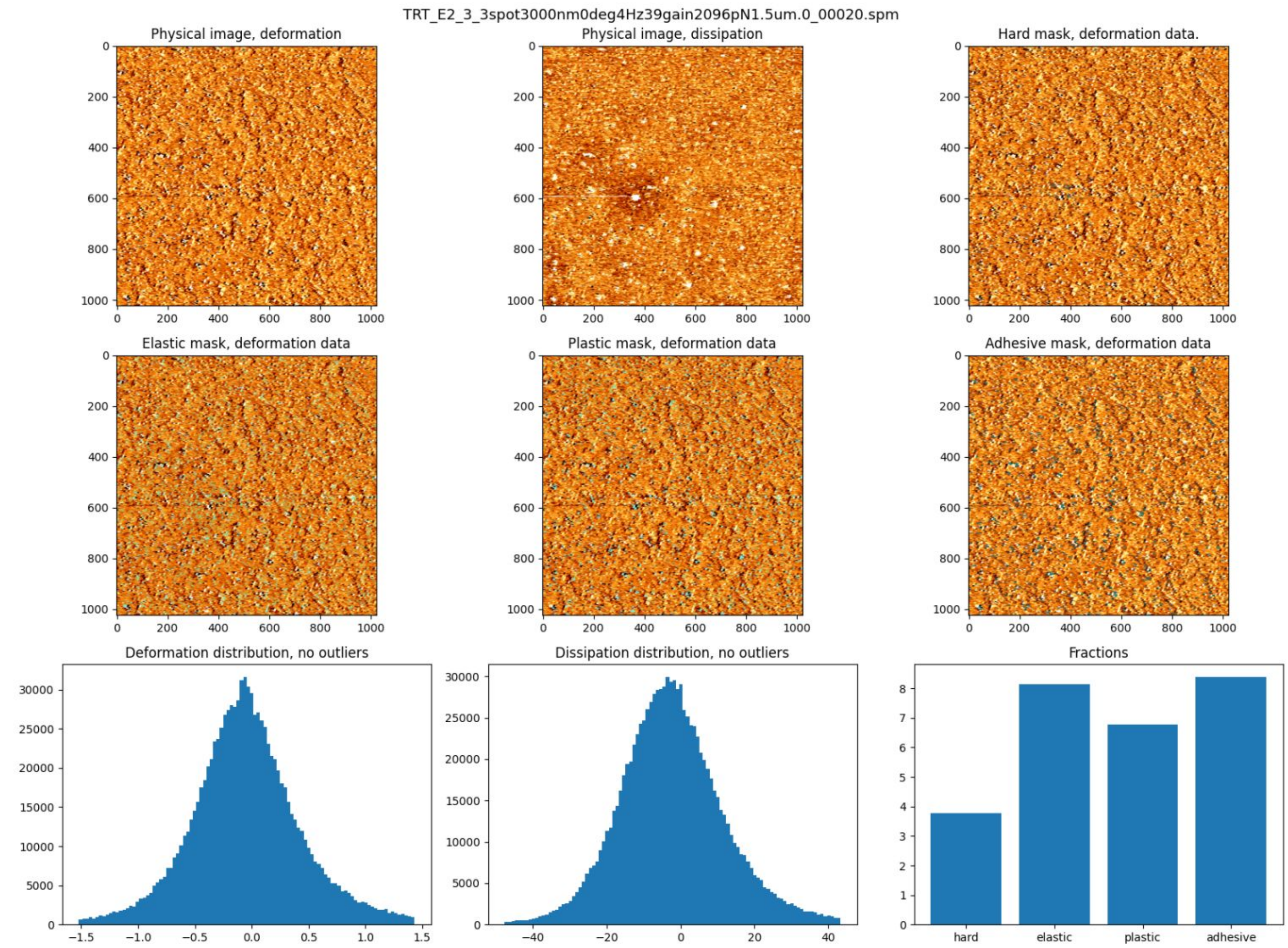
TRT_E2_3_3spot3000nm0deg4Hz39gain2096pN1.5um.0_00020.spm
Physical image, deformation
Physical image, dissipation
Hard mask, deformation data.
Elastic mask, deformation data
Plastic mask, deformation data
Adhesive mask, deformation data
Deformation distribution, no outliers
Dissipation distribution, no outliers
Fractions
hard
elastic
plastic
adhesive

TRT-M, Recipe 3, type 2

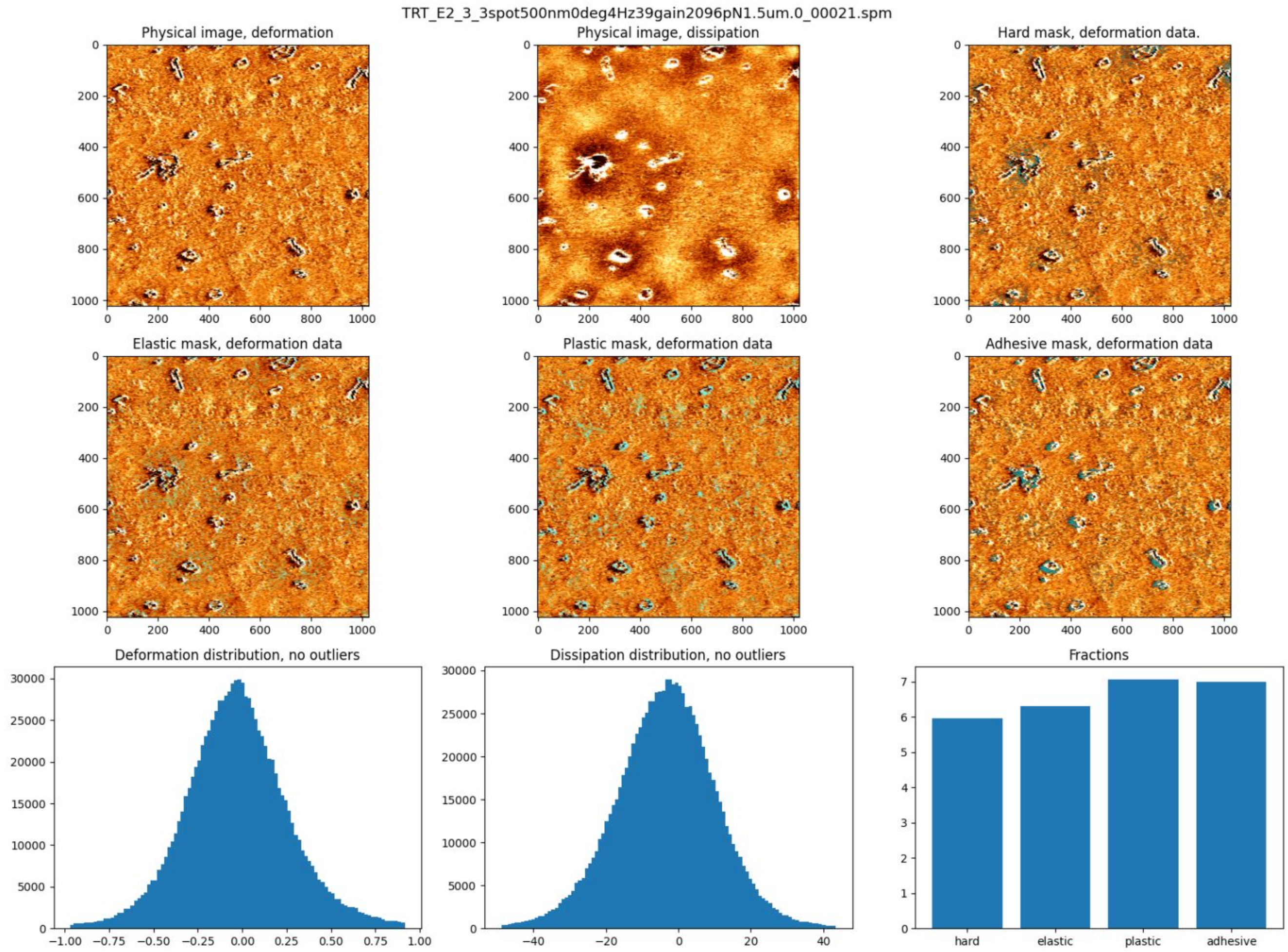

TRT-M, Recipe 4

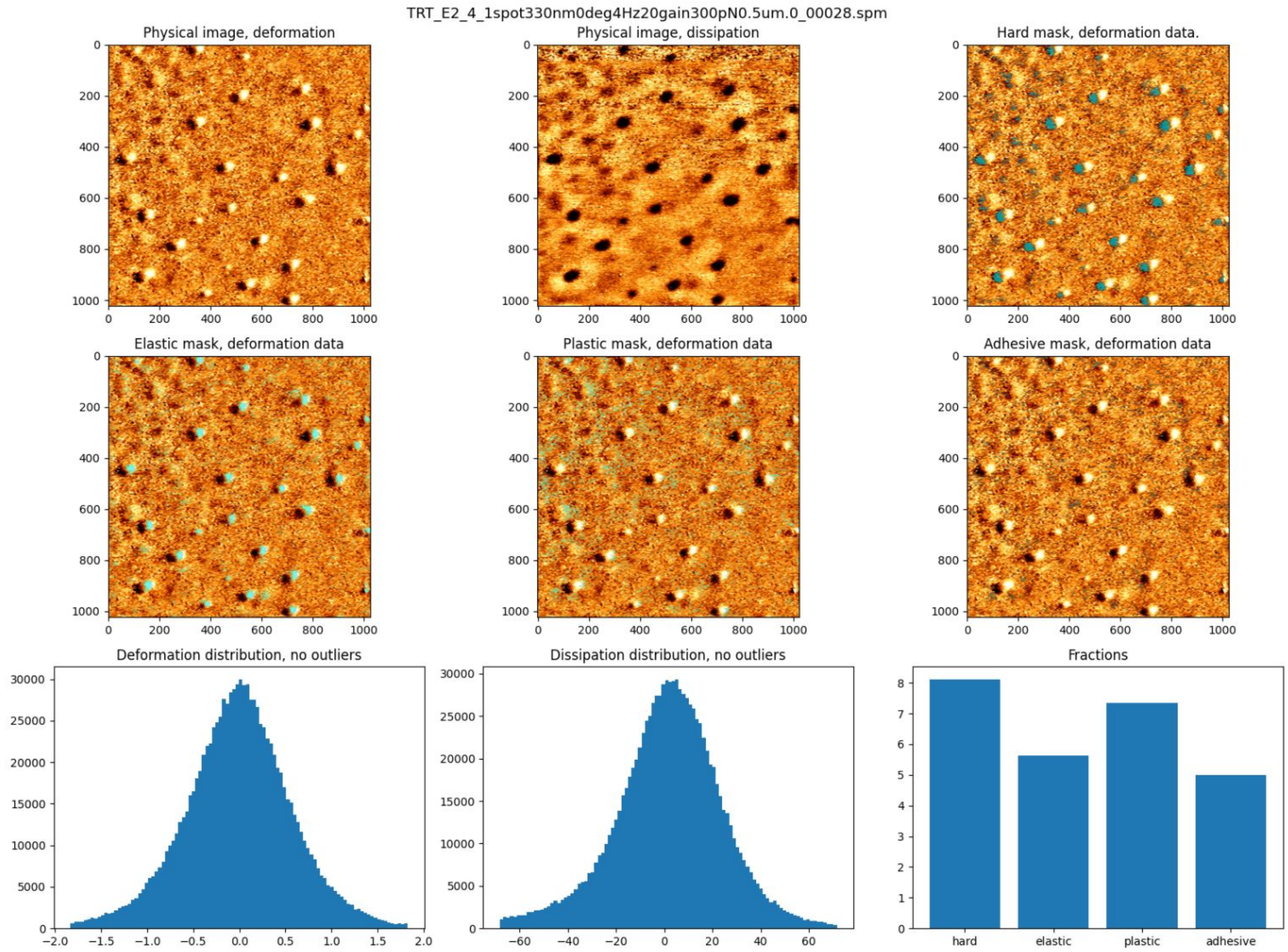

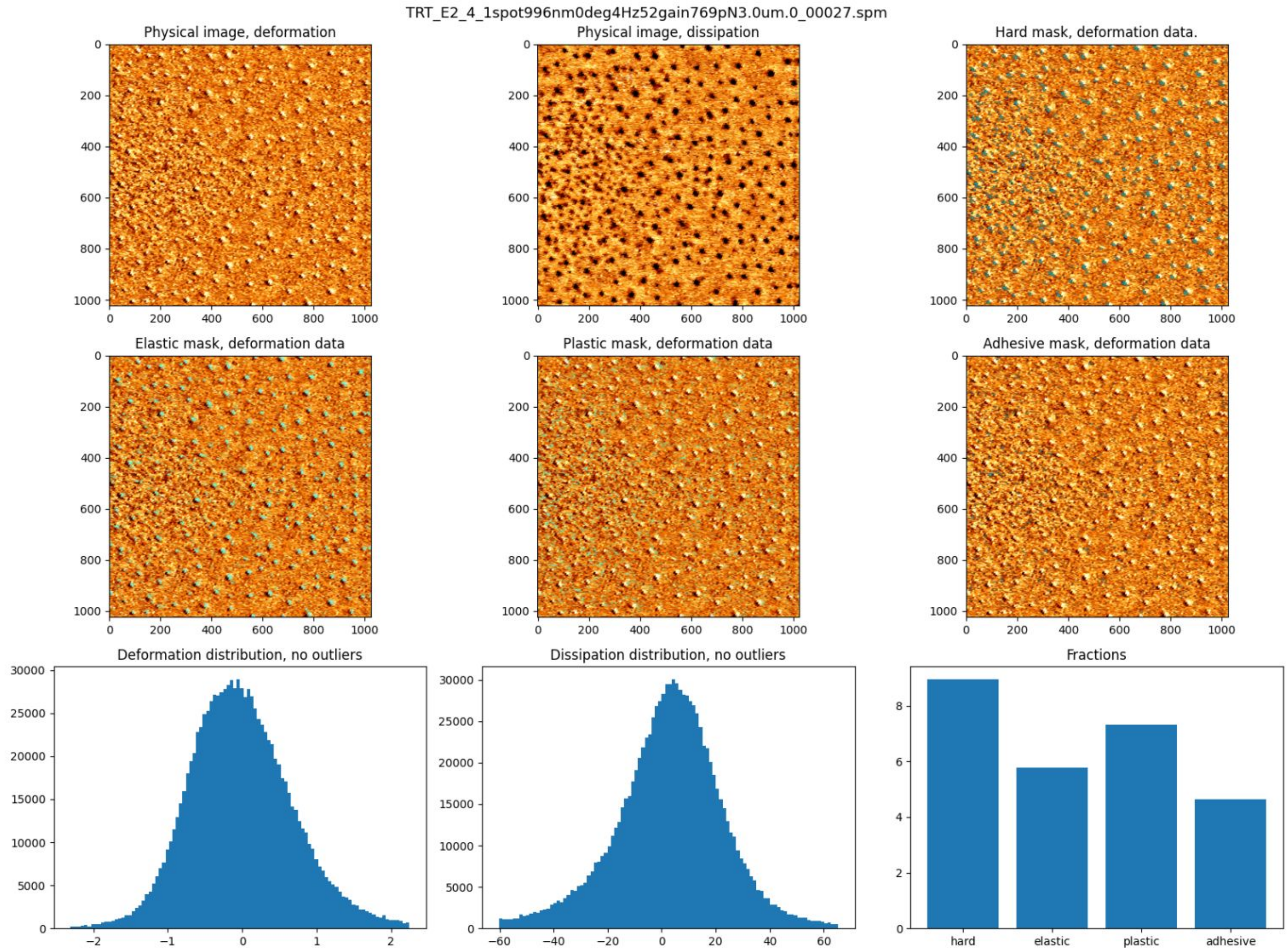
TRT_E2_4_1spot996nm0deg4Hz52gain769pN3.0um.0_00027.spm
Physical image, deformation
Physical image, dissipation
Hard mask, deformation data.
Elastic mask, deformation data
Plastic mask, deformation data
Adhesive mask, deformation data
Deformation distribution, no outliers
Dissipation distribution, no outliers
Fractions
hard
elastic
plastic
adhesive

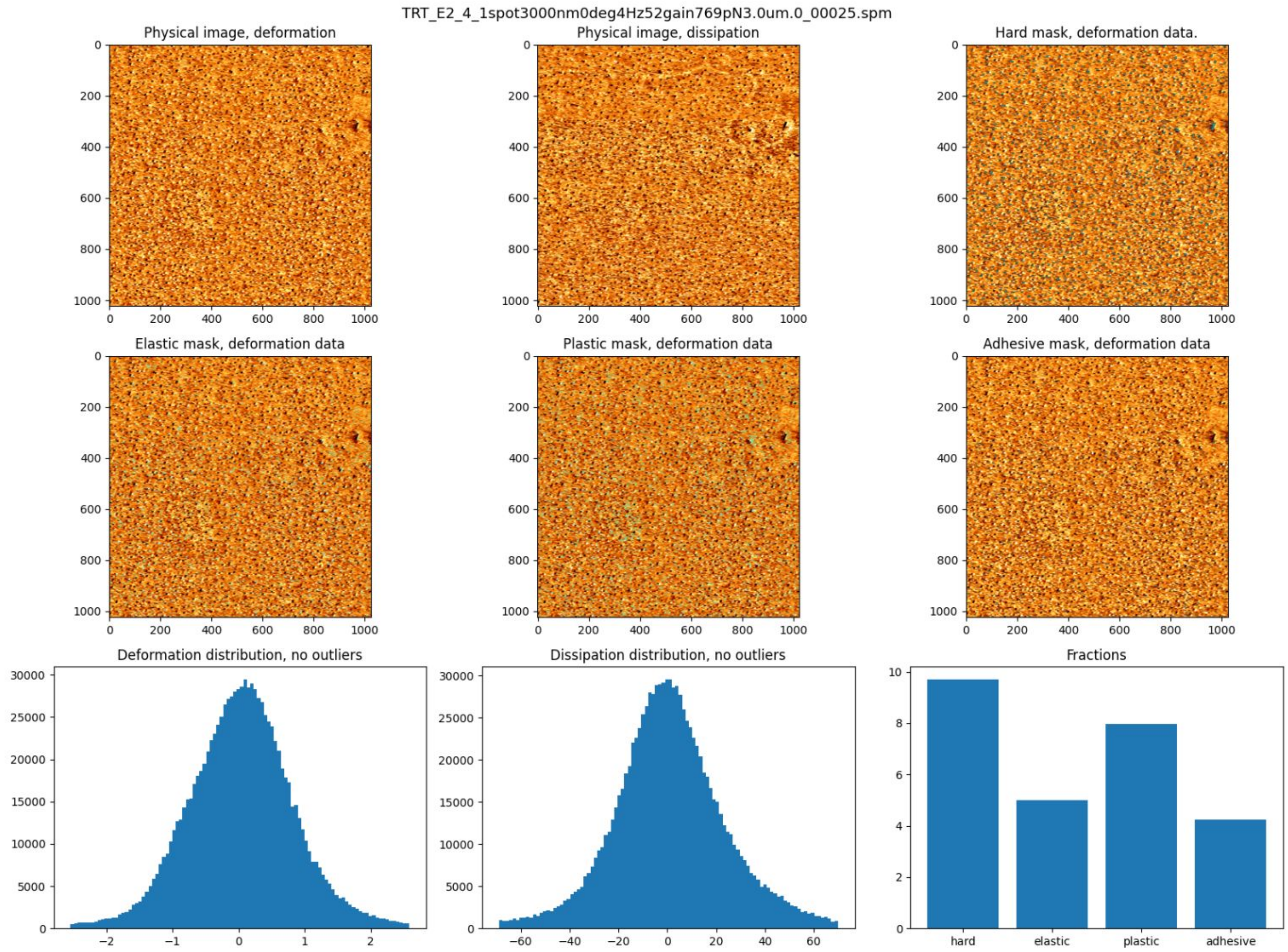

TRT_E2_4_1spot3000nm0deg4Hz52gain769pN3.0um.0_00025.spm
Physical image, deformation
Physical image, dissipation
Hard mask, deformation data.
Elastic mask, deformation data
Plastic mask, deformation data
Adhesive mask, deformation data
Deformation distribution, no outliers
Dissipation distribution, no outliers
Fractions
hard
elastic
plastic
adhesive

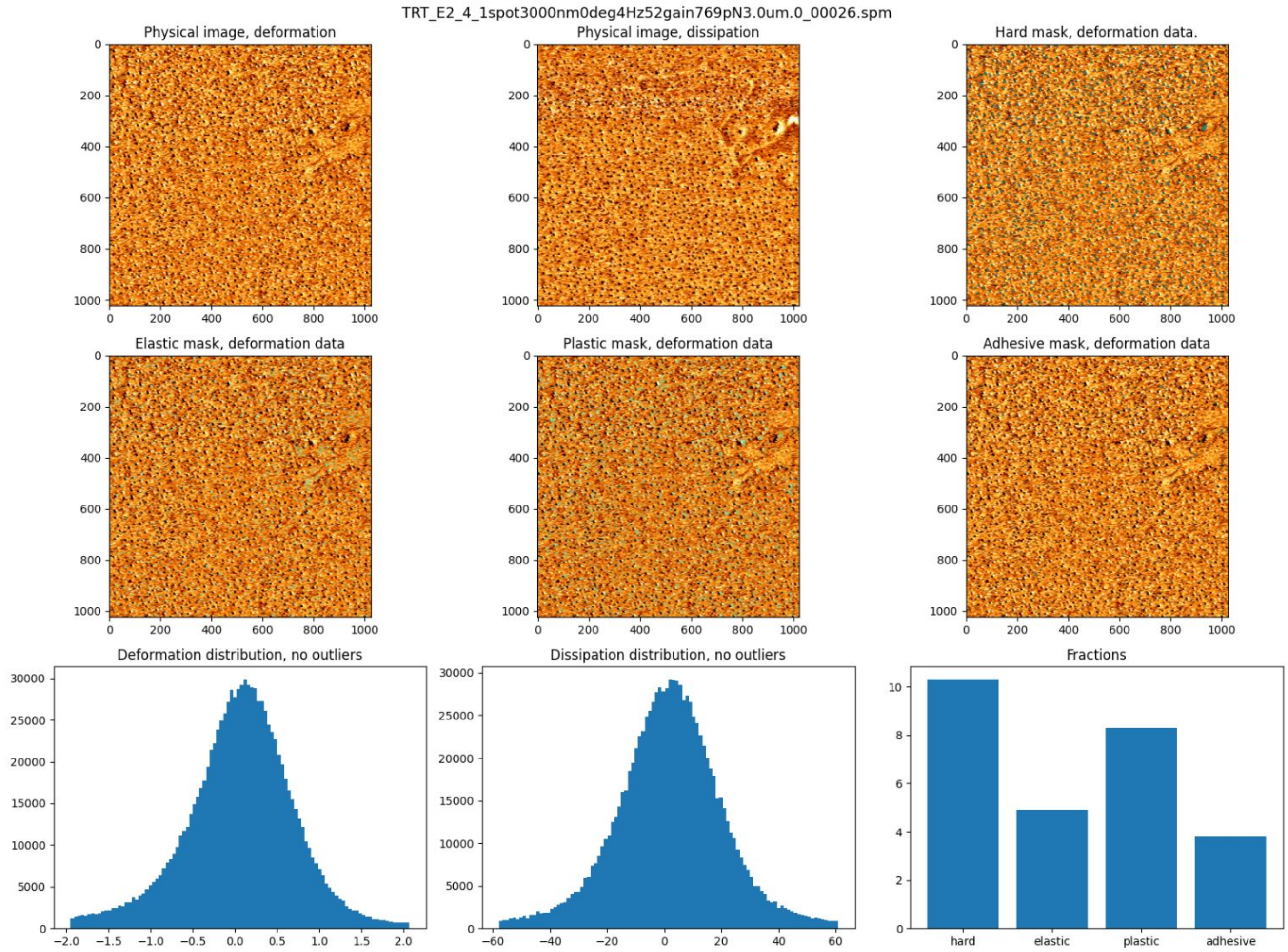
TRT_E2_4_1spot3000nm0deg4Hz52gain769pN3.0um.0_00026.spm
Physical image, deformation
Physical image, dissipation
Hard mask, deformation data.
Elastic mask, deformation data
Plastic mask, deformation data
Adhesive mask, deformation data
Deformation distribution, no outliers
Dissipation distribution, no outliers
Fractions
hard
elastic
plastic
adhesive

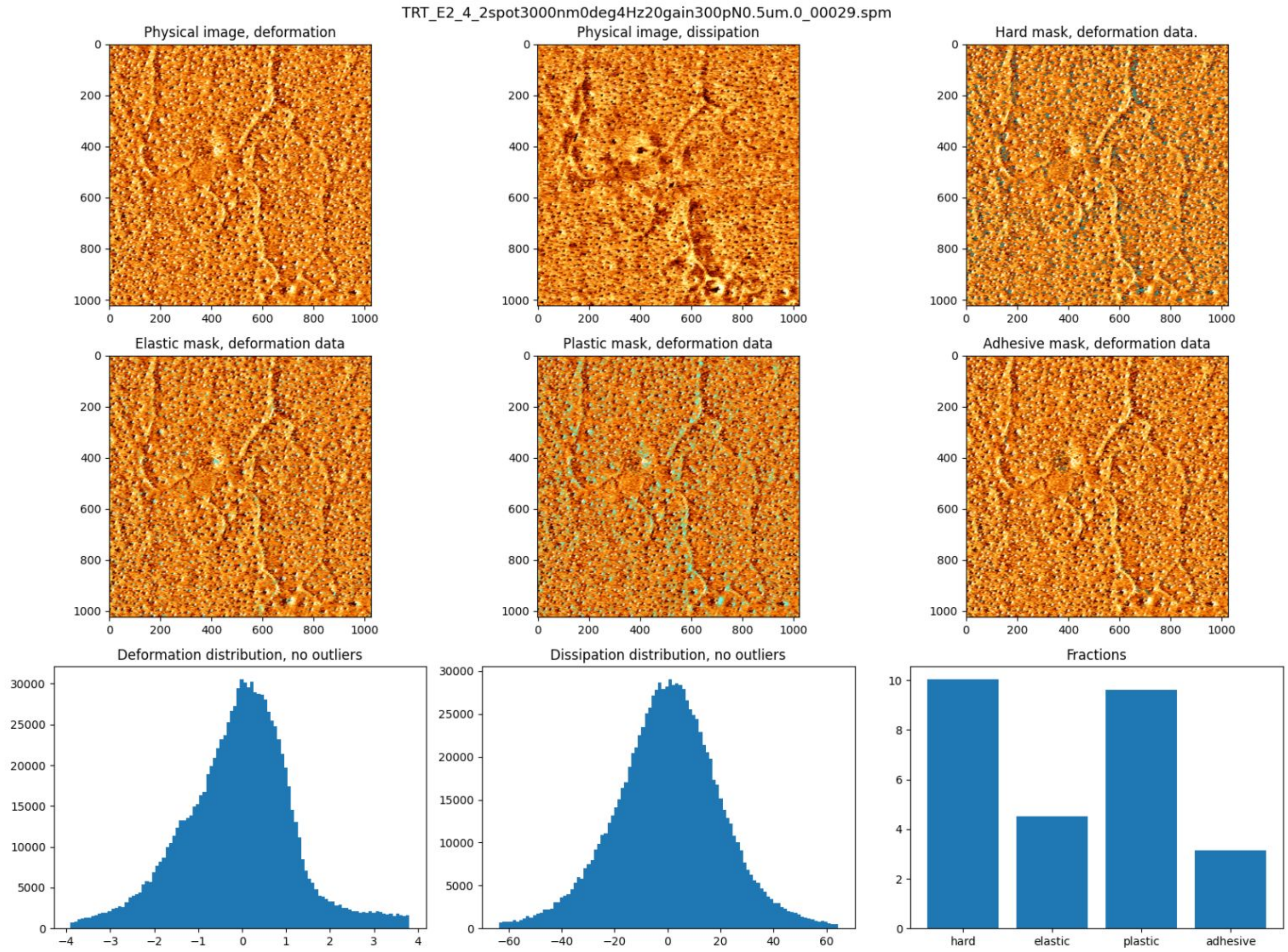
TRT_E2_4_2spot3000nm0deg4Hz20gain300pN0.5um.0_00029.spm
Physical image, deformation
Physical image, dissipation
Hard mask, deformation data.
Elastic mask, deformation data
Plastic mask, deformation data
Adhesive mask, deformation data
Deformation distribution, no outliers
Dissipation distribution, no outliers
Fractions
hard
elastic
plastic
adhesive

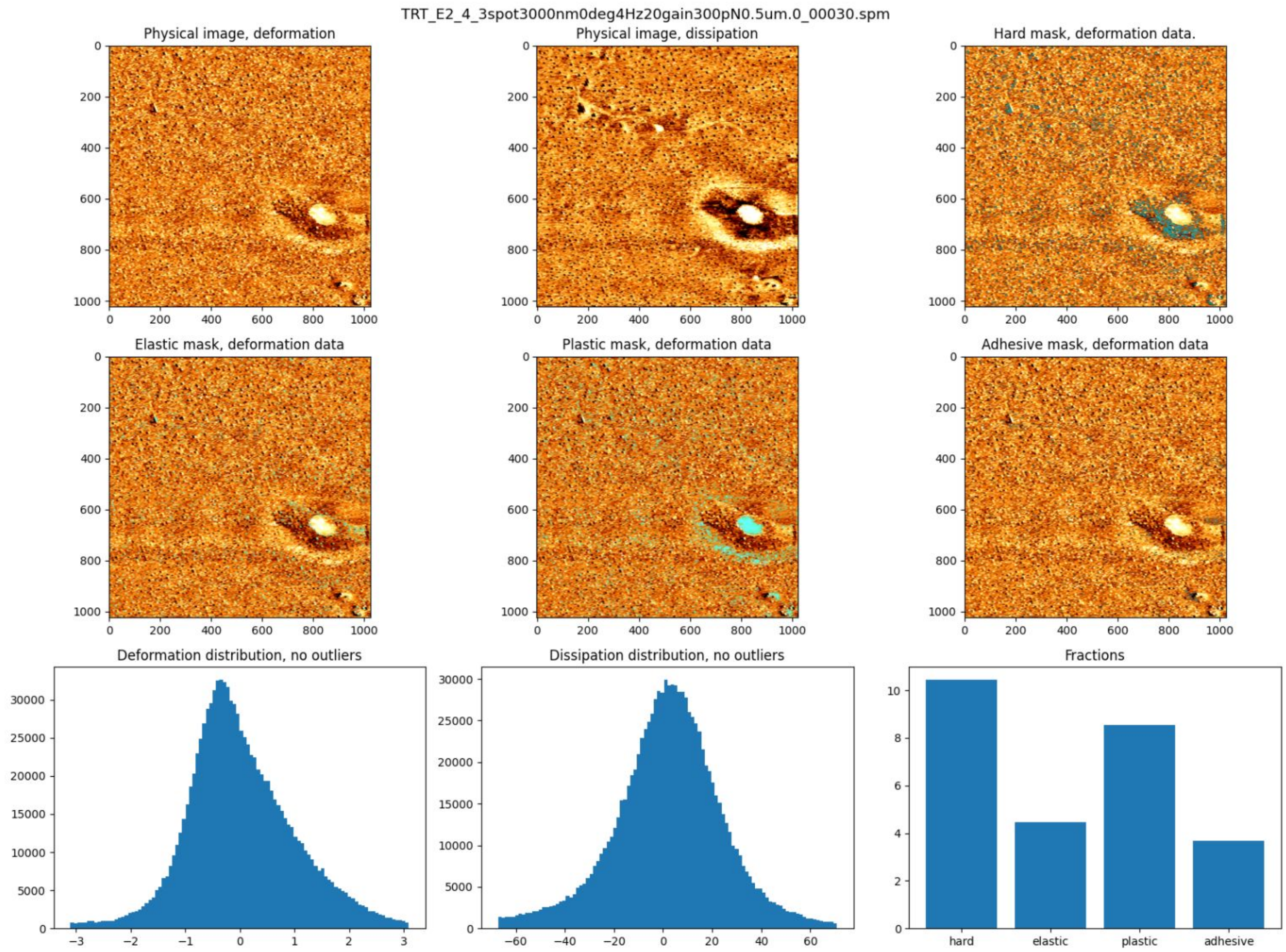
TRT_E2_4_3spot3000nm0deg4Hz20gain300pN0.5um.0_00030.spm
Physical image, deformation
Physical image, dissipation
Hard mask, deformation data.
Elastic mask, deformation data
Plastic mask, deformation data
Adhesive mask, deformation data
Deformation distribution, no outliers
Dissipation distribution, no outliers
Fractions
hard
elastic
plastic
adhesive

TRT-M, Recipe 5

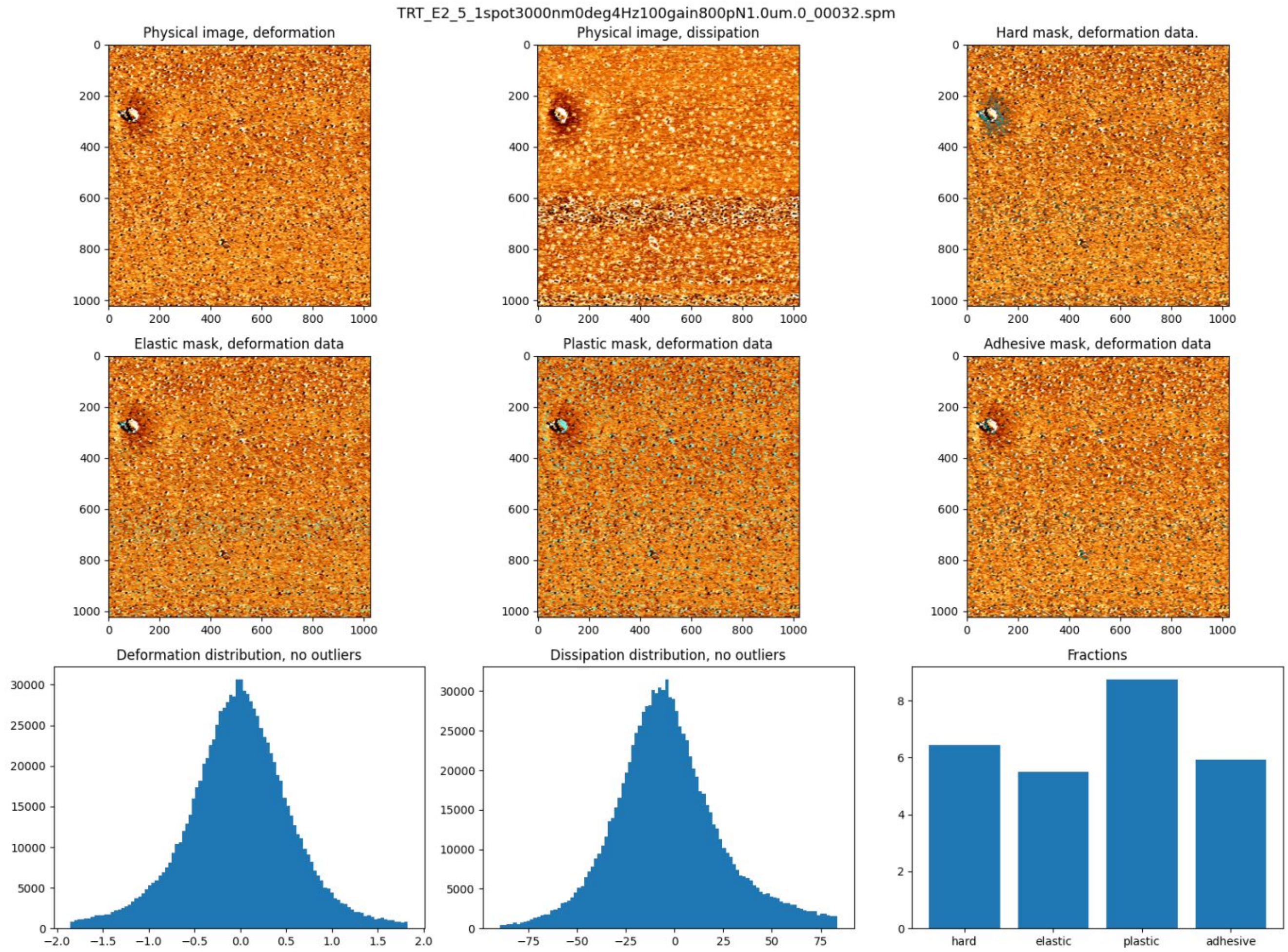

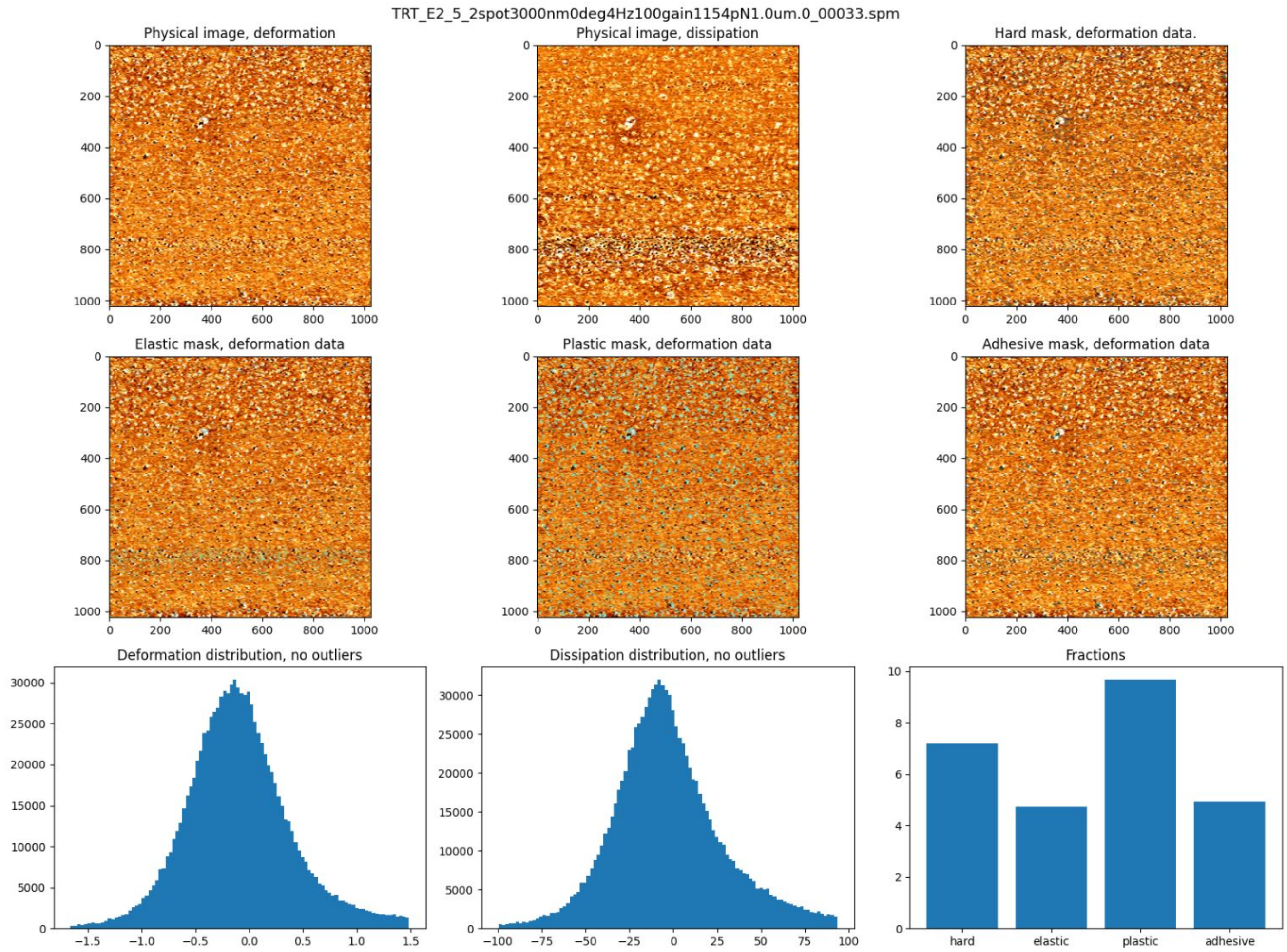

TRT_E2_5_2spot3000nm0deg4Hz100gain1154pN1.0um.0_00033.spm
Physical image, deformation
Physical image, dissipation
Hard mask, deformation data.
Elastic mask, deformation data
Plastic mask, deformation data
Adhesive mask, deformation data
Deformation distribution, no outliers
Dissipation distribution, no outliers
Fractions
hard
elastic
plastic
adhesive

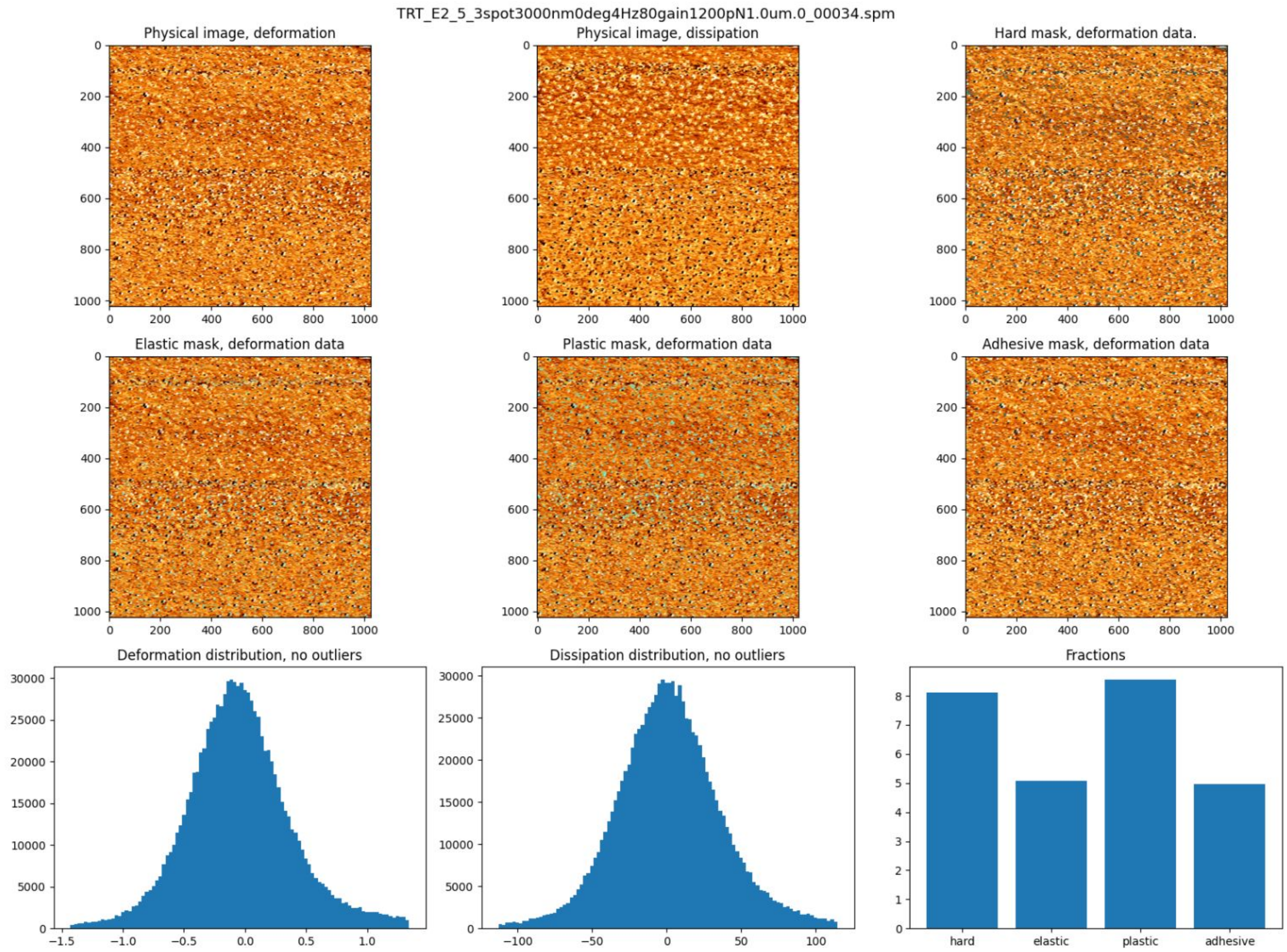
TRT_E2_5_3spot3000nm0deg4Hz80gain1200pN1.0um.0_00034.spm
Physical image, deformation
Physical image, dissipation
Hard mask, deformation data.
Elastic mask, deformation data
Plastic mask, deformation data
Adhesive mask, deformation data
Deformation distribution, no outliers
Dissipation distribution, no outliers
Fractions
hard
elastic
plastic
adhesive

TRT-M, Recipe 6, type 1

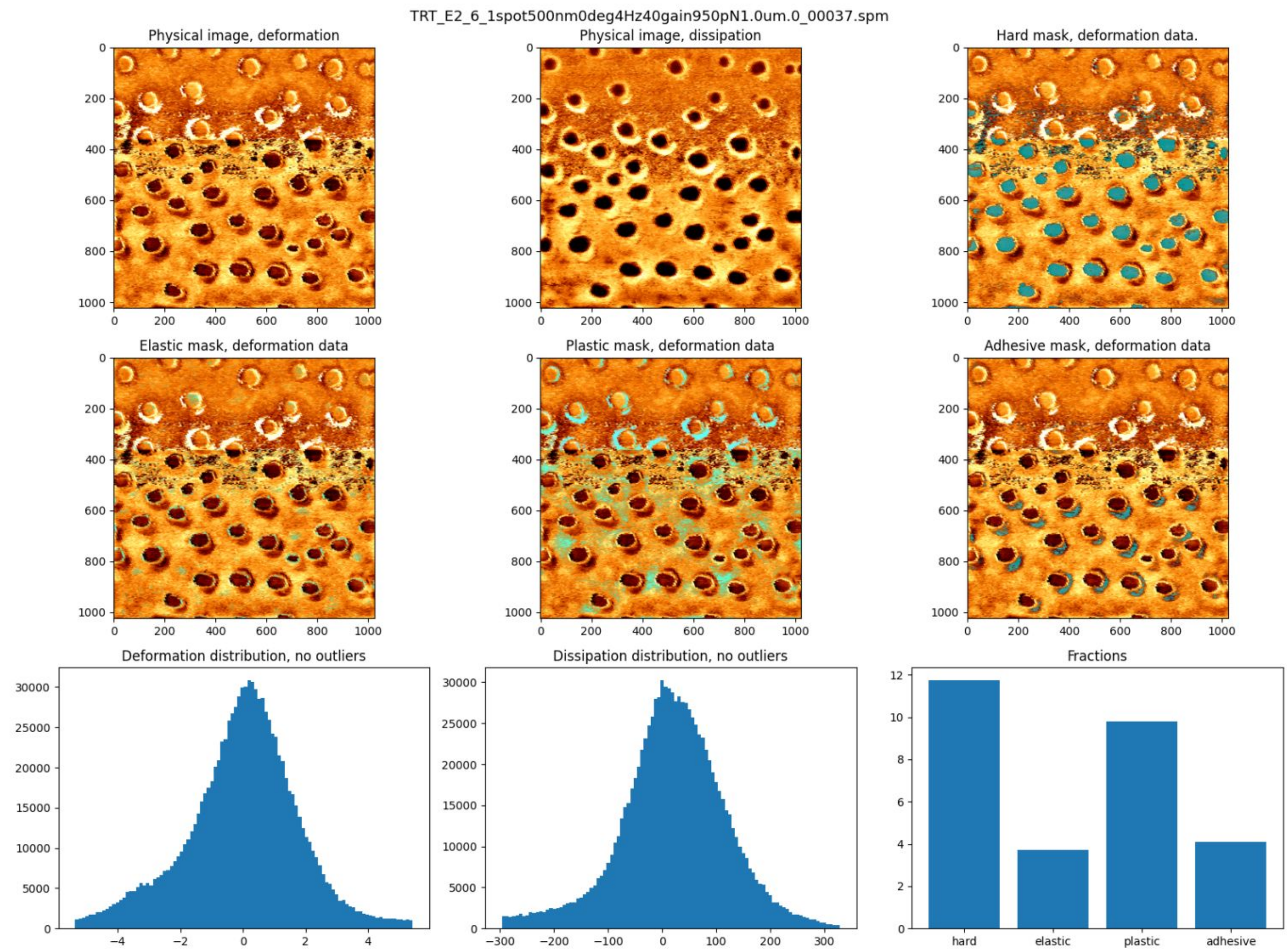

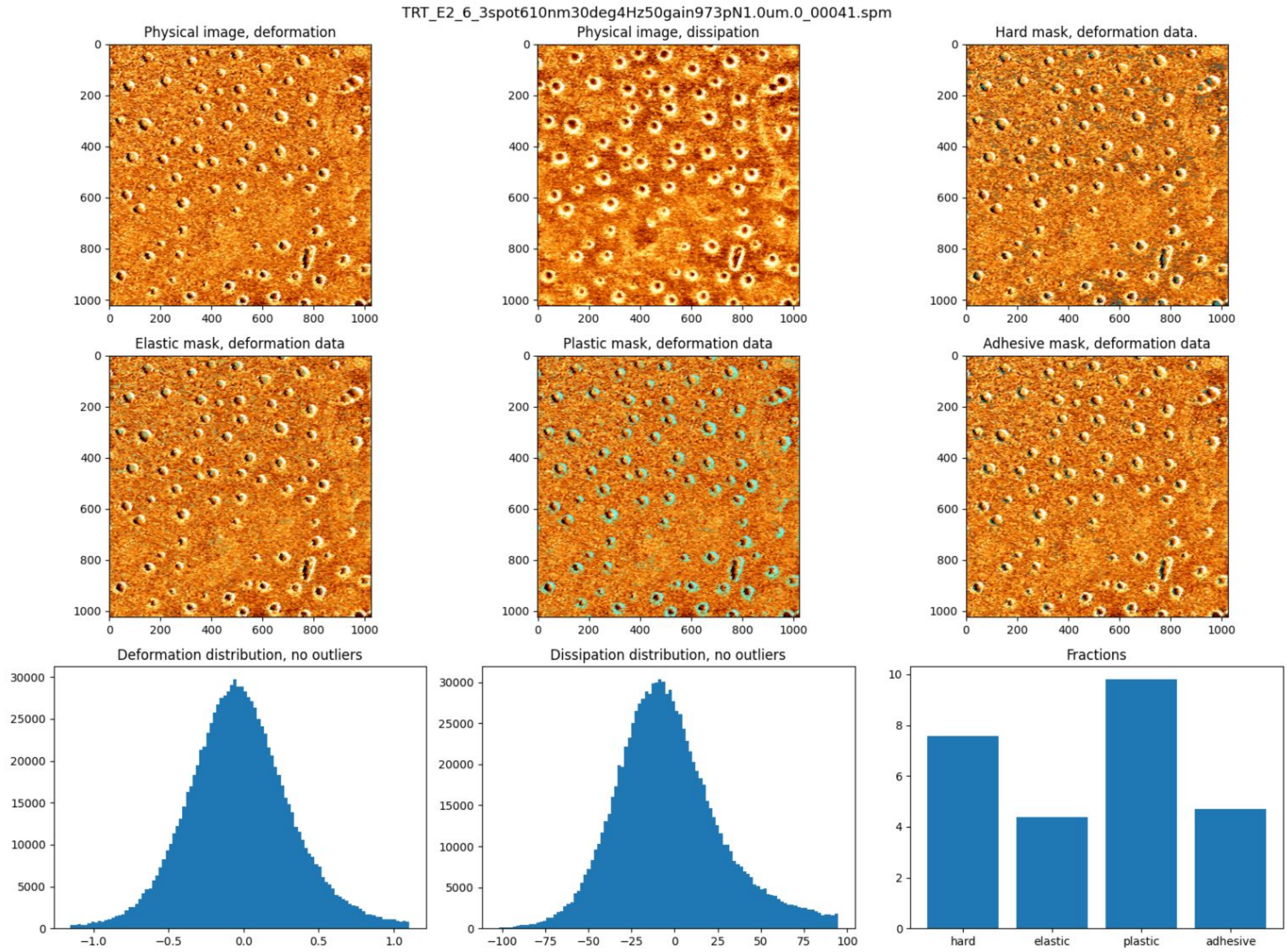

TRT_E2_6_3spot610nm30deg4Hz50gain973pN1.0um.0_00041.spm
Physical image, deformation
Physical image, dissipation
Hard mask, deformation data.
Elastic mask, deformation data
Plastic mask, deformation data
Adhesive mask, deformation data
Deformation distribution, no outliers
Dissipation distribution, no outliers
Fractions
hard
elastic
plastic
adhesive

TRT-M, Recipe 6, type 2

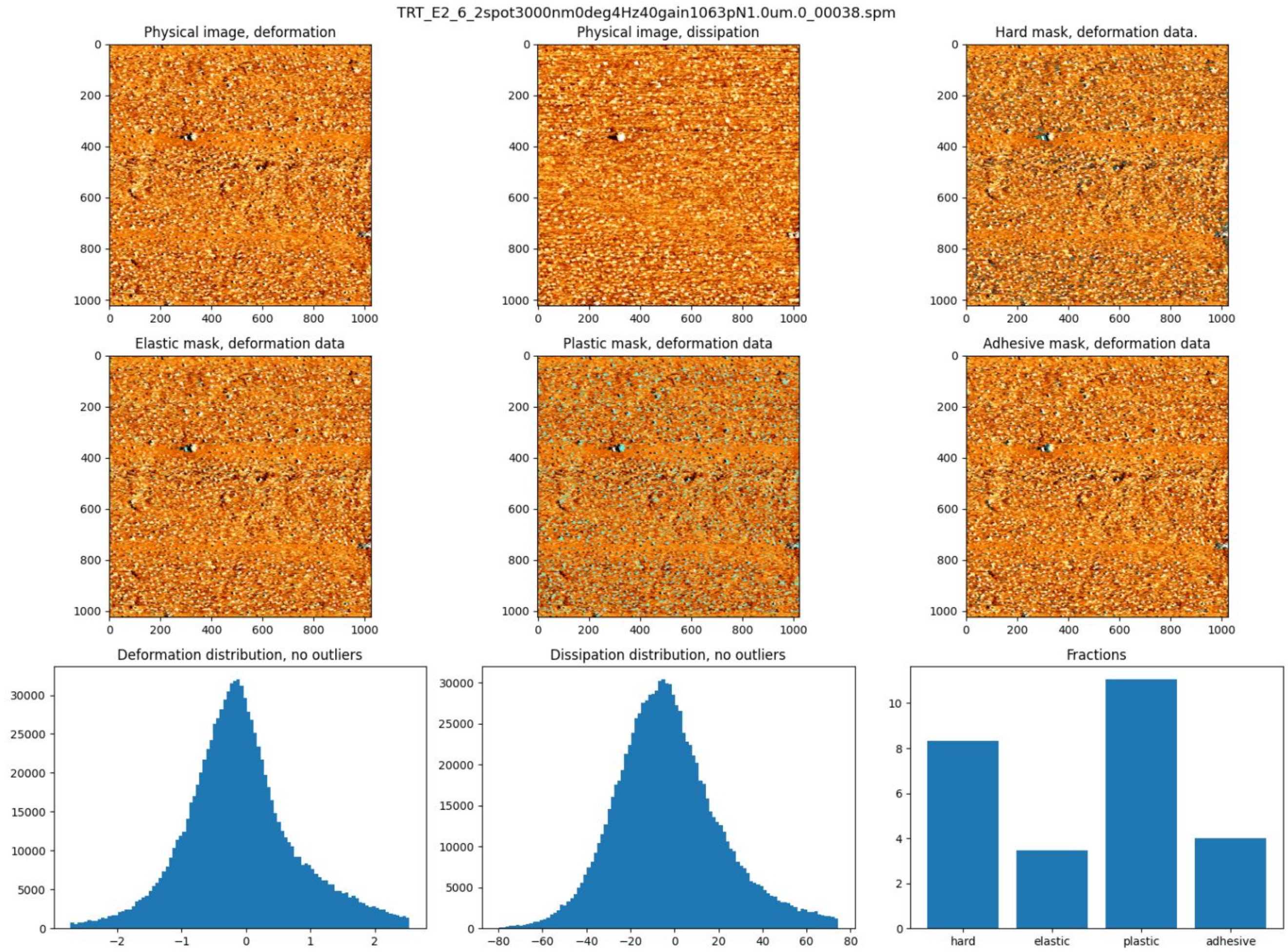

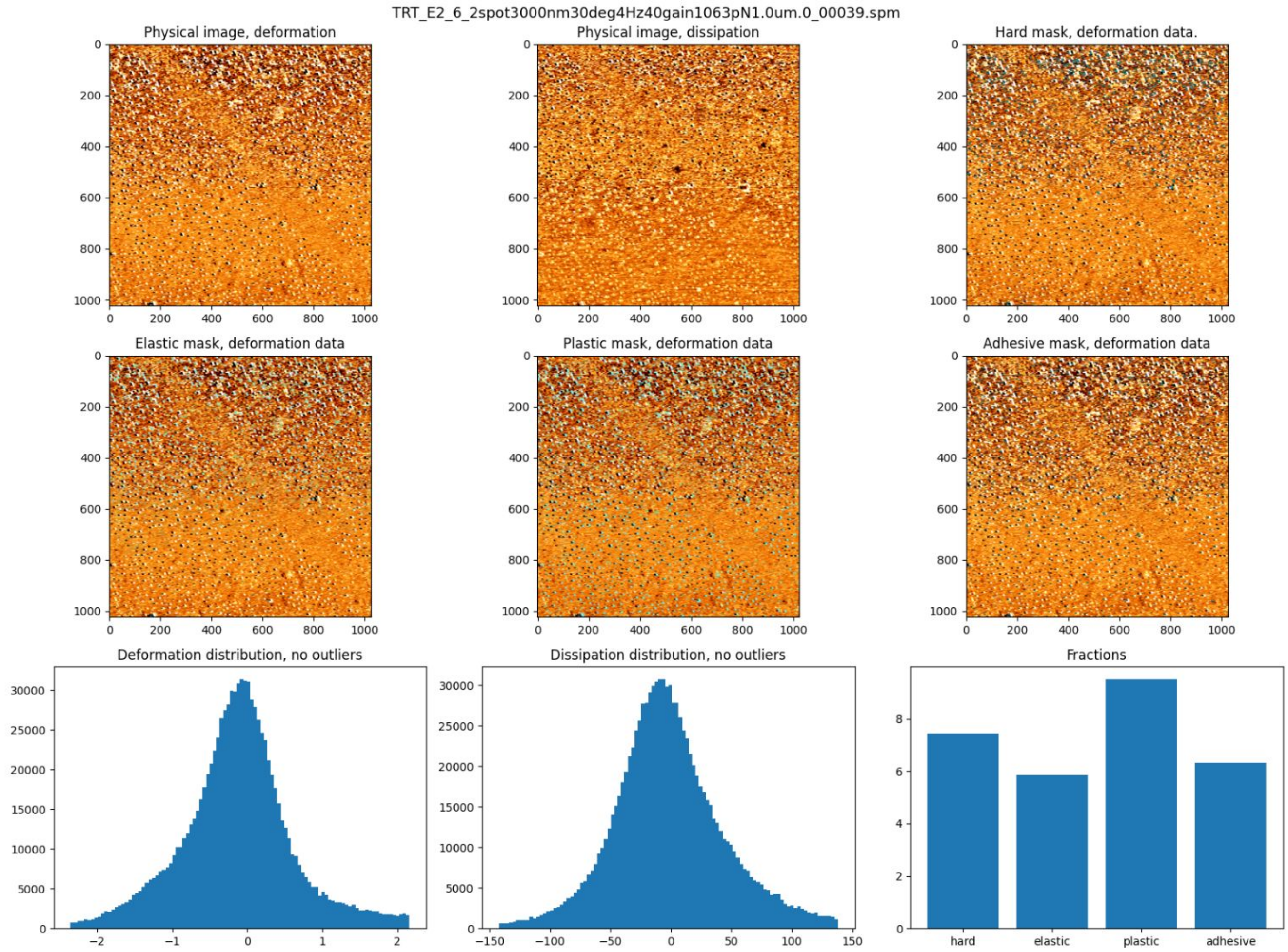
TRT_E2_6_2spot3000nm30deg4Hz40gain1063pN1.0um.0_00039.spm
Physical image, deformation
Physical image, dissipation
Hard mask, deformation data.
Elastic mask, deformation data
Plastic mask, deformation data
Adhesive mask, deformation data
Deformation distribution, no outliers
Dissipation distribution, no outliers
Fractions
hard
elastic
plastic
adhesive

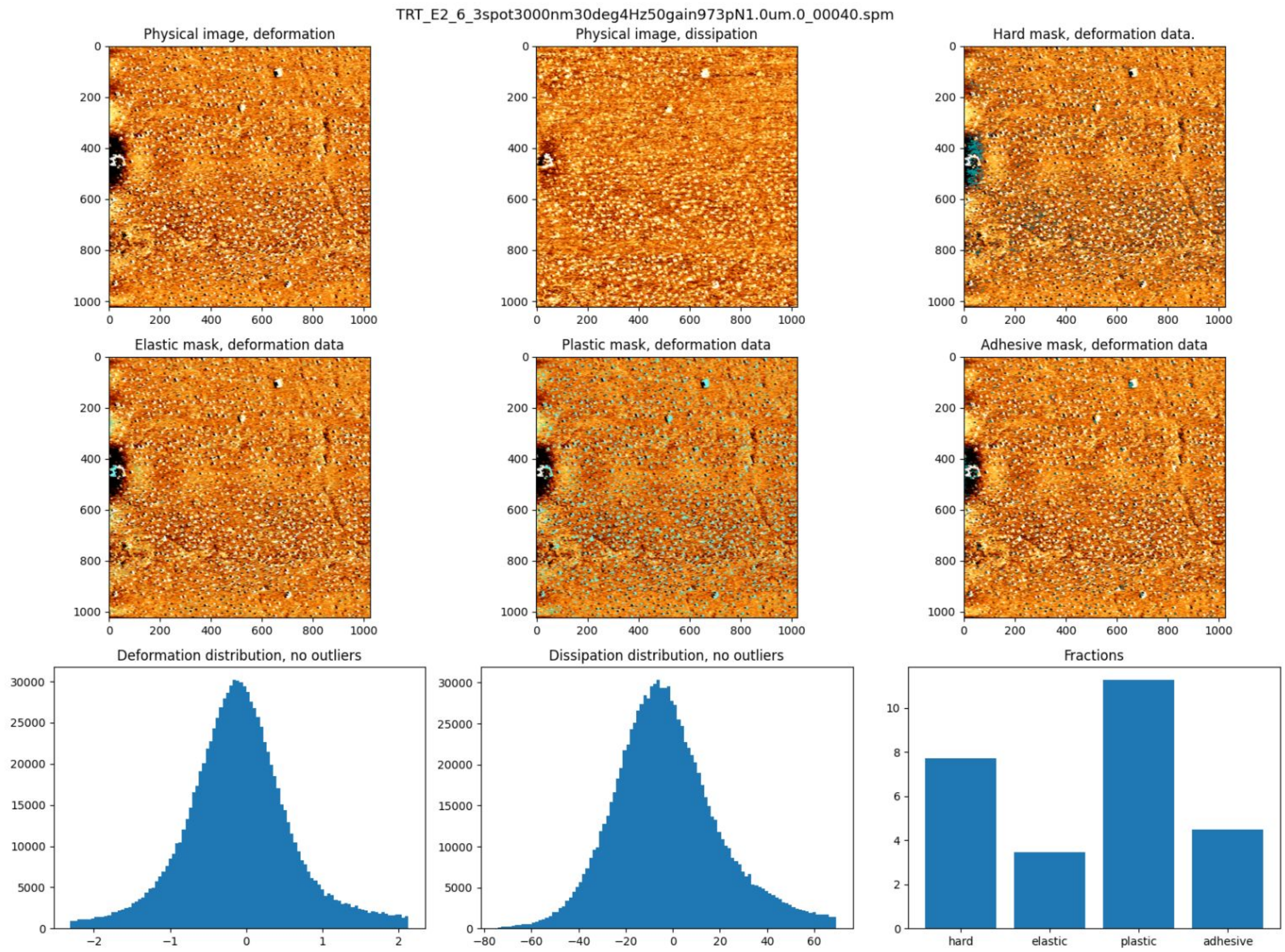
TRT_E2_6_3spot3000nm30deg4Hz50gain973pN1.0um.0_00040.spm
Physical image, deformation
Physical image, dissipation
Hard mask, deformation data.
Elastic mask, deformation data
Plastic mask, deformation data
Adhesive mask, deformation data
Deformation distribution, no outliers
Dissipation distribution, no outliers
Fractions
hard
elastic
plastic
adhesive
