# Supplementary material for: Self-Assembled Monolayers and Solvent Composition Control the Orientation and Work Function of Pyrene-Based [7]Helicenes
Source: Langmuir. 2026 Sep 29;42(40):29622–37. doi: 10.1021/acs.langmuir.6c01280 (PMC13674279; doi:10.1021/acs.langmuir.6c01280)
Supplement: Supplementary file 2 [file la6c01280_si_002.pdf]

Supplementary information:

Self-Assembled Monolayers and Solvent Composition Control Orientation and Work Function of Pyrene-Based [7]Helicenes

Ivan Alic[a+], Katsiaryna Kutsenka[b+], Kristián Kříž[a], Lukáš Kalina[c], David Bína[a,d], Jan Hanus[b], Jaromír Kopeček[e], Anne de la Pena Lopez[a], Radek Litvín[a,d], Milan Mašát*[f], Eva Kaletová[f], Dominik Farka*[a,b]

[a] Department of Chemistry, Faculty of Science, University of South Bohemia in České Budějovice (JU), Branišovská 1760, 370 05 České Budějovice, Czech Republic.
[b] Functional Molecules Group, Institute of Organic Chemistry and Biochemistry (IOCB), Czech Academy of Sciences (CAS); Flemingovo Náměstí 542/2, 160 00 Prague, Czech Republic.
[c] Faculty of Chemistry, Brno University of Technology (BUT), Purkyňova 118, CZ-612 00 Brno, Czech Republic
[d] Institute of Plant Molecular Biology, Biology Centre, Czech Academy of Sciences, Branišovská 31, 370 05 České Budějovice, Czech Republic
[e] Department of Materials Analysis, Institute of Physics (FZU) of Czech Academy of Sciences (CAS); Na Slovance 2, 182 00 Prague 8, Czech Republic.
[f] Molecular Devices Group, Institute of Organic Chemistry and Biochemistry (IOCB), Czech Academy of Sciences (CAS); Flemingovo Náměstí 542/2, 160 00 Prague, Czech Republic.

*corresponding author
[+] These authors contributed equally to the work

Number of pages: 71
Number of figures: 75
Number of schemes:0
Number of tables: 0

Table of Contents:

S2 QNM Guidance and Peculiarities of the Trityl-Group
S3 QNM Results
S59 Spectroscopy
S64 XPS
S65 NMR
S67 Computational
S70 SEM

QNM Guidance and the Peculiarities of the Trityl-Group

The figure below should help the reader interpret our quantitative nano-mechanics (QNM) findings. The central dot presents a feature – in case of topography an elevated mound, in the case of adhesion, deformation, and dissipation the presence (bright) or absence (dark) of the same signal (Fig.S1a).

The most important difference to regular AFM is the possibility to gain insight into the mechanical properties by means of extraction of the adhesive properties and the deformation and dissipation (energy-loss of the cantilever). For our study, the most important channels were that of deformation and dissipation. We therefore created 3D-plots where deformation is represented by topography and dissipation by the colour scale, where black/blue means little to no dissipation, while yellows indicate high dissipation

Simultaneous emergence of signal in both channels indicates **plastic deformation** (Fig.S1a, yellow peaks in 3D plots). Strong deformation in the absence of dissipation indicates **elastic deformation** (blue peaks)- the material returns to its initial height. The absence of signal in both channels indicates a **hard, non-eleastic feature** (likely of crystalline nature, blue lows). Low deformation at high dissipation indicates sticky, adhesive surfaces (yellow lows).

For helicene, we interpret **plastic** features as **disordered** material and **elastic** features as **face-on** oriented helicene, capable to act as a helical coiled spring on the nanoscale. The hard features are likely made-up of edge-on oriented helicene, unable to deform elastically, but sufficiently ordered to withstand the force transferred by the AFM-tip.

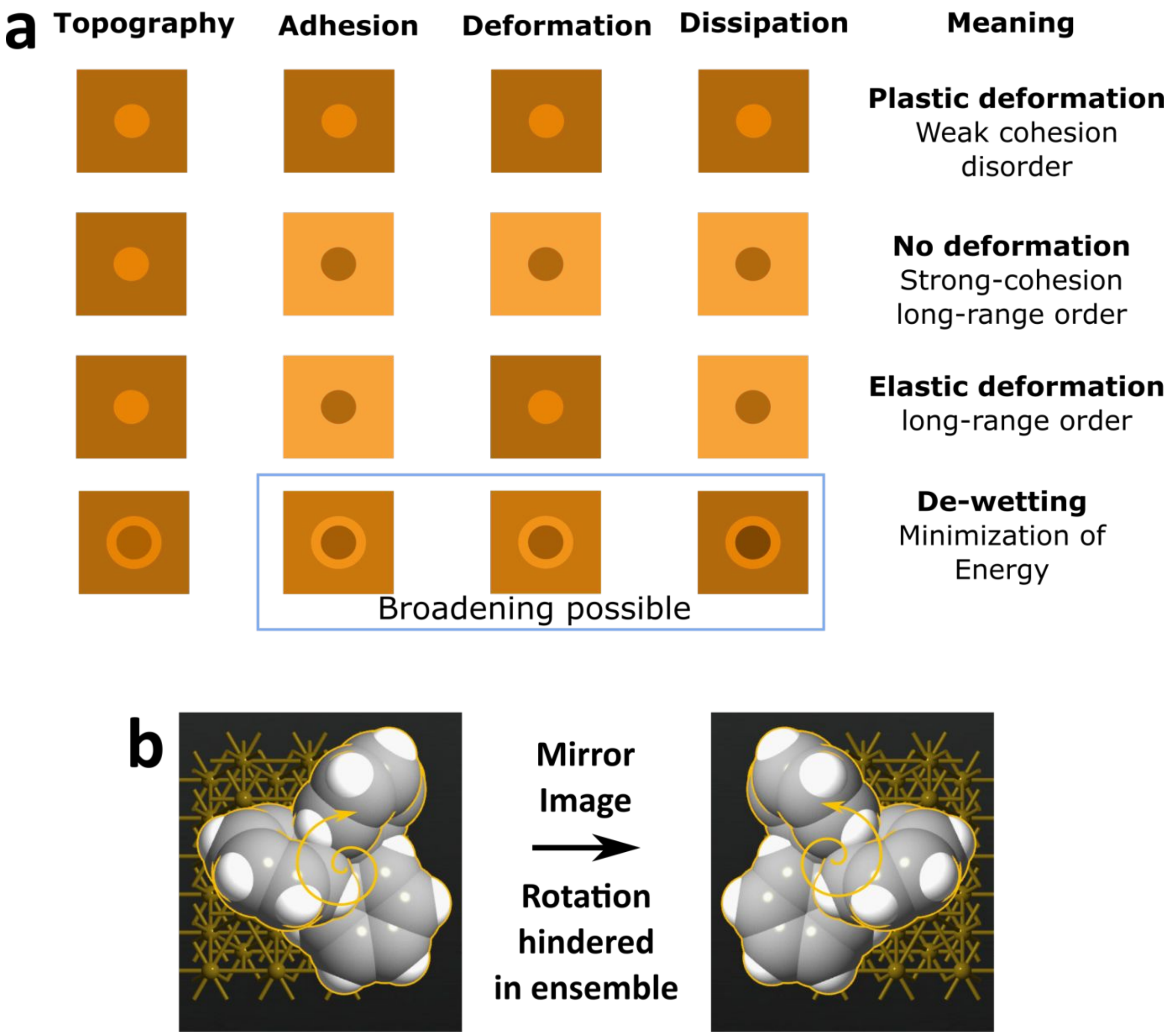

Figure S1: (a) Guide to interpretation of observed behaviour in QNM. Dewetting mechanism illustrates an example where poor inter-molecular cohesion and high adhesion to the tip results in high energy dissipation. **(b) Trityl group on the surface may form chiral structures with weak energy-barrier.**

Further, we need to discuss the trityl group. Intrinsically, it is non-chiral, with each phenyl group essentially free to rotate around the single-bond. However, there is evidence of stereodynamic behaviour: In the presence of a stereogenic centre, *i.e.* a chiral molecule, said chirality can be transmitted to the trytil-group.[1–3] This could be further enhanced when we consider a surface covered in anchored trityl-groups, as it is the case with our trityl- self-assembled-monolayer (TRT-SAM). There, the phenyl-functionalities are pressed closely together and influence each other sterically. The interaction-energy of two phenyl-like rings was calculated to change with their relative angle at which they interact by about 0.2-0.8 kcal/mol, [4] a substantial amount of energy if we consider four such interactions are on a single trityl group- which is in turn surrounded by multiple trityl-groups itself. In other word, we can quickly arrive at substantial energies with relatively small ensembles. In particular if we add a stereogenic centre in the form of helicene.

QNM Results

HOPG

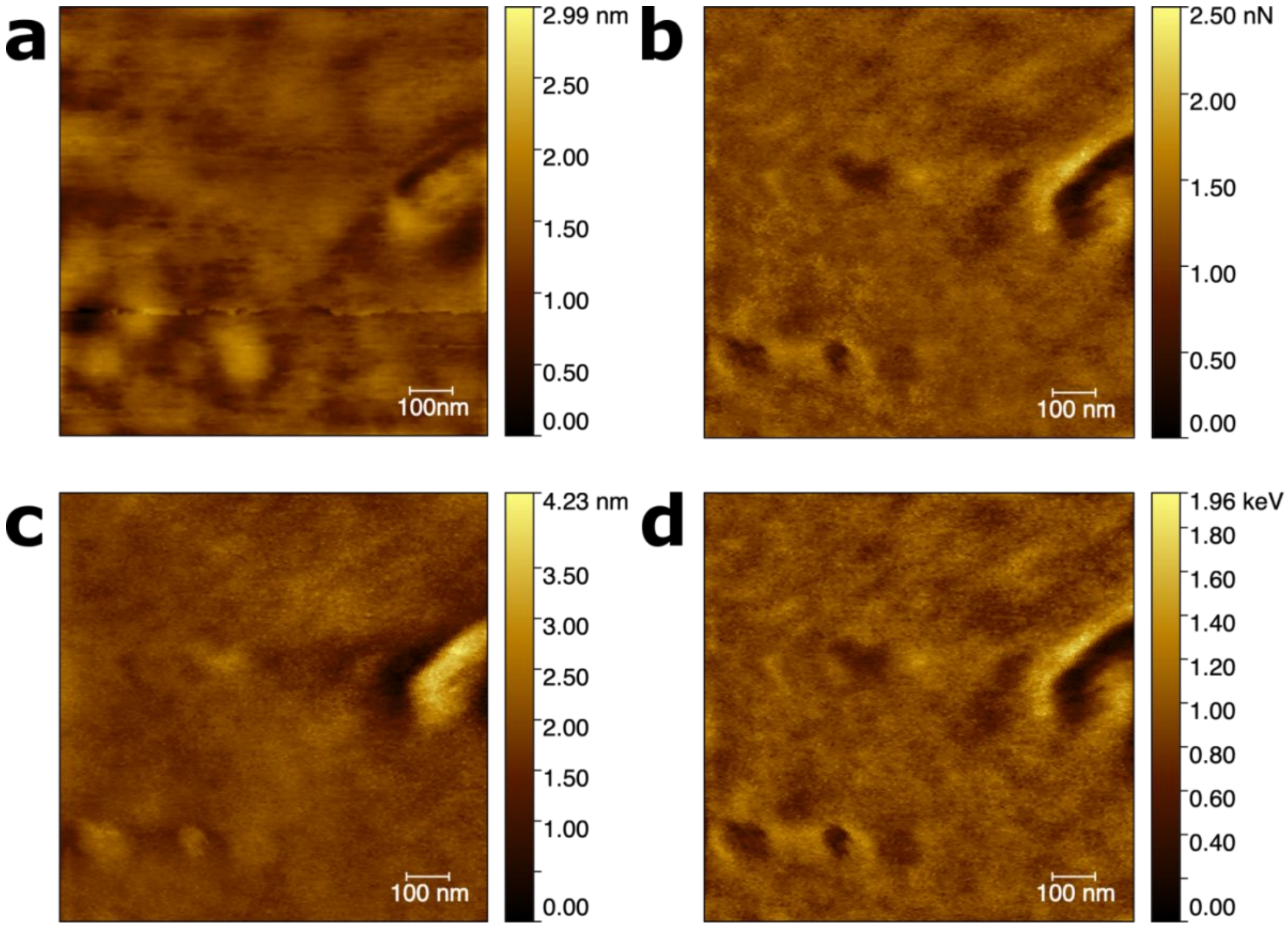

Figure S2: HOPG 1 (a-d) topography, adhesion, deformation, and dissipation, respectively. Topographic features appear to be face-on oriented material.

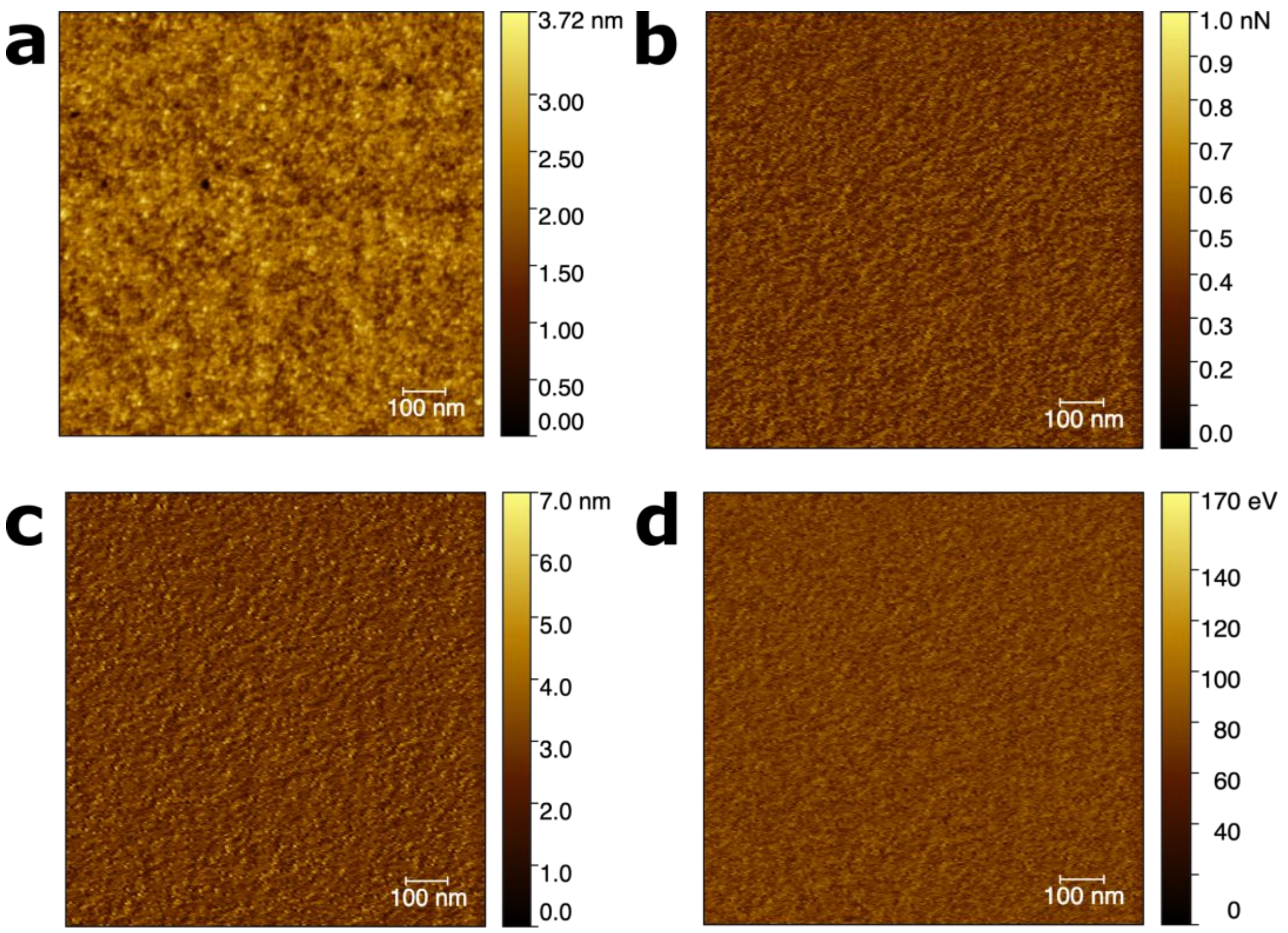

Figure S3: HOPG 2 (a-d) topography, adhesion, deformation, and dissipation, respectively. Topographic features appear to be face-on oriented material.

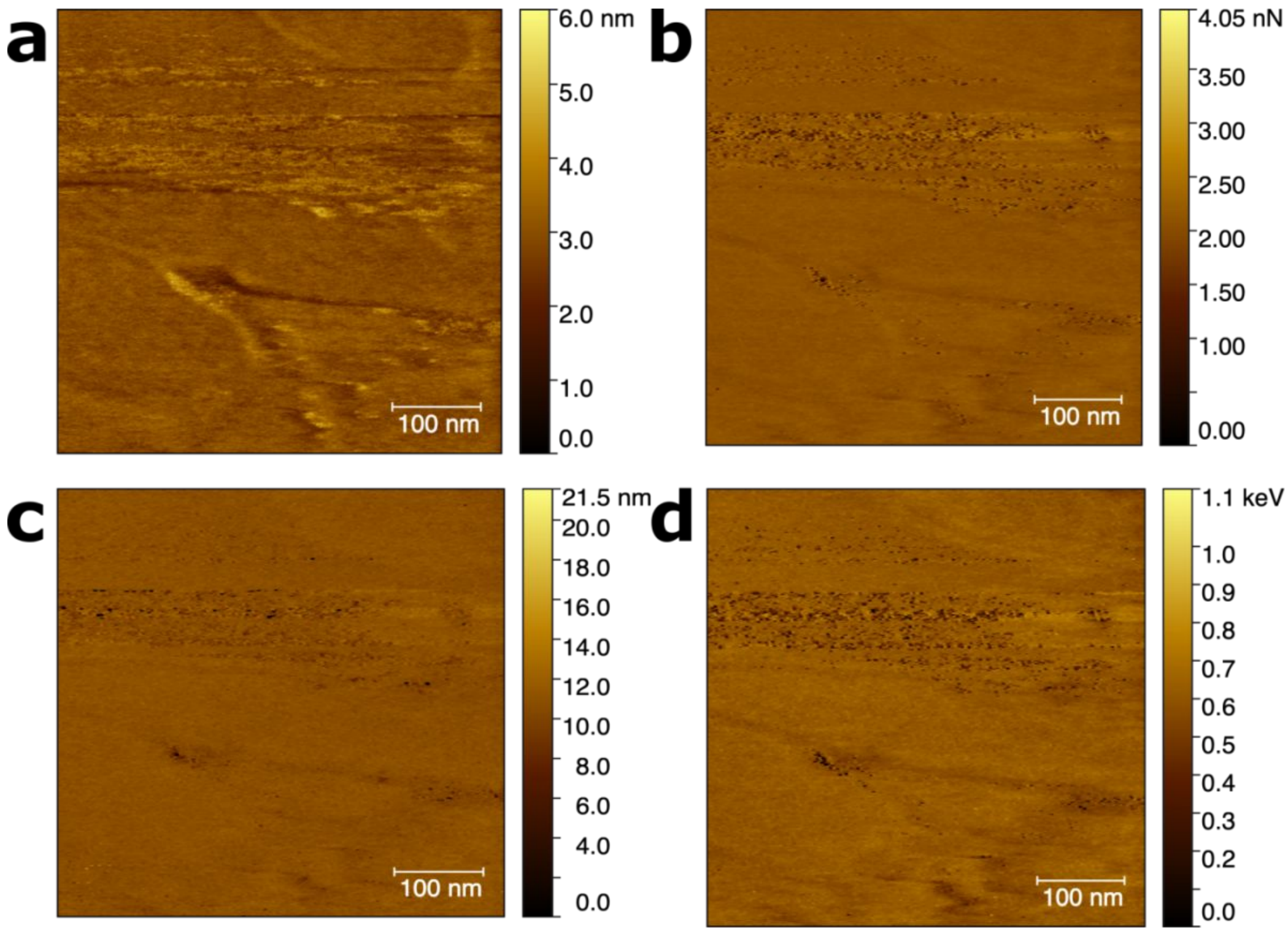

Figure S4: HOPG 3 (a-d) topography, adhesion, deformation, and dissipation, respectively. The observed behaviour indicates poor adhesion/absence of helicene-deposition.

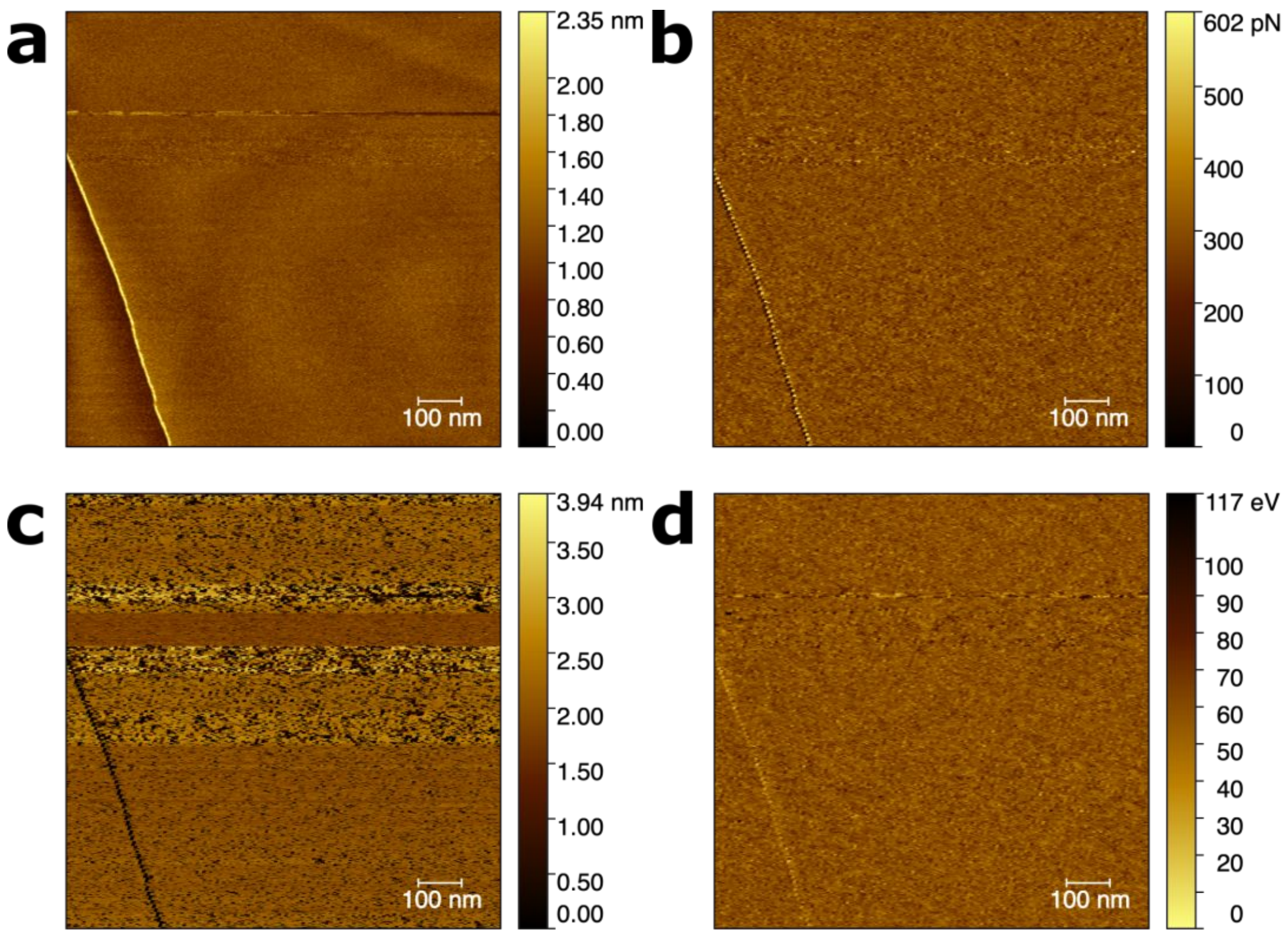

Figure S5: HOPG 4 (a-d) topography, adhesion, deformation, and dissipation, respectively. The observed behaviour indicates poor adhesion/absence of helicene-deposition.

AD-SAM

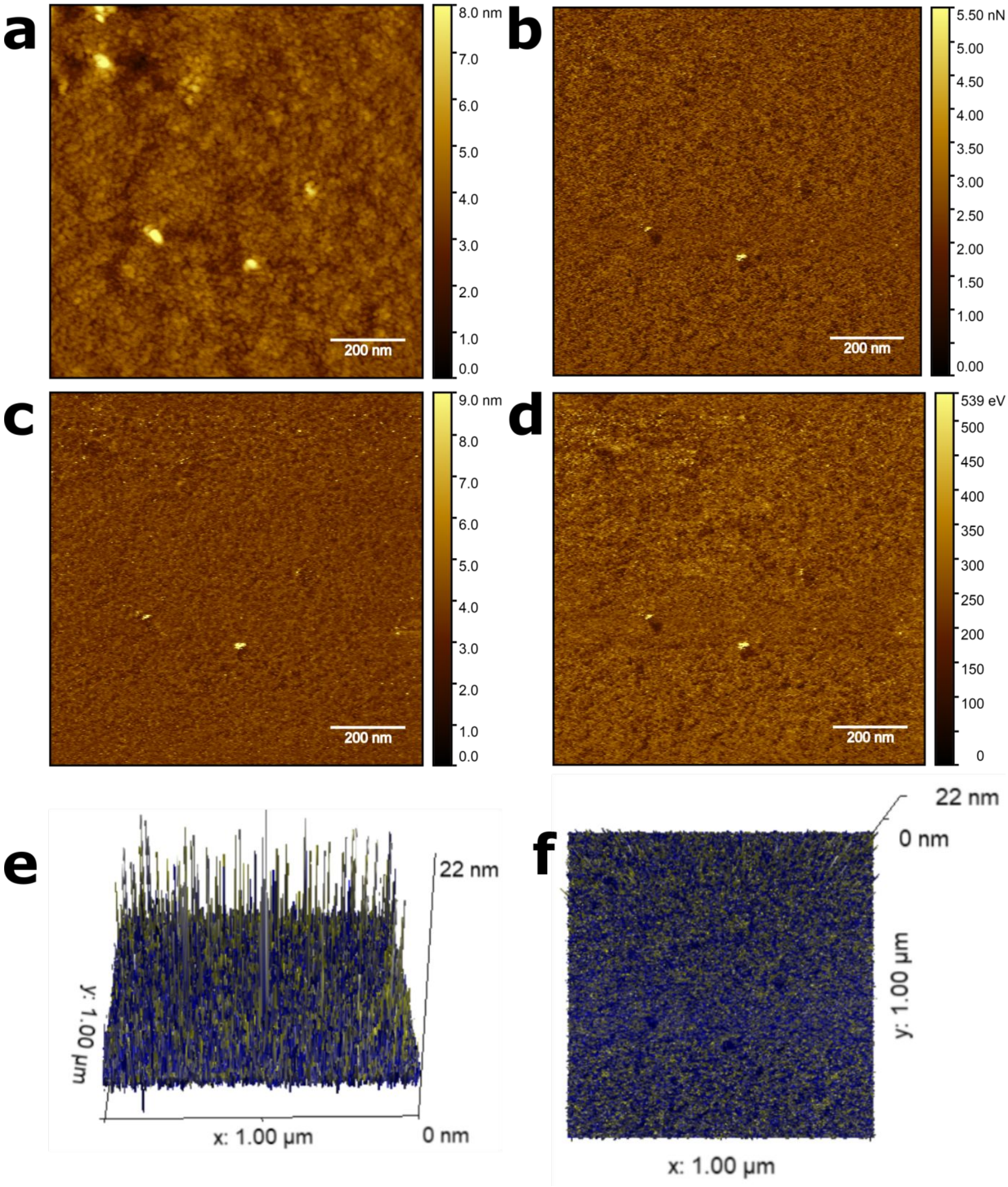

Figure S6: AD1 assembly type 1. (a-d) topography, adhesion, deformation, and dissipation, respectively. (e,f) Correlation of Deformation and Dissipation channel. Both assembly types are dominated by hard features and plastic deformation with. Few blue peaks indicate elastic deformation (5%), indicative of the presence of face-on material.

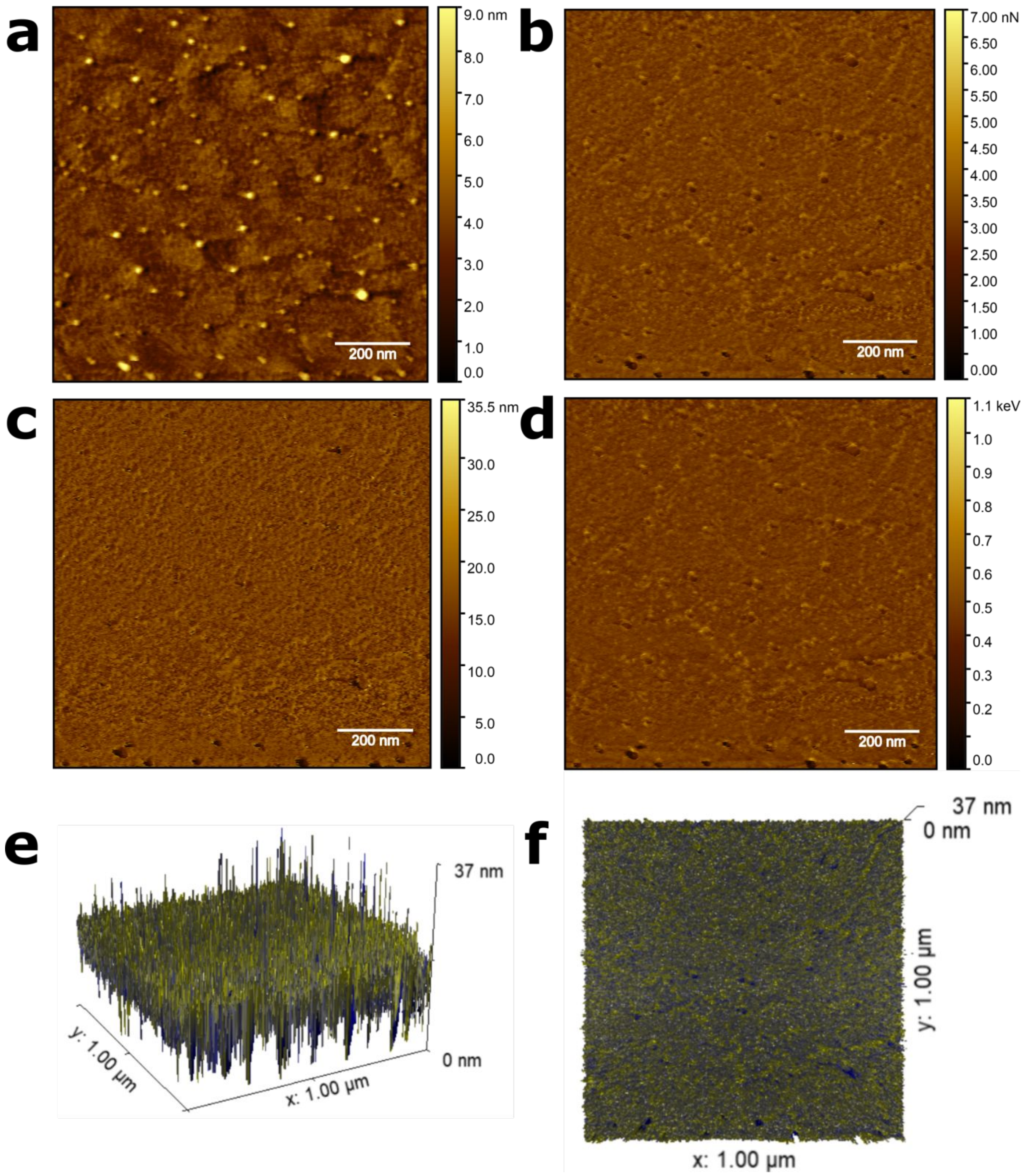

Figure S7: AD2 type 2. (a-d) topography, adhesion, deformation, and dissipation, respectively. (e,f) Correlation of Deformation and Dissipation channel. This assembly type is dominated by plastic deformation (yellow peaks) and about 10% of the are consists of hard features. Elastic deformation (off blue peaks, ~3%) is rare, i.e. edge-on deposition is prevalent in both assembly types.

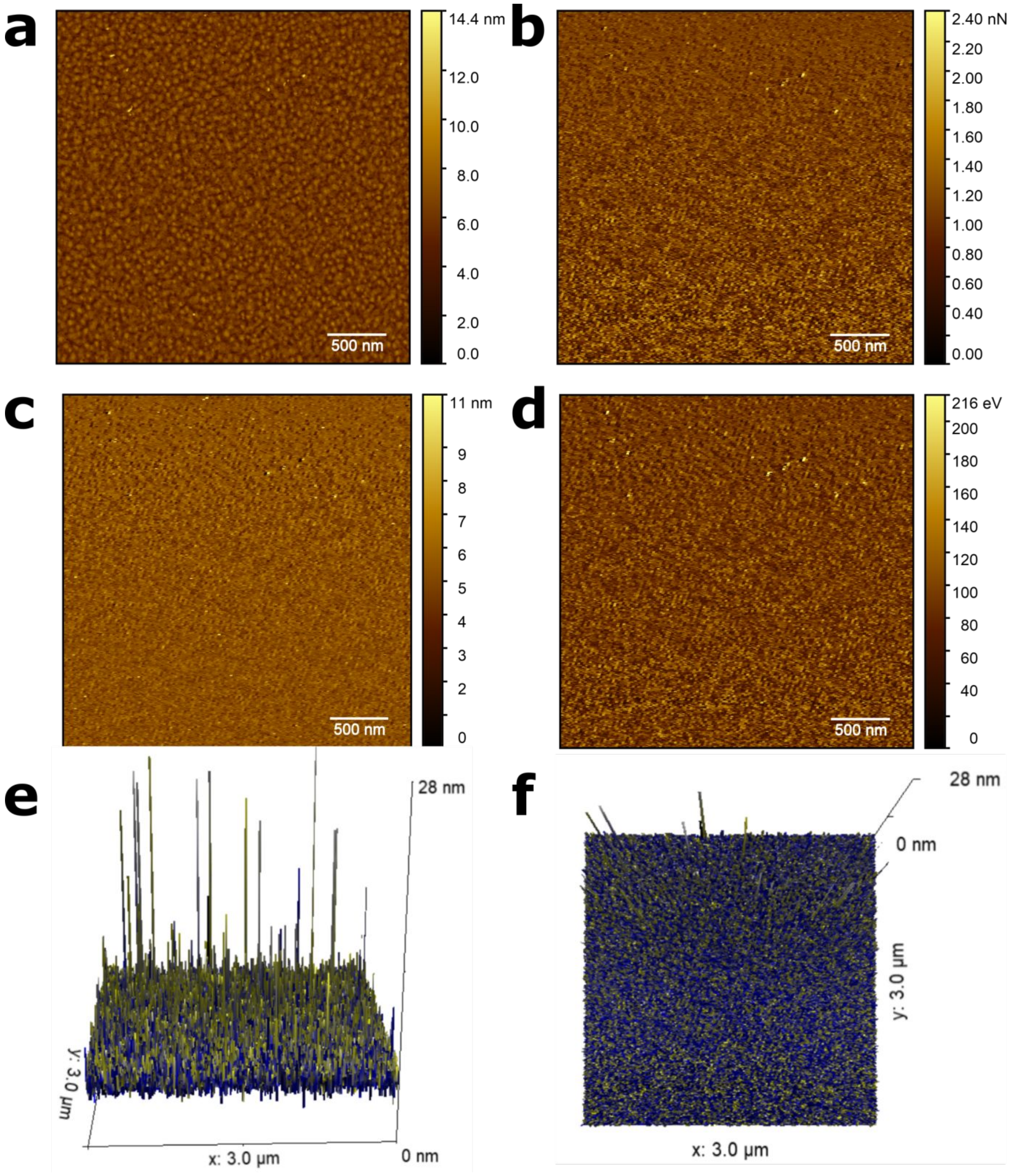

Figure S8: AD3 (a-d) topography, adhesion, deformation, and dissipation, respectively. (e,f) Correlation of Deformation and Dissipation channel. A single assembly type is observed, evidently similar to AD2 type 2. Edge-on deposition is dominant with little face-on oriented material.

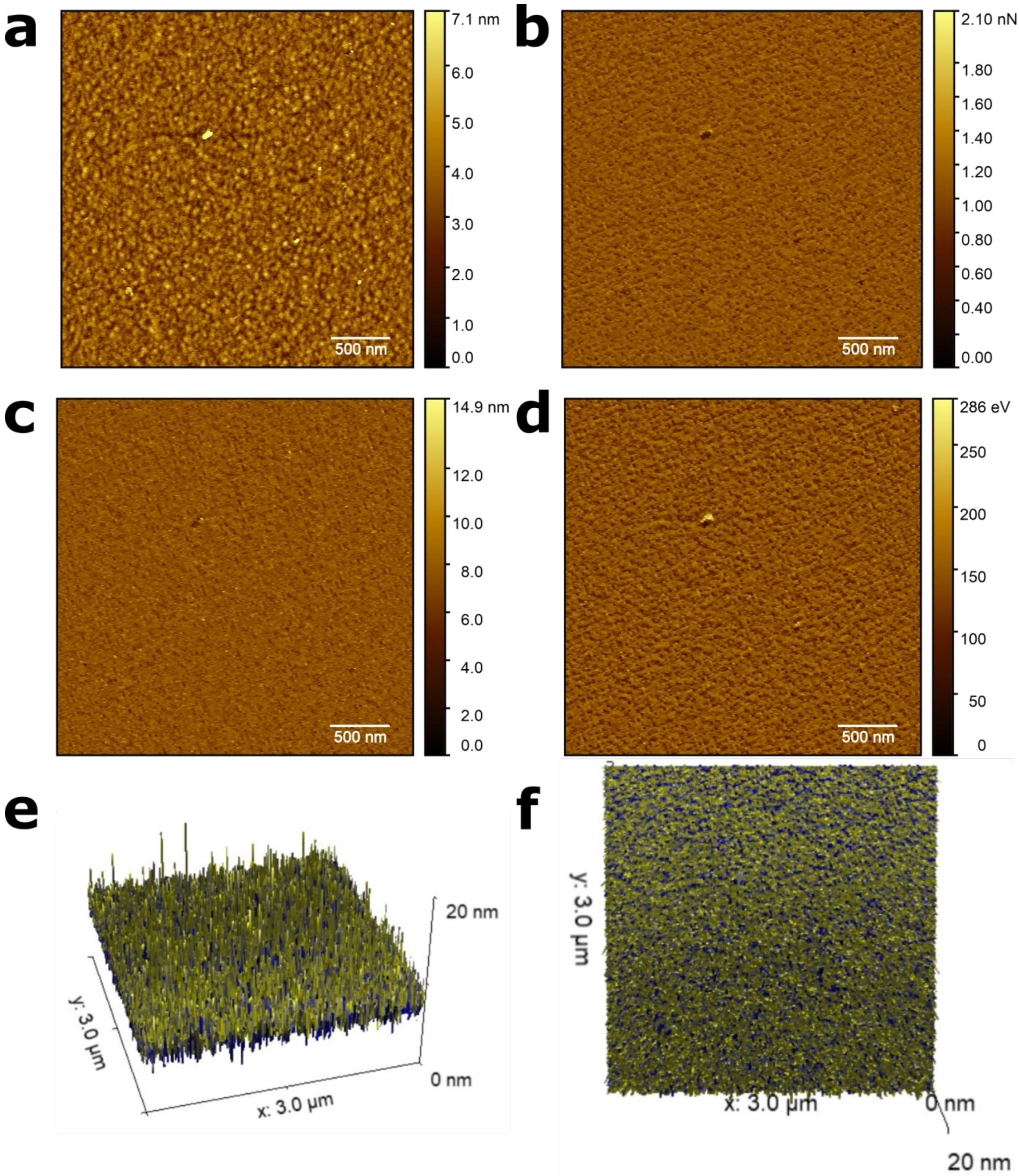

Figure S9: AD4 type 2. (a-d) topography, adhesion, deformation, and dissipation, respectively. (e,f) Correlation of Deformation and Dissipation channel. Two assembly types are observed: type 1 with dominant hard features and type two, which has an increased prevalence of elastic features at a ratio of 4:7. This is indicative of the role of dilution is enforcing a face-on orientation of helicene.

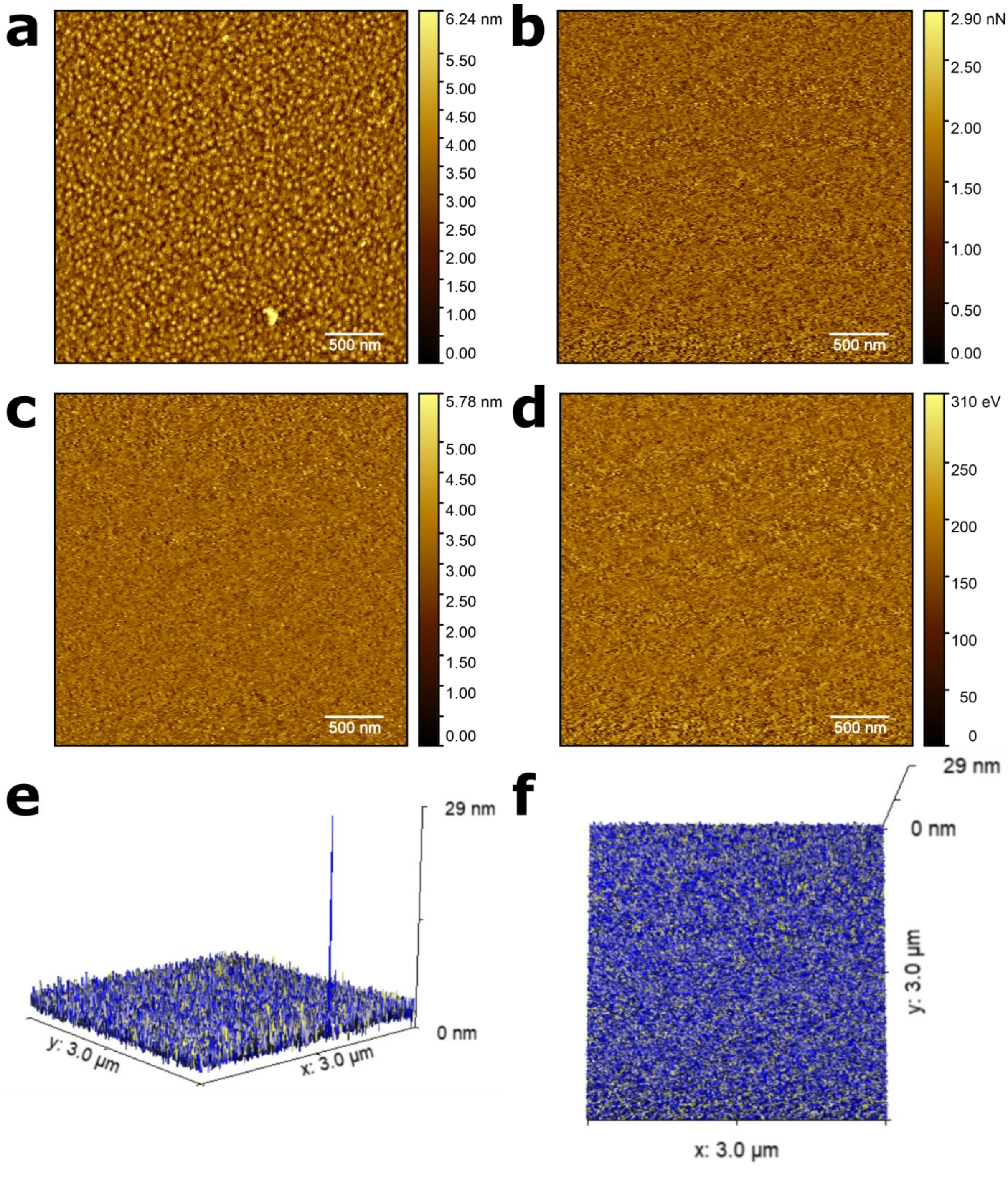

Figure S10: AD5 (a-d) topography, adhesion, deformation, and dissipation, respectively. (e,f) Correlation of Deformation and Dissipation channel. The highly dilute, toluene free solution results in a hard-to-elastic ratio similar to AD1 type 1, i.e. prevalence of hard features at a 8:5 ratio. This suggests that the face-on orientation is a kinetic, rather than a thermodynamic product of deposition.

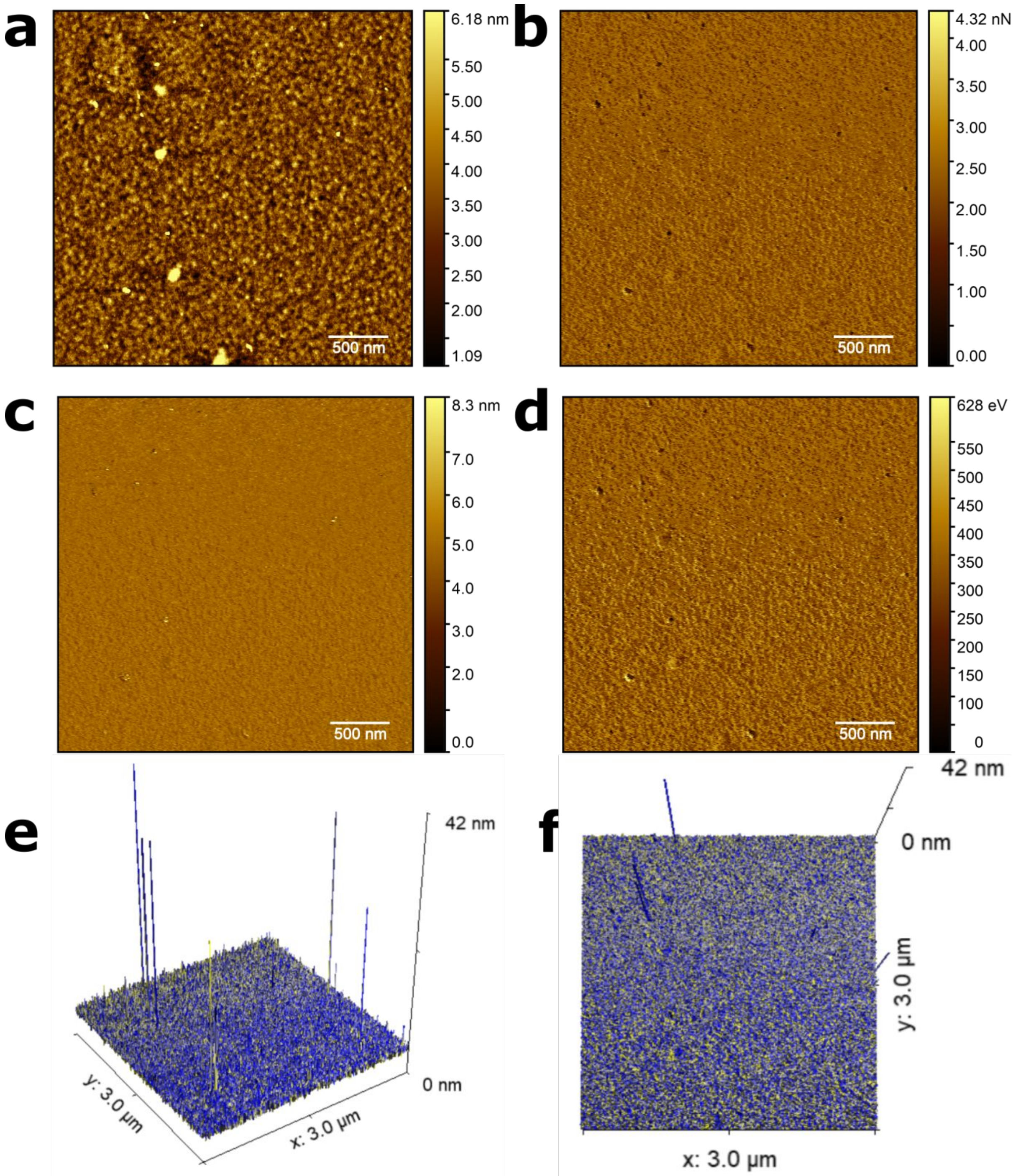

Figure S11: AD6 (a-d) topography, adhesion, deformation, and dissipation, respectively. (e,f) Correlation of Deformation and Dissipation channel. Strong elastic features are seen (blue peaks), with multiple elastic features piercing the landscape at lower deformations. The extremely dilute toluene content drove the formation of face-on oriented helicene; the ratio of hard-to-elastic features is 3:9 of percent of surface area and thus the only case of prevalent elastic features in any AD sample.

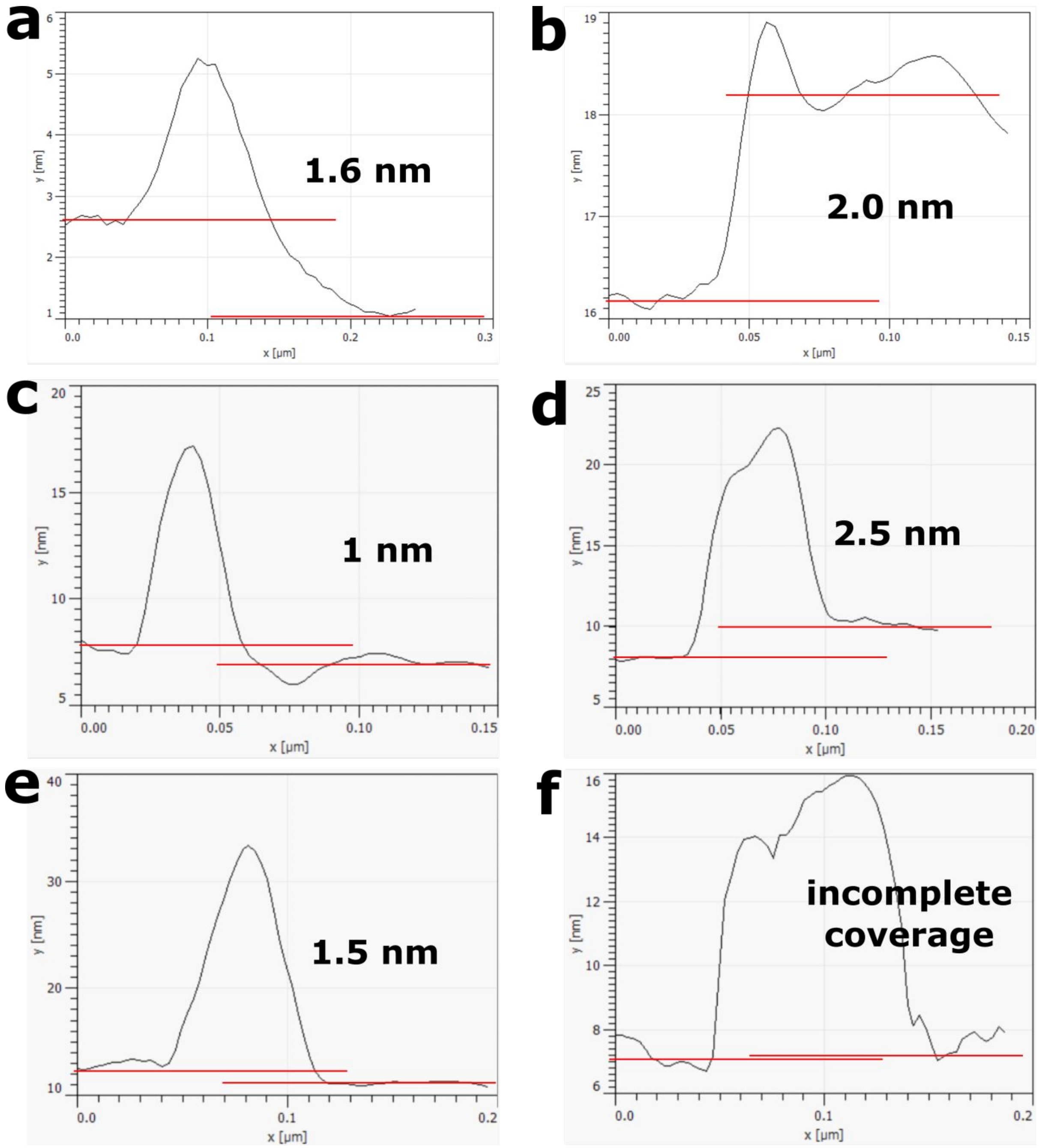

Figure S12: Profiles of scratched surfaces used to determine thickness/coverage by AD-SAM. Red lines as a guide for the eye.

PH-SAM

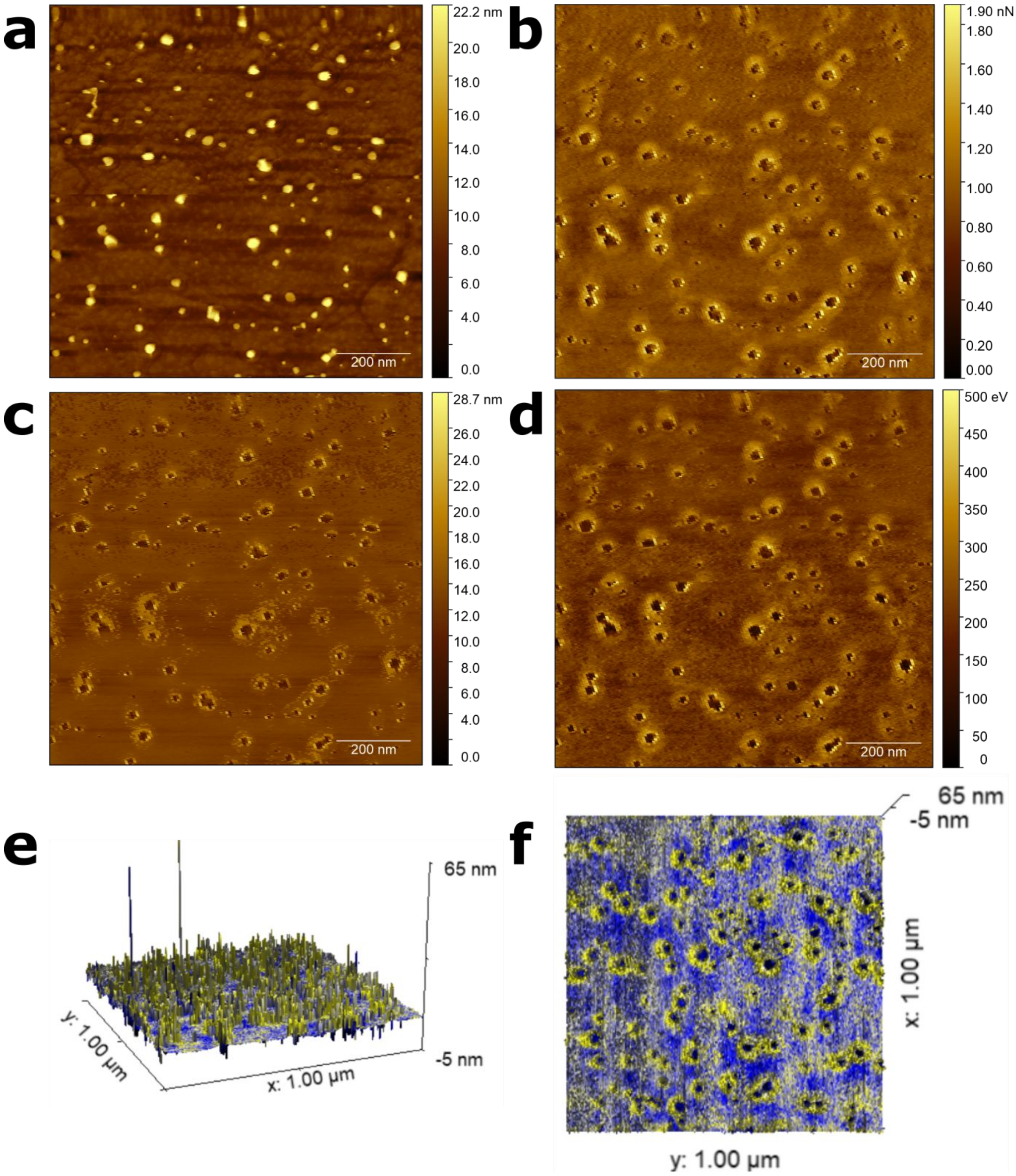

Figure S13: PH1 (a-d) topography, adhesion, deformation, and dissipation, respectively. (e,f) Correlation of Deformation and Dissipation channel. Topographic features correspond to hard features of edge-on oriented material which dominate the surface. Each hard granule is surrounded by plastically deforming, disordered material.

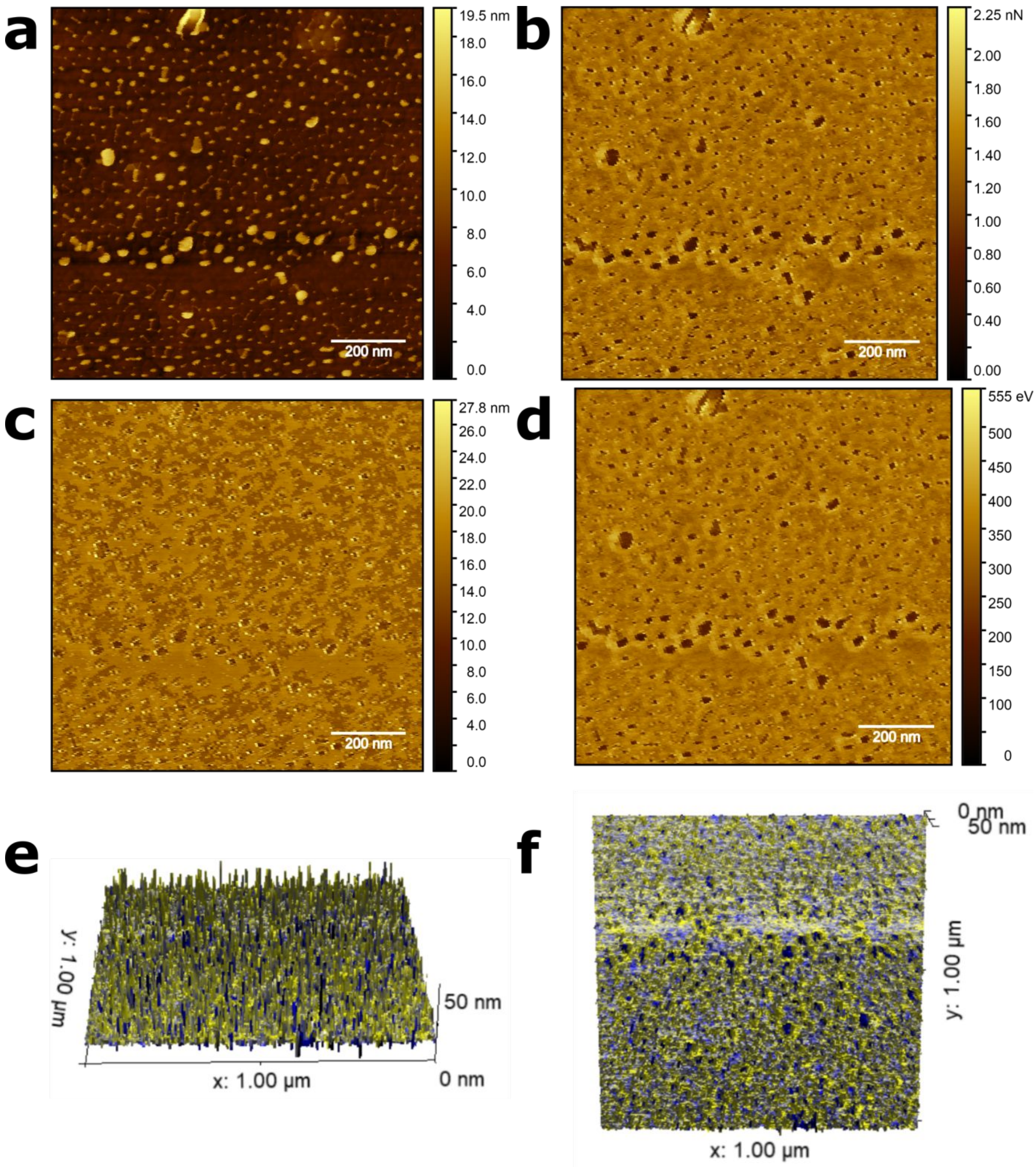

Figure S14: PH2 (a-d) topography, adhesion, deformation, and dissipation, respectively. (e,f) Correlation of Deformation and Dissipation channel. Again, hard features dominate, and are sourounded by a small layer of elastically deforming material at a 12:2 ratio, indicative of dominant edge-on orientation.

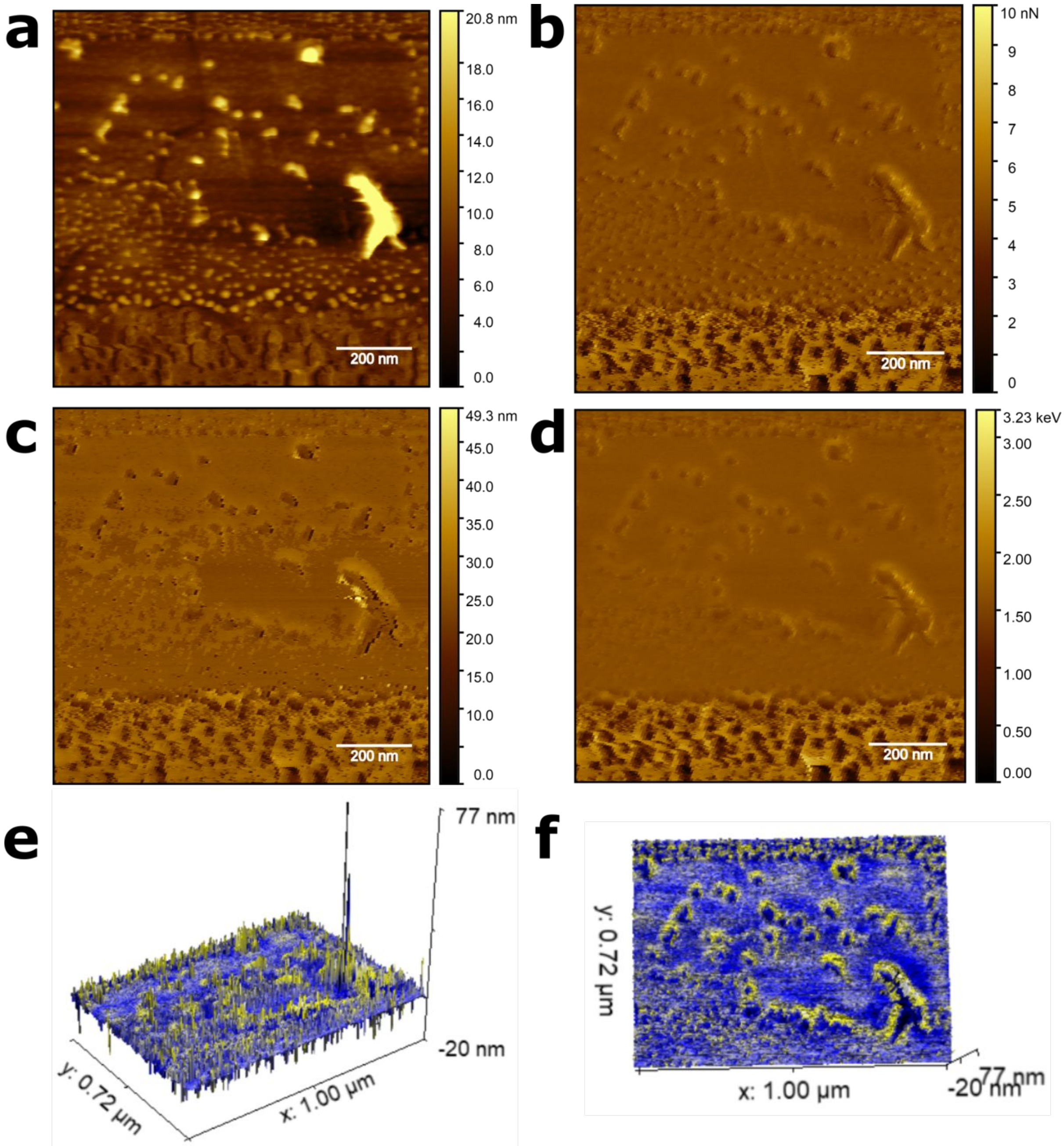

Figure S15: PH3 tip debris (a-d) topography, adhesion, deformation, and dissipation, respectively. (e,f) Correlation of Deformation and Dissipation channel. Similar to PH2, dominant topographic features are hard and surrounded by plastically-deforming material in its vicinity (agglomeration formed during deposition). Elastic deformation (blue peaks, face-on orientation) is inexistent and the surface is dominated by edge-on deposited helicene.

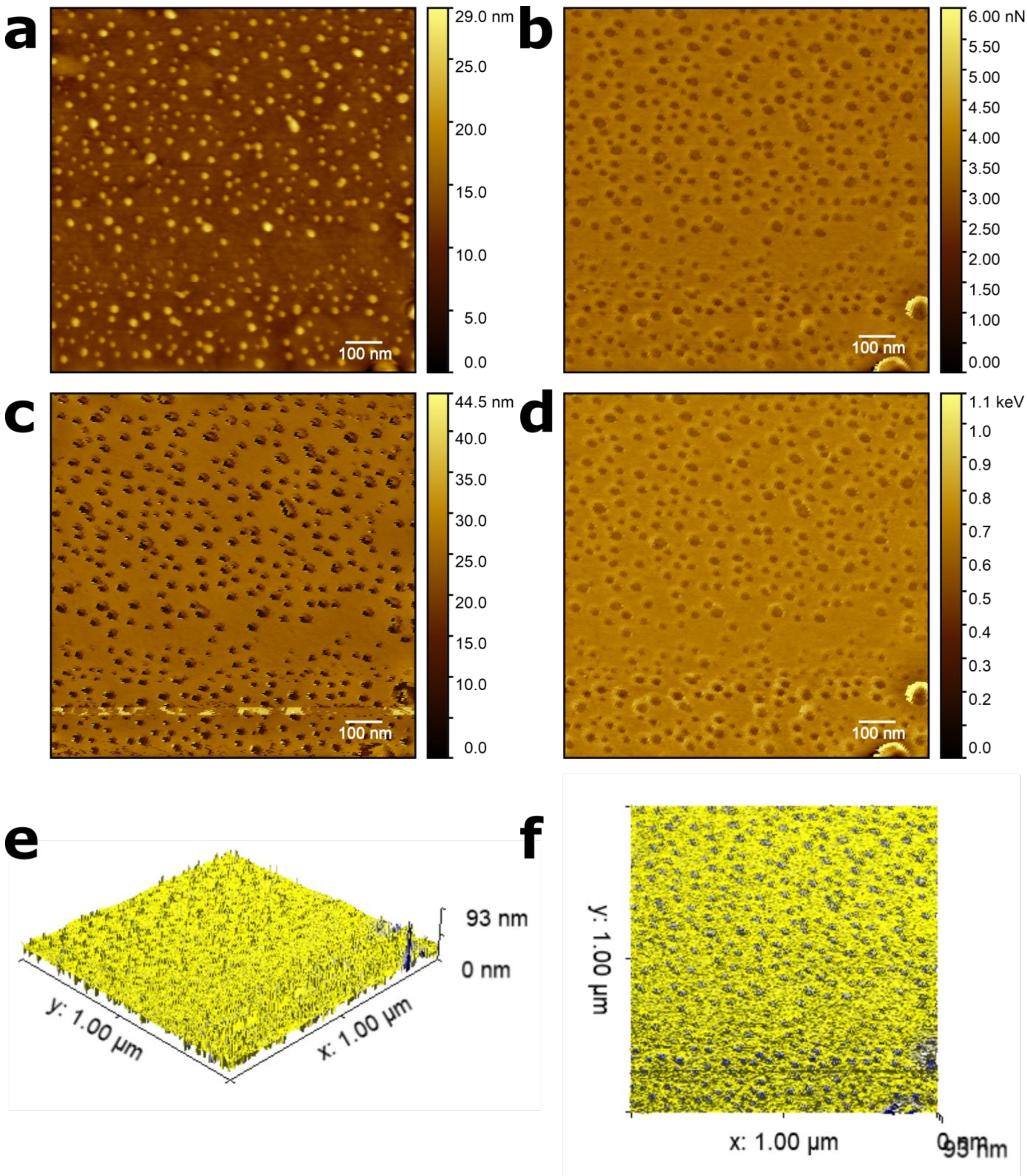

Figure S16: PH3 type 2. (a-d) topography, adhesion, deformation, and dissipation, respectively. (e,f) Correlation of Deformation and Dissipation channel. Again, highly similar results are observed with edge-on material being the dominant feature. Both assembly types are dominated by hard features- type 1 generally softer and less homogenous.

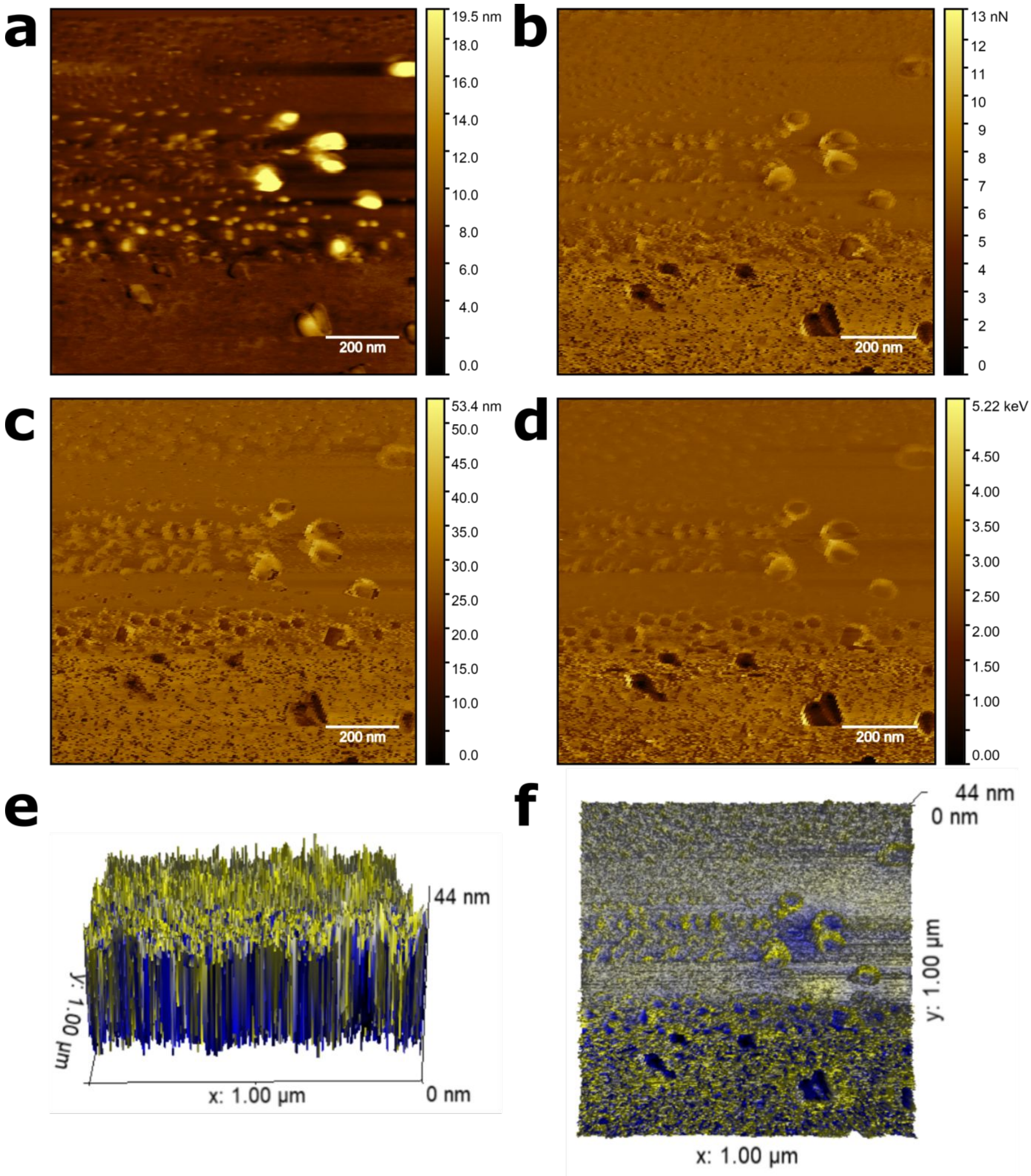

Figure S17: PH4 tip-debris, type 1 (a-d) topography, adhesion, deformation, and dissipation, respectively. (e,f) Correlation of Deformation and Dissipation channel. Hard features dominate the image, indicative of edge-on deposition. All hard features are surrounded by plastically deforming material.

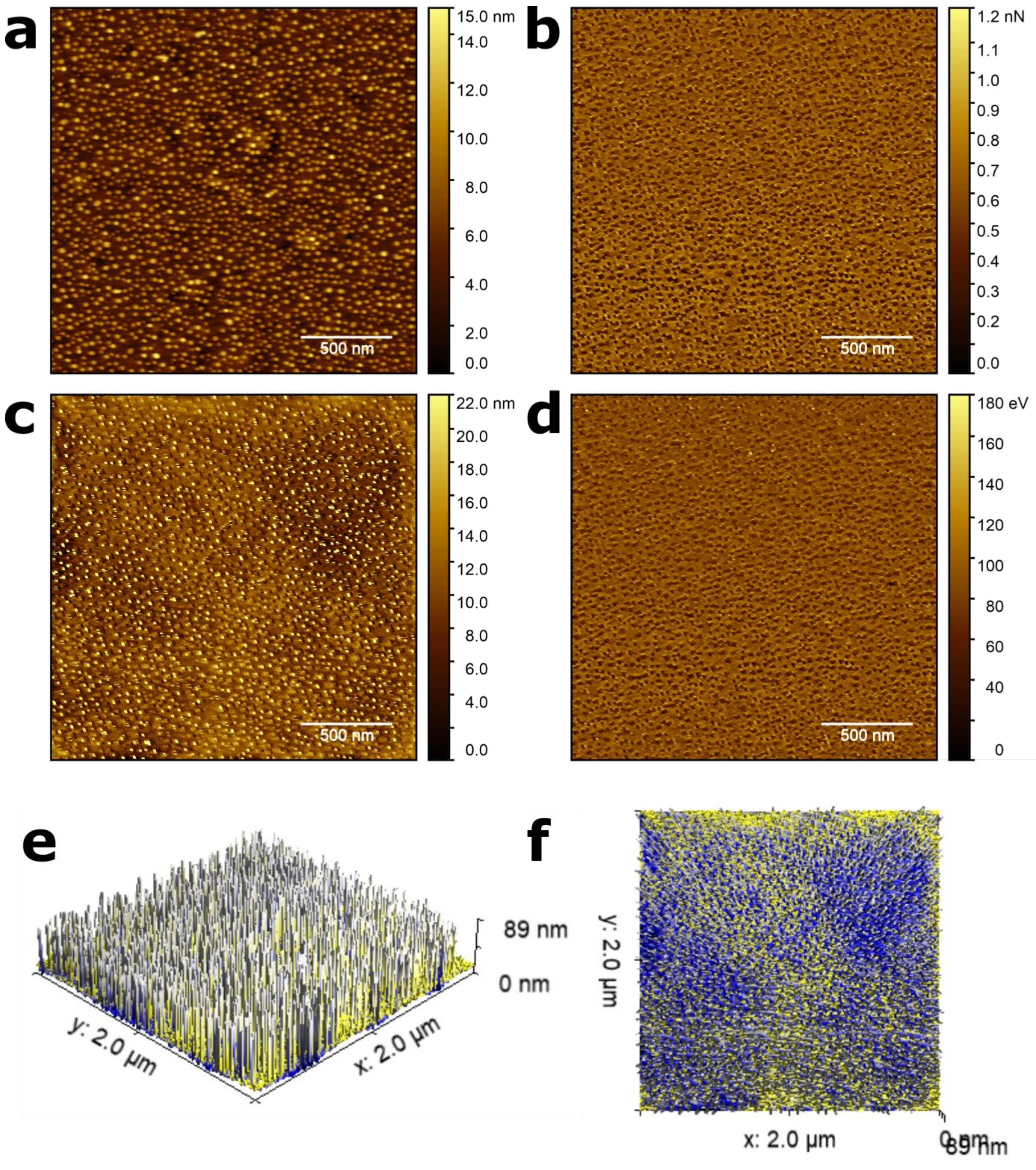

Figure S18: PH4 type 2. (a-d) topography, adhesion, deformation, and dissipation, respectively. (e,f) Correlation of Deformation and Dissipation channel. These surfaces are dominated by high-deformation material deposited around hard, edge-on oriented helicene. More than 6% of the surface is covered in face-on oriented material which deforms elastically.

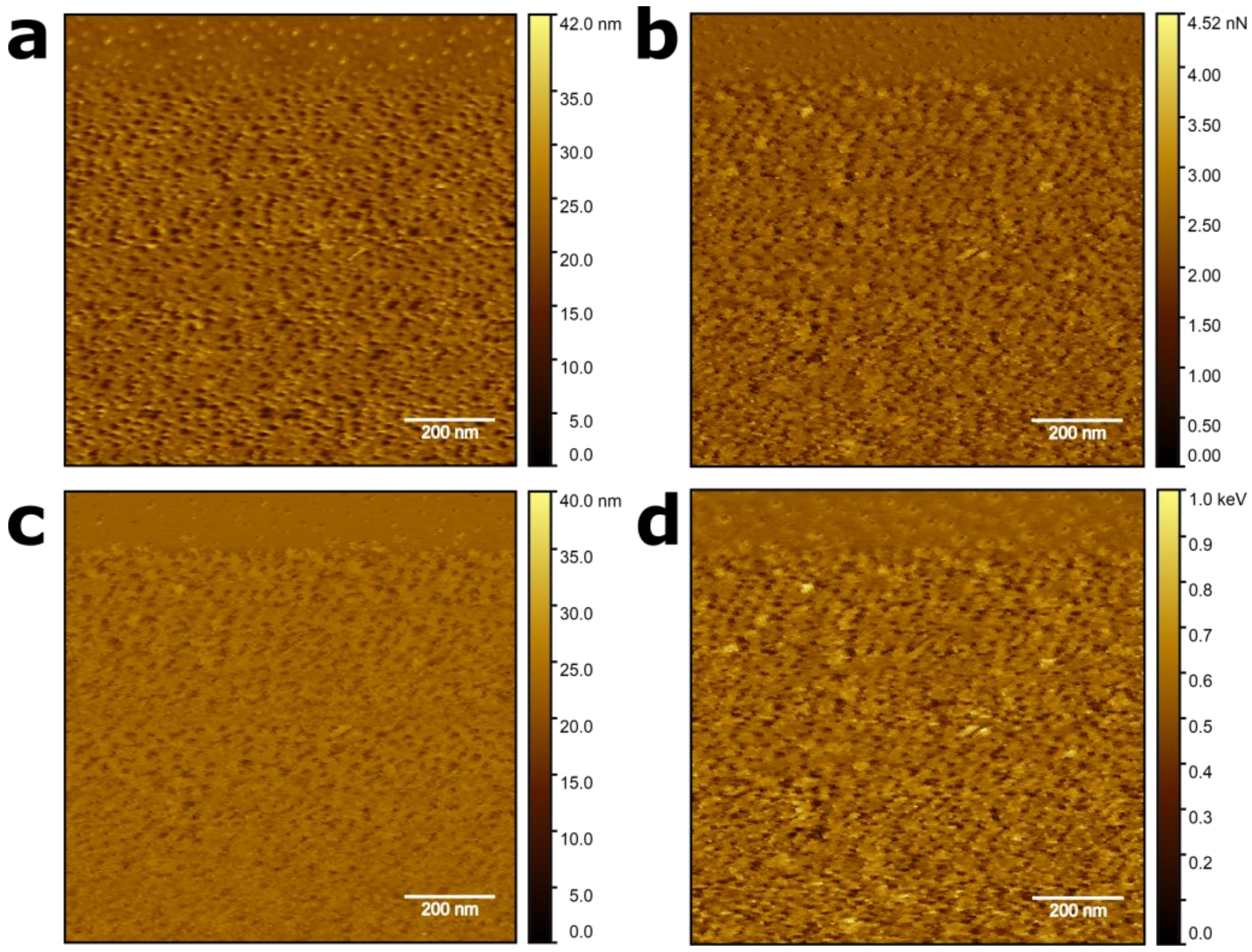

Figure S19: PH4 - Example of tip change. (a-d) topography, adhesion, deformation, and dissipation, respectively.

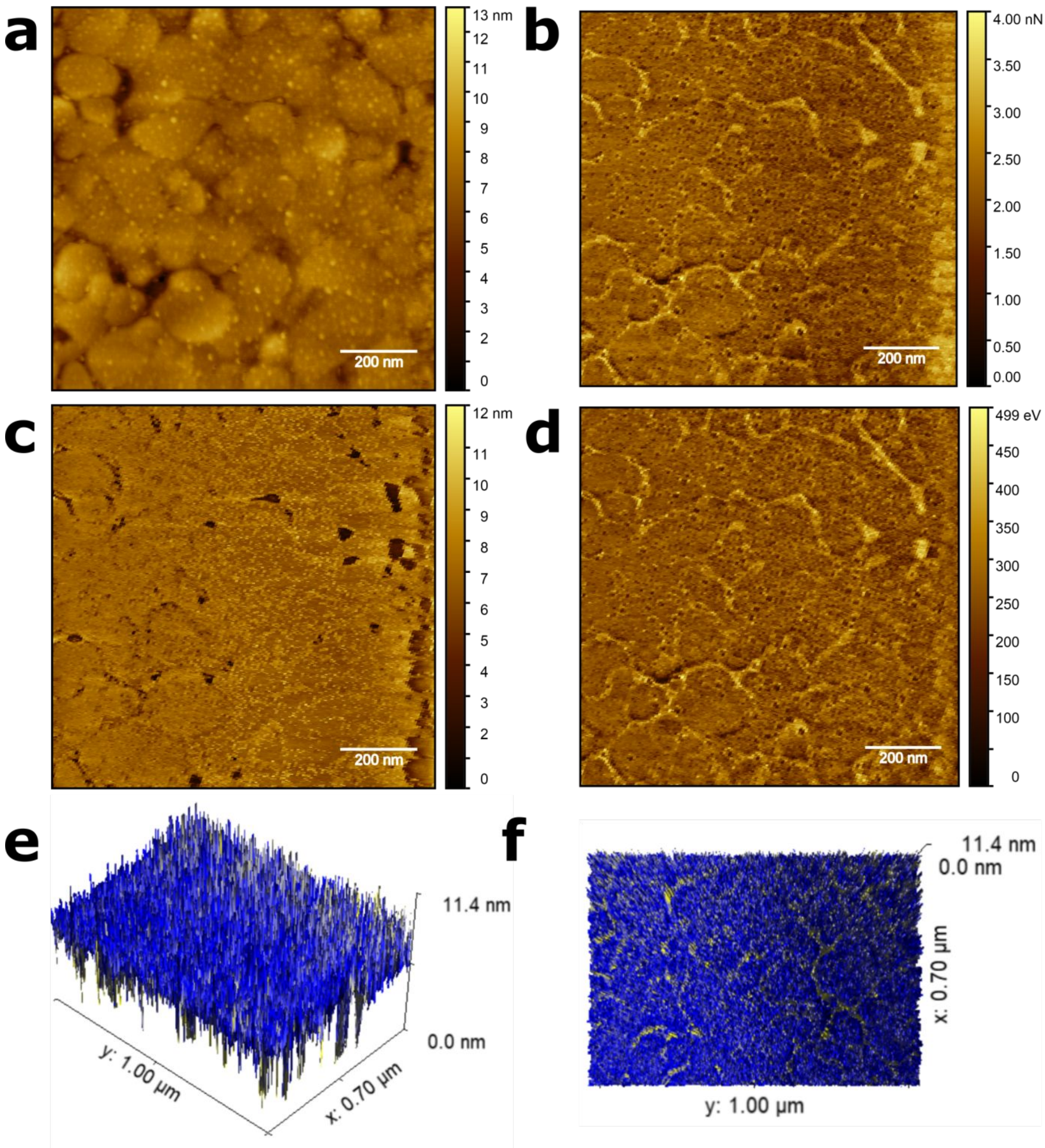

Figure S20: PH5 (a-d) topography, adhesion, deformation, and dissipation, respectively. (e,f) Correlation of Deformation and Dissipation channel. The surfaces show a realative absence of distinct plastic deformation, while hard and elastic features dominate at a 8:6 ratio. This indicates, that at these low concentration levels, both edge- and face-on orientations are present on the former is only slightly favoured.

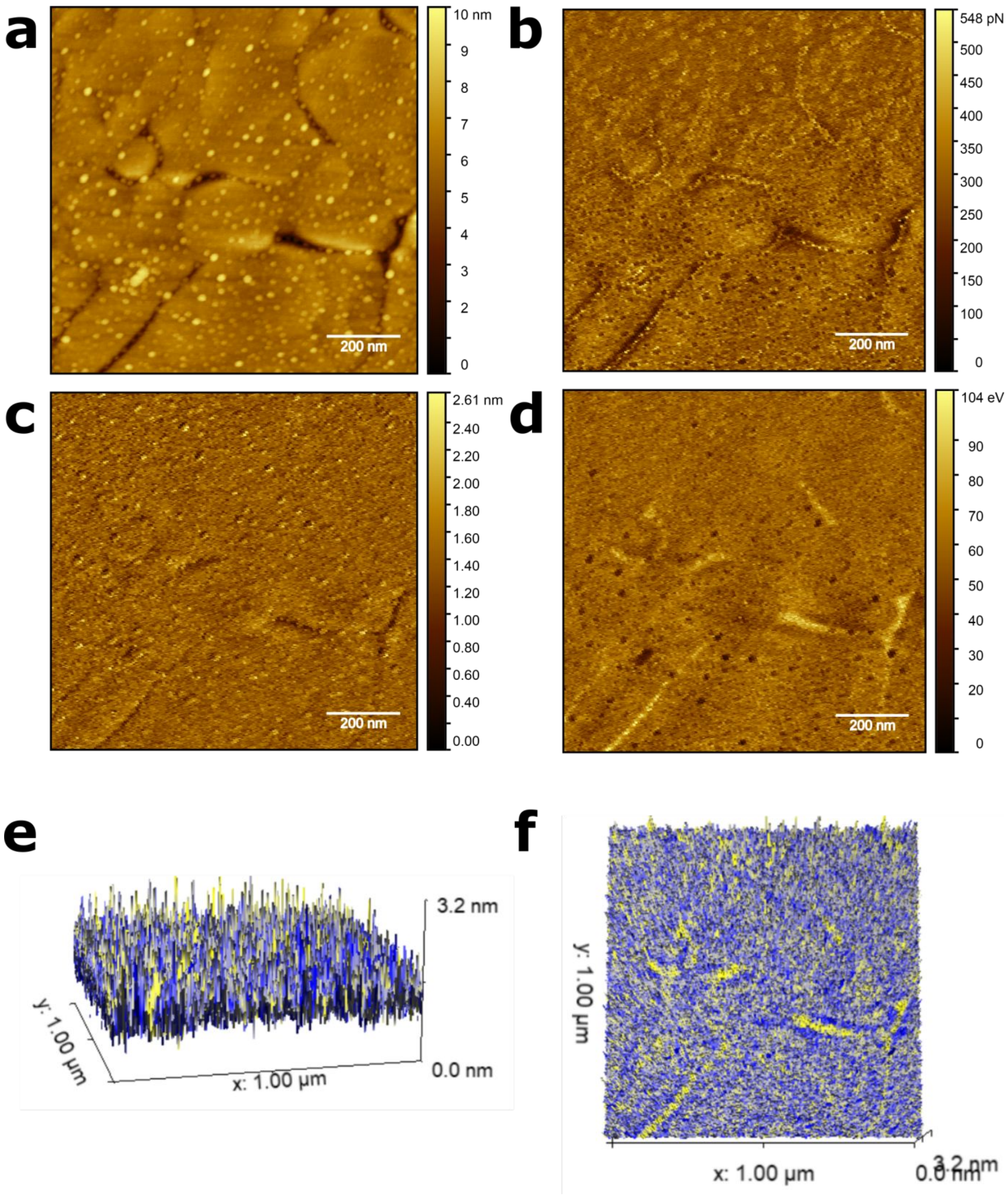

Figure S21: PH6 (a-d) topography, adhesion, deformation, and dissipation, respectively. (e,f) Correlation of Deformation and Dissipation channel. The surface is incompletely covered with most if not all helicene consolidated into the tall features (Fig.S21e). A unified assembly type similar to PH5 was observed, *i.e.* dilution leads to and increased presence of elastic features.

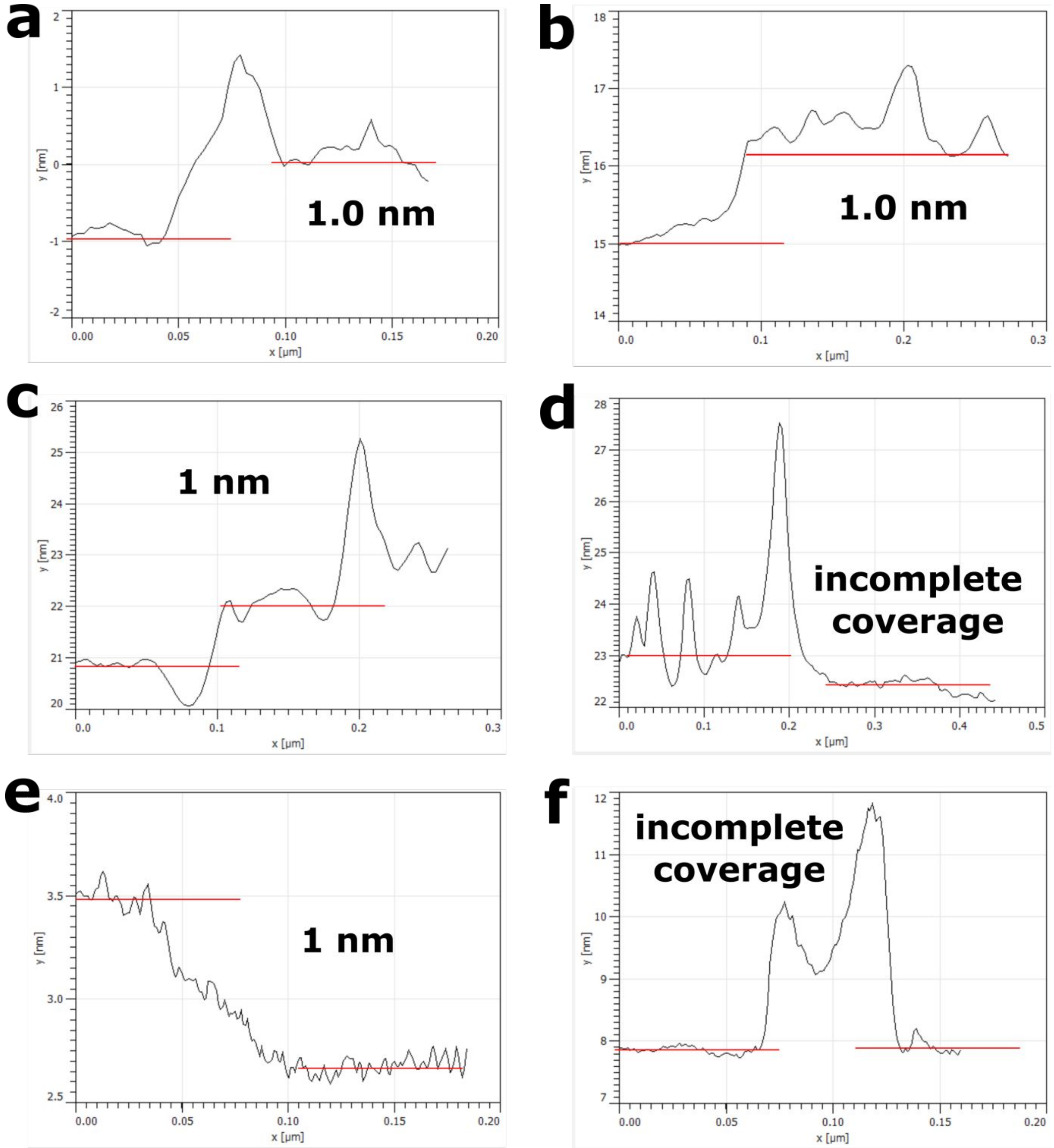

Figure S22: PH-SAM thicknesses. Red lines as a guide for the eye.

TRT-SAM: P-helicene (enantiomer 1)

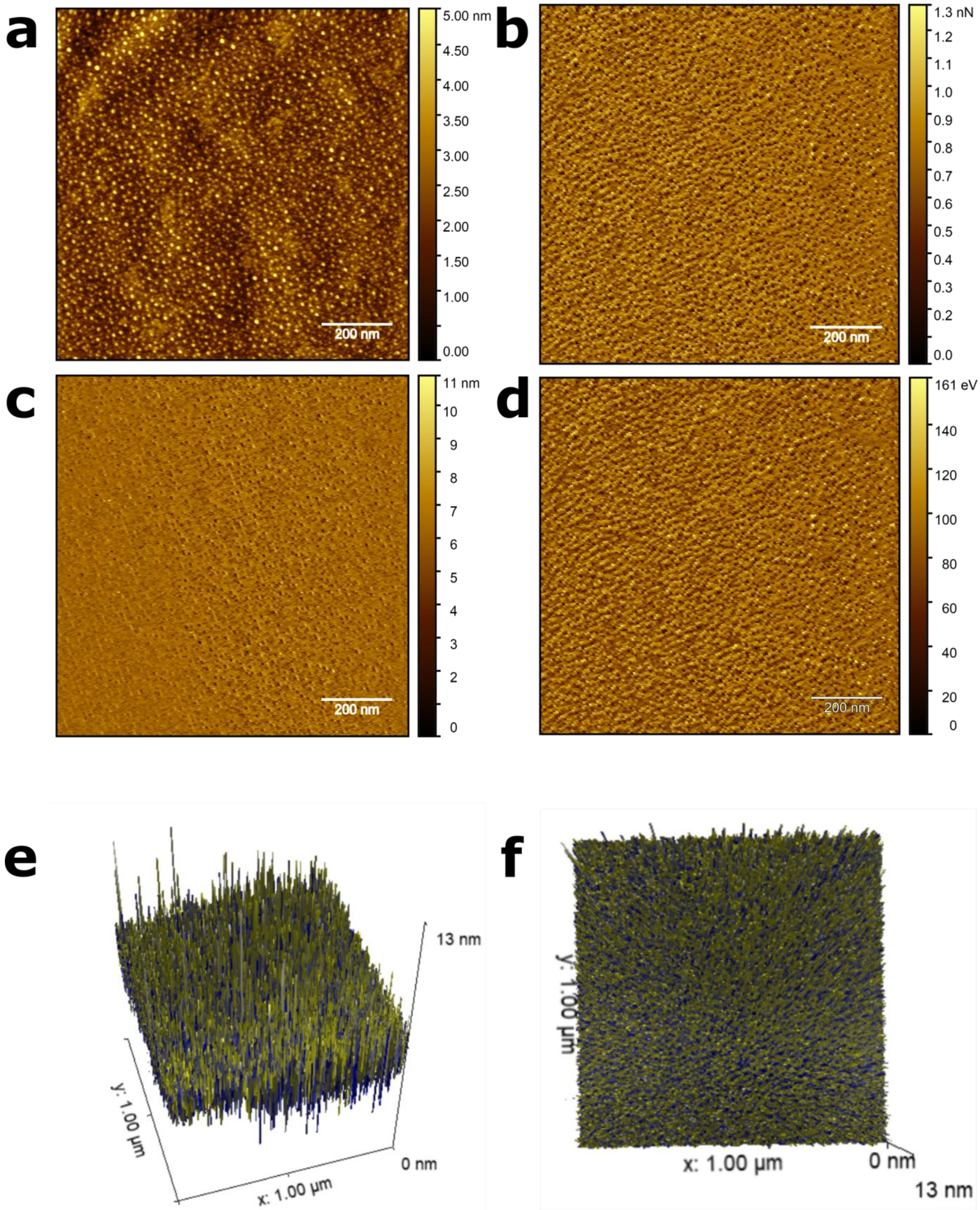

Figure S23: Trt1-P. (a-d) topography, adhesion, deformation, and dissipation, respectively. (e,f) Correlation of Deformation and Dissipation Channel. Plastic deformation dominates the surfaces (yellow peaks) and are interrupted by both hard and elastic features at a 10:4 ratio.

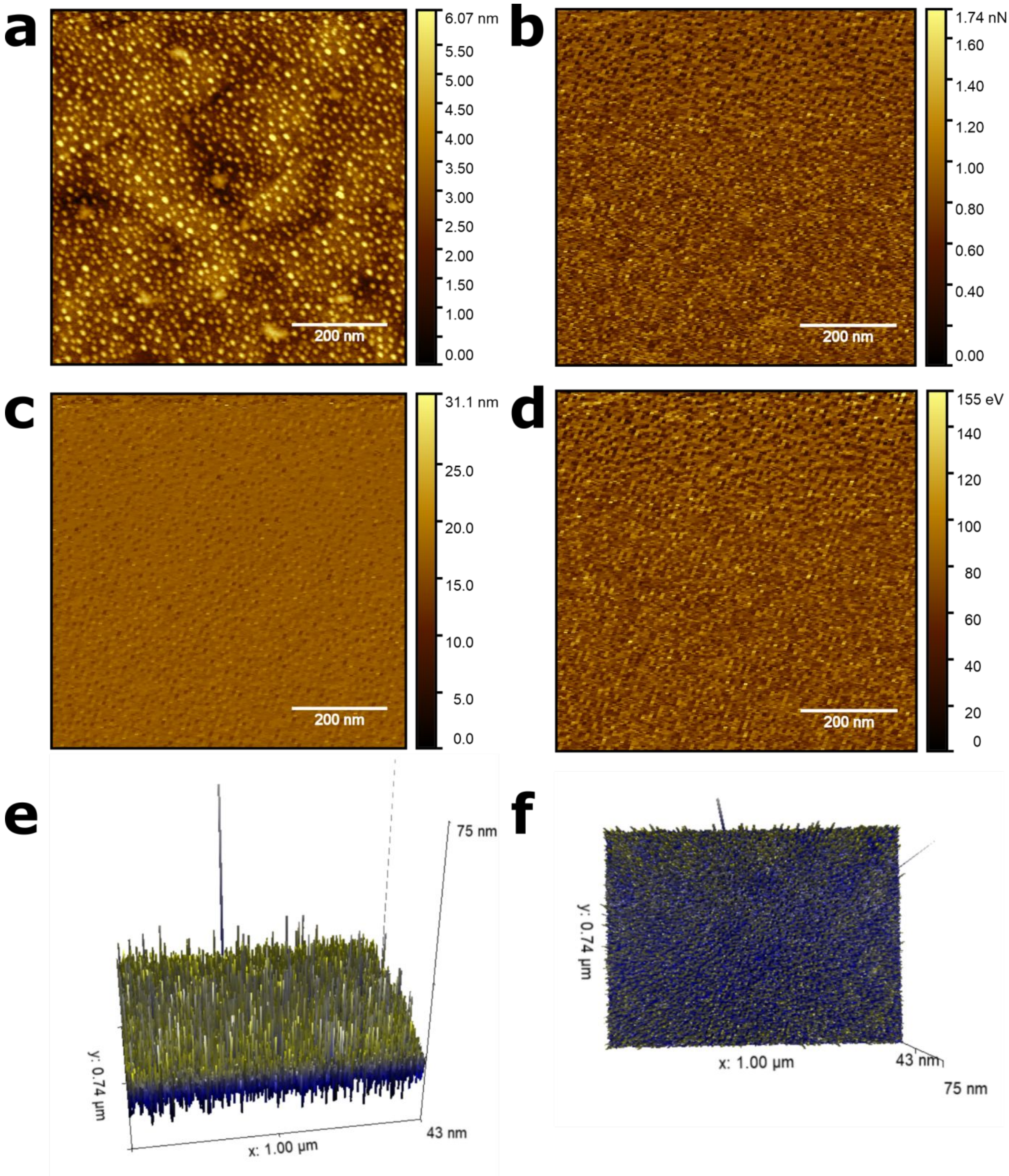

Figure S24: Trt2-P (a-d) topography, adhesion, deformation, and dissipation, respectively. (e,f) Correlation of Deformation and Dissipation Channel. The surfaces are highly similar to those prepared in the absence of toluene. Plastic deformation dominates but is interrupted by hard features and weaker elastic deformation-signals (10:4 ratio). Edge-on orientation is dominant.

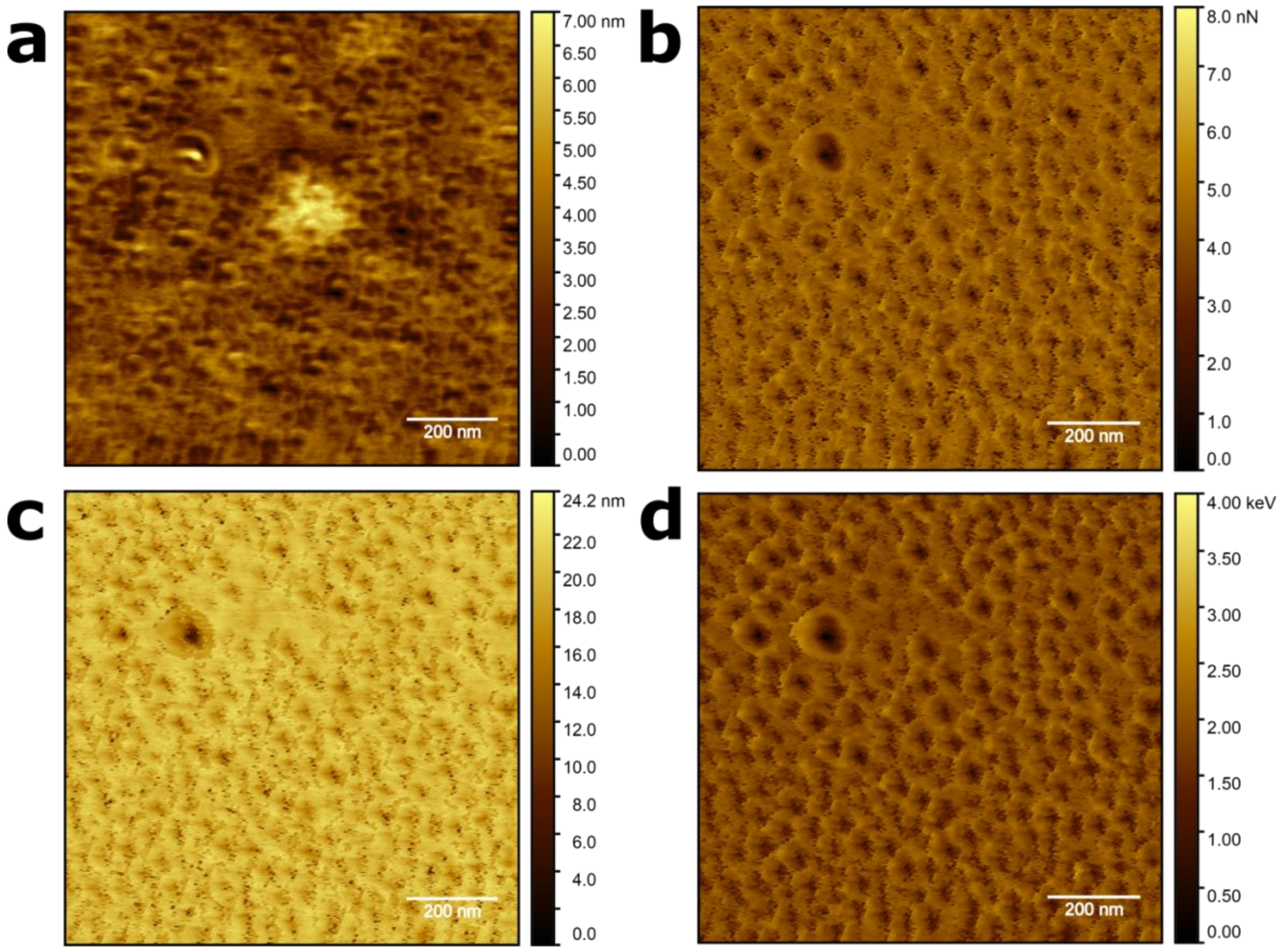

Figure S25: Trt3-P, type1 dewetting – four molecules high, possibly face-on poor inter-molecular cohesion, relatively strong adhesion to tip. (a-d) topography, adhesion, deformation, and dissipation, respectively. This assembly-type was observed in a single batch of samples.

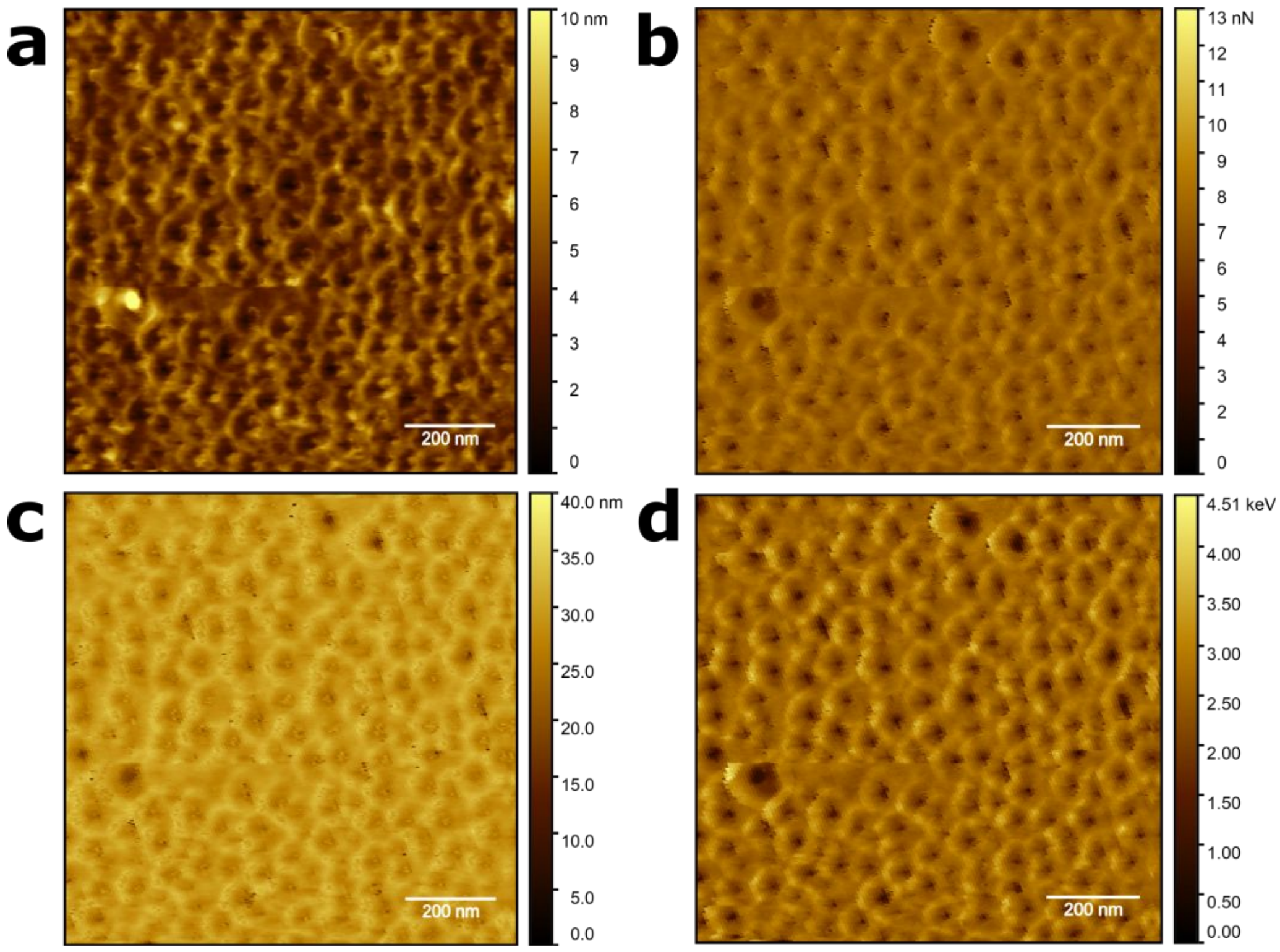

Figure S26: Trt4-P type 1 – dewetting. Seven molecules high stacks, fifteen wide, poor inter-molecular cohesion, relatively strong adhesion to tip. (a-d) topography, adhesion, deformation, and dissipation, respectively. This assembly-type was observed in a single batch of samples.

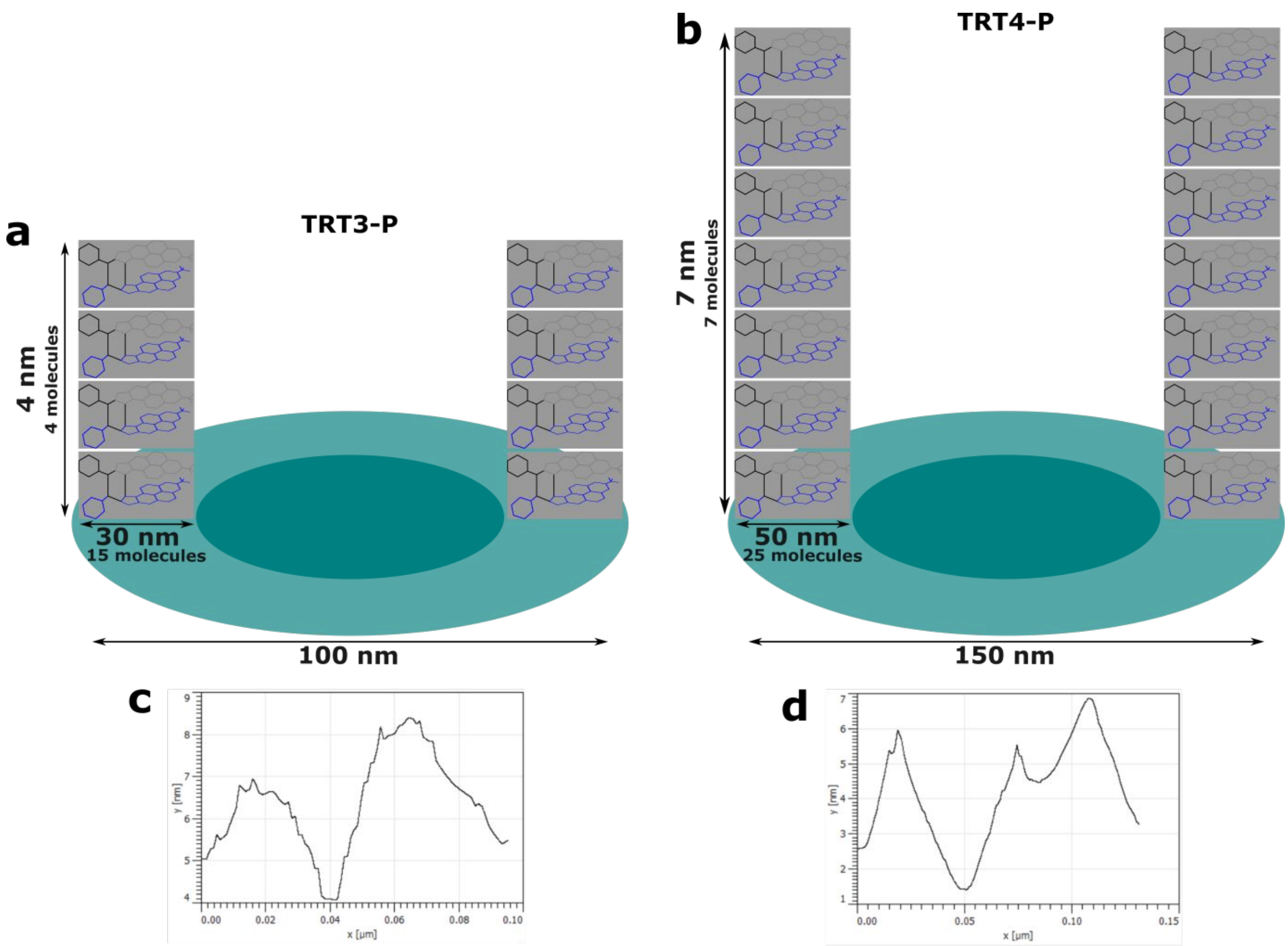

Figure S27: Dewetting process observed for the P-enantiomer. (a) Illustration of assemblies on TRT3 and (b) TRT4, with (c,d) their respective line-scans. This assembly-type was observed in a single batch of samples. As the trityl-group is not intrinsically chiral and need an impulse to adapt a predominant chirality, we suggest that these have been caused by chiral gold-features emerging during the flame-annealing process.[5]

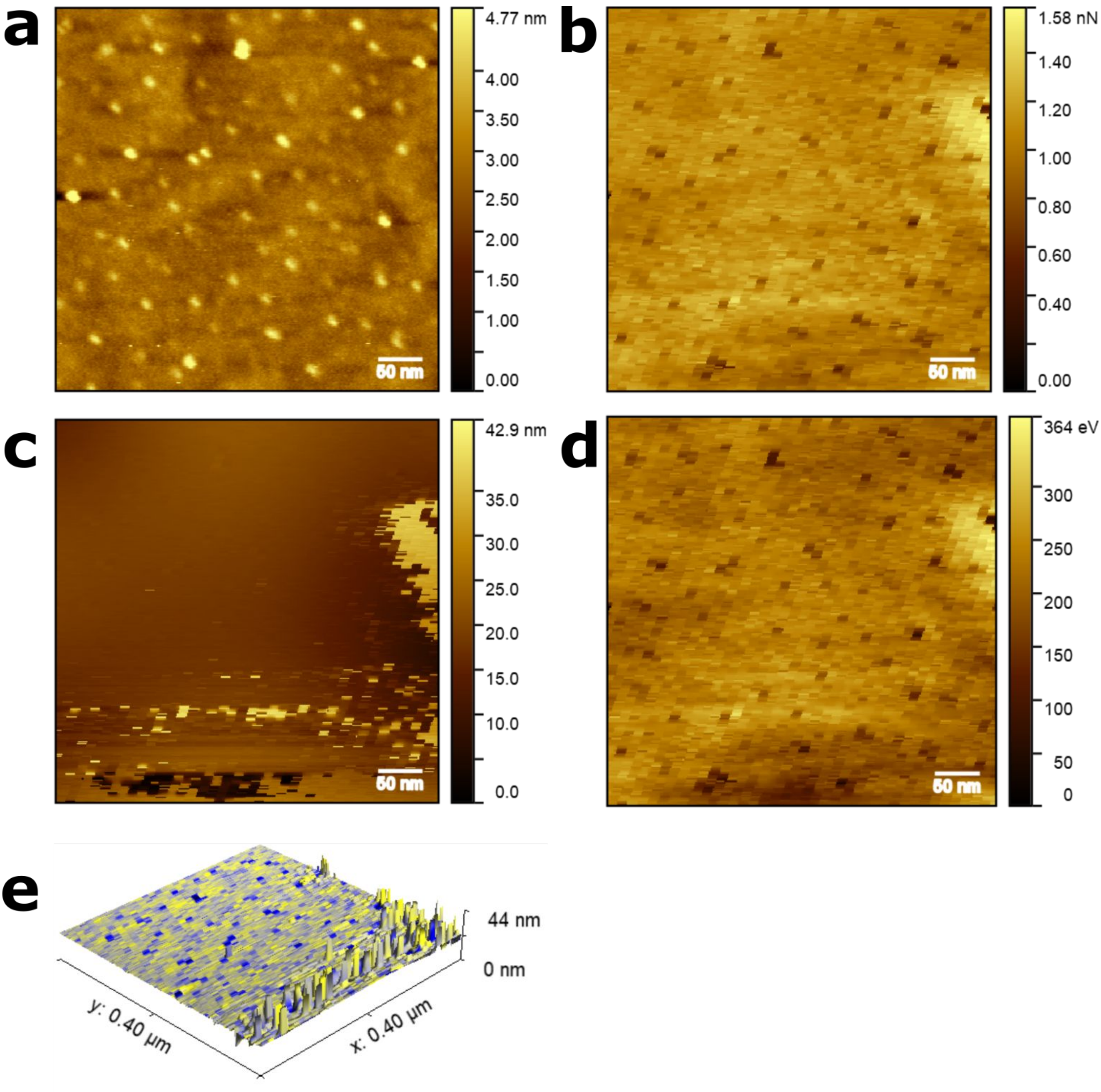

Figure S28: Trt3-P type 2 (no dewetting, but low quality resolution). (a-d) topography, adhesion, deformation, and dissipation, respectively. (e) Correlation of Deformation and Dissipation Channel. These samples were significantly more elastic with an increase with hard and elastic features making up 8% of the surface area.

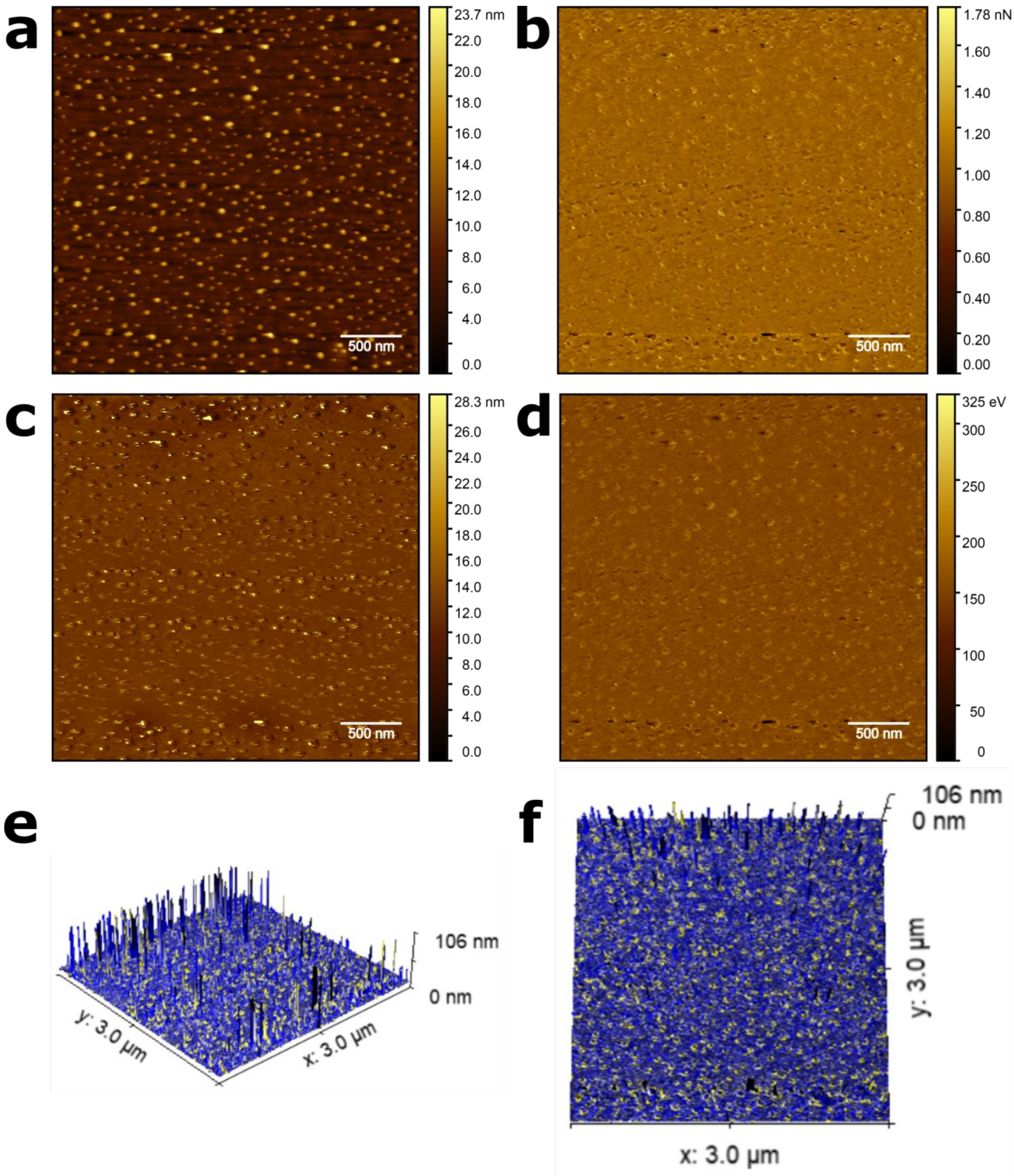

Figure S29: Trt4-P type 2 survey. Absence of dewetting in all regions of the sample. (a-d) topography, adhesion, deformation, and dissipation, respectively. (e,f) Correlation of Deformation and Dissipation channel. A significant increase of elastic features was observed, with a ratio of 4:11 in favour of elastic deformation (face-on helicene dominant). The yellow features herein indicate adhesive patches on teh surface rather than plastically deforming ones with a layer thickness of 1.6 nm and 15 nm tall helicene formations (Fig.S29d).

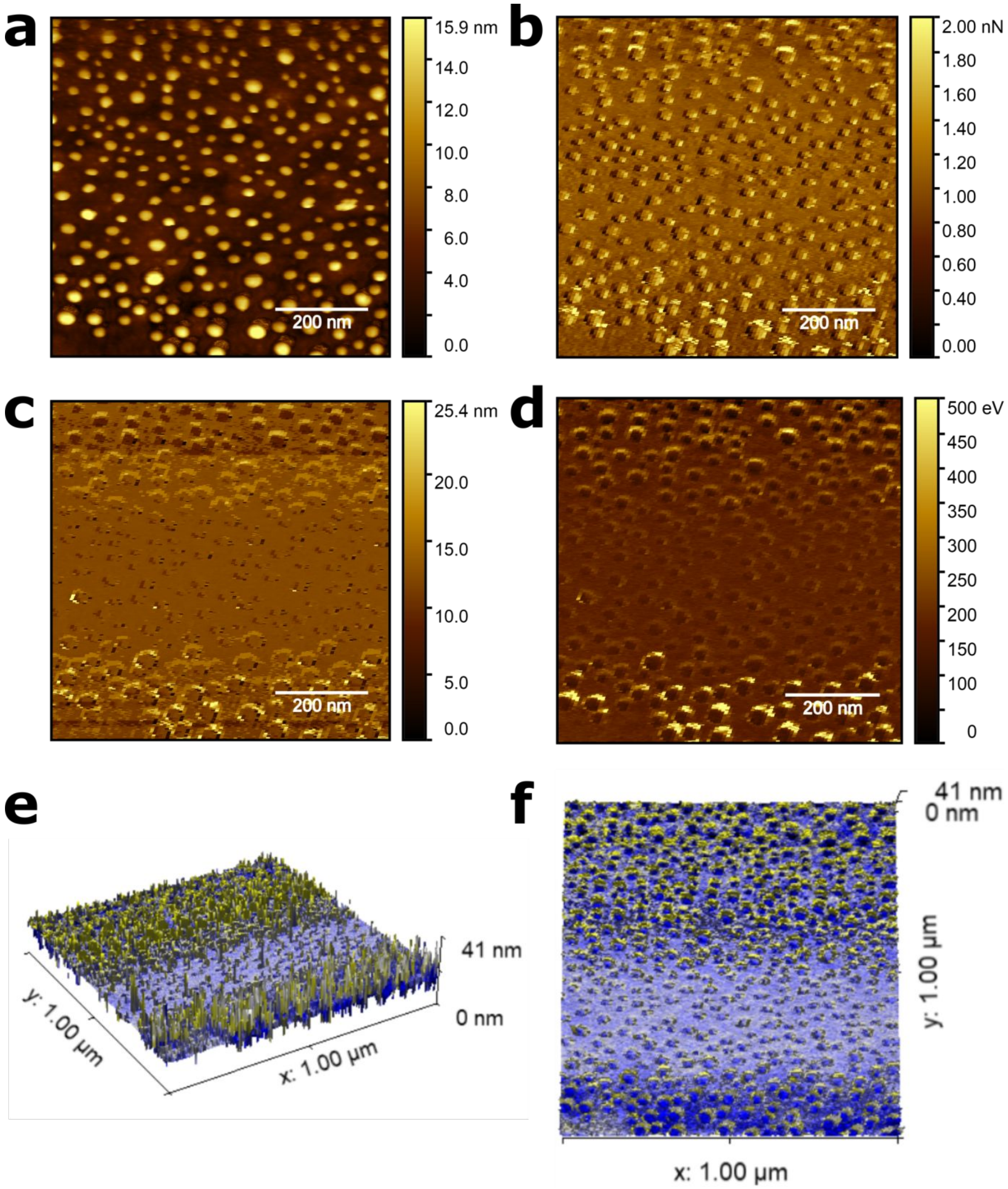

Figure S30: Trt5-P (a-d) topography, adhesion, deformation, and dissipation, respectively. (e,f) Correlation of Deformation and Dissipation Channel. The high dilution has the opposite effect than what we observed for AD and PH surfaces. Observed features are dominantly hard (thus edge-on, 17:3 ratio) in nature and surrounded by poorly cohesive, plastically deforming material.

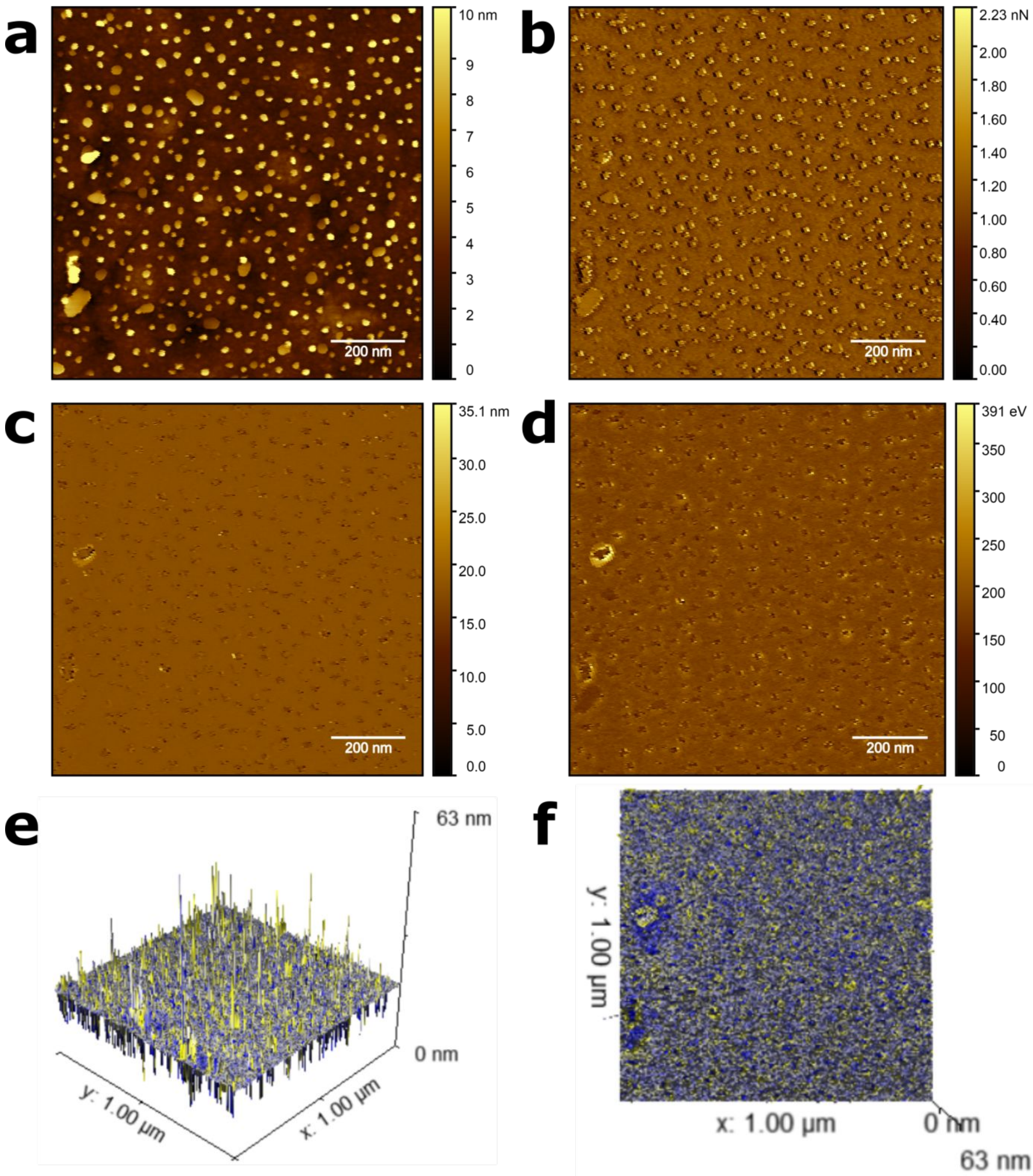

Figure S31: Trt6-P (a-d) topography, adhesion, deformation, and dissipation, respectively. (e,f) Correlation of Deformation and Dissipation Channel. Similar to TRT5-P hard, edge-on oriented material is dominant, yet traces of toluene improve the existence of elastic, face-on oriented material (12:4 ratio). Again, plastic deformation was detected in the viscinity of topographically significant features.

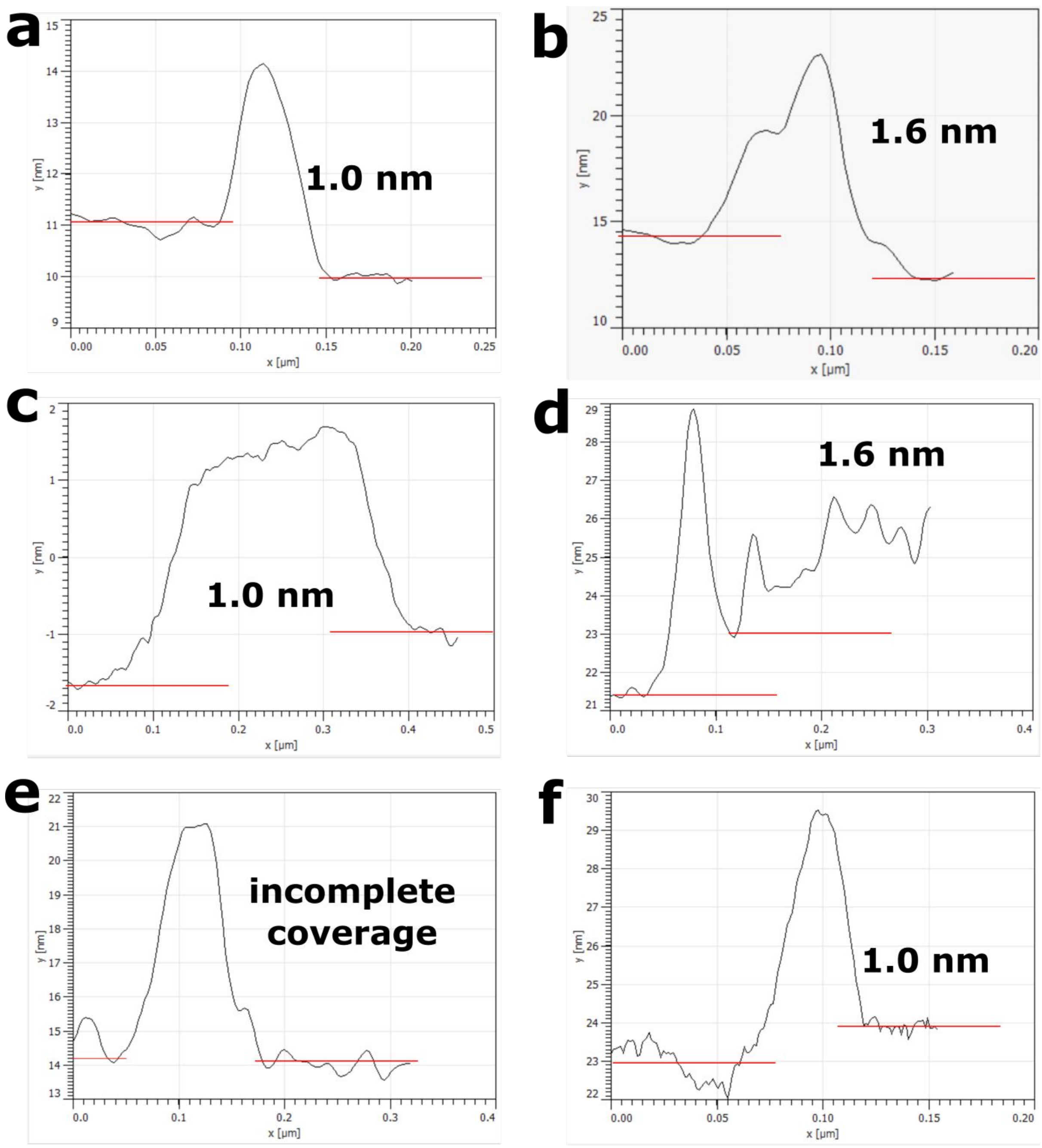

Figure S32: TRT-P thicknesses (P-enantiomer). Red lines as a guide for the eye.

TRT-SAM: M-helicene

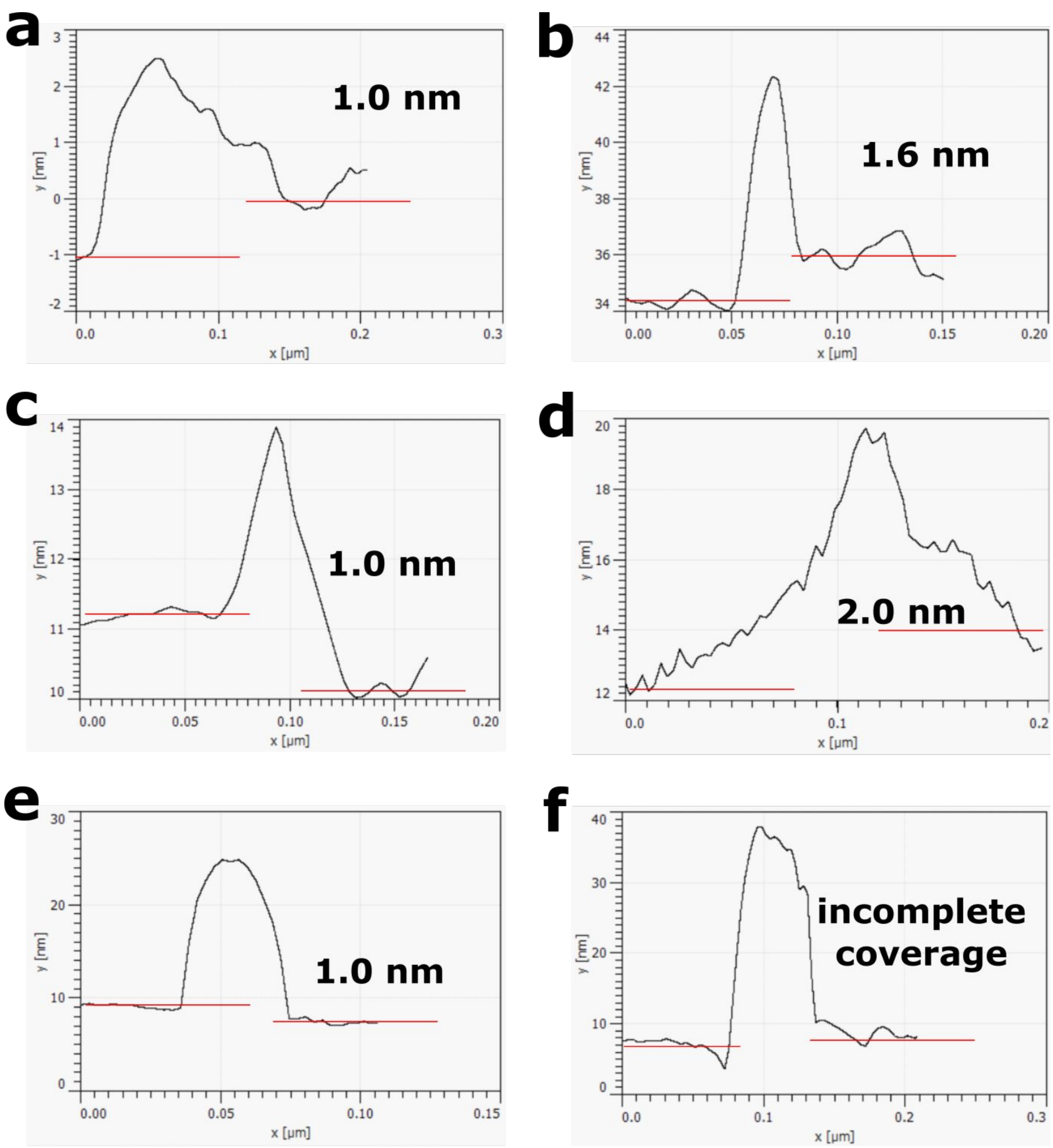

Figure S33: TRT-M thicknesses (M-enantiomer, E2). Red lines as a guide for the eye.

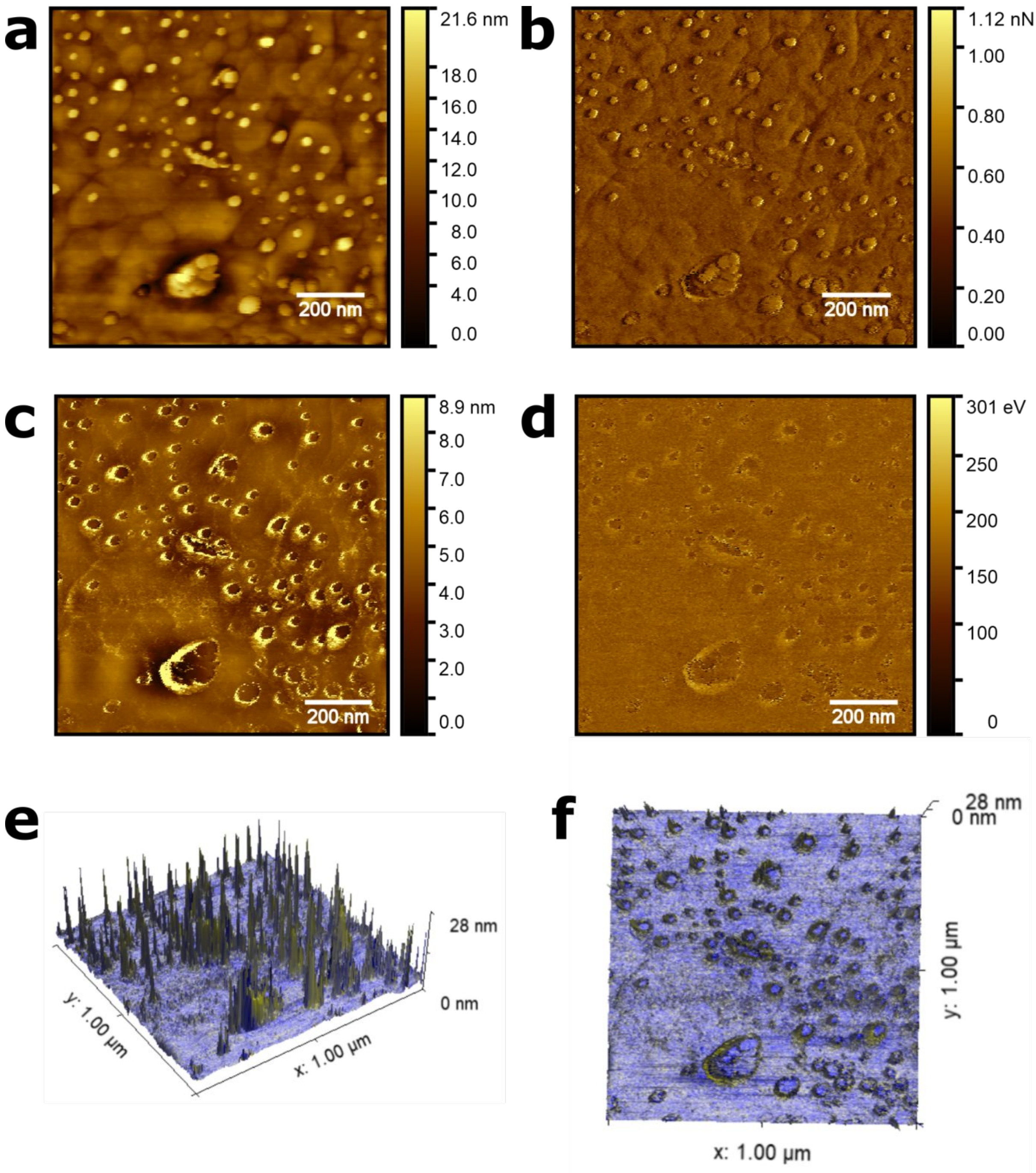

Figure S34: TRT1-M type 1. (a-d) topography, adhesion, deformation, and dissipation, respectively. (e,f) Correlation of Deformation and Dissipation Channel. This surface is dominated by hard features surrounded by plastically deforming material with a hard-to-elastic ratio of 8:5 surface area%. Oddly enough, similar hardness was observed in topological features and the surface, indicative of overall edge-on deposition.

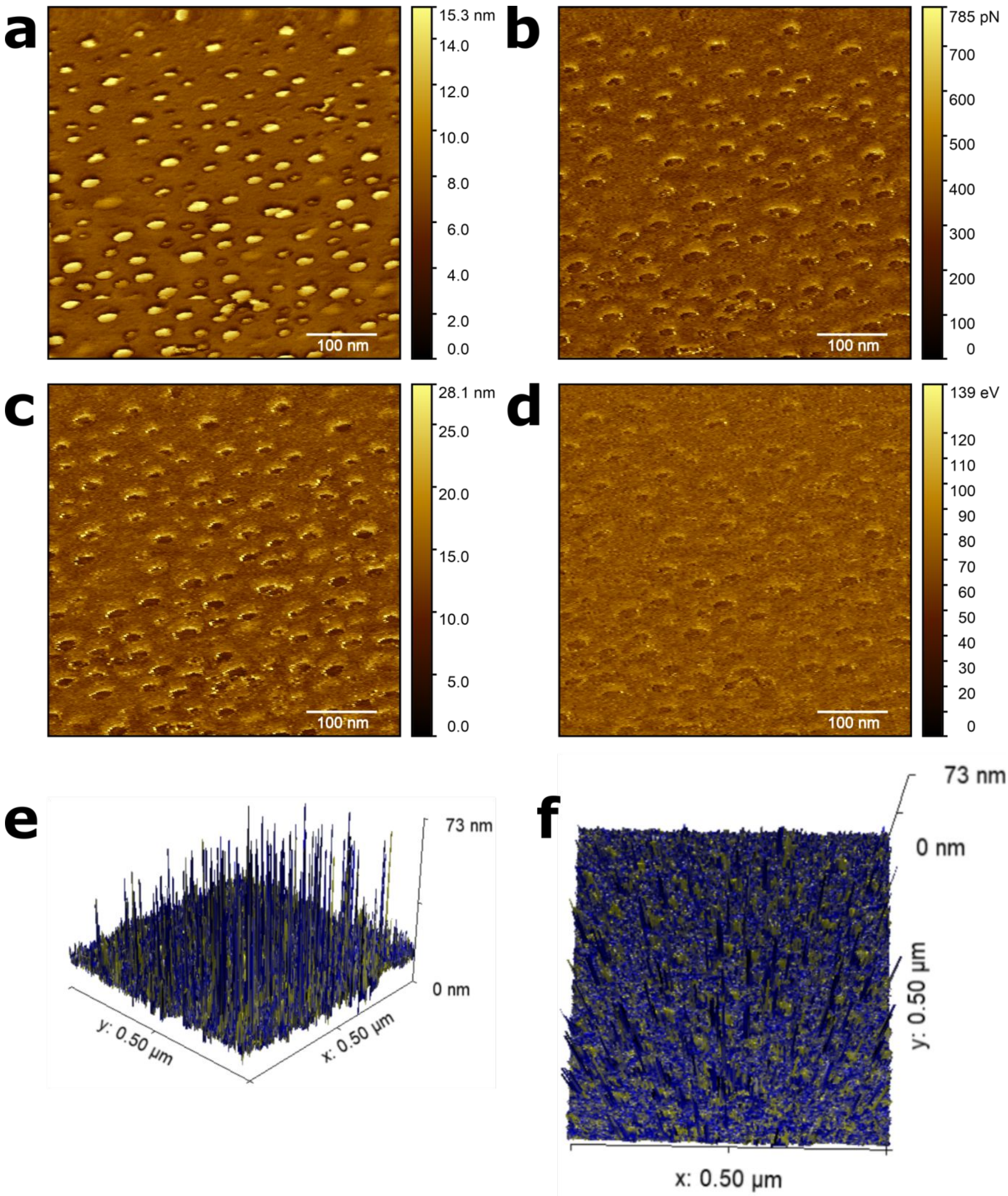

Figure S35: Trt2-M (a-d) topography, adhesion, deformation, and dissipation, respectively. (e,f) Correlation of Deformation and Dissipation Channel. A single deposition type was detected with hard and elastic features coexisting at a 7:8 ratio.

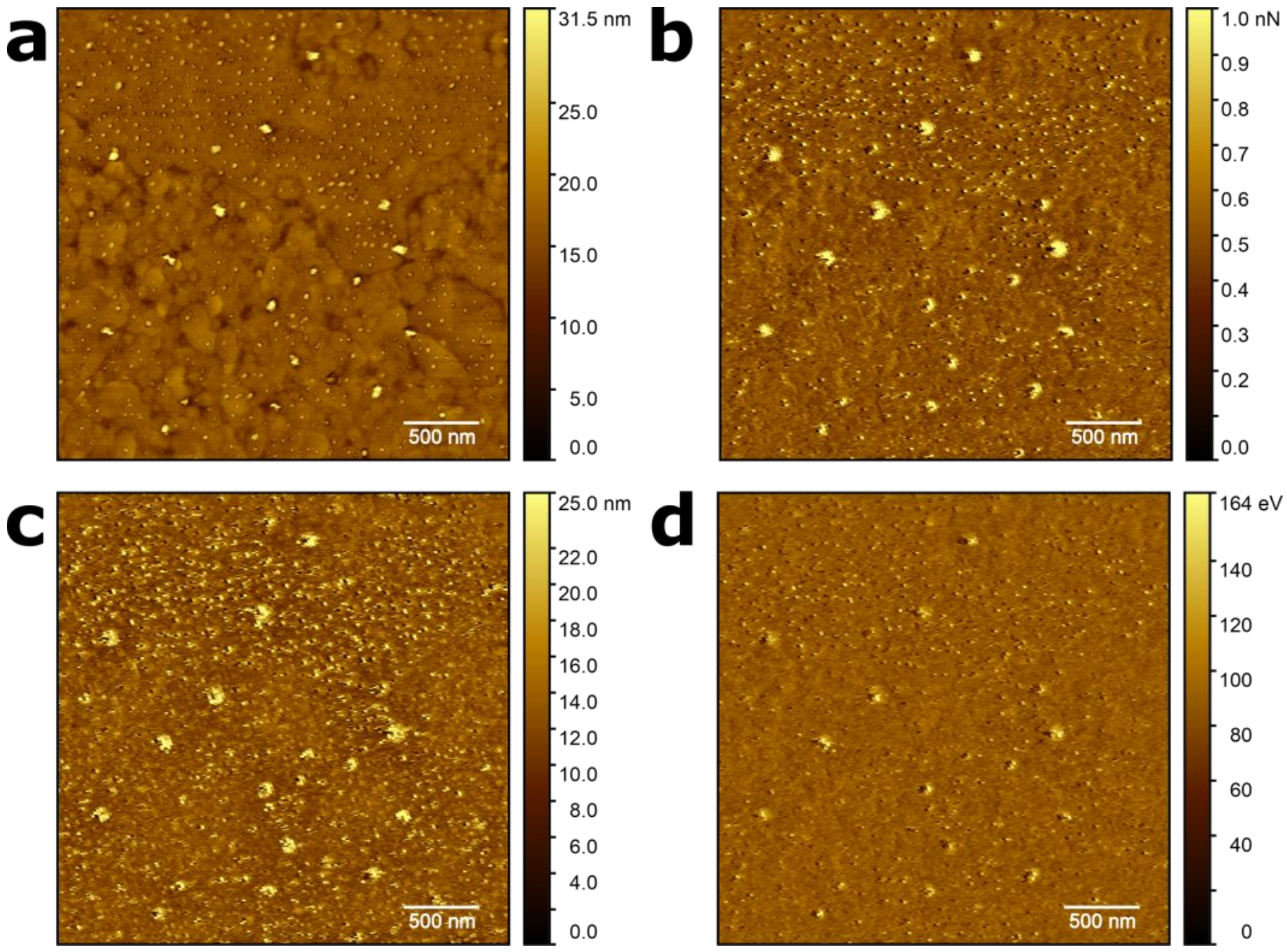

Figure S36: Trt3-M type 1: survey (a-d) topography, adhesion, deformation, and dissipation, respectively. In type 1, elastic features are dominant over hard features.

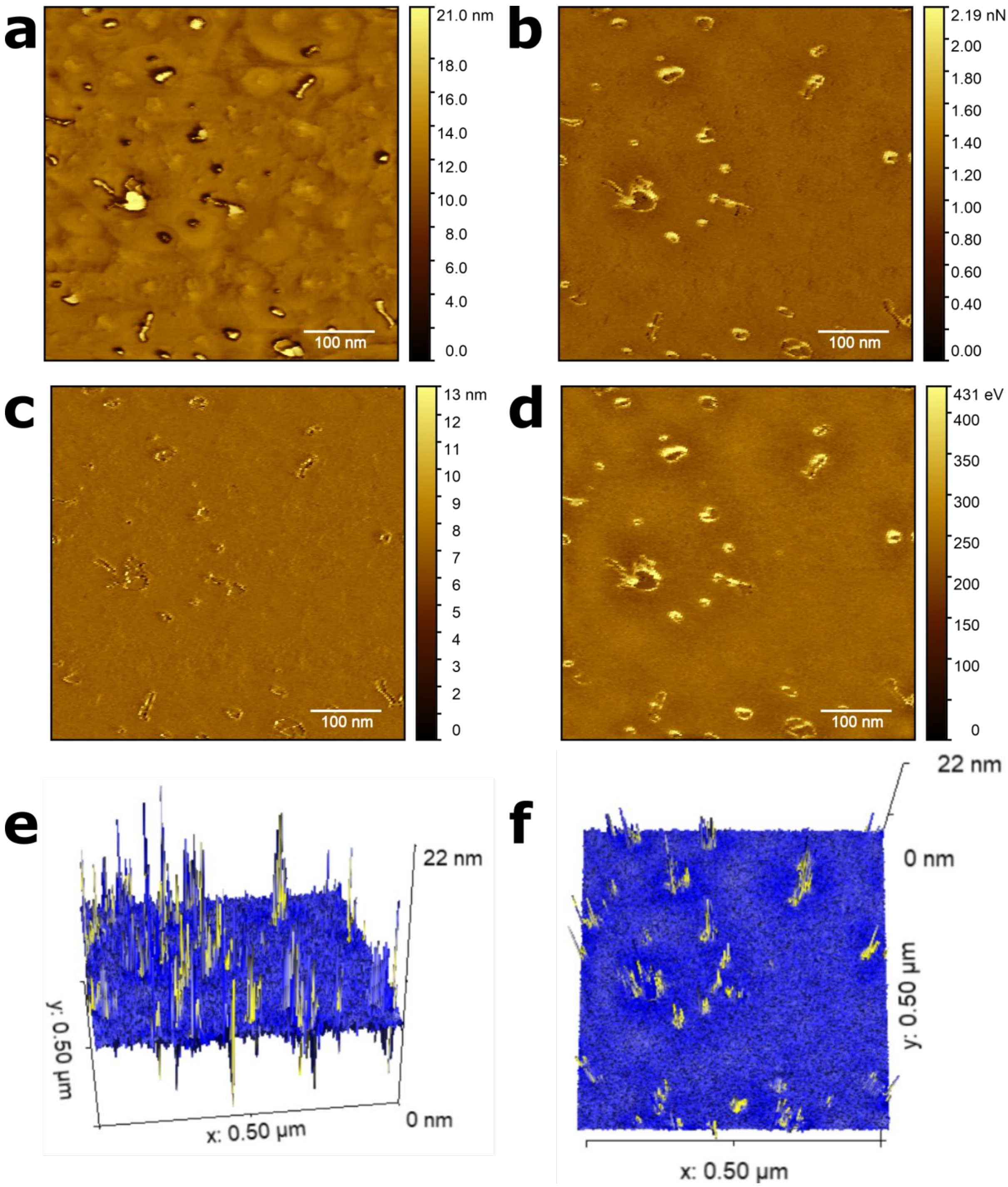

Figure S37: Trt3-M type 2 detail (a-d) topography, adhesion, deformation, and dissipation, respectively. (e,f) Correlation of Deformation and Dissipation Channel. As for recipe 2, hard and elastic features are almost equal at a 7:8 ratio of area%.

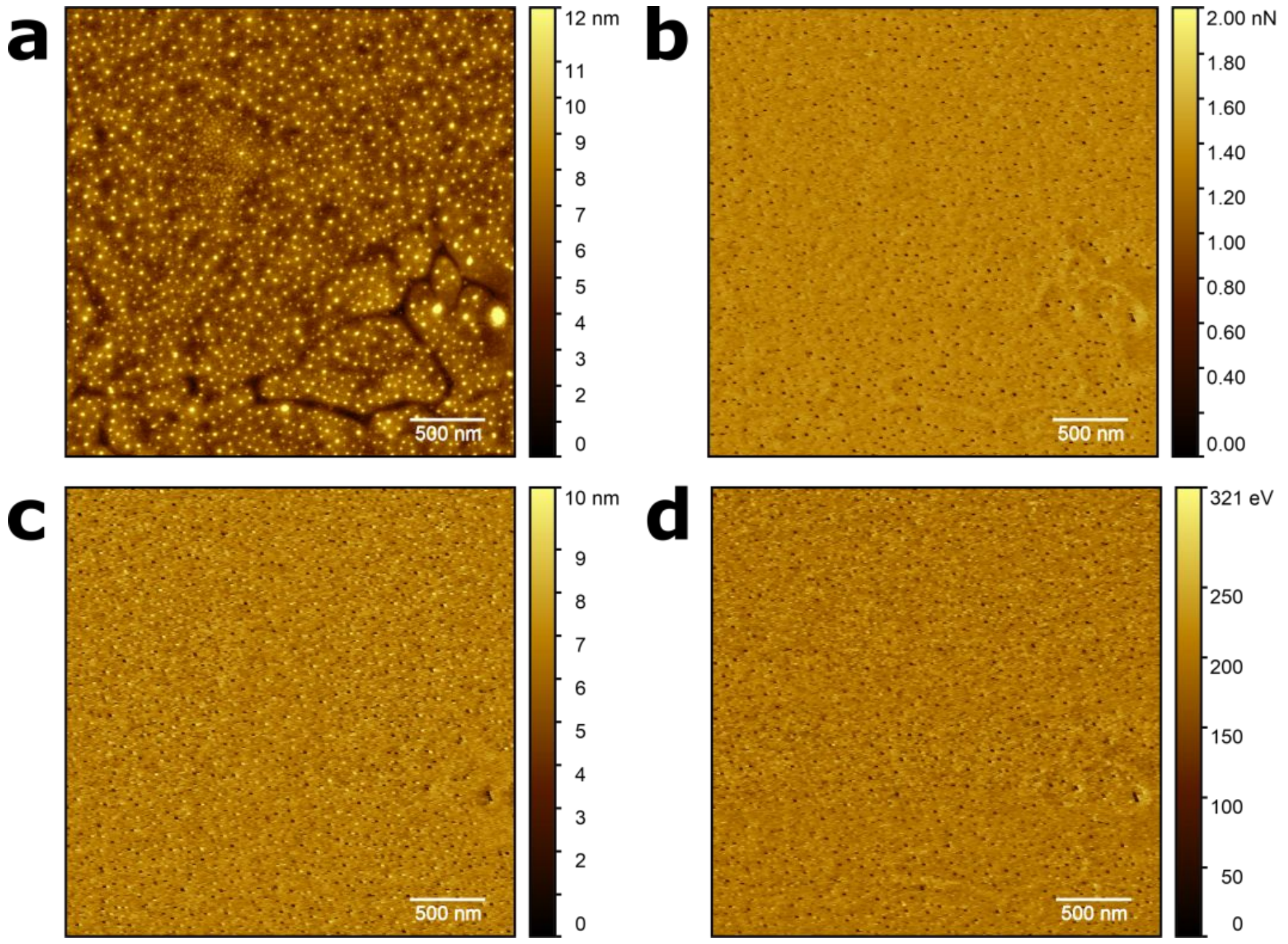

Figure S38: Trt4-M Survey (a-d) topography, adhesion, deformation, and dissipation, respectively.

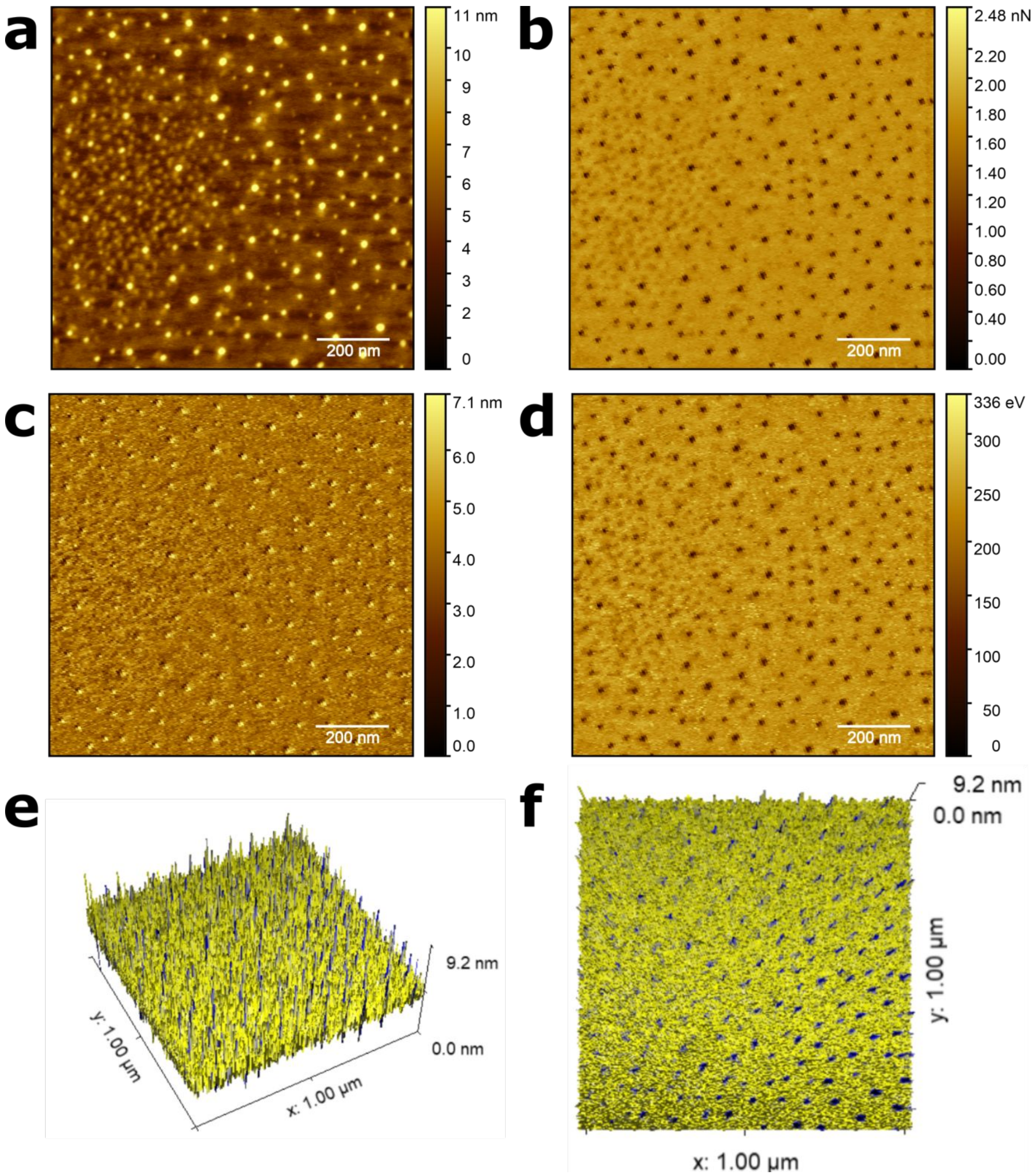

Figure S39: Trt4-M Detail (a-d) topography, adhesion, deformation, and dissipation, respectively. (e,f) Correlation of Deformation and Dissipation Channel. We observe a prevalence of plastic and adhesive behaviour with topographic features being either hard or elastically deforming in nature. The ratio of the latter is 9:5 with a large variance of the elastic fraction in-between samples and sample areas. This presents an inversion of the observations for its TRT-P counterpart, where type 2 resulted in elastic areas being the dominant ordered assembly type.

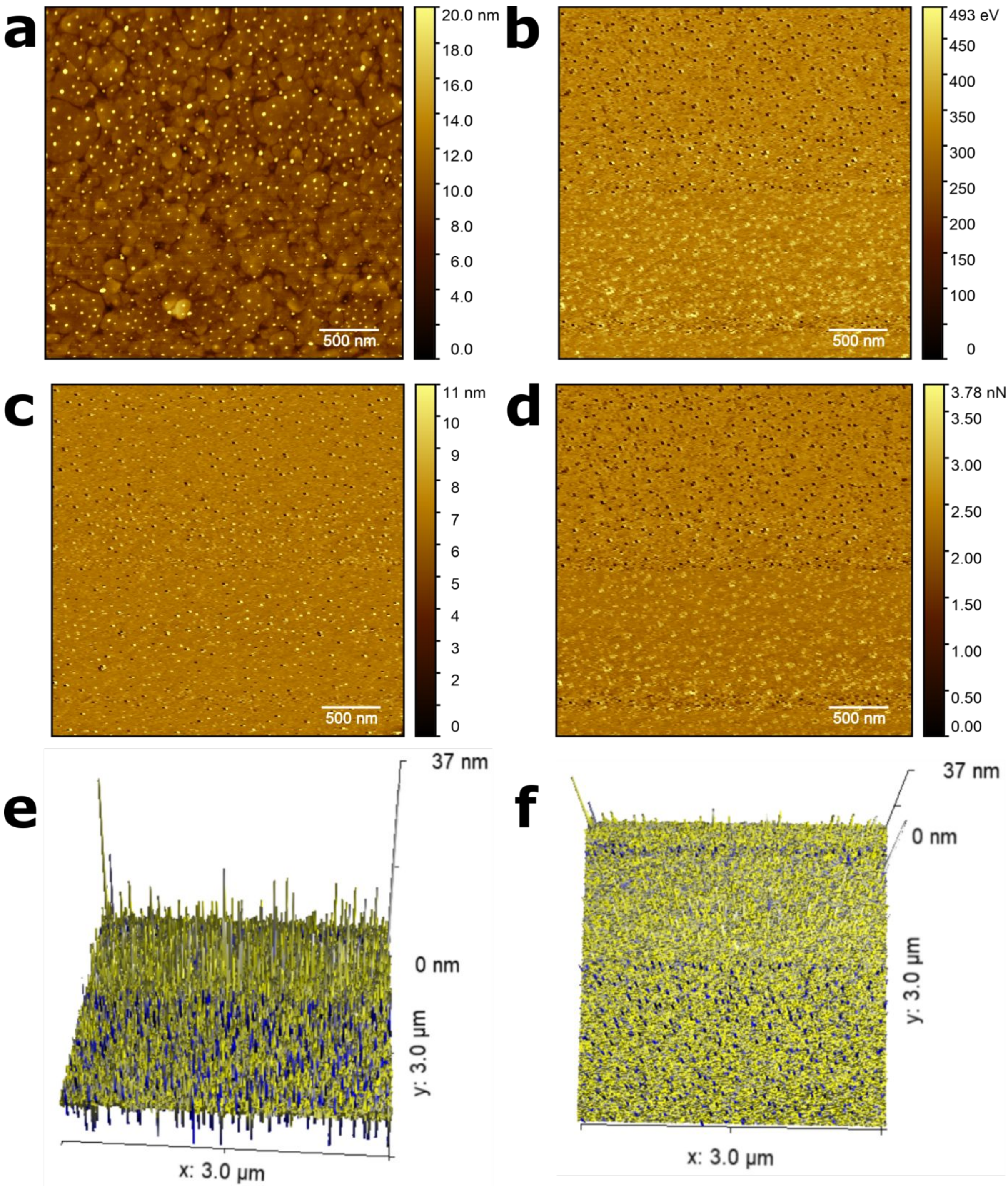

Figure S40: TRT5-M (a-d) topography, adhesion, deformation, and dissipation, respectively. (e,f) Correlation of Deformation and Dissipation Channel. Similar to recipe 4, high dilution leads to a dominance of hard features (8:5).

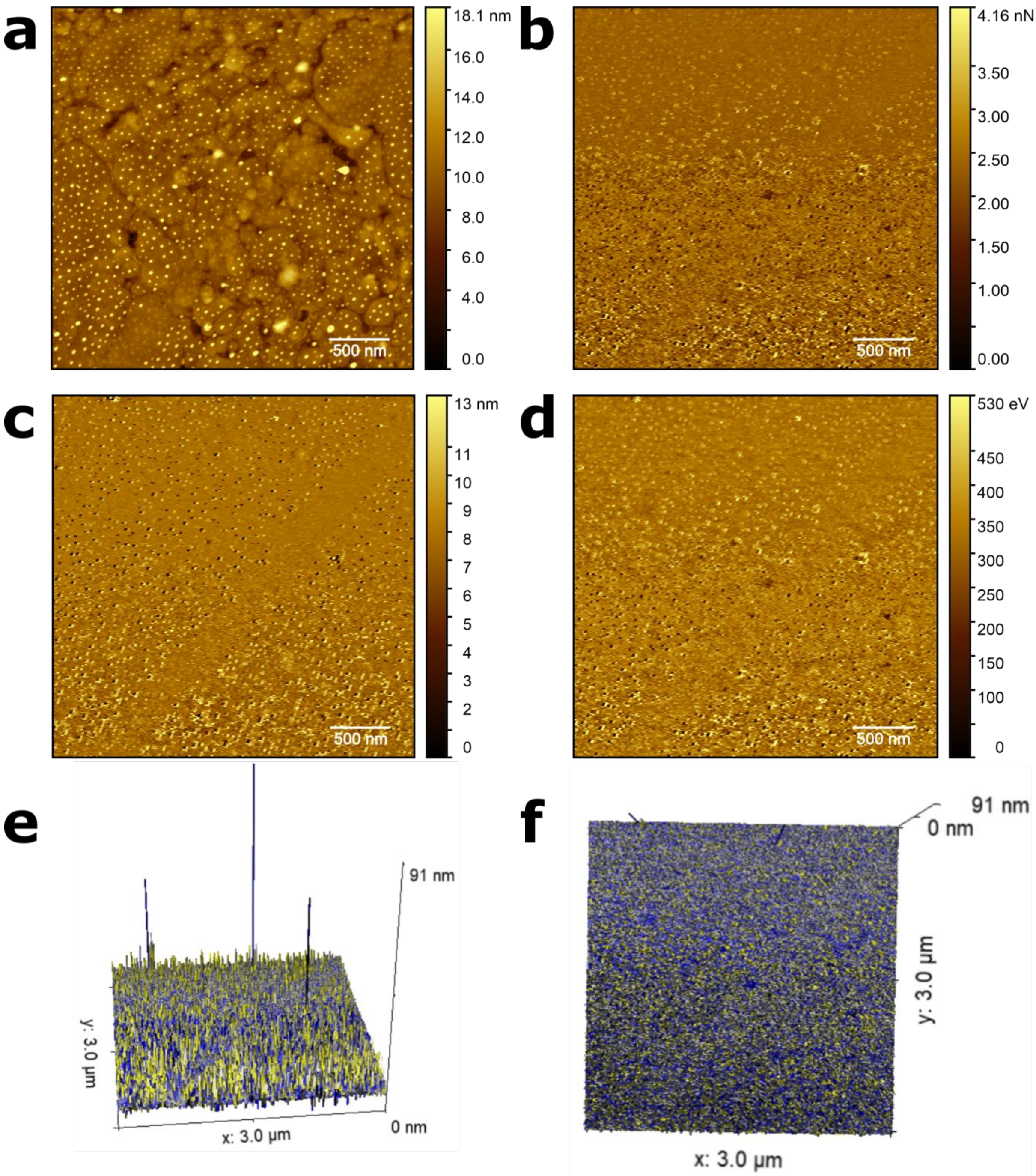

Figure S41: Trt6-M type 2, survey (a-d) topography, adhesion, deformation, and dissipation, respectively. (e,f) Correlation of Deformation and Dissipation Channel. Both, type 1 and 2 are dominated by hard features at similar ratios. In type two, these hard, topographically significant features were found to be surrounded by plastically deforming agglomerations. In both assembly types hard vs. elastic features were found at a 7:4 and 9:4 ratio, respectively, for type 1 and 2.

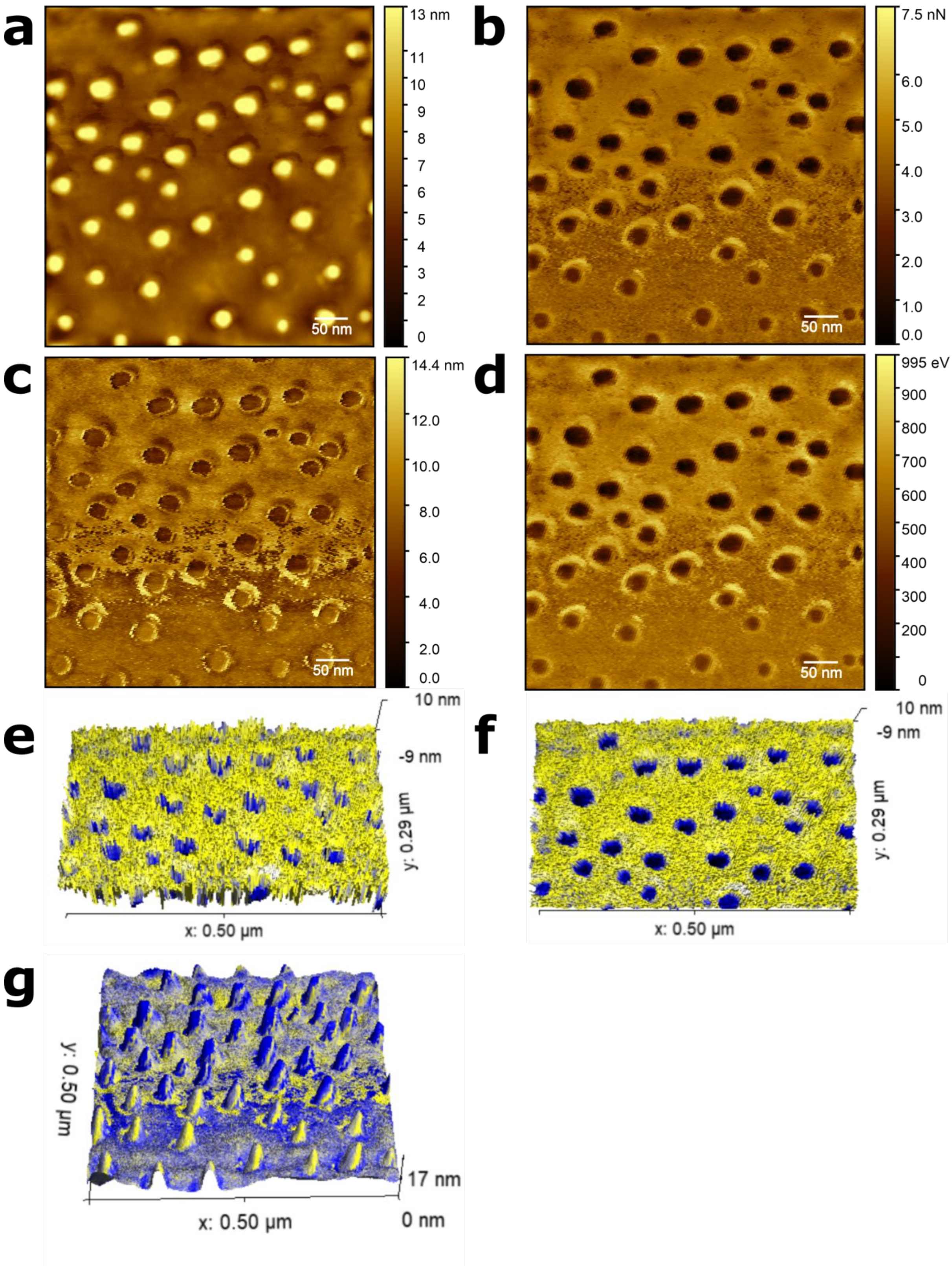

Figure S42: Trt6-M type 2 detail Seedlings of r = 5nm, h = 1nm discovered! Initial layer edge-on. (a-d) topography, adhesion, deformation, and dissipation, respectively. (e,f) Correlation of Deformation and Dissipation channel. (g) Topography vs. dissipation (colour-scale). Close-up images of the TRT. We observe the co-existence of hard elastic features, the former being more densely found on the surface.

High Resolution Images: Trt5 vs. PH5

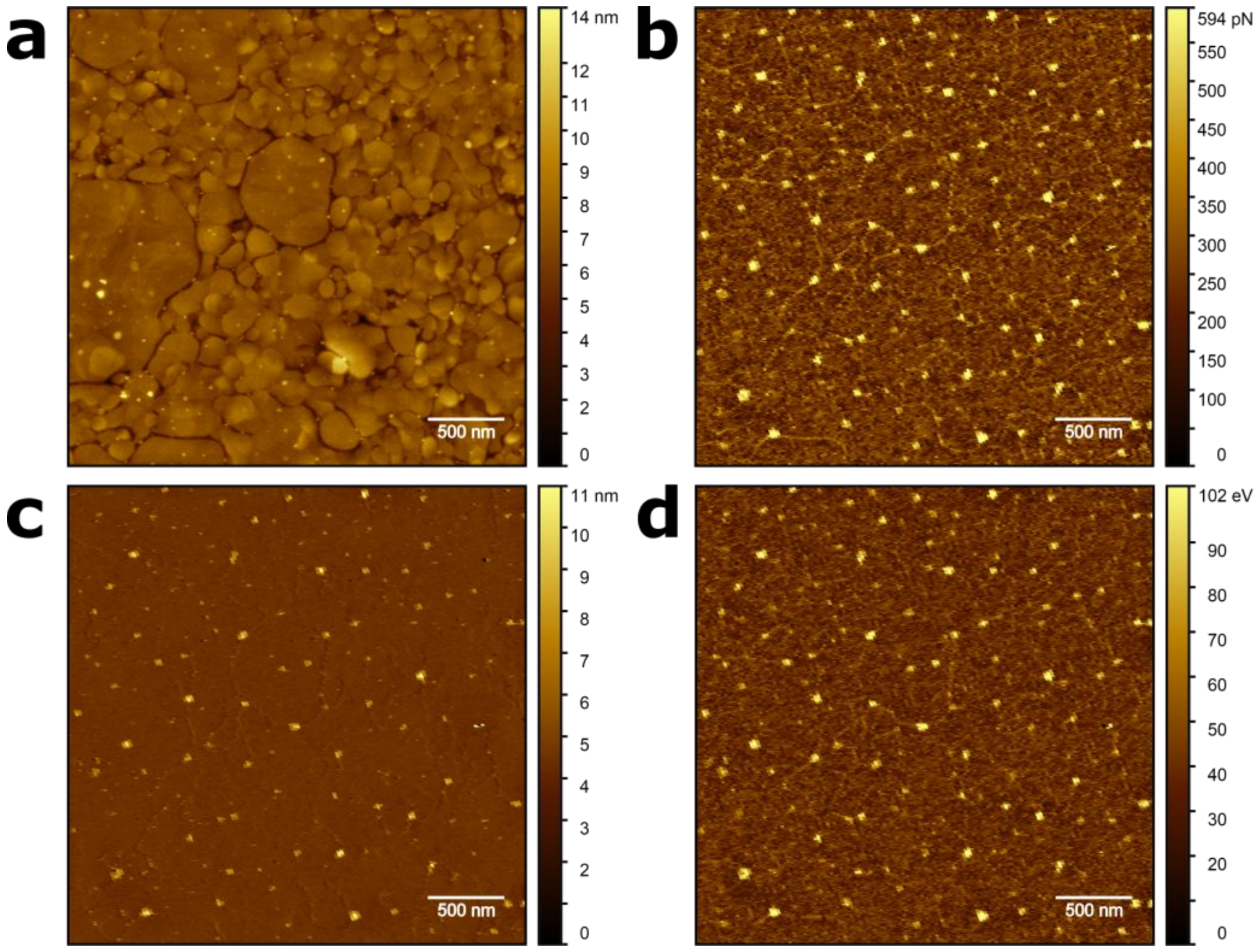

Figure S43: PH5 HR Survey. (a-d) topography, adhesion, deformation, and dissipation, respectively.

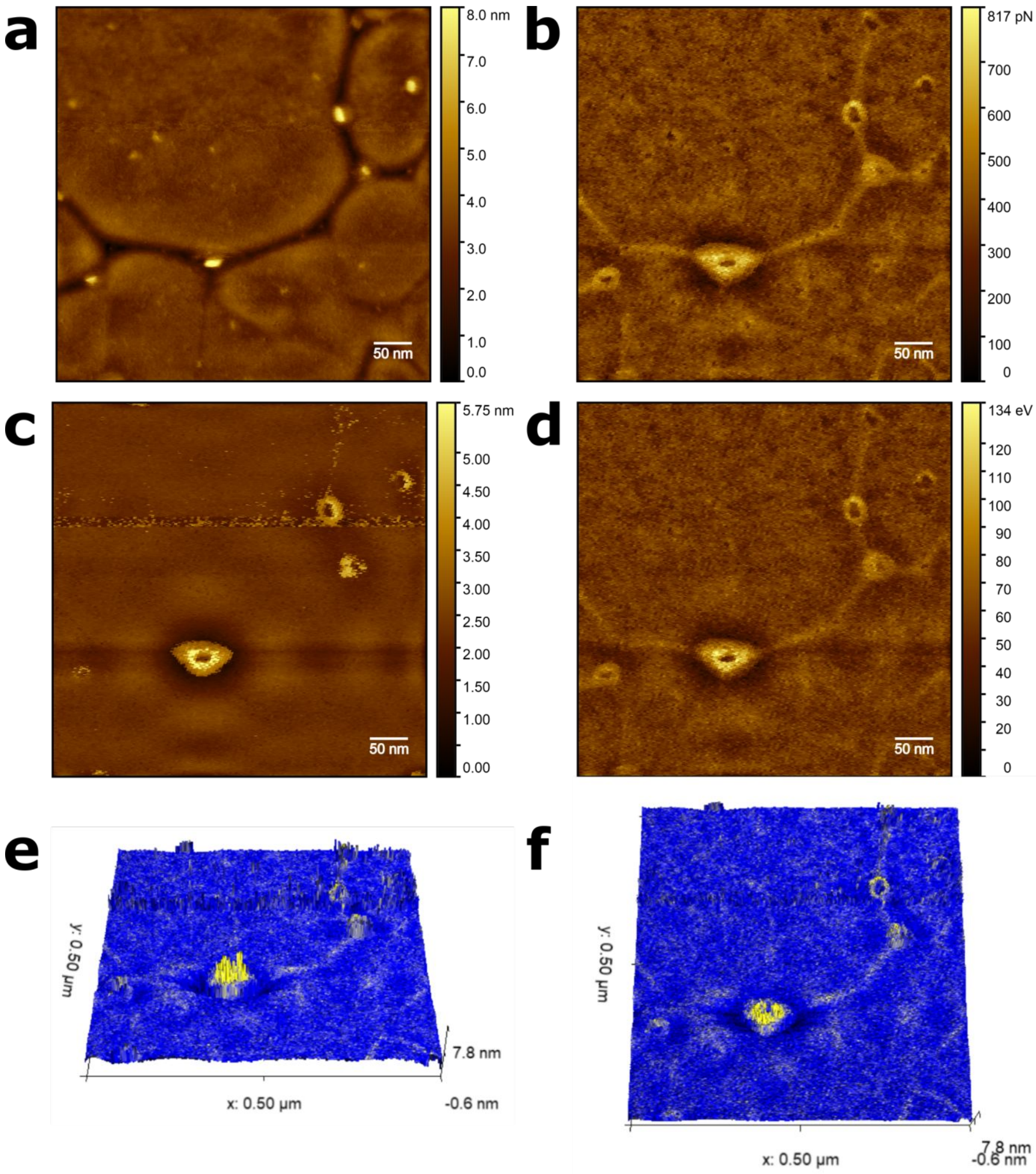

Figure S44: PH5 HR detail: 5 nm repetitive, 1nm high features = nucleation sites. Initial layer edge-on. (a-d) topography, adhesion, deformation, and dissipation, respectively. (e,f) Correlation of Deformation and Dissipation channel. The observed image is in agreement with the survey measurement, i.e. a low-dissipation background is interrupted by a few, high-deformation high-dissipation features coinciding with the biggest topographic features (yellow peaks plastic deformation). The nucleation sites observed in the topography (5 nm spherical features) are hardly visible in the low-dissipation background as they appear as dark, small spots (f). The nucleation sites are hence edge-on oriented.

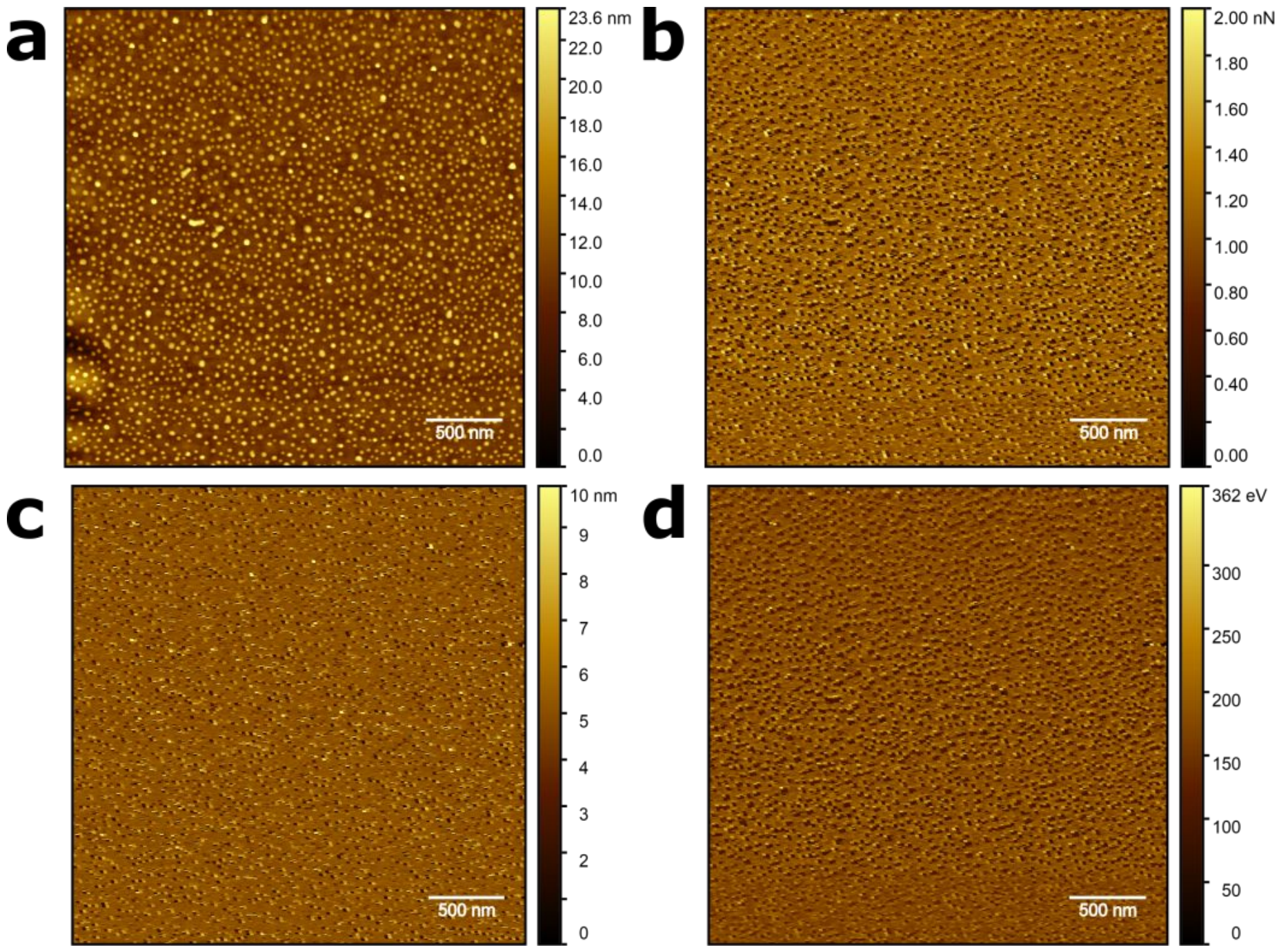

Figure S45: TRT5 HR Survey. (a-d) topography, adhesion, deformation, and dissipation, respectively.

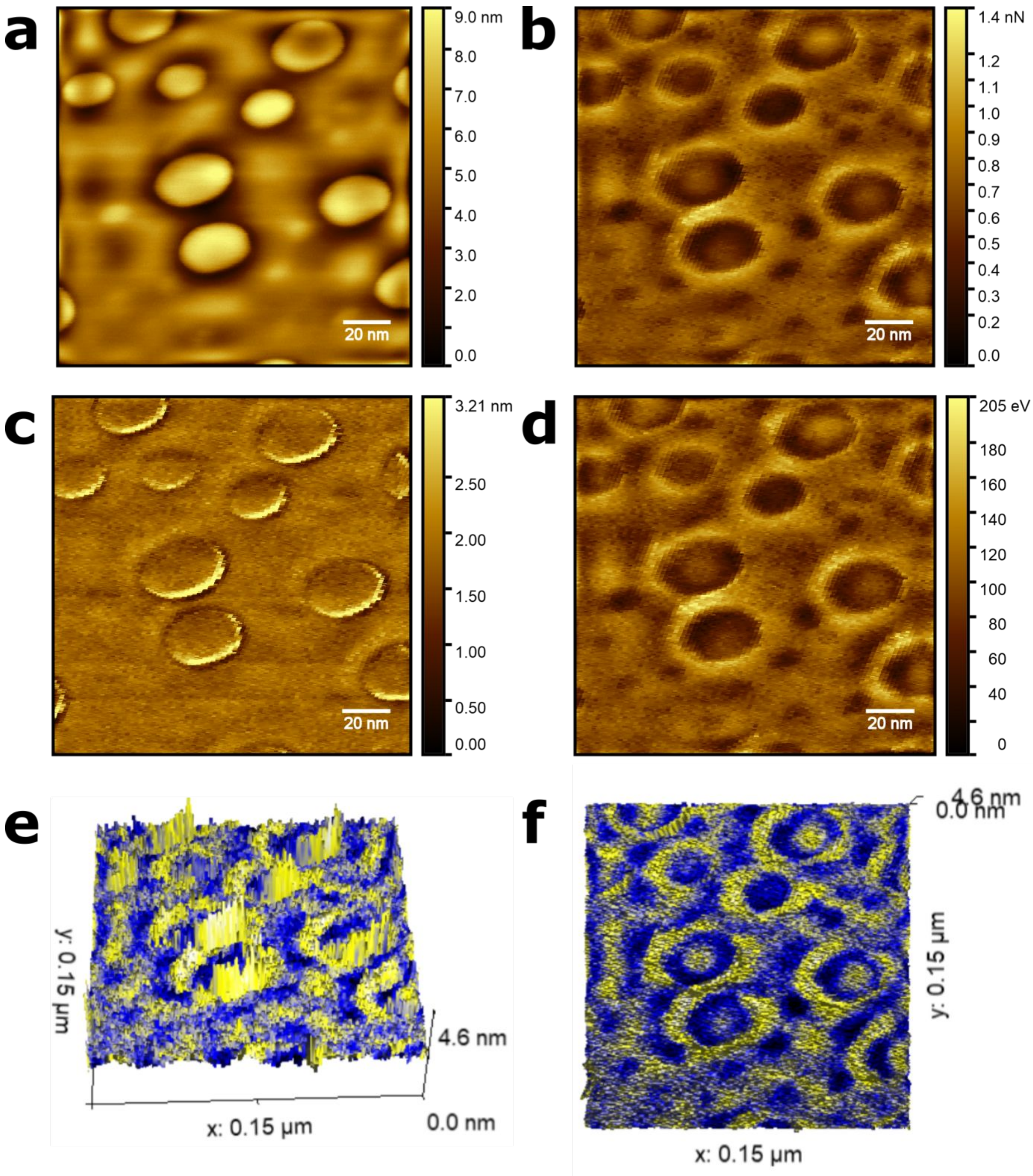

Figure S46: Trt5 HR Detail - nucleation size 10 nm, 1 nm high. Initial layer edge-on. (a-d) topography, adhesion, deformation, and dissipation, respectively. (e,f) Correlation of Deformation and Dissipation channel. The observed image is in agreement with the survey measurement, *i.e.* a low-dissipation background is interrupted by high-deformation high-dissipation signals in the vicinity of topographic features (secondary accumulation), which leads to crosstalk between channels. The readers attention should focus on the smaller nucleation sites of ca. 10 nm in diameter. These are the largest nucleation sites discovered yet are still only about 1 nm in height-too small for secondary accumulation of poorly organized material. These sites are edge-on oriented.

TRT-SAM: Racemate

From the helicenes P and M enantiomer a racemate solution was prepared and deposited on the TRT-SAM. The results are found below.

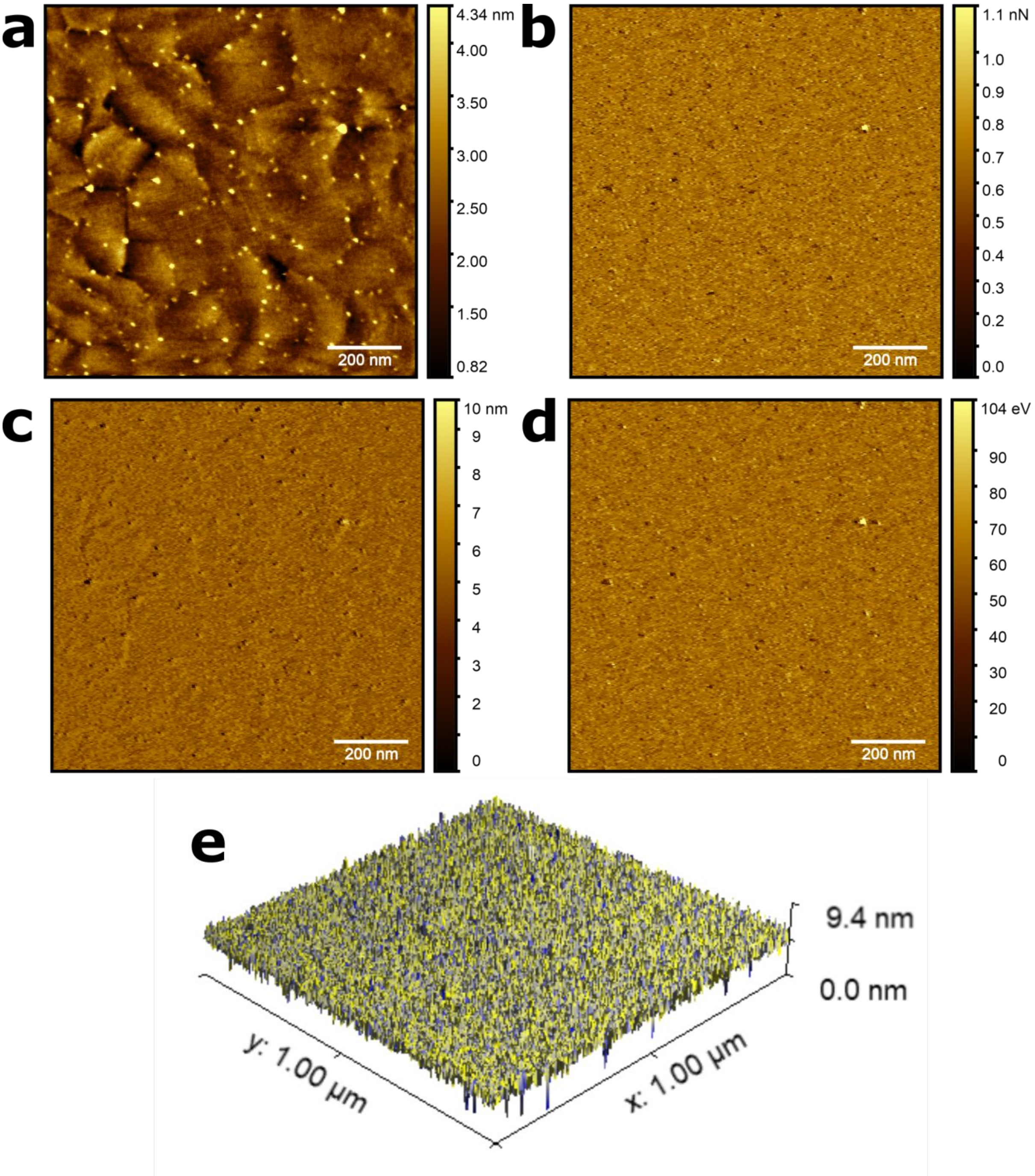

Figure S47: TRT1-R survey. (a-d) topography, adhesion, deformation, and dissipation, respectively. (e) Correlation of Deformation and Dissipation channel. The racemate behaves similarly to the M-enantiomer with a 7:6 ratio for hard-to-elastic area%.

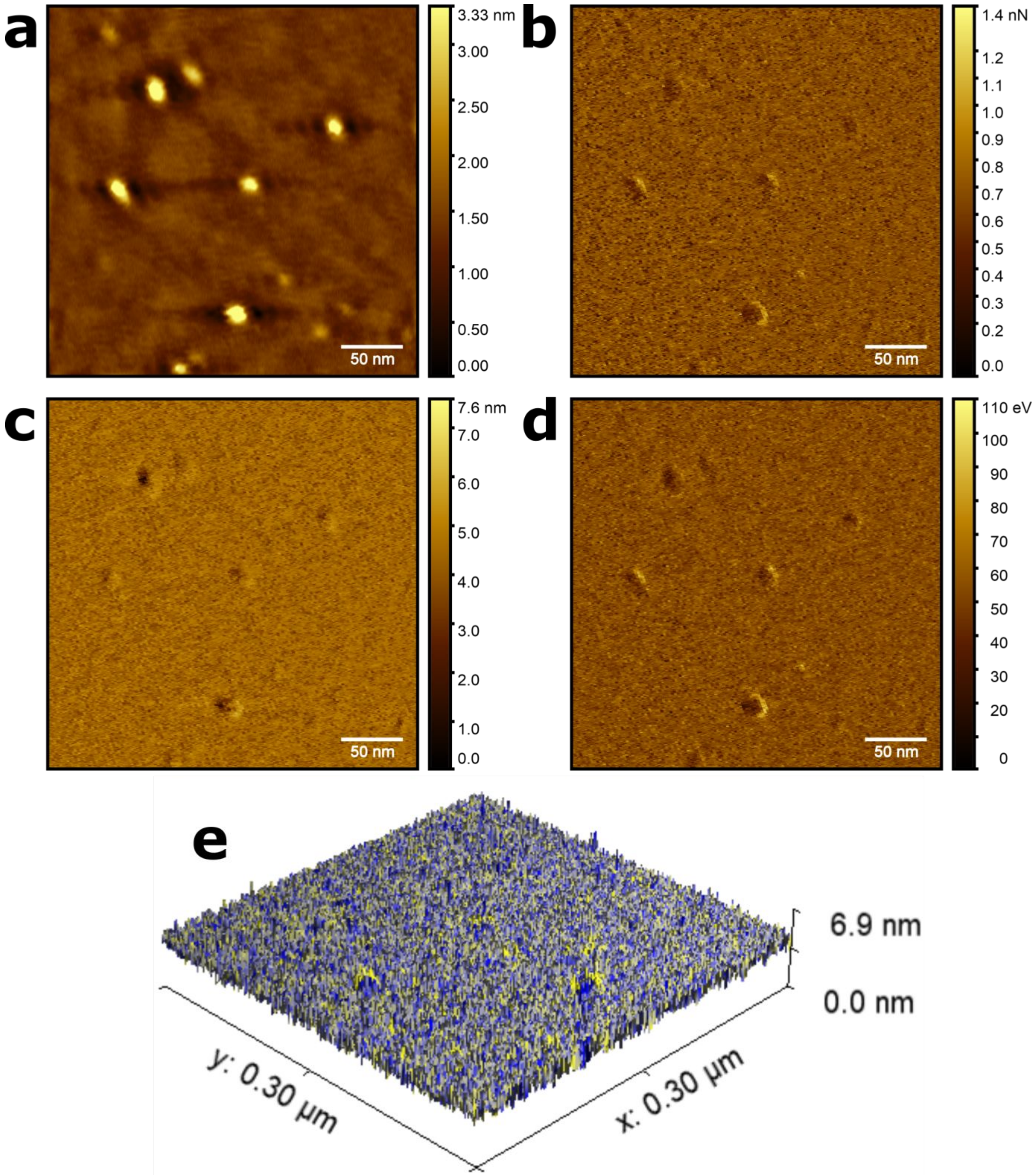

Figure S48: TRT1 - R detail. (a-d) topography, adhesion, deformation, and dissipation, respectively. (e) Correlation of Deformation and Dissipation channel. The racemate behaves similarly to the M-enantiomer with a 7:6 ratio for hard-to-elastic area%.

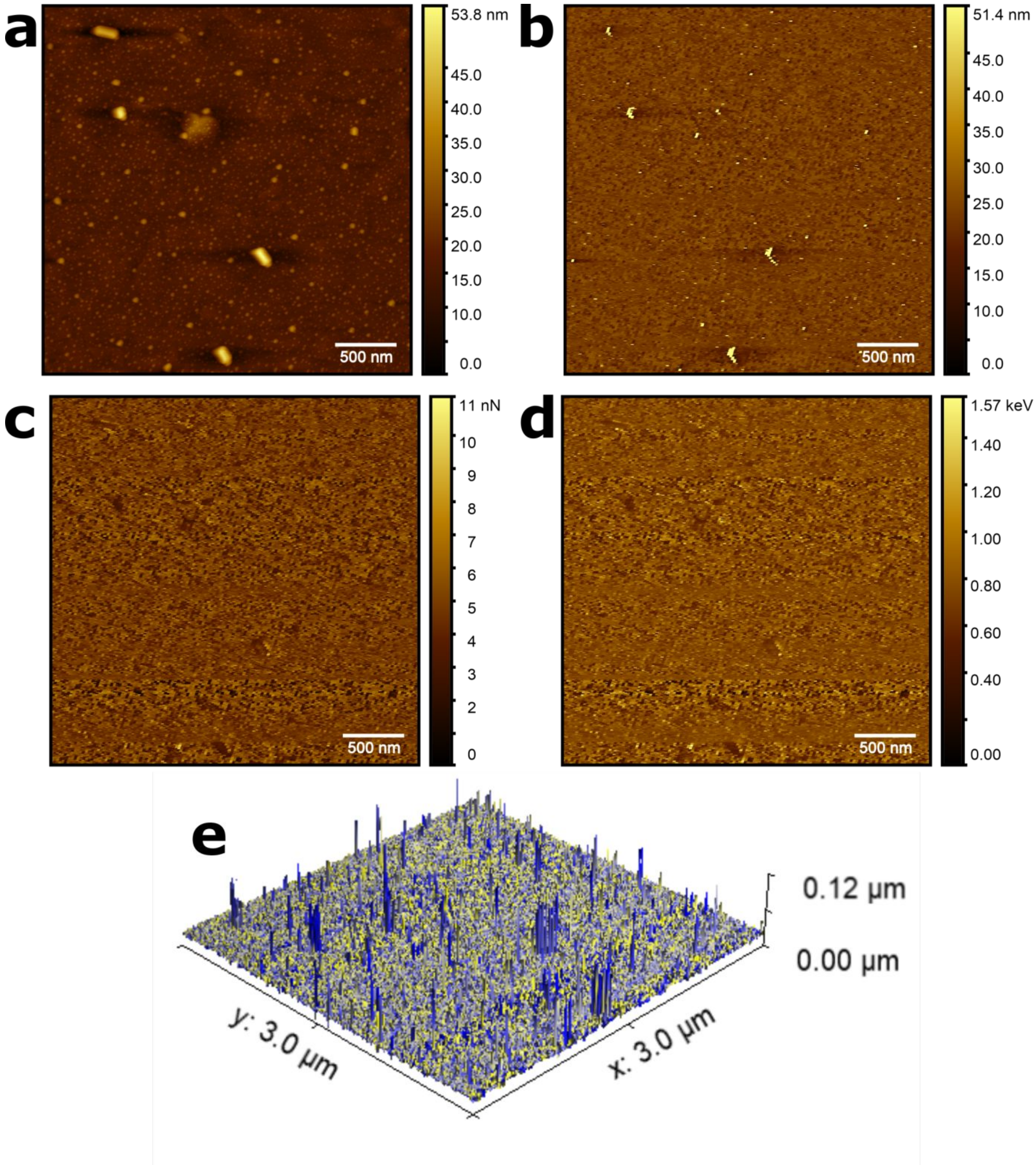

Figure S49: TRT2-R survey. (a-d) topography, adhesion, deformation, and dissipation, respectively. (e) Correlation of Deformation and Dissipation channel. The racemate behaves similarly to the P-enantiomer with a 11:3 ratio for hard-to-elastic area%. The racemic mixture leads to a larger statistical variation, *i.e.* we observe larger difference between samples and areas.

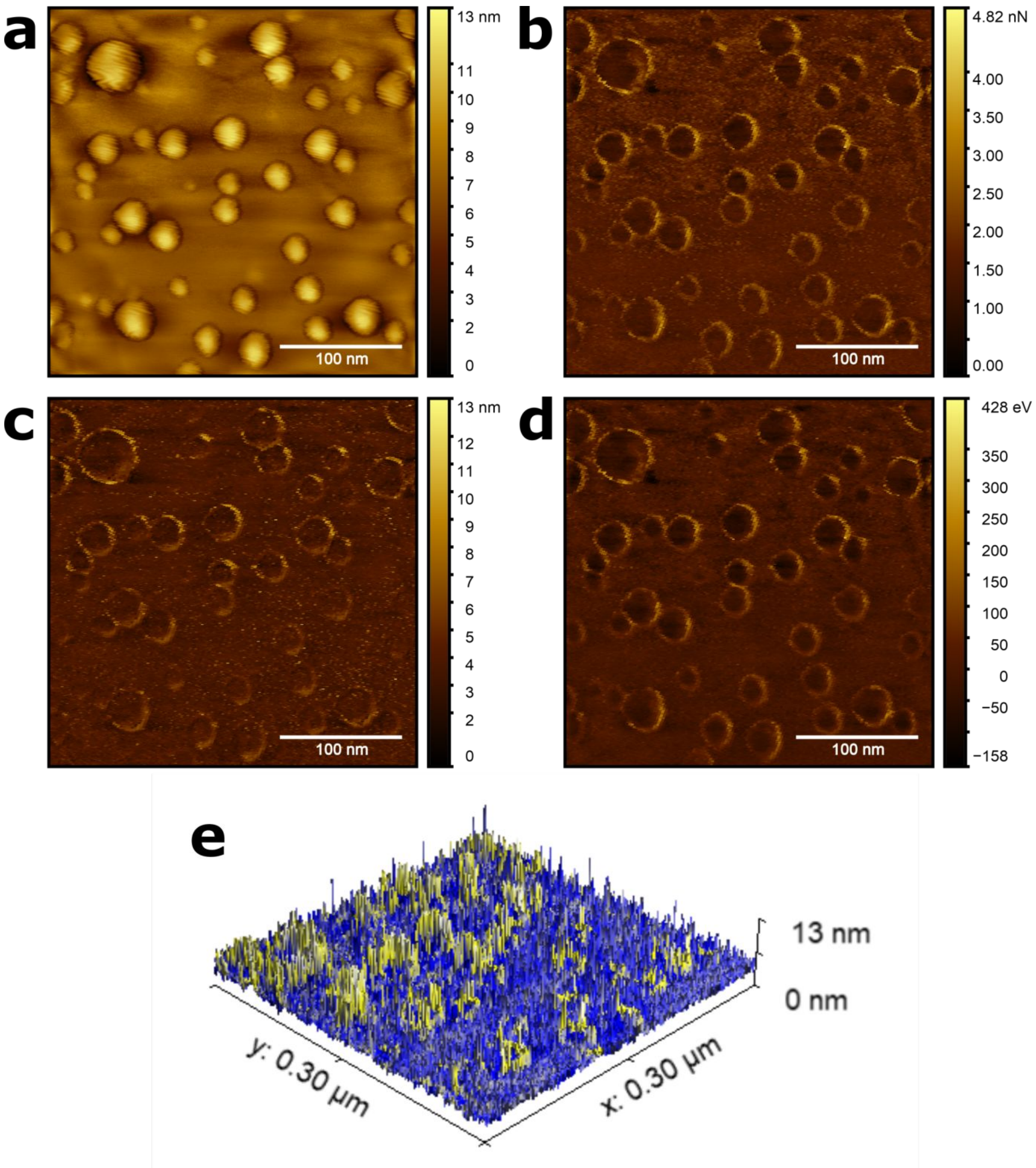

Figure S50: TRT2 - R detail. (a-d) topography, adhesion, deformation, and dissipation, respectively. (e) Correlation of Deformation and Dissipation channel. The hard topographic features are surrounded by poorly cohesive material (plastic deformation, yellow peaks).

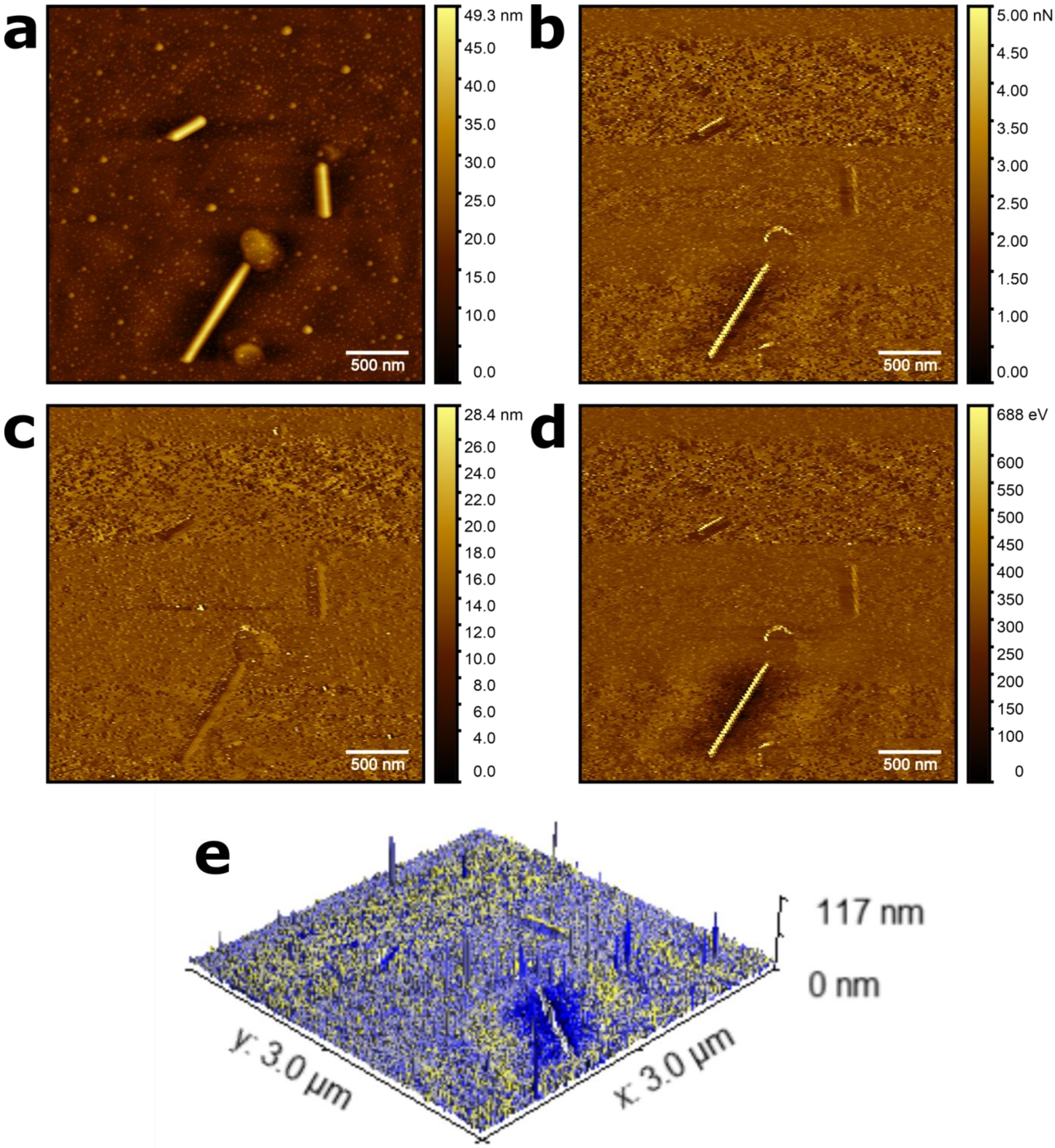

Figure S51: TRT3-R type 2 survey. (a-d) topography, adhesion, deformation, and dissipation, respectively. (e) Correlation of Deformation and Dissipation channel. Elongated structures were found in all the sample surfaces varying only in length. These indicates the formation of racemic crystals.[6] The racemate behaves similarly to the M-enantiomer with a 7:6 ratio for hard-to-elastic area%. Elastic features dominate at a 4:8 ratio.

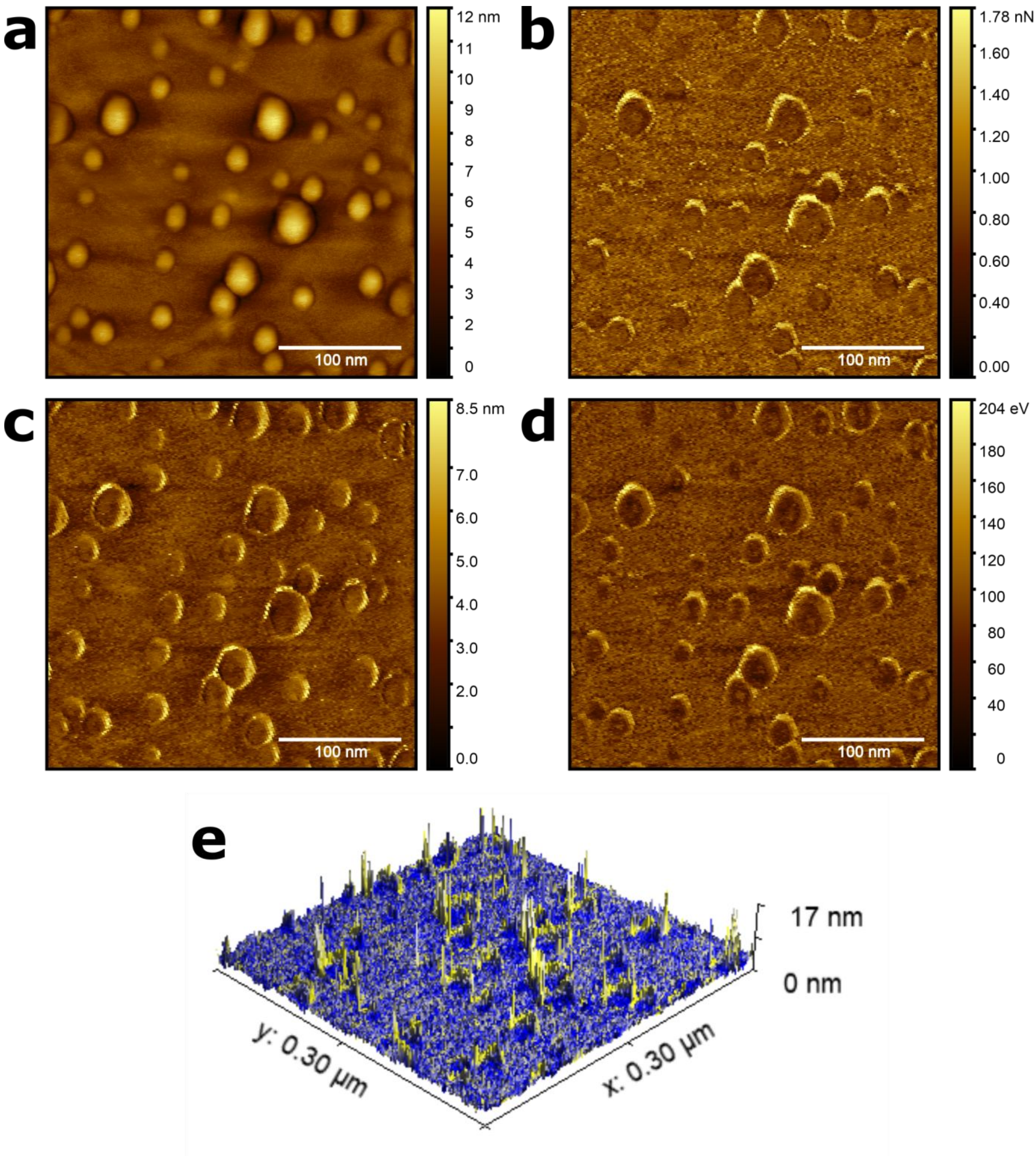

Figure S52: TRT3-R type 1 detail. (a-d) topography, adhesion, deformation, and dissipation, respectively. (e) Correlation of Deformation and Dissipation channel. This assembly type behaves similar to recipe 2 with hard features being the dominant assembly type and in exclusive to spherical structures.

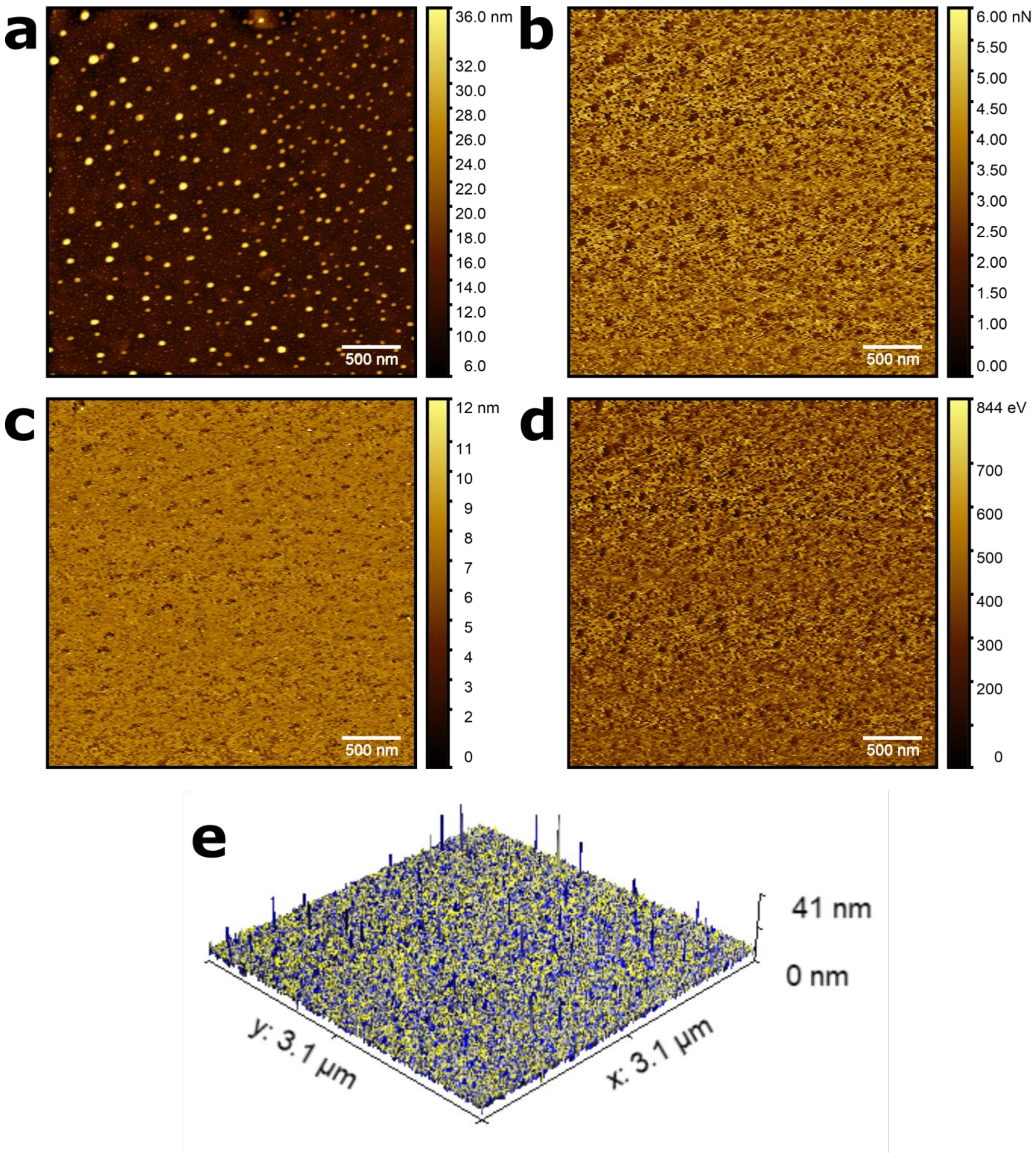

Figure S53: TRT4-R type 2 survey. (a-d) topography, adhesion, deformation, and dissipation, respectively. (e) Correlation of Deformation and Dissipation channel. Hard features and elastic features coexist at a ratio of 12:5 area%. Types 3 shows an additional, strongly adhesive component.

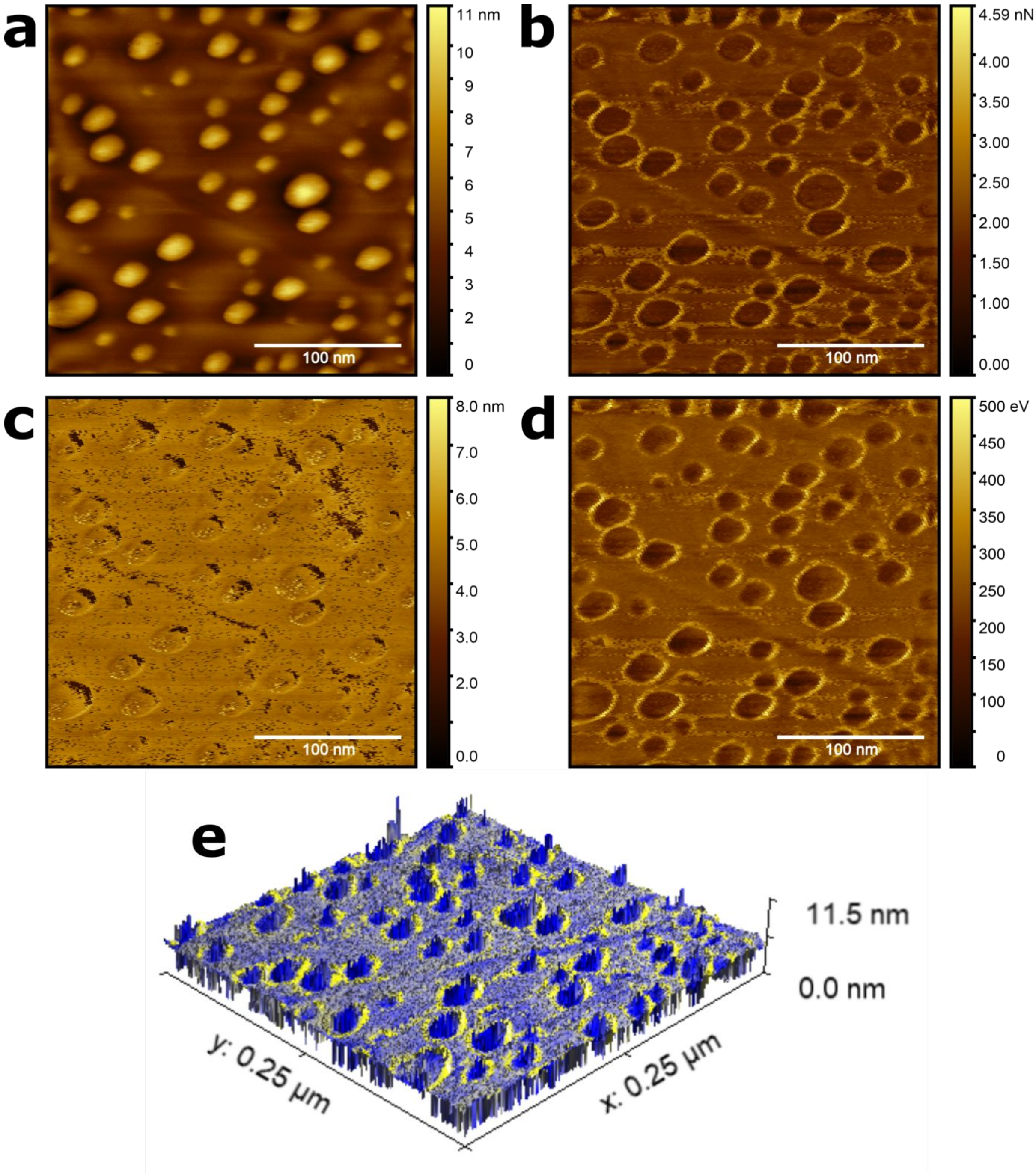

Figure S54: TRT4-R type 1 detail. (a-d) topography, adhesion, deformation, and dissipation, respectively. (e) Correlation of Deformation and Dissipation channel. In this assembly type, elastic features are dominant (3:13 ratio for hard vs. elastic features).

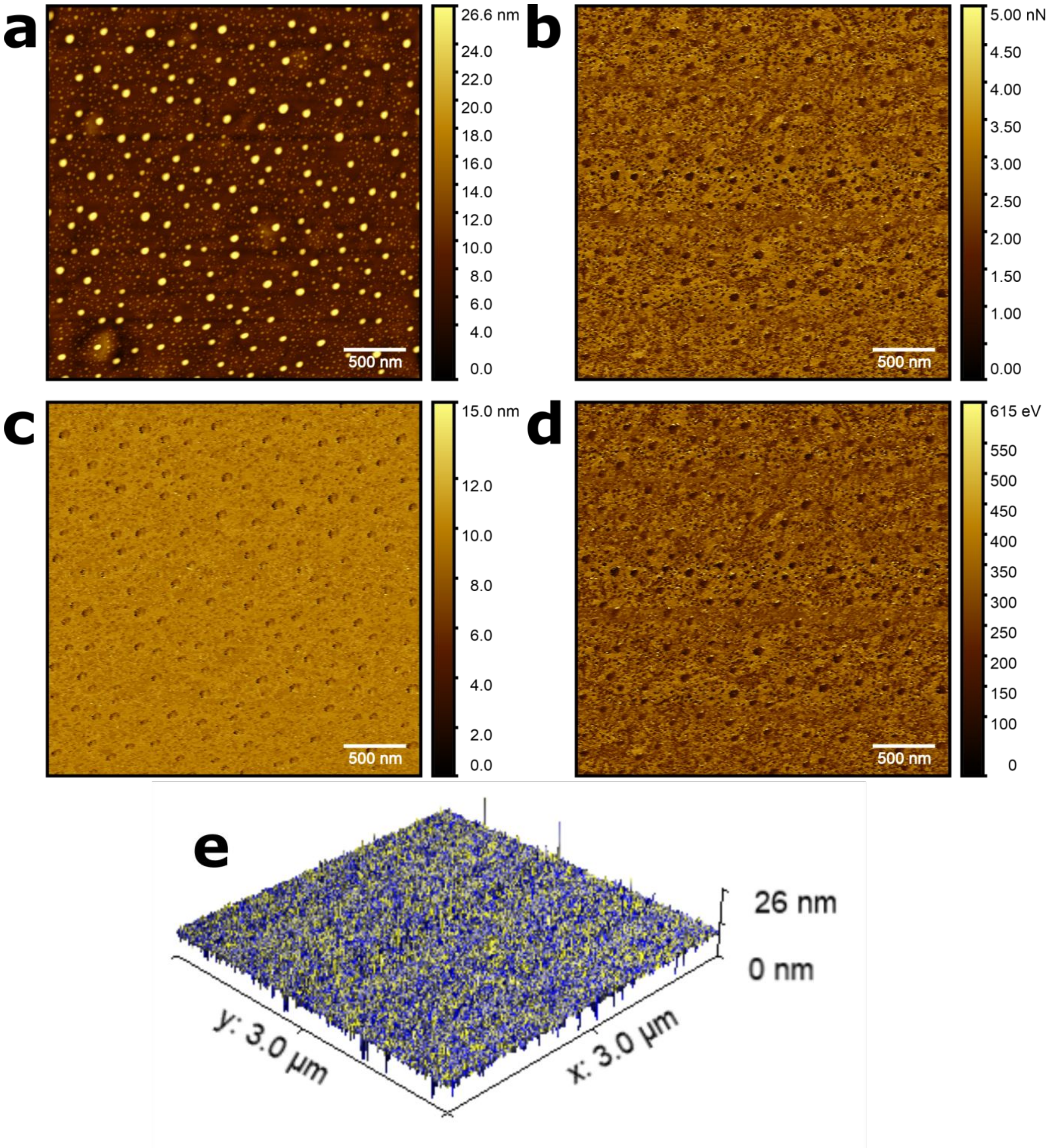

Figure S55: TRT5-R type 1 survey. (a-d) topography, adhesion, deformation, and dissipation, respectively. (e) Correlation of Deformation and Dissipation channel. Hard vs. elastic features (edge- vs. face-on) features exist at a 8:6 ratio, similar to the M-enantiomer.

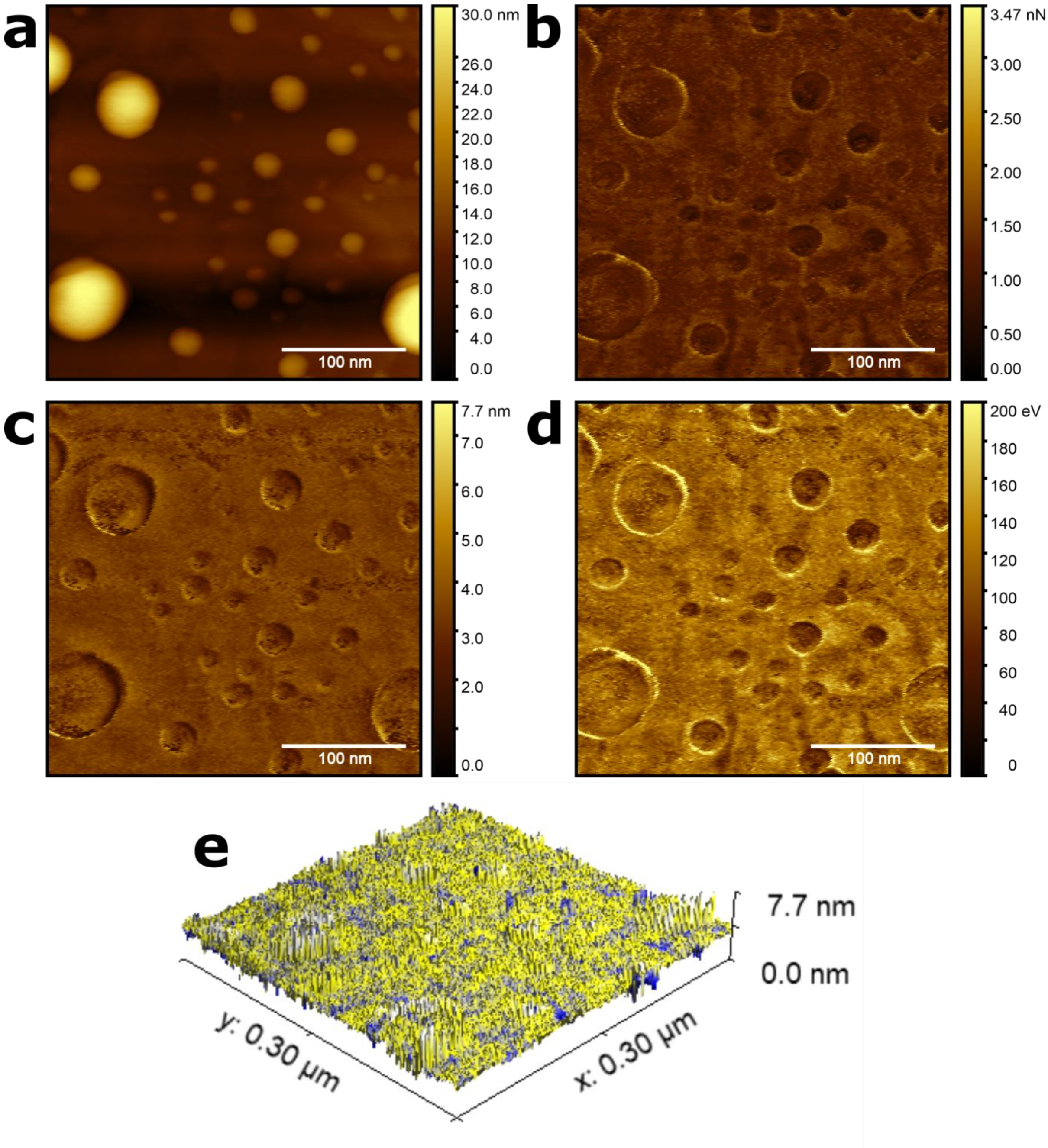

Figure S56: TRT5-R type 2 detail. (a-d) topography, adhesion, deformation, and dissipation, respectively. (e) Correlation of Deformation and Dissipation channel. Locally, we observe areas where a behaviour more similar to the pure P-enantiomer is found (12:4 ratio).

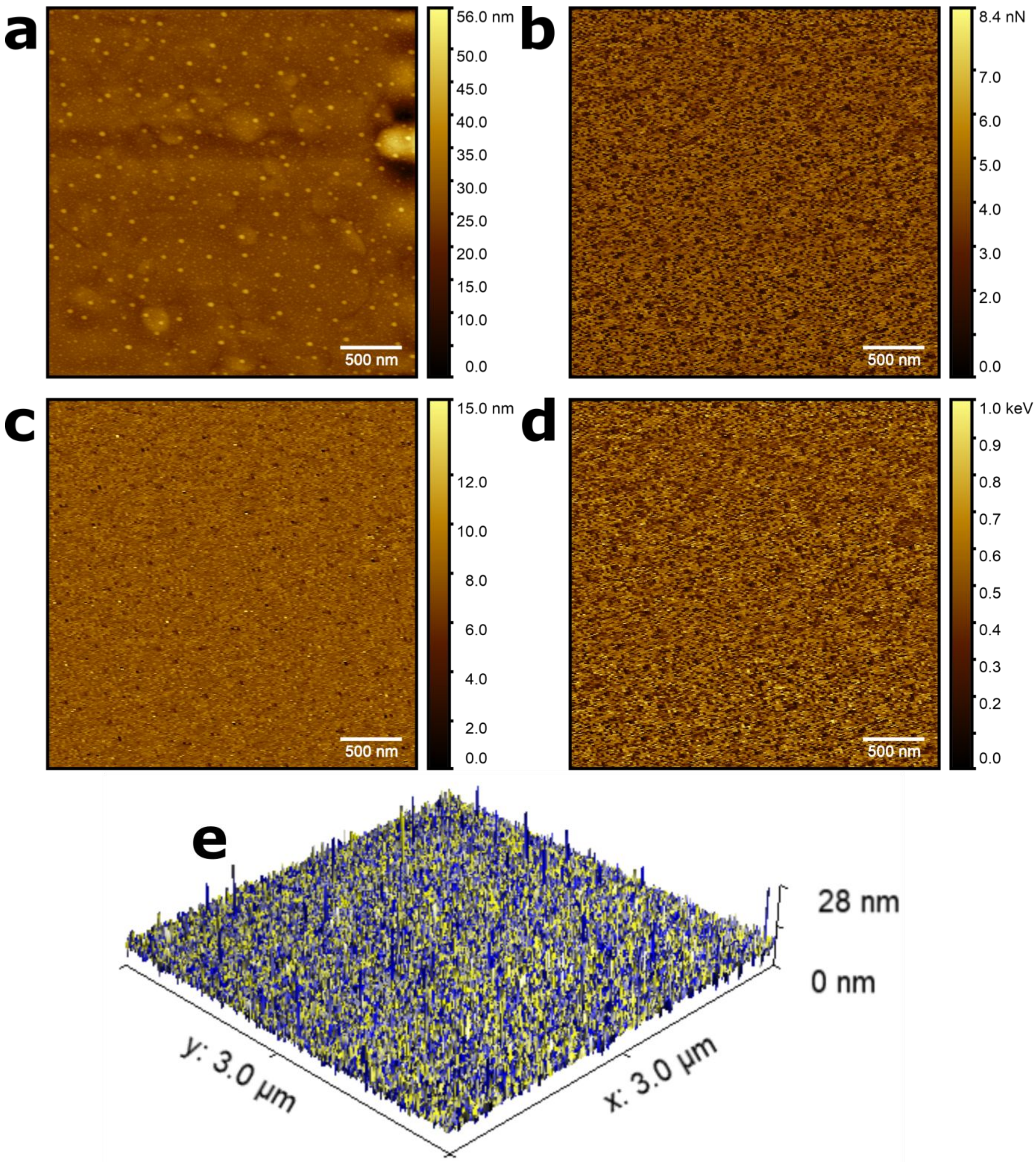

Figure S57: TRT6-R type 1 survey. (a-d) topography, adhesion, deformation, and dissipation, respectively. (e) Correlation of Deformation and Dissipation channel. Edge-on and face-on material co-exist at a 11:3 ratio. The few, blue peaks indicative of elastic deformation are distinct, yet very few. Type 1 shows three distinct populations in the dissipative channel as opposed to type 3, which otherwise mimics the ratio of hard vs. elastic features.

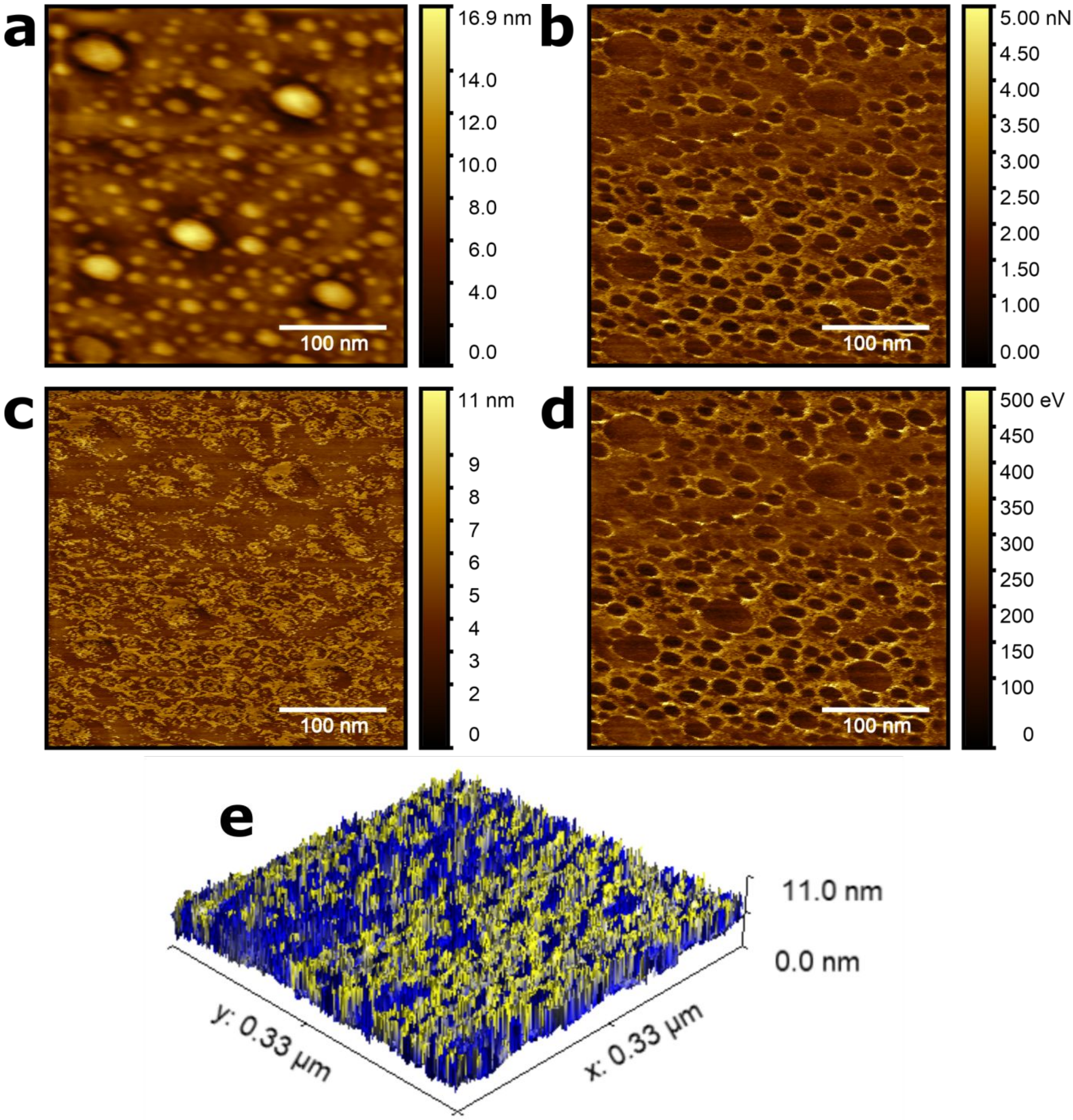

Figure S58: TRT6-R type 2 detail. (a-d) topography, adhesion, deformation, and dissipation, respectively. (e) Correlation of Deformation and Dissipation channel. Locally, edge-on and face-on material can be found at similar rates with a hard vs. elastic surface ratio of 13:13.

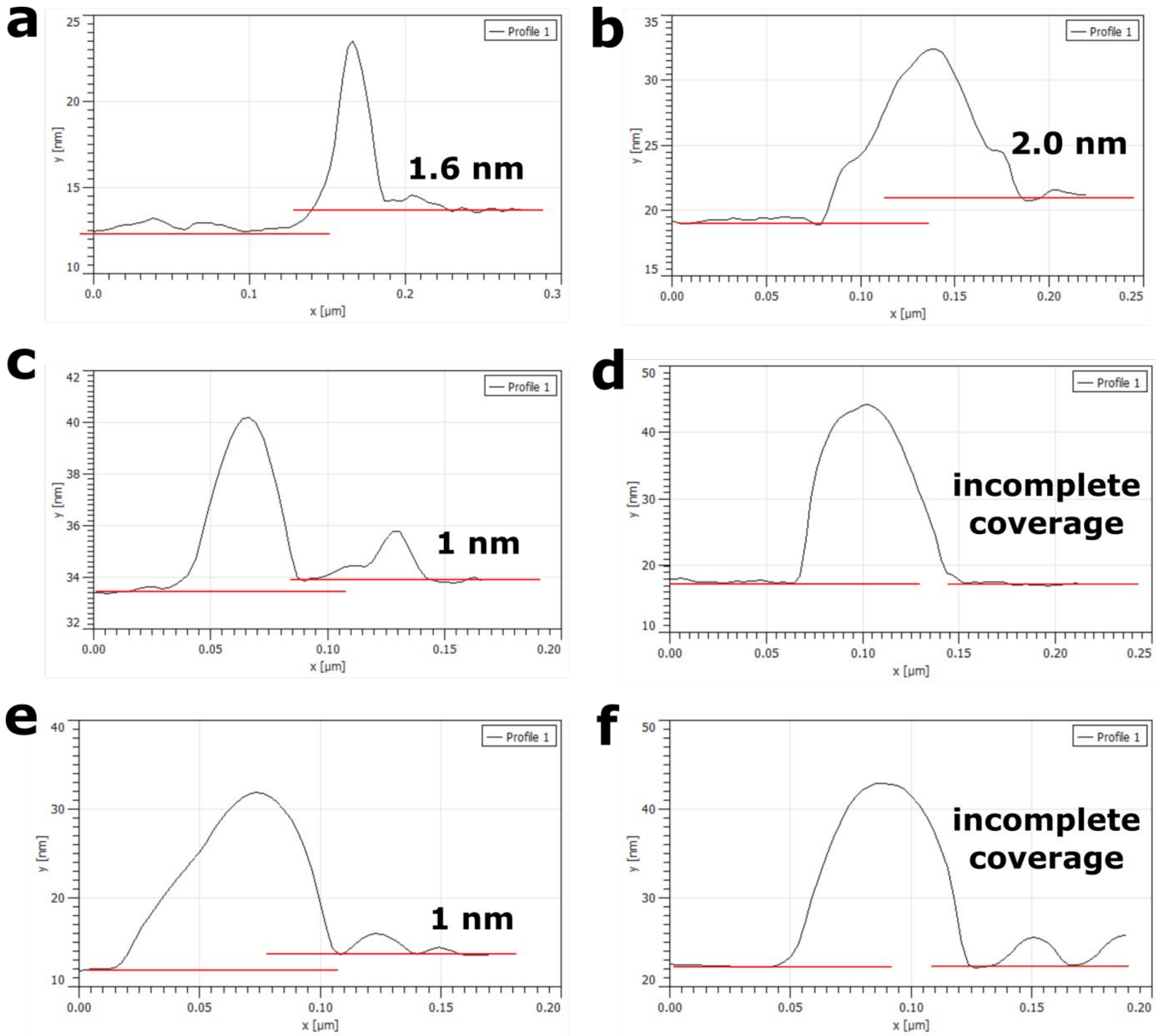

Figure S59: TRT-R thicknesses (racemate). Red lines as a guide for the eye.

Spectroscopy

Solution State Optical Characterisation

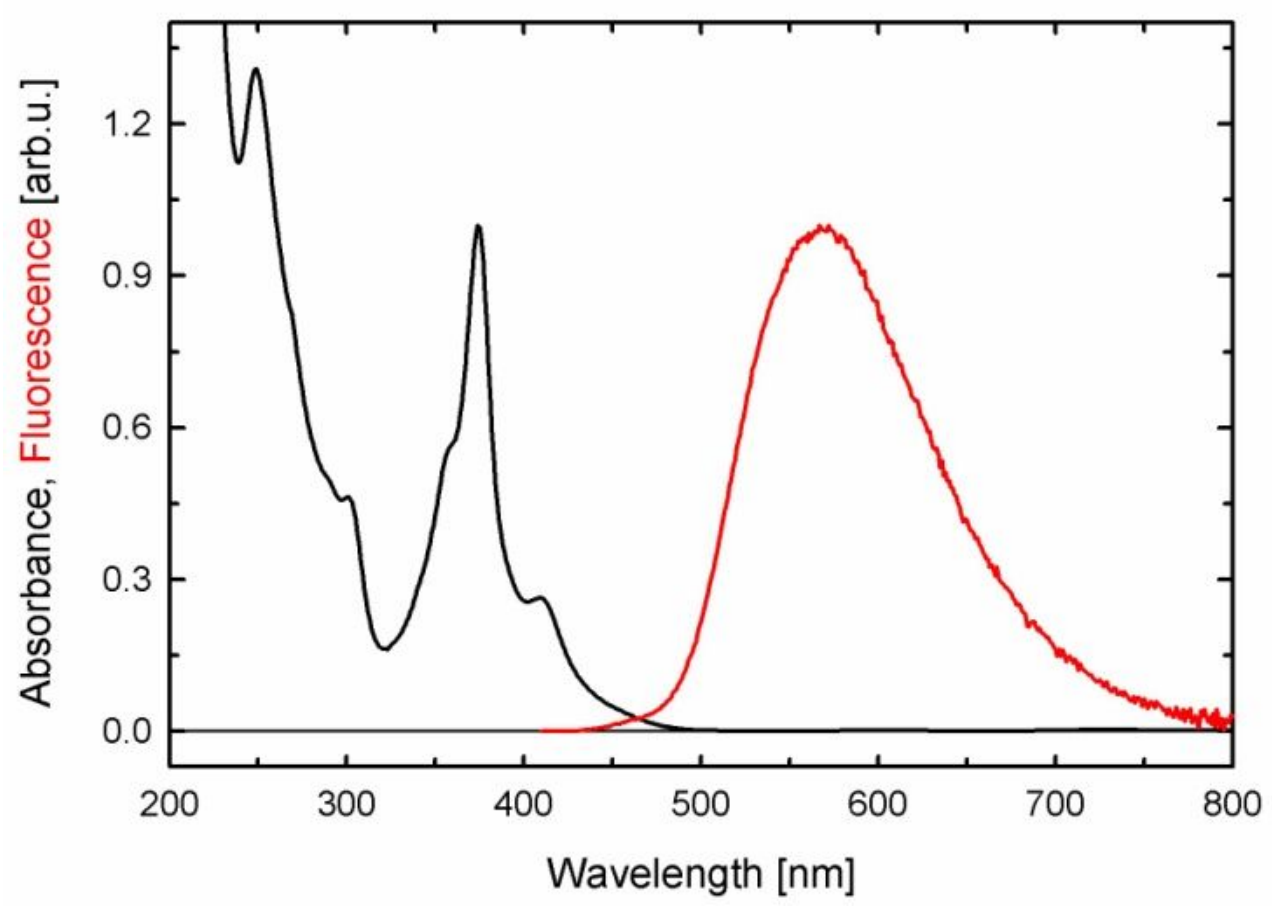

Figure S60: Absorption (black line) and fluorescence (red line) normalised spectra of recipe R1 (P-enantiomer). Excitation wavelength for fluorescence spectrum was 405 nm.

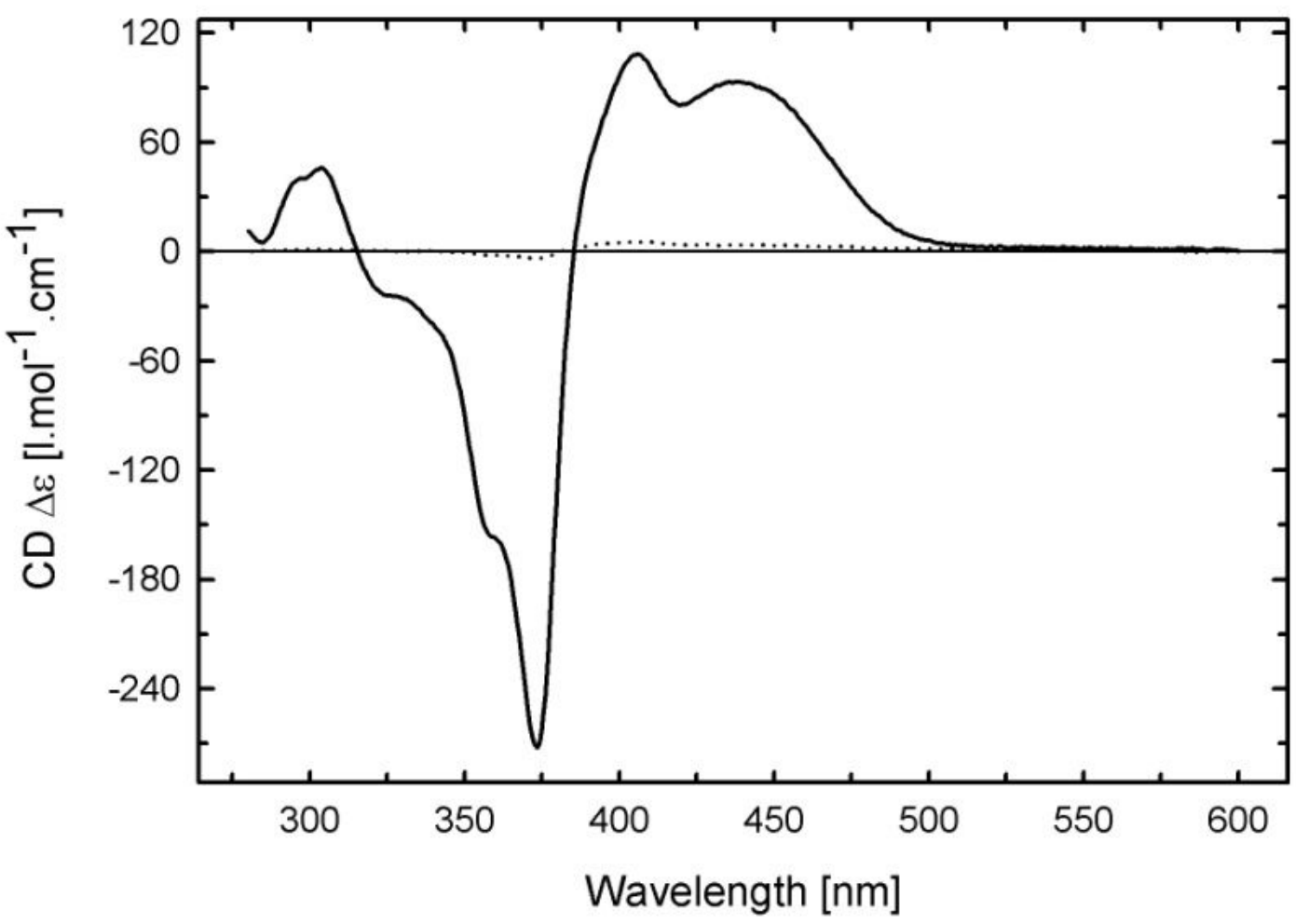

Figure S61: Comparison of electronic CD spectra of recipe R1 of P-enantiomer vs. the racemic solution (dotted line).

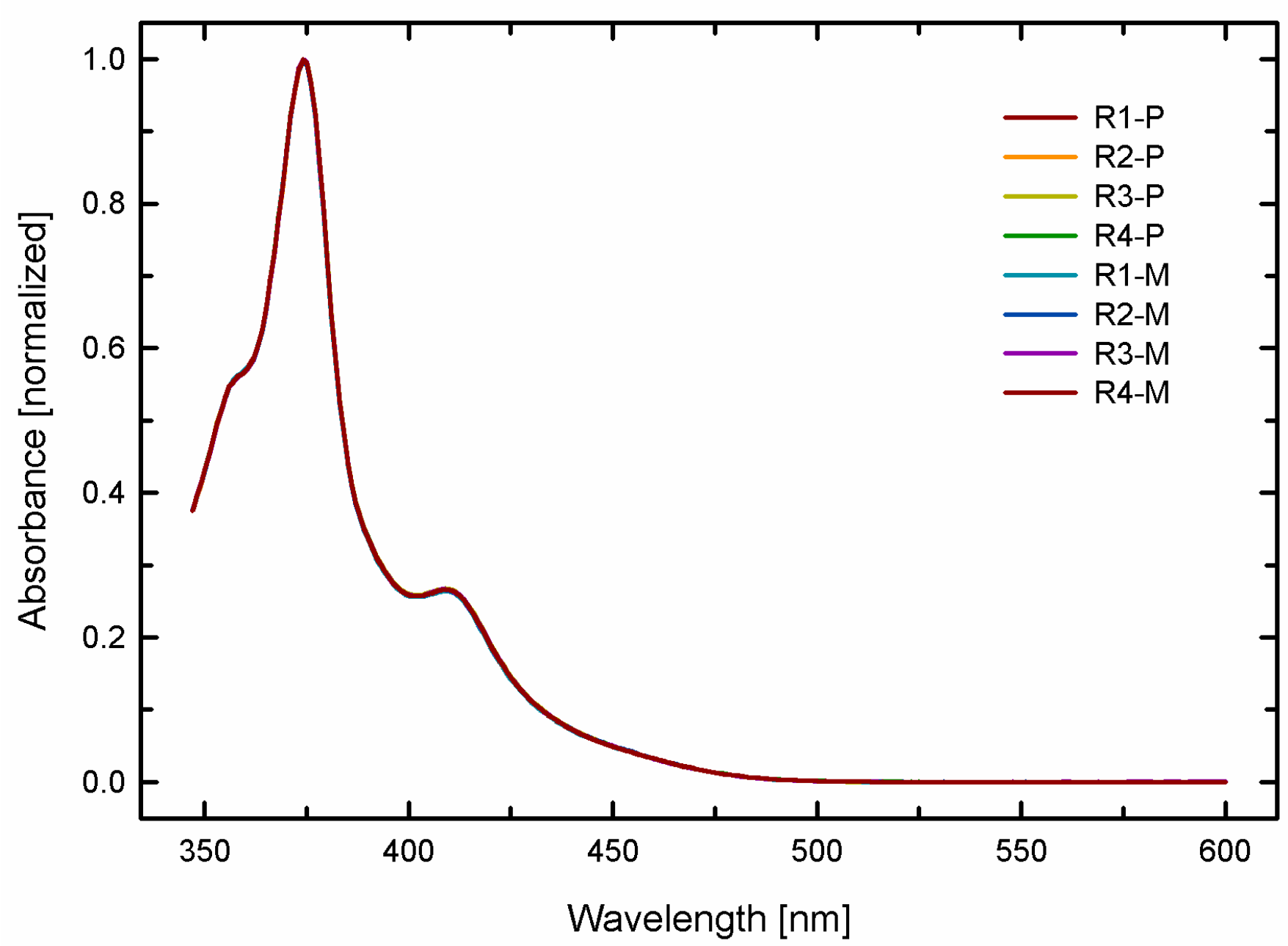

Figure S62: Absorption spectra of solutions used to prepare spin-coating recipe's R1-R6.

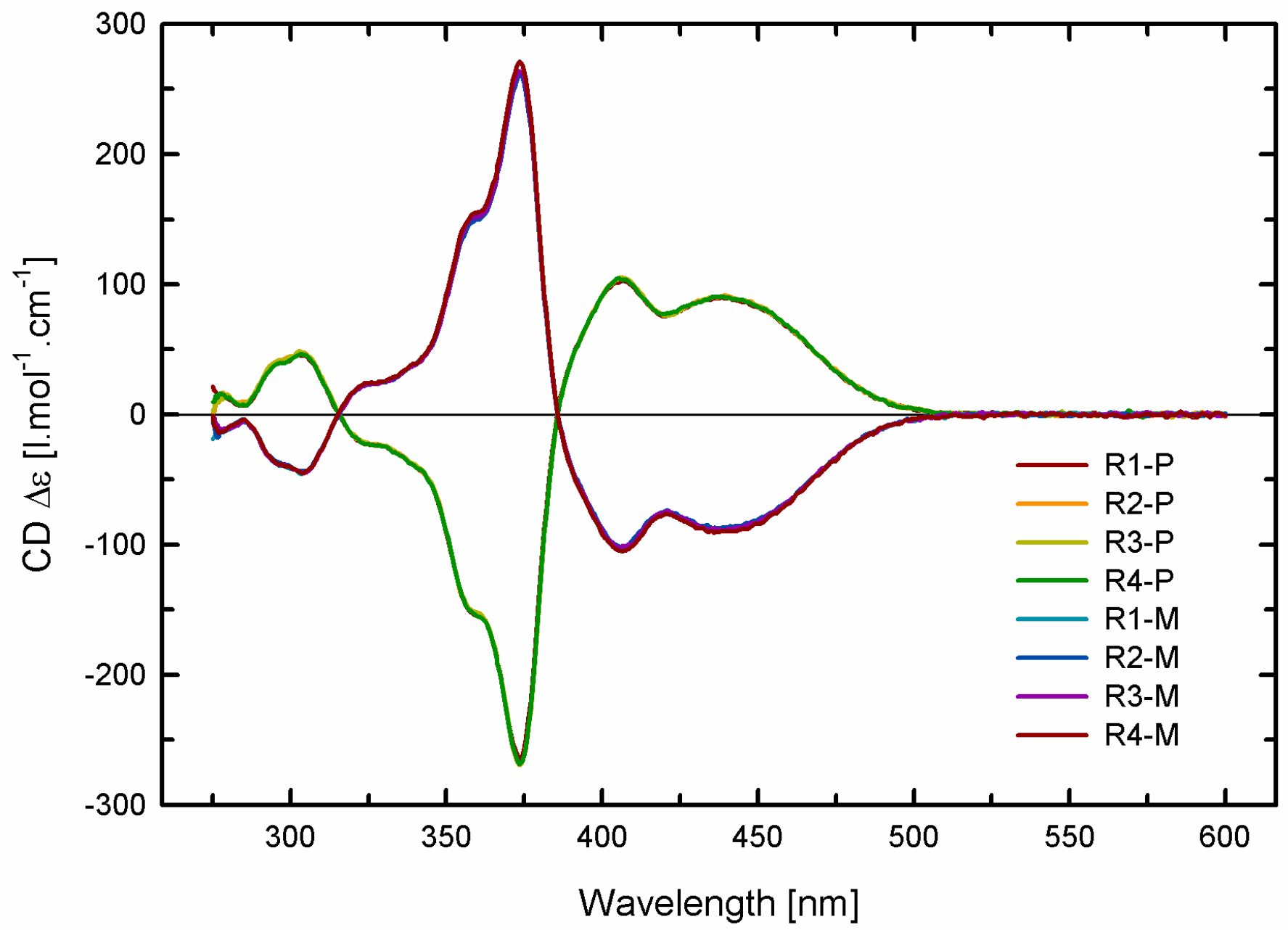

Figure S63: Circular dichroism spectra of solutions used to prepare spin-coating recipe's R1-R6.

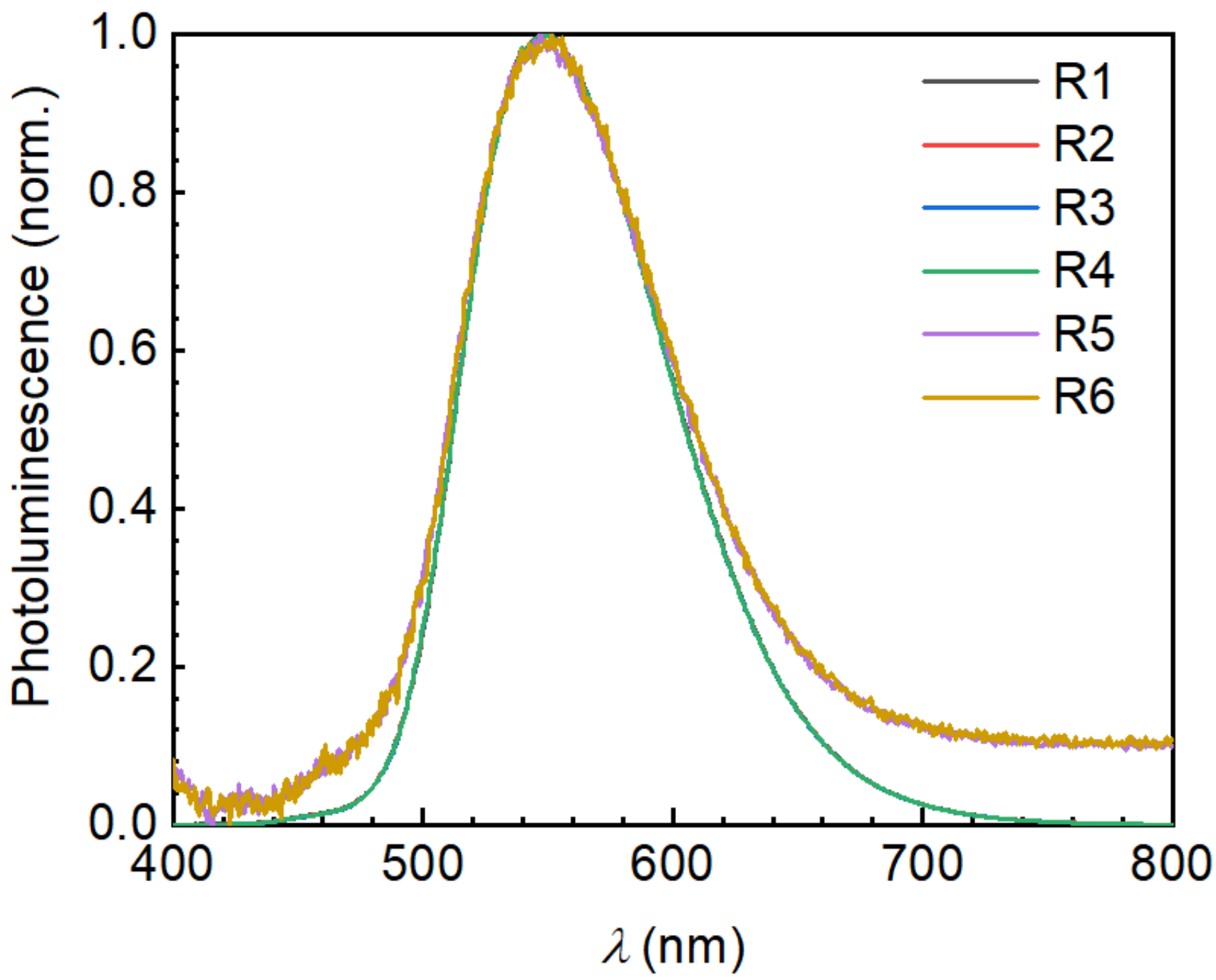

Figure S64: Steady state photoluminescence spectra of solutions used to prepare spin-coating recipe's R1-R6.

Solid State Optical Characterisation

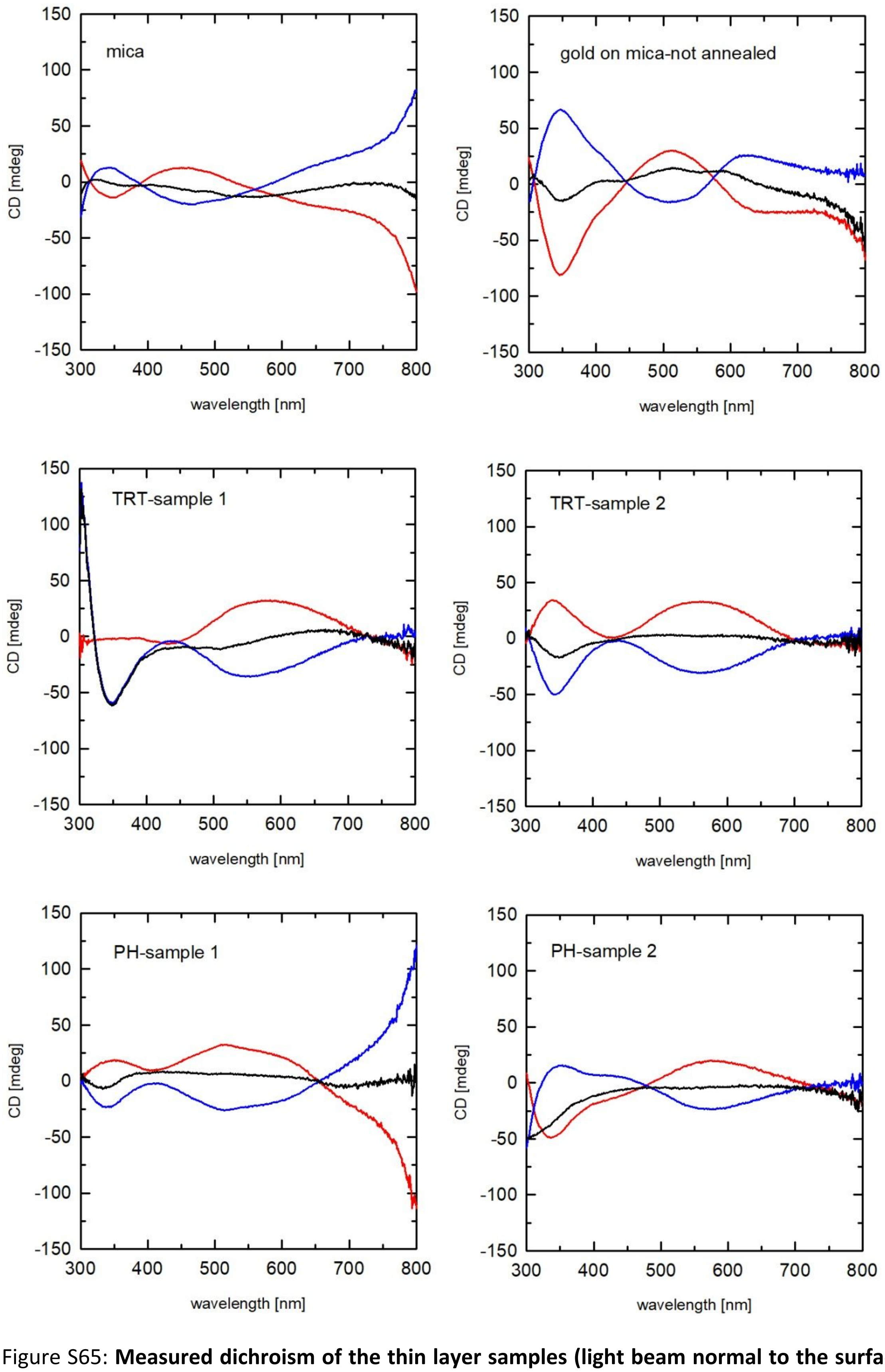

Figure S65: **Measured dichroism of the thin layer samples (light beam normal to the surface).** Red and blue lines indicate measurements taken on the same sample rotated by 90 degrees around the

normal; black line is the sum of blue and red trace. Mica: signal of mica substrate plate; gold on mica: an example of signal of a gold layer deposited on mica substrate.

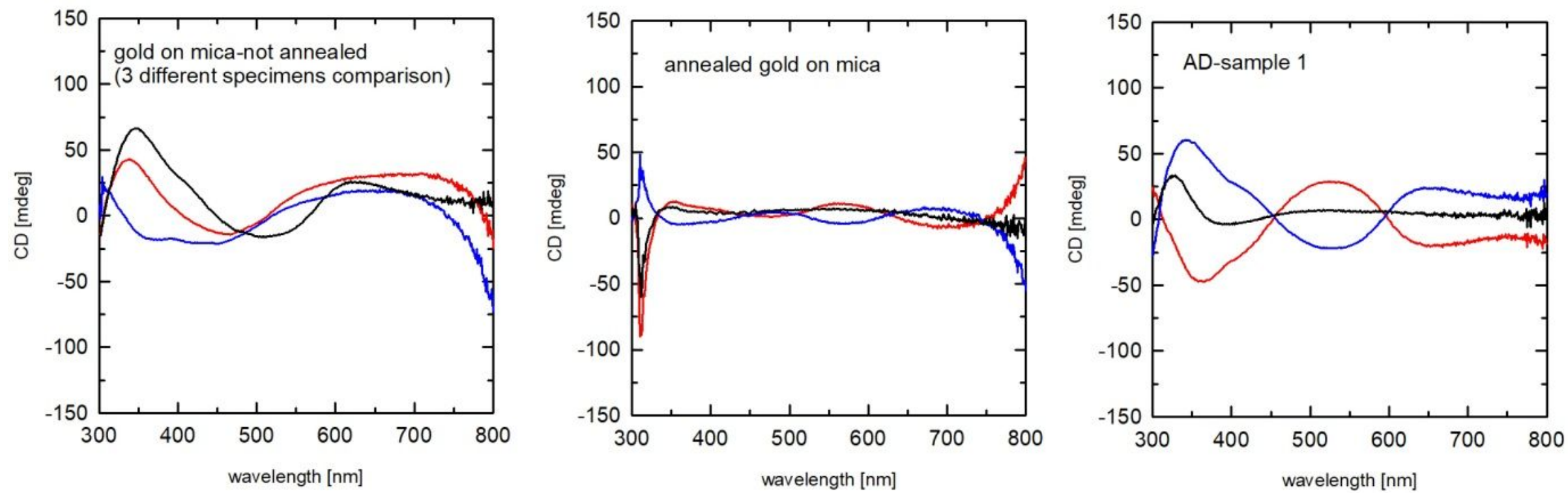

Figure S66: Left pane: comparison of three different samples of gold layers on mica substrate (same orientation). Central paned right: annealed gold layer on mica and AD1 sample, respectively. As in Fig.S60, red and blue correspond to 90 degree rotation and black is the sum.

XPS

The spectra of the TRT-SAM before after each annealing step can be found in the figure below.

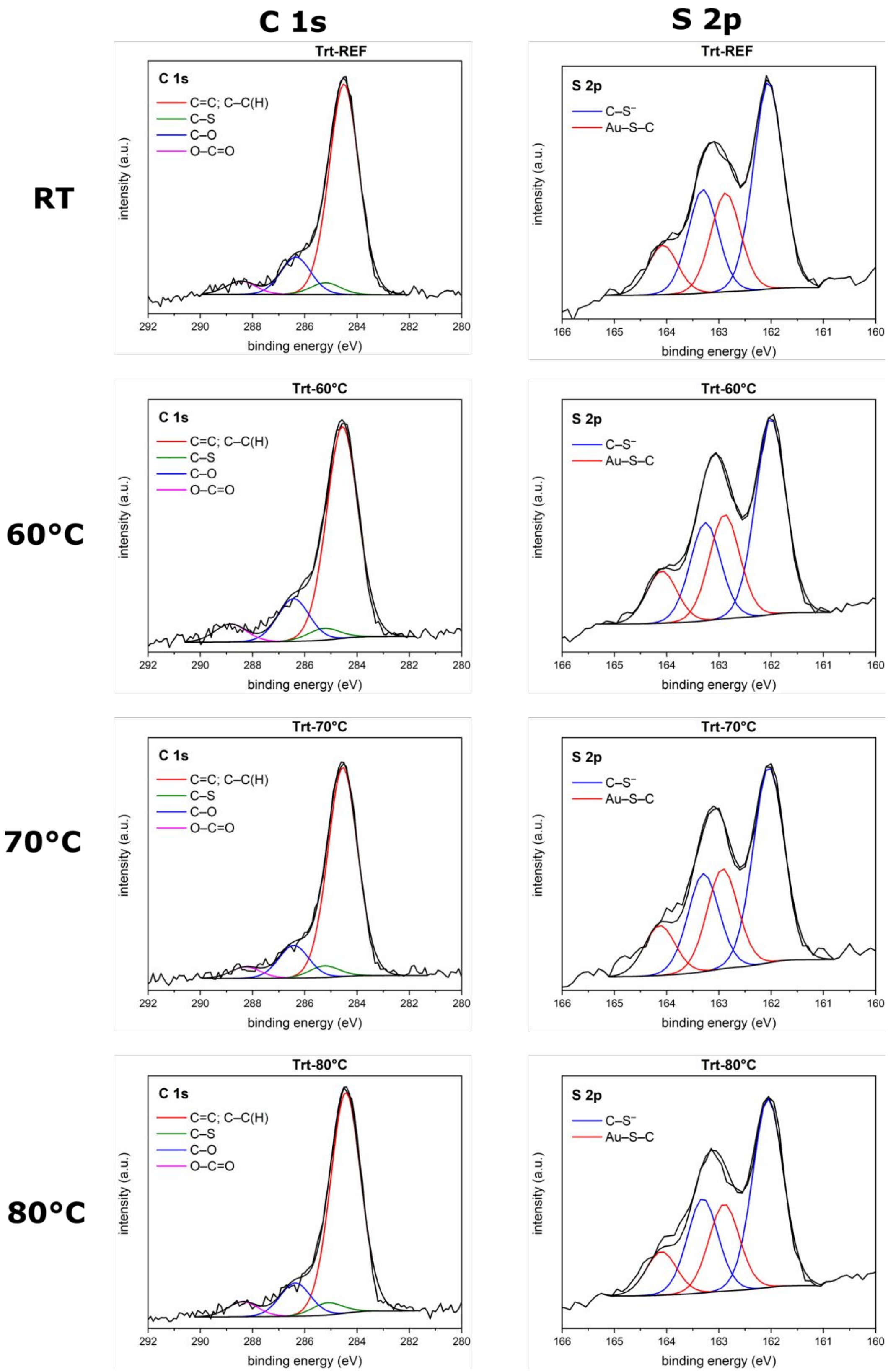

Figure S67: XPS Spectra of TRT-SAM after annealing at various temperatures.

NMR Spectroscopy

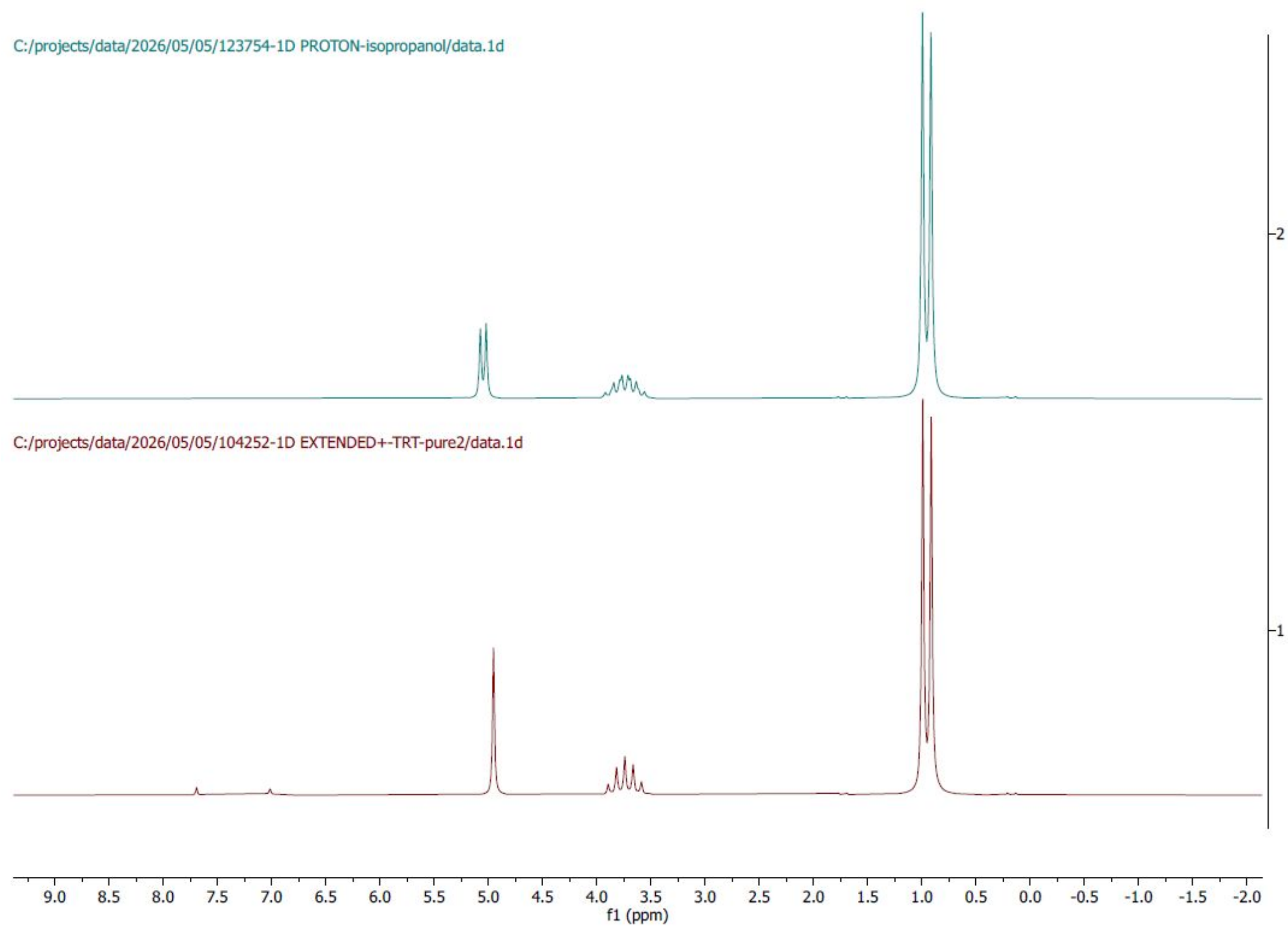

Figure S68: NMR measurements showing the presence of the solvent and two small peaks from the tritylthiol without any impurities (limited by solubility).

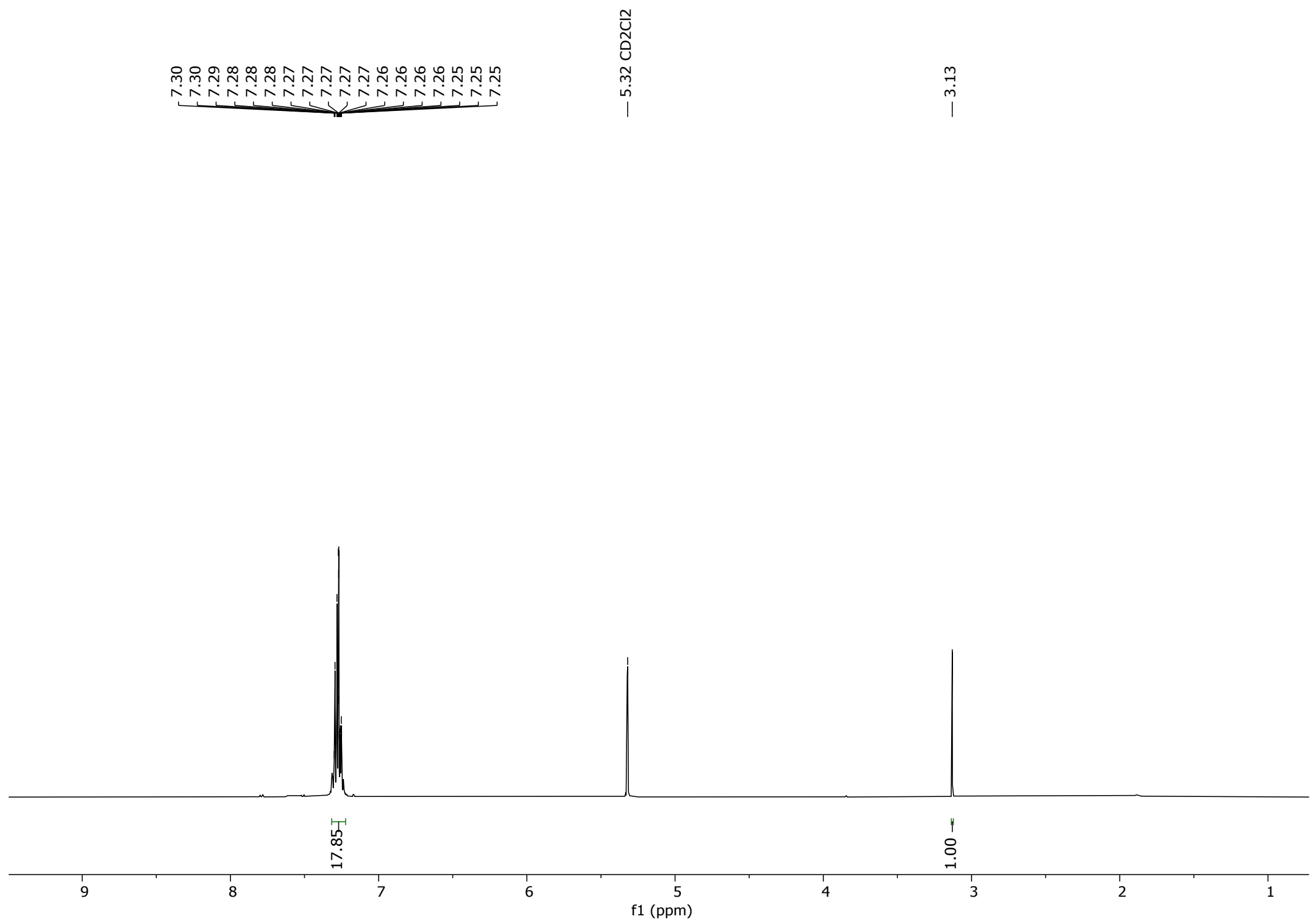

Figure S69: ^{1}H-NMR of tritylthiol. Small impurities are observed, the ratio of the integrals of the aromatic carbon (~7.3 ppm) vs. the thiol-bound proton (3.13 ppm) indicates a slightly lower ratio than expected, *i.e.* some decomposition or aromatic impurities are present.

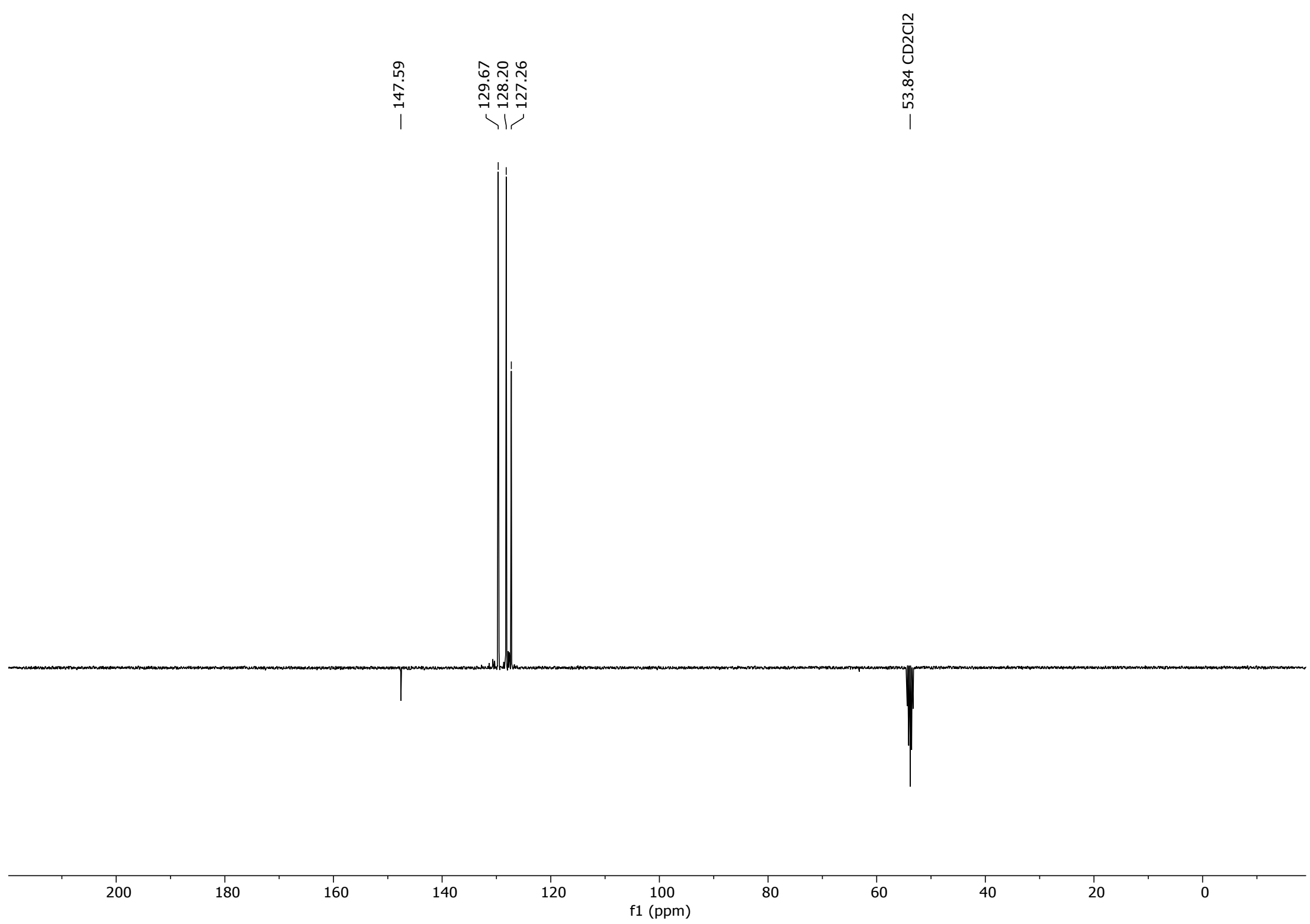

Figure S70: ^{13}C-NMR of tritylthiol. Small impurities are observed. On the contrary of what is indicated by XPS measurements, no carbonyl or similar impurities were observed, i.e. these must come from an adventitious carbon source.

Computational Results

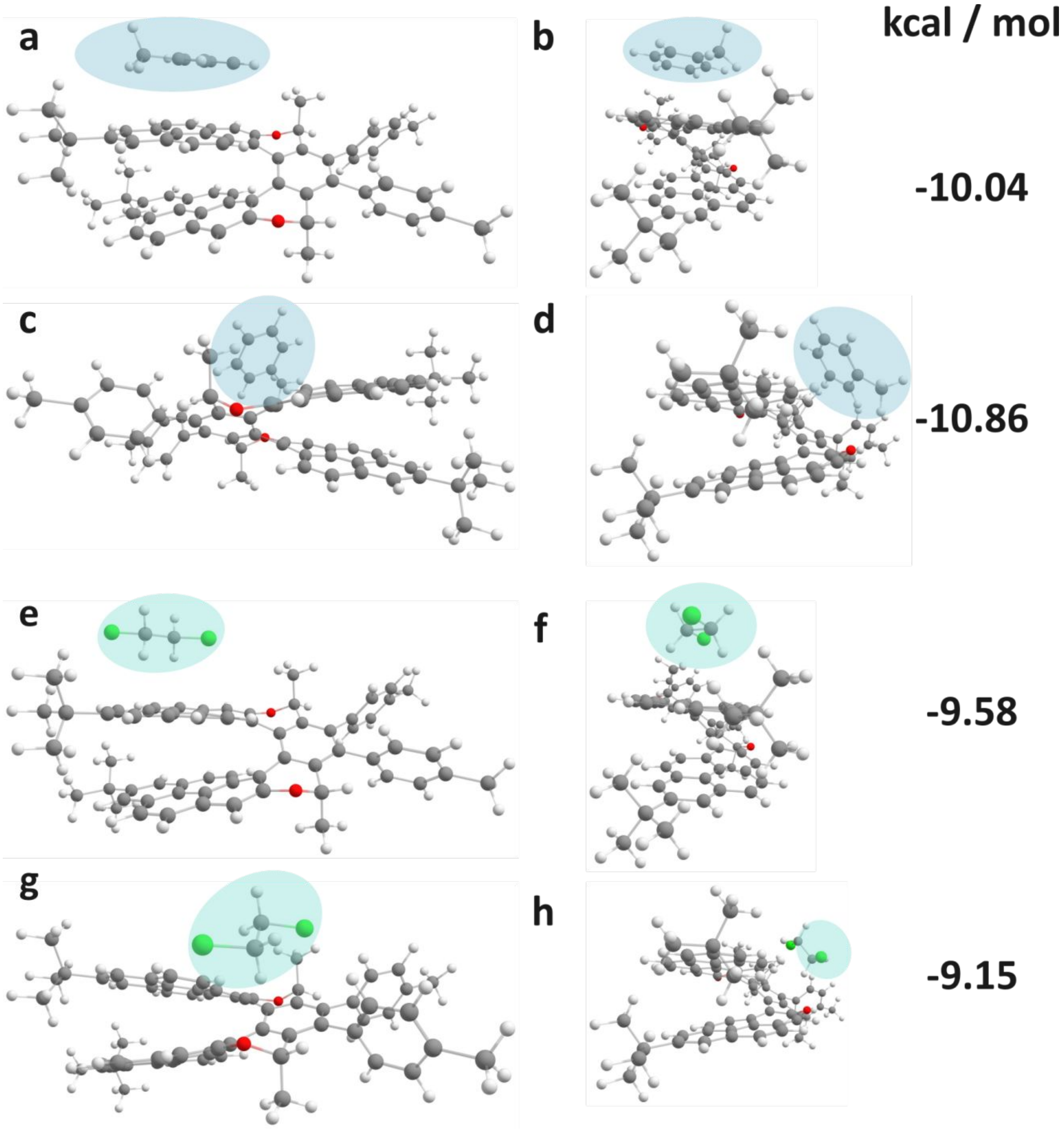

Figure S71: Interaction Energies with a single molecule of each solvent. Conformational space sampled via CREST, reoptimized by ωB97X-V def2-SVP, energies calculated via ωB97X-V def2-TZVP with CP correction. Solvent molecule highlighted by elliptical shape. (a,b) CREST minimum with toluene (face-on or conventional π-stack), (c,d) next lowest CREST result with distinct interaction motif (edge-on or T-stack; **DFT Minimum**).(e,f) CREST minimum with DCE (~face-on, **DFT minimum**), (g,h) next lowest CREST result with distinct interaction motif (interaction with the bridge-part of the helicene)

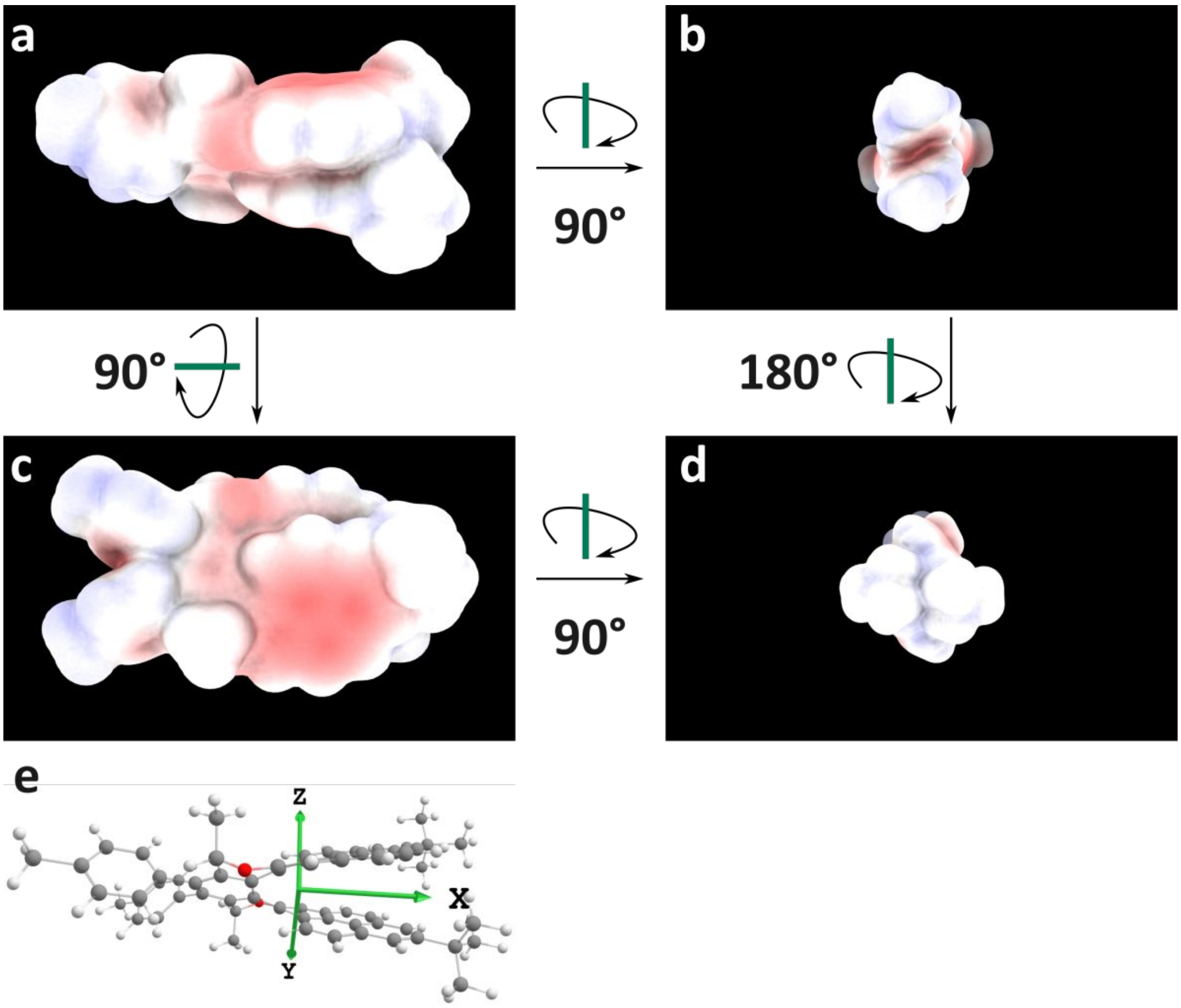

Figure S72: ESP surface of Helicene from various viewpoints. (a) Side view of pyrene-system which corresponds to edge-on helicene and (c) face-on helicene from top-view. View of the narrow side of the helicene from the (b) toluene and (d) tert-butyl stubstituents. (e) Structure and orientation of axes in our calculations (relevant for calculated dipole moments orientation).

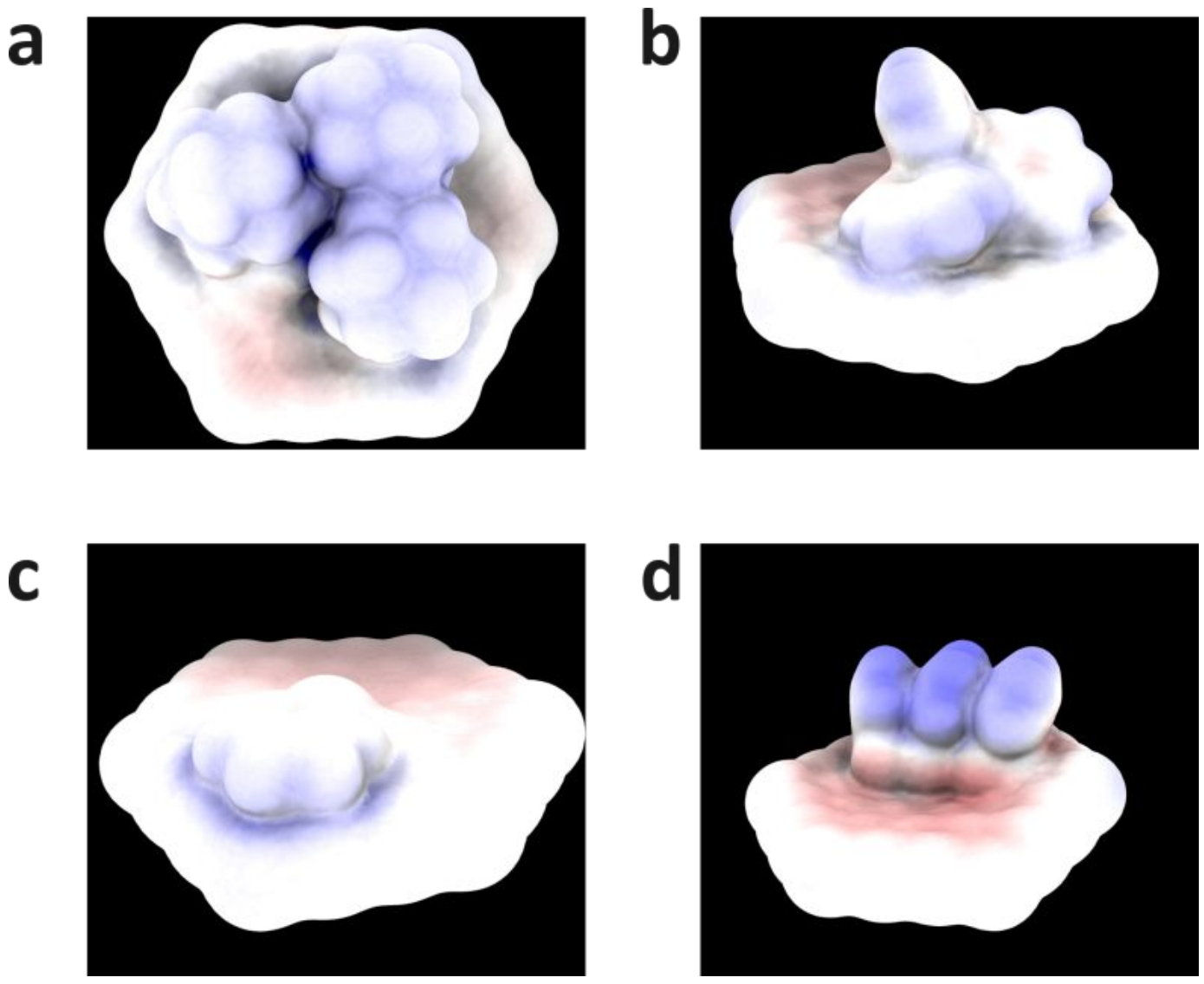

Figure S73: Results of additional ESP-calculations. (a) Three adamantyl-units, (b) side view of trityl, (c) phenyl optimized at non-idea angle results in face-on orientation, while (d) three phenylthiols form edge-on oriented conformer (~ situation in PH-SAM). When the outermost phenylthiols are removed and the single molecule is reoptimized, the edge-on orientation remains.

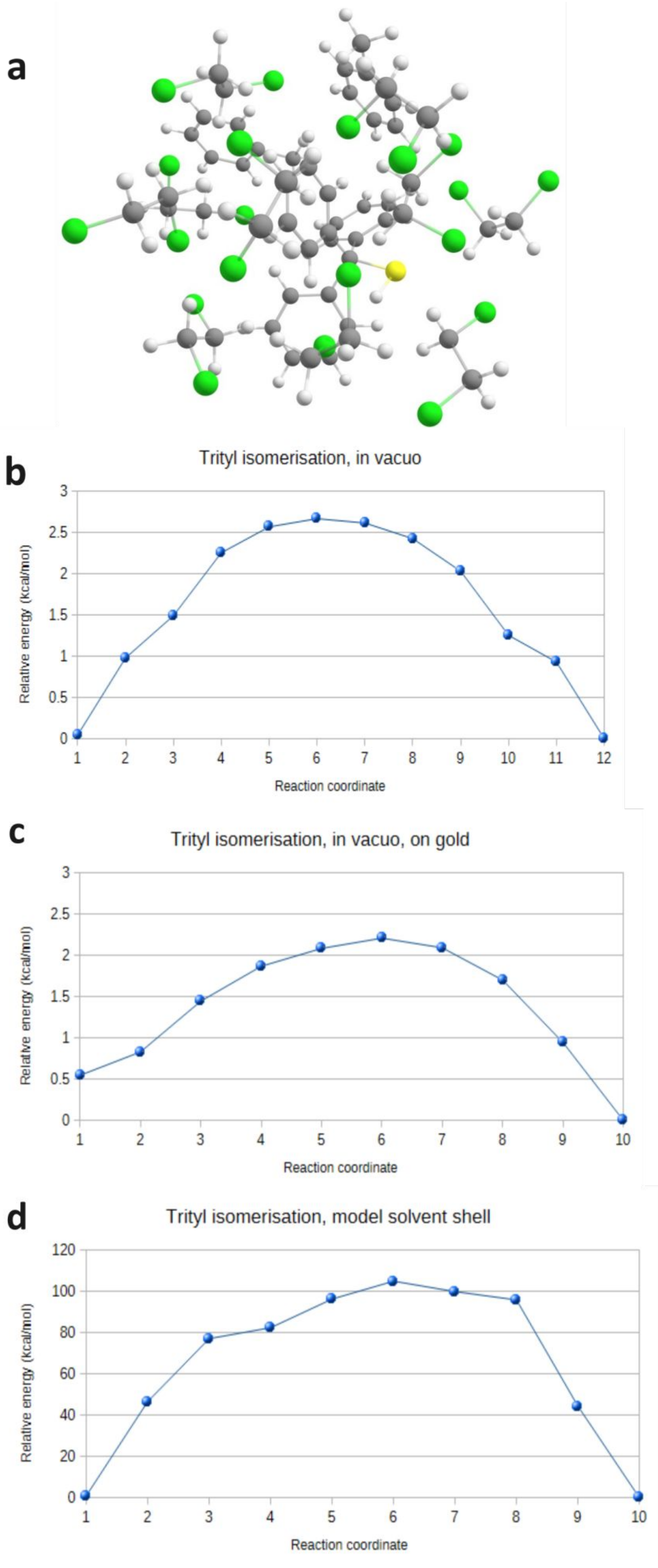

Figure S74: Molecule forming the Trityl-SAM and its isomerisation. (a) Conformation in solvent shell, (b) in vacuo, (c) in vacuo on gold 111, (d) in solvent shell (note: different scale).

SEM

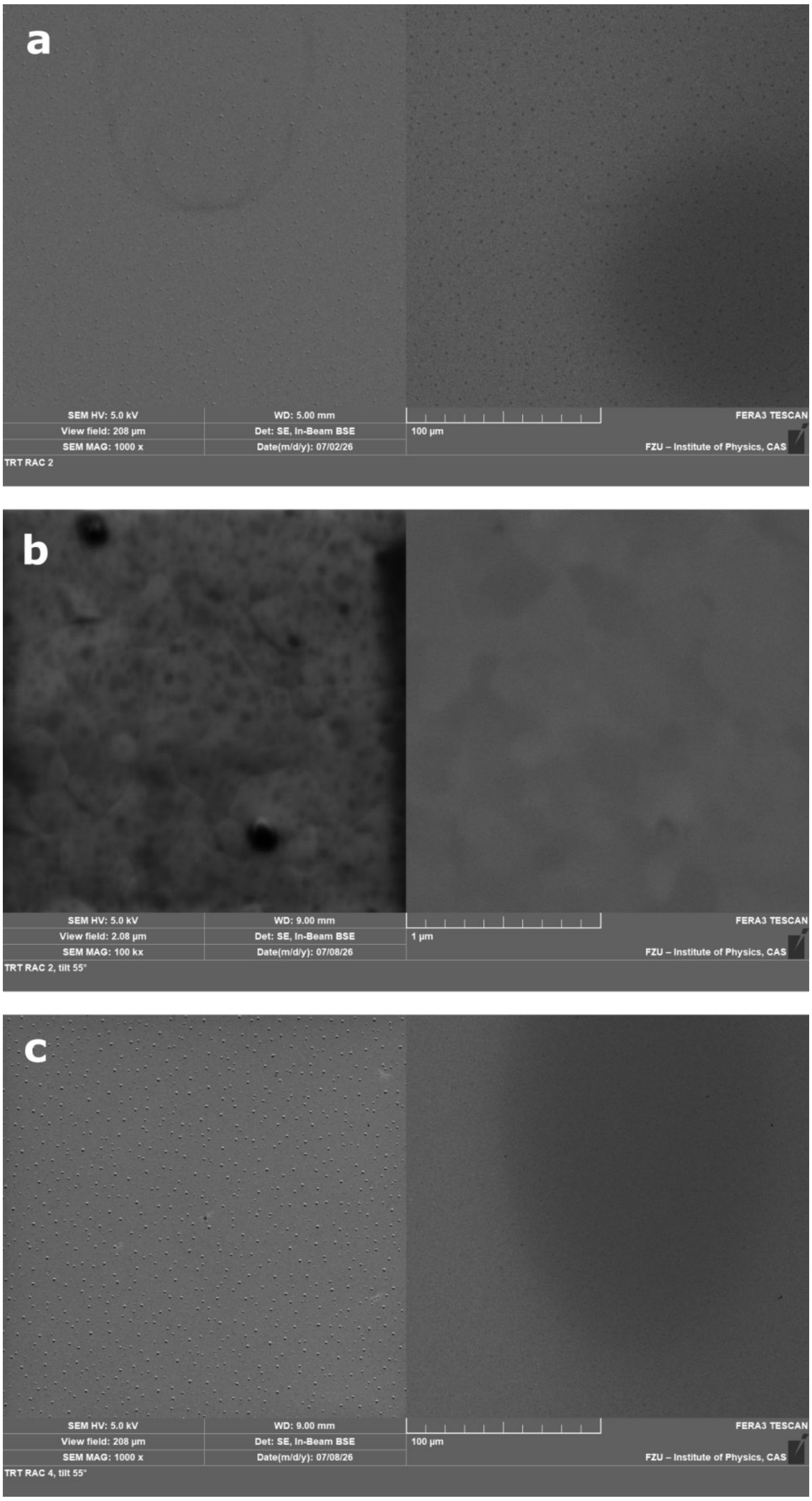

Figure S75: SEM images of the samples TRT-R 2 and TRT-R 4. (a,b) SEM images of TRT-R2 in regular geometry and tilted by 55° to visualize mushroom like structures. (c) SEM image of TRT-R4 tilted by 55°. More detailed images were not possible to acquired as substantial drift was encountered, which proved impossible to suppress.

Literature

(1) Górczyńska, S.; Brzdonkiewicz, A.; Jelecki, M.; Czapik, A.; Stasiak, B.; Kwit, M. Trityl-Containing Alcohols—An Efficient Chirality Transmission Process from Inductor to the Stereodynamic Propeller and Their Solid-State Structural Diversity. *Molecules* **2020**, *25* (3), 707. https://doi.org/10.3390/molecules25030707.

(2) Elemans, J. A. A. W.; Lei, S.; De Feyter, S. Molecular and Supramolecular Networks on Surfaces: From Two-Dimensional Crystal Engineering to Reactivity. *Angew Chem Int Ed* **2009**, *48* (40), 7298–7332. https://doi.org/10.1002/anie.200806339.

(3) Bendzińska-Berus, W.; Jelecki, M.; Kwit, M.; Rychlewska, U. Transfer of Chirality in *N* -Triphenylacetylamino Acids and Chiral Derivatives of *N* -Triphenylacetyl Gly–Gly Dipeptide and Control of Their Assembly with Steric Constraints. *CrystEngComm* **2019**, *21* (22), 3420–3430. https://doi.org/10.1039/C9CE00429G.

(4) Czernek, J.; Brus, J. Revisiting the Most Stable Structures of the Benzene Dimer. *IJMS* **2024**, *25* (15), 8272. https://doi.org/10.3390/ijms25158272.

(5) Huang, L.; Wang, T.; Li, Y.; Wang, P.; Tian, R.; Zhou, B.; Yao, L. Chiral Helically Grooved Gold Nanoarrows for Concurrently Enhancing Oxygen and Hydrogen Evolution from Electrochemical Water Splitting. *Sci. China Chem.* **2024**, *67* (11), 3767–3776. https://doi.org/10.1007/s11426-024-2136-1.

(6) Cao, H.; Minoia, A.; De Cat, I.; Seibel, J.; Waghray, D.; Li, Z.; Cornil, D.; Mali, K. S.; Lazzaroni, R.; Dehaen, W.; De Feyter, S. Hierarchical Self-Assembly of Enantiopure and Racemic Helicenes at the Liquid/Solid Interface: From 2D to 3D. *Nanoscale* **2017**, *9* (45), 18075–18080. https://doi.org/10.1039/C7NR06700C.
