# Supplementary material for: Self-Assembled Monolayers and Solvent Composition Control the Orientation and Work Function of Pyrene-Based [7]Helicenes
Source: Langmuir. 2026 Sep 29;42(40):29622–37. doi: 10.1021/acs.langmuir.6c01280 (PMC13674279; doi:10.1021/acs.langmuir.6c01280)
Supplement: Supplementary file 3 [file la6c01280_si_003.pdf]

Supplementary information III:

Self-Assembled Monolayers and Solvent Composition Control Orientation and Work Function of Pyrene-Based [7]Helicenes

Ivan Alic[a+], Katsiaryna Kutsenka[b+], Kristián Kříž[a], Lukáš Kalina[c], David Bína[a,d], Jan Hanus[b], Jaromír Kopeček[e], Anne de la Pena Lopez[a], Radek Litvín[a,d], Milan Mašát*[f], Eva Kaletová[f], Dominik Farka*[a,b]

[a] Department of Chemistry, Faculty of Science, University of South Bohemia in České Budějovice (JU), Branišovská 1760, 370 05 České Budějovice, Czech Republic.
[b] Functional Molecules Group, Institute of Organic Chemistry and Biochemistry (IOCB), Czech Academy of Sciences (CAS); Flemingovo Náměstí 542/2, 160 00 Prague, Czech Republic.
[c] Faculty of Chemistry, Brno University of Technology (BUT), Purkyňova 118, CZ-612 00 Brno, Czech Republic
[d] Institute of Plant Molecular Biology, Biology Centre, Czech Academy of Sciences, Branišovská 31, 370 05 České Budějovice, Czech Republic
[e] Department of Materials Analysis, Institute of Physics (FZU) of Czech Academy of Sciences (CAS); Na Slovance 2, 182 00 Prague 8, Czech Republic.
[f] Molecular Devices Group, Institute of Organic Chemistry and Biochemistry (IOCB), Czech Academy of Sciences (CAS); Flemingovo Náměstí 542/2, 160 00 Prague, Czech Republic.

*corresponding author
[+] These authors contributed equally to the work

Number of pages: 48
Number of figures: 46
Number of schemes:0
Number of tables: 0

Table of Contents:
S3 Script-based quantification of mechanical features for racemate measurements

Mechanical Features and applied masks.

All the below data were generated via our in-house created Python script.
All the below figures relate to a single QNM-AFM measurement. Each consists of 3 rows of three images. From left to right, the row consists of:
Row 1: Deformation, Dissipation, and deformation with Hard mask overlay (colour: teal)
Row 2: Deformation with Elastic, Plastic, and Adhesive mask, respectively (colour, teal)
Row 3: Histogram of deformation channel, histogram of dissipation channel, and box plot of all four fractions (emergent from the mask area)

The masks were generated as follows:
Hard: areas with **lowest** 25% deformation AND **lowest** 25% dissipation
Elastic: areas with **highest** 25% deformation AND **lowest** 25% dissipation
Plastic: areas with **highest** 25% deformation AND **highest** 25% dissipation

Adhesive: areas with **lowest** 25% deformation AND **highest** 25% dissipation

All images contain a header indicative of the original file-name.
All measured images were treated by a data cutoff (1.5 * interquartile range) for optimal visualization (integral to the script).

TRT- R Recipe 1

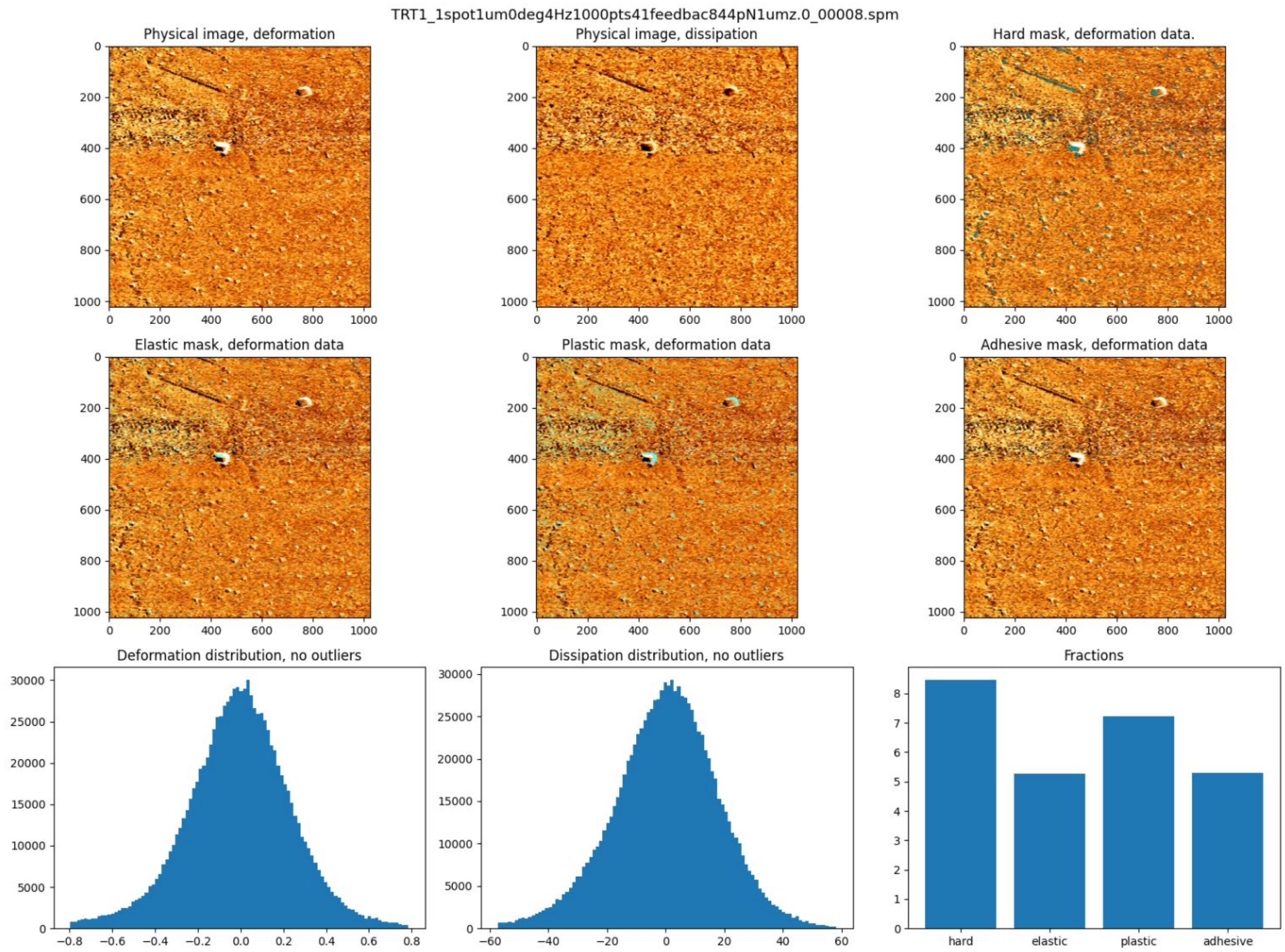

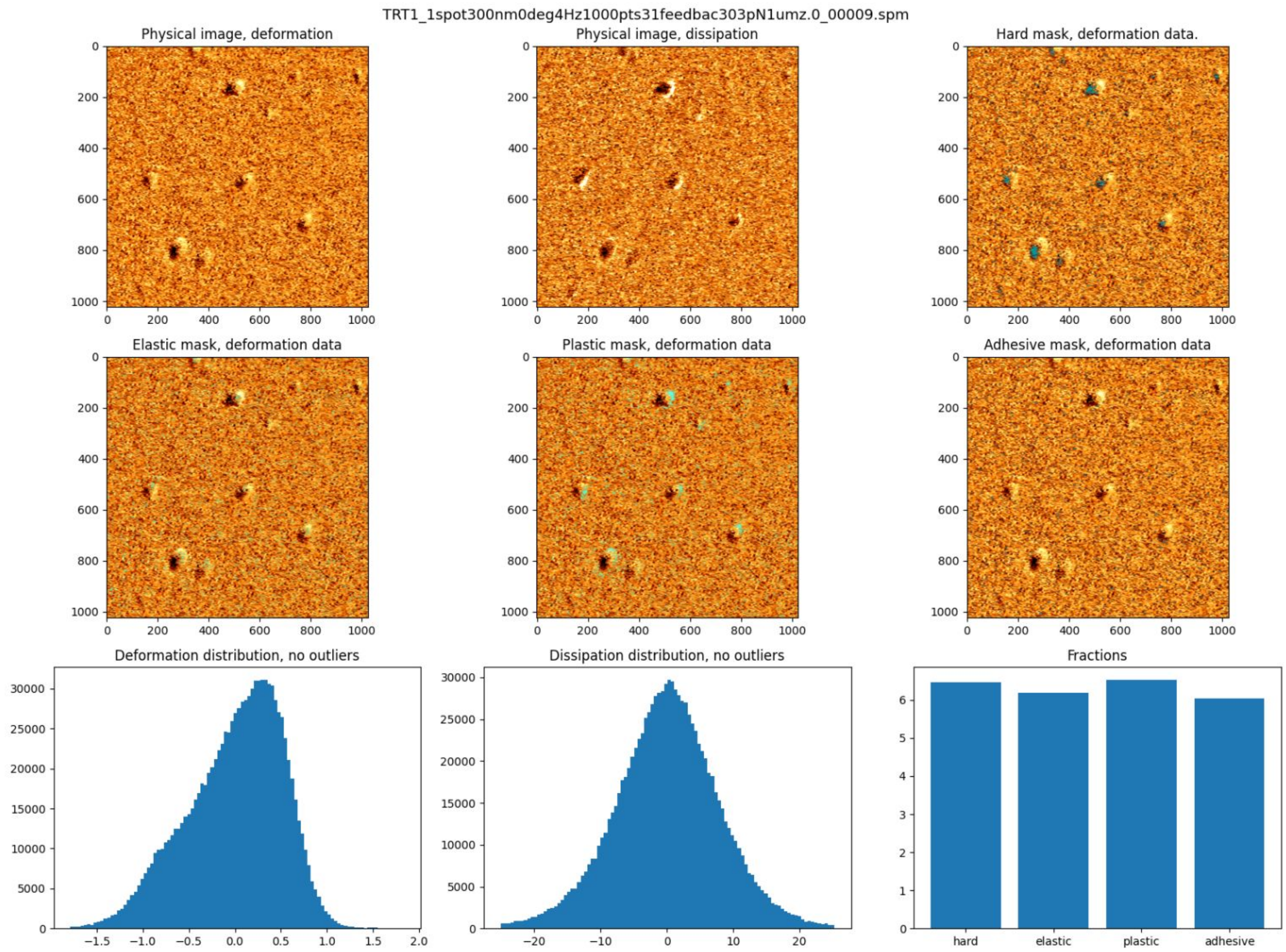
TRT1_1spot300nm0deg4Hz1000pts31feedbac303pN1umz.0_00009.spm
Physical image, deformation
Physical image, dissipation
Hard mask, deformation data.
Elastic mask, deformation data
Plastic mask, deformation data
Adhesive mask, deformation data
Deformation distribution, no outliers
Dissipation distribution, no outliers
Fractions
hard
elastic
plastic
adhesive

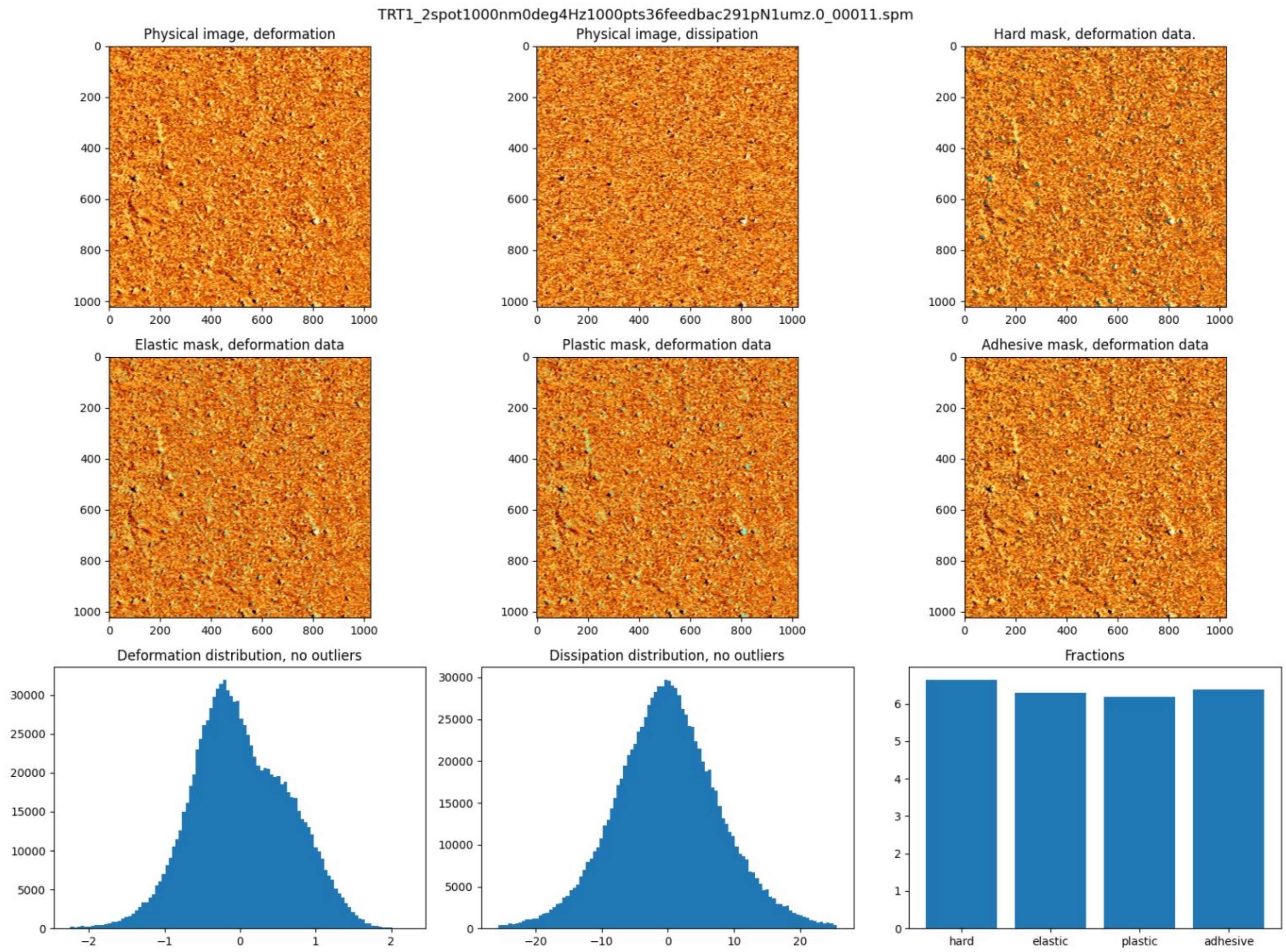

TRT1_2spot1000nm0deg4Hz1000pts36feedbac291pN1umz.0_00011.spm
Physical image, deformation
Physical image, dissipation
Hard mask, deformation data.
Elastic mask, deformation data
Plastic mask, deformation data
Adhesive mask, deformation data
Deformation distribution, no outliers
Dissipation distribution, no outliers
Fractions
hard
elastic
plastic
adhesive

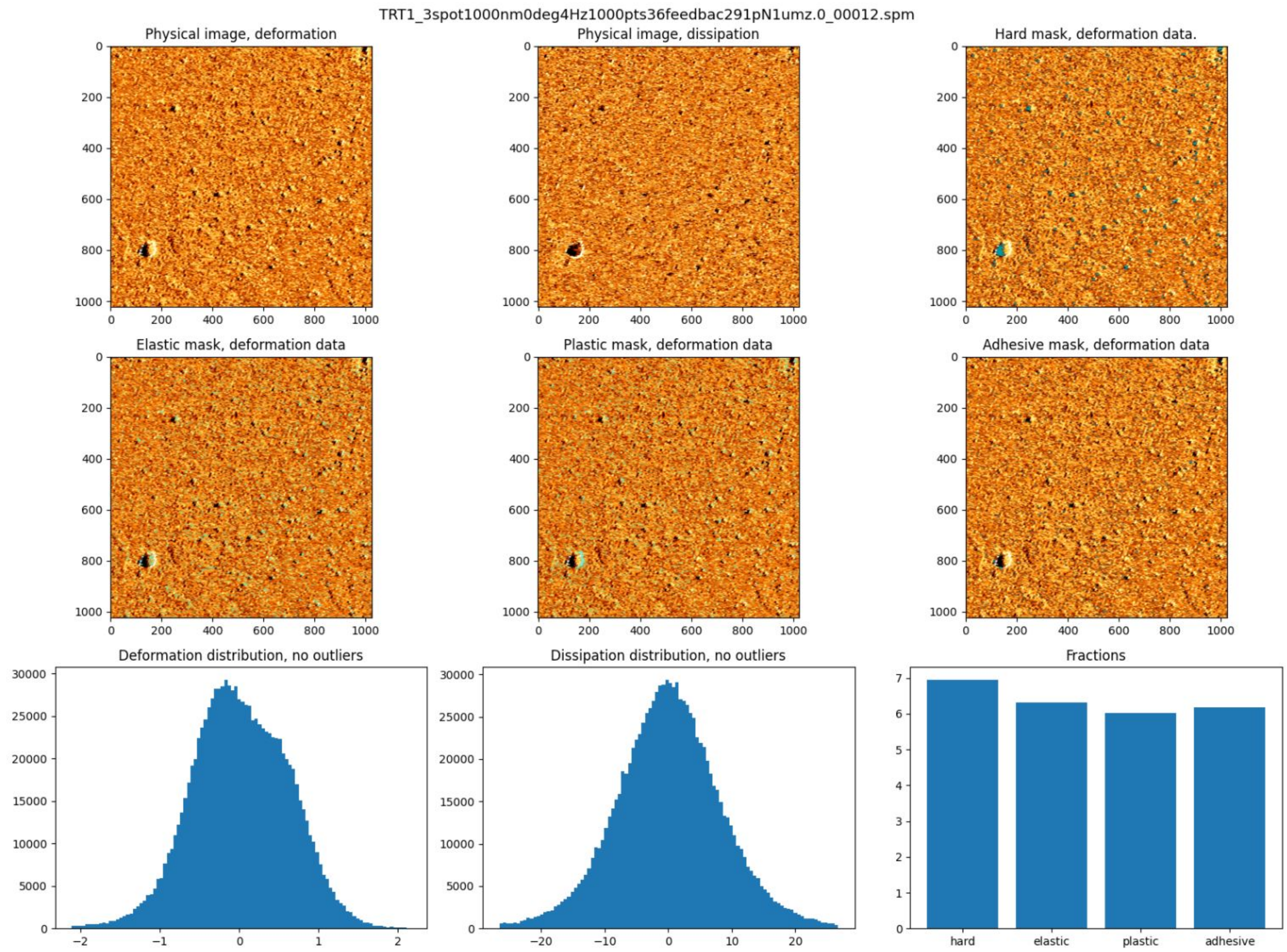
TRT1_3spot1000nm0deg4Hz1000pts36feedbac291pN1umz.0_00012.spm
Physical image, deformation
Physical image, dissipation
Hard mask, deformation data.
Elastic mask, deformation data
Plastic mask, deformation data
Adhesive mask, deformation data
Deformation distribution, no outliers
Dissipation distribution, no outliers
Fractions
hard
elastic
plastic
adhesive

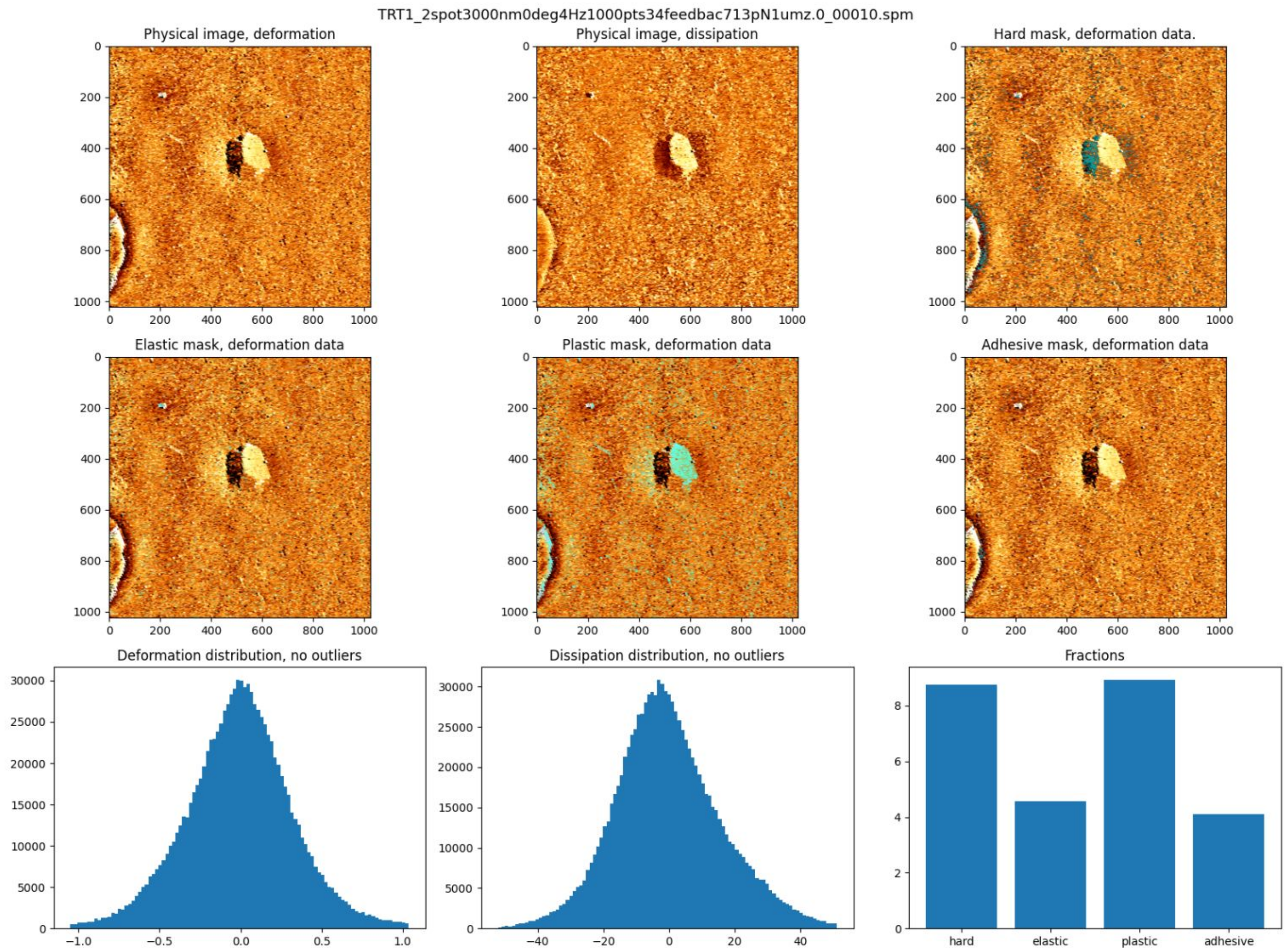

TRT1_2spot3000nm0deg4Hz1000pts34feedbac713pN1umz.0_00010.spm
Physical image, deformation
Physical image, dissipation
Hard mask, deformation data.
Elastic mask, deformation data
Plastic mask, deformation data
Adhesive mask, deformation data
Deformation distribution, no outliers
Dissipation distribution, no outliers
Fractions
hard
elastic
plastic
adhesive

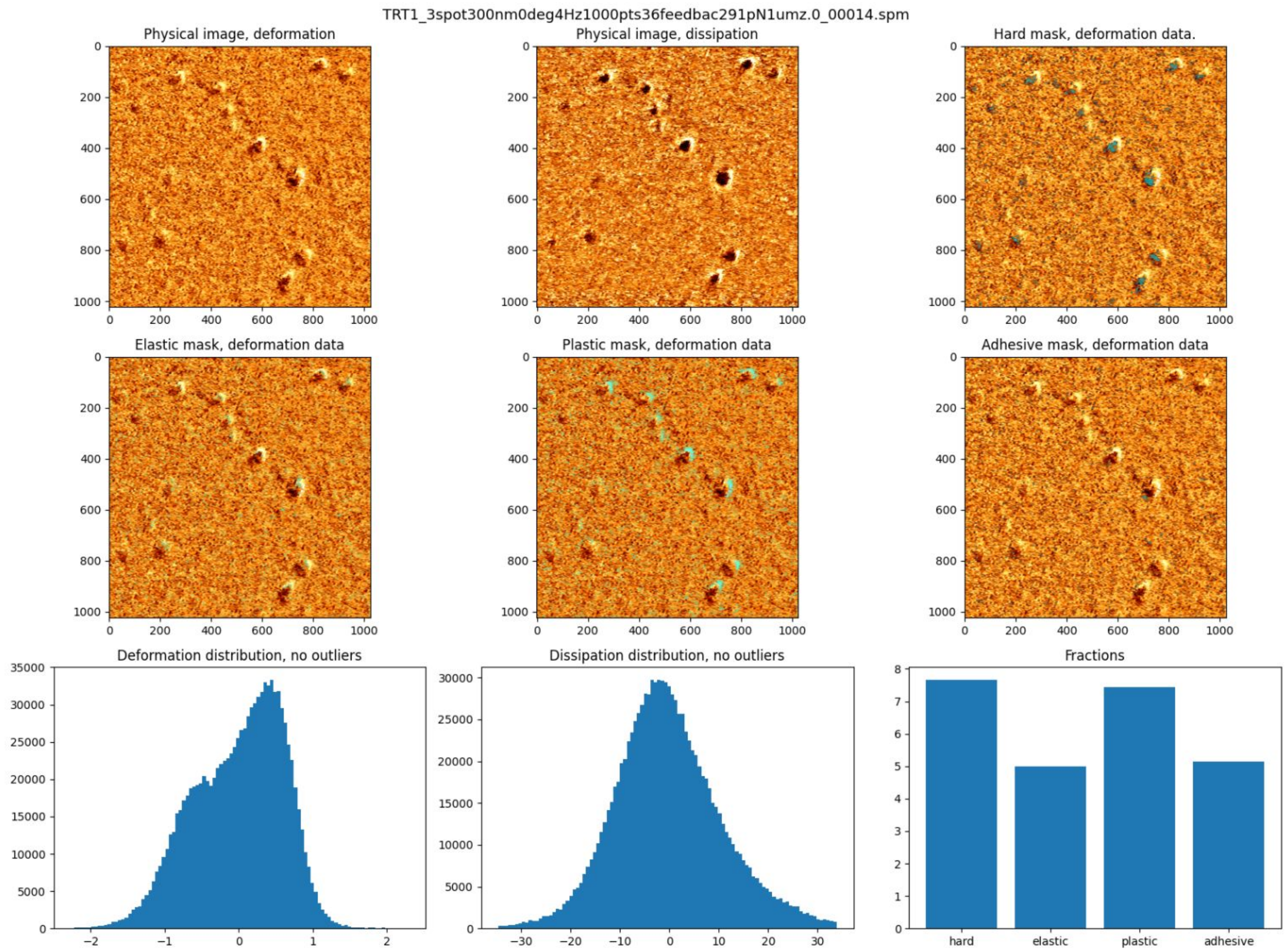

TRT1_3spot300nm0deg4Hz1000pts36feedbac291pN1umz.0_00014.spm
Physical image, deformation
Physical image, dissipation
Hard mask, deformation data.
Elastic mask, deformation data
Plastic mask, deformation data
Adhesive mask, deformation data
Deformation distribution, no outliers
Dissipation distribution, no outliers
Fractions
hard
elastic
plastic
adhesive

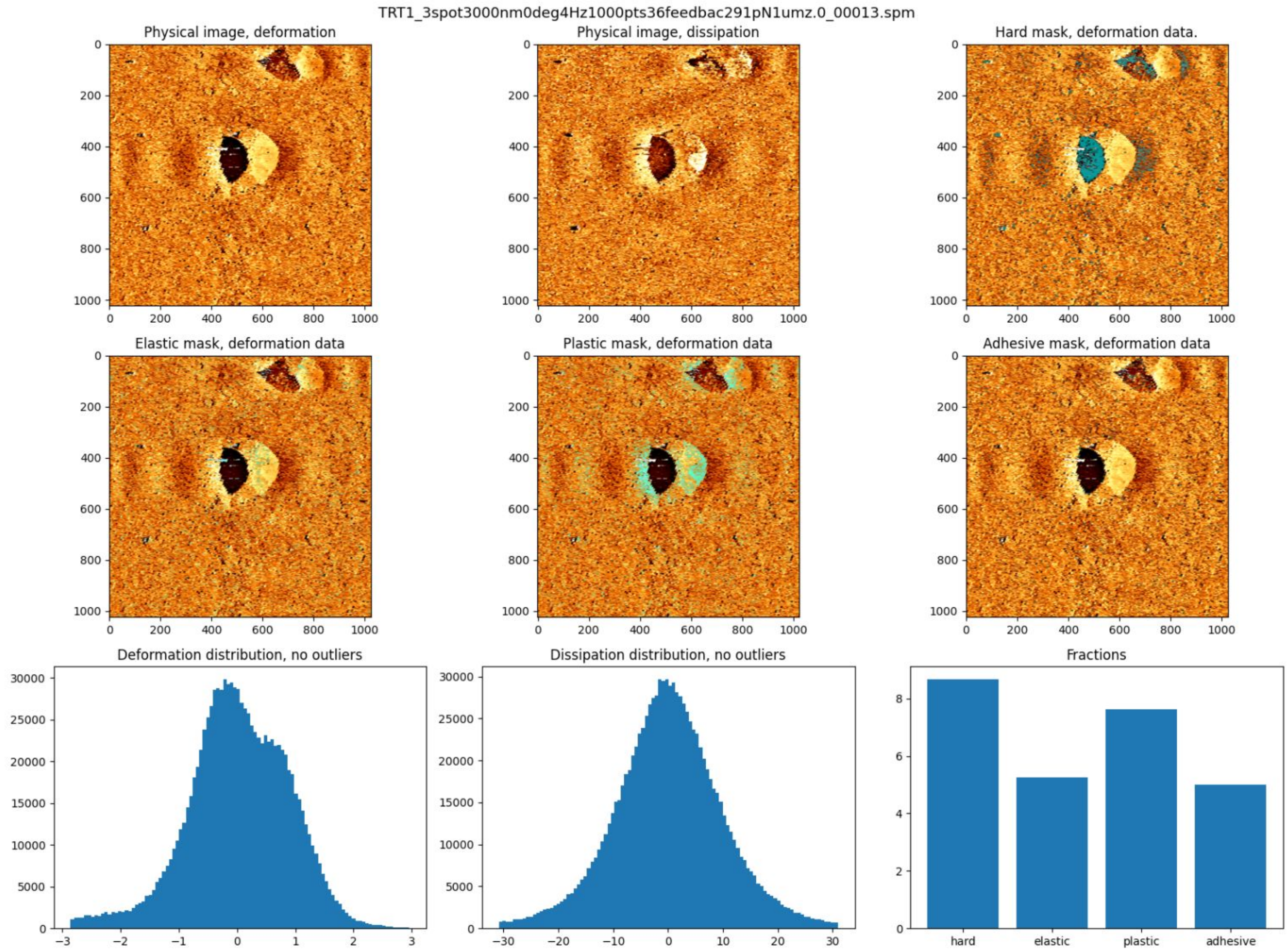
TRT1_3spot3000nm0deg4Hz1000pts36feedbac291pN1umz.0_00013.spm
Physical image, deformation
Physical image, dissipation
Hard mask, deformation data.
Elastic mask, deformation data
Plastic mask, deformation data
Adhesive mask, deformation data
Deformation distribution, no outliers
Dissipation distribution, no outliers
Fractions
hard
elastic
plastic
adhesive

TRT- R Recipe 2

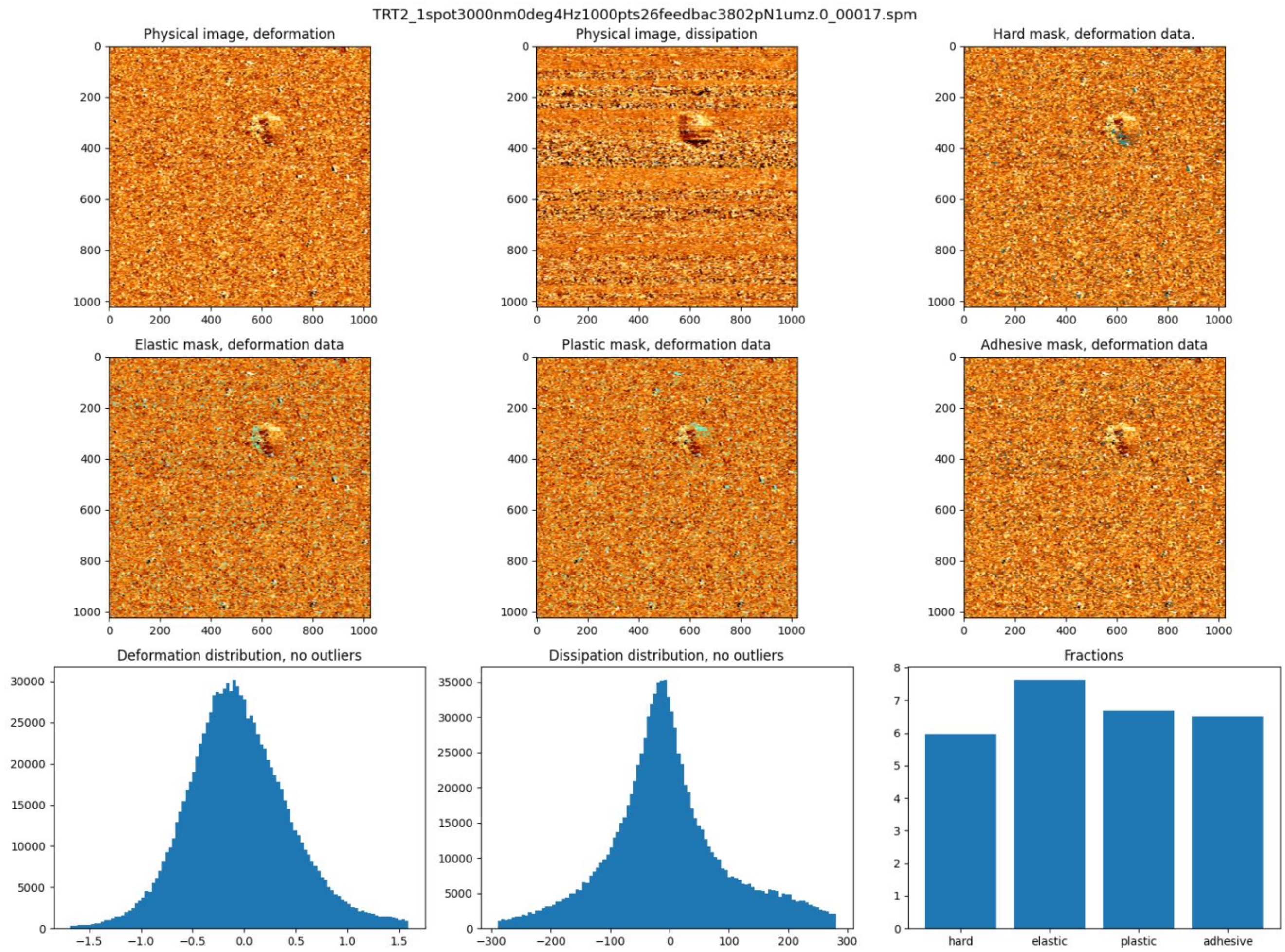

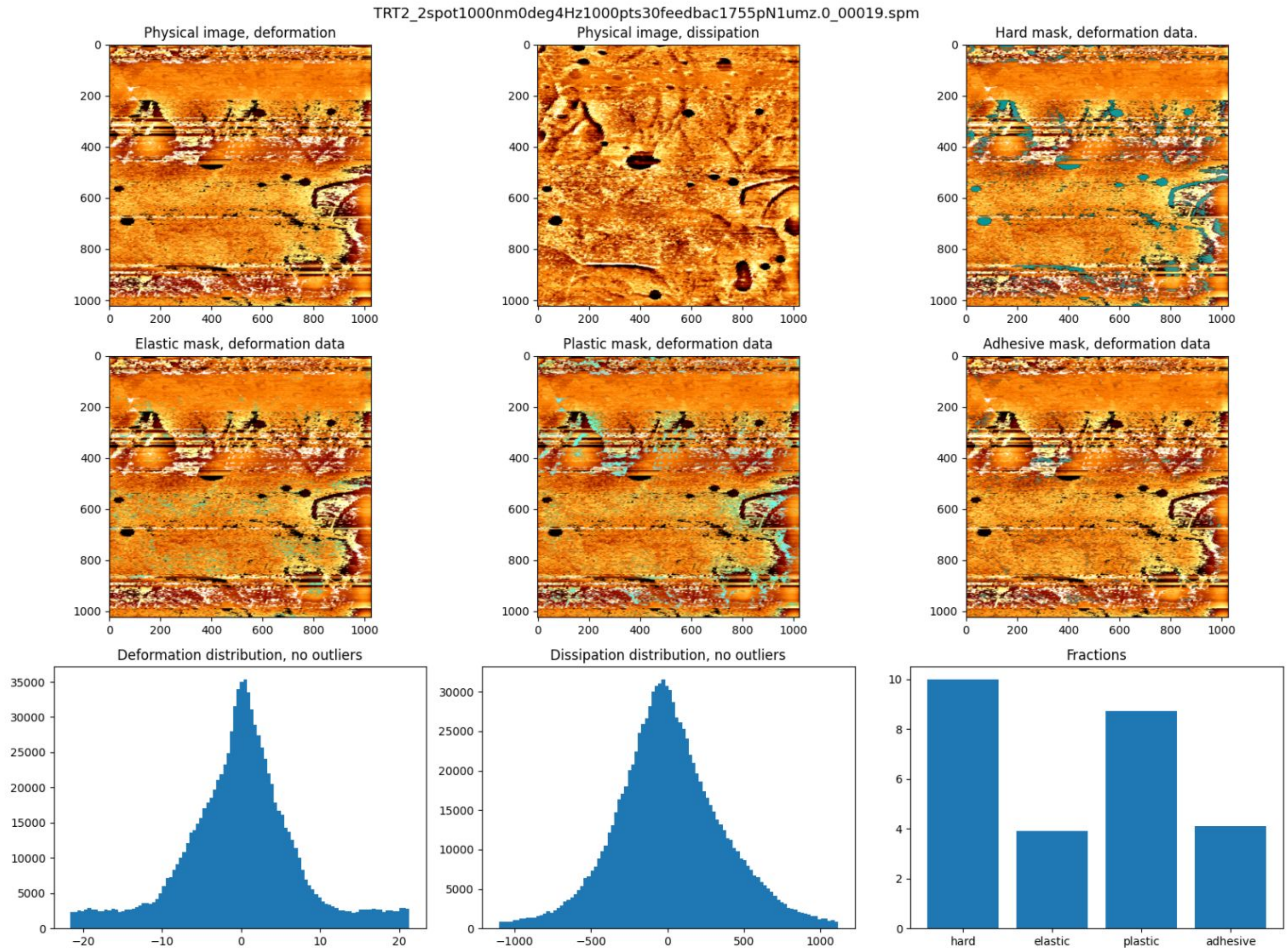
TRT2_2spot1000nm0deg4Hz1000pts30feedbac1755pN1umz.0_00019.spm
Physical image, deformation
Physical image, dissipation
Hard mask, deformation data.
Elastic mask, deformation data
Plastic mask, deformation data
Adhesive mask, deformation data
Deformation distribution, no outliers
Dissipation distribution, no outliers
Fractions
hard
elastic
plastic
adhesive

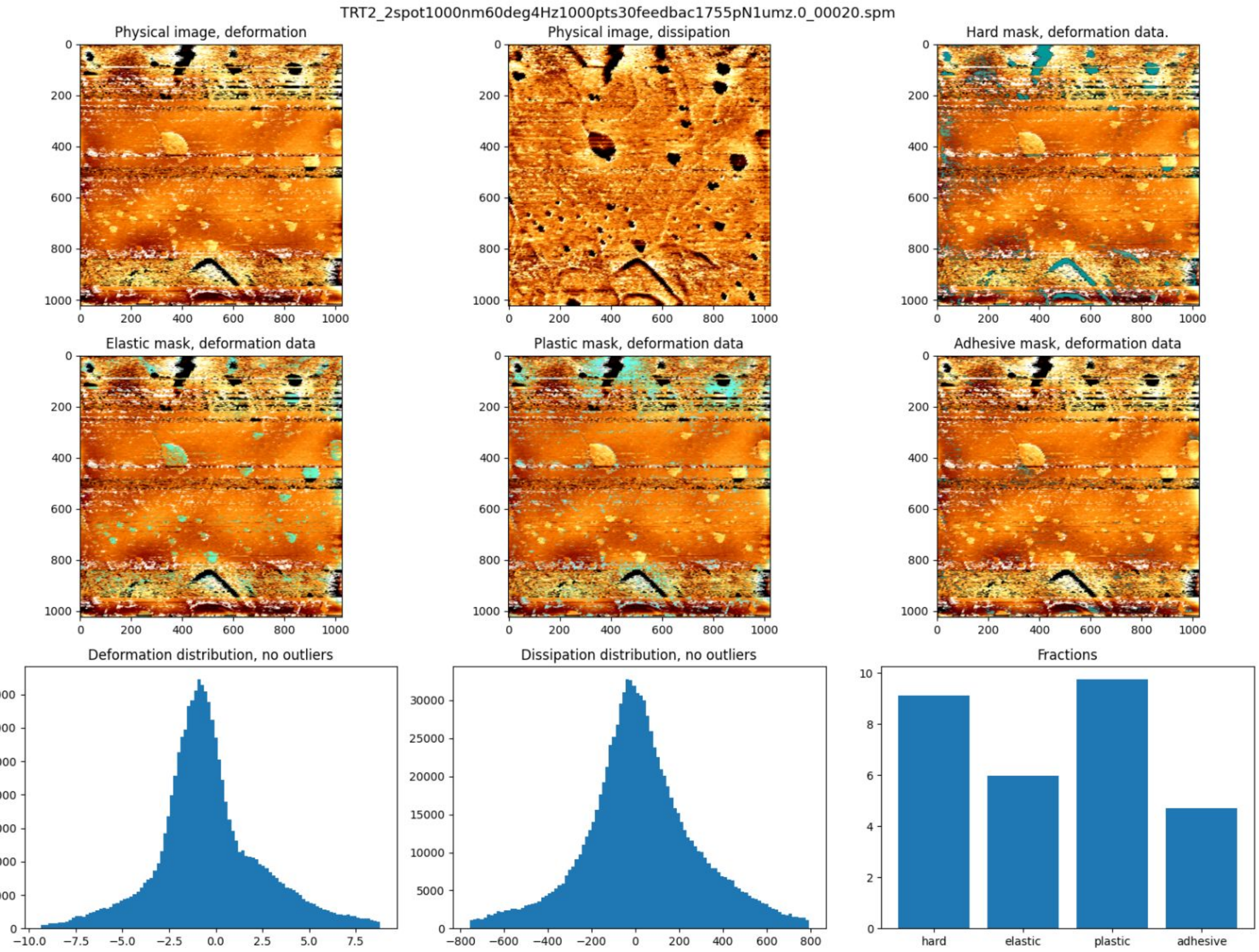
TRT2_2spot1000nm60deg4Hz1000pts30feedbac1755pN1umz.0_00020.spm
Physical image, deformation
Physical image, dissipation
Hard mask, deformation data.
Elastic mask, deformation data
Plastic mask, deformation data
Adhesive mask, deformation data
Deformation distribution, no outliers
Dissipation distribution, no outliers
Fractions
hard
elastic
plastic
adhesive

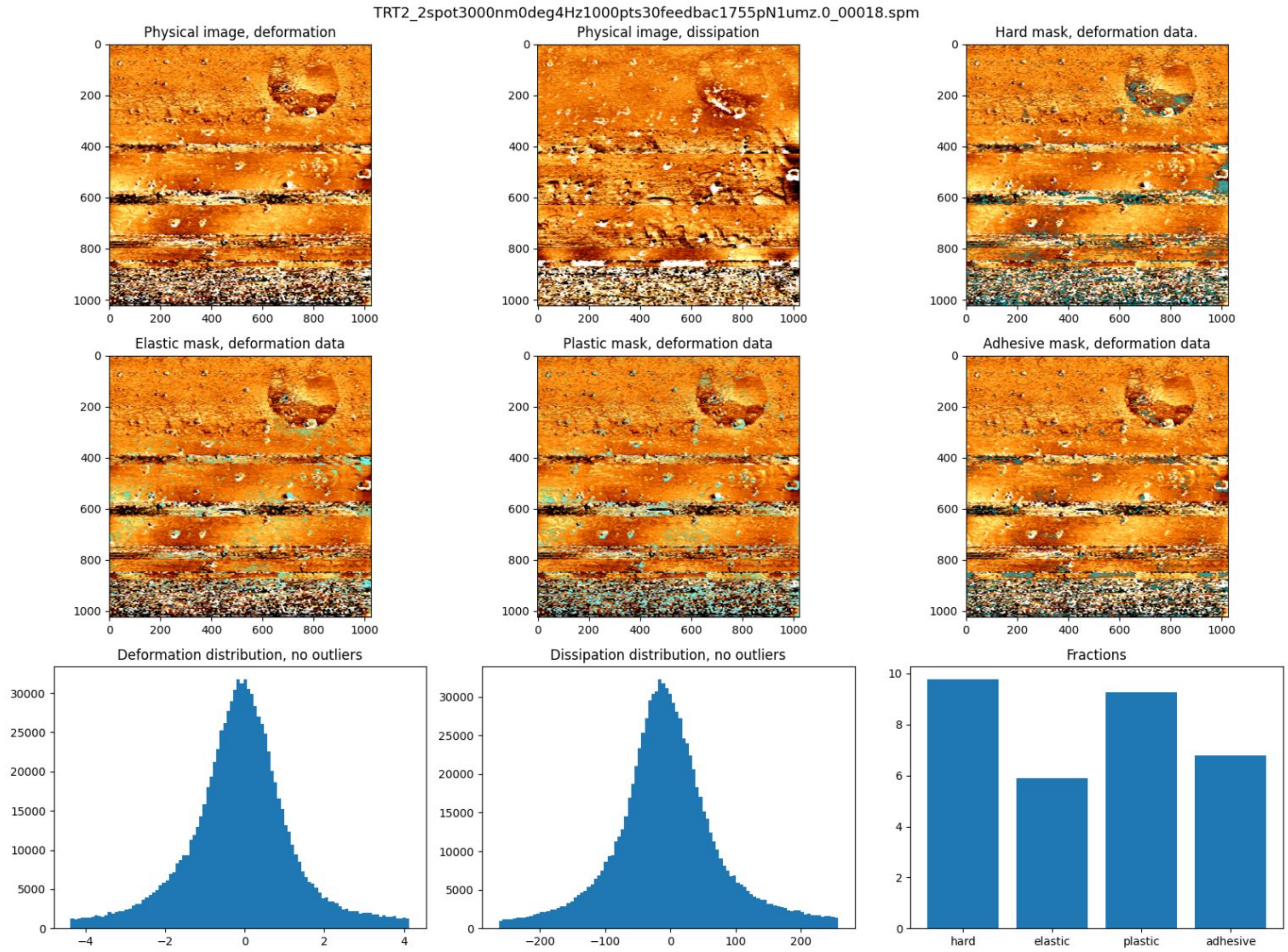
TRT2_2spot3000nm0deg4Hz1000pts30feedbac1755pN1umz.0_00018.spm
Physical image, deformation
Physical image, dissipation
Hard mask, deformation data.
Elastic mask, deformation data
Plastic mask, deformation data
Adhesive mask, deformation data
Deformation distribution, no outliers
Dissipation distribution, no outliers
Fractions
hard
elastic
plastic
adhesive

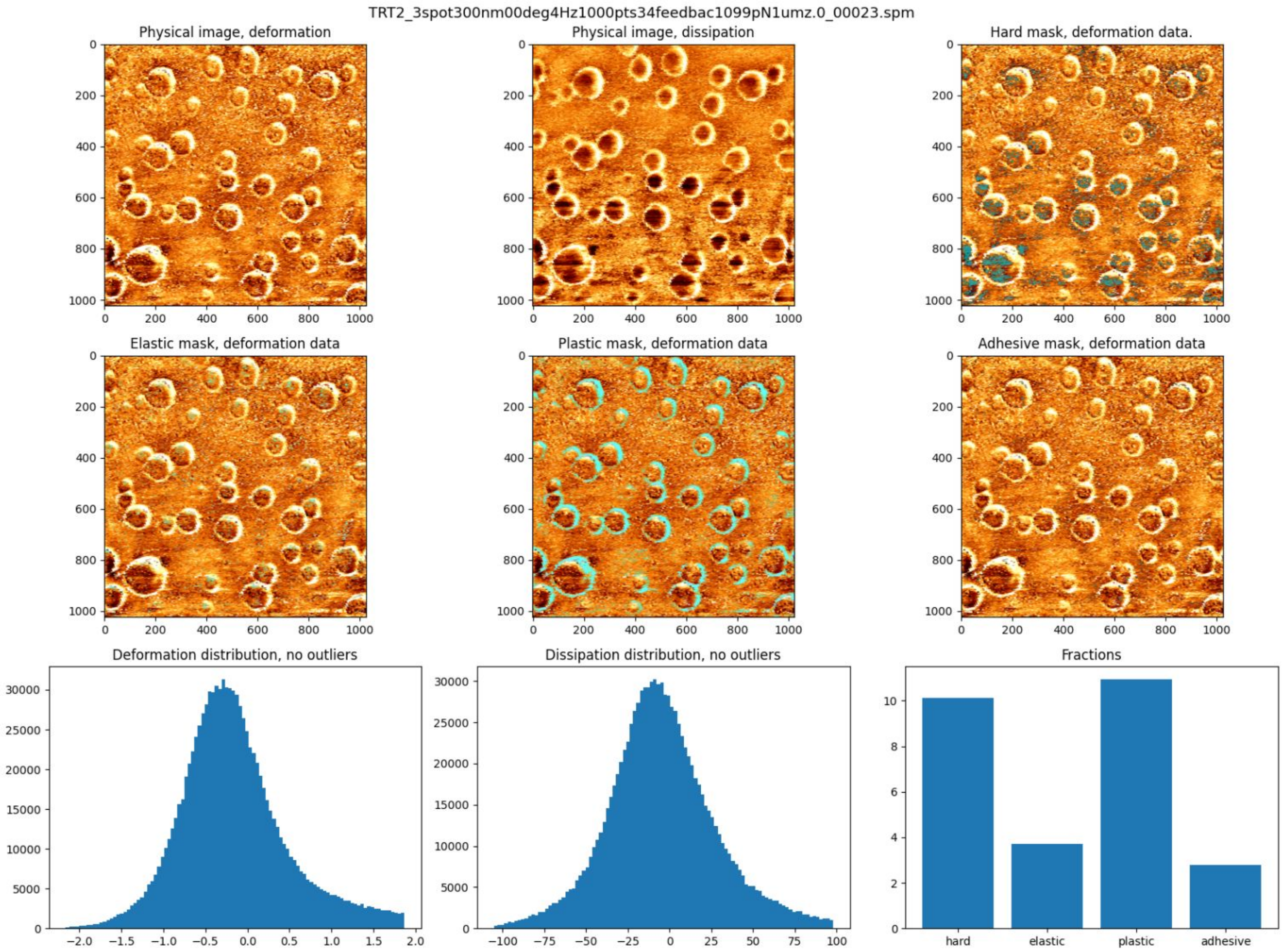
TRT2_3spot300nm00deg4Hz1000pts34feedbac1099pN1umz.0_00023.spm
Physical image, deformation
Physical image, dissipation
Hard mask, deformation data.
Elastic mask, deformation data
Plastic mask, deformation data
Adhesive mask, deformation data
Deformation distribution, no outliers
Dissipation distribution, no outliers
Fractions
hard
elastic
plastic
adhesive

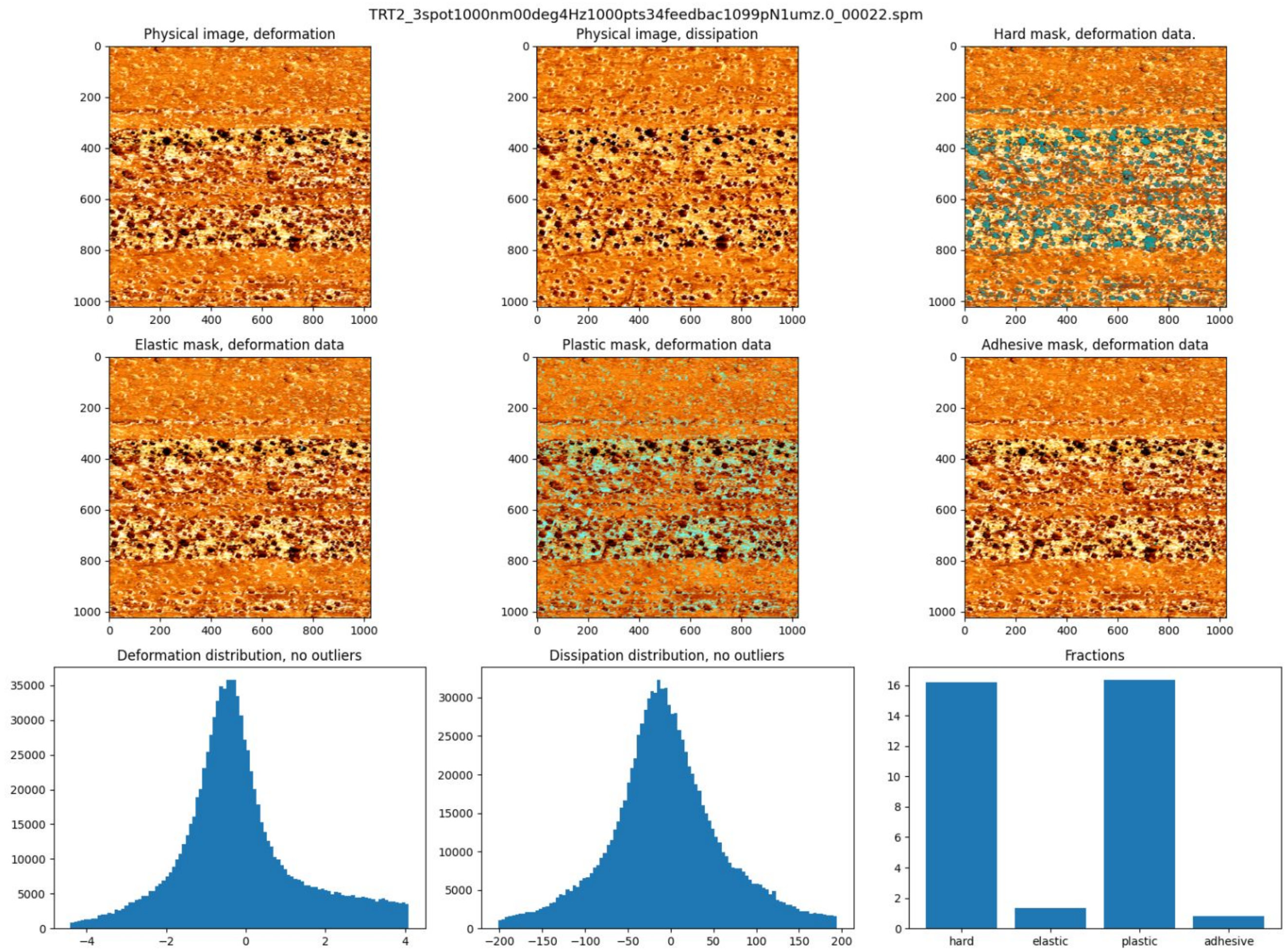
TRT2_3spot1000nm00deg4Hz1000pts34feedbac1099pN1umz.0_00022.spm
Physical image, deformation
Physical image, dissipation
Hard mask, deformation data.
Elastic mask, deformation data
Plastic mask, deformation data
Adhesive mask, deformation data
Deformation distribution, no outliers
Dissipation distribution, no outliers
Fractions
hard
elastic
plastic
adhesive

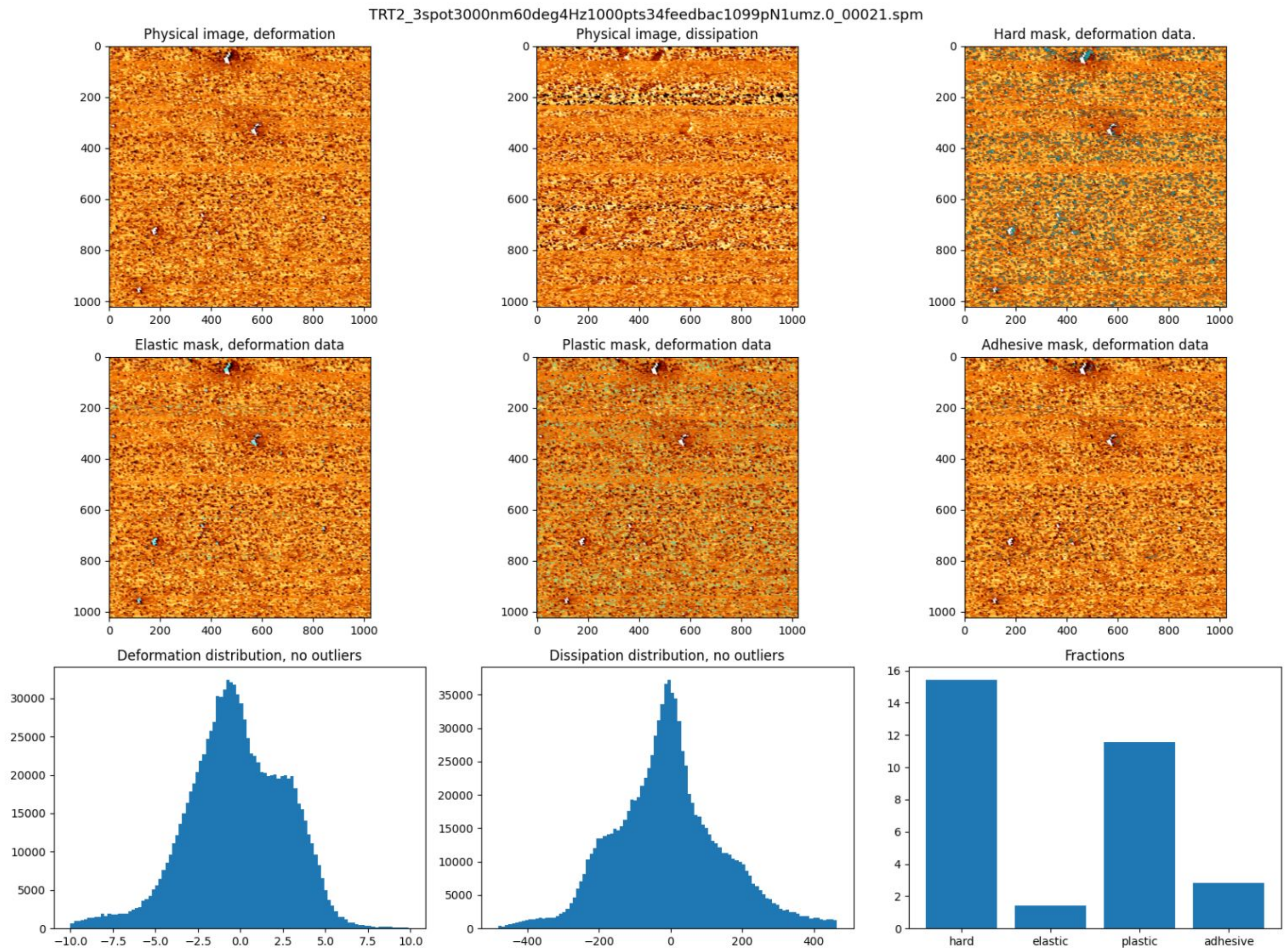
TRT2_3spot3000nm60deg4Hz1000pts34feedbac1099pN1umz.0_00021.spm
Physical image, deformation
Physical image, dissipation
Hard mask, deformation data.
Elastic mask, deformation data
Plastic mask, deformation data
Adhesive mask, deformation data
Deformation distribution, no outliers
Dissipation distribution, no outliers
Fractions
hard
elastic
plastic
adhesive

TRT- R Recipe 3, type 1

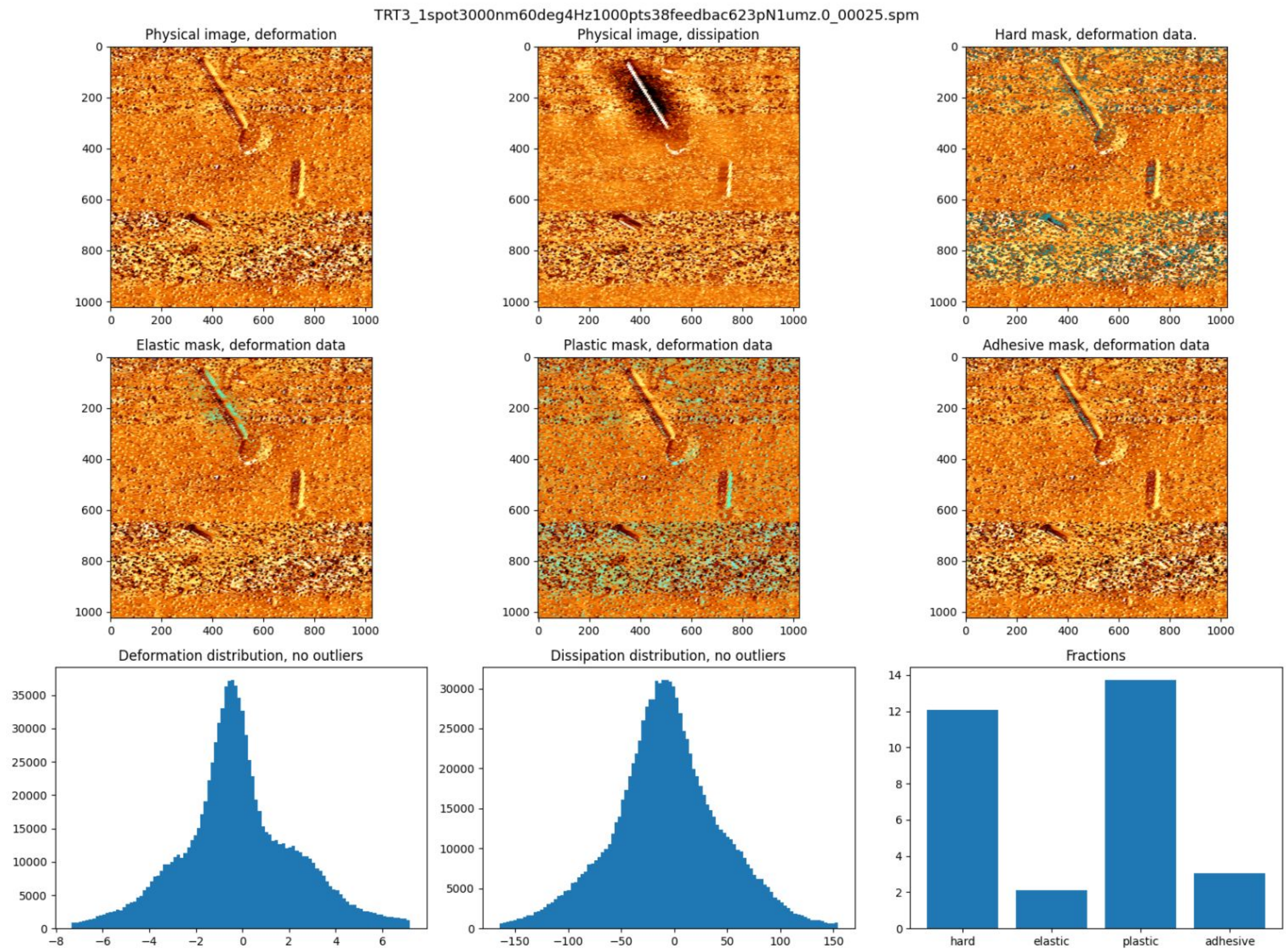

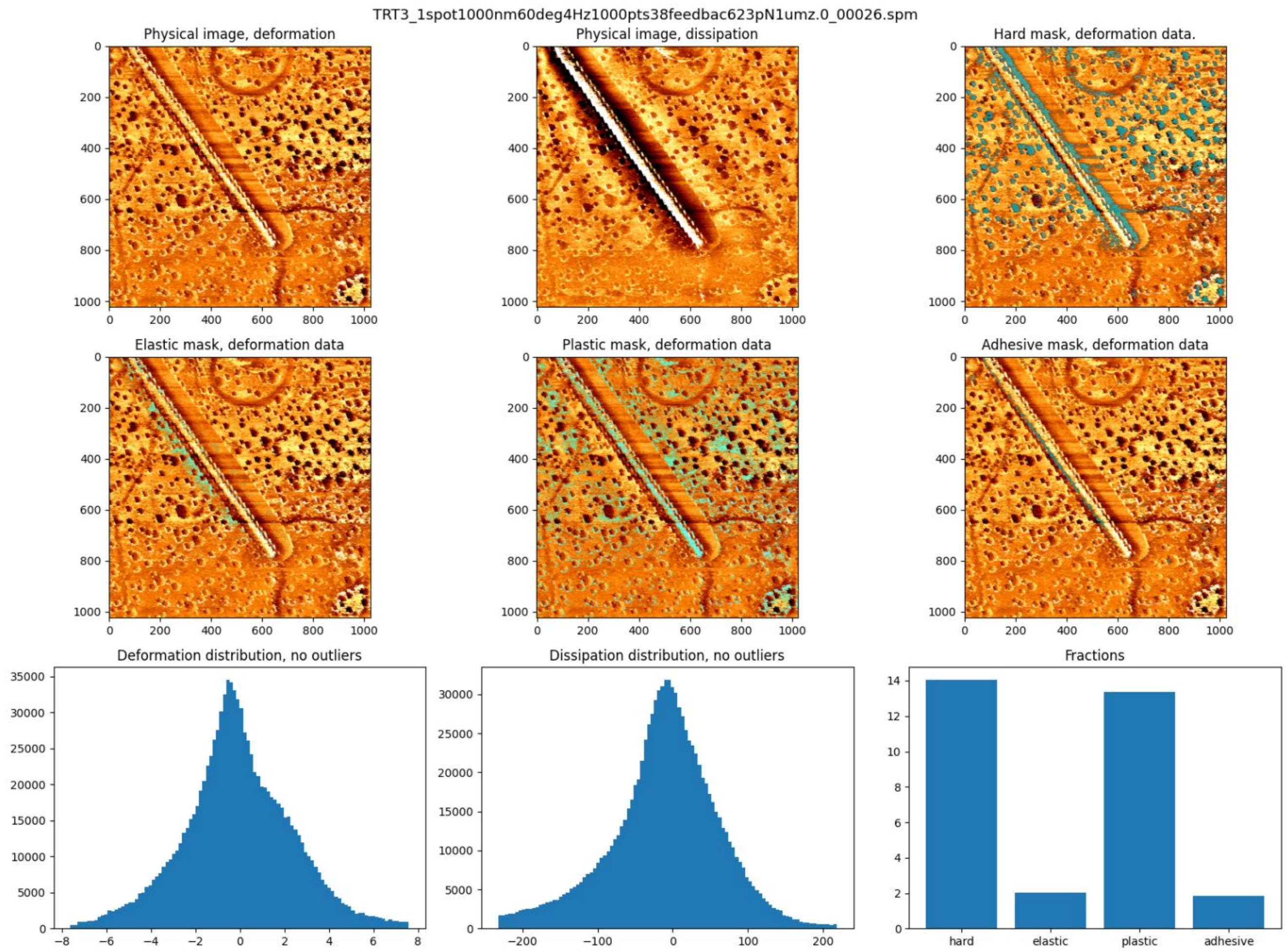
TRT3_1spot1000nm60deg4Hz1000pts38feedbac623pN1umz.0_00026.spm
Physical image, deformation
Physical image, dissipation
Hard mask, deformation data.
Elastic mask, deformation data
Plastic mask, deformation data
Adhesive mask, deformation data
Deformation distribution, no outliers
Dissipation distribution, no outliers
Fractions
hard
elastic
plastic
adhesive

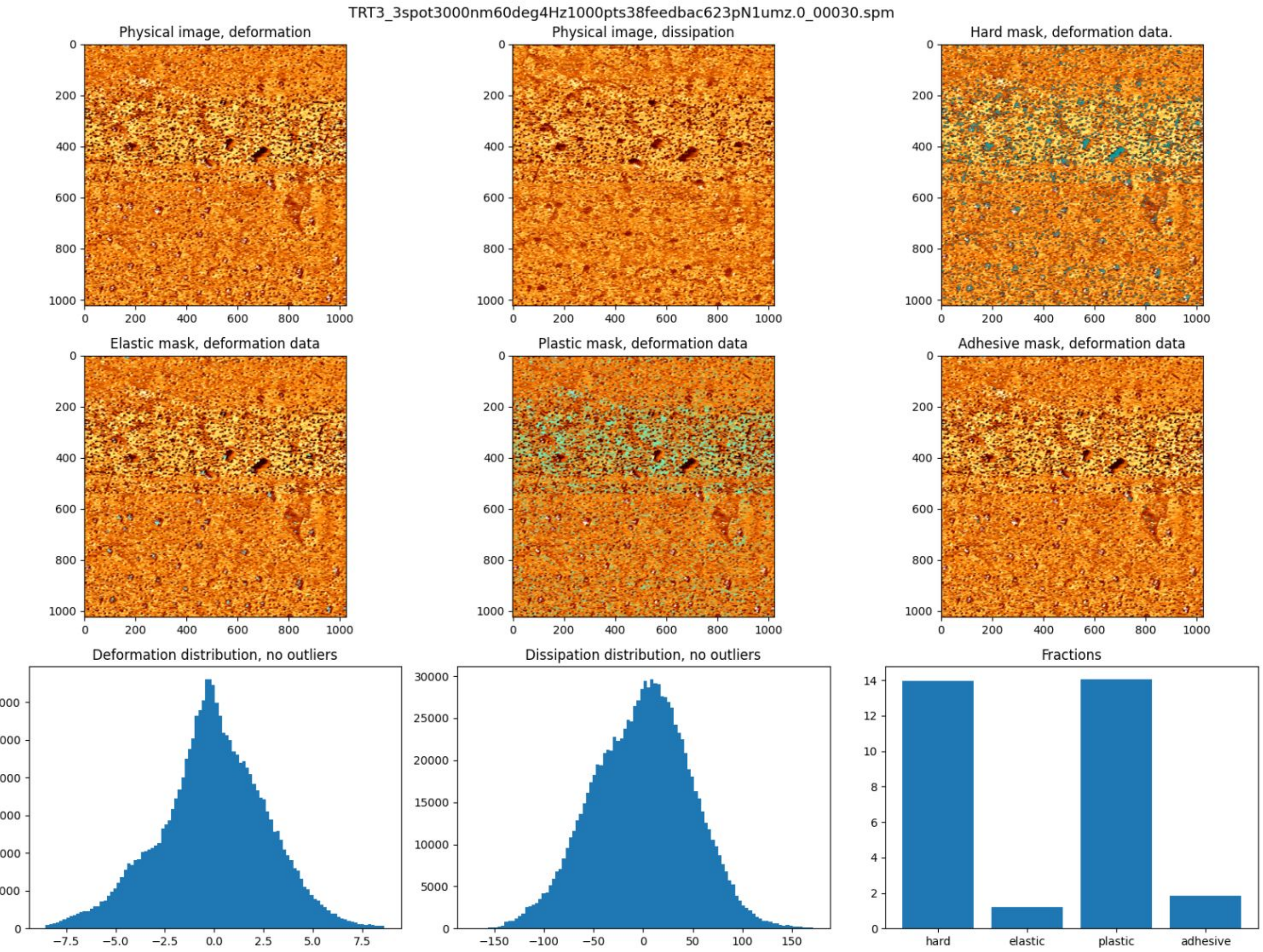
TRT3_3spot3000nm60deg4Hz1000pts38feedbac623pN1umz.0_00030.spm
Physical image, deformation
Physical image, dissipation
Hard mask, deformation data.
Elastic mask, deformation data
Plastic mask, deformation data
Adhesive mask, deformation data
Deformation distribution, no outliers
Dissipation distribution, no outliers
Fractions
hard
elastic
plastic
adhesive

TRT- R Recipe 3, type 2

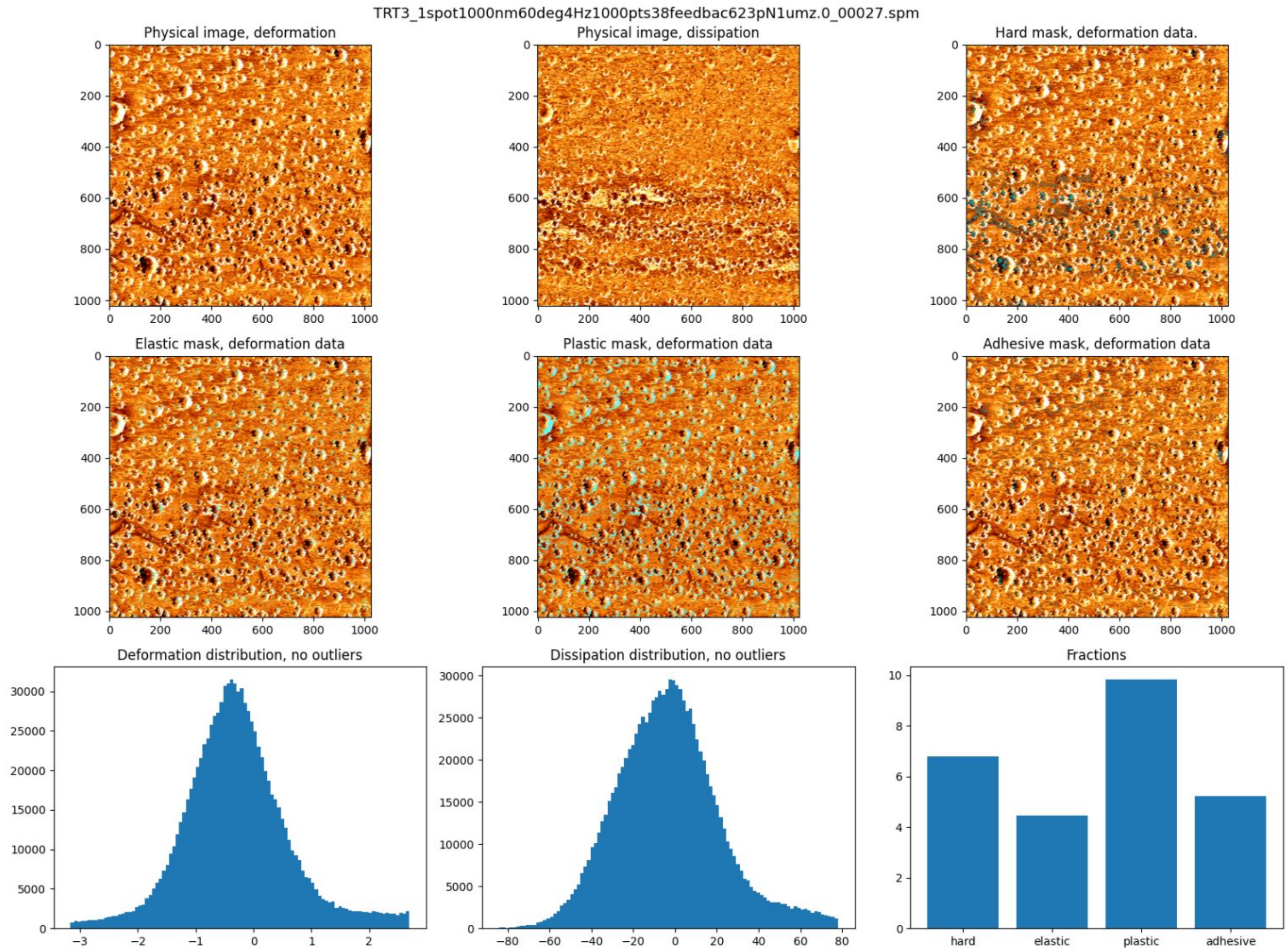

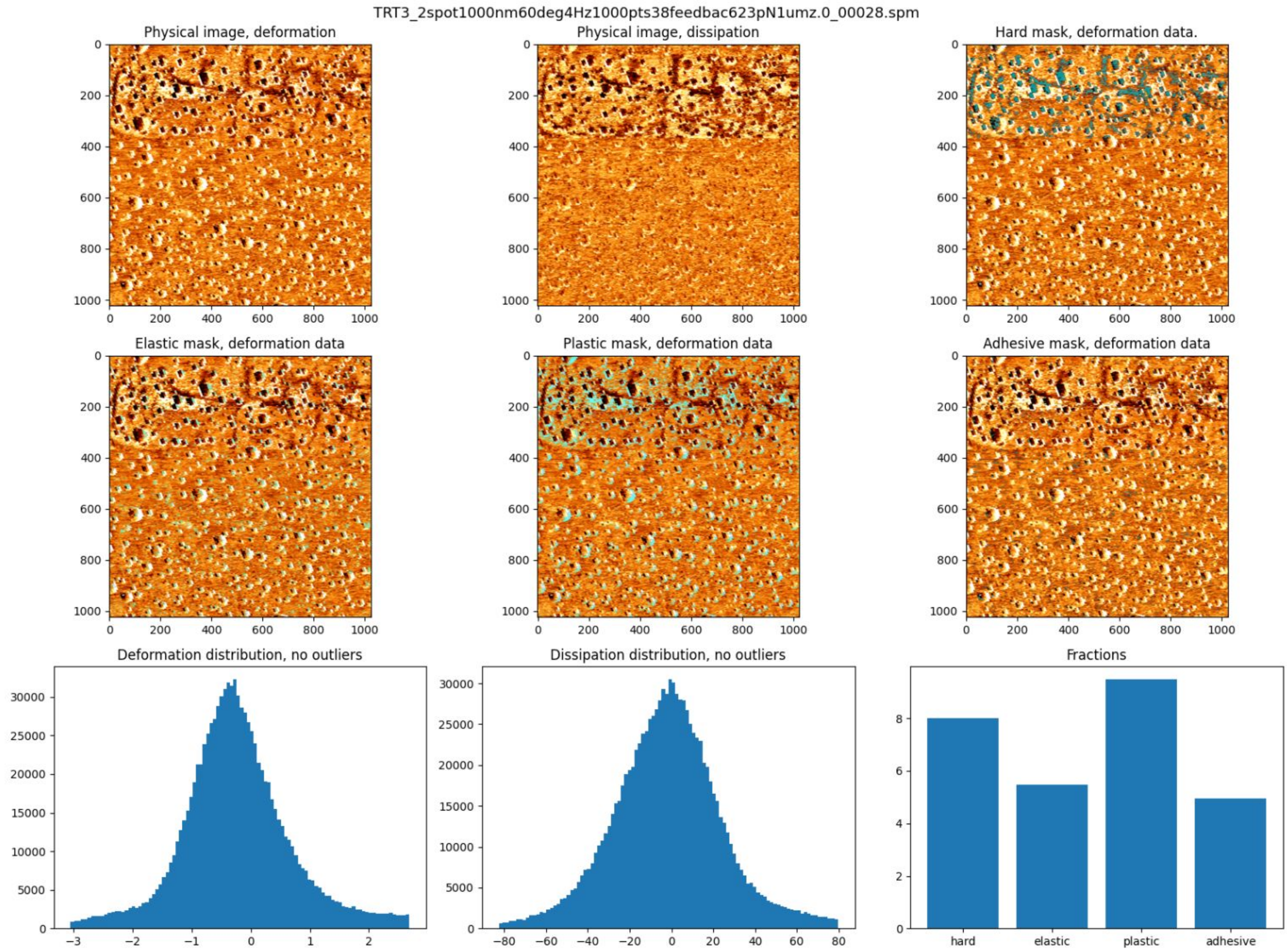
TRT3_2spot1000nm60deg4Hz1000pts38feedbac623pN1umz.0_00028.spm
Physical image, deformation
Physical image, dissipation
Hard mask, deformation data.
Elastic mask, deformation data
Plastic mask, deformation data
Adhesive mask, deformation data
Deformation distribution, no outliers
Dissipation distribution, no outliers
Fractions
hard
elastic
plastic
adhesive

TRT- R Recipe 3, type 3

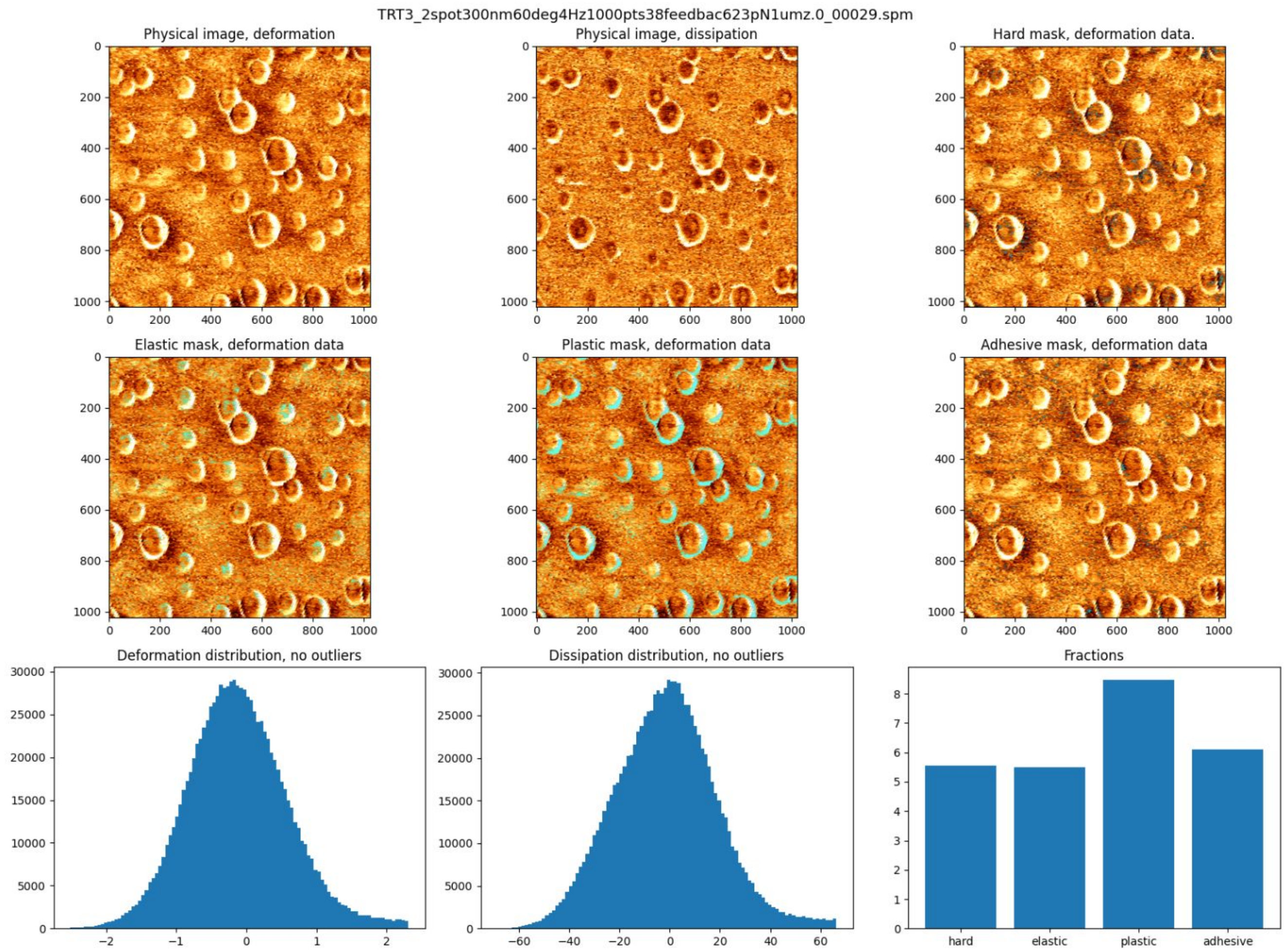

TRT- R Recipe 4, type 1

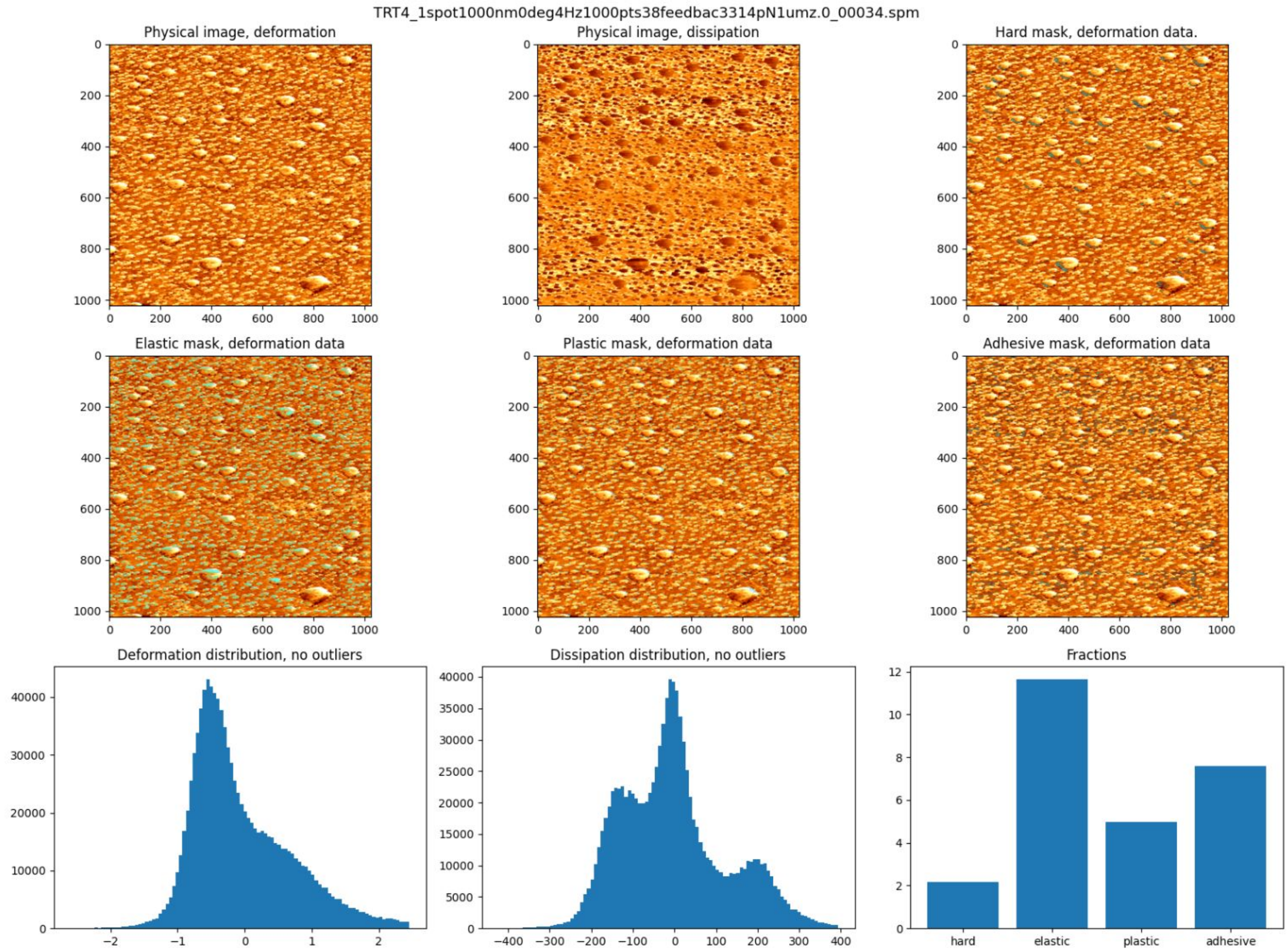

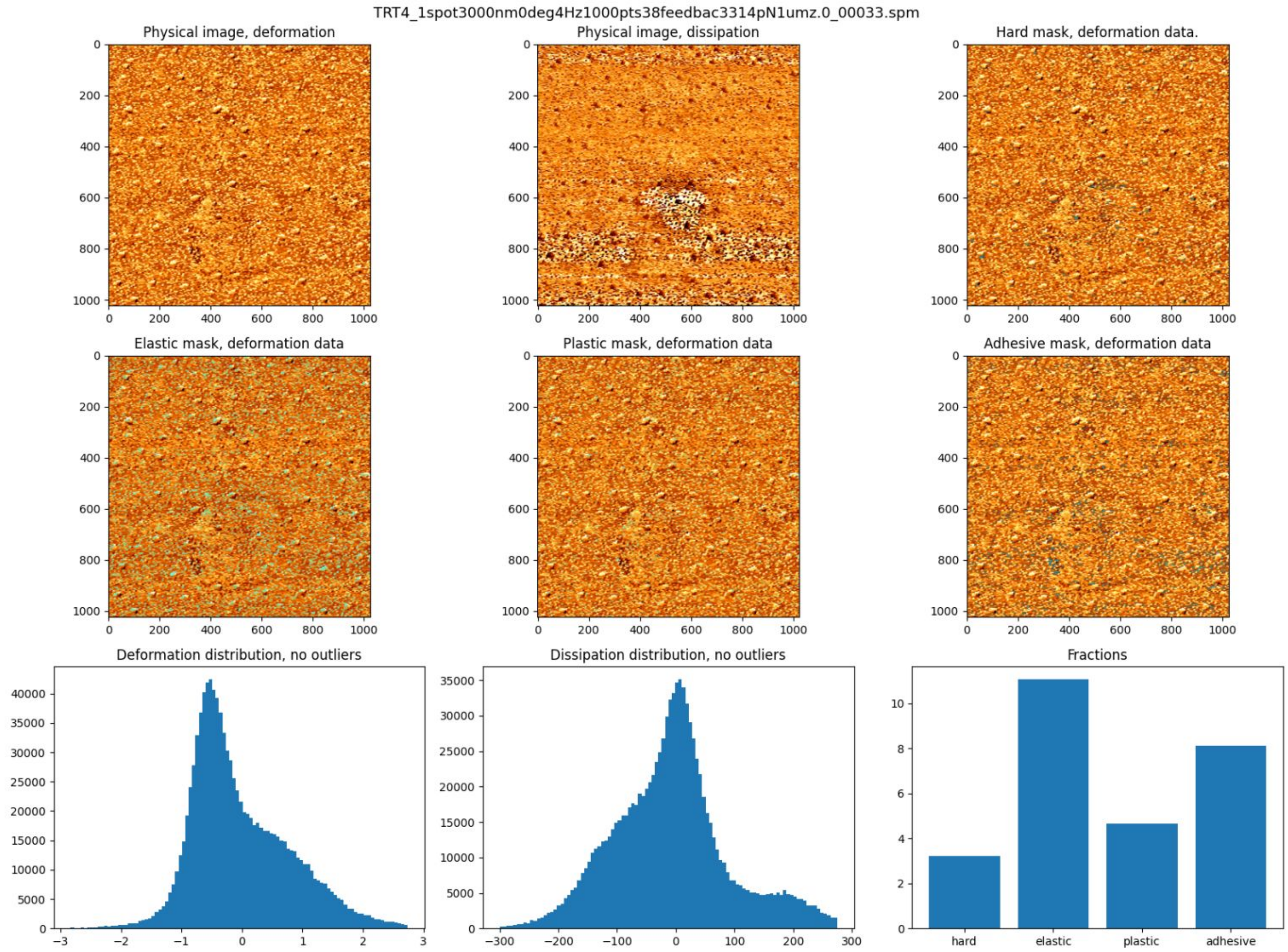
TRT4_1spot3000nm0deg4Hz1000pts38feedbac3314pN1umz.0_00033.spm
Physical image, deformation
Physical image, dissipation
Hard mask, deformation data.
Elastic mask, deformation data
Plastic mask, deformation data
Adhesive mask, deformation data
Deformation distribution, no outliers
Dissipation distribution, no outliers
Fractions
hard
elastic
plastic
adhesive

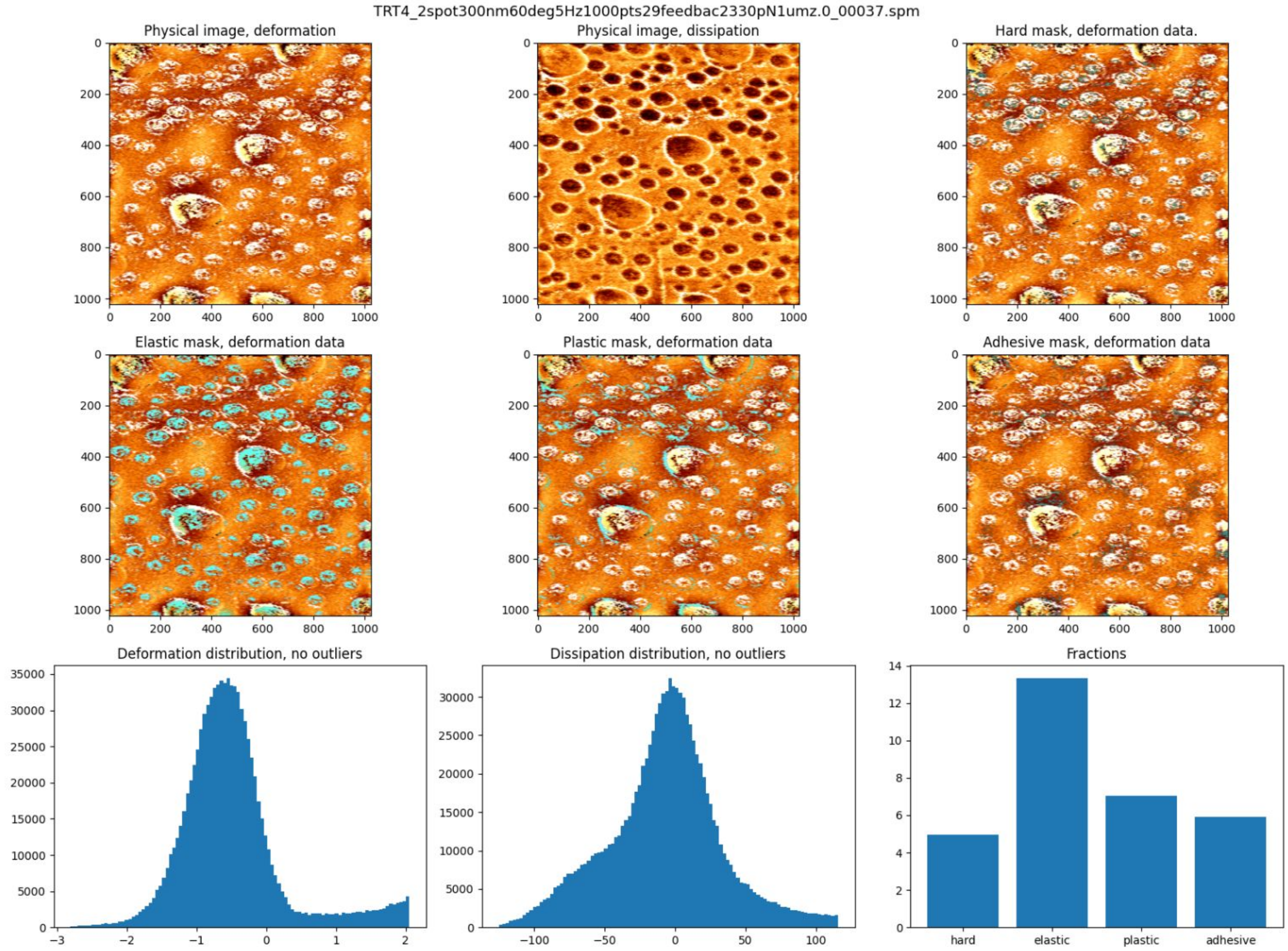
TRT4_2spot300nm60deg5Hz1000pts29feedbac2330pN1umz.0_00037.spm
Physical image, deformation
Physical image, dissipation
Hard mask, deformation data.
Elastic mask, deformation data
Plastic mask, deformation data
Adhesive mask, deformation data
Deformation distribution, no outliers
Dissipation distribution, no outliers
Fractions
hard
elastic
plastic
adhesive

TRT- R Recipe 4, type 2

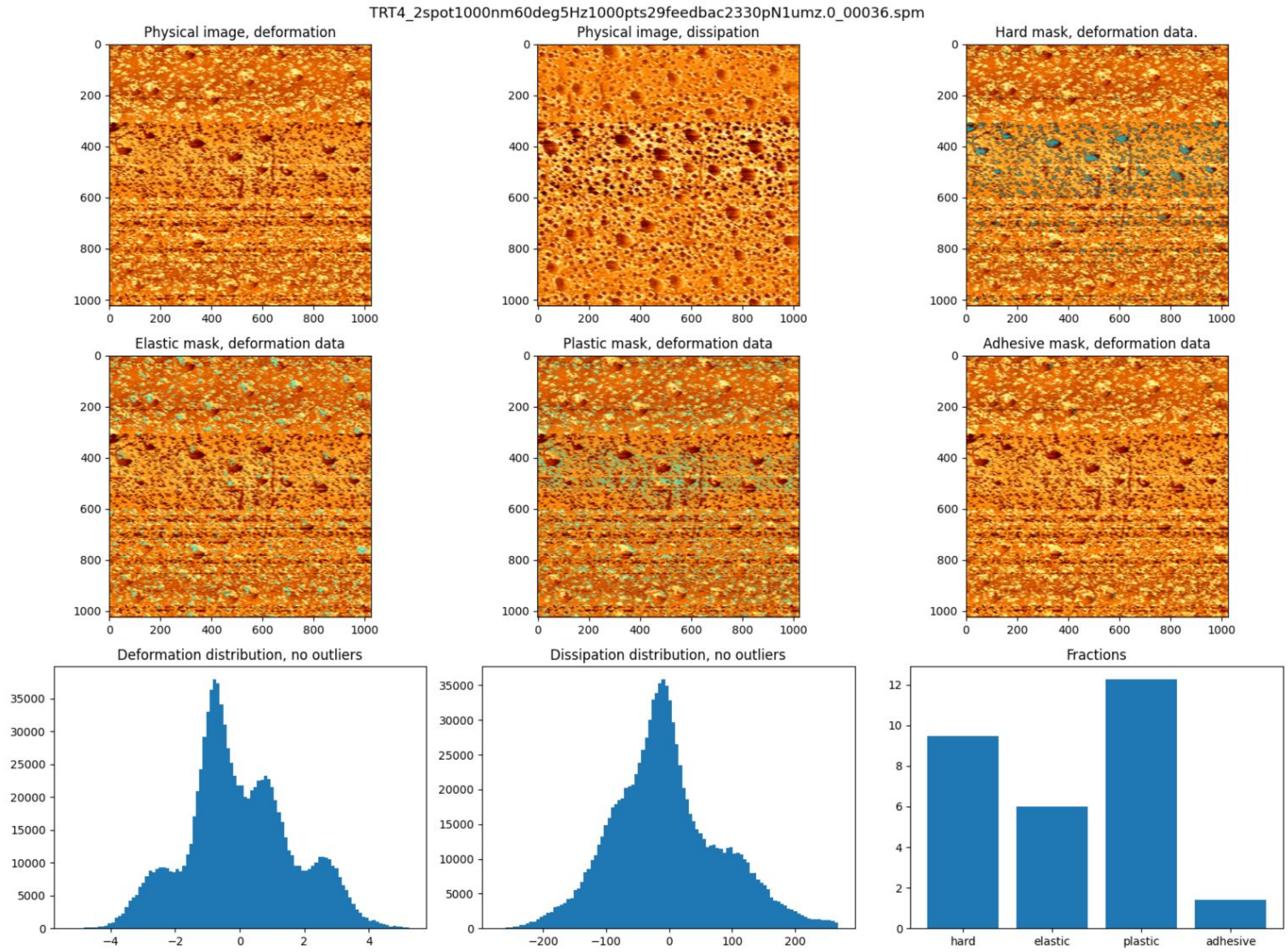

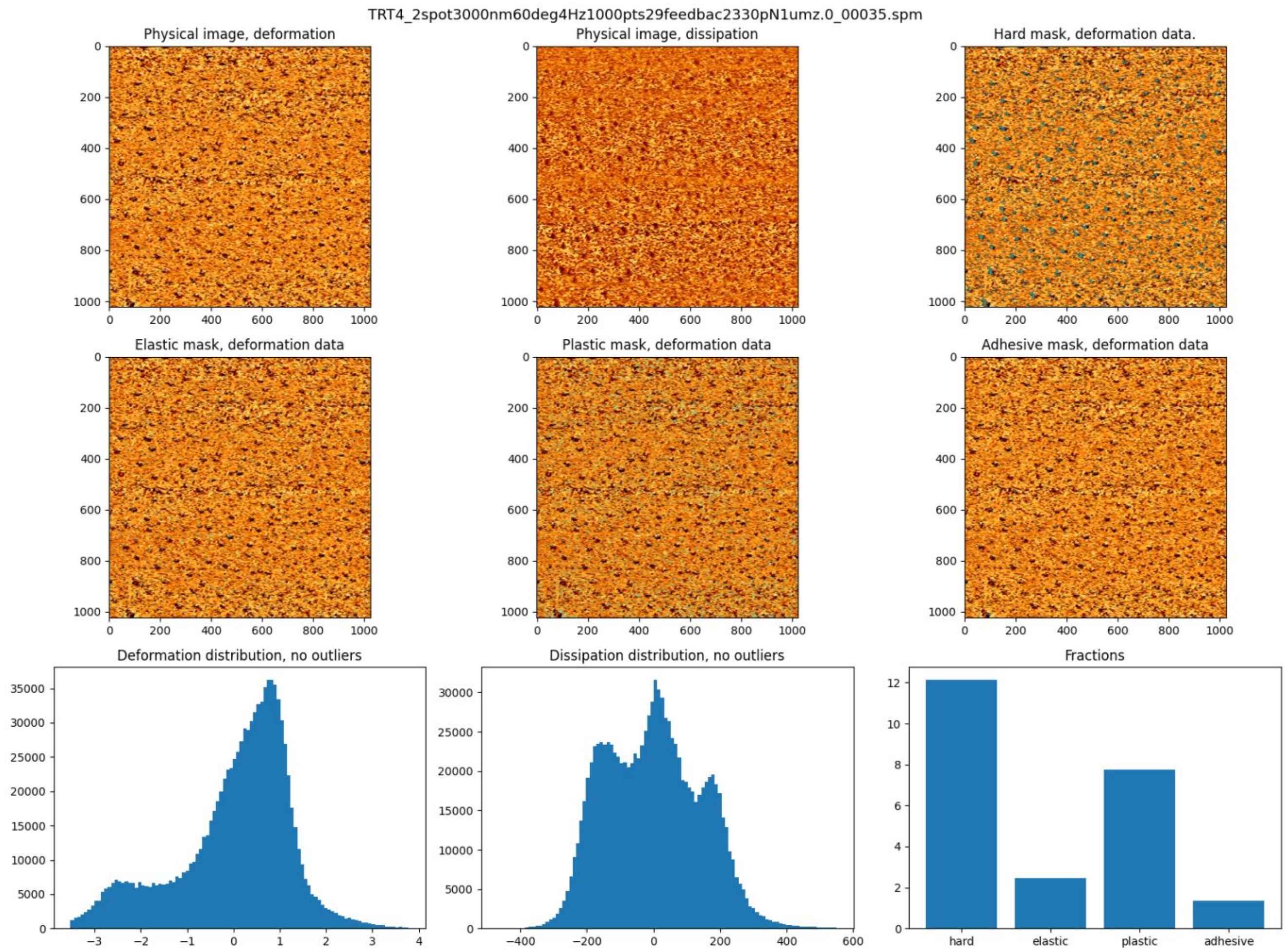
TRT4_2spot3000nm60deg4Hz1000pts29feedbac2330pN1umz.0_00035.spm
Physical image, deformation
Physical image, dissipation
Hard mask, deformation data.
Elastic mask, deformation data
Plastic mask, deformation data
Adhesive mask, deformation data
Deformation distribution, no outliers
Dissipation distribution, no outliers
Fractions
hard
elastic
plastic
adhesive

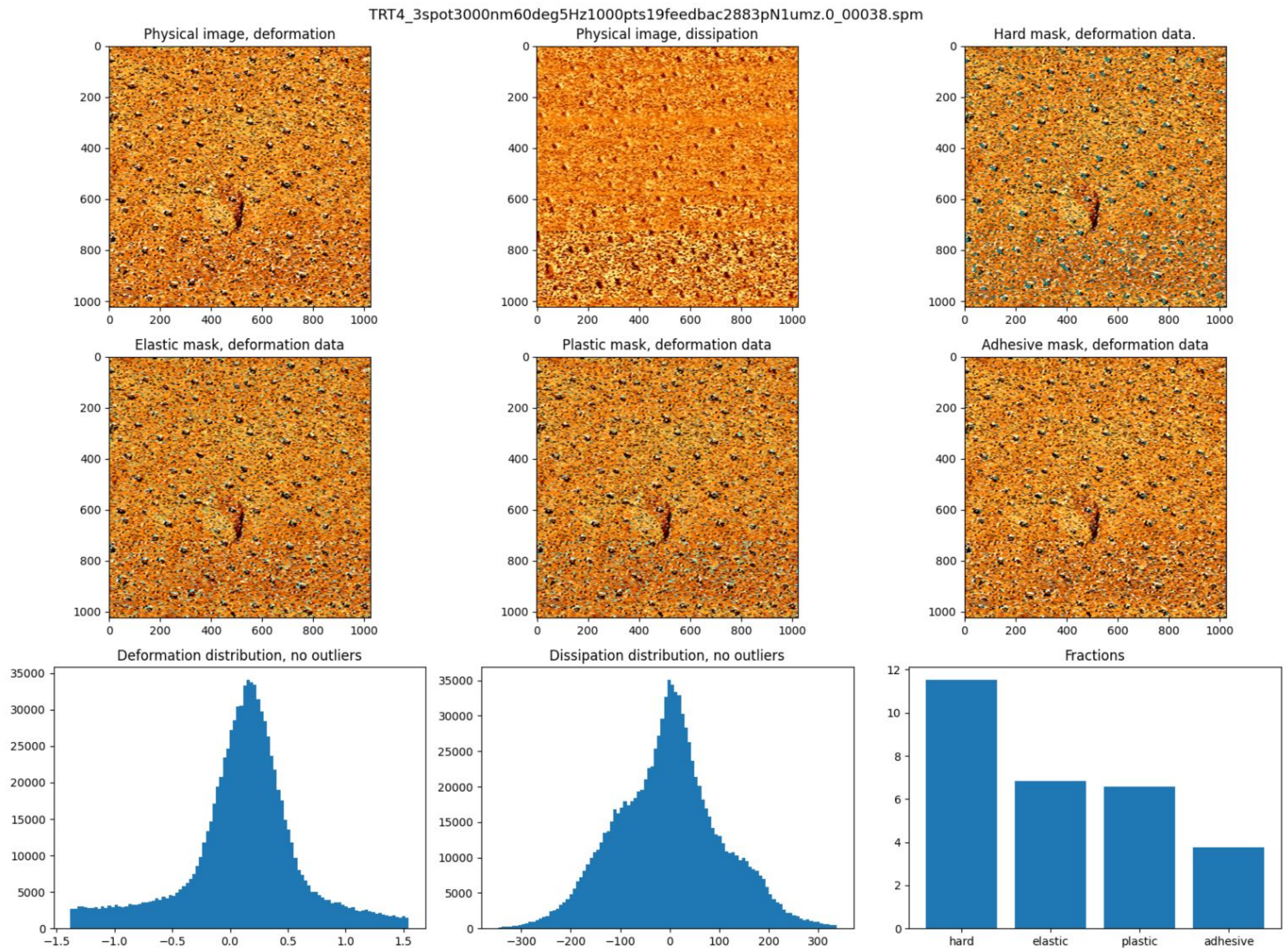

TRT4_3spot3000nm60deg5Hz1000pts19feedbac2883pN1umz.0_00038.spm
Physical image, deformation
Physical image, dissipation
Hard mask, deformation data.
Elastic mask, deformation data
Plastic mask, deformation data
Adhesive mask, deformation data
Deformation distribution, no outliers
Dissipation distribution, no outliers
Fractions
hard
elastic
plastic
adhesive

TRT- R Recipe 4, type 3

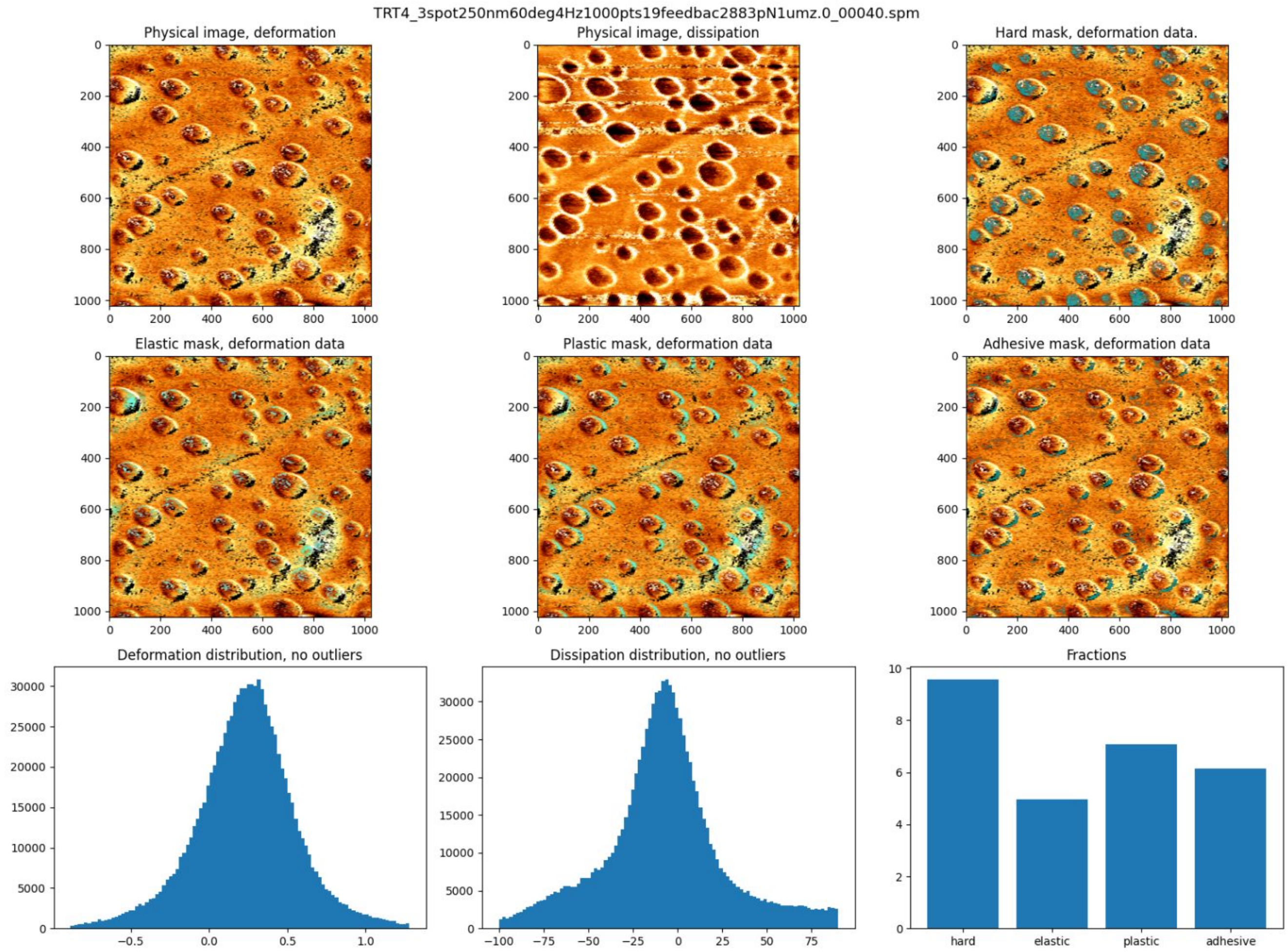

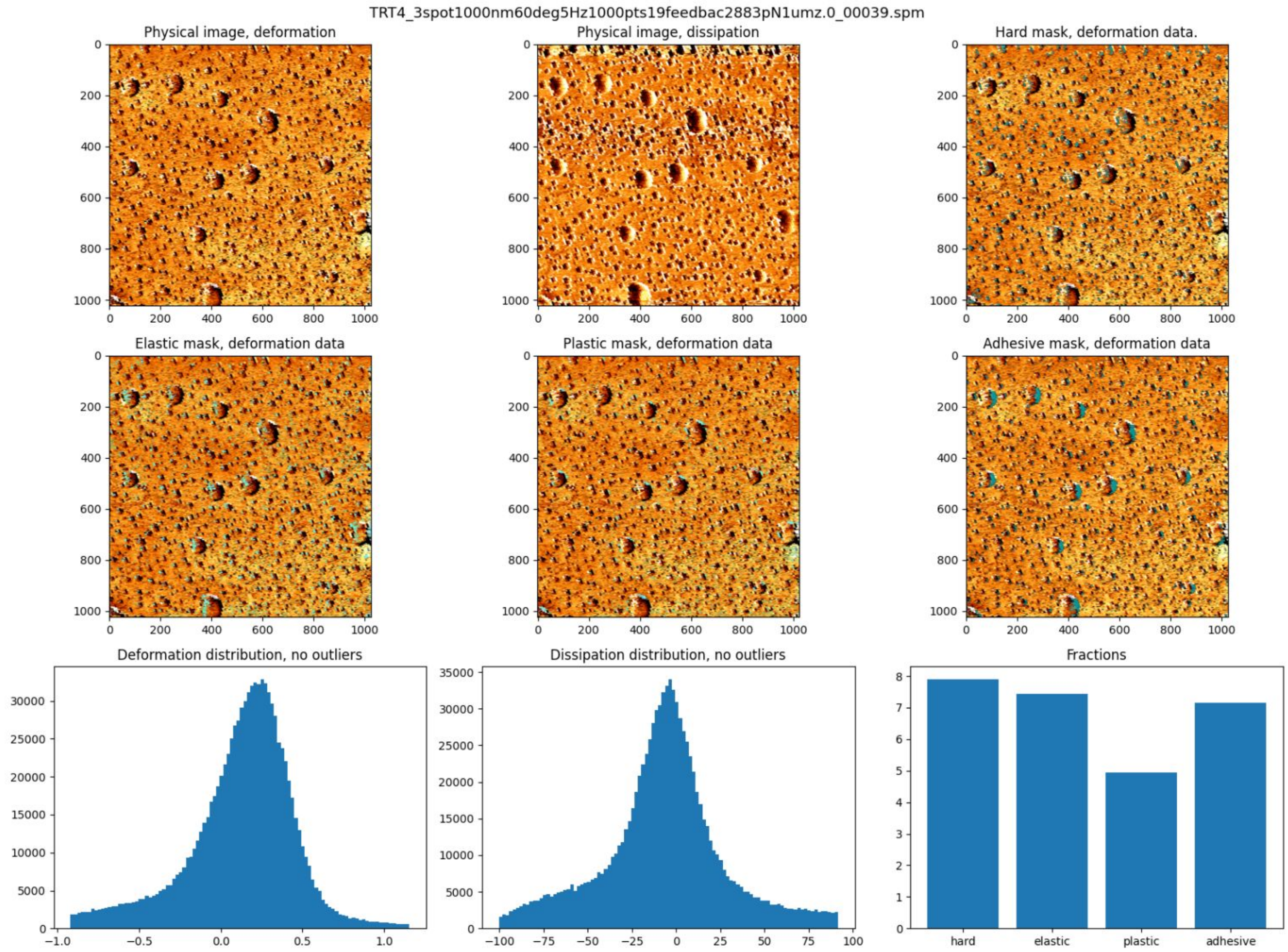

TRT4_3spot1000nm60deg5Hz1000pts19feedbac2883pN1umz.0_00039.spm
Physical image, deformation
Physical image, dissipation
Hard mask, deformation data.
Elastic mask, deformation data
Plastic mask, deformation data
Adhesive mask, deformation data
Deformation distribution, no outliers
Dissipation distribution, no outliers
Fractions
hard
elastic
plastic
adhesive

TRT- R Recipe 5, type 1

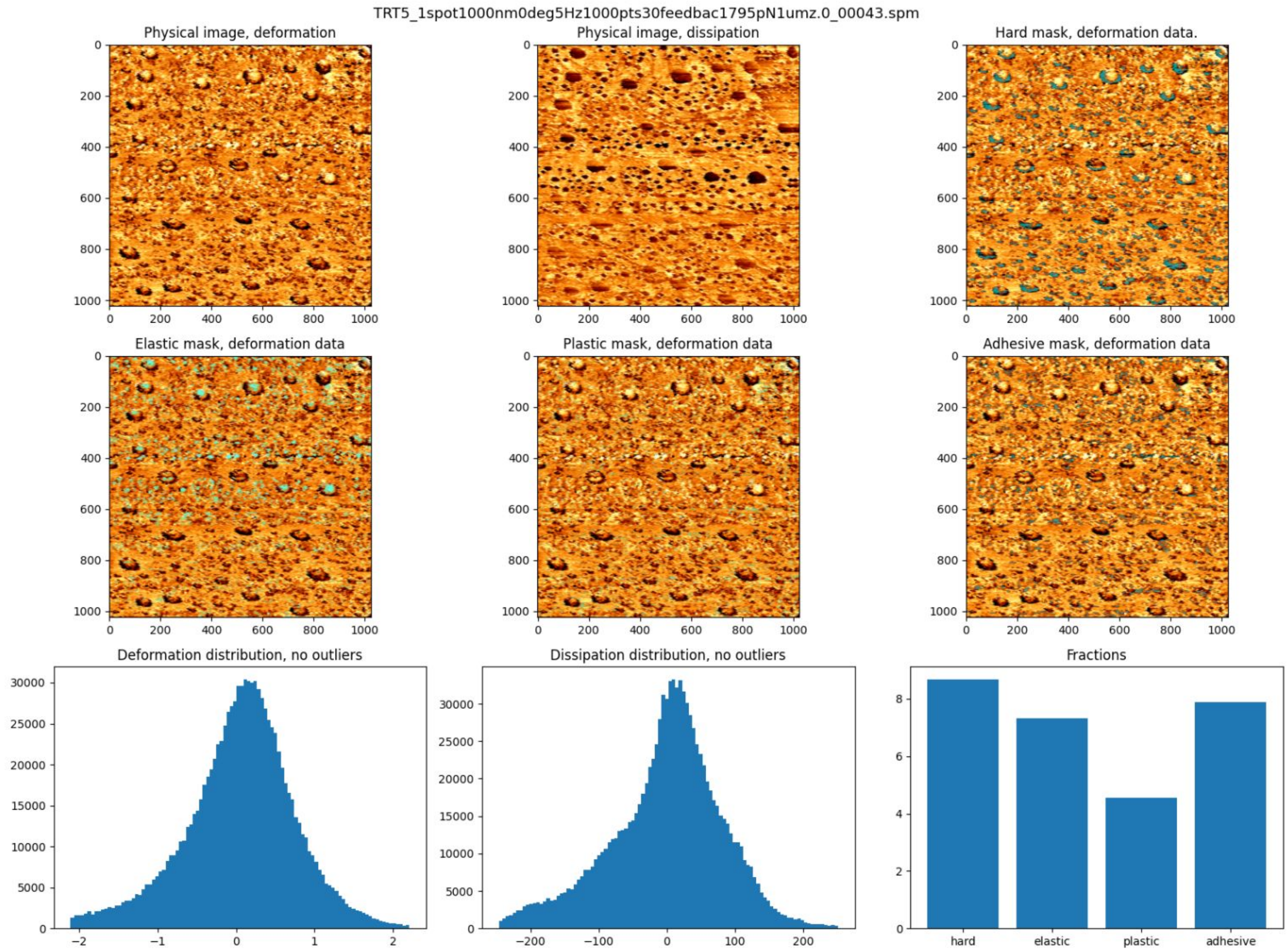

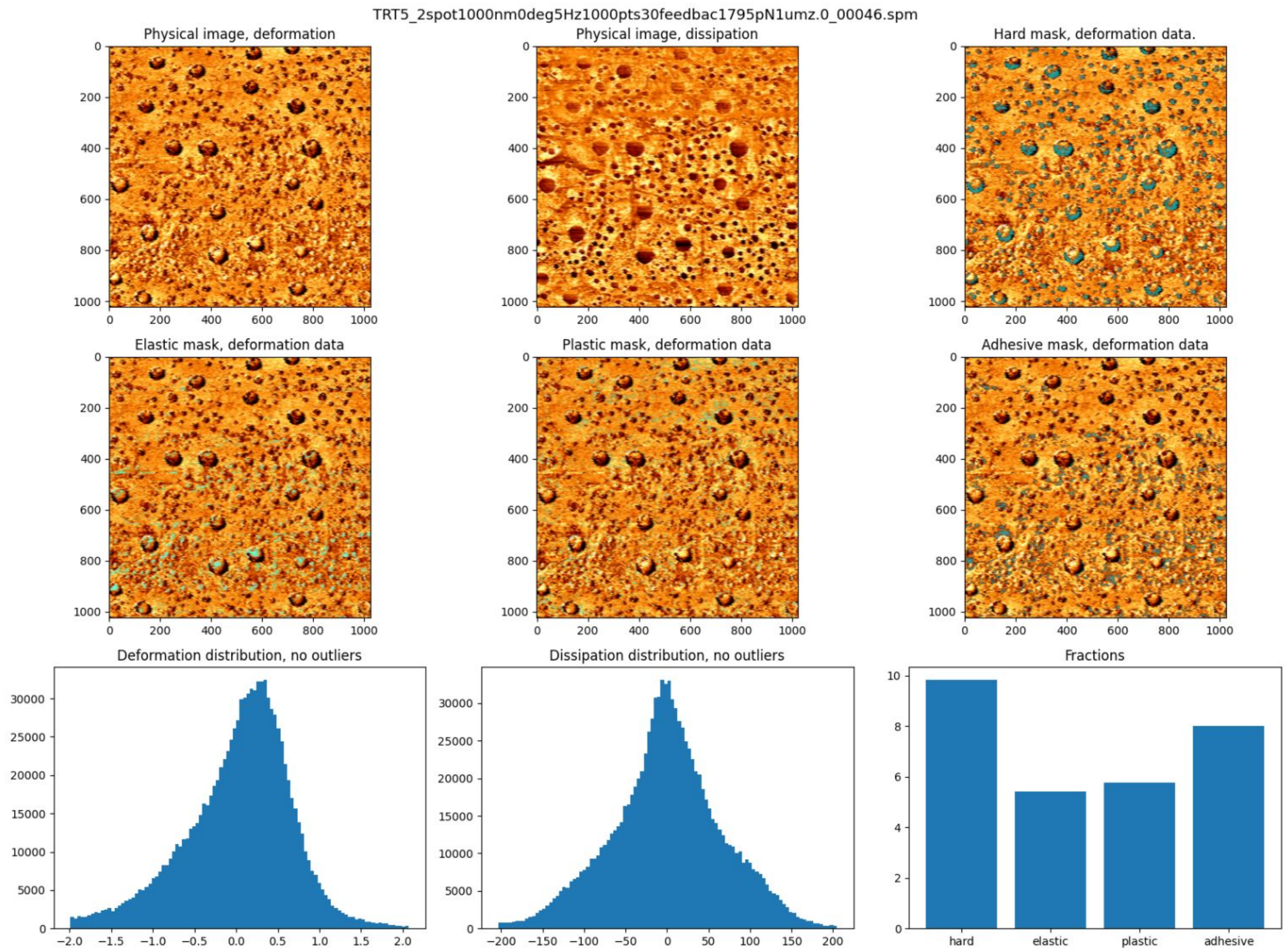
TRT5_2spot1000nm0deg5Hz1000pts30feedbac1795pN1umz.0_00046.spm
Physical image, deformation
Physical image, dissipation
Hard mask, deformation data.
Elastic mask, deformation data
Plastic mask, deformation data
Adhesive mask, deformation data
Deformation distribution, no outliers
Dissipation distribution, no outliers
Fractions
hard
elastic
plastic
adhesive

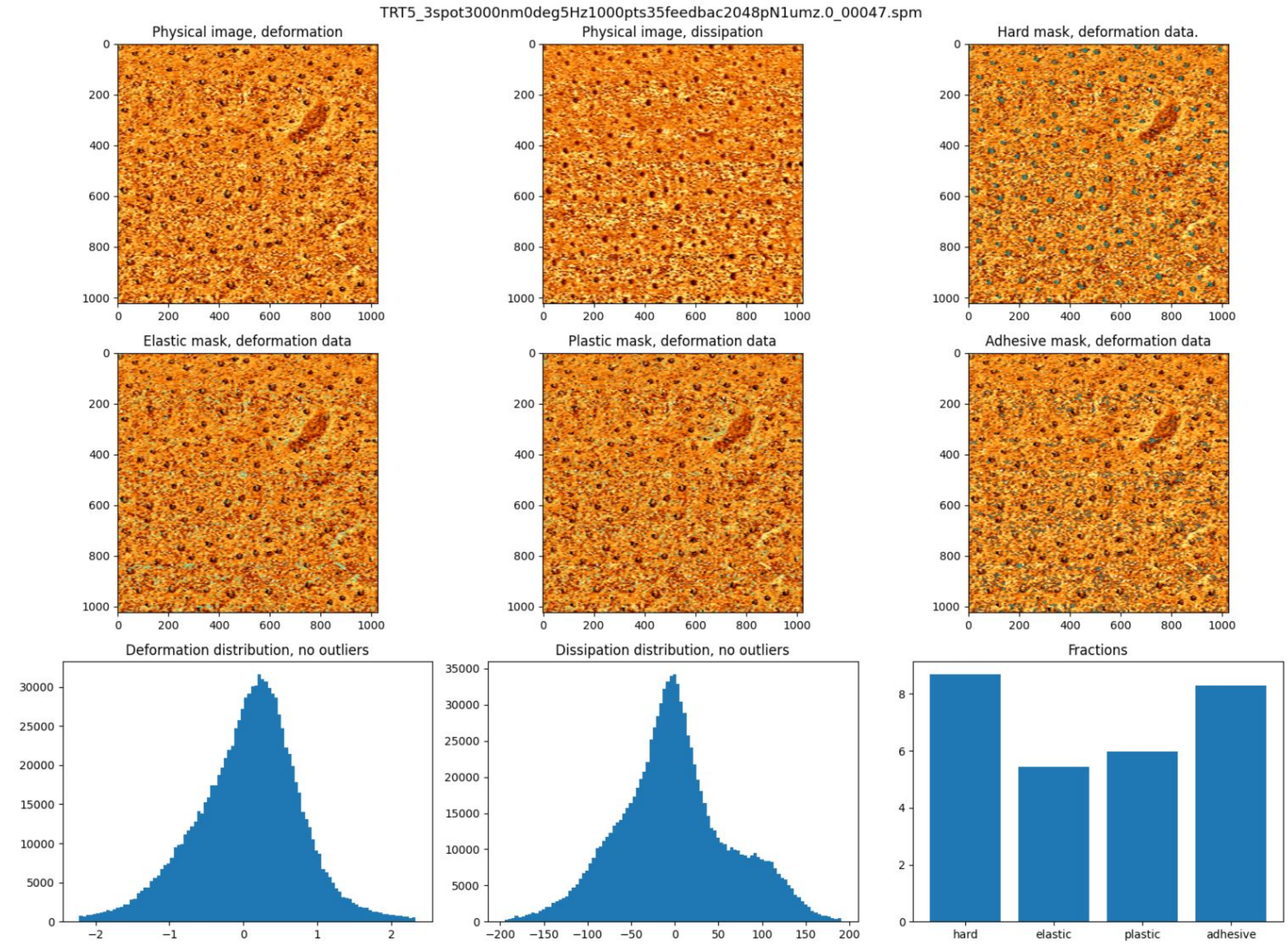
TRT5_3spot3000nm0deg5Hz1000pts35feedbac2048pN1umz.0_00047.spm
Physical image, deformation
Physical image, dissipation
Hard mask, deformation data.
Elastic mask, deformation data
Plastic mask, deformation data
Adhesive mask, deformation data
Deformation distribution, no outliers
Dissipation distribution, no outliers
Fractions
hard
elastic
plastic
adhesive

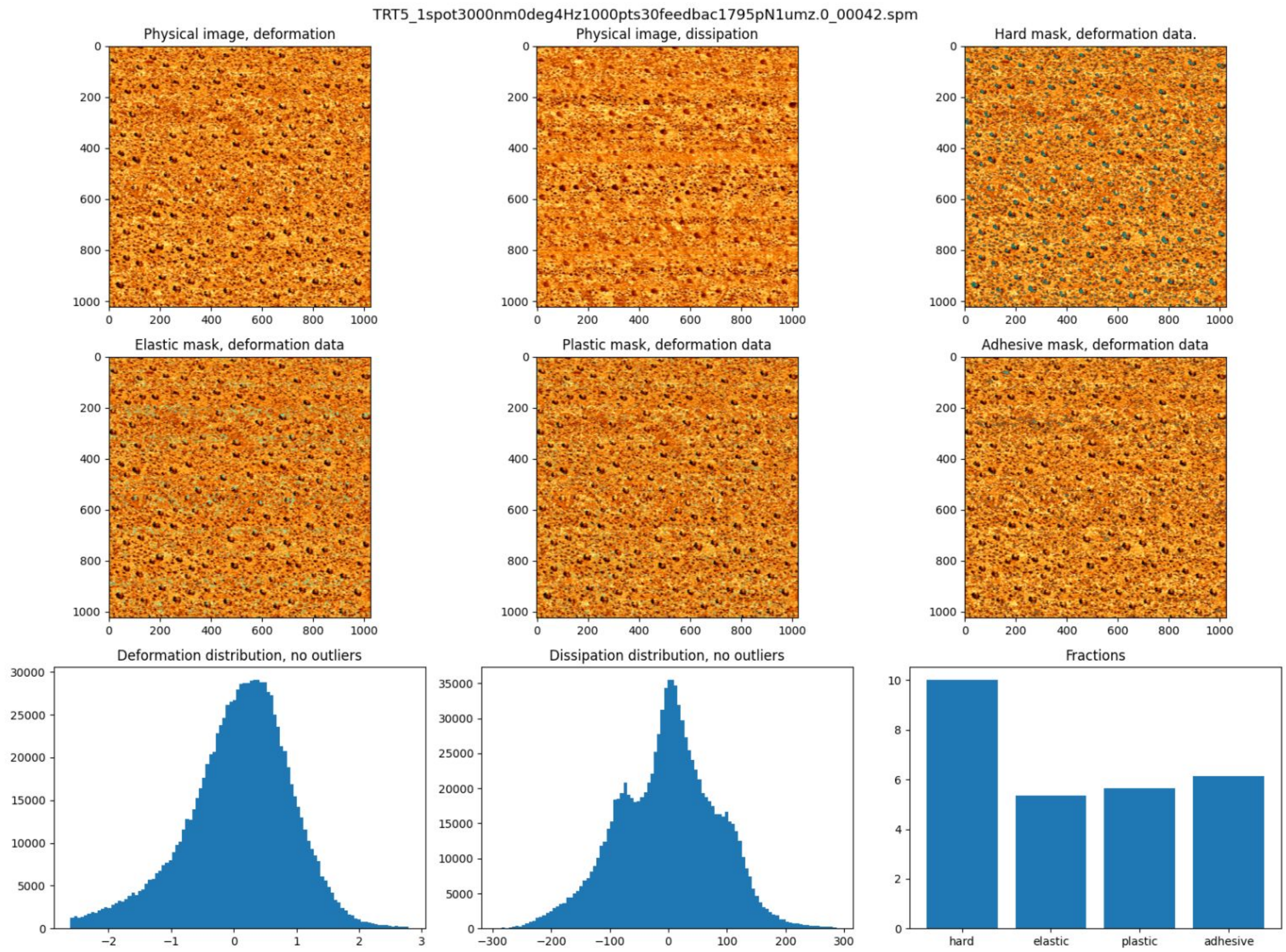
TRT5_1spot3000nm0deg4Hz1000pts30feedbac1795pN1umz.0_00042.spm
Physical image, deformation
Physical image, dissipation
Hard mask, deformation data.
Elastic mask, deformation data
Plastic mask, deformation data
Adhesive mask, deformation data
Deformation distribution, no outliers
Dissipation distribution, no outliers
Fractions
hard
elastic
plastic
adhesive

TRT- R Recipe 5, type 2

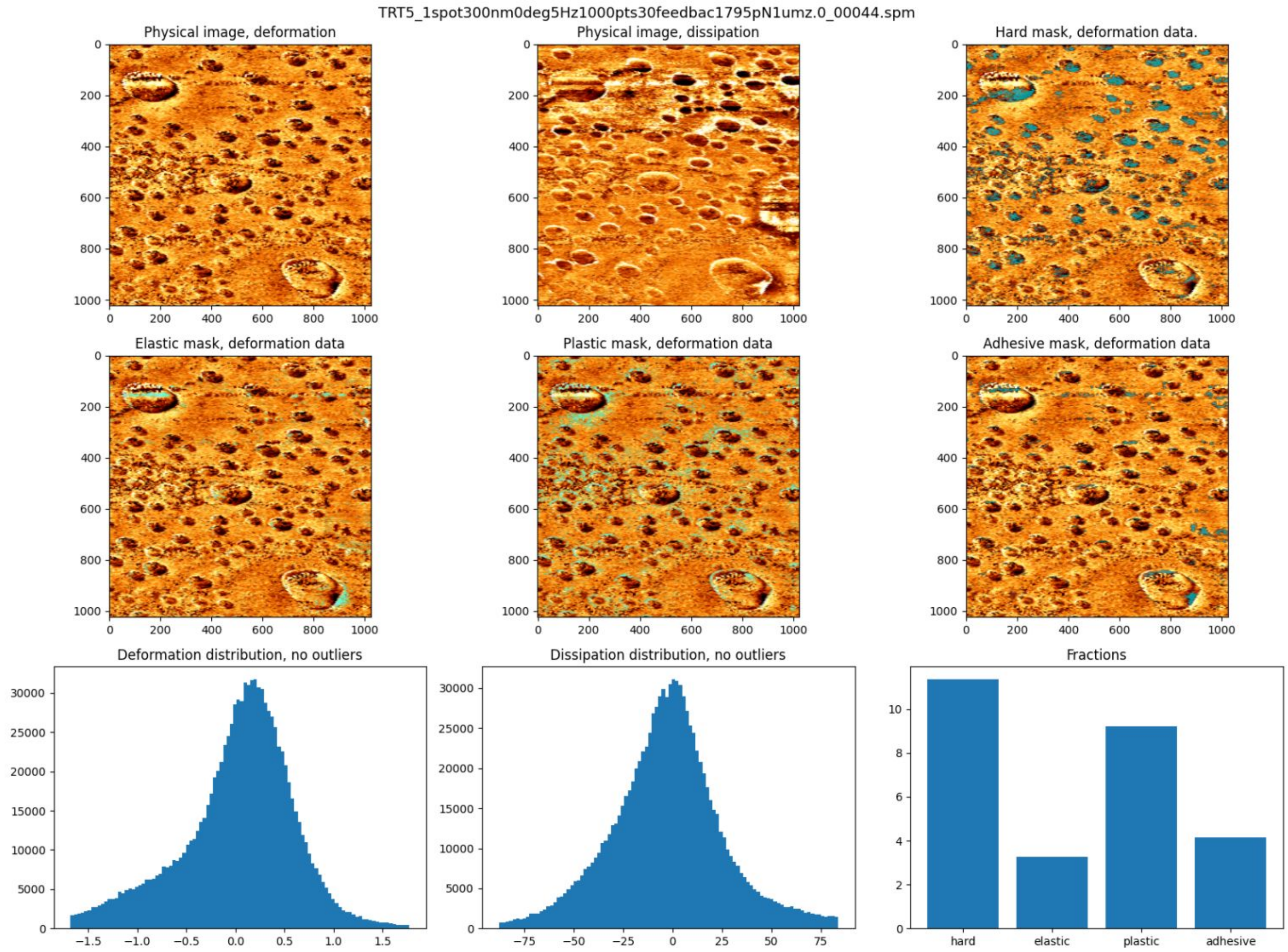

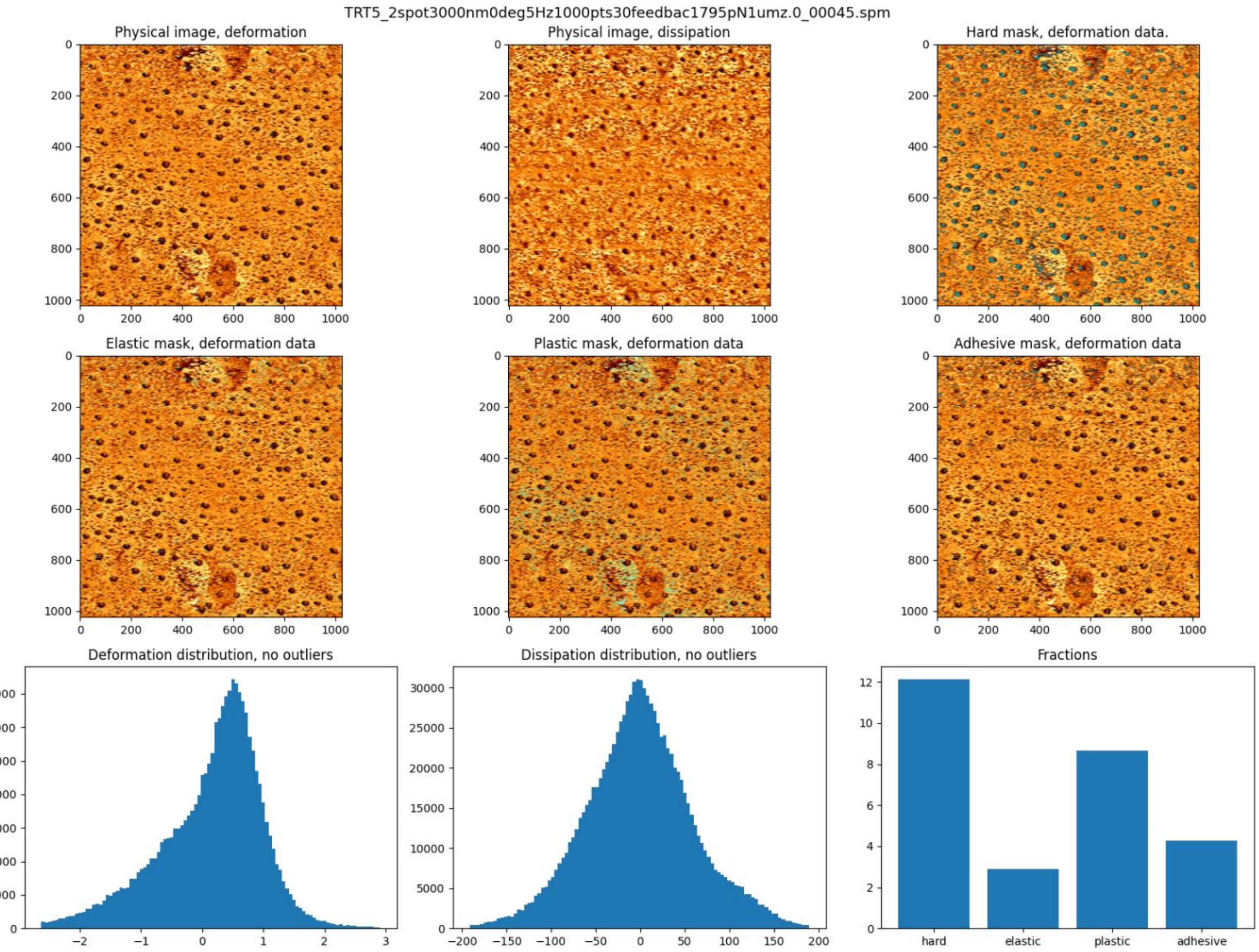

TRT5_2spot3000nm0deg5Hz1000pts30feedbac1795pN1umz.0_00045.spm
Physical image, deformation
Physical image, dissipation
Hard mask, deformation data.
Elastic mask, deformation data
Plastic mask, deformation data
Adhesive mask, deformation data
Deformation distribution, no outliers
Dissipation distribution, no outliers
Fractions
hard
elastic
plastic
adhesive

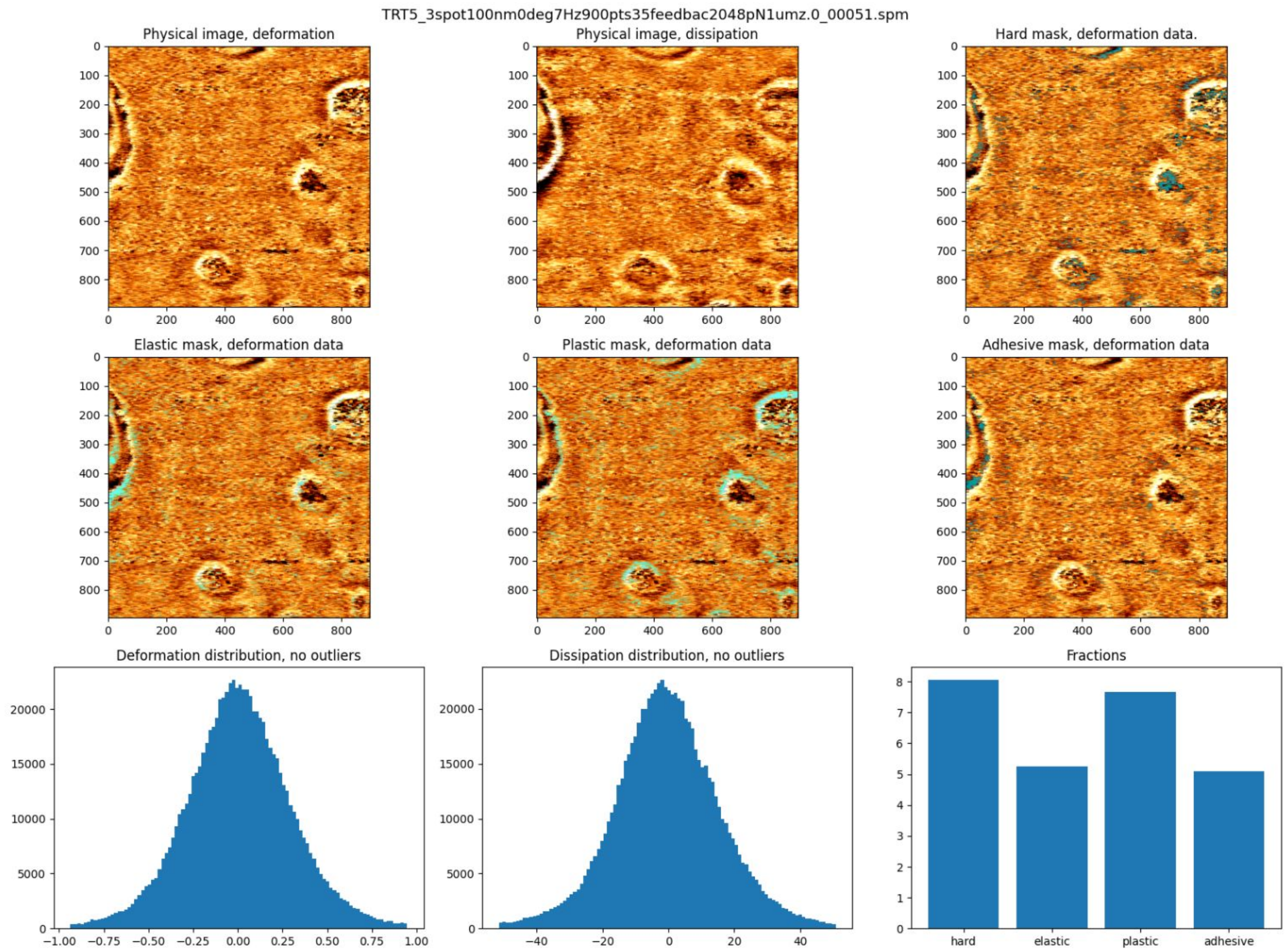
TRT5_3spot100nm0deg7Hz900pts35feedbac2048pN1umz.0_00051.spm
Physical image, deformation
Physical image, dissipation
Hard mask, deformation data.
Elastic mask, deformation data
Plastic mask, deformation data
Adhesive mask, deformation data
Deformation distribution, no outliers
Dissipation distribution, no outliers
Fractions
hard
elastic
plastic
adhesive

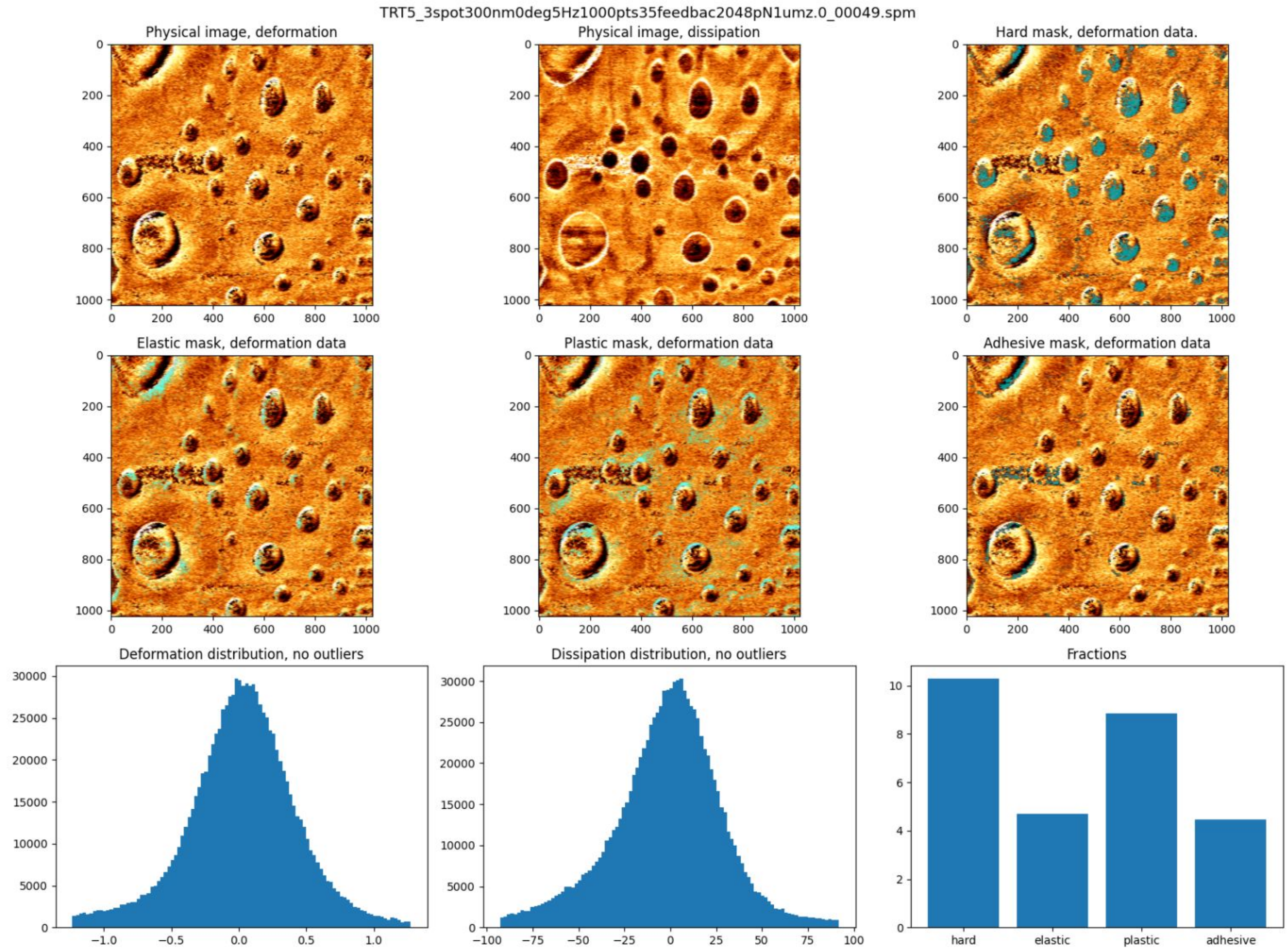
TRT5_3spot300nm0deg5Hz1000pts35feedbac2048pN1umz.0_00049.spm
Physical image, deformation
Physical image, dissipation
Hard mask, deformation data.
Elastic mask, deformation data
Plastic mask, deformation data
Adhesive mask, deformation data
Deformation distribution, no outliers
Dissipation distribution, no outliers
Fractions
hard
elastic
plastic
adhesive

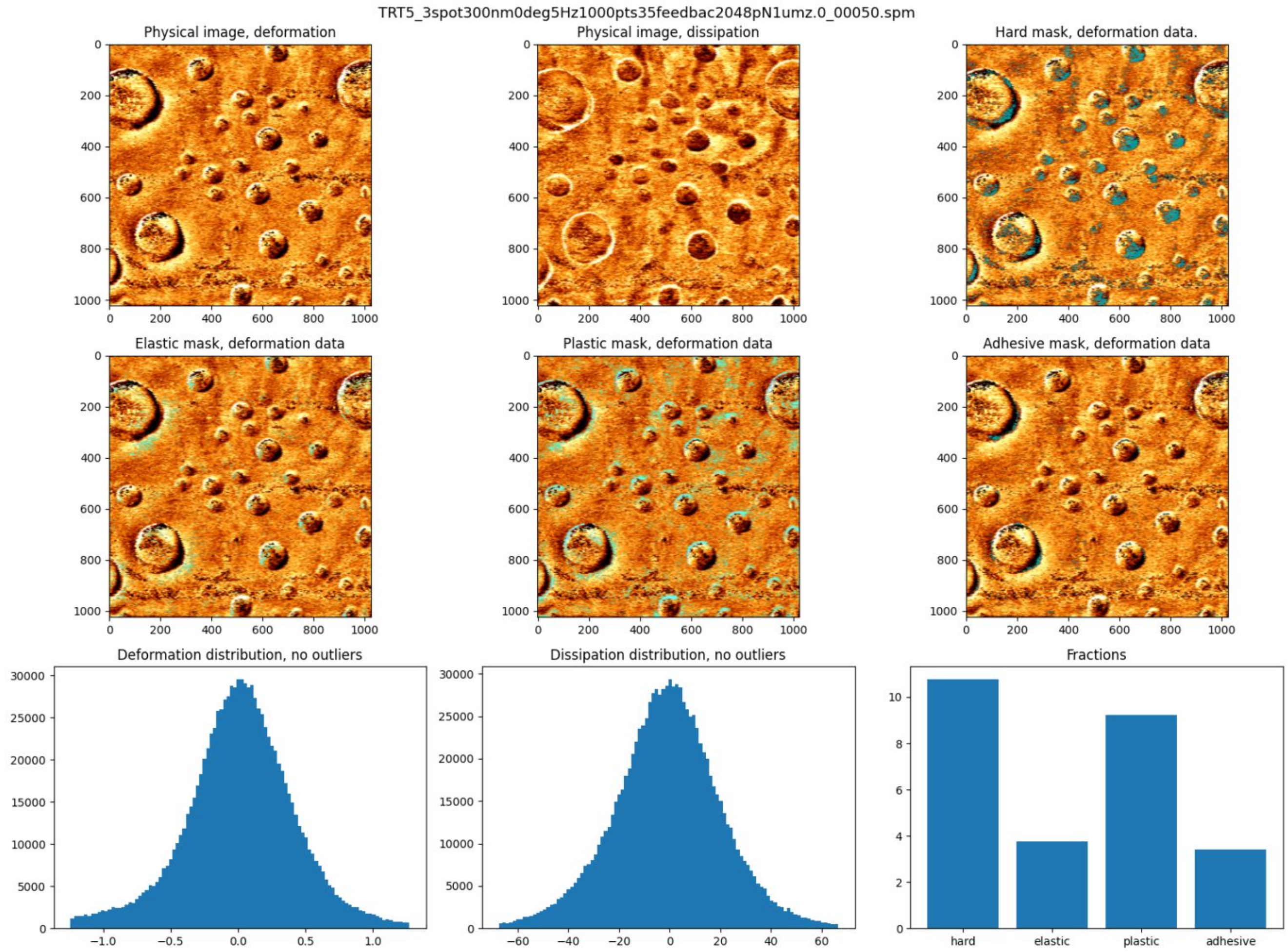
TRT5_3spot300nm0deg5Hz1000pts35feedbac2048pN1umz.0_00050.spm
Physical image, deformation
Physical image, dissipation
Hard mask, deformation data.
Elastic mask, deformation data
Plastic mask, deformation data
Adhesive mask, deformation data
Deformation distribution, no outliers
Dissipation distribution, no outliers
Fractions
hard
elastic
plastic
adhesive

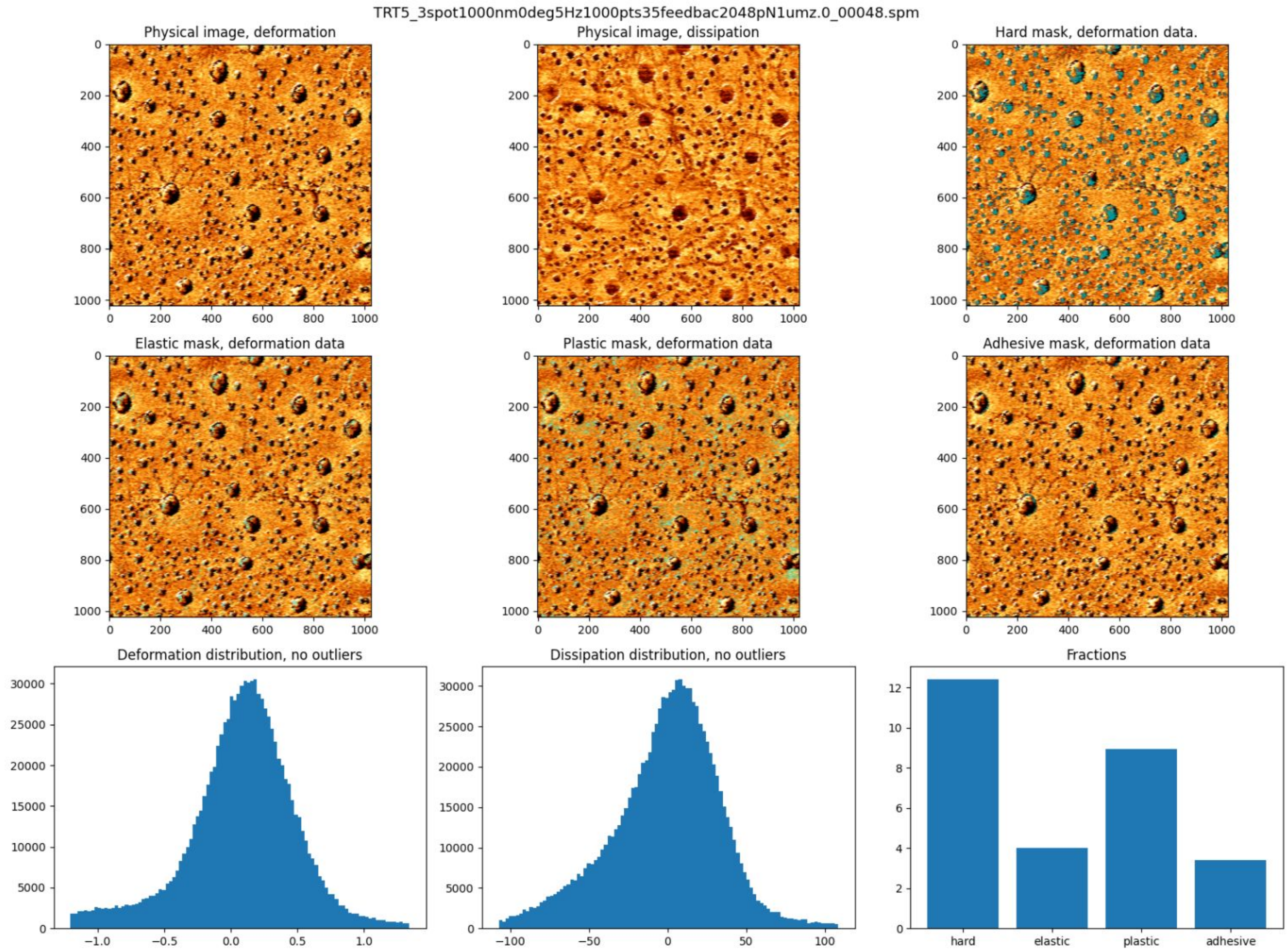

TRT5_3spot1000nm0deg5Hz1000pts35feedbac2048pN1umz.0_00048.spm
Physical image, deformation
Physical image, dissipation
Hard mask, deformation data.
Elastic mask, deformation data
Plastic mask, deformation data
Adhesive mask, deformation data
Deformation distribution, no outliers
Dissipation distribution, no outliers
Fractions
hard
elastic
plastic
adhesive

TRT- R Recipe 6, type 1

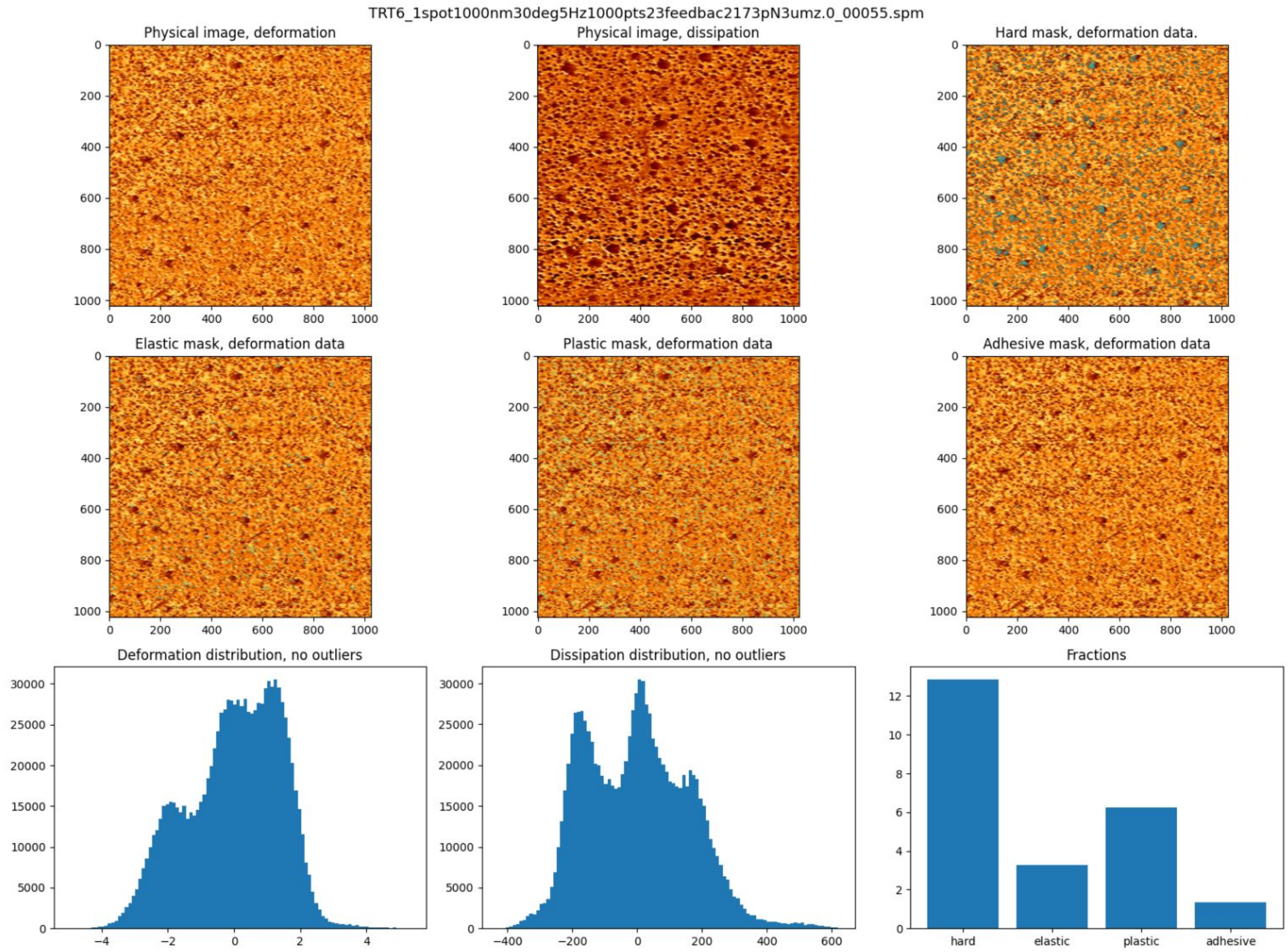

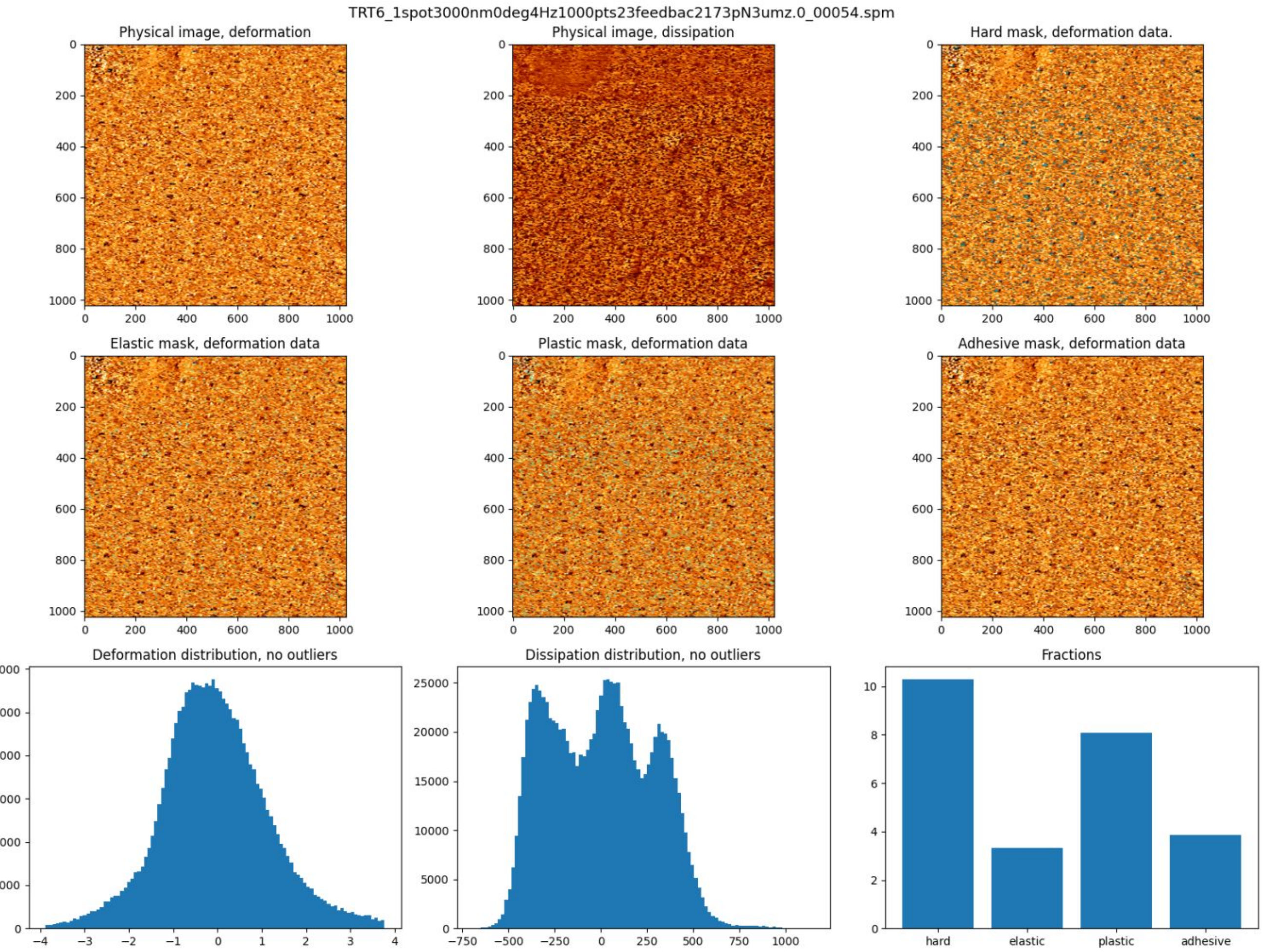
TRT6_1spot3000nm0deg4Hz1000pts23feedbac2173pN3umz.0_00054.spm
Physical image, deformation
Physical image, dissipation
Hard mask, deformation data.
Elastic mask, deformation data
Plastic mask, deformation data
Adhesive mask, deformation data
Deformation distribution, no outliers
Dissipation distribution, no outliers
Fractions
hard
elastic
plastic
adhesive

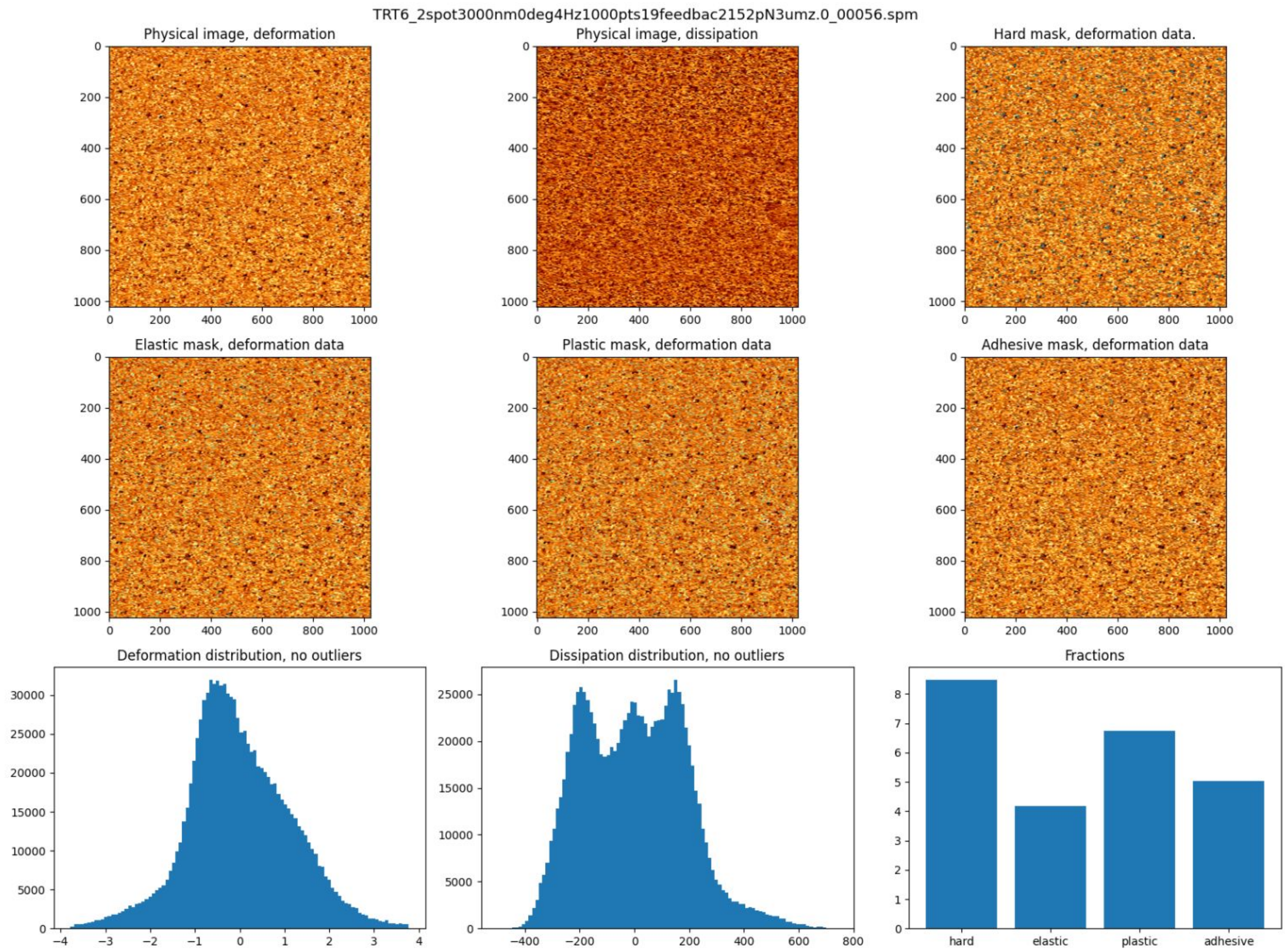
TRT6_2spot3000nm0deg4Hz1000pts19feedbac2152pN3umz.0_00056.spm
Physical image, deformation
Physical image, dissipation
Hard mask, deformation data.
Elastic mask, deformation data
Plastic mask, deformation data
Adhesive mask, deformation data
Deformation distribution, no outliers
Dissipation distribution, no outliers
Fractions
hard
elastic
plastic
adhesive

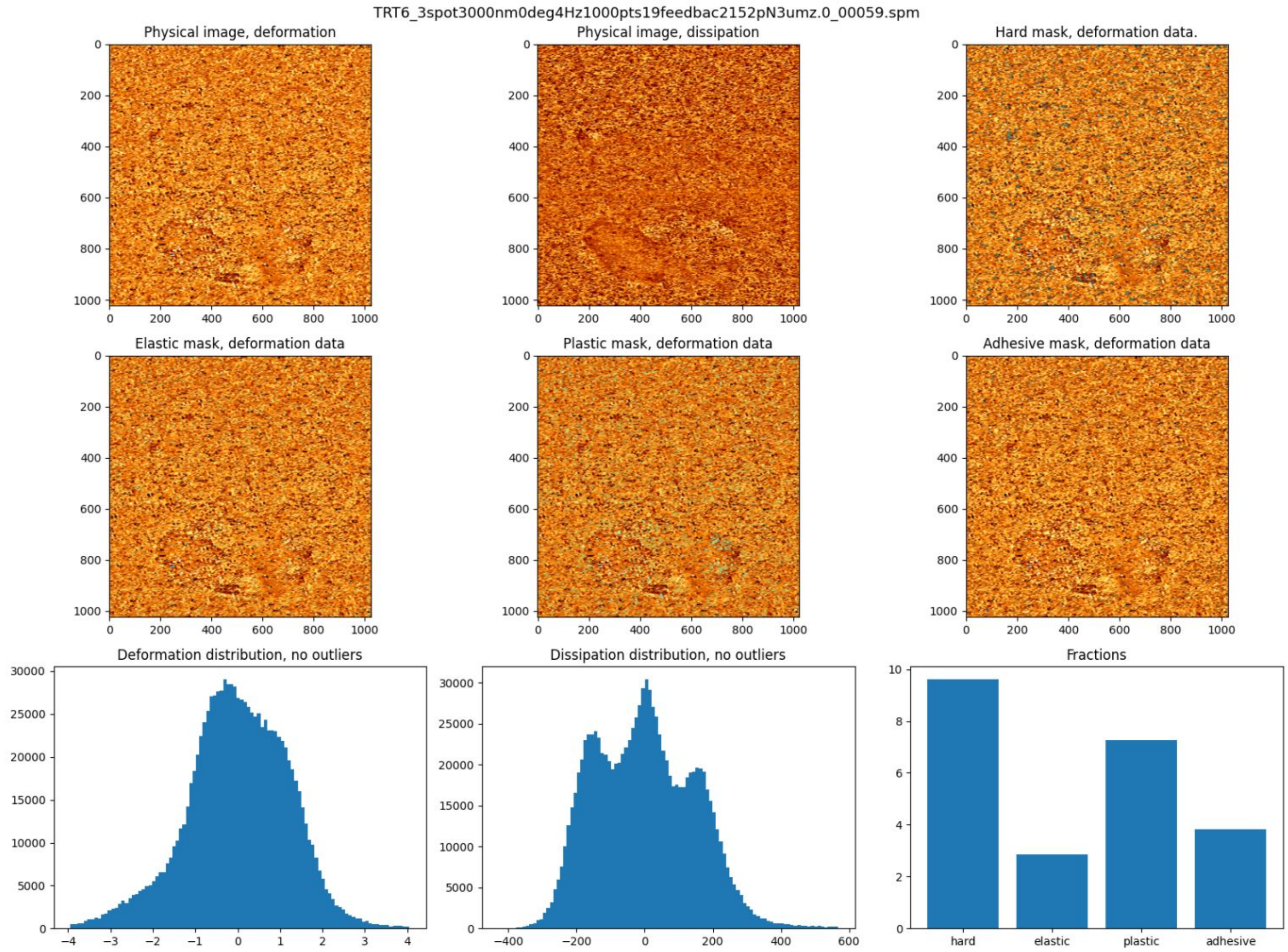
TRT6_3spot3000nm0deg4Hz1000pts19feedbac2152pN3umz.0_00059.spm
Physical image, deformation
Physical image, dissipation
Hard mask, deformation data.
Elastic mask, deformation data
Plastic mask, deformation data
Adhesive mask, deformation data
Deformation distribution, no outliers
Dissipation distribution, no outliers
Fractions
hard
elastic
plastic
adhesive

TRT- R Recipe 6, type 2

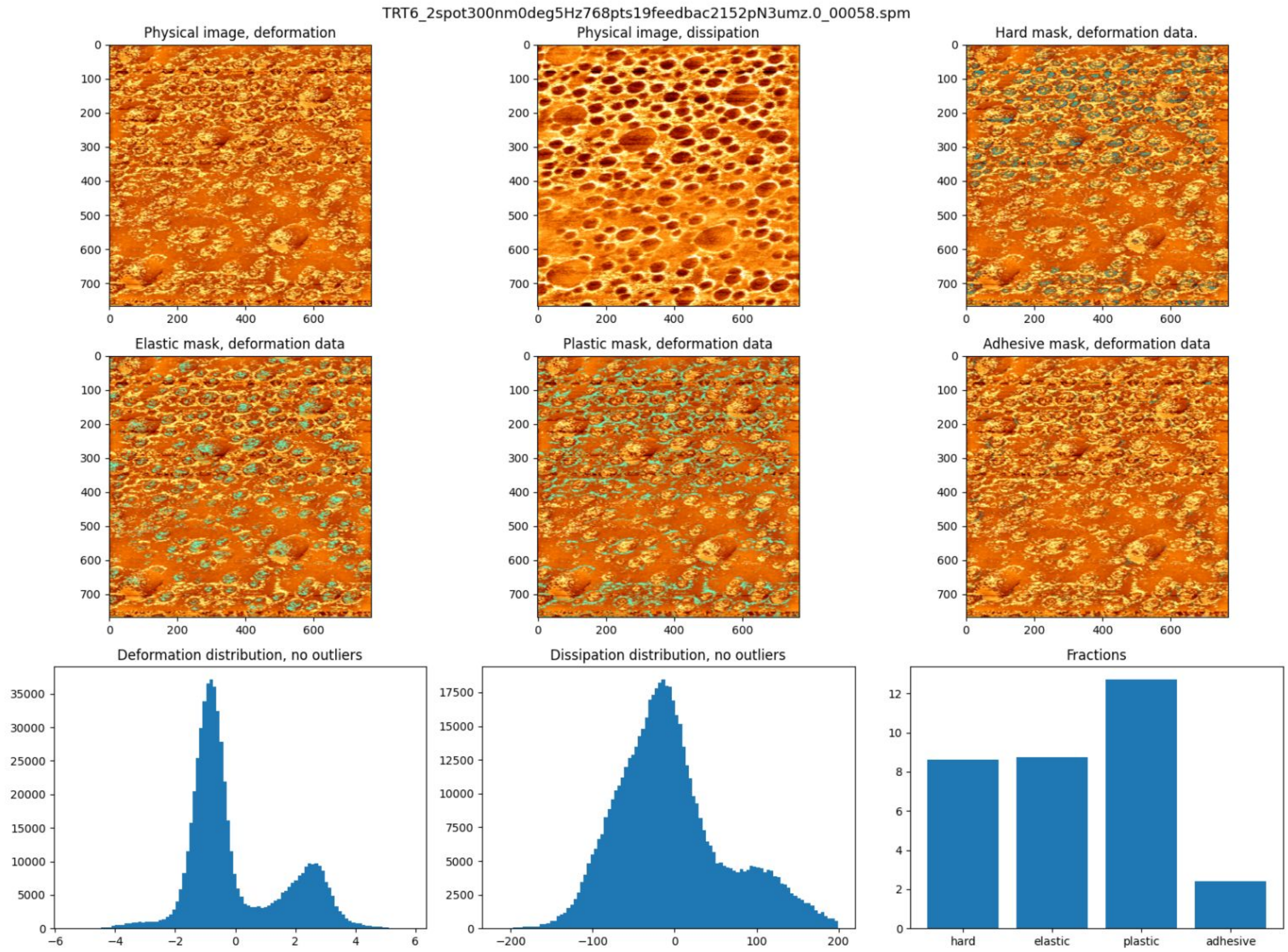

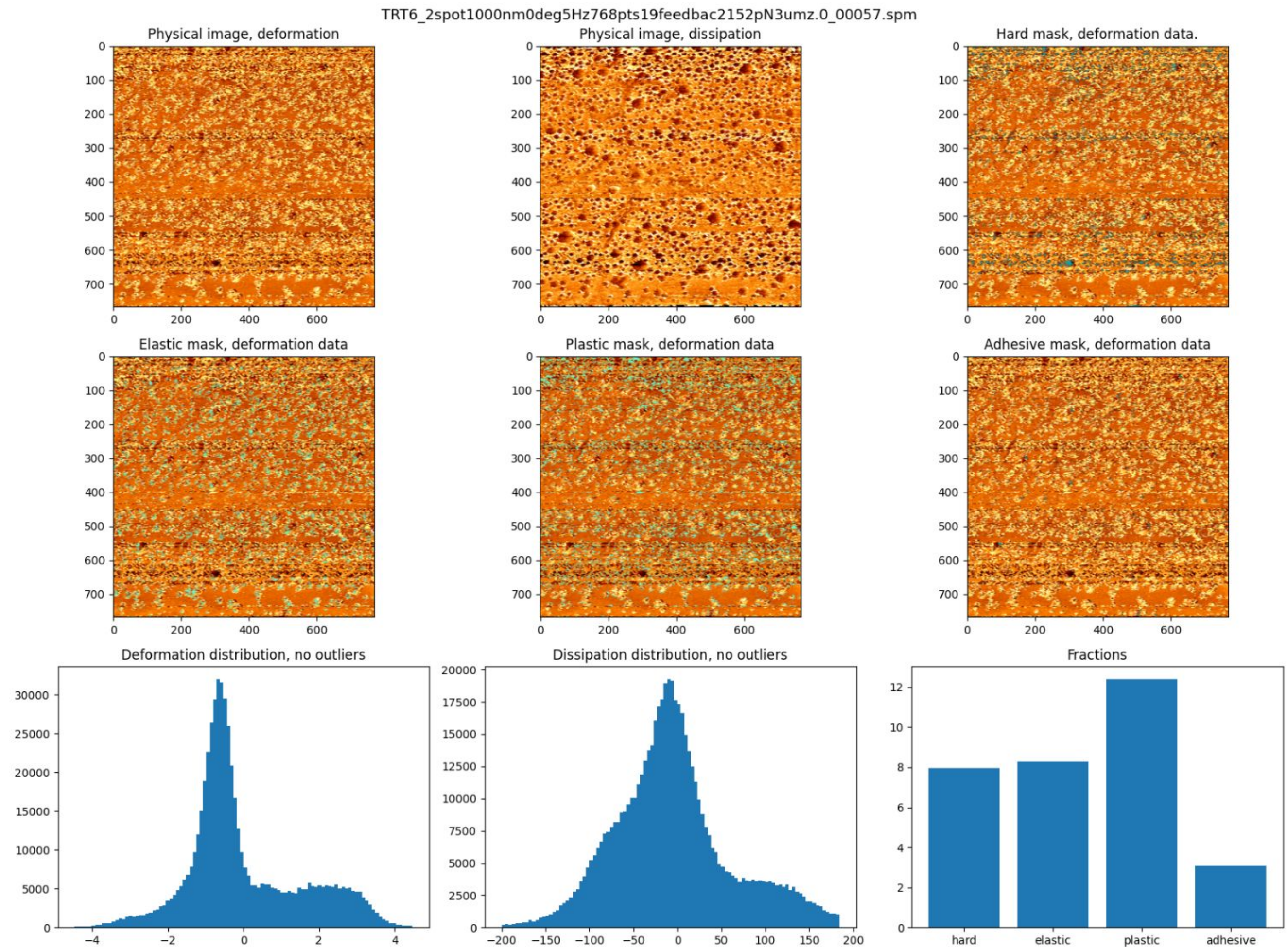
TRT6_2spot1000nm0deg5Hz768pts19feedbac2152pN3umz.0_00057.spm
Physical image, deformation
Physical image, dissipation
Hard mask, deformation data.
Elastic mask, deformation data
Plastic mask, deformation data
Adhesive mask, deformation data
Deformation distribution, no outliers
Dissipation distribution, no outliers
Fractions
hard
elastic
plastic
adhesive

TRT- R Recipe 6, type 3

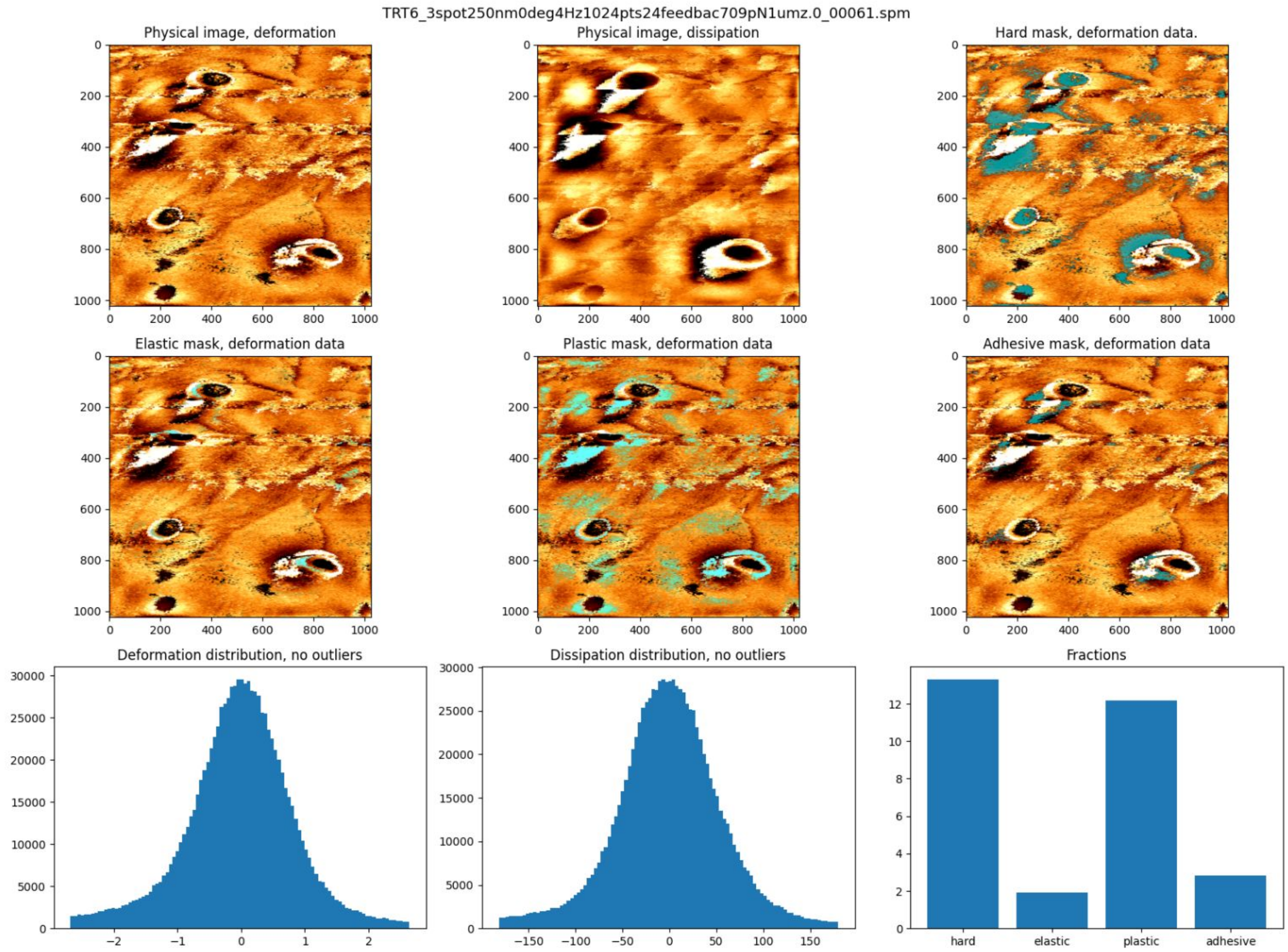

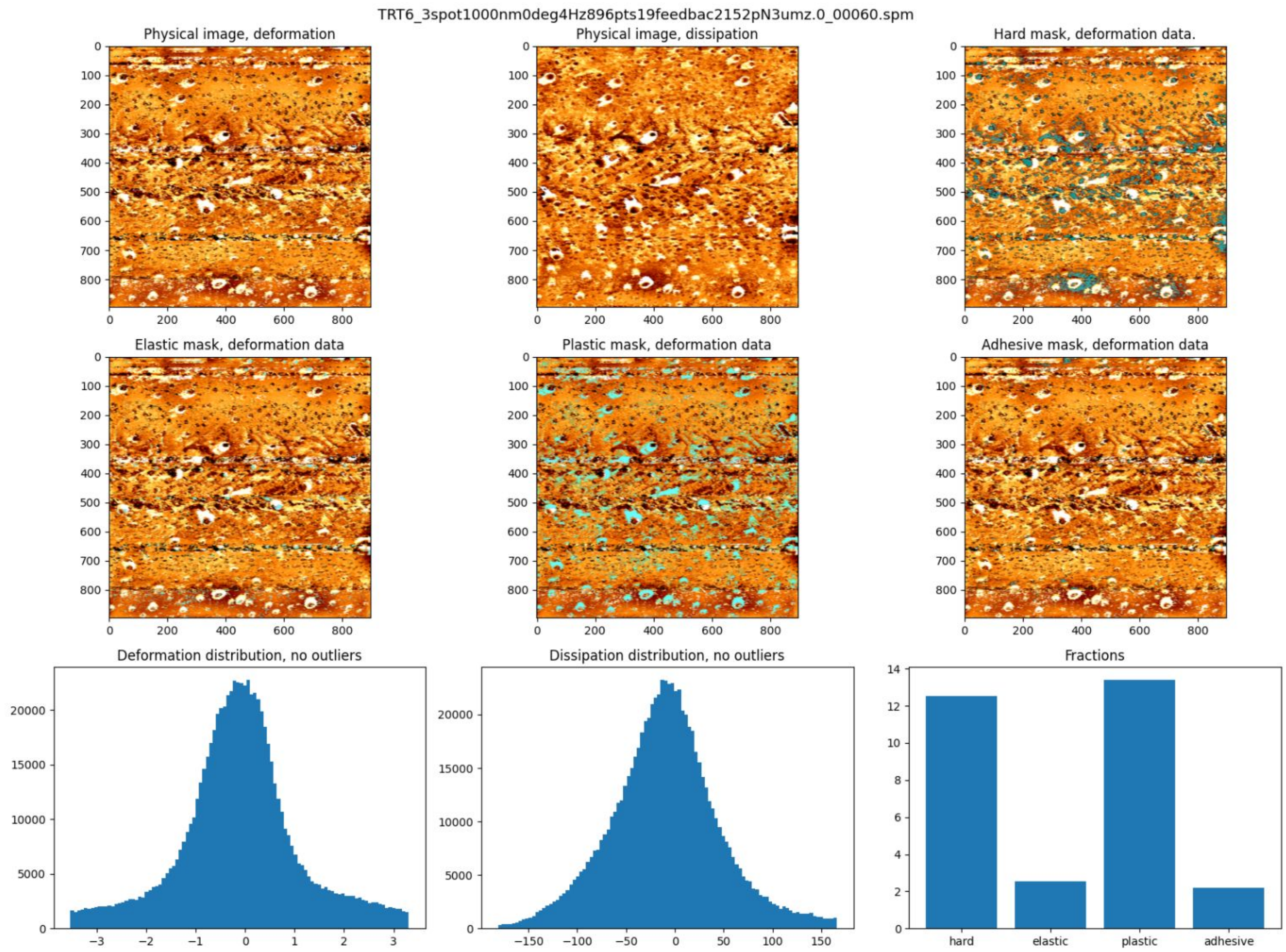

TRT6_3spot1000nm0deg4Hz896pts19feedbac2152pN3umz.0_00060.spm
Physical image, deformation
Physical image, dissipation
Hard mask, deformation data.
Elastic mask, deformation data
Plastic mask, deformation data
Adhesive mask, deformation data
Deformation distribution, no outliers
Dissipation distribution, no outliers
Fractions
hard
elastic
plastic
adhesive
